# Complete genome and comparative analysis of the chemolithoautotrophic bacterium ***Oligotropha carboxidovorans ***OM5

**DOI:** 10.1186/1471-2164-11-511

**Published:** 2010-09-23

**Authors:** Debarati Paul, Susan M Bridges, Shane C Burgess, Yoginder S Dandass, Mark L Lawrence

**Affiliations:** 1 College of Veterinary Medicine, Mississippi State University, Mississippi State, Mississippi, USA; 2 Institute for Digital Biology, Mississippi State University, Mississippi State, Mississippi, USA; 3 Life Sciences and Biotechnology Institute, Mississippi State University, Mississippi State, Mississippi, USA; 4 Department of Computer Science and Engineering, Mississippi State University, Mississippi State, Mississippi, USA

## Abstract

**Background:**

*Oligotropha carboxidovorans *OM5 T. (DSM 1227, ATCC 49405) is a chemolithoautotrophic bacterium capable of utilizing CO (carbon monoxide) and fixing CO_2 _(carbon dioxide). We previously published the draft genome of this organism and recently submitted the complete genome sequence to GenBank.

**Results:**

The genome sequence of the chemolithoautotrophic bacterium *Oligotropha carboxidovorans *OM5 consists of a 3.74-Mb chromosome and a 133-kb megaplasmid that contains the genes responsible for utilization of carbon monoxide, carbon dioxide, and hydrogen. To our knowledge, this strain is the first one to be sequenced in the genus *Oligotropha*, the closest fully sequenced relatives being *Bradyrhizobium *sp. BTAi and USDA110 and *Nitrobacter hamburgiensis *X14. Analysis of the *O. carboxidovorans *genome reveals potential links between plasmid-encoded chemolithoautotrophy and chromosomally-encoded lipid metabolism. Comparative analysis of *O. carboxidovorans *with closely related species revealed differences in metabolic pathways, particularly in carbohydrate and lipid metabolism, as well as transport pathways.

**Conclusion:**

*Oligotropha*, *Bradyrhizobium *sp and *Nitrobacter hamburgiensis *X14 are phylogenetically proximal. Although there is significant conservation of genome organization between the species, there are major differences in many metabolic pathways that reflect the adaptive strategies unique to each species.

## Background

OM5, the type strain of the gram-negative bacterium *Oligotropha carboxidovorans *[1,2] (originally called *Pseudomonas carboxidovorans*), was isolated from soil of waste water sewage treatment settling ponds. OM5 is capable of chemolithoautotrophic growth on CO, CO_2_, and H_2 _[3-5] and can utilize chemoorganoheterotrophic substrates under aerobic conditions, including the salts of pyruvate, formate, glyoxylate, lactate, ascorbate, malate, oxoglutarate, acetate, and oxalate.

Initial 16 S rDNA sequencing [5] showed *Oligotropha carboxidovorans *is phylogenetically close to two Bradyrhizobiaceae: *Bradyrhizobium *sp. BTAi and *Nitrobacter hamburgiensis *X14. All three species share some common gene(s) and operons; however, these three bacteria vary greatly in metabolism. Together they are an excellent model for understanding how closely related bacteria adapt to very different environments.

In addition to its utility for comparative genomics, *O. carboxidovorans *genes may have practical utility for bioenergy production. *O. carboxidovorans *is capable of utilizing syngas, which is a mixture of CO, CO_2_, and H_2 _that results from gasification of organic wastes, for chemolithoautotrophic growth. Microbial fermentation of this gas mixture could serve as a source for biofuels. Many of the chemolithoautotrophy genes in *O. carboxidovorans *are located on a megaplasmid and have been studied [2,4,6,7]. The *O. carboxidovorans *genome will allow further studies to understand how they are regulated and how fixed carbon is assimilated into its metabolism, which could enhance use of syngas as a biofuel source.

We previously announced the *O. carboxidovorans *genome sequence [8]. At the time of our previous announcement, the chromosome sequence was in two large contigs. We now report completion of the *O. carboxidovorans *OM5 genome and comparative genomics analysis with *Bradyrhizobium *sp. BTAi and *N. hamburgiensis *X14. Central intermediary metabolism is similar in *O. carboxidovorans*, *Bradyrhizobium *sp. BTAi, and *N. hamburgiensis *X14, and *O. carboxidovorans *shares other features such as iron transport systems. However, *O. carboxidovorans *also has several unique features, particularly in fatty acid oxidation and glyoxylate cycle.

## Results and discussion

### Genome organization

Closure of the last two gaps produced a 3,745,629 bp long circular OM5 chromosome (62.4% GC) with 3782 predicted genes. The vast majority (3722) are protein coding; 48 encode RNAs, and 12 are predicted as pseudogenes. Of the 48 RNA genes, three are rRNA genes (one rRNA operon is present), and the rest code for tRNAs. Twelve percent of the genome is predicted to be intergenic DNA. Of the OM5 protein coding genes, TIGR role categories were assigned to 87.59%. Of these, 72.01% had a definitive functional assignment, and 1.31% did not have a role category. In addition, 15.99% were identified as conserved hypothetical proteins (CHPs), and 10.67% were designated as hypothetical proteins (HPs). Transporter and enzyme functions are the most abundant major metabolic activities in the OM5 genome, together making up 10.5% of the predicted coding capacity. Table 1 shows predicted functional classifications (COG groups) of *O. carboxidovorans *proteins.

**Table 1:** Number of genes per COG group in the *O. carboxidovorans *genome.

Process	Description	Class ID	CDS No
Cellular Process and Signaling	Cell cycle control, cell division and chromosome partitioning	D	37
Cellular Process and Signaling	Cell wall/membrane/envelope biogenesis	M	205
Cellular Process and Signaling	Cell motility	N	90
Cellular Process and Signaling	Posttranslational modification, protein turnover, chaperones	O	172
Cellular Process and Signaling	Signal transduction mechanisms	T	189
Cellular Process and Signaling	Intracellular trafficking, secretion and vesicular transport	U	117
Cellular Process and Signaling	Defense mechanisms	Y	99
Cellular Process and Signaling	Extracellular structures	W	1
Information storage and processing	Chromatin structure and dynamics	B	1
Information storage and processing	Translation, ribosome structure and biogenesis	J	193
Information storage and processing	Transcription	K	205
Information storage and processing	Replication, recombination and repair	L	175
Metabolism	Energy production and conversion	C	236
Metabolism	Amino acid transport and metabolism	E	444
Metabolism	Nucleotide transport and metabolism	F	79
Metabolism	Carbohydrate transport and metabolism	G	207
Metabolism	Coenzyme transport and metabolism	H	137
Metabolism	Lipid transport and metabolism	I	150
Metabolism	Inorganic ion transport and metabolism	P	345
Metabolism	Secondary metabolites biosynthesis, transport and catabolism	Q	112
Poorly characterized	General function prediction only	R	514
Poorly characterized	Function unknown	S	243

Using RfamScan http://rfam.sanger.ac.uk/[9] in Oligotroscope, we found 10 potential non-coding RNA sequences, which includes small RNA (sRNA) (Table 2). Prediction of prokaryotic sRNAs has limitations because their sequences are diverse; therefore, identification is based on conserved sequence features in the intergenic regions (promoters, terminators, and RNA secondary structures). In several bacteria, sRNAs have important regulatory functions, including regulation of outer membrane protein expression and regulating stress responses (e.g., oxidative stress, SOS/DNA damage, cold shock, and iron stress). Some sRNAs such as DsrA and RprA are translational activators [10-12]. The regulatory RNA SsrA is needed for correct high-level translation of RpoS [13].

**Table 2:** Predicted *O. carboxidovorans *non-coding RNAs

Label	Begin	End	Length	Name	Product
misc_RNA_16	925123	925197	75	suhB	Antisense RNA/nc RNA
misc_RNA_19	1416092	1416176	85	ssrA	10Sa RNA SsrA
misc_RNA_18	1416179	1416292	114	ssrA	10Sa RNA SsrA
misc_RNA_7	1574853	1575108	256	csrC	Regulatory sRNA
misc_RNA_8	2702642	2702856	215	csrC	Regulatory sRNA
misc_RNA_20	2988271	2988368	98	TPP	Riboswitch
misc_RNA_2	3118145	3118195	51	serC	Attenuator
misc_RNA_1	3263978	3264135	158	ssrS	6 S regulatory RNA
misc_RNA_21	3502780	3502877	98	SRP	Bacterial signal recognition particle
misc_RNA_9	3606055	3606129	75	ctRNA	Counter-transcribed RNA

**Figure 1:**
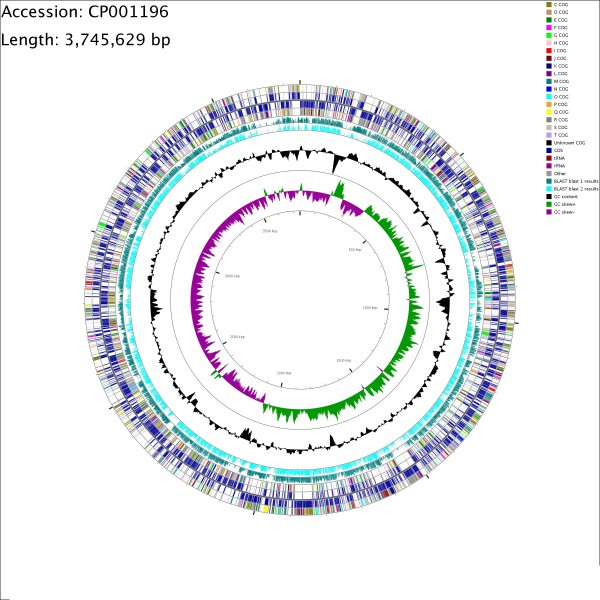
**Circular representation of the *O. carboxidovorans *OM5 genome**. Circles display (from inside): genomic position in kilobases, GC-skew, GC content, BLASTX results with strain *B. japonicum *strain USDA110 (blast 2), BLASTX results with *N. hamburgensis *strain X14 (blast 1), predicted protein-coding sequences (CDS) on the reverse strand, and predicted CDS on the forward strand. Protein-coding sequences on the outer two circles are colored according to predicted COG (clusters of orthologous groups) functional categories. The single letter COG group identifications are described in Table 2.

GC and AT skew analysis http://tubic.tju.edu.cn/Ori-Finder/ suggests a putative replication origin (spanning 150 nt) starting at position 357,583 [14]. The origin has a strong inflection point in the coding strand with 4 DnaA box motifs (tgtttcacg). This motif was identified using search parameters that allowed no more than one mismatch compared to the perfect *E. coli *DnaA box (ttatccaca) (see Figure 1; constructed using CG View) [15].

The final sequences discussed in this paper (GenBank: NC_011386; tax ID 504832) are available at Comprehensive Microbial Resource (CMR) (cmr.jcvi.org/cgi-bin/CMR), Integrated Microbial Genomes (IMG) http://img.jgi.doe.gov/cgi-bin/pub/main.cgi, KEGG, and Genoscope (Oligotroscope).

### Comparative genomics

*O. carboxidovorans*, *N. hamburgensis*, and *Bradyrhizobium *spp. have contrasting physiologies and ecological niches. *N. hamburgensis *is ubiquitous in nature; it has been isolated from soil, building sandstone, and sewage sludge. It is similar to *O. carboxidovorans *in that it is a facultatively lithoautotrophic bacterium, but it differs from *O. carboxidovorans *by using nitrite to nitrate oxidation (nitrification) as its autotrophic growth energy source. *N. hamburgensis *can also grow mixotrophically with NO_2 _and organic components or heterotrophically using only organic compounds [16,17]. Its current biotechnology applications include efficient transformation of fertilizer nitrogen into readily usable forms (such as nitrates) and nitrogen removal during wastewater treatment. *B. japonicum *USDA110 is a nitrogen-fixing bacterium that develops a symbiotic relationship with the soybean plant *Glycine max *by establishing root nodules [18]. Such symbiotic relations are agriculturally important as they obviate the need for expensive and environmentally damaging fertilizer. *Bradyrhizobium sp*. BTAi1 is a photosynthetic rhizobium that is also capable of nitrogen fixation, the first known example of such an organism [19]. It can form stem and root nodules.

**Figure 2:**
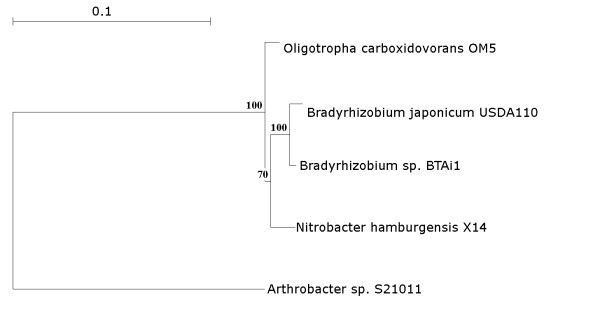
**Phylogenetic tree on the basis of 16 S rRNA genes for *O. carboxidovorans *OM5, *B. japonicum *USDA110, *Nitrobacter hamburgensis *X14, and *Bradyrhizobium *sp. BTAi1 where *Arthrobacter *was used as outgroup**. The tree was created using Treecon. Bootstrapping is shown for values 70% and above. The tree is based on distance matrix where 0.1 means 10% difference between two sequences.

The OM5 16 S rDNA genes are most similar to those of *Nitrobacter hamburgensis *X14, followed by *Bradyrhizobium *sp. strains USDA110 and BTAi1 (Figure 2). The OM5 genome is also more syntenic to *N. hamburgensis *X14 than *Bradyrhizobium *(Table 3). Thus, overall gene synteny substantiates the rDNA phylogeny, but regional synteny between the three strains varies considerably. Synteny is well conserved between the three species in regions containing genes that encode conserved functions, such as ABC transporters, heavy metal binding and transporter proteins, ribosomal proteins, and metabolic pathways (amino acid metabolism, TCA cycle, flagellar proteins, chaperones, etc.). By contrast, synteny is less conserved in genes encoding some of the hypothetical proteins, and this difference is likely due to specific evolutionary adaption. A comparison of general genome features shows considerable variation in genome size in the three species, but otherwise the three species are similar (Table 4).

**Table 3:** Putative orthology and synteny relations between *O. carboxidovorans *and other closely related genomes

Strain	CDS Number*	Percent BBH†	Number of syntons‡	CDS in syntons (%)
*Bradyrhizobium japonicum *USDA110	8317	29.79	501	28.9
*Bradyrhizobium *sp. BTAi1	7394	33.35	467	31.82
*Bradyrhizobium *sp. ORS278	6717	35.22	394	31.40
*Nitrobacter hamburgensis *X14	3804	58.20	288	52.29
*Nitrobacter winogradskyi Nb-255*	3122	66.18	264	60.28
*Rhodopseudomonas palustris *CGA009	4813	47.85	391	43.74
*Rhodopseudomonas palustris *HaA2	4683	66.18	365	60.28
*Azorhizobium caulinodans *ORS571	4717	43.31	484	34.24
*Xanthobacter autotrophicus *Py2	4746	42.96	521	36.98
*Methylobacterium populi *BJ001	5314	39.14	457	27.31
*Oligotropha carboxidovorans *OM5	3722	99.97	1	100

*Number of coding sequences (CDS) encoded in each respective genome

†Percent bidirectional best hits (BBH) in each respective species compared to *O. carboxidovorans*. BBH are defined as gene couples encoding proteins that are considered orthologous based on either reciprocal best BLAST hits or satisfying a BLASTp alignment threshold (a minimum of 35% sequence identity over 80% of the length of the smaller protein).

‡The number of syntons shared between each respective species and *O. carboxidovorans*. A synton is a conserved gene cluster (synteny group) shared between two bacterial genomes. All possible kinds of chromosomal rearrangements are allowed (inversion, insertion/deletion). The gap parameter, representing the maximum number of consecutive genes that are not involved in a synteny group, was set to five genes.

**Table 4:** Comparison of general features between *O. carboxidovorans *OM5, *B. japonicum *USDA110, and *N. hamburgensis *X14

	OM5	USDA110	X14
Size (Mb)	3745629	9105828	4406967
GC%	62.39	64.05	61.7
% Coding	87.59	86.59	83.8
rRNA	3	3	3
tRNA	48	51	55
Genes not assigned role category	1.31%	25.87%	1.33%
Genes assigned role category	72.01%	47.66%	57.94%
Total genes	3738	10740	4853

Although these three genera have different physiologies, they show significant homology at the protein level. *O. carboxidovorans *OM5 shares 1148 orthologous proteins (bidirectional best hits (BBHs) with identity ≥ 70%) [20] with *N. hamburgensis *X14, and it shares 1157 with *B. japonicum *USDA110. Comparing all three strains using the same criteria, there are 953 proteins that have orthologs in all three strains [Additional file 1]. Many of these are "housekeeping" proteins involved in DNA metabolism, repair, protein synthesis, central metabolic pathways, and chaperones/heat shock proteins. Many of the flagellar proteins and proteins responsible for nitrogen uptake and regulation had orthologs in all three species. Membrane proteins involved in uptake of cations and heavy metal ions (such as CusA (OCAR_4024) and CzcA-like proteins (OCAR_4741, OCAR_4871, OCAR_6437, OCAR_6658, OCAR_7479, OCAR_7726, and OCAR_7738)) also had orthologs in all three species.

On the other hand, 1090 *O. carboxidovorans *proteins did not have similarity with any proteins from *B. japonicum *and *N. hamburgensis *(identity < 30%) [21] [Additional file 2]. Most of these proteins are hypothetical; some are phage integrase proteins, and a few are transcriptional regulators or permease genes responsible for transport of ions across the cell membrane. These proteins appear unique to *O. carboxidovorans *and could be interesting for further study to determine their roles in cellular metabolism.

### Mobile and extrachromosomal elements

The *O. carboxidovorans *genome has 49 putative phage or transposon related genes and two prophages as determined by Prophinder. No transposons are present. This number of bacteriophage genes is lower than *N. hamburgensis *strain X14, which has 96 predicted phage-related genes and six prophage genes. High genome plasticity or presence of a large number of mobile elements can indicate the need for rapid changes in response to the environment [22]. For example, the *Pseudomonas fluorescens *Pf-5 genome contains at least 8 large DNA segments that have evolved due to genetic exchanges with other bacteria [23]; these adaptations improved its ability to compete and survive in the dynamic and microbiologically complex rhizosphere. *Shewanella oneidensis *MR-1 also has a large number of mobile elements that provide a means of rapid adaptation [24]. Thus, *O. carboxidovorans *may live in an environment where there is less selective pressure compared to *N. hamburgensis*. By contrast, *B. japonicum *strain USDA110 does not have any predicted prophages (although it has 20 predicted phage-related genes). Genes coding for mobile and extrachromosomal element functions form a distinct and major category only in *N. hamburgensis *and not in the other two species.

### Energy metabolism

Many of the unique features of OM5 energy metabolism enabling it to use CO (carboxidotrophy) or H_2 _(hydrogenotrophy) under aerobic chemolithoautotrophic conditions [25,26] are encoded on the pHCG3 megaplasmid. OM5 central energy metabolism genes are similar to *N. hamburgensis *and *B. japonicum *(as well as other members of Bradyrhizobiaceae). For energy production, OM5 mainly uses NADH-quinone oxidoreductases by oxidative phosphorylation. The chromosome encodes a F-type ATPase that is typical in bacteria and cytochrome c oxidase (cbb3 type), which would be used in various other carbon metabolism pathways. Gene(s) responsible for glycolysis and gluconeogenesis are located on the chromosome; they allow heterotrophic growth on nutrient rich media (tryptic soy broth) and on minimal medium containing acetate [8].

### Carbohydrate metabolism

Genes enabling CO_2 _fixation via the Calvin-Benson-Bassham (CBB) reductive pentose phosphate cycle are located on the megaplasmid [4]. The OM5 chromosome encodes a complete Krebs cycle [Additional file 3] and a complete anaplerotic glyoxylate cycle [Additional file 3]. The glyoxylate cycle functions similar to the Krebs cycle but lacks enzyme steps that generate CO_2_. It is primarily an oxidative pathway that allows synthesis of carbohydrates from acetyl~SCoA, which usually is derived from the oxidation of fatty acids by the β-oxidation pathway. The glyoxylate cycle is found in many β-proteobacteria, including OM5, particularly in organisms that grow well in media in which acetate and other Krebs cycle dicarboxylic acid intermediates are the sole carbon growth source. Compared to *N. hamburgensis*, the OM5 chromosome encodes many gene(s) that participate in glyoxylate and dicarboxylate metabolism alone [Additional file 3]. The difference may reflect the inability of *N. hamburgenesis *to utilize dicarboxylates. *B. japonicum *is capable of catabolizing dicarboxylic acids [16,18]; not surprisingly, it has nearly the same number of gene(s) as *O. carboxidovorans*.

### Fatty acid biosynthesis and oxidation

All three species have enzymes participating in saturated and unsaturated fatty acid and phospholipid synthesis. In OM5, of the 2.54% genes responsible for fatty acid and phospholipid metabolism, 61 code for fatty acid biosynthesis, and 32 code for fat degradation. Genes present in OM5 involved in fatty acid biosynthesis encode orthologs for Acp, AccA, AccB, AccC, AccD, FabD, FabH, FabF, FabG, FabA, FabZ, and FabI [27]. OM5 is capable of generating malonyl-CoA via acetyl-CoA carboxylase (Acc) (OCAR_5979, 5980, 4079) and transferring malonyl-CoA to acyl carrier protein by FabD. It also has genes necessary for fatty acid biosynthesis initiation (*fabH, fabB *and *fabF*), keto group reduction (*fabG*), dehydration (*fabA or fabZ*), and enoyl reduction (*fabI*). As expected, because *O. carboxidovorans*, *N. hamburgensis*, or *B. japonicum *have *fabI*, they do not have *fabK *and *fabL*.

Compared with *N. hamburgensis *or *B. japonicum, O. carboxidovorans *has fewer fatty acid oxidation enzymes [Additional file 3]. In particular, OM5 lacks the genes that degrade long chain fatty acids. For example, *O. carboxidovorans *does not have a gene encoding bll2324 (long-chain-fatty-acid--[acyl-carrier-protein] ligase), which is involved in degradation of long chain fatty acids. This gene is present in *B. japonicum*. Fatty acid methyl ester analysis on strain OM5 indicated an accumulation of some long chain fatty acids when it is grown under different environmental conditions (unpublished results). We believe the absence of these genes in *O. carboxidovorans *may contribute to accumulation of long chain fatty acids under certain conditions.

### Chemolithoautotrophic growth

*O. carboxidovorans *is an autotrophic organism that can grow at the expense of inorganic compounds and use CO_2 _as a carbon source. Its nitrogen comes from inorganic compounds such as NH_3_, NO_3_^-^, or N_2_. During chemolithoautotrophic growth, it derives energy from oxidation of CO to CO_2_. This function is encoded on a megaplasmid encoding the enzyme CO dehydrogenase (*cox *gene cluster). Plasmid-cured mutants (or mutants in which deletions have been introduced into the plasmid) either lose all chemolithoautotrophic growth capabilities or cannot utilize H_2 _plus CO_2 _but retain the ability of using CO plus CO_2 _[4,25,28].

The *N. hamburgensis *genome contains genes that are found in carboxidotrophic bacteria. For example, it has several genes encoding cytochrome b_561_, an important electron transfer component in aerobic carboxidotrophic bacteria [29]. The *N. hamburgensis *genome also has four gene clusters and a lone CDS encoding multiple homologs of molybdopterin-containing carbon monoxide dehydrogenase (Mo-CODH). The largest of these clusters (Nham_2601 to Nham_2608) has high similarity to *B. japonicum *USDA110 and few other species [16]. In fact, the *N. hamburgensis *genome has more copies of Mo-CODH-like genes than it does of nitrite oxidoreductase (membrane bound Mo-dependent enzyme required for converting nitrite to nitrate) [16]. This suggests that these proteins have a vital, but poorly understood, role in the lifestyle of *N. hamburgensis*. *B. japonicum *USDA110 also encodes a Mo-CODH, and it can oxidize CO at the expense of nitrate reduction under anaerobic conditions. However, it does not grow under these conditions [30]. In strain USDA110, anaerobic CO uptake only occurs in the presence of nitrate, which is not a substrate or electron acceptor for anaerobic CODH. Therefore, no carbon fixation occurs [16,31]. Thus, although these two species differ from *O. carboxidovorans *in their chemolithoautotrophic lifestyle, they have similar enzyme(s) for this purpose, particularly CODH.

In *O. carboxidovorans*, the *cox *genes are specifically and coordinately transcribed under chemolithoautotrophic conditions in the presence of CO [26]; therefore, sensing CO is an important function. CO sensors are typically heme-based [32]. Two *O. carboxidovorans *genes encode proteins (CoxC and CoxH) that are putatively associated with CO sensing based on sequence motifs similar to two component regulatory systems [4]. However, the *O. carboxidovorans *sequence upstream of the *cox *cluster does not have genes with unambiguous functions in the sensing of CO or the regulation of *cox *genes. Other species, such as *Azotobacter vinelandii *and *Bradyrhizobium*, contain a gene encoding CooA transcriptional receptor that may be involved in CO sensing and regulating the function of CO dehydrogenase [4]. BLASTP results suggest this protein belongs to the ACAD superfamily, and it shows similarity to acyl-CoA dehydrogenases involved in lipid metabolism. *O. carboxidovorans *has chromosomally encoded acyl-CoA dehydrogenase genes; some of them are directly involved in fatty acid metabolism (OCAR_5221, OCAR_5223, OCAR_5308, OCAR_5791, OCAR_6069, OCAR_6878, and OCAR_6891). Therefore, it is possible that chromosomally encoded lipid metabolism in *O. carboxidovorans *and plasmid-encoded CO fixation are linked, but further studies are needed to confirm this.

### Transport and binding

One tenth of *O. carboxidovorans *genes are responsible for cell motility, binding, and membrane transport. By contrast, 6% of *N. hamburgensis *genes are in this category. This difference likely reflects their contrasting lifestyles. As an environmental species, *O. carboxidovorans *likely has increased need for a diversity of these functions, while the symbiotic species *N. hamburgensis *is in a relatively constant environment.

Iron transport and related proteins are responsible for specific uptake of ferric-siderophore complexes, which are high affinity iron chelators in gram-negative bacteria. *O. carboxidovorans *codes for 3 Fhu gene(s), OCAR_4564, OCAR_4560, and OCAR_4561, that are periplasmic proteins and may function as an ABC-type transport mechanism driven by ATP hydrolysis. The *E. coli fhu *system consists of four genes, designated *fhuA*, *fhuC*, *fhuD *and *fhuB*, that are arranged in one operon [33]. Periplasmic FhuD (31 kDa) and cytoplasmic-membrane-associated FhuC (29 kDa) and FhuB (41 kDa) are proteins necessary for the transport of ferrichrome and other Fe^3+^-hydroxamate compounds (Fe^3+^-aerobactin, Fe^3+^-coprogen) across the cytoplasmic membrane from the periplasm into the cytoplasm [34-36]. The protein complex TonB-ExbB-ExbD [37,38] provides energy for this process. The presence of genes encoding these proteins suggests a functional iron siderophore transport complex in *O. carboxidovorans*.

Iron acquisition is important in bacteria, both as a requirement for growth and as an environmental signal that regulates gene expression [39]. Iron transport is especially important in nitrogen fixing bacteria [40] because it increases fitness in the competitive rhizosphere [41,42]. *Rhizobium leguminosarum *(a symbiotic nitrogen fixing species) contains a *fhuDCB *operon for siderophore uptake [43]. *Bradyrhizobium *also has iron transport systems, but unlike *O. carboxidovorans*, it does not utilize TonB iron transport. The presence of Fe siderophores and transporter genes in non pathogenic, non-nitrogen fixing *O. carboxidovorans *reflects its similarity to nitrogen fixing symbiotic bacteria. The presence of these genes in *O. carboxidovorans *also indicates that its natural environment, like most natural environments, is restricted in iron [40].

Unlike *N. hamburgensis*, *O. carboxidovorans and B. japonicum *have nickel/peptide transporting genes. Other *O. carboxidovorans *transport related proteins (e.g,. oligosaccharide/lipoprotein/amino acid transport related proteins) are also present in *N. hamburgensis *and *B. japonicum*.

## Conclusion

The previously reported sequence of the *O. carboxidovorans *megaplasmid revealed mechanisms for its chemolithoautotrophic growth. The complete O. *carboxidovorans *genome reveals metabolic pathways used during heterotrophic growth and how chemolithoautotrophic growth is linked with central metabolism. Further research is needed to identify genes involved in the regulation of chemolithoautotrophic growth versus heterotrophic growth.

*O. carboxidovorans *appears to be adapted to a relatively stable microenvironment because its genome contains remarkably few phage- or transposon-related genes in comparison to *N. hamburgensis *and other soil bacteria. The lack of any typical Gram-negative communication system based on acylated homoserine lactones (AHLs) also suggests an exclusive microhabitat, similar to *Azoarcus *BH72 [44]. Production of AHLs is not uncommon in environmental bacteria, but it only occurs in some microhabitats under appropriate conditions [45]. Another important characteristic of the *O. carboxidovorans *genome is the presence of TonB-dependent receptors for iron transport across the cell membrane, unlike *N. hamburgensis *and *B. japonicum*. The role of iron acquisition in *O. carboxidovorans *metabolism is another very interesting area for future research.

*O. carboxidovorans*, *N. hamburgensis*, and *B. japonicum *each have unique genetic pathways that enable them to have specialized functions such as CO oxidation, nitrite oxidation, and N_2 _fixation. These genetic pathways have historically been the most well studied from these species. However, comparison of their genomes reveals that there are many metabolic pathways that are conserved between these closely related species. Comparison of these three genomes also revealed previously unknown differences in the "conserved" metabolic pathways. The cumulative effects of these metabolic differences, along with the unique pathways, allow these phylogenetically related species to adapt to diverse environments [46]. Therefore, the unique pathways for these organisms must be understood in the context of their respective genomes. This current analysis is only a beginning, and further work on the function of *O. carboxidovorans *proteins will yield valuable information on how this species survives in its unique environment and how it adapts to both heterotrophic and chemolithoautotrophic lifestyles.

## Methods

### Sequencing methods

*O. carboxidovorans *OM5 (ATCC no. 49405) was grown on carboxydobacterium medium (ATCC no 1789) using acetate as a substrate at 30°C with continuous shaking for 72 h under aerobic conditions. DNA isolation, sequencing, and assembly were conducted as described previously [8]. The assembly at this point had two large contigs. The two gaps were closed by PCR amplification and primer walking. Sequencing reactions were performed to correct a misassembly in the genome. The gaps had G+C rich regions that resulted in early signal loss during sequencing. To circumvent this problem, we used SequenceR_x _Enhancer Solution A (Invitrogen), which enables longer reads on templates containing regions with secondary structure caused by high G+C content, small nucleotide repeats, hairpins, direct repeats, and inverted repeats. Other techniques were used by MWG Biotech (Eurofins) to resolve hard sequencing stops to complete the reads. The complete genome was submitted to NCBI.

Once the complete genome was submitted to NCBI, Comprehensive Microbial Resource (CMR) and Integrated Microbial Genomes (IMG) were used to compare OM5 against all of the publicly available, complete prokaryotic genomes. Orifinder, Prophinder [47]http://aclame.ulb.ac.be/Tools/Prophinder/, and CGVIEW were used to determine the origin of the circular genome, identify prophages, and to obtain a circular graphical representation of the genome.

### Comparative genomics

The *O. carboxidovorans *genome sequence was submitted to Magnifying Genomes Microbial Genome Annotation System (MaGe) [48], which benevolently created Oligotroscope. This website allowed manual annotation as well as orthology and synteny detection of microbial genomes available in NCBI. Putative orthologous relationships between genomes were defined as gene couples satisfying the bidirectional best hit (BBH) criterion or a BLASTP alignment threshold (a minimum of 70% sequence identity over 80% of the length of the smallest protein) [48]. Unique proteins were identified as proteins having <30% sequence identity over 80% of the length. These relationships were subsequently used to search for conserved gene clusters (synteny groups or syntons) among several bacterial genomes using the Syntonizer function. All possible kinds of chromosomal rearrangements were allowed (inversion, insertion/deletion). The gap parameter, representing the maximum number of consecutive genes that are not involved in a synteny group, was set to five genes. Orthology relations were strengthened by synteny detection (conservation of chromosomal co-localization between pairs of orthologous genes from different genomes) using the Phyloprofile synteny option in Oligotroscope. The method used was not restricted to the BBH definition; therefore, it allowed for multiple correspondences between genes (fusion/fission, duplication) and chromosomal rearrangements (inversion, insertion/deletion). Oligotroscope was also used to determine the misc_RNAs in the genome (Table 1).

We determined *O. carboxidovorans-*specific genes (genes encoding proteins where no orthologs were detected in the compared species and not in any synteny group) using the two most closely related species with fully sequenced genomes for comparison, *Bradyrhizobium *sp. (strain USDA110 and BTAi1) and *N. hamburgensis *X14. Cartographic representations in the MaGe web interface allowed us to confirm the manual annotations and detect specific gene(s).

### Phylogenetic tree construction

For tree construction, 16 S rRNA sequences were aligned in ClustalX [49], followed by tree construction using Treecon [50]. Distance estimations were performed in Treecon by Jukes and Cantor method [51]. Tree topology for the 16 S rRNA nucleotide sequences were inferred by the distance matrix using Neighbour Joining method [52].

### Pathway analysis

For pathway analysis, we used the Kyoto Encyclopedia of Genes and Genomes (KEGG) PATHWAY Database, which includes the completed sequence of strain OM5 http://www.genome.jp/kegg-bin/show_organism?org=oca. The KEGG database was also used to trace the enzymes/pathways in *Bradyrhizobium *USDA110 and *N. hamburgensis *X14 and compare with strain OM5.

## Authors' contributions

DP conducted sequencing and closure of the genome as well as annotation and analysis. SMB, SCB, YSD, and MLL conceived and directed the project. SMB and YSD oversaw computational analyses, SCB and MLL oversaw sequencing aspects, and MLL oversaw microbiological aspects. DP wrote the manuscript draft, and all authors edited and approved the final manuscript.

## Supplementary Material

Additional file 1 **Orthologous proteins in *O. carboxidovorans *OM5, *N. hamburgensis *X14, and *Bradyrhizobium *spp. USDA110**. Click here for file

Additional file 2 **Proteins unique to *O. carboxidovorans *OM5 as compared to *N. hamburgensis *X14, and *Bradyrhizobium *spp. USDA110**. Click here for file

Additional file 3 ***O. carboxidovorans *enzyme complexes involved in some metabolic pathways**. Click here for file
