# Supplementary material for: Complete genome and comparative analysis of the chemolithoautotrophic bacterium Oligotropha carboxidovorans OM5
Source: BMC Genomics. 2010 Sep 23;11:511. doi: 10.1186/1471-2164-11-511 (PMC3091675; doi:10.1186/1471-2164-11-511)
Supplement: Additional file 1 — Orthologous proteins in O. carboxidovorans OM5, N. hamburgensis X14, and Bradyrhizobium spp. USDA110. [file 1471-2164-11-511-S1.DOC]

**Additional file 1.** Orthologous proteins in *O. carboxidovorans* OM5, *N. hamburgensis* X14, and *Bradyrhizobium* spp. USDA110 as shown by the Phyloprofile tool of Oligotroscope.

| **Label** | **Gene** | **Product** |
| --- | --- | --- |
| [OCAR_4024](https://www.genoscope.cns.fr/agc/mage/wwwpkgdb/Info/getInfoLabel.php?id=3484063&wwwpkgdb=fc2733d6468c073f1dd738aa598ede55&nocache=62541e24b6e240f187ac920cab60f9da&dir=&wwwpkgdb=fc2733d6468c073f1dd738aa598ede55) | _ | cation efflux system protein CusA |
| [OCAR_4040](https://www.genoscope.cns.fr/agc/mage/wwwpkgdb/Info/getInfoLabel.php?id=3482144&wwwpkgdb=fc2733d6468c073f1dd738aa598ede55&nocache=62541e24b6e240f187ac920cab60f9da&dir=&wwwpkgdb=fc2733d6468c073f1dd738aa598ede55) | _ | hypothetical protein |
| [OCAR_4043](https://www.genoscope.cns.fr/agc/mage/wwwpkgdb/Info/getInfoLabel.php?id=3484080&wwwpkgdb=fc2733d6468c073f1dd738aa598ede55&nocache=62541e24b6e240f187ac920cab60f9da&dir=&wwwpkgdb=fc2733d6468c073f1dd738aa598ede55) | _ | binding-protein-dependent transport systems inner membrane component |
| [OCAR_4045](https://www.genoscope.cns.fr/agc/mage/wwwpkgdb/Info/getInfoLabel.php?id=3484082&wwwpkgdb=fc2733d6468c073f1dd738aa598ede55&nocache=62541e24b6e240f187ac920cab60f9da&dir=&wwwpkgdb=fc2733d6468c073f1dd738aa598ede55) | _ | perplasmic binding protein of ABC transporter |
| [OCAR_4048](https://www.genoscope.cns.fr/agc/mage/wwwpkgdb/Info/getInfoLabel.php?id=3484083&wwwpkgdb=fc2733d6468c073f1dd738aa598ede55&nocache=62541e24b6e240f187ac920cab60f9da&dir=&wwwpkgdb=fc2733d6468c073f1dd738aa598ede55) | _ | glutamate synthase domain protein |
| [OCAR_4052](https://www.genoscope.cns.fr/agc/mage/wwwpkgdb/Info/getInfoLabel.php?id=3484085&wwwpkgdb=fc2733d6468c073f1dd738aa598ede55&nocache=62541e24b6e240f187ac920cab60f9da&dir=&wwwpkgdb=fc2733d6468c073f1dd738aa598ede55) | _ | RND superfamily transporter |
| [OCAR_4055](https://www.genoscope.cns.fr/agc/mage/wwwpkgdb/Info/getInfoLabel.php?id=3482151&wwwpkgdb=fc2733d6468c073f1dd738aa598ede55&nocache=62541e24b6e240f187ac920cab60f9da&dir=&wwwpkgdb=fc2733d6468c073f1dd738aa598ede55) | _ | transglycosylase-associated protein |
| [OCAR_4059](https://www.genoscope.cns.fr/agc/mage/wwwpkgdb/Info/getInfoLabel.php?id=3484087&wwwpkgdb=fc2733d6468c073f1dd738aa598ede55&nocache=62541e24b6e240f187ac920cab60f9da&dir=&wwwpkgdb=fc2733d6468c073f1dd738aa598ede55) | _ | ferredoxin-1 |
| [OCAR_4061](https://www.genoscope.cns.fr/agc/mage/wwwpkgdb/Info/getInfoLabel.php?id=3484089&wwwpkgdb=fc2733d6468c073f1dd738aa598ede55&nocache=62541e24b6e240f187ac920cab60f9da&dir=&wwwpkgdb=fc2733d6468c073f1dd738aa598ede55) | _ | helicase |
| [OCAR_4063](https://www.genoscope.cns.fr/agc/mage/wwwpkgdb/Info/getInfoLabel.php?id=3482154&wwwpkgdb=fc2733d6468c073f1dd738aa598ede55&nocache=62541e24b6e240f187ac920cab60f9da&dir=&wwwpkgdb=fc2733d6468c073f1dd738aa598ede55) | rpmB | 50S ribosomal protein L28 |
| [OCAR_4069](https://www.genoscope.cns.fr/agc/mage/wwwpkgdb/Info/getInfoLabel.php?id=3484096&wwwpkgdb=fc2733d6468c073f1dd738aa598ede55&nocache=62541e24b6e240f187ac920cab60f9da&dir=&wwwpkgdb=fc2733d6468c073f1dd738aa598ede55) | cobT | cobaltochelatase, CobT subunit |
| [OCAR_4070](https://www.genoscope.cns.fr/agc/mage/wwwpkgdb/Info/getInfoLabel.php?id=3484097&wwwpkgdb=fc2733d6468c073f1dd738aa598ede55&nocache=62541e24b6e240f187ac920cab60f9da&dir=&wwwpkgdb=fc2733d6468c073f1dd738aa598ede55) | cobS | cobaltochelatase, CobS subunit |
| [OCAR_4073](https://www.genoscope.cns.fr/agc/mage/wwwpkgdb/Info/getInfoLabel.php?id=3484099&wwwpkgdb=fc2733d6468c073f1dd738aa598ede55&nocache=62541e24b6e240f187ac920cab60f9da&dir=&wwwpkgdb=fc2733d6468c073f1dd738aa598ede55) | _ | Mg2+ and Co2+ transporter CorB family |
| [OCAR_4075](https://www.genoscope.cns.fr/agc/mage/wwwpkgdb/Info/getInfoLabel.php?id=3484101&wwwpkgdb=fc2733d6468c073f1dd738aa598ede55&nocache=62541e24b6e240f187ac920cab60f9da&dir=&wwwpkgdb=fc2733d6468c073f1dd738aa598ede55) | aroB | 3-dehydroquinate synthase |
| [OCAR_4078](https://www.genoscope.cns.fr/agc/mage/wwwpkgdb/Info/getInfoLabel.php?id=3482157&wwwpkgdb=fc2733d6468c073f1dd738aa598ede55&nocache=62541e24b6e240f187ac920cab60f9da&dir=&wwwpkgdb=fc2733d6468c073f1dd738aa598ede55) | xerD | tyrosine recombinase XerD |
| [OCAR_4079](https://www.genoscope.cns.fr/agc/mage/wwwpkgdb/Info/getInfoLabel.php?id=3482158&wwwpkgdb=fc2733d6468c073f1dd738aa598ede55&nocache=62541e24b6e240f187ac920cab60f9da&dir=&wwwpkgdb=fc2733d6468c073f1dd738aa598ede55) | accA | Acetyl-coenzyme A carboxylase carboxyl transferase subunit alpha |
| [OCAR_4082](https://www.genoscope.cns.fr/agc/mage/wwwpkgdb/Info/getInfoLabel.php?id=3484103&wwwpkgdb=fc2733d6468c073f1dd738aa598ede55&nocache=62541e24b6e240f187ac920cab60f9da&dir=&wwwpkgdb=fc2733d6468c073f1dd738aa598ede55) | secA | Protein translocase subunit secA |
| [OCAR_4085](https://www.genoscope.cns.fr/agc/mage/wwwpkgdb/Info/getInfoLabel.php?id=3482162&wwwpkgdb=fc2733d6468c073f1dd738aa598ede55&nocache=62541e24b6e240f187ac920cab60f9da&dir=&wwwpkgdb=fc2733d6468c073f1dd738aa598ede55) | argJ | arginine biosynthesis bifunctional protein ArgJ |
| [OCAR_4086](https://www.genoscope.cns.fr/agc/mage/wwwpkgdb/Info/getInfoLabel.php?id=3482163&wwwpkgdb=fc2733d6468c073f1dd738aa598ede55&nocache=62541e24b6e240f187ac920cab60f9da&dir=&wwwpkgdb=fc2733d6468c073f1dd738aa598ede55) | _ | NTP pyrophosphohydrolase |
| [OCAR_4090](https://www.genoscope.cns.fr/agc/mage/wwwpkgdb/Info/getInfoLabel.php?id=3482166&wwwpkgdb=fc2733d6468c073f1dd738aa598ede55&nocache=62541e24b6e240f187ac920cab60f9da&dir=&wwwpkgdb=fc2733d6468c073f1dd738aa598ede55) | _ | hydrolase Sll0601 |
| [OCAR_4093](https://www.genoscope.cns.fr/agc/mage/wwwpkgdb/Info/getInfoLabel.php?id=3484108&wwwpkgdb=fc2733d6468c073f1dd738aa598ede55&nocache=62541e24b6e240f187ac920cab60f9da&dir=&wwwpkgdb=fc2733d6468c073f1dd738aa598ede55) | ubiG | 3-demethylubiquinone-9 3-O-methyltransferase |
| [OCAR_4095](https://www.genoscope.cns.fr/agc/mage/wwwpkgdb/Info/getInfoLabel.php?id=3482168&wwwpkgdb=fc2733d6468c073f1dd738aa598ede55&nocache=62541e24b6e240f187ac920cab60f9da&dir=&wwwpkgdb=fc2733d6468c073f1dd738aa598ede55) | _ | asparate kinase, monofunctional class |
| [OCAR_4097](https://www.genoscope.cns.fr/agc/mage/wwwpkgdb/Info/getInfoLabel.php?id=3482170&wwwpkgdb=fc2733d6468c073f1dd738aa598ede55&nocache=62541e24b6e240f187ac920cab60f9da&dir=&wwwpkgdb=fc2733d6468c073f1dd738aa598ede55) | ptsP | phosphoenolpyruvate-protein phosphotransferase |
| [OCAR_4098](https://www.genoscope.cns.fr/agc/mage/wwwpkgdb/Info/getInfoLabel.php?id=3482171&wwwpkgdb=fc2733d6468c073f1dd738aa598ede55&nocache=62541e24b6e240f187ac920cab60f9da&dir=&wwwpkgdb=fc2733d6468c073f1dd738aa598ede55) | prfA | Peptide chain release factor 1 |
| [OCAR_4101](https://www.genoscope.cns.fr/agc/mage/wwwpkgdb/Info/getInfoLabel.php?id=3484110&wwwpkgdb=fc2733d6468c073f1dd738aa598ede55&nocache=62541e24b6e240f187ac920cab60f9da&dir=&wwwpkgdb=fc2733d6468c073f1dd738aa598ede55) | _ | methyltransferase type 11 |
| [OCAR_4106](https://www.genoscope.cns.fr/agc/mage/wwwpkgdb/Info/getInfoLabel.php?id=3484112&wwwpkgdb=fc2733d6468c073f1dd738aa598ede55&nocache=62541e24b6e240f187ac920cab60f9da&dir=&wwwpkgdb=fc2733d6468c073f1dd738aa598ede55) | _ | acetoacetyl-CoA reductase |
| [OCAR_4107](https://www.genoscope.cns.fr/agc/mage/wwwpkgdb/Info/getInfoLabel.php?id=3484113&wwwpkgdb=fc2733d6468c073f1dd738aa598ede55&nocache=62541e24b6e240f187ac920cab60f9da&dir=&wwwpkgdb=fc2733d6468c073f1dd738aa598ede55) | _ | acetyl-CoA acetyltransferase |
| [OCAR_4108](https://www.genoscope.cns.fr/agc/mage/wwwpkgdb/Info/getInfoLabel.php?id=3482177&wwwpkgdb=fc2733d6468c073f1dd738aa598ede55&nocache=62541e24b6e240f187ac920cab60f9da&dir=&wwwpkgdb=fc2733d6468c073f1dd738aa598ede55) | _ | polyhydroxyalkanoate synthesis repressor PhaR |
| [OCAR_4114](https://www.genoscope.cns.fr/agc/mage/wwwpkgdb/Info/getInfoLabel.php?id=3484117&wwwpkgdb=fc2733d6468c073f1dd738aa598ede55&nocache=62541e24b6e240f187ac920cab60f9da&dir=&wwwpkgdb=fc2733d6468c073f1dd738aa598ede55) | rpmF | 50S ribosomal protein L32 |
| [OCAR_4116](https://www.genoscope.cns.fr/agc/mage/wwwpkgdb/Info/getInfoLabel.php?id=3482180&wwwpkgdb=fc2733d6468c073f1dd738aa598ede55&nocache=62541e24b6e240f187ac920cab60f9da&dir=&wwwpkgdb=fc2733d6468c073f1dd738aa598ede55) | _ | geranyltranstransferase |
| [OCAR_4119](https://www.genoscope.cns.fr/agc/mage/wwwpkgdb/Info/getInfoLabel.php?id=3484119&wwwpkgdb=fc2733d6468c073f1dd738aa598ede55&nocache=62541e24b6e240f187ac920cab60f9da&dir=&wwwpkgdb=fc2733d6468c073f1dd738aa598ede55) | ispG | 4-hydroxy-3-methylbut-2-en-1-yl diphosphate synthase |
| [OCAR_4120](https://www.genoscope.cns.fr/agc/mage/wwwpkgdb/Info/getInfoLabel.php?id=3484120&wwwpkgdb=fc2733d6468c073f1dd738aa598ede55&nocache=62541e24b6e240f187ac920cab60f9da&dir=&wwwpkgdb=fc2733d6468c073f1dd738aa598ede55) | _ | transcriptional regulator, Fur family |
| [OCAR_4122](https://www.genoscope.cns.fr/agc/mage/wwwpkgdb/Info/getInfoLabel.php?id=3482184&wwwpkgdb=fc2733d6468c073f1dd738aa598ede55&nocache=62541e24b6e240f187ac920cab60f9da&dir=&wwwpkgdb=fc2733d6468c073f1dd738aa598ede55) | mutS | DNA mismatch repair protein MutS |
| [OCAR_4124](https://www.genoscope.cns.fr/agc/mage/wwwpkgdb/Info/getInfoLabel.php?id=3484122&wwwpkgdb=fc2733d6468c073f1dd738aa598ede55&nocache=62541e24b6e240f187ac920cab60f9da&dir=&wwwpkgdb=fc2733d6468c073f1dd738aa598ede55) | _ | OsmC family protein |
| [OCAR_4129](https://www.genoscope.cns.fr/agc/mage/wwwpkgdb/Info/getInfoLabel.php?id=3482185&wwwpkgdb=fc2733d6468c073f1dd738aa598ede55&nocache=62541e24b6e240f187ac920cab60f9da&dir=&wwwpkgdb=fc2733d6468c073f1dd738aa598ede55) | glnD | protein-P-II uridylyltransferase |
| [OCAR_4130](https://www.genoscope.cns.fr/agc/mage/wwwpkgdb/Info/getInfoLabel.php?id=3482186&wwwpkgdb=fc2733d6468c073f1dd738aa598ede55&nocache=62541e24b6e240f187ac920cab60f9da&dir=&wwwpkgdb=fc2733d6468c073f1dd738aa598ede55) | _ | penicillin-binding protein 1A |
| [OCAR_4131](https://www.genoscope.cns.fr/agc/mage/wwwpkgdb/Info/getInfoLabel.php?id=3484127&wwwpkgdb=fc2733d6468c073f1dd738aa598ede55&nocache=62541e24b6e240f187ac920cab60f9da&dir=&wwwpkgdb=fc2733d6468c073f1dd738aa598ede55) | _ | zinc metallopeptidase |
| [OCAR_4132](https://www.genoscope.cns.fr/agc/mage/wwwpkgdb/Info/getInfoLabel.php?id=3484128&wwwpkgdb=fc2733d6468c073f1dd738aa598ede55&nocache=62541e24b6e240f187ac920cab60f9da&dir=&wwwpkgdb=fc2733d6468c073f1dd738aa598ede55) | _ | polyhydroxyalkanoate depolymerase, intracellular |
| [OCAR_4134](https://www.genoscope.cns.fr/agc/mage/wwwpkgdb/Info/getInfoLabel.php?id=3482188&wwwpkgdb=fc2733d6468c073f1dd738aa598ede55&nocache=62541e24b6e240f187ac920cab60f9da&dir=&wwwpkgdb=fc2733d6468c073f1dd738aa598ede55) | _ | photosynthetic apparatus regulatory protein RegA |
| [OCAR_4137](https://www.genoscope.cns.fr/agc/mage/wwwpkgdb/Info/getInfoLabel.php?id=3484129&wwwpkgdb=fc2733d6468c073f1dd738aa598ede55&nocache=62541e24b6e240f187ac920cab60f9da&dir=&wwwpkgdb=fc2733d6468c073f1dd738aa598ede55) | _ | hypothetical protein |
| [OCAR_4138](https://www.genoscope.cns.fr/agc/mage/wwwpkgdb/Info/getInfoLabel.php?id=3482191&wwwpkgdb=fc2733d6468c073f1dd738aa598ede55&nocache=62541e24b6e240f187ac920cab60f9da&dir=&wwwpkgdb=fc2733d6468c073f1dd738aa598ede55) | _ | beta-lactamase domain protein |
| [OCAR_4252](https://www.genoscope.cns.fr/agc/mage/wwwpkgdb/Info/getInfoLabel.php?id=3484202&wwwpkgdb=fc2733d6468c073f1dd738aa598ede55&nocache=62541e24b6e240f187ac920cab60f9da&dir=&wwwpkgdb=fc2733d6468c073f1dd738aa598ede55) | _ | hypothetical protein |
| [OCAR_4259](https://www.genoscope.cns.fr/agc/mage/wwwpkgdb/Info/getInfoLabel.php?id=3484207&wwwpkgdb=fc2733d6468c073f1dd738aa598ede55&nocache=62541e24b6e240f187ac920cab60f9da&dir=&wwwpkgdb=fc2733d6468c073f1dd738aa598ede55) | ispZ | Probable intracellular septation protein |
| [OCAR_4260](https://www.genoscope.cns.fr/agc/mage/wwwpkgdb/Info/getInfoLabel.php?id=3484208&wwwpkgdb=fc2733d6468c073f1dd738aa598ede55&nocache=62541e24b6e240f187ac920cab60f9da&dir=&wwwpkgdb=fc2733d6468c073f1dd738aa598ede55) | ftsY | signal recognition particle-docking protein FtsY |
| [OCAR_4262](https://www.genoscope.cns.fr/agc/mage/wwwpkgdb/Info/getInfoLabel.php?id=3484209&wwwpkgdb=fc2733d6468c073f1dd738aa598ede55&nocache=62541e24b6e240f187ac920cab60f9da&dir=&wwwpkgdb=fc2733d6468c073f1dd738aa598ede55) | _ | hypothetical protein |
| [OCAR_4263](https://www.genoscope.cns.fr/agc/mage/wwwpkgdb/Info/getInfoLabel.php?id=3484210&wwwpkgdb=fc2733d6468c073f1dd738aa598ede55&nocache=62541e24b6e240f187ac920cab60f9da&dir=&wwwpkgdb=fc2733d6468c073f1dd738aa598ede55) | dapF | Diaminopimelate epimerase |
| [OCAR_4267](https://www.genoscope.cns.fr/agc/mage/wwwpkgdb/Info/getInfoLabel.php?id=3482238&wwwpkgdb=fc2733d6468c073f1dd738aa598ede55&nocache=62541e24b6e240f187ac920cab60f9da&dir=&wwwpkgdb=fc2733d6468c073f1dd738aa598ede55) | ffh | signal recognition particle protein |
| [OCAR_4268](https://www.genoscope.cns.fr/agc/mage/wwwpkgdb/Info/getInfoLabel.php?id=3482239&wwwpkgdb=fc2733d6468c073f1dd738aa598ede55&nocache=62541e24b6e240f187ac920cab60f9da&dir=&wwwpkgdb=fc2733d6468c073f1dd738aa598ede55) | rpsP | 30S ribosomal protein S16 |
| [OCAR_4270](https://www.genoscope.cns.fr/agc/mage/wwwpkgdb/Info/getInfoLabel.php?id=3482241&wwwpkgdb=fc2733d6468c073f1dd738aa598ede55&nocache=62541e24b6e240f187ac920cab60f9da&dir=&wwwpkgdb=fc2733d6468c073f1dd738aa598ede55) | trmD | tRNA (guanine-N1)-methyltransferase |
| [OCAR_4271](https://www.genoscope.cns.fr/agc/mage/wwwpkgdb/Info/getInfoLabel.php?id=3482242&wwwpkgdb=fc2733d6468c073f1dd738aa598ede55&nocache=62541e24b6e240f187ac920cab60f9da&dir=&wwwpkgdb=fc2733d6468c073f1dd738aa598ede55) | rplS | 50S ribosomal protein L19 |
| [OCAR_4273](https://www.genoscope.cns.fr/agc/mage/wwwpkgdb/Info/getInfoLabel.php?id=3482243&wwwpkgdb=fc2733d6468c073f1dd738aa598ede55&nocache=62541e24b6e240f187ac920cab60f9da&dir=&wwwpkgdb=fc2733d6468c073f1dd738aa598ede55) | leuC | 3-isopropylmalate dehydratase large subunit |
| [OCAR_4276](https://www.genoscope.cns.fr/agc/mage/wwwpkgdb/Info/getInfoLabel.php?id=3482244&wwwpkgdb=fc2733d6468c073f1dd738aa598ede55&nocache=62541e24b6e240f187ac920cab60f9da&dir=&wwwpkgdb=fc2733d6468c073f1dd738aa598ede55) | leuD | 3-isopropylmalate dehydratase small subunit |
| [OCAR_4279](https://www.genoscope.cns.fr/agc/mage/wwwpkgdb/Info/getInfoLabel.php?id=3484216&wwwpkgdb=fc2733d6468c073f1dd738aa598ede55&nocache=62541e24b6e240f187ac920cab60f9da&dir=&wwwpkgdb=fc2733d6468c073f1dd738aa598ede55) | asd | aspartate-semialdehyde dehydrogenase |
| [OCAR_4283](https://www.genoscope.cns.fr/agc/mage/wwwpkgdb/Info/getInfoLabel.php?id=3484219&wwwpkgdb=fc2733d6468c073f1dd738aa598ede55&nocache=62541e24b6e240f187ac920cab60f9da&dir=&wwwpkgdb=fc2733d6468c073f1dd738aa598ede55) | leuB | 3-isopropylmalate dehydrogenase |
| [OCAR_4285](https://www.genoscope.cns.fr/agc/mage/wwwpkgdb/Info/getInfoLabel.php?id=3482248&wwwpkgdb=fc2733d6468c073f1dd738aa598ede55&nocache=62541e24b6e240f187ac920cab60f9da&dir=&wwwpkgdb=fc2733d6468c073f1dd738aa598ede55) | _ | malonyl-CoA synthase |
| [OCAR_4289](https://www.genoscope.cns.fr/agc/mage/wwwpkgdb/Info/getInfoLabel.php?id=3482251&wwwpkgdb=fc2733d6468c073f1dd738aa598ede55&nocache=62541e24b6e240f187ac920cab60f9da&dir=&wwwpkgdb=fc2733d6468c073f1dd738aa598ede55) | sdhA | succinate dehydrogenase flavoprotein subunit |
| [OCAR_4290](https://www.genoscope.cns.fr/agc/mage/wwwpkgdb/Info/getInfoLabel.php?id=3482252&wwwpkgdb=fc2733d6468c073f1dd738aa598ede55&nocache=62541e24b6e240f187ac920cab60f9da&dir=&wwwpkgdb=fc2733d6468c073f1dd738aa598ede55) | _ | succinate dehydrogenase iron-sulfur subunit |
| [OCAR_4298](https://www.genoscope.cns.fr/agc/mage/wwwpkgdb/Info/getInfoLabel.php?id=3484226&wwwpkgdb=fc2733d6468c073f1dd738aa598ede55&nocache=62541e24b6e240f187ac920cab60f9da&dir=&wwwpkgdb=fc2733d6468c073f1dd738aa598ede55) | _ | radical SAM enzyme, Cfr family |
| [OCAR_4305](https://www.genoscope.cns.fr/agc/mage/wwwpkgdb/Info/getInfoLabel.php?id=3482258&wwwpkgdb=fc2733d6468c073f1dd738aa598ede55&nocache=62541e24b6e240f187ac920cab60f9da&dir=&wwwpkgdb=fc2733d6468c073f1dd738aa598ede55) | typA | GTP-binding protein TypA/BipA |
| [OCAR_4307](https://www.genoscope.cns.fr/agc/mage/wwwpkgdb/Info/getInfoLabel.php?id=3482260&wwwpkgdb=fc2733d6468c073f1dd738aa598ede55&nocache=62541e24b6e240f187ac920cab60f9da&dir=&wwwpkgdb=fc2733d6468c073f1dd738aa598ede55) | _ | acetyltransferase |
| [OCAR_4308](https://www.genoscope.cns.fr/agc/mage/wwwpkgdb/Info/getInfoLabel.php?id=3482261&wwwpkgdb=fc2733d6468c073f1dd738aa598ede55&nocache=62541e24b6e240f187ac920cab60f9da&dir=&wwwpkgdb=fc2733d6468c073f1dd738aa598ede55) | ppa | inorganic diphosphatase |
| [OCAR_4309](https://www.genoscope.cns.fr/agc/mage/wwwpkgdb/Info/getInfoLabel.php?id=3484230&wwwpkgdb=fc2733d6468c073f1dd738aa598ede55&nocache=62541e24b6e240f187ac920cab60f9da&dir=&wwwpkgdb=fc2733d6468c073f1dd738aa598ede55) | folD | Bifunctional protein folD [Includes: Methylenetetrahydrofolate dehydrogenase ; Methenyltetrahydrofolate cyclohydrolase] |
| [OCAR_4311](https://www.genoscope.cns.fr/agc/mage/wwwpkgdb/Info/getInfoLabel.php?id=3484232&wwwpkgdb=fc2733d6468c073f1dd738aa598ede55&nocache=62541e24b6e240f187ac920cab60f9da&dir=&wwwpkgdb=fc2733d6468c073f1dd738aa598ede55) | _ | yggt family protein |
| [OCAR_4327](https://www.genoscope.cns.fr/agc/mage/wwwpkgdb/Info/getInfoLabel.php?id=3482268&wwwpkgdb=fc2733d6468c073f1dd738aa598ede55&nocache=62541e24b6e240f187ac920cab60f9da&dir=&wwwpkgdb=fc2733d6468c073f1dd738aa598ede55) | gcp | Probable O-sialoglycoprotein endopeptidase |
| [OCAR_4329](https://www.genoscope.cns.fr/agc/mage/wwwpkgdb/Info/getInfoLabel.php?id=3482270&wwwpkgdb=fc2733d6468c073f1dd738aa598ede55&nocache=62541e24b6e240f187ac920cab60f9da&dir=&wwwpkgdb=fc2733d6468c073f1dd738aa598ede55) | _ | protein of unknown function DUF55 |
| [OCAR_4330](https://www.genoscope.cns.fr/agc/mage/wwwpkgdb/Info/getInfoLabel.php?id=3482271&wwwpkgdb=fc2733d6468c073f1dd738aa598ede55&nocache=62541e24b6e240f187ac920cab60f9da&dir=&wwwpkgdb=fc2733d6468c073f1dd738aa598ede55) | acsA | acetate--CoA ligase |
| [OCAR_4335](https://www.genoscope.cns.fr/agc/mage/wwwpkgdb/Info/getInfoLabel.php?id=3482273&wwwpkgdb=fc2733d6468c073f1dd738aa598ede55&nocache=62541e24b6e240f187ac920cab60f9da&dir=&wwwpkgdb=fc2733d6468c073f1dd738aa598ede55) | _ | SUN protein |
| [OCAR_4336](https://www.genoscope.cns.fr/agc/mage/wwwpkgdb/Info/getInfoLabel.php?id=3482274&wwwpkgdb=fc2733d6468c073f1dd738aa598ede55&nocache=62541e24b6e240f187ac920cab60f9da&dir=&wwwpkgdb=fc2733d6468c073f1dd738aa598ede55) | _ | heparinase II/III family protein |
| [OCAR_4337](https://www.genoscope.cns.fr/agc/mage/wwwpkgdb/Info/getInfoLabel.php?id=3482275&wwwpkgdb=fc2733d6468c073f1dd738aa598ede55&nocache=62541e24b6e240f187ac920cab60f9da&dir=&wwwpkgdb=fc2733d6468c073f1dd738aa598ede55) | purH | bifunctional purine biosynthesis protein PurH |
| [OCAR_4338](https://www.genoscope.cns.fr/agc/mage/wwwpkgdb/Info/getInfoLabel.php?id=3484245&wwwpkgdb=fc2733d6468c073f1dd738aa598ede55&nocache=62541e24b6e240f187ac920cab60f9da&dir=&wwwpkgdb=fc2733d6468c073f1dd738aa598ede55) | _ | major facilitator superfamily MFS_1 |
| [OCAR_4339](https://www.genoscope.cns.fr/agc/mage/wwwpkgdb/Info/getInfoLabel.php?id=3482276&wwwpkgdb=fc2733d6468c073f1dd738aa598ede55&nocache=62541e24b6e240f187ac920cab60f9da&dir=&wwwpkgdb=fc2733d6468c073f1dd738aa598ede55) | ggt1 | gamma-glutamyltransferase |
| [OCAR_4347](https://www.genoscope.cns.fr/agc/mage/wwwpkgdb/Info/getInfoLabel.php?id=3482280&wwwpkgdb=fc2733d6468c073f1dd738aa598ede55&nocache=62541e24b6e240f187ac920cab60f9da&dir=&wwwpkgdb=fc2733d6468c073f1dd738aa598ede55) | _ | peptidase S16, lon domain protein |
| [OCAR_4348](https://www.genoscope.cns.fr/agc/mage/wwwpkgdb/Info/getInfoLabel.php?id=3482281&wwwpkgdb=fc2733d6468c073f1dd738aa598ede55&nocache=62541e24b6e240f187ac920cab60f9da&dir=&wwwpkgdb=fc2733d6468c073f1dd738aa598ede55) | _ | tetraacyldisaccharide 4'-kinase |
| [OCAR_4349](https://www.genoscope.cns.fr/agc/mage/wwwpkgdb/Info/getInfoLabel.php?id=3484250&wwwpkgdb=fc2733d6468c073f1dd738aa598ede55&nocache=62541e24b6e240f187ac920cab60f9da&dir=&wwwpkgdb=fc2733d6468c073f1dd738aa598ede55) | _ | 2-octaprenyl-6-methoxyphenyl hydroxylase |
| [OCAR_4351](https://www.genoscope.cns.fr/agc/mage/wwwpkgdb/Info/getInfoLabel.php?id=3482283&wwwpkgdb=fc2733d6468c073f1dd738aa598ede55&nocache=62541e24b6e240f187ac920cab60f9da&dir=&wwwpkgdb=fc2733d6468c073f1dd738aa598ede55) | _ | nitrogen regulatory protein P-II |
| [OCAR_4355](https://www.genoscope.cns.fr/agc/mage/wwwpkgdb/Info/getInfoLabel.php?id=3482287&wwwpkgdb=fc2733d6468c073f1dd738aa598ede55&nocache=62541e24b6e240f187ac920cab60f9da&dir=&wwwpkgdb=fc2733d6468c073f1dd738aa598ede55) | _ | DNA translocase FtsK |
| [OCAR_4357](https://www.genoscope.cns.fr/agc/mage/wwwpkgdb/Info/getInfoLabel.php?id=3482289&wwwpkgdb=fc2733d6468c073f1dd738aa598ede55&nocache=62541e24b6e240f187ac920cab60f9da&dir=&wwwpkgdb=fc2733d6468c073f1dd738aa598ede55) | _ | exodeoxyribonuclease III xth |
| [OCAR_4359](https://www.genoscope.cns.fr/agc/mage/wwwpkgdb/Info/getInfoLabel.php?id=3484252&wwwpkgdb=fc2733d6468c073f1dd738aa598ede55&nocache=62541e24b6e240f187ac920cab60f9da&dir=&wwwpkgdb=fc2733d6468c073f1dd738aa598ede55) | _ | two component transcriptional regulator, winged helix family |
| [OCAR_4360](https://www.genoscope.cns.fr/agc/mage/wwwpkgdb/Info/getInfoLabel.php?id=3482290&wwwpkgdb=fc2733d6468c073f1dd738aa598ede55&nocache=62541e24b6e240f187ac920cab60f9da&dir=&wwwpkgdb=fc2733d6468c073f1dd738aa598ede55) | _ | hypothetical protein |
| [OCAR_4361](https://www.genoscope.cns.fr/agc/mage/wwwpkgdb/Info/getInfoLabel.php?id=3484253&wwwpkgdb=fc2733d6468c073f1dd738aa598ede55&nocache=62541e24b6e240f187ac920cab60f9da&dir=&wwwpkgdb=fc2733d6468c073f1dd738aa598ede55) | _ | pyridoxal phosphate enzyme, YggS family |
| [OCAR_4363](https://www.genoscope.cns.fr/agc/mage/wwwpkgdb/Info/getInfoLabel.php?id=3482292&wwwpkgdb=fc2733d6468c073f1dd738aa598ede55&nocache=62541e24b6e240f187ac920cab60f9da&dir=&wwwpkgdb=fc2733d6468c073f1dd738aa598ede55) | leuS | Leucyl-tRNA synthetase |
| [OCAR_4367](https://www.genoscope.cns.fr/agc/mage/wwwpkgdb/Info/getInfoLabel.php?id=3484254&wwwpkgdb=fc2733d6468c073f1dd738aa598ede55&nocache=62541e24b6e240f187ac920cab60f9da&dir=&wwwpkgdb=fc2733d6468c073f1dd738aa598ede55) | _ | ParB family protein |
| [OCAR_4368](https://www.genoscope.cns.fr/agc/mage/wwwpkgdb/Info/getInfoLabel.php?id=3484255&wwwpkgdb=fc2733d6468c073f1dd738aa598ede55&nocache=62541e24b6e240f187ac920cab60f9da&dir=&wwwpkgdb=fc2733d6468c073f1dd738aa598ede55) | _ | chromosome partitioning protein ParA |
| [OCAR_4369](https://www.genoscope.cns.fr/agc/mage/wwwpkgdb/Info/getInfoLabel.php?id=3484256&wwwpkgdb=fc2733d6468c073f1dd738aa598ede55&nocache=62541e24b6e240f187ac920cab60f9da&dir=&wwwpkgdb=fc2733d6468c073f1dd738aa598ede55) | gidB | methyltransferase GidB |
| [OCAR_4370](https://www.genoscope.cns.fr/agc/mage/wwwpkgdb/Info/getInfoLabel.php?id=3484257&wwwpkgdb=fc2733d6468c073f1dd738aa598ede55&nocache=62541e24b6e240f187ac920cab60f9da&dir=&wwwpkgdb=fc2733d6468c073f1dd738aa598ede55) | mnmG | tRNA uridine 5-carboxymethylaminomethyl modification enzyme mnmG |
| [OCAR_4382](https://www.genoscope.cns.fr/agc/mage/wwwpkgdb/Info/getInfoLabel.php?id=3484263&wwwpkgdb=fc2733d6468c073f1dd738aa598ede55&nocache=62541e24b6e240f187ac920cab60f9da&dir=&wwwpkgdb=fc2733d6468c073f1dd738aa598ede55) | rho | transcription termination factor Rho |
| [OCAR_4385](https://www.genoscope.cns.fr/agc/mage/wwwpkgdb/Info/getInfoLabel.php?id=3482303&wwwpkgdb=fc2733d6468c073f1dd738aa598ede55&nocache=62541e24b6e240f187ac920cab60f9da&dir=&wwwpkgdb=fc2733d6468c073f1dd738aa598ede55) | _ | Putative phosphotransferase OCAR_4385 |
| [OCAR_4388](https://www.genoscope.cns.fr/agc/mage/wwwpkgdb/Info/getInfoLabel.php?id=3482306&wwwpkgdb=fc2733d6468c073f1dd738aa598ede55&nocache=62541e24b6e240f187ac920cab60f9da&dir=&wwwpkgdb=fc2733d6468c073f1dd738aa598ede55) | dnaQ | DNA polymerase III, epsilon subunit |
| [OCAR_4393](https://www.genoscope.cns.fr/agc/mage/wwwpkgdb/Info/getInfoLabel.php?id=3484266&wwwpkgdb=fc2733d6468c073f1dd738aa598ede55&nocache=62541e24b6e240f187ac920cab60f9da&dir=&wwwpkgdb=fc2733d6468c073f1dd738aa598ede55) | hslU | heat shock protein HslVU, ATPase subunit HslU |
| [OCAR_4394](https://www.genoscope.cns.fr/agc/mage/wwwpkgdb/Info/getInfoLabel.php?id=3484267&wwwpkgdb=fc2733d6468c073f1dd738aa598ede55&nocache=62541e24b6e240f187ac920cab60f9da&dir=&wwwpkgdb=fc2733d6468c073f1dd738aa598ede55) | hslV | ATP-dependent protease hslV |
| [OCAR_4395](https://www.genoscope.cns.fr/agc/mage/wwwpkgdb/Info/getInfoLabel.php?id=3482310&wwwpkgdb=fc2733d6468c073f1dd738aa598ede55&nocache=62541e24b6e240f187ac920cab60f9da&dir=&wwwpkgdb=fc2733d6468c073f1dd738aa598ede55) | hisB | Imidazoleglycerol-phosphate dehydratase |
| [OCAR_4397](https://www.genoscope.cns.fr/agc/mage/wwwpkgdb/Info/getInfoLabel.php?id=3482312&wwwpkgdb=fc2733d6468c073f1dd738aa598ede55&nocache=62541e24b6e240f187ac920cab60f9da&dir=&wwwpkgdb=fc2733d6468c073f1dd738aa598ede55) | hisH | imidazole glycerol phosphate synthase, glutamine amidotransferase subunit |
| [OCAR_4398](https://www.genoscope.cns.fr/agc/mage/wwwpkgdb/Info/getInfoLabel.php?id=3482313&wwwpkgdb=fc2733d6468c073f1dd738aa598ede55&nocache=62541e24b6e240f187ac920cab60f9da&dir=&wwwpkgdb=fc2733d6468c073f1dd738aa598ede55) | hisA | 1-(5-phosphoribosyl)-5-[(5-phosphoribosylamino)methylideneamino] imidazole-4-carboxamide isomerase |
| [OCAR_4399](https://www.genoscope.cns.fr/agc/mage/wwwpkgdb/Info/getInfoLabel.php?id=3482314&wwwpkgdb=fc2733d6468c073f1dd738aa598ede55&nocache=62541e24b6e240f187ac920cab60f9da&dir=&wwwpkgdb=fc2733d6468c073f1dd738aa598ede55) | hisF | Imidazole glycerol phosphate synthase subunit hisF |
| [OCAR_4401](https://www.genoscope.cns.fr/agc/mage/wwwpkgdb/Info/getInfoLabel.php?id=3482316&wwwpkgdb=fc2733d6468c073f1dd738aa598ede55&nocache=62541e24b6e240f187ac920cab60f9da&dir=&wwwpkgdb=fc2733d6468c073f1dd738aa598ede55) | coaA | Pantothenate kinase |
| [OCAR_4405](https://www.genoscope.cns.fr/agc/mage/wwwpkgdb/Info/getInfoLabel.php?id=3484271&wwwpkgdb=fc2733d6468c073f1dd738aa598ede55&nocache=62541e24b6e240f187ac920cab60f9da&dir=&wwwpkgdb=fc2733d6468c073f1dd738aa598ede55) | _ | Mg chelatase-like protein |
| [OCAR_4407](https://www.genoscope.cns.fr/agc/mage/wwwpkgdb/Info/getInfoLabel.php?id=3484273&wwwpkgdb=fc2733d6468c073f1dd738aa598ede55&nocache=62541e24b6e240f187ac920cab60f9da&dir=&wwwpkgdb=fc2733d6468c073f1dd738aa598ede55) | gshB | glutathione synthase |
| [OCAR_4409](https://www.genoscope.cns.fr/agc/mage/wwwpkgdb/Info/getInfoLabel.php?id=3484275&wwwpkgdb=fc2733d6468c073f1dd738aa598ede55&nocache=62541e24b6e240f187ac920cab60f9da&dir=&wwwpkgdb=fc2733d6468c073f1dd738aa598ede55) | _ | hypothetical protein |
| [OCAR_4411](https://www.genoscope.cns.fr/agc/mage/wwwpkgdb/Info/getInfoLabel.php?id=3484276&wwwpkgdb=fc2733d6468c073f1dd738aa598ede55&nocache=62541e24b6e240f187ac920cab60f9da&dir=&wwwpkgdb=fc2733d6468c073f1dd738aa598ede55) | _ | putative oxygen-independent coproporphyrinogen III oxidase |
| [OCAR_4412](https://www.genoscope.cns.fr/agc/mage/wwwpkgdb/Info/getInfoLabel.php?id=3484277&wwwpkgdb=fc2733d6468c073f1dd738aa598ede55&nocache=62541e24b6e240f187ac920cab60f9da&dir=&wwwpkgdb=fc2733d6468c073f1dd738aa598ede55) | rdgB | non-canonical purine NTP pyrophosphatase |
| [OCAR_4413](https://www.genoscope.cns.fr/agc/mage/wwwpkgdb/Info/getInfoLabel.php?id=3484278&wwwpkgdb=fc2733d6468c073f1dd738aa598ede55&nocache=62541e24b6e240f187ac920cab60f9da&dir=&wwwpkgdb=fc2733d6468c073f1dd738aa598ede55) | rph | Ribonuclease PH |
| [OCAR_4414](https://www.genoscope.cns.fr/agc/mage/wwwpkgdb/Info/getInfoLabel.php?id=3482318&wwwpkgdb=fc2733d6468c073f1dd738aa598ede55&nocache=62541e24b6e240f187ac920cab60f9da&dir=&wwwpkgdb=fc2733d6468c073f1dd738aa598ede55) | hrcA | Heat-inducible transcription repressor hrcA |
| [OCAR_4415](https://www.genoscope.cns.fr/agc/mage/wwwpkgdb/Info/getInfoLabel.php?id=3482319&wwwpkgdb=fc2733d6468c073f1dd738aa598ede55&nocache=62541e24b6e240f187ac920cab60f9da&dir=&wwwpkgdb=fc2733d6468c073f1dd738aa598ede55) | grpE | Protein grpE |
| [OCAR_4417](https://www.genoscope.cns.fr/agc/mage/wwwpkgdb/Info/getInfoLabel.php?id=3482320&wwwpkgdb=fc2733d6468c073f1dd738aa598ede55&nocache=62541e24b6e240f187ac920cab60f9da&dir=&wwwpkgdb=fc2733d6468c073f1dd738aa598ede55) | dnaK | Chaperone protein dnaK |
| [OCAR_4418](https://www.genoscope.cns.fr/agc/mage/wwwpkgdb/Info/getInfoLabel.php?id=3482321&wwwpkgdb=fc2733d6468c073f1dd738aa598ede55&nocache=62541e24b6e240f187ac920cab60f9da&dir=&wwwpkgdb=fc2733d6468c073f1dd738aa598ede55) | dnaJ | chaperone protein DnaJ |
| [OCAR_4419](https://www.genoscope.cns.fr/agc/mage/wwwpkgdb/Info/getInfoLabel.php?id=3482322&wwwpkgdb=fc2733d6468c073f1dd738aa598ede55&nocache=62541e24b6e240f187ac920cab60f9da&dir=&wwwpkgdb=fc2733d6468c073f1dd738aa598ede55) | _ | ribosomal RNA adenine methylase transferase |
| [OCAR_4422](https://www.genoscope.cns.fr/agc/mage/wwwpkgdb/Info/getInfoLabel.php?id=3482325&wwwpkgdb=fc2733d6468c073f1dd738aa598ede55&nocache=62541e24b6e240f187ac920cab60f9da&dir=&wwwpkgdb=fc2733d6468c073f1dd738aa598ede55) | dapB | Dihydrodipicolinate reductase |
| [OCAR_4423](https://www.genoscope.cns.fr/agc/mage/wwwpkgdb/Info/getInfoLabel.php?id=3482326&wwwpkgdb=fc2733d6468c073f1dd738aa598ede55&nocache=62541e24b6e240f187ac920cab60f9da&dir=&wwwpkgdb=fc2733d6468c073f1dd738aa598ede55) | gpmA | 2,3-bisphosphoglycerate-dependent phosphoglycerate mutase |
| [OCAR_4424](https://www.genoscope.cns.fr/agc/mage/wwwpkgdb/Info/getInfoLabel.php?id=3484280&wwwpkgdb=fc2733d6468c073f1dd738aa598ede55&nocache=62541e24b6e240f187ac920cab60f9da&dir=&wwwpkgdb=fc2733d6468c073f1dd738aa598ede55) | _ | transcriptional regulator of AraC family |
| [OCAR_4428](https://www.genoscope.cns.fr/agc/mage/wwwpkgdb/Info/getInfoLabel.php?id=3484283&wwwpkgdb=fc2733d6468c073f1dd738aa598ede55&nocache=62541e24b6e240f187ac920cab60f9da&dir=&wwwpkgdb=fc2733d6468c073f1dd738aa598ede55) | _ | hypothetical protein |
| [OCAR_4438](https://www.genoscope.cns.fr/agc/mage/wwwpkgdb/Info/getInfoLabel.php?id=3484289&wwwpkgdb=fc2733d6468c073f1dd738aa598ede55&nocache=62541e24b6e240f187ac920cab60f9da&dir=&wwwpkgdb=fc2733d6468c073f1dd738aa598ede55) | pheT | phenylalanyl-tRNA synthetase, beta subunit |
| [OCAR_4439](https://www.genoscope.cns.fr/agc/mage/wwwpkgdb/Info/getInfoLabel.php?id=3484290&wwwpkgdb=fc2733d6468c073f1dd738aa598ede55&nocache=62541e24b6e240f187ac920cab60f9da&dir=&wwwpkgdb=fc2733d6468c073f1dd738aa598ede55) | pheS | Phenylalanyl-tRNA synthetase alpha chain |
| [OCAR_4440](https://www.genoscope.cns.fr/agc/mage/wwwpkgdb/Info/getInfoLabel.php?id=3484291&wwwpkgdb=fc2733d6468c073f1dd738aa598ede55&nocache=62541e24b6e240f187ac920cab60f9da&dir=&wwwpkgdb=fc2733d6468c073f1dd738aa598ede55) | rplT | 50S ribosomal protein L20 |
| [OCAR_4441](https://www.genoscope.cns.fr/agc/mage/wwwpkgdb/Info/getInfoLabel.php?id=3484292&wwwpkgdb=fc2733d6468c073f1dd738aa598ede55&nocache=62541e24b6e240f187ac920cab60f9da&dir=&wwwpkgdb=fc2733d6468c073f1dd738aa598ede55) | rpmI | 50S ribosomal protein L35 |
| [OCAR_4442](https://www.genoscope.cns.fr/agc/mage/wwwpkgdb/Info/getInfoLabel.php?id=3484293&wwwpkgdb=fc2733d6468c073f1dd738aa598ede55&nocache=62541e24b6e240f187ac920cab60f9da&dir=&wwwpkgdb=fc2733d6468c073f1dd738aa598ede55) | infC | translation initiation factor IF-3 |
| [OCAR_4446](https://www.genoscope.cns.fr/agc/mage/wwwpkgdb/Info/getInfoLabel.php?id=3484295&wwwpkgdb=fc2733d6468c073f1dd738aa598ede55&nocache=62541e24b6e240f187ac920cab60f9da&dir=&wwwpkgdb=fc2733d6468c073f1dd738aa598ede55) | _ | glutathione S-transferase |
| [OCAR_4447](https://www.genoscope.cns.fr/agc/mage/wwwpkgdb/Info/getInfoLabel.php?id=3482334&wwwpkgdb=fc2733d6468c073f1dd738aa598ede55&nocache=62541e24b6e240f187ac920cab60f9da&dir=&wwwpkgdb=fc2733d6468c073f1dd738aa598ede55) | uppP | Undecaprenyl-diphosphatase |
| [OCAR_4455](https://www.genoscope.cns.fr/agc/mage/wwwpkgdb/Info/getInfoLabel.php?id=3482339&wwwpkgdb=fc2733d6468c073f1dd738aa598ede55&nocache=62541e24b6e240f187ac920cab60f9da&dir=&wwwpkgdb=fc2733d6468c073f1dd738aa598ede55) | _ | 3'-5' exonuclease |
| [OCAR_4459](https://www.genoscope.cns.fr/agc/mage/wwwpkgdb/Info/getInfoLabel.php?id=3482343&wwwpkgdb=fc2733d6468c073f1dd738aa598ede55&nocache=62541e24b6e240f187ac920cab60f9da&dir=&wwwpkgdb=fc2733d6468c073f1dd738aa598ede55) | rpoN | RNA polymerase sigma-54 factor |
| [OCAR_4460](https://www.genoscope.cns.fr/agc/mage/wwwpkgdb/Info/getInfoLabel.php?id=3482344&wwwpkgdb=fc2733d6468c073f1dd738aa598ede55&nocache=62541e24b6e240f187ac920cab60f9da&dir=&wwwpkgdb=fc2733d6468c073f1dd738aa598ede55) | _ | sigma 54 modulation protein/ribosomal protein S30EA |
| [OCAR_4461](https://www.genoscope.cns.fr/agc/mage/wwwpkgdb/Info/getInfoLabel.php?id=3482345&wwwpkgdb=fc2733d6468c073f1dd738aa598ede55&nocache=62541e24b6e240f187ac920cab60f9da&dir=&wwwpkgdb=fc2733d6468c073f1dd738aa598ede55) | _ | hypothetical protein |
| [OCAR_4462](https://www.genoscope.cns.fr/agc/mage/wwwpkgdb/Info/getInfoLabel.php?id=3484299&wwwpkgdb=fc2733d6468c073f1dd738aa598ede55&nocache=62541e24b6e240f187ac920cab60f9da&dir=&wwwpkgdb=fc2733d6468c073f1dd738aa598ede55) | ilvD | dihydroxy-acid dehydratase |
| [OCAR_4463](https://www.genoscope.cns.fr/agc/mage/wwwpkgdb/Info/getInfoLabel.php?id=3484300&wwwpkgdb=fc2733d6468c073f1dd738aa598ede55&nocache=62541e24b6e240f187ac920cab60f9da&dir=&wwwpkgdb=fc2733d6468c073f1dd738aa598ede55) | _ | hypothetical protein |
| [OCAR_4464](https://www.genoscope.cns.fr/agc/mage/wwwpkgdb/Info/getInfoLabel.php?id=3484301&wwwpkgdb=fc2733d6468c073f1dd738aa598ede55&nocache=62541e24b6e240f187ac920cab60f9da&dir=&wwwpkgdb=fc2733d6468c073f1dd738aa598ede55) | _ | heat shock protein Hsp20 |
| [OCAR_4466](https://www.genoscope.cns.fr/agc/mage/wwwpkgdb/Info/getInfoLabel.php?id=3484303&wwwpkgdb=fc2733d6468c073f1dd738aa598ede55&nocache=62541e24b6e240f187ac920cab60f9da&dir=&wwwpkgdb=fc2733d6468c073f1dd738aa598ede55) | _ | hypothetical protein |
| [OCAR_4467](https://www.genoscope.cns.fr/agc/mage/wwwpkgdb/Info/getInfoLabel.php?id=3482346&wwwpkgdb=fc2733d6468c073f1dd738aa598ede55&nocache=62541e24b6e240f187ac920cab60f9da&dir=&wwwpkgdb=fc2733d6468c073f1dd738aa598ede55) | aroA | 3-phosphoshikimate 1-carboxyvinyltransferase |
| [OCAR_4470](https://www.genoscope.cns.fr/agc/mage/wwwpkgdb/Info/getInfoLabel.php?id=3482349&wwwpkgdb=fc2733d6468c073f1dd738aa598ede55&nocache=62541e24b6e240f187ac920cab60f9da&dir=&wwwpkgdb=fc2733d6468c073f1dd738aa598ede55) | cmk | Cytidylate kinase |
| [OCAR_4472](https://www.genoscope.cns.fr/agc/mage/wwwpkgdb/Info/getInfoLabel.php?id=3482350&wwwpkgdb=fc2733d6468c073f1dd738aa598ede55&nocache=62541e24b6e240f187ac920cab60f9da&dir=&wwwpkgdb=fc2733d6468c073f1dd738aa598ede55) | rpsA | ribosomal protein S1 |
| [OCAR_4474](https://www.genoscope.cns.fr/agc/mage/wwwpkgdb/Info/getInfoLabel.php?id=3482352&wwwpkgdb=fc2733d6468c073f1dd738aa598ede55&nocache=62541e24b6e240f187ac920cab60f9da&dir=&wwwpkgdb=fc2733d6468c073f1dd738aa598ede55) | ihfB | Integration host factor subunit beta |
| [OCAR_4477](https://www.genoscope.cns.fr/agc/mage/wwwpkgdb/Info/getInfoLabel.php?id=3482355&wwwpkgdb=fc2733d6468c073f1dd738aa598ede55&nocache=62541e24b6e240f187ac920cab60f9da&dir=&wwwpkgdb=fc2733d6468c073f1dd738aa598ede55) | trpB | Tryptophan synthase beta chain |
| [OCAR_4478](https://www.genoscope.cns.fr/agc/mage/wwwpkgdb/Info/getInfoLabel.php?id=3482356&wwwpkgdb=fc2733d6468c073f1dd738aa598ede55&nocache=62541e24b6e240f187ac920cab60f9da&dir=&wwwpkgdb=fc2733d6468c073f1dd738aa598ede55) | trpA | Tryptophan synthase alpha chain |
| [OCAR_4479](https://www.genoscope.cns.fr/agc/mage/wwwpkgdb/Info/getInfoLabel.php?id=3482357&wwwpkgdb=fc2733d6468c073f1dd738aa598ede55&nocache=62541e24b6e240f187ac920cab60f9da&dir=&wwwpkgdb=fc2733d6468c073f1dd738aa598ede55) | accD | acetyl-CoA carboxylase |
| [OCAR_4481](https://www.genoscope.cns.fr/agc/mage/wwwpkgdb/Info/getInfoLabel.php?id=3484305&wwwpkgdb=fc2733d6468c073f1dd738aa598ede55&nocache=62541e24b6e240f187ac920cab60f9da&dir=&wwwpkgdb=fc2733d6468c073f1dd738aa598ede55) | trx | thioredoxin |
| [OCAR_4485](https://www.genoscope.cns.fr/agc/mage/wwwpkgdb/Info/getInfoLabel.php?id=3484309&wwwpkgdb=fc2733d6468c073f1dd738aa598ede55&nocache=62541e24b6e240f187ac920cab60f9da&dir=&wwwpkgdb=fc2733d6468c073f1dd738aa598ede55) | _ | type IV pilus assembly PilZ |
| [OCAR_4490](https://www.genoscope.cns.fr/agc/mage/wwwpkgdb/Info/getInfoLabel.php?id=3484314&wwwpkgdb=fc2733d6468c073f1dd738aa598ede55&nocache=62541e24b6e240f187ac920cab60f9da&dir=&wwwpkgdb=fc2733d6468c073f1dd738aa598ede55) | ubiB | 2-polyprenylphenol 6-hydroxylase |
| [OCAR_4491](https://www.genoscope.cns.fr/agc/mage/wwwpkgdb/Info/getInfoLabel.php?id=3484315&wwwpkgdb=fc2733d6468c073f1dd738aa598ede55&nocache=62541e24b6e240f187ac920cab60f9da&dir=&wwwpkgdb=fc2733d6468c073f1dd738aa598ede55) | ubiE | ubiquinone/menaquinone biosynthesis methlytransferase UbiE |
| [OCAR_4492](https://www.genoscope.cns.fr/agc/mage/wwwpkgdb/Info/getInfoLabel.php?id=3482359&wwwpkgdb=fc2733d6468c073f1dd738aa598ede55&nocache=62541e24b6e240f187ac920cab60f9da&dir=&wwwpkgdb=fc2733d6468c073f1dd738aa598ede55) | mutM | Formamidopyrimidine-DNA glycosylase |
| [OCAR_4493](https://www.genoscope.cns.fr/agc/mage/wwwpkgdb/Info/getInfoLabel.php?id=3482360&wwwpkgdb=fc2733d6468c073f1dd738aa598ede55&nocache=62541e24b6e240f187ac920cab60f9da&dir=&wwwpkgdb=fc2733d6468c073f1dd738aa598ede55) | _ | uba/thif-type NAD/fad binding fold protein |
| [OCAR_4494](https://www.genoscope.cns.fr/agc/mage/wwwpkgdb/Info/getInfoLabel.php?id=3484316&wwwpkgdb=fc2733d6468c073f1dd738aa598ede55&nocache=62541e24b6e240f187ac920cab60f9da&dir=&wwwpkgdb=fc2733d6468c073f1dd738aa598ede55) | _ | glyoxylate reductase |
| [OCAR_4496](https://www.genoscope.cns.fr/agc/mage/wwwpkgdb/Info/getInfoLabel.php?id=3484317&wwwpkgdb=fc2733d6468c073f1dd738aa598ede55&nocache=62541e24b6e240f187ac920cab60f9da&dir=&wwwpkgdb=fc2733d6468c073f1dd738aa598ede55) | _ | ferric uptake regulator, FUR family |
| [OCAR_4497](https://www.genoscope.cns.fr/agc/mage/wwwpkgdb/Info/getInfoLabel.php?id=3482362&wwwpkgdb=fc2733d6468c073f1dd738aa598ede55&nocache=62541e24b6e240f187ac920cab60f9da&dir=&wwwpkgdb=fc2733d6468c073f1dd738aa598ede55) | fabA | 3-hydroxydecanoyl-[acyl-carrier-protein] dehydratase |
| [OCAR_4498](https://www.genoscope.cns.fr/agc/mage/wwwpkgdb/Info/getInfoLabel.php?id=3482363&wwwpkgdb=fc2733d6468c073f1dd738aa598ede55&nocache=62541e24b6e240f187ac920cab60f9da&dir=&wwwpkgdb=fc2733d6468c073f1dd738aa598ede55) | _ | 3-oxoacyl-[acyl-carrier-protein] synthase 1 |
| [OCAR_4499](https://www.genoscope.cns.fr/agc/mage/wwwpkgdb/Info/getInfoLabel.php?id=3482364&wwwpkgdb=fc2733d6468c073f1dd738aa598ede55&nocache=62541e24b6e240f187ac920cab60f9da&dir=&wwwpkgdb=fc2733d6468c073f1dd738aa598ede55) | _ | enoyl-[acyl-carrier-protein] reductase |
| [OCAR_4503](https://www.genoscope.cns.fr/agc/mage/wwwpkgdb/Info/getInfoLabel.php?id=3484320&wwwpkgdb=fc2733d6468c073f1dd738aa598ede55&nocache=62541e24b6e240f187ac920cab60f9da&dir=&wwwpkgdb=fc2733d6468c073f1dd738aa598ede55) | pnp | Polyribonucleotide nucleotidyltransferase |
| [OCAR_4504](https://www.genoscope.cns.fr/agc/mage/wwwpkgdb/Info/getInfoLabel.php?id=3484321&wwwpkgdb=fc2733d6468c073f1dd738aa598ede55&nocache=62541e24b6e240f187ac920cab60f9da&dir=&wwwpkgdb=fc2733d6468c073f1dd738aa598ede55) | rpsO | 30S ribosomal protein S15 |
| [OCAR_4505](https://www.genoscope.cns.fr/agc/mage/wwwpkgdb/Info/getInfoLabel.php?id=3484322&wwwpkgdb=fc2733d6468c073f1dd738aa598ede55&nocache=62541e24b6e240f187ac920cab60f9da&dir=&wwwpkgdb=fc2733d6468c073f1dd738aa598ede55) | _ | tRNA pseudouridine synthase B |
| [OCAR_4506](https://www.genoscope.cns.fr/agc/mage/wwwpkgdb/Info/getInfoLabel.php?id=3484323&wwwpkgdb=fc2733d6468c073f1dd738aa598ede55&nocache=62541e24b6e240f187ac920cab60f9da&dir=&wwwpkgdb=fc2733d6468c073f1dd738aa598ede55) | rbfA | Ribosome-binding factor A |
| [OCAR_4508](https://www.genoscope.cns.fr/agc/mage/wwwpkgdb/Info/getInfoLabel.php?id=3484325&wwwpkgdb=fc2733d6468c073f1dd738aa598ede55&nocache=62541e24b6e240f187ac920cab60f9da&dir=&wwwpkgdb=fc2733d6468c073f1dd738aa598ede55) | infB | translation initiation factor IF-2 |
| [OCAR_4511](https://www.genoscope.cns.fr/agc/mage/wwwpkgdb/Info/getInfoLabel.php?id=3484327&wwwpkgdb=fc2733d6468c073f1dd738aa598ede55&nocache=62541e24b6e240f187ac920cab60f9da&dir=&wwwpkgdb=fc2733d6468c073f1dd738aa598ede55) | _ | transcription elongation factor NusA |
| [OCAR_4515](https://www.genoscope.cns.fr/agc/mage/wwwpkgdb/Info/getInfoLabel.php?id=3484330&wwwpkgdb=fc2733d6468c073f1dd738aa598ede55&nocache=62541e24b6e240f187ac920cab60f9da&dir=&wwwpkgdb=fc2733d6468c073f1dd738aa598ede55) | _ | transcriptional regulator of XRE family |
| [OCAR_4520](https://www.genoscope.cns.fr/agc/mage/wwwpkgdb/Info/getInfoLabel.php?id=3484334&wwwpkgdb=fc2733d6468c073f1dd738aa598ede55&nocache=62541e24b6e240f187ac920cab60f9da&dir=&wwwpkgdb=fc2733d6468c073f1dd738aa598ede55) | _ | PhoH family protein |
| [OCAR_4521](https://www.genoscope.cns.fr/agc/mage/wwwpkgdb/Info/getInfoLabel.php?id=3484335&wwwpkgdb=fc2733d6468c073f1dd738aa598ede55&nocache=62541e24b6e240f187ac920cab60f9da&dir=&wwwpkgdb=fc2733d6468c073f1dd738aa598ede55) | miaB | (Dimethylallyl)adenosine tRNA methylthiotransferase miaB |
| [OCAR_4522](https://www.genoscope.cns.fr/agc/mage/wwwpkgdb/Info/getInfoLabel.php?id=3484336&wwwpkgdb=fc2733d6468c073f1dd738aa598ede55&nocache=62541e24b6e240f187ac920cab60f9da&dir=&wwwpkgdb=fc2733d6468c073f1dd738aa598ede55) | _ | ferric uptake regulation protein |
| [OCAR_4525](https://www.genoscope.cns.fr/agc/mage/wwwpkgdb/Info/getInfoLabel.php?id=3484339&wwwpkgdb=fc2733d6468c073f1dd738aa598ede55&nocache=62541e24b6e240f187ac920cab60f9da&dir=&wwwpkgdb=fc2733d6468c073f1dd738aa598ede55) | _ | nitrogen-fixing NifU domain protein |
| [OCAR_4526](https://www.genoscope.cns.fr/agc/mage/wwwpkgdb/Info/getInfoLabel.php?id=3484340&wwwpkgdb=fc2733d6468c073f1dd738aa598ede55&nocache=62541e24b6e240f187ac920cab60f9da&dir=&wwwpkgdb=fc2733d6468c073f1dd738aa598ede55) | trpS | tryptophanyl-tRNA synthetase |
| [OCAR_4528](https://www.genoscope.cns.fr/agc/mage/wwwpkgdb/Info/getInfoLabel.php?id=3482369&wwwpkgdb=fc2733d6468c073f1dd738aa598ede55&nocache=62541e24b6e240f187ac920cab60f9da&dir=&wwwpkgdb=fc2733d6468c073f1dd738aa598ede55) | _ | PfkB |
| [OCAR_4531](https://www.genoscope.cns.fr/agc/mage/wwwpkgdb/Info/getInfoLabel.php?id=3484343&wwwpkgdb=fc2733d6468c073f1dd738aa598ede55&nocache=62541e24b6e240f187ac920cab60f9da&dir=&wwwpkgdb=fc2733d6468c073f1dd738aa598ede55) | _ | pirin domain protein |
| [OCAR_4532](https://www.genoscope.cns.fr/agc/mage/wwwpkgdb/Info/getInfoLabel.php?id=3484344&wwwpkgdb=fc2733d6468c073f1dd738aa598ede55&nocache=62541e24b6e240f187ac920cab60f9da&dir=&wwwpkgdb=fc2733d6468c073f1dd738aa598ede55) | gyrB | DNA gyrase, B subunit |
| [OCAR_4534](https://www.genoscope.cns.fr/agc/mage/wwwpkgdb/Info/getInfoLabel.php?id=3484345&wwwpkgdb=fc2733d6468c073f1dd738aa598ede55&nocache=62541e24b6e240f187ac920cab60f9da&dir=&wwwpkgdb=fc2733d6468c073f1dd738aa598ede55) | _ | DNA replication and repair protein RecF |
| [OCAR_4536](https://www.genoscope.cns.fr/agc/mage/wwwpkgdb/Info/getInfoLabel.php?id=3484347&wwwpkgdb=fc2733d6468c073f1dd738aa598ede55&nocache=62541e24b6e240f187ac920cab60f9da&dir=&wwwpkgdb=fc2733d6468c073f1dd738aa598ede55) | dnaN | DNA polymerase III, beta subunit |
| [OCAR_4538](https://www.genoscope.cns.fr/agc/mage/wwwpkgdb/Info/getInfoLabel.php?id=3484348&wwwpkgdb=fc2733d6468c073f1dd738aa598ede55&nocache=62541e24b6e240f187ac920cab60f9da&dir=&wwwpkgdb=fc2733d6468c073f1dd738aa598ede55) | dnaA | chromosomal replication initiator protein DnaA |
| [OCAR_4540](https://www.genoscope.cns.fr/agc/mage/wwwpkgdb/Info/getInfoLabel.php?id=3484350&wwwpkgdb=fc2733d6468c073f1dd738aa598ede55&nocache=62541e24b6e240f187ac920cab60f9da&dir=&wwwpkgdb=fc2733d6468c073f1dd738aa598ede55) | rpsT | 30S ribosomal protein S20 |
| [OCAR_4568](https://www.genoscope.cns.fr/agc/mage/wwwpkgdb/Info/getInfoLabel.php?id=3484364&wwwpkgdb=fc2733d6468c073f1dd738aa598ede55&nocache=62541e24b6e240f187ac920cab60f9da&dir=&wwwpkgdb=fc2733d6468c073f1dd738aa598ede55) | _ | heme exporter protein CcmC |
| [OCAR_4569](https://www.genoscope.cns.fr/agc/mage/wwwpkgdb/Info/getInfoLabel.php?id=3484365&wwwpkgdb=fc2733d6468c073f1dd738aa598ede55&nocache=62541e24b6e240f187ac920cab60f9da&dir=&wwwpkgdb=fc2733d6468c073f1dd738aa598ede55) | ccmB | heme exporter protein CcmB |
| [OCAR_4571](https://www.genoscope.cns.fr/agc/mage/wwwpkgdb/Info/getInfoLabel.php?id=3482387&wwwpkgdb=fc2733d6468c073f1dd738aa598ede55&nocache=62541e24b6e240f187ac920cab60f9da&dir=&wwwpkgdb=fc2733d6468c073f1dd738aa598ede55) | acnA | aconitate hydratase 1 |
| [OCAR_4573](https://www.genoscope.cns.fr/agc/mage/wwwpkgdb/Info/getInfoLabel.php?id=3482389&wwwpkgdb=fc2733d6468c073f1dd738aa598ede55&nocache=62541e24b6e240f187ac920cab60f9da&dir=&wwwpkgdb=fc2733d6468c073f1dd738aa598ede55) | _ | hypothetical protein |
| [OCAR_4575](https://www.genoscope.cns.fr/agc/mage/wwwpkgdb/Info/getInfoLabel.php?id=3484368&wwwpkgdb=fc2733d6468c073f1dd738aa598ede55&nocache=62541e24b6e240f187ac920cab60f9da&dir=&wwwpkgdb=fc2733d6468c073f1dd738aa598ede55) | _ | integral membrane protein |
| [OCAR_4577](https://www.genoscope.cns.fr/agc/mage/wwwpkgdb/Info/getInfoLabel.php?id=3484370&wwwpkgdb=fc2733d6468c073f1dd738aa598ede55&nocache=62541e24b6e240f187ac920cab60f9da&dir=&wwwpkgdb=fc2733d6468c073f1dd738aa598ede55) | _ | lipoprotein-releasing system ATP-binding protein LolD |
| [OCAR_4579](https://www.genoscope.cns.fr/agc/mage/wwwpkgdb/Info/getInfoLabel.php?id=3482391&wwwpkgdb=fc2733d6468c073f1dd738aa598ede55&nocache=62541e24b6e240f187ac920cab60f9da&dir=&wwwpkgdb=fc2733d6468c073f1dd738aa598ede55) | _ | 2'-5' RNA ligase |
| [OCAR_4581](https://www.genoscope.cns.fr/agc/mage/wwwpkgdb/Info/getInfoLabel.php?id=3482393&wwwpkgdb=fc2733d6468c073f1dd738aa598ede55&nocache=62541e24b6e240f187ac920cab60f9da&dir=&wwwpkgdb=fc2733d6468c073f1dd738aa598ede55) | _ | malate dehydrogenase |
| [OCAR_4582](https://www.genoscope.cns.fr/agc/mage/wwwpkgdb/Info/getInfoLabel.php?id=3482394&wwwpkgdb=fc2733d6468c073f1dd738aa598ede55&nocache=62541e24b6e240f187ac920cab60f9da&dir=&wwwpkgdb=fc2733d6468c073f1dd738aa598ede55) | sucA | oxoglutarate dehydrogenase |
| [OCAR_4583](https://www.genoscope.cns.fr/agc/mage/wwwpkgdb/Info/getInfoLabel.php?id=3482395&wwwpkgdb=fc2733d6468c073f1dd738aa598ede55&nocache=62541e24b6e240f187ac920cab60f9da&dir=&wwwpkgdb=fc2733d6468c073f1dd738aa598ede55) | sucB | dihydrolipoyllysine-residue succinyltransferase |
| [OCAR_4585](https://www.genoscope.cns.fr/agc/mage/wwwpkgdb/Info/getInfoLabel.php?id=3482397&wwwpkgdb=fc2733d6468c073f1dd738aa598ede55&nocache=62541e24b6e240f187ac920cab60f9da&dir=&wwwpkgdb=fc2733d6468c073f1dd738aa598ede55) | lpdA | dihydrolipoyl dehydrogenase |
| [OCAR_4586](https://www.genoscope.cns.fr/agc/mage/wwwpkgdb/Info/getInfoLabel.php?id=3484371&wwwpkgdb=fc2733d6468c073f1dd738aa598ede55&nocache=62541e24b6e240f187ac920cab60f9da&dir=&wwwpkgdb=fc2733d6468c073f1dd738aa598ede55) | _ | hypothetical protein |
| [OCAR_4587](https://www.genoscope.cns.fr/agc/mage/wwwpkgdb/Info/getInfoLabel.php?id=3484372&wwwpkgdb=fc2733d6468c073f1dd738aa598ede55&nocache=62541e24b6e240f187ac920cab60f9da&dir=&wwwpkgdb=fc2733d6468c073f1dd738aa598ede55) | xerC | tyrosine recombinase XerC |
| [OCAR_4588](https://www.genoscope.cns.fr/agc/mage/wwwpkgdb/Info/getInfoLabel.php?id=3482398&wwwpkgdb=fc2733d6468c073f1dd738aa598ede55&nocache=62541e24b6e240f187ac920cab60f9da&dir=&wwwpkgdb=fc2733d6468c073f1dd738aa598ede55) | priA | primosomal protein N' |
| [OCAR_4589](https://www.genoscope.cns.fr/agc/mage/wwwpkgdb/Info/getInfoLabel.php?id=3484373&wwwpkgdb=fc2733d6468c073f1dd738aa598ede55&nocache=62541e24b6e240f187ac920cab60f9da&dir=&wwwpkgdb=fc2733d6468c073f1dd738aa598ede55) | _ | rare lipoprotein A |
| [OCAR_4591](https://www.genoscope.cns.fr/agc/mage/wwwpkgdb/Info/getInfoLabel.php?id=3482399&wwwpkgdb=fc2733d6468c073f1dd738aa598ede55&nocache=62541e24b6e240f187ac920cab60f9da&dir=&wwwpkgdb=fc2733d6468c073f1dd738aa598ede55) | atpH | ATP synthase subunit delta |
| [OCAR_4592](https://www.genoscope.cns.fr/agc/mage/wwwpkgdb/Info/getInfoLabel.php?id=3482400&wwwpkgdb=fc2733d6468c073f1dd738aa598ede55&nocache=62541e24b6e240f187ac920cab60f9da&dir=&wwwpkgdb=fc2733d6468c073f1dd738aa598ede55) | atpA | ATP synthase subunit alpha |
| [OCAR_4593](https://www.genoscope.cns.fr/agc/mage/wwwpkgdb/Info/getInfoLabel.php?id=3482401&wwwpkgdb=fc2733d6468c073f1dd738aa598ede55&nocache=62541e24b6e240f187ac920cab60f9da&dir=&wwwpkgdb=fc2733d6468c073f1dd738aa598ede55) | atpG | ATP synthase F1, gamma subunit |
| [OCAR_4595](https://www.genoscope.cns.fr/agc/mage/wwwpkgdb/Info/getInfoLabel.php?id=3482403&wwwpkgdb=fc2733d6468c073f1dd738aa598ede55&nocache=62541e24b6e240f187ac920cab60f9da&dir=&wwwpkgdb=fc2733d6468c073f1dd738aa598ede55) | atpD | ATP synthase subunit beta |
| [OCAR_4596](https://www.genoscope.cns.fr/agc/mage/wwwpkgdb/Info/getInfoLabel.php?id=3482404&wwwpkgdb=fc2733d6468c073f1dd738aa598ede55&nocache=62541e24b6e240f187ac920cab60f9da&dir=&wwwpkgdb=fc2733d6468c073f1dd738aa598ede55) | atpC | ATP synthase epsilon chain |
| [OCAR_4597](https://www.genoscope.cns.fr/agc/mage/wwwpkgdb/Info/getInfoLabel.php?id=3484375&wwwpkgdb=fc2733d6468c073f1dd738aa598ede55&nocache=62541e24b6e240f187ac920cab60f9da&dir=&wwwpkgdb=fc2733d6468c073f1dd738aa598ede55) | rppH | RNA pyrophosphohydrolase |
| [OCAR_4600](https://www.genoscope.cns.fr/agc/mage/wwwpkgdb/Info/getInfoLabel.php?id=3484378&wwwpkgdb=fc2733d6468c073f1dd738aa598ede55&nocache=62541e24b6e240f187ac920cab60f9da&dir=&wwwpkgdb=fc2733d6468c073f1dd738aa598ede55) | _ | carboxy--processing protease |
| [OCAR_4603](https://www.genoscope.cns.fr/agc/mage/wwwpkgdb/Info/getInfoLabel.php?id=3484381&wwwpkgdb=fc2733d6468c073f1dd738aa598ede55&nocache=62541e24b6e240f187ac920cab60f9da&dir=&wwwpkgdb=fc2733d6468c073f1dd738aa598ede55) | _ | iojap protein family |
| [OCAR_4605](https://www.genoscope.cns.fr/agc/mage/wwwpkgdb/Info/getInfoLabel.php?id=3484383&wwwpkgdb=fc2733d6468c073f1dd738aa598ede55&nocache=62541e24b6e240f187ac920cab60f9da&dir=&wwwpkgdb=fc2733d6468c073f1dd738aa598ede55) | proA | Gamma-glutamyl phosphate reductase |
| [OCAR_4606](https://www.genoscope.cns.fr/agc/mage/wwwpkgdb/Info/getInfoLabel.php?id=3484384&wwwpkgdb=fc2733d6468c073f1dd738aa598ede55&nocache=62541e24b6e240f187ac920cab60f9da&dir=&wwwpkgdb=fc2733d6468c073f1dd738aa598ede55) | proB | glutamate 5-kinase |
| [OCAR_4607](https://www.genoscope.cns.fr/agc/mage/wwwpkgdb/Info/getInfoLabel.php?id=3484385&wwwpkgdb=fc2733d6468c073f1dd738aa598ede55&nocache=62541e24b6e240f187ac920cab60f9da&dir=&wwwpkgdb=fc2733d6468c073f1dd738aa598ede55) | _ | GTP-binding protein Obg/CgtA |
| [OCAR_4609](https://www.genoscope.cns.fr/agc/mage/wwwpkgdb/Info/getInfoLabel.php?id=3484387&wwwpkgdb=fc2733d6468c073f1dd738aa598ede55&nocache=62541e24b6e240f187ac920cab60f9da&dir=&wwwpkgdb=fc2733d6468c073f1dd738aa598ede55) | rpmA | 50S ribosomal protein L27 |
| [OCAR_4610](https://www.genoscope.cns.fr/agc/mage/wwwpkgdb/Info/getInfoLabel.php?id=3484388&wwwpkgdb=fc2733d6468c073f1dd738aa598ede55&nocache=62541e24b6e240f187ac920cab60f9da&dir=&wwwpkgdb=fc2733d6468c073f1dd738aa598ede55) | rplU | 50S ribosomal protein L21 |
| [OCAR_4611](https://www.genoscope.cns.fr/agc/mage/wwwpkgdb/Info/getInfoLabel.php?id=3482405&wwwpkgdb=fc2733d6468c073f1dd738aa598ede55&nocache=62541e24b6e240f187ac920cab60f9da&dir=&wwwpkgdb=fc2733d6468c073f1dd738aa598ede55) | _ | glucokinase |
| [OCAR_4612](https://www.genoscope.cns.fr/agc/mage/wwwpkgdb/Info/getInfoLabel.php?id=3482406&wwwpkgdb=fc2733d6468c073f1dd738aa598ede55&nocache=62541e24b6e240f187ac920cab60f9da&dir=&wwwpkgdb=fc2733d6468c073f1dd738aa598ede55) | _ | major facilitator superfamily MFS_1 |
| [OCAR_4614](https://www.genoscope.cns.fr/agc/mage/wwwpkgdb/Info/getInfoLabel.php?id=3482408&wwwpkgdb=fc2733d6468c073f1dd738aa598ede55&nocache=62541e24b6e240f187ac920cab60f9da&dir=&wwwpkgdb=fc2733d6468c073f1dd738aa598ede55) | otsA | alpha,alpha-trehalose-phosphate synthase |
| [OCAR_4633](https://www.genoscope.cns.fr/agc/mage/wwwpkgdb/Info/getInfoLabel.php?id=3482424&wwwpkgdb=fc2733d6468c073f1dd738aa598ede55&nocache=62541e24b6e240f187ac920cab60f9da&dir=&wwwpkgdb=fc2733d6468c073f1dd738aa598ede55) | feoB | ferrous iron transport protein B |
| [OCAR_4634](https://www.genoscope.cns.fr/agc/mage/wwwpkgdb/Info/getInfoLabel.php?id=3484392&wwwpkgdb=fc2733d6468c073f1dd738aa598ede55&nocache=62541e24b6e240f187ac920cab60f9da&dir=&wwwpkgdb=fc2733d6468c073f1dd738aa598ede55) | _ | glyoxalase/bleomycin resistance protein/dioxygenase |
| [OCAR_4635](https://www.genoscope.cns.fr/agc/mage/wwwpkgdb/Info/getInfoLabel.php?id=3484393&wwwpkgdb=fc2733d6468c073f1dd738aa598ede55&nocache=62541e24b6e240f187ac920cab60f9da&dir=&wwwpkgdb=fc2733d6468c073f1dd738aa598ede55) | _ | general L-amino acid-binding periplasmic protein AapJ |
| [OCAR_4646](https://www.genoscope.cns.fr/agc/mage/wwwpkgdb/Info/getInfoLabel.php?id=3482430&wwwpkgdb=fc2733d6468c073f1dd738aa598ede55&nocache=62541e24b6e240f187ac920cab60f9da&dir=&wwwpkgdb=fc2733d6468c073f1dd738aa598ede55) | pstS | phosphate ABC transporter |
| [OCAR_4647](https://www.genoscope.cns.fr/agc/mage/wwwpkgdb/Info/getInfoLabel.php?id=3482431&wwwpkgdb=fc2733d6468c073f1dd738aa598ede55&nocache=62541e24b6e240f187ac920cab60f9da&dir=&wwwpkgdb=fc2733d6468c073f1dd738aa598ede55) | pstC | phosphate ABC transporter |
| [OCAR_4648](https://www.genoscope.cns.fr/agc/mage/wwwpkgdb/Info/getInfoLabel.php?id=3482432&wwwpkgdb=fc2733d6468c073f1dd738aa598ede55&nocache=62541e24b6e240f187ac920cab60f9da&dir=&wwwpkgdb=fc2733d6468c073f1dd738aa598ede55) | pstA | phosphate ABC transporter |
| [OCAR_4649](https://www.genoscope.cns.fr/agc/mage/wwwpkgdb/Info/getInfoLabel.php?id=3482433&wwwpkgdb=fc2733d6468c073f1dd738aa598ede55&nocache=62541e24b6e240f187ac920cab60f9da&dir=&wwwpkgdb=fc2733d6468c073f1dd738aa598ede55) | pstB | phosphate ABC transporter |
| [OCAR_4650](https://www.genoscope.cns.fr/agc/mage/wwwpkgdb/Info/getInfoLabel.php?id=3482434&wwwpkgdb=fc2733d6468c073f1dd738aa598ede55&nocache=62541e24b6e240f187ac920cab60f9da&dir=&wwwpkgdb=fc2733d6468c073f1dd738aa598ede55) | phoU | phosphate transport system regulatory protein PhoU |
| [OCAR_4651](https://www.genoscope.cns.fr/agc/mage/wwwpkgdb/Info/getInfoLabel.php?id=3482435&wwwpkgdb=fc2733d6468c073f1dd738aa598ede55&nocache=62541e24b6e240f187ac920cab60f9da&dir=&wwwpkgdb=fc2733d6468c073f1dd738aa598ede55) | phoB | phosphate regulon transcriptional regulatory protein PhoB |
| [OCAR_4652](https://www.genoscope.cns.fr/agc/mage/wwwpkgdb/Info/getInfoLabel.php?id=3484399&wwwpkgdb=fc2733d6468c073f1dd738aa598ede55&nocache=62541e24b6e240f187ac920cab60f9da&dir=&wwwpkgdb=fc2733d6468c073f1dd738aa598ede55) | _ | GcrA cell cycle regulator |
| [OCAR_4654](https://www.genoscope.cns.fr/agc/mage/wwwpkgdb/Info/getInfoLabel.php?id=3482436&wwwpkgdb=fc2733d6468c073f1dd738aa598ede55&nocache=62541e24b6e240f187ac920cab60f9da&dir=&wwwpkgdb=fc2733d6468c073f1dd738aa598ede55) | argD | acetylornithine aminotransferase |
| [OCAR_4655](https://www.genoscope.cns.fr/agc/mage/wwwpkgdb/Info/getInfoLabel.php?id=3482437&wwwpkgdb=fc2733d6468c073f1dd738aa598ede55&nocache=62541e24b6e240f187ac920cab60f9da&dir=&wwwpkgdb=fc2733d6468c073f1dd738aa598ede55) | argF | ornithine carbamoyltransferase |
| [OCAR_4656](https://www.genoscope.cns.fr/agc/mage/wwwpkgdb/Info/getInfoLabel.php?id=3482438&wwwpkgdb=fc2733d6468c073f1dd738aa598ede55&nocache=62541e24b6e240f187ac920cab60f9da&dir=&wwwpkgdb=fc2733d6468c073f1dd738aa598ede55) | hslO | chaperonin HslO |
| [OCAR_4658](https://www.genoscope.cns.fr/agc/mage/wwwpkgdb/Info/getInfoLabel.php?id=3482439&wwwpkgdb=fc2733d6468c073f1dd738aa598ede55&nocache=62541e24b6e240f187ac920cab60f9da&dir=&wwwpkgdb=fc2733d6468c073f1dd738aa598ede55) | _ | acyltransferase 3 |
| [OCAR_4661](https://www.genoscope.cns.fr/agc/mage/wwwpkgdb/Info/getInfoLabel.php?id=3484402&wwwpkgdb=fc2733d6468c073f1dd738aa598ede55&nocache=62541e24b6e240f187ac920cab60f9da&dir=&wwwpkgdb=fc2733d6468c073f1dd738aa598ede55) | metZ | O-succinylhomoserine sulfhydrylase |
| [OCAR_4662](https://www.genoscope.cns.fr/agc/mage/wwwpkgdb/Info/getInfoLabel.php?id=3482442&wwwpkgdb=fc2733d6468c073f1dd738aa598ede55&nocache=62541e24b6e240f187ac920cab60f9da&dir=&wwwpkgdb=fc2733d6468c073f1dd738aa598ede55) | _ | 2-deoxycytidine 5-triphosphate deaminase |
| [OCAR_4664](https://www.genoscope.cns.fr/agc/mage/wwwpkgdb/Info/getInfoLabel.php?id=3482444&wwwpkgdb=fc2733d6468c073f1dd738aa598ede55&nocache=62541e24b6e240f187ac920cab60f9da&dir=&wwwpkgdb=fc2733d6468c073f1dd738aa598ede55) | _ | hypothetical protein |
| [OCAR_4667](https://www.genoscope.cns.fr/agc/mage/wwwpkgdb/Info/getInfoLabel.php?id=3482446&wwwpkgdb=fc2733d6468c073f1dd738aa598ede55&nocache=62541e24b6e240f187ac920cab60f9da&dir=&wwwpkgdb=fc2733d6468c073f1dd738aa598ede55) | _ | extracellular solute-binding protein, family 3 |
| [OCAR_4668](https://www.genoscope.cns.fr/agc/mage/wwwpkgdb/Info/getInfoLabel.php?id=3482447&wwwpkgdb=fc2733d6468c073f1dd738aa598ede55&nocache=62541e24b6e240f187ac920cab60f9da&dir=&wwwpkgdb=fc2733d6468c073f1dd738aa598ede55) | lysS | lysyl-tRNA synthetase |
| [OCAR_4674](https://www.genoscope.cns.fr/agc/mage/wwwpkgdb/Info/getInfoLabel.php?id=3482450&wwwpkgdb=fc2733d6468c073f1dd738aa598ede55&nocache=62541e24b6e240f187ac920cab60f9da&dir=&wwwpkgdb=fc2733d6468c073f1dd738aa598ede55) | pyrD | dihydroorotate oxidase |
| [OCAR_4678](https://www.genoscope.cns.fr/agc/mage/wwwpkgdb/Info/getInfoLabel.php?id=3482452&wwwpkgdb=fc2733d6468c073f1dd738aa598ede55&nocache=62541e24b6e240f187ac920cab60f9da&dir=&wwwpkgdb=fc2733d6468c073f1dd738aa598ede55) | _ | transcriptional regulator, MarR family |
| [OCAR_4679](https://www.genoscope.cns.fr/agc/mage/wwwpkgdb/Info/getInfoLabel.php?id=3482453&wwwpkgdb=fc2733d6468c073f1dd738aa598ede55&nocache=62541e24b6e240f187ac920cab60f9da&dir=&wwwpkgdb=fc2733d6468c073f1dd738aa598ede55) | _ | glutamate--cysteine ligase |
| [OCAR_4682](https://www.genoscope.cns.fr/agc/mage/wwwpkgdb/Info/getInfoLabel.php?id=3484410&wwwpkgdb=fc2733d6468c073f1dd738aa598ede55&nocache=62541e24b6e240f187ac920cab60f9da&dir=&wwwpkgdb=fc2733d6468c073f1dd738aa598ede55) | _ | peptidase U62, modulator of DNA gyrase |
| [OCAR_4683](https://www.genoscope.cns.fr/agc/mage/wwwpkgdb/Info/getInfoLabel.php?id=3484411&wwwpkgdb=fc2733d6468c073f1dd738aa598ede55&nocache=62541e24b6e240f187ac920cab60f9da&dir=&wwwpkgdb=fc2733d6468c073f1dd738aa598ede55) | _ | invasion associated locus B |
| [OCAR_4686](https://www.genoscope.cns.fr/agc/mage/wwwpkgdb/Info/getInfoLabel.php?id=3482456&wwwpkgdb=fc2733d6468c073f1dd738aa598ede55&nocache=62541e24b6e240f187ac920cab60f9da&dir=&wwwpkgdb=fc2733d6468c073f1dd738aa598ede55) | ctaD | cytochrome c oxidase, subunit I |
| [OCAR_4687](https://www.genoscope.cns.fr/agc/mage/wwwpkgdb/Info/getInfoLabel.php?id=3482457&wwwpkgdb=fc2733d6468c073f1dd738aa598ede55&nocache=62541e24b6e240f187ac920cab60f9da&dir=&wwwpkgdb=fc2733d6468c073f1dd738aa598ede55) | ctaB | Protoheme IX farnesyltransferase |
| [OCAR_4690](https://www.genoscope.cns.fr/agc/mage/wwwpkgdb/Info/getInfoLabel.php?id=3482460&wwwpkgdb=fc2733d6468c073f1dd738aa598ede55&nocache=62541e24b6e240f187ac920cab60f9da&dir=&wwwpkgdb=fc2733d6468c073f1dd738aa598ede55) | _ | cytochrome c oxidase subunit 3 |
| [OCAR_4693](https://www.genoscope.cns.fr/agc/mage/wwwpkgdb/Info/getInfoLabel.php?id=3482463&wwwpkgdb=fc2733d6468c073f1dd738aa598ede55&nocache=62541e24b6e240f187ac920cab60f9da&dir=&wwwpkgdb=fc2733d6468c073f1dd738aa598ede55) | thrC | threonine synthase |
| [OCAR_4694](https://www.genoscope.cns.fr/agc/mage/wwwpkgdb/Info/getInfoLabel.php?id=3482464&wwwpkgdb=fc2733d6468c073f1dd738aa598ede55&nocache=62541e24b6e240f187ac920cab60f9da&dir=&wwwpkgdb=fc2733d6468c073f1dd738aa598ede55) | _ | peptidase M16 domain protein |
| [OCAR_4695](https://www.genoscope.cns.fr/agc/mage/wwwpkgdb/Info/getInfoLabel.php?id=3482465&wwwpkgdb=fc2733d6468c073f1dd738aa598ede55&nocache=62541e24b6e240f187ac920cab60f9da&dir=&wwwpkgdb=fc2733d6468c073f1dd738aa598ede55) | _ | ribosomal-protein-alanine N-acetyltransferase |
| [OCAR_4699](https://www.genoscope.cns.fr/agc/mage/wwwpkgdb/Info/getInfoLabel.php?id=3484415&wwwpkgdb=fc2733d6468c073f1dd738aa598ede55&nocache=62541e24b6e240f187ac920cab60f9da&dir=&wwwpkgdb=fc2733d6468c073f1dd738aa598ede55) | _ | hypothetical protein |
| [OCAR_4704](https://www.genoscope.cns.fr/agc/mage/wwwpkgdb/Info/getInfoLabel.php?id=3482468&wwwpkgdb=fc2733d6468c073f1dd738aa598ede55&nocache=62541e24b6e240f187ac920cab60f9da&dir=&wwwpkgdb=fc2733d6468c073f1dd738aa598ede55) | _ | two component transcriptional regulator, LuxR family |
| [OCAR_4703](https://www.genoscope.cns.fr/agc/mage/wwwpkgdb/Info/getInfoLabel.php?id=3484418&wwwpkgdb=fc2733d6468c073f1dd738aa598ede55&nocache=62541e24b6e240f187ac920cab60f9da&dir=&wwwpkgdb=fc2733d6468c073f1dd738aa598ede55) | _ | two-component hybrid sensor and regulator |
| [OCAR_4706](https://www.genoscope.cns.fr/agc/mage/wwwpkgdb/Info/getInfoLabel.php?id=3484419&wwwpkgdb=fc2733d6468c073f1dd738aa598ede55&nocache=62541e24b6e240f187ac920cab60f9da&dir=&wwwpkgdb=fc2733d6468c073f1dd738aa598ede55) | hemA | 5-aminolevulinic acid synthase |
| [OCAR_4707](https://www.genoscope.cns.fr/agc/mage/wwwpkgdb/Info/getInfoLabel.php?id=3484420&wwwpkgdb=fc2733d6468c073f1dd738aa598ede55&nocache=62541e24b6e240f187ac920cab60f9da&dir=&wwwpkgdb=fc2733d6468c073f1dd738aa598ede55) | bioB | Biotin synthase |
| [OCAR_4730](https://www.genoscope.cns.fr/agc/mage/wwwpkgdb/Info/getInfoLabel.php?id=3482487&wwwpkgdb=fc2733d6468c073f1dd738aa598ede55&nocache=62541e24b6e240f187ac920cab60f9da&dir=&wwwpkgdb=fc2733d6468c073f1dd738aa598ede55) | hisD | histidinol dehydrogenase |
| [OCAR_4731](https://www.genoscope.cns.fr/agc/mage/wwwpkgdb/Info/getInfoLabel.php?id=3482488&wwwpkgdb=fc2733d6468c073f1dd738aa598ede55&nocache=62541e24b6e240f187ac920cab60f9da&dir=&wwwpkgdb=fc2733d6468c073f1dd738aa598ede55) | _ | hypothetical protein |
| [OCAR_4733](https://www.genoscope.cns.fr/agc/mage/wwwpkgdb/Info/getInfoLabel.php?id=3482490&wwwpkgdb=fc2733d6468c073f1dd738aa598ede55&nocache=62541e24b6e240f187ac920cab60f9da&dir=&wwwpkgdb=fc2733d6468c073f1dd738aa598ede55) | maf | septum formation protein Maf |
| [OCAR_4734](https://www.genoscope.cns.fr/agc/mage/wwwpkgdb/Info/getInfoLabel.php?id=3482491&wwwpkgdb=fc2733d6468c073f1dd738aa598ede55&nocache=62541e24b6e240f187ac920cab60f9da&dir=&wwwpkgdb=fc2733d6468c073f1dd738aa598ede55) | _ | hypothetical protein |
| [OCAR_4746](https://www.genoscope.cns.fr/agc/mage/wwwpkgdb/Info/getInfoLabel.php?id=3484432&wwwpkgdb=fc2733d6468c073f1dd738aa598ede55&nocache=62541e24b6e240f187ac920cab60f9da&dir=&wwwpkgdb=fc2733d6468c073f1dd738aa598ede55) | _ | HNH endonuclease |
| [OCAR_4752](https://www.genoscope.cns.fr/agc/mage/wwwpkgdb/Info/getInfoLabel.php?id=3482501&wwwpkgdb=fc2733d6468c073f1dd738aa598ede55&nocache=62541e24b6e240f187ac920cab60f9da&dir=&wwwpkgdb=fc2733d6468c073f1dd738aa598ede55) | _ | ATP:cob(I)alamin adenosyltransferase |
| [OCAR_4753](https://www.genoscope.cns.fr/agc/mage/wwwpkgdb/Info/getInfoLabel.php?id=3482502&wwwpkgdb=fc2733d6468c073f1dd738aa598ede55&nocache=62541e24b6e240f187ac920cab60f9da&dir=&wwwpkgdb=fc2733d6468c073f1dd738aa598ede55) | _ | electron transfer flavoprotein subunit beta (beta-etf) |
| [OCAR_4754](https://www.genoscope.cns.fr/agc/mage/wwwpkgdb/Info/getInfoLabel.php?id=3482503&wwwpkgdb=fc2733d6468c073f1dd738aa598ede55&nocache=62541e24b6e240f187ac920cab60f9da&dir=&wwwpkgdb=fc2733d6468c073f1dd738aa598ede55) | _ | electron transfer flavoprotein subunit alpha (alpha-etf) |
| [OCAR_4755](https://www.genoscope.cns.fr/agc/mage/wwwpkgdb/Info/getInfoLabel.php?id=3482504&wwwpkgdb=fc2733d6468c073f1dd738aa598ede55&nocache=62541e24b6e240f187ac920cab60f9da&dir=&wwwpkgdb=fc2733d6468c073f1dd738aa598ede55) | _ | 3-hydroxybutyryl-coa dehydrogenase |
| [OCAR_4760](https://www.genoscope.cns.fr/agc/mage/wwwpkgdb/Info/getInfoLabel.php?id=3482506&wwwpkgdb=fc2733d6468c073f1dd738aa598ede55&nocache=62541e24b6e240f187ac920cab60f9da&dir=&wwwpkgdb=fc2733d6468c073f1dd738aa598ede55) | argH | argininosuccinate lyase |
| [OCAR_4762](https://www.genoscope.cns.fr/agc/mage/wwwpkgdb/Info/getInfoLabel.php?id=3482508&wwwpkgdb=fc2733d6468c073f1dd738aa598ede55&nocache=62541e24b6e240f187ac920cab60f9da&dir=&wwwpkgdb=fc2733d6468c073f1dd738aa598ede55) | lysA | diaminopimelate decarboxylase |
| [OCAR_4764](https://www.genoscope.cns.fr/agc/mage/wwwpkgdb/Info/getInfoLabel.php?id=3484437&wwwpkgdb=fc2733d6468c073f1dd738aa598ede55&nocache=62541e24b6e240f187ac920cab60f9da&dir=&wwwpkgdb=fc2733d6468c073f1dd738aa598ede55) | _ | response regulator receiver protein |
| [OCAR_4766](https://www.genoscope.cns.fr/agc/mage/wwwpkgdb/Info/getInfoLabel.php?id=3482510&wwwpkgdb=fc2733d6468c073f1dd738aa598ede55&nocache=62541e24b6e240f187ac920cab60f9da&dir=&wwwpkgdb=fc2733d6468c073f1dd738aa598ede55) | ftsE | cell division ATP-binding protein FtsE |
| [OCAR_4767](https://www.genoscope.cns.fr/agc/mage/wwwpkgdb/Info/getInfoLabel.php?id=3482511&wwwpkgdb=fc2733d6468c073f1dd738aa598ede55&nocache=62541e24b6e240f187ac920cab60f9da&dir=&wwwpkgdb=fc2733d6468c073f1dd738aa598ede55) | _ | cell division protein |
| [OCAR_4772](https://www.genoscope.cns.fr/agc/mage/wwwpkgdb/Info/getInfoLabel.php?id=3484440&wwwpkgdb=fc2733d6468c073f1dd738aa598ede55&nocache=62541e24b6e240f187ac920cab60f9da&dir=&wwwpkgdb=fc2733d6468c073f1dd738aa598ede55) | _ | prephenate dehydrogenase |
| [OCAR_4774](https://www.genoscope.cns.fr/agc/mage/wwwpkgdb/Info/getInfoLabel.php?id=3484442&wwwpkgdb=fc2733d6468c073f1dd738aa598ede55&nocache=62541e24b6e240f187ac920cab60f9da&dir=&wwwpkgdb=fc2733d6468c073f1dd738aa598ede55) | _ | chorismate mutase |
| [OCAR_4776](https://www.genoscope.cns.fr/agc/mage/wwwpkgdb/Info/getInfoLabel.php?id=3482515&wwwpkgdb=fc2733d6468c073f1dd738aa598ede55&nocache=62541e24b6e240f187ac920cab60f9da&dir=&wwwpkgdb=fc2733d6468c073f1dd738aa598ede55) | metX | homoserine O-acetyltransferase |
| [OCAR_4777](https://www.genoscope.cns.fr/agc/mage/wwwpkgdb/Info/getInfoLabel.php?id=3482516&wwwpkgdb=fc2733d6468c073f1dd738aa598ede55&nocache=62541e24b6e240f187ac920cab60f9da&dir=&wwwpkgdb=fc2733d6468c073f1dd738aa598ede55) | metW | methionine biosynthesis protein MetW |
| [OCAR_4780](https://www.genoscope.cns.fr/agc/mage/wwwpkgdb/Info/getInfoLabel.php?id=3482518&wwwpkgdb=fc2733d6468c073f1dd738aa598ede55&nocache=62541e24b6e240f187ac920cab60f9da&dir=&wwwpkgdb=fc2733d6468c073f1dd738aa598ede55) | clpB | ATP-dependent chaperone ClpB |
| [OCAR_4781](https://www.genoscope.cns.fr/agc/mage/wwwpkgdb/Info/getInfoLabel.php?id=3484445&wwwpkgdb=fc2733d6468c073f1dd738aa598ede55&nocache=62541e24b6e240f187ac920cab60f9da&dir=&wwwpkgdb=fc2733d6468c073f1dd738aa598ede55) | _ | peptidase M23B |
| [OCAR_4902](https://www.genoscope.cns.fr/agc/mage/wwwpkgdb/Info/getInfoLabel.php?id=3482605&wwwpkgdb=fc2733d6468c073f1dd738aa598ede55&nocache=62541e24b6e240f187ac920cab60f9da&dir=&wwwpkgdb=fc2733d6468c073f1dd738aa598ede55) | _ | superoxide dismutase |
| [OCAR_4905](https://www.genoscope.cns.fr/agc/mage/wwwpkgdb/Info/getInfoLabel.php?id=3482608&wwwpkgdb=fc2733d6468c073f1dd738aa598ede55&nocache=62541e24b6e240f187ac920cab60f9da&dir=&wwwpkgdb=fc2733d6468c073f1dd738aa598ede55) | _ | manganese transport system ATP-binding protein MntA |
| [OCAR_4906](https://www.genoscope.cns.fr/agc/mage/wwwpkgdb/Info/getInfoLabel.php?id=3482609&wwwpkgdb=fc2733d6468c073f1dd738aa598ede55&nocache=62541e24b6e240f187ac920cab60f9da&dir=&wwwpkgdb=fc2733d6468c073f1dd738aa598ede55) | _ | ABC-3 |
| [OCAR_4908](https://www.genoscope.cns.fr/agc/mage/wwwpkgdb/Info/getInfoLabel.php?id=3482611&wwwpkgdb=fc2733d6468c073f1dd738aa598ede55&nocache=62541e24b6e240f187ac920cab60f9da&dir=&wwwpkgdb=fc2733d6468c073f1dd738aa598ede55) | _ | cobalamin synthesis protein, P47K |
| [OCAR_4912](https://www.genoscope.cns.fr/agc/mage/wwwpkgdb/Info/getInfoLabel.php?id=3484483&wwwpkgdb=fc2733d6468c073f1dd738aa598ede55&nocache=62541e24b6e240f187ac920cab60f9da&dir=&wwwpkgdb=fc2733d6468c073f1dd738aa598ede55) | _ | homospermidine synthase (HSS) |
| [OCAR_4918](https://www.genoscope.cns.fr/agc/mage/wwwpkgdb/Info/getInfoLabel.php?id=3484487&wwwpkgdb=fc2733d6468c073f1dd738aa598ede55&nocache=62541e24b6e240f187ac920cab60f9da&dir=&wwwpkgdb=fc2733d6468c073f1dd738aa598ede55) | _ | ornithine decarboxylase |
| [OCAR_4920](https://www.genoscope.cns.fr/agc/mage/wwwpkgdb/Info/getInfoLabel.php?id=3484488&wwwpkgdb=fc2733d6468c073f1dd738aa598ede55&nocache=62541e24b6e240f187ac920cab60f9da&dir=&wwwpkgdb=fc2733d6468c073f1dd738aa598ede55) | _ | putative exported protein of unknown function |
| [OCAR_4921](https://www.genoscope.cns.fr/agc/mage/wwwpkgdb/Info/getInfoLabel.php?id=3482618&wwwpkgdb=fc2733d6468c073f1dd738aa598ede55&nocache=62541e24b6e240f187ac920cab60f9da&dir=&wwwpkgdb=fc2733d6468c073f1dd738aa598ede55) | _ | peptidyl-dipeptidase dcp |
| [OCAR_4924](https://www.genoscope.cns.fr/agc/mage/wwwpkgdb/Info/getInfoLabel.php?id=3482621&wwwpkgdb=fc2733d6468c073f1dd738aa598ede55&nocache=62541e24b6e240f187ac920cab60f9da&dir=&wwwpkgdb=fc2733d6468c073f1dd738aa598ede55) | hemH | ferrochelatase |
| [OCAR_4934](https://www.genoscope.cns.fr/agc/mage/wwwpkgdb/Info/getInfoLabel.php?id=3484490&wwwpkgdb=fc2733d6468c073f1dd738aa598ede55&nocache=62541e24b6e240f187ac920cab60f9da&dir=&wwwpkgdb=fc2733d6468c073f1dd738aa598ede55) | galU | UTP-glucose-1-phosphate uridylyltransferase |
| [OCAR_4938](https://www.genoscope.cns.fr/agc/mage/wwwpkgdb/Info/getInfoLabel.php?id=3484491&wwwpkgdb=fc2733d6468c073f1dd738aa598ede55&nocache=62541e24b6e240f187ac920cab60f9da&dir=&wwwpkgdb=fc2733d6468c073f1dd738aa598ede55) | _ | glutamate synthase |
| [OCAR_4939](https://www.genoscope.cns.fr/agc/mage/wwwpkgdb/Info/getInfoLabel.php?id=3484492&wwwpkgdb=fc2733d6468c073f1dd738aa598ede55&nocache=62541e24b6e240f187ac920cab60f9da&dir=&wwwpkgdb=fc2733d6468c073f1dd738aa598ede55) | _ | glutamate synthase |
| [OCAR_4943](https://www.genoscope.cns.fr/agc/mage/wwwpkgdb/Info/getInfoLabel.php?id=3484494&wwwpkgdb=fc2733d6468c073f1dd738aa598ede55&nocache=62541e24b6e240f187ac920cab60f9da&dir=&wwwpkgdb=fc2733d6468c073f1dd738aa598ede55) | _ | heat shock protein Hsp20 |
| [OCAR_4944](https://www.genoscope.cns.fr/agc/mage/wwwpkgdb/Info/getInfoLabel.php?id=3482635&wwwpkgdb=fc2733d6468c073f1dd738aa598ede55&nocache=62541e24b6e240f187ac920cab60f9da&dir=&wwwpkgdb=fc2733d6468c073f1dd738aa598ede55) | _ | lysophospholipase |
| [OCAR_4947](https://www.genoscope.cns.fr/agc/mage/wwwpkgdb/Info/getInfoLabel.php?id=3484496&wwwpkgdb=fc2733d6468c073f1dd738aa598ede55&nocache=62541e24b6e240f187ac920cab60f9da&dir=&wwwpkgdb=fc2733d6468c073f1dd738aa598ede55) | _ | inositol monophosphatase family protein |
| [OCAR_4948](https://www.genoscope.cns.fr/agc/mage/wwwpkgdb/Info/getInfoLabel.php?id=3484497&wwwpkgdb=fc2733d6468c073f1dd738aa598ede55&nocache=62541e24b6e240f187ac920cab60f9da&dir=&wwwpkgdb=fc2733d6468c073f1dd738aa598ede55) | _ | N-formylglutamate amidohydrolase |
| [OCAR_4950](https://www.genoscope.cns.fr/agc/mage/wwwpkgdb/Info/getInfoLabel.php?id=3482638&wwwpkgdb=fc2733d6468c073f1dd738aa598ede55&nocache=62541e24b6e240f187ac920cab60f9da&dir=&wwwpkgdb=fc2733d6468c073f1dd738aa598ede55) | _ | response regulator receiver domain protein |
| [OCAR_4978](https://www.genoscope.cns.fr/agc/mage/wwwpkgdb/Info/getInfoLabel.php?id=3482651&wwwpkgdb=fc2733d6468c073f1dd738aa598ede55&nocache=62541e24b6e240f187ac920cab60f9da&dir=&wwwpkgdb=fc2733d6468c073f1dd738aa598ede55) | _ | transposase IS3 |
| [OCAR_4979](https://www.genoscope.cns.fr/agc/mage/wwwpkgdb/Info/getInfoLabel.php?id=3482652&wwwpkgdb=fc2733d6468c073f1dd738aa598ede55&nocache=62541e24b6e240f187ac920cab60f9da&dir=&wwwpkgdb=fc2733d6468c073f1dd738aa598ede55) | _ | integrase, catalytic region |
| [OCAR_5017](https://www.genoscope.cns.fr/agc/mage/wwwpkgdb/Info/getInfoLabel.php?id=3484525&wwwpkgdb=fc2733d6468c073f1dd738aa598ede55&nocache=62541e24b6e240f187ac920cab60f9da&dir=&wwwpkgdb=fc2733d6468c073f1dd738aa598ede55) | _ | histone family protein DNA-binding protein |
| [OCAR_5060](https://www.genoscope.cns.fr/agc/mage/wwwpkgdb/Info/getInfoLabel.php?id=3484545&wwwpkgdb=fc2733d6468c073f1dd738aa598ede55&nocache=62541e24b6e240f187ac920cab60f9da&dir=&wwwpkgdb=fc2733d6468c073f1dd738aa598ede55) | _ | hypothetical protein |
| [OCAR_5062](https://www.genoscope.cns.fr/agc/mage/wwwpkgdb/Info/getInfoLabel.php?id=3484547&wwwpkgdb=fc2733d6468c073f1dd738aa598ede55&nocache=62541e24b6e240f187ac920cab60f9da&dir=&wwwpkgdb=fc2733d6468c073f1dd738aa598ede55) | _ | amino acid regulated cytosolic protein |
| [OCAR_5069](https://www.genoscope.cns.fr/agc/mage/wwwpkgdb/Info/getInfoLabel.php?id=3484552&wwwpkgdb=fc2733d6468c073f1dd738aa598ede55&nocache=62541e24b6e240f187ac920cab60f9da&dir=&wwwpkgdb=fc2733d6468c073f1dd738aa598ede55) | gnd | 6-phosphogluconate dehydrogenase |
| [OCAR_5080](https://www.genoscope.cns.fr/agc/mage/wwwpkgdb/Info/getInfoLabel.php?id=3482707&wwwpkgdb=fc2733d6468c073f1dd738aa598ede55&nocache=62541e24b6e240f187ac920cab60f9da&dir=&wwwpkgdb=fc2733d6468c073f1dd738aa598ede55) | rpmJ | ribosomal protein L36 |
| [OCAR_5083](https://www.genoscope.cns.fr/agc/mage/wwwpkgdb/Info/getInfoLabel.php?id=3484558&wwwpkgdb=fc2733d6468c073f1dd738aa598ede55&nocache=62541e24b6e240f187ac920cab60f9da&dir=&wwwpkgdb=fc2733d6468c073f1dd738aa598ede55) | pyk | pyruvate kinase |
| [OCAR_5084](https://www.genoscope.cns.fr/agc/mage/wwwpkgdb/Info/getInfoLabel.php?id=3484559&wwwpkgdb=fc2733d6468c073f1dd738aa598ede55&nocache=62541e24b6e240f187ac920cab60f9da&dir=&wwwpkgdb=fc2733d6468c073f1dd738aa598ede55) | _ | hypothetical protein |
| [OCAR_5085](https://www.genoscope.cns.fr/agc/mage/wwwpkgdb/Info/getInfoLabel.php?id=3482710&wwwpkgdb=fc2733d6468c073f1dd738aa598ede55&nocache=62541e24b6e240f187ac920cab60f9da&dir=&wwwpkgdb=fc2733d6468c073f1dd738aa598ede55) | _ | deoxycytidine triphosphate deaminase |
| [OCAR_5086](https://www.genoscope.cns.fr/agc/mage/wwwpkgdb/Info/getInfoLabel.php?id=3482711&wwwpkgdb=fc2733d6468c073f1dd738aa598ede55&nocache=62541e24b6e240f187ac920cab60f9da&dir=&wwwpkgdb=fc2733d6468c073f1dd738aa598ede55) | _ | UPF0335 protein OCAR_5086 |
| [OCAR_5089](https://www.genoscope.cns.fr/agc/mage/wwwpkgdb/Info/getInfoLabel.php?id=3484562&wwwpkgdb=fc2733d6468c073f1dd738aa598ede55&nocache=62541e24b6e240f187ac920cab60f9da&dir=&wwwpkgdb=fc2733d6468c073f1dd738aa598ede55) | _ | transcriptional regulatory protein ZraR |
| [OCAR_5090](https://www.genoscope.cns.fr/agc/mage/wwwpkgdb/Info/getInfoLabel.php?id=3482712&wwwpkgdb=fc2733d6468c073f1dd738aa598ede55&nocache=62541e24b6e240f187ac920cab60f9da&dir=&wwwpkgdb=fc2733d6468c073f1dd738aa598ede55) | _ | oligoendopeptidase, PepF/M3 family |
| [OCAR_5094](https://www.genoscope.cns.fr/agc/mage/wwwpkgdb/Info/getInfoLabel.php?id=3482716&wwwpkgdb=fc2733d6468c073f1dd738aa598ede55&nocache=62541e24b6e240f187ac920cab60f9da&dir=&wwwpkgdb=fc2733d6468c073f1dd738aa598ede55) | _ | NAD(P) transhydrogenase subunit alpha |
| [OCAR_5095](https://www.genoscope.cns.fr/agc/mage/wwwpkgdb/Info/getInfoLabel.php?id=3482717&wwwpkgdb=fc2733d6468c073f1dd738aa598ede55&nocache=62541e24b6e240f187ac920cab60f9da&dir=&wwwpkgdb=fc2733d6468c073f1dd738aa598ede55) | _ | NAD(P) transhydrogenase subunit alpha |
| [OCAR_5096](https://www.genoscope.cns.fr/agc/mage/wwwpkgdb/Info/getInfoLabel.php?id=3482718&wwwpkgdb=fc2733d6468c073f1dd738aa598ede55&nocache=62541e24b6e240f187ac920cab60f9da&dir=&wwwpkgdb=fc2733d6468c073f1dd738aa598ede55) | _ | NAD(p) transhydrogenase subunit beta |
| [OCAR_5101](https://www.genoscope.cns.fr/agc/mage/wwwpkgdb/Info/getInfoLabel.php?id=3484564&wwwpkgdb=fc2733d6468c073f1dd738aa598ede55&nocache=62541e24b6e240f187ac920cab60f9da&dir=&wwwpkgdb=fc2733d6468c073f1dd738aa598ede55) | rpsU | 30S ribosomal protein S21 |
| [OCAR_5102](https://www.genoscope.cns.fr/agc/mage/wwwpkgdb/Info/getInfoLabel.php?id=3482722&wwwpkgdb=fc2733d6468c073f1dd738aa598ede55&nocache=62541e24b6e240f187ac920cab60f9da&dir=&wwwpkgdb=fc2733d6468c073f1dd738aa598ede55) | _ | MIP family channel protein |
| [OCAR_5103](https://www.genoscope.cns.fr/agc/mage/wwwpkgdb/Info/getInfoLabel.php?id=3484565&wwwpkgdb=fc2733d6468c073f1dd738aa598ede55&nocache=62541e24b6e240f187ac920cab60f9da&dir=&wwwpkgdb=fc2733d6468c073f1dd738aa598ede55) | purK | phosphoribosylaminoimidazole carboxylase, ATPase subunit |
| [OCAR_5104](https://www.genoscope.cns.fr/agc/mage/wwwpkgdb/Info/getInfoLabel.php?id=3484566&wwwpkgdb=fc2733d6468c073f1dd738aa598ede55&nocache=62541e24b6e240f187ac920cab60f9da&dir=&wwwpkgdb=fc2733d6468c073f1dd738aa598ede55) | purE | phosphoribosylaminoimidazole carboxylase, catalytic subunit |
| [OCAR_5105](https://www.genoscope.cns.fr/agc/mage/wwwpkgdb/Info/getInfoLabel.php?id=3484567&wwwpkgdb=fc2733d6468c073f1dd738aa598ede55&nocache=62541e24b6e240f187ac920cab60f9da&dir=&wwwpkgdb=fc2733d6468c073f1dd738aa598ede55) | _ | hypothetical protein |
| [OCAR_5112](https://www.genoscope.cns.fr/agc/mage/wwwpkgdb/Info/getInfoLabel.php?id=3482727&wwwpkgdb=fc2733d6468c073f1dd738aa598ede55&nocache=62541e24b6e240f187ac920cab60f9da&dir=&wwwpkgdb=fc2733d6468c073f1dd738aa598ede55) | _ | putrescine transport system permease protein PotH |
| [OCAR_5113](https://www.genoscope.cns.fr/agc/mage/wwwpkgdb/Info/getInfoLabel.php?id=3482728&wwwpkgdb=fc2733d6468c073f1dd738aa598ede55&nocache=62541e24b6e240f187ac920cab60f9da&dir=&wwwpkgdb=fc2733d6468c073f1dd738aa598ede55) | _ | putrescine transport system permease protein PotI |
| [OCAR_5116](https://www.genoscope.cns.fr/agc/mage/wwwpkgdb/Info/getInfoLabel.php?id=3482730&wwwpkgdb=fc2733d6468c073f1dd738aa598ede55&nocache=62541e24b6e240f187ac920cab60f9da&dir=&wwwpkgdb=fc2733d6468c073f1dd738aa598ede55) | _ | succinyl-coa:3-ketoacid-coenzyme a transferase subunit a |
| [OCAR_5117](https://www.genoscope.cns.fr/agc/mage/wwwpkgdb/Info/getInfoLabel.php?id=3482731&wwwpkgdb=fc2733d6468c073f1dd738aa598ede55&nocache=62541e24b6e240f187ac920cab60f9da&dir=&wwwpkgdb=fc2733d6468c073f1dd738aa598ede55) | _ | 3-oxoadipate CoA-succinyl transferase beta subunit |
| [OCAR_5124](https://www.genoscope.cns.fr/agc/mage/wwwpkgdb/Info/getInfoLabel.php?id=3484574&wwwpkgdb=fc2733d6468c073f1dd738aa598ede55&nocache=62541e24b6e240f187ac920cab60f9da&dir=&wwwpkgdb=fc2733d6468c073f1dd738aa598ede55) | _ | transcriptional regulator, LysR family |
| [OCAR_5125](https://www.genoscope.cns.fr/agc/mage/wwwpkgdb/Info/getInfoLabel.php?id=3484575&wwwpkgdb=fc2733d6468c073f1dd738aa598ede55&nocache=62541e24b6e240f187ac920cab60f9da&dir=&wwwpkgdb=fc2733d6468c073f1dd738aa598ede55) | trxB | thioredoxin-disulfide reductase |
| [OCAR_5126](https://www.genoscope.cns.fr/agc/mage/wwwpkgdb/Info/getInfoLabel.php?id=3482735&wwwpkgdb=fc2733d6468c073f1dd738aa598ede55&nocache=62541e24b6e240f187ac920cab60f9da&dir=&wwwpkgdb=fc2733d6468c073f1dd738aa598ede55) | _ | Bkd operon transcriptional regulator |
| [OCAR_5130](https://www.genoscope.cns.fr/agc/mage/wwwpkgdb/Info/getInfoLabel.php?id=3484578&wwwpkgdb=fc2733d6468c073f1dd738aa598ede55&nocache=62541e24b6e240f187ac920cab60f9da&dir=&wwwpkgdb=fc2733d6468c073f1dd738aa598ede55) | _ | transcription elongation factor GreA |
| [OCAR_5131](https://www.genoscope.cns.fr/agc/mage/wwwpkgdb/Info/getInfoLabel.php?id=3484579&wwwpkgdb=fc2733d6468c073f1dd738aa598ede55&nocache=62541e24b6e240f187ac920cab60f9da&dir=&wwwpkgdb=fc2733d6468c073f1dd738aa598ede55) | carB | carbamoyl-phosphate synthase, large subunit |
| [OCAR_5134](https://www.genoscope.cns.fr/agc/mage/wwwpkgdb/Info/getInfoLabel.php?id=3482737&wwwpkgdb=fc2733d6468c073f1dd738aa598ede55&nocache=62541e24b6e240f187ac920cab60f9da&dir=&wwwpkgdb=fc2733d6468c073f1dd738aa598ede55) | _ | DNA-binding stress response protein Dps family |
| [OCAR_5135](https://www.genoscope.cns.fr/agc/mage/wwwpkgdb/Info/getInfoLabel.php?id=3484582&wwwpkgdb=fc2733d6468c073f1dd738aa598ede55&nocache=62541e24b6e240f187ac920cab60f9da&dir=&wwwpkgdb=fc2733d6468c073f1dd738aa598ede55) | carA | carbamoyl-phosphate synthase, small subunit |
| [OCAR_5136](https://www.genoscope.cns.fr/agc/mage/wwwpkgdb/Info/getInfoLabel.php?id=3482738&wwwpkgdb=fc2733d6468c073f1dd738aa598ede55&nocache=62541e24b6e240f187ac920cab60f9da&dir=&wwwpkgdb=fc2733d6468c073f1dd738aa598ede55) | _ | GatB/Yqey domain protein |
| [OCAR_5141](https://www.genoscope.cns.fr/agc/mage/wwwpkgdb/Info/getInfoLabel.php?id=3482740&wwwpkgdb=fc2733d6468c073f1dd738aa598ede55&nocache=62541e24b6e240f187ac920cab60f9da&dir=&wwwpkgdb=fc2733d6468c073f1dd738aa598ede55) | rpoD | RNA polymerase sigma factor RpoD |
| [OCAR_5144](https://www.genoscope.cns.fr/agc/mage/wwwpkgdb/Info/getInfoLabel.php?id=3482742&wwwpkgdb=fc2733d6468c073f1dd738aa598ede55&nocache=62541e24b6e240f187ac920cab60f9da&dir=&wwwpkgdb=fc2733d6468c073f1dd738aa598ede55) | _ | lytic murein transglycosylase |
| [OCAR_5146](https://www.genoscope.cns.fr/agc/mage/wwwpkgdb/Info/getInfoLabel.php?id=3484587&wwwpkgdb=fc2733d6468c073f1dd738aa598ede55&nocache=62541e24b6e240f187ac920cab60f9da&dir=&wwwpkgdb=fc2733d6468c073f1dd738aa598ede55) | _ | hypothetical protein |
| [OCAR_5147](https://www.genoscope.cns.fr/agc/mage/wwwpkgdb/Info/getInfoLabel.php?id=3484588&wwwpkgdb=fc2733d6468c073f1dd738aa598ede55&nocache=62541e24b6e240f187ac920cab60f9da&dir=&wwwpkgdb=fc2733d6468c073f1dd738aa598ede55) | msrB | methionine-R-sulfoxide reductase |
| [OCAR_5150](https://www.genoscope.cns.fr/agc/mage/wwwpkgdb/Info/getInfoLabel.php?id=3484590&wwwpkgdb=fc2733d6468c073f1dd738aa598ede55&nocache=62541e24b6e240f187ac920cab60f9da&dir=&wwwpkgdb=fc2733d6468c073f1dd738aa598ede55) | _ | exodeoxyribonuclease V |
| [OCAR_5169](https://www.genoscope.cns.fr/agc/mage/wwwpkgdb/Info/getInfoLabel.php?id=3484595&wwwpkgdb=fc2733d6468c073f1dd738aa598ede55&nocache=62541e24b6e240f187ac920cab60f9da&dir=&wwwpkgdb=fc2733d6468c073f1dd738aa598ede55) | _ | metallophosphoesterase |
| [OCAR_5170](https://www.genoscope.cns.fr/agc/mage/wwwpkgdb/Info/getInfoLabel.php?id=3484596&wwwpkgdb=fc2733d6468c073f1dd738aa598ede55&nocache=62541e24b6e240f187ac920cab60f9da&dir=&wwwpkgdb=fc2733d6468c073f1dd738aa598ede55) | _ | dead/deah box helicase |
| [OCAR_5171](https://www.genoscope.cns.fr/agc/mage/wwwpkgdb/Info/getInfoLabel.php?id=3482759&wwwpkgdb=fc2733d6468c073f1dd738aa598ede55&nocache=62541e24b6e240f187ac920cab60f9da&dir=&wwwpkgdb=fc2733d6468c073f1dd738aa598ede55) | _ | mRNA 3'-end processing factor |
| [OCAR_5172](https://www.genoscope.cns.fr/agc/mage/wwwpkgdb/Info/getInfoLabel.php?id=3482760&wwwpkgdb=fc2733d6468c073f1dd738aa598ede55&nocache=62541e24b6e240f187ac920cab60f9da&dir=&wwwpkgdb=fc2733d6468c073f1dd738aa598ede55) | _ | DNA ligase |
| [OCAR_5176](https://www.genoscope.cns.fr/agc/mage/wwwpkgdb/Info/getInfoLabel.php?id=3482763&wwwpkgdb=fc2733d6468c073f1dd738aa598ede55&nocache=62541e24b6e240f187ac920cab60f9da&dir=&wwwpkgdb=fc2733d6468c073f1dd738aa598ede55) | _ | error-prone DNA polymerase |
| [OCAR_5184](https://www.genoscope.cns.fr/agc/mage/wwwpkgdb/Info/getInfoLabel.php?id=3482768&wwwpkgdb=fc2733d6468c073f1dd738aa598ede55&nocache=62541e24b6e240f187ac920cab60f9da&dir=&wwwpkgdb=fc2733d6468c073f1dd738aa598ede55) | _ | sulfur oxidation protein |
| [OCAR_5206](https://www.genoscope.cns.fr/agc/mage/wwwpkgdb/Info/getInfoLabel.php?id=3484611&wwwpkgdb=fc2733d6468c073f1dd738aa598ede55&nocache=62541e24b6e240f187ac920cab60f9da&dir=&wwwpkgdb=fc2733d6468c073f1dd738aa598ede55) | _ | phosphatidylethanolamine N-methyltransferase |
| [OCAR_5207](https://www.genoscope.cns.fr/agc/mage/wwwpkgdb/Info/getInfoLabel.php?id=3484612&wwwpkgdb=fc2733d6468c073f1dd738aa598ede55&nocache=62541e24b6e240f187ac920cab60f9da&dir=&wwwpkgdb=fc2733d6468c073f1dd738aa598ede55) | mnmA | tRNA-specific 2-thiouridylase mnmA |
| [OCAR_5210](https://www.genoscope.cns.fr/agc/mage/wwwpkgdb/Info/getInfoLabel.php?id=3482782&wwwpkgdb=fc2733d6468c073f1dd738aa598ede55&nocache=62541e24b6e240f187ac920cab60f9da&dir=&wwwpkgdb=fc2733d6468c073f1dd738aa598ede55) | _ | flag1 |
| [OCAR_5212](https://www.genoscope.cns.fr/agc/mage/wwwpkgdb/Info/getInfoLabel.php?id=3482783&wwwpkgdb=fc2733d6468c073f1dd738aa598ede55&nocache=62541e24b6e240f187ac920cab60f9da&dir=&wwwpkgdb=fc2733d6468c073f1dd738aa598ede55) | fliF | flagellar M-ring protein FliF |
| [OCAR_5213](https://www.genoscope.cns.fr/agc/mage/wwwpkgdb/Info/getInfoLabel.php?id=3482784&wwwpkgdb=fc2733d6468c073f1dd738aa598ede55&nocache=62541e24b6e240f187ac920cab60f9da&dir=&wwwpkgdb=fc2733d6468c073f1dd738aa598ede55) | fliG | flagellar motor switch protein FliG |
| [OCAR_5215](https://www.genoscope.cns.fr/agc/mage/wwwpkgdb/Info/getInfoLabel.php?id=3482786&wwwpkgdb=fc2733d6468c073f1dd738aa598ede55&nocache=62541e24b6e240f187ac920cab60f9da&dir=&wwwpkgdb=fc2733d6468c073f1dd738aa598ede55) | _ | flagellar motor switch phosphatase FliY |
| [OCAR_5216](https://www.genoscope.cns.fr/agc/mage/wwwpkgdb/Info/getInfoLabel.php?id=3482787&wwwpkgdb=fc2733d6468c073f1dd738aa598ede55&nocache=62541e24b6e240f187ac920cab60f9da&dir=&wwwpkgdb=fc2733d6468c073f1dd738aa598ede55) | _ | transcriptional regulatory protein FlbD |
| [OCAR_5221](https://www.genoscope.cns.fr/agc/mage/wwwpkgdb/Info/getInfoLabel.php?id=3482791&wwwpkgdb=fc2733d6468c073f1dd738aa598ede55&nocache=62541e24b6e240f187ac920cab60f9da&dir=&wwwpkgdb=fc2733d6468c073f1dd738aa598ede55) | _ | acyl-coenzyme a dehydrogenase (acdh) |
| [OCAR_5222](https://www.genoscope.cns.fr/agc/mage/wwwpkgdb/Info/getInfoLabel.php?id=3482792&wwwpkgdb=fc2733d6468c073f1dd738aa598ede55&nocache=62541e24b6e240f187ac920cab60f9da&dir=&wwwpkgdb=fc2733d6468c073f1dd738aa598ede55) | _ | 3-ketoacyl-CoA thiolase |
| [OCAR_5234](https://www.genoscope.cns.fr/agc/mage/wwwpkgdb/Info/getInfoLabel.php?id=3482798&wwwpkgdb=fc2733d6468c073f1dd738aa598ede55&nocache=62541e24b6e240f187ac920cab60f9da&dir=&wwwpkgdb=fc2733d6468c073f1dd738aa598ede55) | mraW | S-adenosyl-methyltransferase MraW |
| [OCAR_5236](https://www.genoscope.cns.fr/agc/mage/wwwpkgdb/Info/getInfoLabel.php?id=3482800&wwwpkgdb=fc2733d6468c073f1dd738aa598ede55&nocache=62541e24b6e240f187ac920cab60f9da&dir=&wwwpkgdb=fc2733d6468c073f1dd738aa598ede55) | _ | peptidoglycan glycosyltransferase |
| [OCAR_5237](https://www.genoscope.cns.fr/agc/mage/wwwpkgdb/Info/getInfoLabel.php?id=3482801&wwwpkgdb=fc2733d6468c073f1dd738aa598ede55&nocache=62541e24b6e240f187ac920cab60f9da&dir=&wwwpkgdb=fc2733d6468c073f1dd738aa598ede55) | _ | UDP-N-acetylmuramoylalanyl-D-glutamate--2, 6-diaminopimelate ligase |
| [OCAR_5239](https://www.genoscope.cns.fr/agc/mage/wwwpkgdb/Info/getInfoLabel.php?id=3482803&wwwpkgdb=fc2733d6468c073f1dd738aa598ede55&nocache=62541e24b6e240f187ac920cab60f9da&dir=&wwwpkgdb=fc2733d6468c073f1dd738aa598ede55) | mraY | Phospho-N-acetylmuramoyl-pentapeptide-transferase |
| [OCAR_5241](https://www.genoscope.cns.fr/agc/mage/wwwpkgdb/Info/getInfoLabel.php?id=3482804&wwwpkgdb=fc2733d6468c073f1dd738aa598ede55&nocache=62541e24b6e240f187ac920cab60f9da&dir=&wwwpkgdb=fc2733d6468c073f1dd738aa598ede55) | murD | UDP-N-acetylmuramoylalanine--D-glutamate ligase |
| [OCAR_5242](https://www.genoscope.cns.fr/agc/mage/wwwpkgdb/Info/getInfoLabel.php?id=3482805&wwwpkgdb=fc2733d6468c073f1dd738aa598ede55&nocache=62541e24b6e240f187ac920cab60f9da&dir=&wwwpkgdb=fc2733d6468c073f1dd738aa598ede55) | ftsW | cell division protein FtsW |
| [OCAR_5243](https://www.genoscope.cns.fr/agc/mage/wwwpkgdb/Info/getInfoLabel.php?id=3482806&wwwpkgdb=fc2733d6468c073f1dd738aa598ede55&nocache=62541e24b6e240f187ac920cab60f9da&dir=&wwwpkgdb=fc2733d6468c073f1dd738aa598ede55) | murG | undecaprenyldiphospho-muramoylpentapeptide beta-N-acetylglucosaminyltransferase |
| [OCAR_5244](https://www.genoscope.cns.fr/agc/mage/wwwpkgdb/Info/getInfoLabel.php?id=3482807&wwwpkgdb=fc2733d6468c073f1dd738aa598ede55&nocache=62541e24b6e240f187ac920cab60f9da&dir=&wwwpkgdb=fc2733d6468c073f1dd738aa598ede55) | murC | UDP-N-acetylmuramate--alanine ligase |
| [OCAR_5245](https://www.genoscope.cns.fr/agc/mage/wwwpkgdb/Info/getInfoLabel.php?id=3482808&wwwpkgdb=fc2733d6468c073f1dd738aa598ede55&nocache=62541e24b6e240f187ac920cab60f9da&dir=&wwwpkgdb=fc2733d6468c073f1dd738aa598ede55) | murB | UDP-N-acetylenolpyruvoylglucosamine reductase |
| [OCAR_5249](https://www.genoscope.cns.fr/agc/mage/wwwpkgdb/Info/getInfoLabel.php?id=3482811&wwwpkgdb=fc2733d6468c073f1dd738aa598ede55&nocache=62541e24b6e240f187ac920cab60f9da&dir=&wwwpkgdb=fc2733d6468c073f1dd738aa598ede55) | ftsA | cell division protein FtsA |
| [OCAR_5250](https://www.genoscope.cns.fr/agc/mage/wwwpkgdb/Info/getInfoLabel.php?id=3482812&wwwpkgdb=fc2733d6468c073f1dd738aa598ede55&nocache=62541e24b6e240f187ac920cab60f9da&dir=&wwwpkgdb=fc2733d6468c073f1dd738aa598ede55) | _ | cell division protein FtsZ |
| [OCAR_5251](https://www.genoscope.cns.fr/agc/mage/wwwpkgdb/Info/getInfoLabel.php?id=3482813&wwwpkgdb=fc2733d6468c073f1dd738aa598ede55&nocache=62541e24b6e240f187ac920cab60f9da&dir=&wwwpkgdb=fc2733d6468c073f1dd738aa598ede55) | lpxC | UDP-3-O-[3-hydroxymyristoyl] N-acetylglucosamine deacetylase |
| [OCAR_5252](https://www.genoscope.cns.fr/agc/mage/wwwpkgdb/Info/getInfoLabel.php?id=3482814&wwwpkgdb=fc2733d6468c073f1dd738aa598ede55&nocache=62541e24b6e240f187ac920cab60f9da&dir=&wwwpkgdb=fc2733d6468c073f1dd738aa598ede55) | _ | coml, competence lipoprotein |
| [OCAR_5253](https://www.genoscope.cns.fr/agc/mage/wwwpkgdb/Info/getInfoLabel.php?id=3482815&wwwpkgdb=fc2733d6468c073f1dd738aa598ede55&nocache=62541e24b6e240f187ac920cab60f9da&dir=&wwwpkgdb=fc2733d6468c073f1dd738aa598ede55) | recN | DNA repair protein RecN |
| [OCAR_5256](https://www.genoscope.cns.fr/agc/mage/wwwpkgdb/Info/getInfoLabel.php?id=3484623&wwwpkgdb=fc2733d6468c073f1dd738aa598ede55&nocache=62541e24b6e240f187ac920cab60f9da&dir=&wwwpkgdb=fc2733d6468c073f1dd738aa598ede55) | _ | Xaa-Pro aminopeptidase 1 |
| [OCAR_5259](https://www.genoscope.cns.fr/agc/mage/wwwpkgdb/Info/getInfoLabel.php?id=3484625&wwwpkgdb=fc2733d6468c073f1dd738aa598ede55&nocache=62541e24b6e240f187ac920cab60f9da&dir=&wwwpkgdb=fc2733d6468c073f1dd738aa598ede55) | _ | oxidoreductase C10orf33 like protein |
| [OCAR_5263](https://www.genoscope.cns.fr/agc/mage/wwwpkgdb/Info/getInfoLabel.php?id=3484627&wwwpkgdb=fc2733d6468c073f1dd738aa598ede55&nocache=62541e24b6e240f187ac920cab60f9da&dir=&wwwpkgdb=fc2733d6468c073f1dd738aa598ede55) | _ | ABC-2 |
| [OCAR_5264](https://www.genoscope.cns.fr/agc/mage/wwwpkgdb/Info/getInfoLabel.php?id=3484628&wwwpkgdb=fc2733d6468c073f1dd738aa598ede55&nocache=62541e24b6e240f187ac920cab60f9da&dir=&wwwpkgdb=fc2733d6468c073f1dd738aa598ede55) | _ | hypothetical ABC transporter ATP-binding protein YadG |
| [OCAR_5265](https://www.genoscope.cns.fr/agc/mage/wwwpkgdb/Info/getInfoLabel.php?id=3484629&wwwpkgdb=fc2733d6468c073f1dd738aa598ede55&nocache=62541e24b6e240f187ac920cab60f9da&dir=&wwwpkgdb=fc2733d6468c073f1dd738aa598ede55) | _ | DNA helicase II |
| [OCAR_5266](https://www.genoscope.cns.fr/agc/mage/wwwpkgdb/Info/getInfoLabel.php?id=3482821&wwwpkgdb=fc2733d6468c073f1dd738aa598ede55&nocache=62541e24b6e240f187ac920cab60f9da&dir=&wwwpkgdb=fc2733d6468c073f1dd738aa598ede55) | _ | UPF0391 membrane protein OCAR_5266 |
| [OCAR_5272](https://www.genoscope.cns.fr/agc/mage/wwwpkgdb/Info/getInfoLabel.php?id=3484633&wwwpkgdb=fc2733d6468c073f1dd738aa598ede55&nocache=62541e24b6e240f187ac920cab60f9da&dir=&wwwpkgdb=fc2733d6468c073f1dd738aa598ede55) | _ | hypothetical protein |
| [OCAR_5274](https://www.genoscope.cns.fr/agc/mage/wwwpkgdb/Info/getInfoLabel.php?id=3484634&wwwpkgdb=fc2733d6468c073f1dd738aa598ede55&nocache=62541e24b6e240f187ac920cab60f9da&dir=&wwwpkgdb=fc2733d6468c073f1dd738aa598ede55) | fumC | fumarate hydratase, class II |
| [OCAR_5276](https://www.genoscope.cns.fr/agc/mage/wwwpkgdb/Info/getInfoLabel.php?id=3482825&wwwpkgdb=fc2733d6468c073f1dd738aa598ede55&nocache=62541e24b6e240f187ac920cab60f9da&dir=&wwwpkgdb=fc2733d6468c073f1dd738aa598ede55) | thyA | Thymidylate synthase |
| [OCAR_5278](https://www.genoscope.cns.fr/agc/mage/wwwpkgdb/Info/getInfoLabel.php?id=3482827&wwwpkgdb=fc2733d6468c073f1dd738aa598ede55&nocache=62541e24b6e240f187ac920cab60f9da&dir=&wwwpkgdb=fc2733d6468c073f1dd738aa598ede55) | hflK | HflK protein |
| [OCAR_5279](https://www.genoscope.cns.fr/agc/mage/wwwpkgdb/Info/getInfoLabel.php?id=3482828&wwwpkgdb=fc2733d6468c073f1dd738aa598ede55&nocache=62541e24b6e240f187ac920cab60f9da&dir=&wwwpkgdb=fc2733d6468c073f1dd738aa598ede55) | hflC | HflC protein |
| [OCAR_5281](https://www.genoscope.cns.fr/agc/mage/wwwpkgdb/Info/getInfoLabel.php?id=3482830&wwwpkgdb=fc2733d6468c073f1dd738aa598ede55&nocache=62541e24b6e240f187ac920cab60f9da&dir=&wwwpkgdb=fc2733d6468c073f1dd738aa598ede55) | _ | protease Do subfamily |
| [OCAR_5282](https://www.genoscope.cns.fr/agc/mage/wwwpkgdb/Info/getInfoLabel.php?id=3484636&wwwpkgdb=fc2733d6468c073f1dd738aa598ede55&nocache=62541e24b6e240f187ac920cab60f9da&dir=&wwwpkgdb=fc2733d6468c073f1dd738aa598ede55) | serB | phosphoserine phosphatase SerB |
| [OCAR_5283](https://www.genoscope.cns.fr/agc/mage/wwwpkgdb/Info/getInfoLabel.php?id=3482831&wwwpkgdb=fc2733d6468c073f1dd738aa598ede55&nocache=62541e24b6e240f187ac920cab60f9da&dir=&wwwpkgdb=fc2733d6468c073f1dd738aa598ede55) | miaA | tRNA Delta(2)-isopentenylpyrophosphate transferase |
| [OCAR_5290](https://www.genoscope.cns.fr/agc/mage/wwwpkgdb/Info/getInfoLabel.php?id=3482833&wwwpkgdb=fc2733d6468c073f1dd738aa598ede55&nocache=62541e24b6e240f187ac920cab60f9da&dir=&wwwpkgdb=fc2733d6468c073f1dd738aa598ede55) | ilvB | large subunit of acetolactate synthase |
| [OCAR_5291](https://www.genoscope.cns.fr/agc/mage/wwwpkgdb/Info/getInfoLabel.php?id=3482834&wwwpkgdb=fc2733d6468c073f1dd738aa598ede55&nocache=62541e24b6e240f187ac920cab60f9da&dir=&wwwpkgdb=fc2733d6468c073f1dd738aa598ede55) | ilvN | small subunit of acetolactate synthase |
| [OCAR_5292](https://www.genoscope.cns.fr/agc/mage/wwwpkgdb/Info/getInfoLabel.php?id=3482835&wwwpkgdb=fc2733d6468c073f1dd738aa598ede55&nocache=62541e24b6e240f187ac920cab60f9da&dir=&wwwpkgdb=fc2733d6468c073f1dd738aa598ede55) | ilvC | Ketol-acid reductoisomerase |
| [OCAR_5296](https://www.genoscope.cns.fr/agc/mage/wwwpkgdb/Info/getInfoLabel.php?id=3482838&wwwpkgdb=fc2733d6468c073f1dd738aa598ede55&nocache=62541e24b6e240f187ac920cab60f9da&dir=&wwwpkgdb=fc2733d6468c073f1dd738aa598ede55) | leuA | 2-isopropylmalate synthase |
| [OCAR_5313](https://www.genoscope.cns.fr/agc/mage/wwwpkgdb/Info/getInfoLabel.php?id=3482853&wwwpkgdb=fc2733d6468c073f1dd738aa598ede55&nocache=62541e24b6e240f187ac920cab60f9da&dir=&wwwpkgdb=fc2733d6468c073f1dd738aa598ede55) | ppc | phosphoenolpyruvate carboxylase |
| [OCAR_5318](https://www.genoscope.cns.fr/agc/mage/wwwpkgdb/Info/getInfoLabel.php?id=3482857&wwwpkgdb=fc2733d6468c073f1dd738aa598ede55&nocache=62541e24b6e240f187ac920cab60f9da&dir=&wwwpkgdb=fc2733d6468c073f1dd738aa598ede55) | _ | hypothetical protein |
| [OCAR_5319](https://www.genoscope.cns.fr/agc/mage/wwwpkgdb/Info/getInfoLabel.php?id=3482858&wwwpkgdb=fc2733d6468c073f1dd738aa598ede55&nocache=62541e24b6e240f187ac920cab60f9da&dir=&wwwpkgdb=fc2733d6468c073f1dd738aa598ede55) | metK | methionine adenosyltransferase |
| [OCAR_5321](https://www.genoscope.cns.fr/agc/mage/wwwpkgdb/Info/getInfoLabel.php?id=3482859&wwwpkgdb=fc2733d6468c073f1dd738aa598ede55&nocache=62541e24b6e240f187ac920cab60f9da&dir=&wwwpkgdb=fc2733d6468c073f1dd738aa598ede55) | ahcY | adenosylhomocysteinase |
| [OCAR_5322](https://www.genoscope.cns.fr/agc/mage/wwwpkgdb/Info/getInfoLabel.php?id=3482860&wwwpkgdb=fc2733d6468c073f1dd738aa598ede55&nocache=62541e24b6e240f187ac920cab60f9da&dir=&wwwpkgdb=fc2733d6468c073f1dd738aa598ede55) | _ | hypothetical protein |
| [OCAR_5328](https://www.genoscope.cns.fr/agc/mage/wwwpkgdb/Info/getInfoLabel.php?id=3484648&wwwpkgdb=fc2733d6468c073f1dd738aa598ede55&nocache=62541e24b6e240f187ac920cab60f9da&dir=&wwwpkgdb=fc2733d6468c073f1dd738aa598ede55) | _ | lipid A export ATP-binding/permease protein MsbA |
| [OCAR_5329](https://www.genoscope.cns.fr/agc/mage/wwwpkgdb/Info/getInfoLabel.php?id=3484649&wwwpkgdb=fc2733d6468c073f1dd738aa598ede55&nocache=62541e24b6e240f187ac920cab60f9da&dir=&wwwpkgdb=fc2733d6468c073f1dd738aa598ede55) | galE | UDP-glucose 4-epimerase |
| [OCAR_5334](https://www.genoscope.cns.fr/agc/mage/wwwpkgdb/Info/getInfoLabel.php?id=3482866&wwwpkgdb=fc2733d6468c073f1dd738aa598ede55&nocache=62541e24b6e240f187ac920cab60f9da&dir=&wwwpkgdb=fc2733d6468c073f1dd738aa598ede55) | rfaD | ADP-glyceromanno-heptose 6-epimerase |
| [OCAR_5338](https://www.genoscope.cns.fr/agc/mage/wwwpkgdb/Info/getInfoLabel.php?id=3482869&wwwpkgdb=fc2733d6468c073f1dd738aa598ede55&nocache=62541e24b6e240f187ac920cab60f9da&dir=&wwwpkgdb=fc2733d6468c073f1dd738aa598ede55) | _ | mannose-1-phosphate guanylyltransferase/mannose-6-phosphate isomerase |
| [OCAR_5339](https://www.genoscope.cns.fr/agc/mage/wwwpkgdb/Info/getInfoLabel.php?id=3482870&wwwpkgdb=fc2733d6468c073f1dd738aa598ede55&nocache=62541e24b6e240f187ac920cab60f9da&dir=&wwwpkgdb=fc2733d6468c073f1dd738aa598ede55) | _ | oxidoreductase domain protein |
| [OCAR_5340](https://www.genoscope.cns.fr/agc/mage/wwwpkgdb/Info/getInfoLabel.php?id=3482871&wwwpkgdb=fc2733d6468c073f1dd738aa598ede55&nocache=62541e24b6e240f187ac920cab60f9da&dir=&wwwpkgdb=fc2733d6468c073f1dd738aa598ede55) | _ | erythromycin biosynthesis sensory transduction protein eryC1 |
| [OCAR_5341](https://www.genoscope.cns.fr/agc/mage/wwwpkgdb/Info/getInfoLabel.php?id=3482872&wwwpkgdb=fc2733d6468c073f1dd738aa598ede55&nocache=62541e24b6e240f187ac920cab60f9da&dir=&wwwpkgdb=fc2733d6468c073f1dd738aa598ede55) | mviN | integral membrane protein MviN |
| [OCAR_5345](https://www.genoscope.cns.fr/agc/mage/wwwpkgdb/Info/getInfoLabel.php?id=3482875&wwwpkgdb=fc2733d6468c073f1dd738aa598ede55&nocache=62541e24b6e240f187ac920cab60f9da&dir=&wwwpkgdb=fc2733d6468c073f1dd738aa598ede55) | _ | metallophosphoesterase |
| [OCAR_5346](https://www.genoscope.cns.fr/agc/mage/wwwpkgdb/Info/getInfoLabel.php?id=3482876&wwwpkgdb=fc2733d6468c073f1dd738aa598ede55&nocache=62541e24b6e240f187ac920cab60f9da&dir=&wwwpkgdb=fc2733d6468c073f1dd738aa598ede55) | _ | glycosyl transferase, group 1 |
| [OCAR_5352](https://www.genoscope.cns.fr/agc/mage/wwwpkgdb/Info/getInfoLabel.php?id=3482880&wwwpkgdb=fc2733d6468c073f1dd738aa598ede55&nocache=62541e24b6e240f187ac920cab60f9da&dir=&wwwpkgdb=fc2733d6468c073f1dd738aa598ede55) | _ | ABC transporter, ATP-binding protein |
| [OCAR_5357](https://www.genoscope.cns.fr/agc/mage/wwwpkgdb/Info/getInfoLabel.php?id=3482884&wwwpkgdb=fc2733d6468c073f1dd738aa598ede55&nocache=62541e24b6e240f187ac920cab60f9da&dir=&wwwpkgdb=fc2733d6468c073f1dd738aa598ede55) | _ | 2Fe-2S ferredoxin (FdII) |
| [OCAR_5358](https://www.genoscope.cns.fr/agc/mage/wwwpkgdb/Info/getInfoLabel.php?id=3482885&wwwpkgdb=fc2733d6468c073f1dd738aa598ede55&nocache=62541e24b6e240f187ac920cab60f9da&dir=&wwwpkgdb=fc2733d6468c073f1dd738aa598ede55) | _ | FAD-dependent pyridine nucleotide-disulphide oxidoreductase |
| [OCAR_5372](https://www.genoscope.cns.fr/agc/mage/wwwpkgdb/Info/getInfoLabel.php?id=3484664&wwwpkgdb=fc2733d6468c073f1dd738aa598ede55&nocache=62541e24b6e240f187ac920cab60f9da&dir=&wwwpkgdb=fc2733d6468c073f1dd738aa598ede55) | _ | flagellar FlbT |
| [OCAR_5380](https://www.genoscope.cns.fr/agc/mage/wwwpkgdb/Info/getInfoLabel.php?id=3484668&wwwpkgdb=fc2733d6468c073f1dd738aa598ede55&nocache=62541e24b6e240f187ac920cab60f9da&dir=&wwwpkgdb=fc2733d6468c073f1dd738aa598ede55) | flgI | flagellar P-ring protein FlgI |
| [OCAR_5382](https://www.genoscope.cns.fr/agc/mage/wwwpkgdb/Info/getInfoLabel.php?id=3482898&wwwpkgdb=fc2733d6468c073f1dd738aa598ede55&nocache=62541e24b6e240f187ac920cab60f9da&dir=&wwwpkgdb=fc2733d6468c073f1dd738aa598ede55) | dksA | RNA polymerase-binding protein DksA |
| [OCAR_5384](https://www.genoscope.cns.fr/agc/mage/wwwpkgdb/Info/getInfoLabel.php?id=3484670&wwwpkgdb=fc2733d6468c073f1dd738aa598ede55&nocache=62541e24b6e240f187ac920cab60f9da&dir=&wwwpkgdb=fc2733d6468c073f1dd738aa598ede55) | flgH | Flagellar L-ring protein |
| [OCAR_5386](https://www.genoscope.cns.fr/agc/mage/wwwpkgdb/Info/getInfoLabel.php?id=3484672&wwwpkgdb=fc2733d6468c073f1dd738aa598ede55&nocache=62541e24b6e240f187ac920cab60f9da&dir=&wwwpkgdb=fc2733d6468c073f1dd738aa598ede55) | flgG | flagellar basal-body rod protein FlgG |
| [OCAR_5389](https://www.genoscope.cns.fr/agc/mage/wwwpkgdb/Info/getInfoLabel.php?id=3482900&wwwpkgdb=fc2733d6468c073f1dd738aa598ede55&nocache=62541e24b6e240f187ac920cab60f9da&dir=&wwwpkgdb=fc2733d6468c073f1dd738aa598ede55) | fliM | flagellar motor switch protein FliM |
| [OCAR_5393](https://www.genoscope.cns.fr/agc/mage/wwwpkgdb/Info/getInfoLabel.php?id=3484674&wwwpkgdb=fc2733d6468c073f1dd738aa598ede55&nocache=62541e24b6e240f187ac920cab60f9da&dir=&wwwpkgdb=fc2733d6468c073f1dd738aa598ede55) | fliP | flagellar biosynthetic protein FliP |
| [OCAR_5396](https://www.genoscope.cns.fr/agc/mage/wwwpkgdb/Info/getInfoLabel.php?id=3482905&wwwpkgdb=fc2733d6468c073f1dd738aa598ede55&nocache=62541e24b6e240f187ac920cab60f9da&dir=&wwwpkgdb=fc2733d6468c073f1dd738aa598ede55) | flgC | flagellar basal-body rod protein FlgC |
| [OCAR_5398](https://www.genoscope.cns.fr/agc/mage/wwwpkgdb/Info/getInfoLabel.php?id=3482907&wwwpkgdb=fc2733d6468c073f1dd738aa598ede55&nocache=62541e24b6e240f187ac920cab60f9da&dir=&wwwpkgdb=fc2733d6468c073f1dd738aa598ede55) | fliQ | flagellar biosynthetic protein FliQ |
| [OCAR_5399](https://www.genoscope.cns.fr/agc/mage/wwwpkgdb/Info/getInfoLabel.php?id=3482908&wwwpkgdb=fc2733d6468c073f1dd738aa598ede55&nocache=62541e24b6e240f187ac920cab60f9da&dir=&wwwpkgdb=fc2733d6468c073f1dd738aa598ede55) | fliR | flagellar biosynthetic protein FliR |
| [OCAR_5401](https://www.genoscope.cns.fr/agc/mage/wwwpkgdb/Info/getInfoLabel.php?id=3482910&wwwpkgdb=fc2733d6468c073f1dd738aa598ede55&nocache=62541e24b6e240f187ac920cab60f9da&dir=&wwwpkgdb=fc2733d6468c073f1dd738aa598ede55) | _ | multi-sensor hybrid histidine kinase |
| [OCAR_5431](https://www.genoscope.cns.fr/agc/mage/wwwpkgdb/Info/getInfoLabel.php?id=3482930&wwwpkgdb=fc2733d6468c073f1dd738aa598ede55&nocache=62541e24b6e240f187ac920cab60f9da&dir=&wwwpkgdb=fc2733d6468c073f1dd738aa598ede55) | recA | protein RecA |
| [OCAR_5432](https://www.genoscope.cns.fr/agc/mage/wwwpkgdb/Info/getInfoLabel.php?id=3484686&wwwpkgdb=fc2733d6468c073f1dd738aa598ede55&nocache=62541e24b6e240f187ac920cab60f9da&dir=&wwwpkgdb=fc2733d6468c073f1dd738aa598ede55) | gcvP | glycine dehydrogenase |
| [OCAR_5433](https://www.genoscope.cns.fr/agc/mage/wwwpkgdb/Info/getInfoLabel.php?id=3484687&wwwpkgdb=fc2733d6468c073f1dd738aa598ede55&nocache=62541e24b6e240f187ac920cab60f9da&dir=&wwwpkgdb=fc2733d6468c073f1dd738aa598ede55) | gcvH | glycine cleavage system H protein |
| [OCAR_5434](https://www.genoscope.cns.fr/agc/mage/wwwpkgdb/Info/getInfoLabel.php?id=3484688&wwwpkgdb=fc2733d6468c073f1dd738aa598ede55&nocache=62541e24b6e240f187ac920cab60f9da&dir=&wwwpkgdb=fc2733d6468c073f1dd738aa598ede55) | gcvT | glycine cleavage system T protein |
| [OCAR_5435](https://www.genoscope.cns.fr/agc/mage/wwwpkgdb/Info/getInfoLabel.php?id=3482931&wwwpkgdb=fc2733d6468c073f1dd738aa598ede55&nocache=62541e24b6e240f187ac920cab60f9da&dir=&wwwpkgdb=fc2733d6468c073f1dd738aa598ede55) | alaS | alanyl-tRNA synthetase |
| [OCAR_5440](https://www.genoscope.cns.fr/agc/mage/wwwpkgdb/Info/getInfoLabel.php?id=3484691&wwwpkgdb=fc2733d6468c073f1dd738aa598ede55&nocache=62541e24b6e240f187ac920cab60f9da&dir=&wwwpkgdb=fc2733d6468c073f1dd738aa598ede55) | _ | isocitrate dehydrogenase, NADP-dependent |
| [OCAR_5443](https://www.genoscope.cns.fr/agc/mage/wwwpkgdb/Info/getInfoLabel.php?id=3482935&wwwpkgdb=fc2733d6468c073f1dd738aa598ede55&nocache=62541e24b6e240f187ac920cab60f9da&dir=&wwwpkgdb=fc2733d6468c073f1dd738aa598ede55) | _ | RNA methyltransferase TrmH, group 1 |
| [OCAR_5447](https://www.genoscope.cns.fr/agc/mage/wwwpkgdb/Info/getInfoLabel.php?id=3484695&wwwpkgdb=fc2733d6468c073f1dd738aa598ede55&nocache=62541e24b6e240f187ac920cab60f9da&dir=&wwwpkgdb=fc2733d6468c073f1dd738aa598ede55) | _ | HpcH/HpaI aldolase |
| [OCAR_5449](https://www.genoscope.cns.fr/agc/mage/wwwpkgdb/Info/getInfoLabel.php?id=3482937&wwwpkgdb=fc2733d6468c073f1dd738aa598ede55&nocache=62541e24b6e240f187ac920cab60f9da&dir=&wwwpkgdb=fc2733d6468c073f1dd738aa598ede55) | purC | phosphoribosylaminoimidazole-succinocarboxamide synthase |
| [OCAR_5450](https://www.genoscope.cns.fr/agc/mage/wwwpkgdb/Info/getInfoLabel.php?id=3482938&wwwpkgdb=fc2733d6468c073f1dd738aa598ede55&nocache=62541e24b6e240f187ac920cab60f9da&dir=&wwwpkgdb=fc2733d6468c073f1dd738aa598ede55) | purS | phosphoribosylformylglycinamidine synthase, PurS protein |
| [OCAR_5451](https://www.genoscope.cns.fr/agc/mage/wwwpkgdb/Info/getInfoLabel.php?id=3482939&wwwpkgdb=fc2733d6468c073f1dd738aa598ede55&nocache=62541e24b6e240f187ac920cab60f9da&dir=&wwwpkgdb=fc2733d6468c073f1dd738aa598ede55) | purQ | phosphoribosylformylglycinamidine synthase I |
| [OCAR_5452](https://www.genoscope.cns.fr/agc/mage/wwwpkgdb/Info/getInfoLabel.php?id=3482940&wwwpkgdb=fc2733d6468c073f1dd738aa598ede55&nocache=62541e24b6e240f187ac920cab60f9da&dir=&wwwpkgdb=fc2733d6468c073f1dd738aa598ede55) | purL | phosphoribosylformylglycinamidine synthase II |
| [OCAR_5454](https://www.genoscope.cns.fr/agc/mage/wwwpkgdb/Info/getInfoLabel.php?id=3482941&wwwpkgdb=fc2733d6468c073f1dd738aa598ede55&nocache=62541e24b6e240f187ac920cab60f9da&dir=&wwwpkgdb=fc2733d6468c073f1dd738aa598ede55) | _ | BolA family protein |
| [OCAR_5456](https://www.genoscope.cns.fr/agc/mage/wwwpkgdb/Info/getInfoLabel.php?id=3484699&wwwpkgdb=fc2733d6468c073f1dd738aa598ede55&nocache=62541e24b6e240f187ac920cab60f9da&dir=&wwwpkgdb=fc2733d6468c073f1dd738aa598ede55) | _ | hypothetical protein |
| [OCAR_5457](https://www.genoscope.cns.fr/agc/mage/wwwpkgdb/Info/getInfoLabel.php?id=3482942&wwwpkgdb=fc2733d6468c073f1dd738aa598ede55&nocache=62541e24b6e240f187ac920cab60f9da&dir=&wwwpkgdb=fc2733d6468c073f1dd738aa598ede55) | _ | putative glutaredoxin like protein |
| [OCAR_5458](https://www.genoscope.cns.fr/agc/mage/wwwpkgdb/Info/getInfoLabel.php?id=3484700&wwwpkgdb=fc2733d6468c073f1dd738aa598ede55&nocache=62541e24b6e240f187ac920cab60f9da&dir=&wwwpkgdb=fc2733d6468c073f1dd738aa598ede55) | rpsD | 30S ribosomal protein S4 |
| [OCAR_5462](https://www.genoscope.cns.fr/agc/mage/wwwpkgdb/Info/getInfoLabel.php?id=3484703&wwwpkgdb=fc2733d6468c073f1dd738aa598ede55&nocache=62541e24b6e240f187ac920cab60f9da&dir=&wwwpkgdb=fc2733d6468c073f1dd738aa598ede55) | purB | adenylosuccinate lyase |
| [OCAR_5463](https://www.genoscope.cns.fr/agc/mage/wwwpkgdb/Info/getInfoLabel.php?id=3484704&wwwpkgdb=fc2733d6468c073f1dd738aa598ede55&nocache=62541e24b6e240f187ac920cab60f9da&dir=&wwwpkgdb=fc2733d6468c073f1dd738aa598ede55) | rpe | ribulose-phosphate 3-epimerase |
| [OCAR_5474](https://www.genoscope.cns.fr/agc/mage/wwwpkgdb/Info/getInfoLabel.php?id=3482946&wwwpkgdb=fc2733d6468c073f1dd738aa598ede55&nocache=62541e24b6e240f187ac920cab60f9da&dir=&wwwpkgdb=fc2733d6468c073f1dd738aa598ede55) | _ | C-type cytochrome biogenesis protein |
| [OCAR_5475](https://www.genoscope.cns.fr/agc/mage/wwwpkgdb/Info/getInfoLabel.php?id=3484713&wwwpkgdb=fc2733d6468c073f1dd738aa598ede55&nocache=62541e24b6e240f187ac920cab60f9da&dir=&wwwpkgdb=fc2733d6468c073f1dd738aa598ede55) | _ | Leu/Ile/Val-binding protein (LIV-BP) |
| [OCAR_5477](https://www.genoscope.cns.fr/agc/mage/wwwpkgdb/Info/getInfoLabel.php?id=3484715&wwwpkgdb=fc2733d6468c073f1dd738aa598ede55&nocache=62541e24b6e240f187ac920cab60f9da&dir=&wwwpkgdb=fc2733d6468c073f1dd738aa598ede55) | _ | high-affinity branched-chain amino acid transport ATP-binding proteinlivF (LIV-I protein F) |
| [OCAR_5478](https://www.genoscope.cns.fr/agc/mage/wwwpkgdb/Info/getInfoLabel.php?id=3484716&wwwpkgdb=fc2733d6468c073f1dd738aa598ede55&nocache=62541e24b6e240f187ac920cab60f9da&dir=&wwwpkgdb=fc2733d6468c073f1dd738aa598ede55) | _ | high-affinity branched-chain amino acid transport ATP-binding proteinlivG (LIV-I protein G) |
| [OCAR_5479](https://www.genoscope.cns.fr/agc/mage/wwwpkgdb/Info/getInfoLabel.php?id=3484717&wwwpkgdb=fc2733d6468c073f1dd738aa598ede55&nocache=62541e24b6e240f187ac920cab60f9da&dir=&wwwpkgdb=fc2733d6468c073f1dd738aa598ede55) | _ | high-affinity branched-chain amino acid transport system permeaseprotein LivM (LIV-I protein M) |
| [OCAR_5480](https://www.genoscope.cns.fr/agc/mage/wwwpkgdb/Info/getInfoLabel.php?id=3484718&wwwpkgdb=fc2733d6468c073f1dd738aa598ede55&nocache=62541e24b6e240f187ac920cab60f9da&dir=&wwwpkgdb=fc2733d6468c073f1dd738aa598ede55) | _ | high-affinity branched-chain amino acid transport system permeaseprotein LivH (LIV-I protein H) |
| [OCAR_5484](https://www.genoscope.cns.fr/agc/mage/wwwpkgdb/Info/getInfoLabel.php?id=3482949&wwwpkgdb=fc2733d6468c073f1dd738aa598ede55&nocache=62541e24b6e240f187ac920cab60f9da&dir=&wwwpkgdb=fc2733d6468c073f1dd738aa598ede55) | _ | XdhC/CoxI family protein |
| [OCAR_5490](https://www.genoscope.cns.fr/agc/mage/wwwpkgdb/Info/getInfoLabel.php?id=3484722&wwwpkgdb=fc2733d6468c073f1dd738aa598ede55&nocache=62541e24b6e240f187ac920cab60f9da&dir=&wwwpkgdb=fc2733d6468c073f1dd738aa598ede55) | _ | DUF72 |
| [OCAR_5491](https://www.genoscope.cns.fr/agc/mage/wwwpkgdb/Info/getInfoLabel.php?id=3482953&wwwpkgdb=fc2733d6468c073f1dd738aa598ede55&nocache=62541e24b6e240f187ac920cab60f9da&dir=&wwwpkgdb=fc2733d6468c073f1dd738aa598ede55) | _ | dolichyl-phosphate beta-D-mannosyltransferase |
| [OCAR_5502](https://www.genoscope.cns.fr/agc/mage/wwwpkgdb/Info/getInfoLabel.php?id=3484726&wwwpkgdb=fc2733d6468c073f1dd738aa598ede55&nocache=62541e24b6e240f187ac920cab60f9da&dir=&wwwpkgdb=fc2733d6468c073f1dd738aa598ede55) | groL | chaperonin GroL |
| [OCAR_5503](https://www.genoscope.cns.fr/agc/mage/wwwpkgdb/Info/getInfoLabel.php?id=3484727&wwwpkgdb=fc2733d6468c073f1dd738aa598ede55&nocache=62541e24b6e240f187ac920cab60f9da&dir=&wwwpkgdb=fc2733d6468c073f1dd738aa598ede55) | groS | chaperonin GroS |
| [OCAR_5504](https://www.genoscope.cns.fr/agc/mage/wwwpkgdb/Info/getInfoLabel.php?id=3482961&wwwpkgdb=fc2733d6468c073f1dd738aa598ede55&nocache=62541e24b6e240f187ac920cab60f9da&dir=&wwwpkgdb=fc2733d6468c073f1dd738aa598ede55) | _ | Usg family protein |
| [OCAR_5507](https://www.genoscope.cns.fr/agc/mage/wwwpkgdb/Info/getInfoLabel.php?id=3482962&wwwpkgdb=fc2733d6468c073f1dd738aa598ede55&nocache=62541e24b6e240f187ac920cab60f9da&dir=&wwwpkgdb=fc2733d6468c073f1dd738aa598ede55) | _ | competence-damage associated protein |
| [OCAR_5515](https://www.genoscope.cns.fr/agc/mage/wwwpkgdb/Info/getInfoLabel.php?id=3484731&wwwpkgdb=fc2733d6468c073f1dd738aa598ede55&nocache=62541e24b6e240f187ac920cab60f9da&dir=&wwwpkgdb=fc2733d6468c073f1dd738aa598ede55) | _ | hypothetical protein |
| [OCAR_5518](https://www.genoscope.cns.fr/agc/mage/wwwpkgdb/Info/getInfoLabel.php?id=3482971&wwwpkgdb=fc2733d6468c073f1dd738aa598ede55&nocache=62541e24b6e240f187ac920cab60f9da&dir=&wwwpkgdb=fc2733d6468c073f1dd738aa598ede55) | _ | serine acetyltransferase 4 |
| [OCAR_5519](https://www.genoscope.cns.fr/agc/mage/wwwpkgdb/Info/getInfoLabel.php?id=3482972&wwwpkgdb=fc2733d6468c073f1dd738aa598ede55&nocache=62541e24b6e240f187ac920cab60f9da&dir=&wwwpkgdb=fc2733d6468c073f1dd738aa598ede55) | _ | hypothetical protein |
| [OCAR_5529](https://www.genoscope.cns.fr/agc/mage/wwwpkgdb/Info/getInfoLabel.php?id=3484736&wwwpkgdb=fc2733d6468c073f1dd738aa598ede55&nocache=62541e24b6e240f187ac920cab60f9da&dir=&wwwpkgdb=fc2733d6468c073f1dd738aa598ede55) | _ | type IV pilus assembly PilZ |
| [OCAR_5539](https://www.genoscope.cns.fr/agc/mage/wwwpkgdb/Info/getInfoLabel.php?id=3484745&wwwpkgdb=fc2733d6468c073f1dd738aa598ede55&nocache=62541e24b6e240f187ac920cab60f9da&dir=&wwwpkgdb=fc2733d6468c073f1dd738aa598ede55) | _ | single-stranded DNA-binding protein (SSB) |
| [OCAR_5615](https://www.genoscope.cns.fr/agc/mage/wwwpkgdb/Info/getInfoLabel.php?id=3484761&wwwpkgdb=fc2733d6468c073f1dd738aa598ede55&nocache=62541e24b6e240f187ac920cab60f9da&dir=&wwwpkgdb=fc2733d6468c073f1dd738aa598ede55) | thrS | threonyl-tRNA synthetase |
| [OCAR_5619](https://www.genoscope.cns.fr/agc/mage/wwwpkgdb/Info/getInfoLabel.php?id=3484765&wwwpkgdb=fc2733d6468c073f1dd738aa598ede55&nocache=62541e24b6e240f187ac920cab60f9da&dir=&wwwpkgdb=fc2733d6468c073f1dd738aa598ede55) | _ | hypothetical protein |
| [OCAR_5622](https://www.genoscope.cns.fr/agc/mage/wwwpkgdb/Info/getInfoLabel.php?id=3484766&wwwpkgdb=fc2733d6468c073f1dd738aa598ede55&nocache=62541e24b6e240f187ac920cab60f9da&dir=&wwwpkgdb=fc2733d6468c073f1dd738aa598ede55) | _ | nitrogen-fixing NifU domain protein |
| [OCAR_5624](https://www.genoscope.cns.fr/agc/mage/wwwpkgdb/Info/getInfoLabel.php?id=3483041&wwwpkgdb=fc2733d6468c073f1dd738aa598ede55&nocache=62541e24b6e240f187ac920cab60f9da&dir=&wwwpkgdb=fc2733d6468c073f1dd738aa598ede55) | folE | GTP cyclohydrolase I |
| [OCAR_5625](https://www.genoscope.cns.fr/agc/mage/wwwpkgdb/Info/getInfoLabel.php?id=3483042&wwwpkgdb=fc2733d6468c073f1dd738aa598ede55&nocache=62541e24b6e240f187ac920cab60f9da&dir=&wwwpkgdb=fc2733d6468c073f1dd738aa598ede55) | _ | phosphoribosyl-AMP cyclohydrolase (PRA-CH) |
| [OCAR_5629](https://www.genoscope.cns.fr/agc/mage/wwwpkgdb/Info/getInfoLabel.php?id=3484769&wwwpkgdb=fc2733d6468c073f1dd738aa598ede55&nocache=62541e24b6e240f187ac920cab60f9da&dir=&wwwpkgdb=fc2733d6468c073f1dd738aa598ede55) | _ | two-component response regulator |
| [OCAR_5630](https://www.genoscope.cns.fr/agc/mage/wwwpkgdb/Info/getInfoLabel.php?id=3484770&wwwpkgdb=fc2733d6468c073f1dd738aa598ede55&nocache=62541e24b6e240f187ac920cab60f9da&dir=&wwwpkgdb=fc2733d6468c073f1dd738aa598ede55) | _ | ATP-NAD kinase |
| [OCAR_5640](https://www.genoscope.cns.fr/agc/mage/wwwpkgdb/Info/getInfoLabel.php?id=3484774&wwwpkgdb=fc2733d6468c073f1dd738aa598ede55&nocache=62541e24b6e240f187ac920cab60f9da&dir=&wwwpkgdb=fc2733d6468c073f1dd738aa598ede55) | _ | RNA methyltransferase, TrmH family, group 3 |
| [OCAR_5656](https://www.genoscope.cns.fr/agc/mage/wwwpkgdb/Info/getInfoLabel.php?id=3483060&wwwpkgdb=fc2733d6468c073f1dd738aa598ede55&nocache=62541e24b6e240f187ac920cab60f9da&dir=&wwwpkgdb=fc2733d6468c073f1dd738aa598ede55) | secE | preprotein translocase, SecE subunit |
| [OCAR_5657](https://www.genoscope.cns.fr/agc/mage/wwwpkgdb/Info/getInfoLabel.php?id=3483061&wwwpkgdb=fc2733d6468c073f1dd738aa598ede55&nocache=62541e24b6e240f187ac920cab60f9da&dir=&wwwpkgdb=fc2733d6468c073f1dd738aa598ede55) | nusG | transcription termination/antitermination factor NusG |
| [OCAR_5659](https://www.genoscope.cns.fr/agc/mage/wwwpkgdb/Info/getInfoLabel.php?id=3483062&wwwpkgdb=fc2733d6468c073f1dd738aa598ede55&nocache=62541e24b6e240f187ac920cab60f9da&dir=&wwwpkgdb=fc2733d6468c073f1dd738aa598ede55) | rplK | 50S ribosomal protein L11 |
| [OCAR_5660](https://www.genoscope.cns.fr/agc/mage/wwwpkgdb/Info/getInfoLabel.php?id=3483063&wwwpkgdb=fc2733d6468c073f1dd738aa598ede55&nocache=62541e24b6e240f187ac920cab60f9da&dir=&wwwpkgdb=fc2733d6468c073f1dd738aa598ede55) | rplA | 50S ribosomal protein L1 |
| [OCAR_5661](https://www.genoscope.cns.fr/agc/mage/wwwpkgdb/Info/getInfoLabel.php?id=3483064&wwwpkgdb=fc2733d6468c073f1dd738aa598ede55&nocache=62541e24b6e240f187ac920cab60f9da&dir=&wwwpkgdb=fc2733d6468c073f1dd738aa598ede55) | rplJ | 50S ribosomal protein L10 |
| [OCAR_5662](https://www.genoscope.cns.fr/agc/mage/wwwpkgdb/Info/getInfoLabel.php?id=3483065&wwwpkgdb=fc2733d6468c073f1dd738aa598ede55&nocache=62541e24b6e240f187ac920cab60f9da&dir=&wwwpkgdb=fc2733d6468c073f1dd738aa598ede55) | rplL | 50S ribosomal protein L7/L12 |
| [OCAR_5663](https://www.genoscope.cns.fr/agc/mage/wwwpkgdb/Info/getInfoLabel.php?id=3483066&wwwpkgdb=fc2733d6468c073f1dd738aa598ede55&nocache=62541e24b6e240f187ac920cab60f9da&dir=&wwwpkgdb=fc2733d6468c073f1dd738aa598ede55) | rpoB | DNA-directed RNA polymerase, beta subunit |
| [OCAR_5665](https://www.genoscope.cns.fr/agc/mage/wwwpkgdb/Info/getInfoLabel.php?id=3483067&wwwpkgdb=fc2733d6468c073f1dd738aa598ede55&nocache=62541e24b6e240f187ac920cab60f9da&dir=&wwwpkgdb=fc2733d6468c073f1dd738aa598ede55) | rpoC | DNA-directed RNA polymerase subunit beta' |
| [OCAR_5666](https://www.genoscope.cns.fr/agc/mage/wwwpkgdb/Info/getInfoLabel.php?id=3483068&wwwpkgdb=fc2733d6468c073f1dd738aa598ede55&nocache=62541e24b6e240f187ac920cab60f9da&dir=&wwwpkgdb=fc2733d6468c073f1dd738aa598ede55) | _ | amine oxidase |
| [OCAR_5667](https://www.genoscope.cns.fr/agc/mage/wwwpkgdb/Info/getInfoLabel.php?id=3483069&wwwpkgdb=fc2733d6468c073f1dd738aa598ede55&nocache=62541e24b6e240f187ac920cab60f9da&dir=&wwwpkgdb=fc2733d6468c073f1dd738aa598ede55) | _ | ABC transporter |
| [OCAR_5672](https://www.genoscope.cns.fr/agc/mage/wwwpkgdb/Info/getInfoLabel.php?id=3483072&wwwpkgdb=fc2733d6468c073f1dd738aa598ede55&nocache=62541e24b6e240f187ac920cab60f9da&dir=&wwwpkgdb=fc2733d6468c073f1dd738aa598ede55) | rpsL | 30S ribosomal protein S12 |
| [OCAR_5673](https://www.genoscope.cns.fr/agc/mage/wwwpkgdb/Info/getInfoLabel.php?id=3483073&wwwpkgdb=fc2733d6468c073f1dd738aa598ede55&nocache=62541e24b6e240f187ac920cab60f9da&dir=&wwwpkgdb=fc2733d6468c073f1dd738aa598ede55) | rpsG | 30S ribosomal protein S7 |
| [OCAR_5674](https://www.genoscope.cns.fr/agc/mage/wwwpkgdb/Info/getInfoLabel.php?id=3483074&wwwpkgdb=fc2733d6468c073f1dd738aa598ede55&nocache=62541e24b6e240f187ac920cab60f9da&dir=&wwwpkgdb=fc2733d6468c073f1dd738aa598ede55) | fusA | Elongation factor G |
| [OCAR_5675](https://www.genoscope.cns.fr/agc/mage/wwwpkgdb/Info/getInfoLabel.php?id=3483075&wwwpkgdb=fc2733d6468c073f1dd738aa598ede55&nocache=62541e24b6e240f187ac920cab60f9da&dir=&wwwpkgdb=fc2733d6468c073f1dd738aa598ede55) | tuf | Elongation factor Tu |
| [OCAR_5676](https://www.genoscope.cns.fr/agc/mage/wwwpkgdb/Info/getInfoLabel.php?id=3483076&wwwpkgdb=fc2733d6468c073f1dd738aa598ede55&nocache=62541e24b6e240f187ac920cab60f9da&dir=&wwwpkgdb=fc2733d6468c073f1dd738aa598ede55) | rpsJ | 30S ribosomal protein S10 |
| [OCAR_5677](https://www.genoscope.cns.fr/agc/mage/wwwpkgdb/Info/getInfoLabel.php?id=3483077&wwwpkgdb=fc2733d6468c073f1dd738aa598ede55&nocache=62541e24b6e240f187ac920cab60f9da&dir=&wwwpkgdb=fc2733d6468c073f1dd738aa598ede55) | rplC | 50S ribosomal protein L3 |
| [OCAR_5678](https://www.genoscope.cns.fr/agc/mage/wwwpkgdb/Info/getInfoLabel.php?id=3483078&wwwpkgdb=fc2733d6468c073f1dd738aa598ede55&nocache=62541e24b6e240f187ac920cab60f9da&dir=&wwwpkgdb=fc2733d6468c073f1dd738aa598ede55) | rplD | 50S ribosomal protein L4 |
| [OCAR_5679](https://www.genoscope.cns.fr/agc/mage/wwwpkgdb/Info/getInfoLabel.php?id=3483079&wwwpkgdb=fc2733d6468c073f1dd738aa598ede55&nocache=62541e24b6e240f187ac920cab60f9da&dir=&wwwpkgdb=fc2733d6468c073f1dd738aa598ede55) | rplW | ribosomal protein L23 |
| [OCAR_5680](https://www.genoscope.cns.fr/agc/mage/wwwpkgdb/Info/getInfoLabel.php?id=3483080&wwwpkgdb=fc2733d6468c073f1dd738aa598ede55&nocache=62541e24b6e240f187ac920cab60f9da&dir=&wwwpkgdb=fc2733d6468c073f1dd738aa598ede55) | rplB | 50S ribosomal protein L2 |
| [OCAR_5681](https://www.genoscope.cns.fr/agc/mage/wwwpkgdb/Info/getInfoLabel.php?id=3483081&wwwpkgdb=fc2733d6468c073f1dd738aa598ede55&nocache=62541e24b6e240f187ac920cab60f9da&dir=&wwwpkgdb=fc2733d6468c073f1dd738aa598ede55) | rpsS | 30S ribosomal protein S19 |
| [OCAR_5682](https://www.genoscope.cns.fr/agc/mage/wwwpkgdb/Info/getInfoLabel.php?id=3483082&wwwpkgdb=fc2733d6468c073f1dd738aa598ede55&nocache=62541e24b6e240f187ac920cab60f9da&dir=&wwwpkgdb=fc2733d6468c073f1dd738aa598ede55) | rplV | ribosomal protein L22 |
| [OCAR_5683](https://www.genoscope.cns.fr/agc/mage/wwwpkgdb/Info/getInfoLabel.php?id=3483083&wwwpkgdb=fc2733d6468c073f1dd738aa598ede55&nocache=62541e24b6e240f187ac920cab60f9da&dir=&wwwpkgdb=fc2733d6468c073f1dd738aa598ede55) | rpsC | 30S ribosomal protein S3 |
| [OCAR_5684](https://www.genoscope.cns.fr/agc/mage/wwwpkgdb/Info/getInfoLabel.php?id=3483084&wwwpkgdb=fc2733d6468c073f1dd738aa598ede55&nocache=62541e24b6e240f187ac920cab60f9da&dir=&wwwpkgdb=fc2733d6468c073f1dd738aa598ede55) | rplP | 50S ribosomal protein L16 |
| [OCAR_5685](https://www.genoscope.cns.fr/agc/mage/wwwpkgdb/Info/getInfoLabel.php?id=3483085&wwwpkgdb=fc2733d6468c073f1dd738aa598ede55&nocache=62541e24b6e240f187ac920cab60f9da&dir=&wwwpkgdb=fc2733d6468c073f1dd738aa598ede55) | rpmC | ribosomal protein L29 |
| [OCAR_5686](https://www.genoscope.cns.fr/agc/mage/wwwpkgdb/Info/getInfoLabel.php?id=3483086&wwwpkgdb=fc2733d6468c073f1dd738aa598ede55&nocache=62541e24b6e240f187ac920cab60f9da&dir=&wwwpkgdb=fc2733d6468c073f1dd738aa598ede55) | rpsQ | 30S ribosomal protein S17 |
| [OCAR_5687](https://www.genoscope.cns.fr/agc/mage/wwwpkgdb/Info/getInfoLabel.php?id=3483087&wwwpkgdb=fc2733d6468c073f1dd738aa598ede55&nocache=62541e24b6e240f187ac920cab60f9da&dir=&wwwpkgdb=fc2733d6468c073f1dd738aa598ede55) | rplN | 50S ribosomal protein L14 |
| [OCAR_5688](https://www.genoscope.cns.fr/agc/mage/wwwpkgdb/Info/getInfoLabel.php?id=3483088&wwwpkgdb=fc2733d6468c073f1dd738aa598ede55&nocache=62541e24b6e240f187ac920cab60f9da&dir=&wwwpkgdb=fc2733d6468c073f1dd738aa598ede55) | rplX | 50S ribosomal protein L24 |
| [OCAR_5689](https://www.genoscope.cns.fr/agc/mage/wwwpkgdb/Info/getInfoLabel.php?id=3483089&wwwpkgdb=fc2733d6468c073f1dd738aa598ede55&nocache=62541e24b6e240f187ac920cab60f9da&dir=&wwwpkgdb=fc2733d6468c073f1dd738aa598ede55) | rplE | 50S ribosomal protein L5 |
| [OCAR_5690](https://www.genoscope.cns.fr/agc/mage/wwwpkgdb/Info/getInfoLabel.php?id=3483090&wwwpkgdb=fc2733d6468c073f1dd738aa598ede55&nocache=62541e24b6e240f187ac920cab60f9da&dir=&wwwpkgdb=fc2733d6468c073f1dd738aa598ede55) | rpsN | 30S ribosomal protein S14 |
| [OCAR_5691](https://www.genoscope.cns.fr/agc/mage/wwwpkgdb/Info/getInfoLabel.php?id=3483091&wwwpkgdb=fc2733d6468c073f1dd738aa598ede55&nocache=62541e24b6e240f187ac920cab60f9da&dir=&wwwpkgdb=fc2733d6468c073f1dd738aa598ede55) | rpsH | 30S ribosomal protein S8 |
| [OCAR_5692](https://www.genoscope.cns.fr/agc/mage/wwwpkgdb/Info/getInfoLabel.php?id=3483092&wwwpkgdb=fc2733d6468c073f1dd738aa598ede55&nocache=62541e24b6e240f187ac920cab60f9da&dir=&wwwpkgdb=fc2733d6468c073f1dd738aa598ede55) | rplF | 50S ribosomal protein L6 |
| [OCAR_5693](https://www.genoscope.cns.fr/agc/mage/wwwpkgdb/Info/getInfoLabel.php?id=3483093&wwwpkgdb=fc2733d6468c073f1dd738aa598ede55&nocache=62541e24b6e240f187ac920cab60f9da&dir=&wwwpkgdb=fc2733d6468c073f1dd738aa598ede55) | rplR | 50S ribosomal protein L18 |
| [OCAR_5694](https://www.genoscope.cns.fr/agc/mage/wwwpkgdb/Info/getInfoLabel.php?id=3483094&wwwpkgdb=fc2733d6468c073f1dd738aa598ede55&nocache=62541e24b6e240f187ac920cab60f9da&dir=&wwwpkgdb=fc2733d6468c073f1dd738aa598ede55) | rpsE | 30S ribosomal protein S5 |
| [OCAR_5695](https://www.genoscope.cns.fr/agc/mage/wwwpkgdb/Info/getInfoLabel.php?id=3483095&wwwpkgdb=fc2733d6468c073f1dd738aa598ede55&nocache=62541e24b6e240f187ac920cab60f9da&dir=&wwwpkgdb=fc2733d6468c073f1dd738aa598ede55) | rpmD | ribosomal protein L30 |
| [OCAR_5696](https://www.genoscope.cns.fr/agc/mage/wwwpkgdb/Info/getInfoLabel.php?id=3483096&wwwpkgdb=fc2733d6468c073f1dd738aa598ede55&nocache=62541e24b6e240f187ac920cab60f9da&dir=&wwwpkgdb=fc2733d6468c073f1dd738aa598ede55) | rplO | 50S ribosomal protein L15 |
| [OCAR_5697](https://www.genoscope.cns.fr/agc/mage/wwwpkgdb/Info/getInfoLabel.php?id=3483097&wwwpkgdb=fc2733d6468c073f1dd738aa598ede55&nocache=62541e24b6e240f187ac920cab60f9da&dir=&wwwpkgdb=fc2733d6468c073f1dd738aa598ede55) | _ | preprotein translocase, SecY subunit |
| [OCAR_5699](https://www.genoscope.cns.fr/agc/mage/wwwpkgdb/Info/getInfoLabel.php?id=3483099&wwwpkgdb=fc2733d6468c073f1dd738aa598ede55&nocache=62541e24b6e240f187ac920cab60f9da&dir=&wwwpkgdb=fc2733d6468c073f1dd738aa598ede55) | rpsM | ribosomal protein S13p/S18e |
| [OCAR_5700](https://www.genoscope.cns.fr/agc/mage/wwwpkgdb/Info/getInfoLabel.php?id=3483100&wwwpkgdb=fc2733d6468c073f1dd738aa598ede55&nocache=62541e24b6e240f187ac920cab60f9da&dir=&wwwpkgdb=fc2733d6468c073f1dd738aa598ede55) | rpsK | ribosomal protein S11 |
| [OCAR_5701](https://www.genoscope.cns.fr/agc/mage/wwwpkgdb/Info/getInfoLabel.php?id=3483101&wwwpkgdb=fc2733d6468c073f1dd738aa598ede55&nocache=62541e24b6e240f187ac920cab60f9da&dir=&wwwpkgdb=fc2733d6468c073f1dd738aa598ede55) | rpoA | DNA-directed RNA polymerase subunit alpha |
| [OCAR_5702](https://www.genoscope.cns.fr/agc/mage/wwwpkgdb/Info/getInfoLabel.php?id=3483102&wwwpkgdb=fc2733d6468c073f1dd738aa598ede55&nocache=62541e24b6e240f187ac920cab60f9da&dir=&wwwpkgdb=fc2733d6468c073f1dd738aa598ede55) | rplQ | 50S ribosomal protein L17 |
| [OCAR_5704](https://www.genoscope.cns.fr/agc/mage/wwwpkgdb/Info/getInfoLabel.php?id=3483104&wwwpkgdb=fc2733d6468c073f1dd738aa598ede55&nocache=62541e24b6e240f187ac920cab60f9da&dir=&wwwpkgdb=fc2733d6468c073f1dd738aa598ede55) | _ | oxidoreductase, short chain dehydrogenase/reductase family |
| [OCAR_5708](https://www.genoscope.cns.fr/agc/mage/wwwpkgdb/Info/getInfoLabel.php?id=3483107&wwwpkgdb=fc2733d6468c073f1dd738aa598ede55&nocache=62541e24b6e240f187ac920cab60f9da&dir=&wwwpkgdb=fc2733d6468c073f1dd738aa598ede55) | _ | protease do |
| [OCAR_5709](https://www.genoscope.cns.fr/agc/mage/wwwpkgdb/Info/getInfoLabel.php?id=3483108&wwwpkgdb=fc2733d6468c073f1dd738aa598ede55&nocache=62541e24b6e240f187ac920cab60f9da&dir=&wwwpkgdb=fc2733d6468c073f1dd738aa598ede55) | _ | AAA ATPase, central region |
| [OCAR_5710](https://www.genoscope.cns.fr/agc/mage/wwwpkgdb/Info/getInfoLabel.php?id=3483109&wwwpkgdb=fc2733d6468c073f1dd738aa598ede55&nocache=62541e24b6e240f187ac920cab60f9da&dir=&wwwpkgdb=fc2733d6468c073f1dd738aa598ede55) | _ | pseudouridine synthase, RluD |
| [OCAR_5714](https://www.genoscope.cns.fr/agc/mage/wwwpkgdb/Info/getInfoLabel.php?id=3483113&wwwpkgdb=fc2733d6468c073f1dd738aa598ede55&nocache=62541e24b6e240f187ac920cab60f9da&dir=&wwwpkgdb=fc2733d6468c073f1dd738aa598ede55) | _ | flagellar biosynthetic protein FlhB |
| [OCAR_5733](https://www.genoscope.cns.fr/agc/mage/wwwpkgdb/Info/getInfoLabel.php?id=3484795&wwwpkgdb=fc2733d6468c073f1dd738aa598ede55&nocache=62541e24b6e240f187ac920cab60f9da&dir=&wwwpkgdb=fc2733d6468c073f1dd738aa598ede55) | _ | S-(hydroxymethyl)glutathione dehydrogenase/class III alcohol dehydrogenase |
| [OCAR_5736](https://www.genoscope.cns.fr/agc/mage/wwwpkgdb/Info/getInfoLabel.php?id=3484797&wwwpkgdb=fc2733d6468c073f1dd738aa598ede55&nocache=62541e24b6e240f187ac920cab60f9da&dir=&wwwpkgdb=fc2733d6468c073f1dd738aa598ede55) | _ | peroxiredoxin-6 |
| [OCAR_5741](https://www.genoscope.cns.fr/agc/mage/wwwpkgdb/Info/getInfoLabel.php?id=3484798&wwwpkgdb=fc2733d6468c073f1dd738aa598ede55&nocache=62541e24b6e240f187ac920cab60f9da&dir=&wwwpkgdb=fc2733d6468c073f1dd738aa598ede55) | lipB | Octanoyltransferase |
| [OCAR_5742](https://www.genoscope.cns.fr/agc/mage/wwwpkgdb/Info/getInfoLabel.php?id=3483129&wwwpkgdb=fc2733d6468c073f1dd738aa598ede55&nocache=62541e24b6e240f187ac920cab60f9da&dir=&wwwpkgdb=fc2733d6468c073f1dd738aa598ede55) | _ | surface presentation of antigens |
| [OCAR_5756](https://www.genoscope.cns.fr/agc/mage/wwwpkgdb/Info/getInfoLabel.php?id=3483137&wwwpkgdb=fc2733d6468c073f1dd738aa598ede55&nocache=62541e24b6e240f187ac920cab60f9da&dir=&wwwpkgdb=fc2733d6468c073f1dd738aa598ede55) | _ | putative exported protein of unknown function |
| [OCAR_5765](https://www.genoscope.cns.fr/agc/mage/wwwpkgdb/Info/getInfoLabel.php?id=3483143&wwwpkgdb=fc2733d6468c073f1dd738aa598ede55&nocache=62541e24b6e240f187ac920cab60f9da&dir=&wwwpkgdb=fc2733d6468c073f1dd738aa598ede55) | _ | ATP-dependent Clp protease adaptor protein ClpS |
| [OCAR_5767](https://www.genoscope.cns.fr/agc/mage/wwwpkgdb/Info/getInfoLabel.php?id=3483144&wwwpkgdb=fc2733d6468c073f1dd738aa598ede55&nocache=62541e24b6e240f187ac920cab60f9da&dir=&wwwpkgdb=fc2733d6468c073f1dd738aa598ede55) | clpA | ATP-dependent Clp protease ATP-binding subunit ClpA |
| [OCAR_5772](https://www.genoscope.cns.fr/agc/mage/wwwpkgdb/Info/getInfoLabel.php?id=3484812&wwwpkgdb=fc2733d6468c073f1dd738aa598ede55&nocache=62541e24b6e240f187ac920cab60f9da&dir=&wwwpkgdb=fc2733d6468c073f1dd738aa598ede55) | _ | endoribonuclease L-PSP |
| [OCAR_5774](https://www.genoscope.cns.fr/agc/mage/wwwpkgdb/Info/getInfoLabel.php?id=3484813&wwwpkgdb=fc2733d6468c073f1dd738aa598ede55&nocache=62541e24b6e240f187ac920cab60f9da&dir=&wwwpkgdb=fc2733d6468c073f1dd738aa598ede55) | _ | DNA polymerase IV (Pol IV) |
| [OCAR_5776](https://www.genoscope.cns.fr/agc/mage/wwwpkgdb/Info/getInfoLabel.php?id=3483147&wwwpkgdb=fc2733d6468c073f1dd738aa598ede55&nocache=62541e24b6e240f187ac920cab60f9da&dir=&wwwpkgdb=fc2733d6468c073f1dd738aa598ede55) | _ | cell division response regulator DivK |
| [OCAR_5777](https://www.genoscope.cns.fr/agc/mage/wwwpkgdb/Info/getInfoLabel.php?id=3483148&wwwpkgdb=fc2733d6468c073f1dd738aa598ede55&nocache=62541e24b6e240f187ac920cab60f9da&dir=&wwwpkgdb=fc2733d6468c073f1dd738aa598ede55) | _ | response regulator protein |
| [OCAR_5778](https://www.genoscope.cns.fr/agc/mage/wwwpkgdb/Info/getInfoLabel.php?id=3484815&wwwpkgdb=fc2733d6468c073f1dd738aa598ede55&nocache=62541e24b6e240f187ac920cab60f9da&dir=&wwwpkgdb=fc2733d6468c073f1dd738aa598ede55) | rpmG | ribosomal protein L33 |
| [OCAR_5783](https://www.genoscope.cns.fr/agc/mage/wwwpkgdb/Info/getInfoLabel.php?id=3484820&wwwpkgdb=fc2733d6468c073f1dd738aa598ede55&nocache=62541e24b6e240f187ac920cab60f9da&dir=&wwwpkgdb=fc2733d6468c073f1dd738aa598ede55) | _ | DNA topoisomerase 1 |
| [OCAR_5785](https://www.genoscope.cns.fr/agc/mage/wwwpkgdb/Info/getInfoLabel.php?id=3484821&wwwpkgdb=fc2733d6468c073f1dd738aa598ede55&nocache=62541e24b6e240f187ac920cab60f9da&dir=&wwwpkgdb=fc2733d6468c073f1dd738aa598ede55) | _ | SMF protein |
| [OCAR_5787](https://www.genoscope.cns.fr/agc/mage/wwwpkgdb/Info/getInfoLabel.php?id=3484823&wwwpkgdb=fc2733d6468c073f1dd738aa598ede55&nocache=62541e24b6e240f187ac920cab60f9da&dir=&wwwpkgdb=fc2733d6468c073f1dd738aa598ede55) | _ | dihydroorotase (DHOase) |
| [OCAR_5788](https://www.genoscope.cns.fr/agc/mage/wwwpkgdb/Info/getInfoLabel.php?id=3484824&wwwpkgdb=fc2733d6468c073f1dd738aa598ede55&nocache=62541e24b6e240f187ac920cab60f9da&dir=&wwwpkgdb=fc2733d6468c073f1dd738aa598ede55) | pyrB | Aspartate carbamoyltransferase |
| [OCAR_5791](https://www.genoscope.cns.fr/agc/mage/wwwpkgdb/Info/getInfoLabel.php?id=3483151&wwwpkgdb=fc2733d6468c073f1dd738aa598ede55&nocache=62541e24b6e240f187ac920cab60f9da&dir=&wwwpkgdb=fc2733d6468c073f1dd738aa598ede55) | _ | protein AidB |
| [OCAR_5793](https://www.genoscope.cns.fr/agc/mage/wwwpkgdb/Info/getInfoLabel.php?id=3484826&wwwpkgdb=fc2733d6468c073f1dd738aa598ede55&nocache=62541e24b6e240f187ac920cab60f9da&dir=&wwwpkgdb=fc2733d6468c073f1dd738aa598ede55) | _ | holliday junction resolvase YqgF |
| [OCAR_5794](https://www.genoscope.cns.fr/agc/mage/wwwpkgdb/Info/getInfoLabel.php?id=3483153&wwwpkgdb=fc2733d6468c073f1dd738aa598ede55&nocache=62541e24b6e240f187ac920cab60f9da&dir=&wwwpkgdb=fc2733d6468c073f1dd738aa598ede55) | gatC | Aspartyl/glutamyl-tRNA(Asn/Gln) amidotransferase subunit C |
| [OCAR_5796](https://www.genoscope.cns.fr/agc/mage/wwwpkgdb/Info/getInfoLabel.php?id=3483155&wwwpkgdb=fc2733d6468c073f1dd738aa598ede55&nocache=62541e24b6e240f187ac920cab60f9da&dir=&wwwpkgdb=fc2733d6468c073f1dd738aa598ede55) | gatA | Glutamyl-tRNA(Gln) amidotransferase subunit A |
| [OCAR_5800](https://www.genoscope.cns.fr/agc/mage/wwwpkgdb/Info/getInfoLabel.php?id=3483159&wwwpkgdb=fc2733d6468c073f1dd738aa598ede55&nocache=62541e24b6e240f187ac920cab60f9da&dir=&wwwpkgdb=fc2733d6468c073f1dd738aa598ede55) | gatB | Aspartyl/glutamyl-tRNA(Asn/Gln) amidotransferase subunit B |
| [OCAR_5803](https://www.genoscope.cns.fr/agc/mage/wwwpkgdb/Info/getInfoLabel.php?id=3483160&wwwpkgdb=fc2733d6468c073f1dd738aa598ede55&nocache=62541e24b6e240f187ac920cab60f9da&dir=&wwwpkgdb=fc2733d6468c073f1dd738aa598ede55) | _ | hypothetical protein |
| [OCAR_5830](https://www.genoscope.cns.fr/agc/mage/wwwpkgdb/Info/getInfoLabel.php?id=3483175&wwwpkgdb=fc2733d6468c073f1dd738aa598ede55&nocache=62541e24b6e240f187ac920cab60f9da&dir=&wwwpkgdb=fc2733d6468c073f1dd738aa598ede55) | dapA | Dihydrodipicolinate synthase |
| [OCAR_5831](https://www.genoscope.cns.fr/agc/mage/wwwpkgdb/Info/getInfoLabel.php?id=3483176&wwwpkgdb=fc2733d6468c073f1dd738aa598ede55&nocache=62541e24b6e240f187ac920cab60f9da&dir=&wwwpkgdb=fc2733d6468c073f1dd738aa598ede55) | mscL | Large-conductance mechanosensitive channel |
| [OCAR_5832](https://www.genoscope.cns.fr/agc/mage/wwwpkgdb/Info/getInfoLabel.php?id=3483177&wwwpkgdb=fc2733d6468c073f1dd738aa598ede55&nocache=62541e24b6e240f187ac920cab60f9da&dir=&wwwpkgdb=fc2733d6468c073f1dd738aa598ede55) | smpB | SsrA-binding protein |
| [OCAR_5835](https://www.genoscope.cns.fr/agc/mage/wwwpkgdb/Info/getInfoLabel.php?id=3484842&wwwpkgdb=fc2733d6468c073f1dd738aa598ede55&nocache=62541e24b6e240f187ac920cab60f9da&dir=&wwwpkgdb=fc2733d6468c073f1dd738aa598ede55) | _ | DUF88 |
| [OCAR_5837](https://www.genoscope.cns.fr/agc/mage/wwwpkgdb/Info/getInfoLabel.php?id=3483179&wwwpkgdb=fc2733d6468c073f1dd738aa598ede55&nocache=62541e24b6e240f187ac920cab60f9da&dir=&wwwpkgdb=fc2733d6468c073f1dd738aa598ede55) | rpoZ | DNA-directed RNA polymerase subunit omega |
| [OCAR_5838](https://www.genoscope.cns.fr/agc/mage/wwwpkgdb/Info/getInfoLabel.php?id=3483180&wwwpkgdb=fc2733d6468c073f1dd738aa598ede55&nocache=62541e24b6e240f187ac920cab60f9da&dir=&wwwpkgdb=fc2733d6468c073f1dd738aa598ede55) | _ | GTP pyrophosphokinase |
| [OCAR_5839](https://www.genoscope.cns.fr/agc/mage/wwwpkgdb/Info/getInfoLabel.php?id=3483181&wwwpkgdb=fc2733d6468c073f1dd738aa598ede55&nocache=62541e24b6e240f187ac920cab60f9da&dir=&wwwpkgdb=fc2733d6468c073f1dd738aa598ede55) | pdxJ | pyridoxal phosphate biosynthetic protein PdxJ |
| [OCAR_5840](https://www.genoscope.cns.fr/agc/mage/wwwpkgdb/Info/getInfoLabel.php?id=3483182&wwwpkgdb=fc2733d6468c073f1dd738aa598ede55&nocache=62541e24b6e240f187ac920cab60f9da&dir=&wwwpkgdb=fc2733d6468c073f1dd738aa598ede55) | acpS | Holo-[acyl-carrier-protein] synthase |
| [OCAR_5841](https://www.genoscope.cns.fr/agc/mage/wwwpkgdb/Info/getInfoLabel.php?id=3483183&wwwpkgdb=fc2733d6468c073f1dd738aa598ede55&nocache=62541e24b6e240f187ac920cab60f9da&dir=&wwwpkgdb=fc2733d6468c073f1dd738aa598ede55) | lepB | signal peptidase I |
| [OCAR_5843](https://www.genoscope.cns.fr/agc/mage/wwwpkgdb/Info/getInfoLabel.php?id=3483185&wwwpkgdb=fc2733d6468c073f1dd738aa598ede55&nocache=62541e24b6e240f187ac920cab60f9da&dir=&wwwpkgdb=fc2733d6468c073f1dd738aa598ede55) | era | GTP-binding protein era homolog |
| [OCAR_5846](https://www.genoscope.cns.fr/agc/mage/wwwpkgdb/Info/getInfoLabel.php?id=3483188&wwwpkgdb=fc2733d6468c073f1dd738aa598ede55&nocache=62541e24b6e240f187ac920cab60f9da&dir=&wwwpkgdb=fc2733d6468c073f1dd738aa598ede55) | recO | DNA repair protein recO |
| [OCAR_5848](https://www.genoscope.cns.fr/agc/mage/wwwpkgdb/Info/getInfoLabel.php?id=3483189&wwwpkgdb=fc2733d6468c073f1dd738aa598ede55&nocache=62541e24b6e240f187ac920cab60f9da&dir=&wwwpkgdb=fc2733d6468c073f1dd738aa598ede55) | parC | DNA topoisomerase IV, A subunit |
| [OCAR_5853](https://www.genoscope.cns.fr/agc/mage/wwwpkgdb/Info/getInfoLabel.php?id=3484846&wwwpkgdb=fc2733d6468c073f1dd738aa598ede55&nocache=62541e24b6e240f187ac920cab60f9da&dir=&wwwpkgdb=fc2733d6468c073f1dd738aa598ede55) | ate | Putative arginyl-tRNA--protein transferase |
| [OCAR_5855](https://www.genoscope.cns.fr/agc/mage/wwwpkgdb/Info/getInfoLabel.php?id=3484848&wwwpkgdb=fc2733d6468c073f1dd738aa598ede55&nocache=62541e24b6e240f187ac920cab60f9da&dir=&wwwpkgdb=fc2733d6468c073f1dd738aa598ede55) | hemB | delta-aminolevulinic acid dehydratase |
| [OCAR_5856](https://www.genoscope.cns.fr/agc/mage/wwwpkgdb/Info/getInfoLabel.php?id=3483193&wwwpkgdb=fc2733d6468c073f1dd738aa598ede55&nocache=62541e24b6e240f187ac920cab60f9da&dir=&wwwpkgdb=fc2733d6468c073f1dd738aa598ede55) | _ | hypothetical protein |
| [OCAR_5857](https://www.genoscope.cns.fr/agc/mage/wwwpkgdb/Info/getInfoLabel.php?id=3483194&wwwpkgdb=fc2733d6468c073f1dd738aa598ede55&nocache=62541e24b6e240f187ac920cab60f9da&dir=&wwwpkgdb=fc2733d6468c073f1dd738aa598ede55) | _ | transcriptional regulator of MarR family |
| [OCAR_5860](https://www.genoscope.cns.fr/agc/mage/wwwpkgdb/Info/getInfoLabel.php?id=3483197&wwwpkgdb=fc2733d6468c073f1dd738aa598ede55&nocache=62541e24b6e240f187ac920cab60f9da&dir=&wwwpkgdb=fc2733d6468c073f1dd738aa598ede55) | glyA | Serine hydroxymethyltransferase |
| [OCAR_5861](https://www.genoscope.cns.fr/agc/mage/wwwpkgdb/Info/getInfoLabel.php?id=3483198&wwwpkgdb=fc2733d6468c073f1dd738aa598ede55&nocache=62541e24b6e240f187ac920cab60f9da&dir=&wwwpkgdb=fc2733d6468c073f1dd738aa598ede55) | nrdR | Transcriptional repressor nrdR |
| [OCAR_5863](https://www.genoscope.cns.fr/agc/mage/wwwpkgdb/Info/getInfoLabel.php?id=3483200&wwwpkgdb=fc2733d6468c073f1dd738aa598ede55&nocache=62541e24b6e240f187ac920cab60f9da&dir=&wwwpkgdb=fc2733d6468c073f1dd738aa598ede55) | ribE | riboflavin synthase, alpha subunit |
| [OCAR_5864](https://www.genoscope.cns.fr/agc/mage/wwwpkgdb/Info/getInfoLabel.php?id=3483201&wwwpkgdb=fc2733d6468c073f1dd738aa598ede55&nocache=62541e24b6e240f187ac920cab60f9da&dir=&wwwpkgdb=fc2733d6468c073f1dd738aa598ede55) | ribH | 6,7-dimethyl-8-ribityllumazine synthase |
| [OCAR_5865](https://www.genoscope.cns.fr/agc/mage/wwwpkgdb/Info/getInfoLabel.php?id=3483202&wwwpkgdb=fc2733d6468c073f1dd738aa598ede55&nocache=62541e24b6e240f187ac920cab60f9da&dir=&wwwpkgdb=fc2733d6468c073f1dd738aa598ede55) | nusB | transcription antitermination factor NusB |
| [OCAR_5867](https://www.genoscope.cns.fr/agc/mage/wwwpkgdb/Info/getInfoLabel.php?id=3483204&wwwpkgdb=fc2733d6468c073f1dd738aa598ede55&nocache=62541e24b6e240f187ac920cab60f9da&dir=&wwwpkgdb=fc2733d6468c073f1dd738aa598ede55) | _ | V-type H(+)-translocating pyrophosphatase |
| [OCAR_5872](https://www.genoscope.cns.fr/agc/mage/wwwpkgdb/Info/getInfoLabel.php?id=3484851&wwwpkgdb=fc2733d6468c073f1dd738aa598ede55&nocache=62541e24b6e240f187ac920cab60f9da&dir=&wwwpkgdb=fc2733d6468c073f1dd738aa598ede55) | _ | SmpA/OmlA |
| [OCAR_5875](https://www.genoscope.cns.fr/agc/mage/wwwpkgdb/Info/getInfoLabel.php?id=3483209&wwwpkgdb=fc2733d6468c073f1dd738aa598ede55&nocache=62541e24b6e240f187ac920cab60f9da&dir=&wwwpkgdb=fc2733d6468c073f1dd738aa598ede55) | plsX | Fatty acid/phospholipid synthesis protein plsX |
| [OCAR_5876](https://www.genoscope.cns.fr/agc/mage/wwwpkgdb/Info/getInfoLabel.php?id=3483210&wwwpkgdb=fc2733d6468c073f1dd738aa598ede55&nocache=62541e24b6e240f187ac920cab60f9da&dir=&wwwpkgdb=fc2733d6468c073f1dd738aa598ede55) | _ | 3-oxoacyl-[acyl-carrier-protein] synthase 3 |
| [OCAR_5877](https://www.genoscope.cns.fr/agc/mage/wwwpkgdb/Info/getInfoLabel.php?id=3483211&wwwpkgdb=fc2733d6468c073f1dd738aa598ede55&nocache=62541e24b6e240f187ac920cab60f9da&dir=&wwwpkgdb=fc2733d6468c073f1dd738aa598ede55) | ihfA | Integration host factor subunit alpha |
| [OCAR_5885](https://www.genoscope.cns.fr/agc/mage/wwwpkgdb/Info/getInfoLabel.php?id=3484854&wwwpkgdb=fc2733d6468c073f1dd738aa598ede55&nocache=62541e24b6e240f187ac920cab60f9da&dir=&wwwpkgdb=fc2733d6468c073f1dd738aa598ede55) | _ | undecaprenyl-phosphate glucose phosphotransferase |
| [OCAR_5886](https://www.genoscope.cns.fr/agc/mage/wwwpkgdb/Info/getInfoLabel.php?id=3484855&wwwpkgdb=fc2733d6468c073f1dd738aa598ede55&nocache=62541e24b6e240f187ac920cab60f9da&dir=&wwwpkgdb=fc2733d6468c073f1dd738aa598ede55) | _ | glycosyl transferase, group 1 |
| [OCAR_5892](https://www.genoscope.cns.fr/agc/mage/wwwpkgdb/Info/getInfoLabel.php?id=3484858&wwwpkgdb=fc2733d6468c073f1dd738aa598ede55&nocache=62541e24b6e240f187ac920cab60f9da&dir=&wwwpkgdb=fc2733d6468c073f1dd738aa598ede55) | _ | O-acetylhomoserine (thiol)-lyase |
| [OCAR_5893](https://www.genoscope.cns.fr/agc/mage/wwwpkgdb/Info/getInfoLabel.php?id=3484859&wwwpkgdb=fc2733d6468c073f1dd738aa598ede55&nocache=62541e24b6e240f187ac920cab60f9da&dir=&wwwpkgdb=fc2733d6468c073f1dd738aa598ede55) | _ | CoA-binding |
| [OCAR_5894](https://www.genoscope.cns.fr/agc/mage/wwwpkgdb/Info/getInfoLabel.php?id=3483220&wwwpkgdb=fc2733d6468c073f1dd738aa598ede55&nocache=62541e24b6e240f187ac920cab60f9da&dir=&wwwpkgdb=fc2733d6468c073f1dd738aa598ede55) | _ | thioesterase family protein |
| [OCAR_5895](https://www.genoscope.cns.fr/agc/mage/wwwpkgdb/Info/getInfoLabel.php?id=3483221&wwwpkgdb=fc2733d6468c073f1dd738aa598ede55&nocache=62541e24b6e240f187ac920cab60f9da&dir=&wwwpkgdb=fc2733d6468c073f1dd738aa598ede55) | rplM | 50S ribosomal protein L13 |
| [OCAR_5896](https://www.genoscope.cns.fr/agc/mage/wwwpkgdb/Info/getInfoLabel.php?id=3483222&wwwpkgdb=fc2733d6468c073f1dd738aa598ede55&nocache=62541e24b6e240f187ac920cab60f9da&dir=&wwwpkgdb=fc2733d6468c073f1dd738aa598ede55) | rpsI | 30S ribosomal protein S9 |
| [OCAR_5901](https://www.genoscope.cns.fr/agc/mage/wwwpkgdb/Info/getInfoLabel.php?id=3484861&wwwpkgdb=fc2733d6468c073f1dd738aa598ede55&nocache=62541e24b6e240f187ac920cab60f9da&dir=&wwwpkgdb=fc2733d6468c073f1dd738aa598ede55) | glnA | glutamine synthetase, type I |
| [OCAR_5902](https://www.genoscope.cns.fr/agc/mage/wwwpkgdb/Info/getInfoLabel.php?id=3484862&wwwpkgdb=fc2733d6468c073f1dd738aa598ede55&nocache=62541e24b6e240f187ac920cab60f9da&dir=&wwwpkgdb=fc2733d6468c073f1dd738aa598ede55) | _ | nitrogen regulatory protein P-II |
| [OCAR_5908](https://www.genoscope.cns.fr/agc/mage/wwwpkgdb/Info/getInfoLabel.php?id=3483229&wwwpkgdb=fc2733d6468c073f1dd738aa598ede55&nocache=62541e24b6e240f187ac920cab60f9da&dir=&wwwpkgdb=fc2733d6468c073f1dd738aa598ede55) | clpP | ATP-dependent Clp protease proteolytic subunit |
| [OCAR_5909](https://www.genoscope.cns.fr/agc/mage/wwwpkgdb/Info/getInfoLabel.php?id=3483230&wwwpkgdb=fc2733d6468c073f1dd738aa598ede55&nocache=62541e24b6e240f187ac920cab60f9da&dir=&wwwpkgdb=fc2733d6468c073f1dd738aa598ede55) | clpX | ATP-dependent Clp protease ATP-binding subunit clpX |
| [OCAR_5911](https://www.genoscope.cns.fr/agc/mage/wwwpkgdb/Info/getInfoLabel.php?id=3483231&wwwpkgdb=fc2733d6468c073f1dd738aa598ede55&nocache=62541e24b6e240f187ac920cab60f9da&dir=&wwwpkgdb=fc2733d6468c073f1dd738aa598ede55) | lon | ATP-dependent protease La |
| [OCAR_5917](https://www.genoscope.cns.fr/agc/mage/wwwpkgdb/Info/getInfoLabel.php?id=3484869&wwwpkgdb=fc2733d6468c073f1dd738aa598ede55&nocache=62541e24b6e240f187ac920cab60f9da&dir=&wwwpkgdb=fc2733d6468c073f1dd738aa598ede55) | pepT | peptidase T |
| [OCAR_5919](https://www.genoscope.cns.fr/agc/mage/wwwpkgdb/Info/getInfoLabel.php?id=3483234&wwwpkgdb=fc2733d6468c073f1dd738aa598ede55&nocache=62541e24b6e240f187ac920cab60f9da&dir=&wwwpkgdb=fc2733d6468c073f1dd738aa598ede55) | _ | DNA-binding protein HU 1 |
| [OCAR_5922](https://www.genoscope.cns.fr/agc/mage/wwwpkgdb/Info/getInfoLabel.php?id=3483237&wwwpkgdb=fc2733d6468c073f1dd738aa598ede55&nocache=62541e24b6e240f187ac920cab60f9da&dir=&wwwpkgdb=fc2733d6468c073f1dd738aa598ede55) | _ | NADH-quinone oxidoreductase subunit a |
| [OCAR_5923](https://www.genoscope.cns.fr/agc/mage/wwwpkgdb/Info/getInfoLabel.php?id=3483238&wwwpkgdb=fc2733d6468c073f1dd738aa598ede55&nocache=62541e24b6e240f187ac920cab60f9da&dir=&wwwpkgdb=fc2733d6468c073f1dd738aa598ede55) | nuoB | NADH-quinone oxidoreductase subunit B |
| [OCAR_5924](https://www.genoscope.cns.fr/agc/mage/wwwpkgdb/Info/getInfoLabel.php?id=3483239&wwwpkgdb=fc2733d6468c073f1dd738aa598ede55&nocache=62541e24b6e240f187ac920cab60f9da&dir=&wwwpkgdb=fc2733d6468c073f1dd738aa598ede55) | _ | NADH-quinone oxidoreductase subunit c |
| [OCAR_5926](https://www.genoscope.cns.fr/agc/mage/wwwpkgdb/Info/getInfoLabel.php?id=3483241&wwwpkgdb=fc2733d6468c073f1dd738aa598ede55&nocache=62541e24b6e240f187ac920cab60f9da&dir=&wwwpkgdb=fc2733d6468c073f1dd738aa598ede55) | nuoD | NADH-quinone oxidoreductase subunit D |
| [OCAR_5928](https://www.genoscope.cns.fr/agc/mage/wwwpkgdb/Info/getInfoLabel.php?id=3483243&wwwpkgdb=fc2733d6468c073f1dd738aa598ede55&nocache=62541e24b6e240f187ac920cab60f9da&dir=&wwwpkgdb=fc2733d6468c073f1dd738aa598ede55) | nuoF | f subunit of NADH oxidoreductase (quinone) |
| [OCAR_5929](https://www.genoscope.cns.fr/agc/mage/wwwpkgdb/Info/getInfoLabel.php?id=3483244&wwwpkgdb=fc2733d6468c073f1dd738aa598ede55&nocache=62541e24b6e240f187ac920cab60f9da&dir=&wwwpkgdb=fc2733d6468c073f1dd738aa598ede55) | nuoG | g subunit of NADH dehydrogenase (quinone) |
| [OCAR_5930](https://www.genoscope.cns.fr/agc/mage/wwwpkgdb/Info/getInfoLabel.php?id=3483245&wwwpkgdb=fc2733d6468c073f1dd738aa598ede55&nocache=62541e24b6e240f187ac920cab60f9da&dir=&wwwpkgdb=fc2733d6468c073f1dd738aa598ede55) | _ | NADH-quinone oxidoreductase subunit h |
| [OCAR_5931](https://www.genoscope.cns.fr/agc/mage/wwwpkgdb/Info/getInfoLabel.php?id=3483246&wwwpkgdb=fc2733d6468c073f1dd738aa598ede55&nocache=62541e24b6e240f187ac920cab60f9da&dir=&wwwpkgdb=fc2733d6468c073f1dd738aa598ede55) | nuoI | NADH-quinone oxidoreductase subunit I |
| [OCAR_5932](https://www.genoscope.cns.fr/agc/mage/wwwpkgdb/Info/getInfoLabel.php?id=3483247&wwwpkgdb=fc2733d6468c073f1dd738aa598ede55&nocache=62541e24b6e240f187ac920cab60f9da&dir=&wwwpkgdb=fc2733d6468c073f1dd738aa598ede55) | nuoJ | j subunit of NADH dehydrogenase i |
| [OCAR_5933](https://www.genoscope.cns.fr/agc/mage/wwwpkgdb/Info/getInfoLabel.php?id=3483248&wwwpkgdb=fc2733d6468c073f1dd738aa598ede55&nocache=62541e24b6e240f187ac920cab60f9da&dir=&wwwpkgdb=fc2733d6468c073f1dd738aa598ede55) | _ | NADH-quinone oxidoreductase subunit k |
| [OCAR_5934](https://www.genoscope.cns.fr/agc/mage/wwwpkgdb/Info/getInfoLabel.php?id=3483249&wwwpkgdb=fc2733d6468c073f1dd738aa598ede55&nocache=62541e24b6e240f187ac920cab60f9da&dir=&wwwpkgdb=fc2733d6468c073f1dd738aa598ede55) | _ | NADH-quinone oxidoreductase subunit l |
| [OCAR_5935](https://www.genoscope.cns.fr/agc/mage/wwwpkgdb/Info/getInfoLabel.php?id=3483250&wwwpkgdb=fc2733d6468c073f1dd738aa598ede55&nocache=62541e24b6e240f187ac920cab60f9da&dir=&wwwpkgdb=fc2733d6468c073f1dd738aa598ede55) | _ | NADH-quinone oxidoreductase subunit m |
| [OCAR_5936](https://www.genoscope.cns.fr/agc/mage/wwwpkgdb/Info/getInfoLabel.php?id=3483251&wwwpkgdb=fc2733d6468c073f1dd738aa598ede55&nocache=62541e24b6e240f187ac920cab60f9da&dir=&wwwpkgdb=fc2733d6468c073f1dd738aa598ede55) | _ | NADH-quinone oxidoreductase subunit n |
| [OCAR_5938](https://www.genoscope.cns.fr/agc/mage/wwwpkgdb/Info/getInfoLabel.php?id=3483253&wwwpkgdb=fc2733d6468c073f1dd738aa598ede55&nocache=62541e24b6e240f187ac920cab60f9da&dir=&wwwpkgdb=fc2733d6468c073f1dd738aa598ede55) | _ | beta-lactamase domain protein |
| [OCAR_5939](https://www.genoscope.cns.fr/agc/mage/wwwpkgdb/Info/getInfoLabel.php?id=3483254&wwwpkgdb=fc2733d6468c073f1dd738aa598ede55&nocache=62541e24b6e240f187ac920cab60f9da&dir=&wwwpkgdb=fc2733d6468c073f1dd738aa598ede55) | mce | methylmalonyl-CoA epimerase |
| [OCAR_5947](https://www.genoscope.cns.fr/agc/mage/wwwpkgdb/Info/getInfoLabel.php?id=3483262&wwwpkgdb=fc2733d6468c073f1dd738aa598ede55&nocache=62541e24b6e240f187ac920cab60f9da&dir=&wwwpkgdb=fc2733d6468c073f1dd738aa598ede55) | proS | Prolyl-tRNA synthetase |
| [OCAR_5948](https://www.genoscope.cns.fr/agc/mage/wwwpkgdb/Info/getInfoLabel.php?id=3483263&wwwpkgdb=fc2733d6468c073f1dd738aa598ede55&nocache=62541e24b6e240f187ac920cab60f9da&dir=&wwwpkgdb=fc2733d6468c073f1dd738aa598ede55) | lolC | lipoprotein releasing system transmembrane protein LolC |
| [OCAR_5949](https://www.genoscope.cns.fr/agc/mage/wwwpkgdb/Info/getInfoLabel.php?id=3483264&wwwpkgdb=fc2733d6468c073f1dd738aa598ede55&nocache=62541e24b6e240f187ac920cab60f9da&dir=&wwwpkgdb=fc2733d6468c073f1dd738aa598ede55) | _ | lipoprotein-releasing system ATP-binding protein LolD |
| [OCAR_5953](https://www.genoscope.cns.fr/agc/mage/wwwpkgdb/Info/getInfoLabel.php?id=3483265&wwwpkgdb=fc2733d6468c073f1dd738aa598ede55&nocache=62541e24b6e240f187ac920cab60f9da&dir=&wwwpkgdb=fc2733d6468c073f1dd738aa598ede55) | _ | DNA polymerase III subunit alpha |
| [OCAR_5955](https://www.genoscope.cns.fr/agc/mage/wwwpkgdb/Info/getInfoLabel.php?id=3483267&wwwpkgdb=fc2733d6468c073f1dd738aa598ede55&nocache=62541e24b6e240f187ac920cab60f9da&dir=&wwwpkgdb=fc2733d6468c073f1dd738aa598ede55) | rpsB | 30S ribosomal protein S2 |
| [OCAR_5956](https://www.genoscope.cns.fr/agc/mage/wwwpkgdb/Info/getInfoLabel.php?id=3483268&wwwpkgdb=fc2733d6468c073f1dd738aa598ede55&nocache=62541e24b6e240f187ac920cab60f9da&dir=&wwwpkgdb=fc2733d6468c073f1dd738aa598ede55) | tsf | translation elongation factor Ts |
| [OCAR_5957](https://www.genoscope.cns.fr/agc/mage/wwwpkgdb/Info/getInfoLabel.php?id=3483269&wwwpkgdb=fc2733d6468c073f1dd738aa598ede55&nocache=62541e24b6e240f187ac920cab60f9da&dir=&wwwpkgdb=fc2733d6468c073f1dd738aa598ede55) | pyrH | UMP kinase |
| [OCAR_5958](https://www.genoscope.cns.fr/agc/mage/wwwpkgdb/Info/getInfoLabel.php?id=3483270&wwwpkgdb=fc2733d6468c073f1dd738aa598ede55&nocache=62541e24b6e240f187ac920cab60f9da&dir=&wwwpkgdb=fc2733d6468c073f1dd738aa598ede55) | frr | Ribosome-recycling factor |
| [OCAR_5959](https://www.genoscope.cns.fr/agc/mage/wwwpkgdb/Info/getInfoLabel.php?id=3483271&wwwpkgdb=fc2733d6468c073f1dd738aa598ede55&nocache=62541e24b6e240f187ac920cab60f9da&dir=&wwwpkgdb=fc2733d6468c073f1dd738aa598ede55) | uppS | di-trans,poly-cis-decaprenylcistransferase |
| [OCAR_5966](https://www.genoscope.cns.fr/agc/mage/wwwpkgdb/Info/getInfoLabel.php?id=3483277&wwwpkgdb=fc2733d6468c073f1dd738aa598ede55&nocache=62541e24b6e240f187ac920cab60f9da&dir=&wwwpkgdb=fc2733d6468c073f1dd738aa598ede55) | fabZ | beta-hydroxyacyl-(acyl-carrier-protein) dehydratase FabZ |
| [OCAR_5970](https://www.genoscope.cns.fr/agc/mage/wwwpkgdb/Info/getInfoLabel.php?id=3483281&wwwpkgdb=fc2733d6468c073f1dd738aa598ede55&nocache=62541e24b6e240f187ac920cab60f9da&dir=&wwwpkgdb=fc2733d6468c073f1dd738aa598ede55) | _ | methyl-accepting chemotaxis sensory transducer with Pas/Pac sensor |
| [OCAR_5971](https://www.genoscope.cns.fr/agc/mage/wwwpkgdb/Info/getInfoLabel.php?id=3484874&wwwpkgdb=fc2733d6468c073f1dd738aa598ede55&nocache=62541e24b6e240f187ac920cab60f9da&dir=&wwwpkgdb=fc2733d6468c073f1dd738aa598ede55) | gltA | citrate (Si)-synthase |
| [OCAR_5972](https://www.genoscope.cns.fr/agc/mage/wwwpkgdb/Info/getInfoLabel.php?id=3484875&wwwpkgdb=fc2733d6468c073f1dd738aa598ede55&nocache=62541e24b6e240f187ac920cab60f9da&dir=&wwwpkgdb=fc2733d6468c073f1dd738aa598ede55) | gltX | Glutamyl-tRNA synthetase |
| [OCAR_5973](https://www.genoscope.cns.fr/agc/mage/wwwpkgdb/Info/getInfoLabel.php?id=3483282&wwwpkgdb=fc2733d6468c073f1dd738aa598ede55&nocache=62541e24b6e240f187ac920cab60f9da&dir=&wwwpkgdb=fc2733d6468c073f1dd738aa598ede55) | glnS | glutaminyl-tRNA synthetase |
| [OCAR_5974](https://www.genoscope.cns.fr/agc/mage/wwwpkgdb/Info/getInfoLabel.php?id=3484876&wwwpkgdb=fc2733d6468c073f1dd738aa598ede55&nocache=62541e24b6e240f187ac920cab60f9da&dir=&wwwpkgdb=fc2733d6468c073f1dd738aa598ede55) | aat | Leucyl/phenylalanyl-tRNA--protein transferase |
| [OCAR_5976](https://www.genoscope.cns.fr/agc/mage/wwwpkgdb/Info/getInfoLabel.php?id=3484877&wwwpkgdb=fc2733d6468c073f1dd738aa598ede55&nocache=62541e24b6e240f187ac920cab60f9da&dir=&wwwpkgdb=fc2733d6468c073f1dd738aa598ede55) | _ | histidine kinase, dimerisation/phosphoacceptor |
| [OCAR_5977](https://www.genoscope.cns.fr/agc/mage/wwwpkgdb/Info/getInfoLabel.php?id=3484878&wwwpkgdb=fc2733d6468c073f1dd738aa598ede55&nocache=62541e24b6e240f187ac920cab60f9da&dir=&wwwpkgdb=fc2733d6468c073f1dd738aa598ede55) | _ | response regulator receiver protein |
| [OCAR_5979](https://www.genoscope.cns.fr/agc/mage/wwwpkgdb/Info/getInfoLabel.php?id=3484880&wwwpkgdb=fc2733d6468c073f1dd738aa598ede55&nocache=62541e24b6e240f187ac920cab60f9da&dir=&wwwpkgdb=fc2733d6468c073f1dd738aa598ede55) | accC | acetyl-CoA carboxylase |
| [OCAR_5981](https://www.genoscope.cns.fr/agc/mage/wwwpkgdb/Info/getInfoLabel.php?id=3484882&wwwpkgdb=fc2733d6468c073f1dd738aa598ede55&nocache=62541e24b6e240f187ac920cab60f9da&dir=&wwwpkgdb=fc2733d6468c073f1dd738aa598ede55) | aroQ | 3-dehydroquinate dehydratase |
| [OCAR_5983](https://www.genoscope.cns.fr/agc/mage/wwwpkgdb/Info/getInfoLabel.php?id=3484884&wwwpkgdb=fc2733d6468c073f1dd738aa598ede55&nocache=62541e24b6e240f187ac920cab60f9da&dir=&wwwpkgdb=fc2733d6468c073f1dd738aa598ede55) | _ | peptidase M48, Ste24p |
| [OCAR_5984](https://www.genoscope.cns.fr/agc/mage/wwwpkgdb/Info/getInfoLabel.php?id=3483284&wwwpkgdb=fc2733d6468c073f1dd738aa598ede55&nocache=62541e24b6e240f187ac920cab60f9da&dir=&wwwpkgdb=fc2733d6468c073f1dd738aa598ede55) | _ | aspartate transaminase |
| [OCAR_5987](https://www.genoscope.cns.fr/agc/mage/wwwpkgdb/Info/getInfoLabel.php?id=3483286&wwwpkgdb=fc2733d6468c073f1dd738aa598ede55&nocache=62541e24b6e240f187ac920cab60f9da&dir=&wwwpkgdb=fc2733d6468c073f1dd738aa598ede55) | _ | penicillin-binding protein 1A |
| [OCAR_5988](https://www.genoscope.cns.fr/agc/mage/wwwpkgdb/Info/getInfoLabel.php?id=3483287&wwwpkgdb=fc2733d6468c073f1dd738aa598ede55&nocache=62541e24b6e240f187ac920cab60f9da&dir=&wwwpkgdb=fc2733d6468c073f1dd738aa598ede55) | prfB | peptide chain release factor 2 |
| [OCAR_5997](https://www.genoscope.cns.fr/agc/mage/wwwpkgdb/Info/getInfoLabel.php?id=3484891&wwwpkgdb=fc2733d6468c073f1dd738aa598ede55&nocache=62541e24b6e240f187ac920cab60f9da&dir=&wwwpkgdb=fc2733d6468c073f1dd738aa598ede55) | _ | alpha/beta hydrolase |
| [OCAR_5999](https://www.genoscope.cns.fr/agc/mage/wwwpkgdb/Info/getInfoLabel.php?id=3483292&wwwpkgdb=fc2733d6468c073f1dd738aa598ede55&nocache=62541e24b6e240f187ac920cab60f9da&dir=&wwwpkgdb=fc2733d6468c073f1dd738aa598ede55) | sufB | FeS assembly protein SufB |
| [OCAR_6000](https://www.genoscope.cns.fr/agc/mage/wwwpkgdb/Info/getInfoLabel.php?id=3483293&wwwpkgdb=fc2733d6468c073f1dd738aa598ede55&nocache=62541e24b6e240f187ac920cab60f9da&dir=&wwwpkgdb=fc2733d6468c073f1dd738aa598ede55) | sufC | FeS assembly ATPase SufC |
| [OCAR_6002](https://www.genoscope.cns.fr/agc/mage/wwwpkgdb/Info/getInfoLabel.php?id=3483295&wwwpkgdb=fc2733d6468c073f1dd738aa598ede55&nocache=62541e24b6e240f187ac920cab60f9da&dir=&wwwpkgdb=fc2733d6468c073f1dd738aa598ede55) | _ | cysteine desulfurase |
| [OCAR_6003](https://www.genoscope.cns.fr/agc/mage/wwwpkgdb/Info/getInfoLabel.php?id=3483296&wwwpkgdb=fc2733d6468c073f1dd738aa598ede55&nocache=62541e24b6e240f187ac920cab60f9da&dir=&wwwpkgdb=fc2733d6468c073f1dd738aa598ede55) | _ | FeS assembly SUF system protein |
| [OCAR_6005](https://www.genoscope.cns.fr/agc/mage/wwwpkgdb/Info/getInfoLabel.php?id=3484892&wwwpkgdb=fc2733d6468c073f1dd738aa598ede55&nocache=62541e24b6e240f187ac920cab60f9da&dir=&wwwpkgdb=fc2733d6468c073f1dd738aa598ede55) | _ | diguanylate cyclase |
| [OCAR_6006](https://www.genoscope.cns.fr/agc/mage/wwwpkgdb/Info/getInfoLabel.php?id=3484893&wwwpkgdb=fc2733d6468c073f1dd738aa598ede55&nocache=62541e24b6e240f187ac920cab60f9da&dir=&wwwpkgdb=fc2733d6468c073f1dd738aa598ede55) | _ | putative ATP-dependent RNA helicase RhlE |
| [OCAR_6007](https://www.genoscope.cns.fr/agc/mage/wwwpkgdb/Info/getInfoLabel.php?id=3484894&wwwpkgdb=fc2733d6468c073f1dd738aa598ede55&nocache=62541e24b6e240f187ac920cab60f9da&dir=&wwwpkgdb=fc2733d6468c073f1dd738aa598ede55) | parE | DNA topoisomerase IV, B subunit |
| [OCAR_6011](https://www.genoscope.cns.fr/agc/mage/wwwpkgdb/Info/getInfoLabel.php?id=3484895&wwwpkgdb=fc2733d6468c073f1dd738aa598ede55&nocache=62541e24b6e240f187ac920cab60f9da&dir=&wwwpkgdb=fc2733d6468c073f1dd738aa598ede55) | _ | membrane-associated proteins in eicosanoid and glutathione metabolism |
| [OCAR_6012](https://www.genoscope.cns.fr/agc/mage/wwwpkgdb/Info/getInfoLabel.php?id=3484896&wwwpkgdb=fc2733d6468c073f1dd738aa598ede55&nocache=62541e24b6e240f187ac920cab60f9da&dir=&wwwpkgdb=fc2733d6468c073f1dd738aa598ede55) | _ | poly-beta-hydroxybutyrate polymerase |
| [OCAR_6015](https://www.genoscope.cns.fr/agc/mage/wwwpkgdb/Info/getInfoLabel.php?id=3483302&wwwpkgdb=fc2733d6468c073f1dd738aa598ede55&nocache=62541e24b6e240f187ac920cab60f9da&dir=&wwwpkgdb=fc2733d6468c073f1dd738aa598ede55) | _ | hypothetical aminotransferase YfdZ |
| [OCAR_6016](https://www.genoscope.cns.fr/agc/mage/wwwpkgdb/Info/getInfoLabel.php?id=3483303&wwwpkgdb=fc2733d6468c073f1dd738aa598ede55&nocache=62541e24b6e240f187ac920cab60f9da&dir=&wwwpkgdb=fc2733d6468c073f1dd738aa598ede55) | _ | homoserine dehydrogenase (HDH) |
| [OCAR_6017](https://www.genoscope.cns.fr/agc/mage/wwwpkgdb/Info/getInfoLabel.php?id=3483304&wwwpkgdb=fc2733d6468c073f1dd738aa598ede55&nocache=62541e24b6e240f187ac920cab60f9da&dir=&wwwpkgdb=fc2733d6468c073f1dd738aa598ede55) | glpX | fructose-1,6-bisphosphatase, class II |
| [OCAR_6019](https://www.genoscope.cns.fr/agc/mage/wwwpkgdb/Info/getInfoLabel.php?id=3483306&wwwpkgdb=fc2733d6468c073f1dd738aa598ede55&nocache=62541e24b6e240f187ac920cab60f9da&dir=&wwwpkgdb=fc2733d6468c073f1dd738aa598ede55) | recJ | single-stranded-DNA-specific exonuclease RecJ |
| [OCAR_6020](https://www.genoscope.cns.fr/agc/mage/wwwpkgdb/Info/getInfoLabel.php?id=3484898&wwwpkgdb=fc2733d6468c073f1dd738aa598ede55&nocache=62541e24b6e240f187ac920cab60f9da&dir=&wwwpkgdb=fc2733d6468c073f1dd738aa598ede55) | _ | lytic murein transglycosylase |
| [OCAR_6024](https://www.genoscope.cns.fr/agc/mage/wwwpkgdb/Info/getInfoLabel.php?id=3484902&wwwpkgdb=fc2733d6468c073f1dd738aa598ede55&nocache=62541e24b6e240f187ac920cab60f9da&dir=&wwwpkgdb=fc2733d6468c073f1dd738aa598ede55) | efp | Elongation factor P |
| [OCAR_6025](https://www.genoscope.cns.fr/agc/mage/wwwpkgdb/Info/getInfoLabel.php?id=3483307&wwwpkgdb=fc2733d6468c073f1dd738aa598ede55&nocache=62541e24b6e240f187ac920cab60f9da&dir=&wwwpkgdb=fc2733d6468c073f1dd738aa598ede55) | _ | putative lysyl-tRNA synthetase |
| [OCAR_6026](https://www.genoscope.cns.fr/agc/mage/wwwpkgdb/Info/getInfoLabel.php?id=3483308&wwwpkgdb=fc2733d6468c073f1dd738aa598ede55&nocache=62541e24b6e240f187ac920cab60f9da&dir=&wwwpkgdb=fc2733d6468c073f1dd738aa598ede55) | _ | L-lysine 2,3-aminomutase (KAM) (LAM) |
| [OCAR_6031](https://www.genoscope.cns.fr/agc/mage/wwwpkgdb/Info/getInfoLabel.php?id=3484903&wwwpkgdb=fc2733d6468c073f1dd738aa598ede55&nocache=62541e24b6e240f187ac920cab60f9da&dir=&wwwpkgdb=fc2733d6468c073f1dd738aa598ede55) | _ | glyoxalase/bleomycin resistance protein/dioxygenase |
| [OCAR_6033](https://www.genoscope.cns.fr/agc/mage/wwwpkgdb/Info/getInfoLabel.php?id=3483313&wwwpkgdb=fc2733d6468c073f1dd738aa598ede55&nocache=62541e24b6e240f187ac920cab60f9da&dir=&wwwpkgdb=fc2733d6468c073f1dd738aa598ede55) | _ | cold-shock DNA-binding domain protein |
| [OCAR_6045](https://www.genoscope.cns.fr/agc/mage/wwwpkgdb/Info/getInfoLabel.php?id=3483317&wwwpkgdb=fc2733d6468c073f1dd738aa598ede55&nocache=62541e24b6e240f187ac920cab60f9da&dir=&wwwpkgdb=fc2733d6468c073f1dd738aa598ede55) | _ | NADH dehydrogenase |
| [OCAR_6053](https://www.genoscope.cns.fr/agc/mage/wwwpkgdb/Info/getInfoLabel.php?id=3483323&wwwpkgdb=fc2733d6468c073f1dd738aa598ede55&nocache=62541e24b6e240f187ac920cab60f9da&dir=&wwwpkgdb=fc2733d6468c073f1dd738aa598ede55) | _ | glycosyl transferase, family 2 |
| [OCAR_6055](https://www.genoscope.cns.fr/agc/mage/wwwpkgdb/Info/getInfoLabel.php?id=3484915&wwwpkgdb=fc2733d6468c073f1dd738aa598ede55&nocache=62541e24b6e240f187ac920cab60f9da&dir=&wwwpkgdb=fc2733d6468c073f1dd738aa598ede55) | metC | cystathionine beta-lyase |
| [OCAR_6056](https://www.genoscope.cns.fr/agc/mage/wwwpkgdb/Info/getInfoLabel.php?id=3483325&wwwpkgdb=fc2733d6468c073f1dd738aa598ede55&nocache=62541e24b6e240f187ac920cab60f9da&dir=&wwwpkgdb=fc2733d6468c073f1dd738aa598ede55) | _ | general L-amino acid-binding periplasmic protein AapJ |
| [OCAR_6059](https://www.genoscope.cns.fr/agc/mage/wwwpkgdb/Info/getInfoLabel.php?id=3483328&wwwpkgdb=fc2733d6468c073f1dd738aa598ede55&nocache=62541e24b6e240f187ac920cab60f9da&dir=&wwwpkgdb=fc2733d6468c073f1dd738aa598ede55) | _ | general L-amino acid transport ATP-binding protein AapP |
| [OCAR_6064](https://www.genoscope.cns.fr/agc/mage/wwwpkgdb/Info/getInfoLabel.php?id=3483332&wwwpkgdb=fc2733d6468c073f1dd738aa598ede55&nocache=62541e24b6e240f187ac920cab60f9da&dir=&wwwpkgdb=fc2733d6468c073f1dd738aa598ede55) | _ | multidrug resistance protein MdtB |
| [OCAR_6065](https://www.genoscope.cns.fr/agc/mage/wwwpkgdb/Info/getInfoLabel.php?id=3483333&wwwpkgdb=fc2733d6468c073f1dd738aa598ede55&nocache=62541e24b6e240f187ac920cab60f9da&dir=&wwwpkgdb=fc2733d6468c073f1dd738aa598ede55) | _ | multidrug resistance protein MdtC |
| [OCAR_6087](https://www.genoscope.cns.fr/agc/mage/wwwpkgdb/Info/getInfoLabel.php?id=3483350&wwwpkgdb=fc2733d6468c073f1dd738aa598ede55&nocache=62541e24b6e240f187ac920cab60f9da&dir=&wwwpkgdb=fc2733d6468c073f1dd738aa598ede55) | _ | type I secretion outer membrane protein, TolC family |
| [OCAR_6089](https://www.genoscope.cns.fr/agc/mage/wwwpkgdb/Info/getInfoLabel.php?id=3483352&wwwpkgdb=fc2733d6468c073f1dd738aa598ede55&nocache=62541e24b6e240f187ac920cab60f9da&dir=&wwwpkgdb=fc2733d6468c073f1dd738aa598ede55) | valS | valyl-tRNA synthetase |
| [OCAR_6090](https://www.genoscope.cns.fr/agc/mage/wwwpkgdb/Info/getInfoLabel.php?id=3484922&wwwpkgdb=fc2733d6468c073f1dd738aa598ede55&nocache=62541e24b6e240f187ac920cab60f9da&dir=&wwwpkgdb=fc2733d6468c073f1dd738aa598ede55) | _ | putative 3-methyladenine DNA glycosylase |
| [OCAR_6091](https://www.genoscope.cns.fr/agc/mage/wwwpkgdb/Info/getInfoLabel.php?id=3483353&wwwpkgdb=fc2733d6468c073f1dd738aa598ede55&nocache=62541e24b6e240f187ac920cab60f9da&dir=&wwwpkgdb=fc2733d6468c073f1dd738aa598ede55) | lipA | Lipoyl synthase |
| [OCAR_2074](https://www.genoscope.cns.fr/agc/mage/wwwpkgdb/Info/getInfoLabel.php?id=3481985&wwwpkgdb=fc2733d6468c073f1dd738aa598ede55&nocache=62541e24b6e240f187ac920cab60f9da&dir=&wwwpkgdb=fc2733d6468c073f1dd738aa598ede55) | _ | _ |
| [OCAR_6092](https://www.genoscope.cns.fr/agc/mage/wwwpkgdb/Info/getInfoLabel.php?id=3484923&wwwpkgdb=fc2733d6468c073f1dd738aa598ede55&nocache=62541e24b6e240f187ac920cab60f9da&dir=&wwwpkgdb=fc2733d6468c073f1dd738aa598ede55) | _ | CinA protein |
| [OCAR_6093](https://www.genoscope.cns.fr/agc/mage/wwwpkgdb/Info/getInfoLabel.php?id=3484924&wwwpkgdb=fc2733d6468c073f1dd738aa598ede55&nocache=62541e24b6e240f187ac920cab60f9da&dir=&wwwpkgdb=fc2733d6468c073f1dd738aa598ede55) | _ | IspD/IspF bifunctional enzyme |
| [OCAR_6094](https://www.genoscope.cns.fr/agc/mage/wwwpkgdb/Info/getInfoLabel.php?id=3483354&wwwpkgdb=fc2733d6468c073f1dd738aa598ede55&nocache=62541e24b6e240f187ac920cab60f9da&dir=&wwwpkgdb=fc2733d6468c073f1dd738aa598ede55) | _ | probable tRNA-dihydrouridine synthase |
| [OCAR_6095](https://www.genoscope.cns.fr/agc/mage/wwwpkgdb/Info/getInfoLabel.php?id=3483355&wwwpkgdb=fc2733d6468c073f1dd738aa598ede55&nocache=62541e24b6e240f187ac920cab60f9da&dir=&wwwpkgdb=fc2733d6468c073f1dd738aa598ede55) | _ | nitrogen regulation protein NtrB |
| [OCAR_6096](https://www.genoscope.cns.fr/agc/mage/wwwpkgdb/Info/getInfoLabel.php?id=3483356&wwwpkgdb=fc2733d6468c073f1dd738aa598ede55&nocache=62541e24b6e240f187ac920cab60f9da&dir=&wwwpkgdb=fc2733d6468c073f1dd738aa598ede55) | ntrC | nitrogen regulation protein NR(I) |
| [OCAR_6097](https://www.genoscope.cns.fr/agc/mage/wwwpkgdb/Info/getInfoLabel.php?id=3483357&wwwpkgdb=fc2733d6468c073f1dd738aa598ede55&nocache=62541e24b6e240f187ac920cab60f9da&dir=&wwwpkgdb=fc2733d6468c073f1dd738aa598ede55) | _ | nitrogen regulation protein NtrY |
| [OCAR_6098](https://www.genoscope.cns.fr/agc/mage/wwwpkgdb/Info/getInfoLabel.php?id=3483358&wwwpkgdb=fc2733d6468c073f1dd738aa598ede55&nocache=62541e24b6e240f187ac920cab60f9da&dir=&wwwpkgdb=fc2733d6468c073f1dd738aa598ede55) | _ | nitrogen assimilation regulatory protein NtrX |
| [OCAR_6100](https://www.genoscope.cns.fr/agc/mage/wwwpkgdb/Info/getInfoLabel.php?id=3483360&wwwpkgdb=fc2733d6468c073f1dd738aa598ede55&nocache=62541e24b6e240f187ac920cab60f9da&dir=&wwwpkgdb=fc2733d6468c073f1dd738aa598ede55) | hfq | Protein hfq |
| [OCAR_6101](https://www.genoscope.cns.fr/agc/mage/wwwpkgdb/Info/getInfoLabel.php?id=3483361&wwwpkgdb=fc2733d6468c073f1dd738aa598ede55&nocache=62541e24b6e240f187ac920cab60f9da&dir=&wwwpkgdb=fc2733d6468c073f1dd738aa598ede55) | hflX | GTP-binding proten HflX |
| [OCAR_6102](https://www.genoscope.cns.fr/agc/mage/wwwpkgdb/Info/getInfoLabel.php?id=3484925&wwwpkgdb=fc2733d6468c073f1dd738aa598ede55&nocache=62541e24b6e240f187ac920cab60f9da&dir=&wwwpkgdb=fc2733d6468c073f1dd738aa598ede55) | _ | MazG family protein |
| [OCAR_6103](https://www.genoscope.cns.fr/agc/mage/wwwpkgdb/Info/getInfoLabel.php?id=3483362&wwwpkgdb=fc2733d6468c073f1dd738aa598ede55&nocache=62541e24b6e240f187ac920cab60f9da&dir=&wwwpkgdb=fc2733d6468c073f1dd738aa598ede55) | _ | ExsB protein |
| [OCAR_6123](https://www.genoscope.cns.fr/agc/mage/wwwpkgdb/Info/getInfoLabel.php?id=3484941&wwwpkgdb=fc2733d6468c073f1dd738aa598ede55&nocache=62541e24b6e240f187ac920cab60f9da&dir=&wwwpkgdb=fc2733d6468c073f1dd738aa598ede55) | _ | hydrolase |
| [OCAR_6124](https://www.genoscope.cns.fr/agc/mage/wwwpkgdb/Info/getInfoLabel.php?id=3484942&wwwpkgdb=fc2733d6468c073f1dd738aa598ede55&nocache=62541e24b6e240f187ac920cab60f9da&dir=&wwwpkgdb=fc2733d6468c073f1dd738aa598ede55) | _ | putative deoxyribonuclease |
| [OCAR_6125](https://www.genoscope.cns.fr/agc/mage/wwwpkgdb/Info/getInfoLabel.php?id=3484943&wwwpkgdb=fc2733d6468c073f1dd738aa598ede55&nocache=62541e24b6e240f187ac920cab60f9da&dir=&wwwpkgdb=fc2733d6468c073f1dd738aa598ede55) | metG | methionyl-tRNA synthetase |
| [OCAR_6185](https://www.genoscope.cns.fr/agc/mage/wwwpkgdb/Info/getInfoLabel.php?id=3484992&wwwpkgdb=fc2733d6468c073f1dd738aa598ede55&nocache=62541e24b6e240f187ac920cab60f9da&dir=&wwwpkgdb=fc2733d6468c073f1dd738aa598ede55) | _ | AcrB protein |
| [OCAR_6199](https://www.genoscope.cns.fr/agc/mage/wwwpkgdb/Info/getInfoLabel.php?id=3484999&wwwpkgdb=fc2733d6468c073f1dd738aa598ede55&nocache=62541e24b6e240f187ac920cab60f9da&dir=&wwwpkgdb=fc2733d6468c073f1dd738aa598ede55) | _ | quinone oxidoreductase |
| [OCAR_6200](https://www.genoscope.cns.fr/agc/mage/wwwpkgdb/Info/getInfoLabel.php?id=3485000&wwwpkgdb=fc2733d6468c073f1dd738aa598ede55&nocache=62541e24b6e240f187ac920cab60f9da&dir=&wwwpkgdb=fc2733d6468c073f1dd738aa598ede55) | _ | protein YegH |
| [OCAR_6203](https://www.genoscope.cns.fr/agc/mage/wwwpkgdb/Info/getInfoLabel.php?id=3483386&wwwpkgdb=fc2733d6468c073f1dd738aa598ede55&nocache=62541e24b6e240f187ac920cab60f9da&dir=&wwwpkgdb=fc2733d6468c073f1dd738aa598ede55) | _ | hemimethylated DNA-binding region |
| [OCAR_6206](https://www.genoscope.cns.fr/agc/mage/wwwpkgdb/Info/getInfoLabel.php?id=3483387&wwwpkgdb=fc2733d6468c073f1dd738aa598ede55&nocache=62541e24b6e240f187ac920cab60f9da&dir=&wwwpkgdb=fc2733d6468c073f1dd738aa598ede55) | _ | extracellular solute-binding protein, family 5 |
| [OCAR_6210](https://www.genoscope.cns.fr/agc/mage/wwwpkgdb/Info/getInfoLabel.php?id=3485004&wwwpkgdb=fc2733d6468c073f1dd738aa598ede55&nocache=62541e24b6e240f187ac920cab60f9da&dir=&wwwpkgdb=fc2733d6468c073f1dd738aa598ede55) | mfd | transcription-repair coupling factor |
| [OCAR_6213](https://www.genoscope.cns.fr/agc/mage/wwwpkgdb/Info/getInfoLabel.php?id=3483391&wwwpkgdb=fc2733d6468c073f1dd738aa598ede55&nocache=62541e24b6e240f187ac920cab60f9da&dir=&wwwpkgdb=fc2733d6468c073f1dd738aa598ede55) | _ | ATP-dependent DNA helicase RecG |
| [OCAR_6216](https://www.genoscope.cns.fr/agc/mage/wwwpkgdb/Info/getInfoLabel.php?id=3485009&wwwpkgdb=fc2733d6468c073f1dd738aa598ede55&nocache=62541e24b6e240f187ac920cab60f9da&dir=&wwwpkgdb=fc2733d6468c073f1dd738aa598ede55) | glmS | glutamine-fructose-6-phosphate transaminase |
| [OCAR_6217](https://www.genoscope.cns.fr/agc/mage/wwwpkgdb/Info/getInfoLabel.php?id=3485010&wwwpkgdb=fc2733d6468c073f1dd738aa598ede55&nocache=62541e24b6e240f187ac920cab60f9da&dir=&wwwpkgdb=fc2733d6468c073f1dd738aa598ede55) | glmU | Bifunctional protein glmU [Includes: UDP-N-acetylglucosamine pyrophosphorylase ; Glucosamine-1-phosphate N-acetyltransferase] |
| [OCAR_6221](https://www.genoscope.cns.fr/agc/mage/wwwpkgdb/Info/getInfoLabel.php?id=3483394&wwwpkgdb=fc2733d6468c073f1dd738aa598ede55&nocache=62541e24b6e240f187ac920cab60f9da&dir=&wwwpkgdb=fc2733d6468c073f1dd738aa598ede55) | _ | glycosyl transferase, family 2 |
| [OCAR_6225](https://www.genoscope.cns.fr/agc/mage/wwwpkgdb/Info/getInfoLabel.php?id=3485015&wwwpkgdb=fc2733d6468c073f1dd738aa598ede55&nocache=62541e24b6e240f187ac920cab60f9da&dir=&wwwpkgdb=fc2733d6468c073f1dd738aa598ede55) | cysK | cysteine synthase A |
| [OCAR_6226](https://www.genoscope.cns.fr/agc/mage/wwwpkgdb/Info/getInfoLabel.php?id=3485016&wwwpkgdb=fc2733d6468c073f1dd738aa598ede55&nocache=62541e24b6e240f187ac920cab60f9da&dir=&wwwpkgdb=fc2733d6468c073f1dd738aa598ede55) | tgt | queuine tRNA-ribosyltransferase |
| [OCAR_6228](https://www.genoscope.cns.fr/agc/mage/wwwpkgdb/Info/getInfoLabel.php?id=3485017&wwwpkgdb=fc2733d6468c073f1dd738aa598ede55&nocache=62541e24b6e240f187ac920cab60f9da&dir=&wwwpkgdb=fc2733d6468c073f1dd738aa598ede55) | queA | S-adenosylmethionine:tRNA ribosyltransferase-isomerase |
| [OCAR_6229](https://www.genoscope.cns.fr/agc/mage/wwwpkgdb/Info/getInfoLabel.php?id=3485018&wwwpkgdb=fc2733d6468c073f1dd738aa598ede55&nocache=62541e24b6e240f187ac920cab60f9da&dir=&wwwpkgdb=fc2733d6468c073f1dd738aa598ede55) | _ | peptidyl-prolyl cis-trans isomerase, cyclophilin-type |
| [OCAR_6230](https://www.genoscope.cns.fr/agc/mage/wwwpkgdb/Info/getInfoLabel.php?id=3485019&wwwpkgdb=fc2733d6468c073f1dd738aa598ede55&nocache=62541e24b6e240f187ac920cab60f9da&dir=&wwwpkgdb=fc2733d6468c073f1dd738aa598ede55) | _ | peptidyl-prolyl cis-trans isomerase, cyclophilin type |
| [OCAR_6233](https://www.genoscope.cns.fr/agc/mage/wwwpkgdb/Info/getInfoLabel.php?id=3485021&wwwpkgdb=fc2733d6468c073f1dd738aa598ede55&nocache=62541e24b6e240f187ac920cab60f9da&dir=&wwwpkgdb=fc2733d6468c073f1dd738aa598ede55) | gyrA | DNA gyrase, A subunit |
| [OCAR_6235](https://www.genoscope.cns.fr/agc/mage/wwwpkgdb/Info/getInfoLabel.php?id=3485022&wwwpkgdb=fc2733d6468c073f1dd738aa598ede55&nocache=62541e24b6e240f187ac920cab60f9da&dir=&wwwpkgdb=fc2733d6468c073f1dd738aa598ede55) | _ | single-stranded DNA-binding protein (SSB) |
| [OCAR_6238](https://www.genoscope.cns.fr/agc/mage/wwwpkgdb/Info/getInfoLabel.php?id=3483400&wwwpkgdb=fc2733d6468c073f1dd738aa598ede55&nocache=62541e24b6e240f187ac920cab60f9da&dir=&wwwpkgdb=fc2733d6468c073f1dd738aa598ede55) | uvrA | excinuclease ABC, A subunit |
| [OCAR_6250](https://www.genoscope.cns.fr/agc/mage/wwwpkgdb/Info/getInfoLabel.php?id=3483407&wwwpkgdb=fc2733d6468c073f1dd738aa598ede55&nocache=62541e24b6e240f187ac920cab60f9da&dir=&wwwpkgdb=fc2733d6468c073f1dd738aa598ede55) | trmFO | tRNA:M(5)U-54 methyltransferase |
| [OCAR_6253](https://www.genoscope.cns.fr/agc/mage/wwwpkgdb/Info/getInfoLabel.php?id=3485030&wwwpkgdb=fc2733d6468c073f1dd738aa598ede55&nocache=62541e24b6e240f187ac920cab60f9da&dir=&wwwpkgdb=fc2733d6468c073f1dd738aa598ede55) | secF | protein-export membrane protein SecF |
| [OCAR_6254](https://www.genoscope.cns.fr/agc/mage/wwwpkgdb/Info/getInfoLabel.php?id=3485031&wwwpkgdb=fc2733d6468c073f1dd738aa598ede55&nocache=62541e24b6e240f187ac920cab60f9da&dir=&wwwpkgdb=fc2733d6468c073f1dd738aa598ede55) | secD | protein-export membrane protein SecD |
| [OCAR_6255](https://www.genoscope.cns.fr/agc/mage/wwwpkgdb/Info/getInfoLabel.php?id=3485032&wwwpkgdb=fc2733d6468c073f1dd738aa598ede55&nocache=62541e24b6e240f187ac920cab60f9da&dir=&wwwpkgdb=fc2733d6468c073f1dd738aa598ede55) | yajC | preprotein translocase, YajC subunit |
| [OCAR_6256](https://www.genoscope.cns.fr/agc/mage/wwwpkgdb/Info/getInfoLabel.php?id=3483408&wwwpkgdb=fc2733d6468c073f1dd738aa598ede55&nocache=62541e24b6e240f187ac920cab60f9da&dir=&wwwpkgdb=fc2733d6468c073f1dd738aa598ede55) | _ | ATP-dependent protease subunit |
| [OCAR_6262](https://www.genoscope.cns.fr/agc/mage/wwwpkgdb/Info/getInfoLabel.php?id=3483410&wwwpkgdb=fc2733d6468c073f1dd738aa598ede55&nocache=62541e24b6e240f187ac920cab60f9da&dir=&wwwpkgdb=fc2733d6468c073f1dd738aa598ede55) | _ | response regulator receiver protein |
| [OCAR_6263](https://www.genoscope.cns.fr/agc/mage/wwwpkgdb/Info/getInfoLabel.php?id=3485037&wwwpkgdb=fc2733d6468c073f1dd738aa598ede55&nocache=62541e24b6e240f187ac920cab60f9da&dir=&wwwpkgdb=fc2733d6468c073f1dd738aa598ede55) | surE | 5'-nucleotidase surE |
| [OCAR_6264](https://www.genoscope.cns.fr/agc/mage/wwwpkgdb/Info/getInfoLabel.php?id=3485038&wwwpkgdb=fc2733d6468c073f1dd738aa598ede55&nocache=62541e24b6e240f187ac920cab60f9da&dir=&wwwpkgdb=fc2733d6468c073f1dd738aa598ede55) | serS | Seryl-tRNA synthetase |
| [OCAR_6265](https://www.genoscope.cns.fr/agc/mage/wwwpkgdb/Info/getInfoLabel.php?id=3485039&wwwpkgdb=fc2733d6468c073f1dd738aa598ede55&nocache=62541e24b6e240f187ac920cab60f9da&dir=&wwwpkgdb=fc2733d6468c073f1dd738aa598ede55) | tatC | twin arginine-targeting protein translocase TatC |
| [OCAR_6269](https://www.genoscope.cns.fr/agc/mage/wwwpkgdb/Info/getInfoLabel.php?id=3485043&wwwpkgdb=fc2733d6468c073f1dd738aa598ede55&nocache=62541e24b6e240f187ac920cab60f9da&dir=&wwwpkgdb=fc2733d6468c073f1dd738aa598ede55) | _ | chromosome segregation and condensation protein ScpB |
| [OCAR_6270](https://www.genoscope.cns.fr/agc/mage/wwwpkgdb/Info/getInfoLabel.php?id=3485044&wwwpkgdb=fc2733d6468c073f1dd738aa598ede55&nocache=62541e24b6e240f187ac920cab60f9da&dir=&wwwpkgdb=fc2733d6468c073f1dd738aa598ede55) | _ | ScpA/B protein |
| [OCAR_6271](https://www.genoscope.cns.fr/agc/mage/wwwpkgdb/Info/getInfoLabel.php?id=3485045&wwwpkgdb=fc2733d6468c073f1dd738aa598ede55&nocache=62541e24b6e240f187ac920cab60f9da&dir=&wwwpkgdb=fc2733d6468c073f1dd738aa598ede55) | _ | beta-hexosaminidase |
| [OCAR_6273](https://www.genoscope.cns.fr/agc/mage/wwwpkgdb/Info/getInfoLabel.php?id=3485047&wwwpkgdb=fc2733d6468c073f1dd738aa598ede55&nocache=62541e24b6e240f187ac920cab60f9da&dir=&wwwpkgdb=fc2733d6468c073f1dd738aa598ede55) | argS | arginyl-tRNA synthetase |
| [OCAR_6275](https://www.genoscope.cns.fr/agc/mage/wwwpkgdb/Info/getInfoLabel.php?id=3483411&wwwpkgdb=fc2733d6468c073f1dd738aa598ede55&nocache=62541e24b6e240f187ac920cab60f9da&dir=&wwwpkgdb=fc2733d6468c073f1dd738aa598ede55) | _ | HesB/YadR/YfhF |
| [OCAR_6276](https://www.genoscope.cns.fr/agc/mage/wwwpkgdb/Info/getInfoLabel.php?id=3483412&wwwpkgdb=fc2733d6468c073f1dd738aa598ede55&nocache=62541e24b6e240f187ac920cab60f9da&dir=&wwwpkgdb=fc2733d6468c073f1dd738aa598ede55) | xth | exodeoxyribonuclease III |
| [OCAR_6281](https://www.genoscope.cns.fr/agc/mage/wwwpkgdb/Info/getInfoLabel.php?id=3485052&wwwpkgdb=fc2733d6468c073f1dd738aa598ede55&nocache=62541e24b6e240f187ac920cab60f9da&dir=&wwwpkgdb=fc2733d6468c073f1dd738aa598ede55) | lpdA | dihydrolipoyl dehydrogenase |
| [OCAR_6285](https://www.genoscope.cns.fr/agc/mage/wwwpkgdb/Info/getInfoLabel.php?id=3485056&wwwpkgdb=fc2733d6468c073f1dd738aa598ede55&nocache=62541e24b6e240f187ac920cab60f9da&dir=&wwwpkgdb=fc2733d6468c073f1dd738aa598ede55) | _ | pyruvate dehydrogenase E1 component subunit beta |
| [OCAR_6286](https://www.genoscope.cns.fr/agc/mage/wwwpkgdb/Info/getInfoLabel.php?id=3485057&wwwpkgdb=fc2733d6468c073f1dd738aa598ede55&nocache=62541e24b6e240f187ac920cab60f9da&dir=&wwwpkgdb=fc2733d6468c073f1dd738aa598ede55) | pdhA | pyruvate dehydrogenase (acetyl-transferring) E1 component, alpha subunit |
| [OCAR_6292](https://www.genoscope.cns.fr/agc/mage/wwwpkgdb/Info/getInfoLabel.php?id=3485062&wwwpkgdb=fc2733d6468c073f1dd738aa598ede55&nocache=62541e24b6e240f187ac920cab60f9da&dir=&wwwpkgdb=fc2733d6468c073f1dd738aa598ede55) | eno | Enolase |
| [OCAR_6293](https://www.genoscope.cns.fr/agc/mage/wwwpkgdb/Info/getInfoLabel.php?id=3483415&wwwpkgdb=fc2733d6468c073f1dd738aa598ede55&nocache=62541e24b6e240f187ac920cab60f9da&dir=&wwwpkgdb=fc2733d6468c073f1dd738aa598ede55) | queF | 7-cyano-7-deazaguanine reductase |
| [OCAR_6296](https://www.genoscope.cns.fr/agc/mage/wwwpkgdb/Info/getInfoLabel.php?id=3485065&wwwpkgdb=fc2733d6468c073f1dd738aa598ede55&nocache=62541e24b6e240f187ac920cab60f9da&dir=&wwwpkgdb=fc2733d6468c073f1dd738aa598ede55) | kdsA | 3-deoxy-8-phosphooctulonate synthase |
| [OCAR_6300](https://www.genoscope.cns.fr/agc/mage/wwwpkgdb/Info/getInfoLabel.php?id=3485069&wwwpkgdb=fc2733d6468c073f1dd738aa598ede55&nocache=62541e24b6e240f187ac920cab60f9da&dir=&wwwpkgdb=fc2733d6468c073f1dd738aa598ede55) | pyrG | CTP synthase |
| [OCAR_6304](https://www.genoscope.cns.fr/agc/mage/wwwpkgdb/Info/getInfoLabel.php?id=3483417&wwwpkgdb=fc2733d6468c073f1dd738aa598ede55&nocache=62541e24b6e240f187ac920cab60f9da&dir=&wwwpkgdb=fc2733d6468c073f1dd738aa598ede55) | trpD | Anthranilate phosphoribosyltransferase |
| [OCAR_6305](https://www.genoscope.cns.fr/agc/mage/wwwpkgdb/Info/getInfoLabel.php?id=3483418&wwwpkgdb=fc2733d6468c073f1dd738aa598ede55&nocache=62541e24b6e240f187ac920cab60f9da&dir=&wwwpkgdb=fc2733d6468c073f1dd738aa598ede55) | trpC | Indole-3-glycerol phosphate synthase |
| [OCAR_6306](https://www.genoscope.cns.fr/agc/mage/wwwpkgdb/Info/getInfoLabel.php?id=3483419&wwwpkgdb=fc2733d6468c073f1dd738aa598ede55&nocache=62541e24b6e240f187ac920cab60f9da&dir=&wwwpkgdb=fc2733d6468c073f1dd738aa598ede55) | moaC | molybdenum cofactor biosynthesis protein C |
| [OCAR_6310](https://www.genoscope.cns.fr/agc/mage/wwwpkgdb/Info/getInfoLabel.php?id=3483421&wwwpkgdb=fc2733d6468c073f1dd738aa598ede55&nocache=62541e24b6e240f187ac920cab60f9da&dir=&wwwpkgdb=fc2733d6468c073f1dd738aa598ede55) | lexA | LexA repressor |
| [OCAR_6312](https://www.genoscope.cns.fr/agc/mage/wwwpkgdb/Info/getInfoLabel.php?id=3485075&wwwpkgdb=fc2733d6468c073f1dd738aa598ede55&nocache=62541e24b6e240f187ac920cab60f9da&dir=&wwwpkgdb=fc2733d6468c073f1dd738aa598ede55) | _ | NADH:ubiquinone oxidoreductase 17.2 kd subunit |
| [OCAR_6327](https://www.genoscope.cns.fr/agc/mage/wwwpkgdb/Info/getInfoLabel.php?id=3485083&wwwpkgdb=fc2733d6468c073f1dd738aa598ede55&nocache=62541e24b6e240f187ac920cab60f9da&dir=&wwwpkgdb=fc2733d6468c073f1dd738aa598ede55) | _ | CreA |
| [OCAR_6333](https://www.genoscope.cns.fr/agc/mage/wwwpkgdb/Info/getInfoLabel.php?id=3485086&wwwpkgdb=fc2733d6468c073f1dd738aa598ede55&nocache=62541e24b6e240f187ac920cab60f9da&dir=&wwwpkgdb=fc2733d6468c073f1dd738aa598ede55) | maeB | NADP-dependent malic enzyme |
| [OCAR_6334](https://www.genoscope.cns.fr/agc/mage/wwwpkgdb/Info/getInfoLabel.php?id=3485087&wwwpkgdb=fc2733d6468c073f1dd738aa598ede55&nocache=62541e24b6e240f187ac920cab60f9da&dir=&wwwpkgdb=fc2733d6468c073f1dd738aa598ede55) | aspS | Aspartyl-tRNA synthetase |
| [OCAR_6337](https://www.genoscope.cns.fr/agc/mage/wwwpkgdb/Info/getInfoLabel.php?id=3483433&wwwpkgdb=fc2733d6468c073f1dd738aa598ede55&nocache=62541e24b6e240f187ac920cab60f9da&dir=&wwwpkgdb=fc2733d6468c073f1dd738aa598ede55) | rnd | ribonuclease D |
| [OCAR_6339](https://www.genoscope.cns.fr/agc/mage/wwwpkgdb/Info/getInfoLabel.php?id=3485090&wwwpkgdb=fc2733d6468c073f1dd738aa598ede55&nocache=62541e24b6e240f187ac920cab60f9da&dir=&wwwpkgdb=fc2733d6468c073f1dd738aa598ede55) | ppk | polyphosphate kinase |
| [OCAR_6340](https://www.genoscope.cns.fr/agc/mage/wwwpkgdb/Info/getInfoLabel.php?id=3485091&wwwpkgdb=fc2733d6468c073f1dd738aa598ede55&nocache=62541e24b6e240f187ac920cab60f9da&dir=&wwwpkgdb=fc2733d6468c073f1dd738aa598ede55) | _ | chromosomal replication initiator, DnaA |
| [OCAR_6341](https://www.genoscope.cns.fr/agc/mage/wwwpkgdb/Info/getInfoLabel.php?id=3485092&wwwpkgdb=fc2733d6468c073f1dd738aa598ede55&nocache=62541e24b6e240f187ac920cab60f9da&dir=&wwwpkgdb=fc2733d6468c073f1dd738aa598ede55) | _ | CDP-alcohol phosphatidyltransferase |
| [OCAR_6343](https://www.genoscope.cns.fr/agc/mage/wwwpkgdb/Info/getInfoLabel.php?id=3483434&wwwpkgdb=fc2733d6468c073f1dd738aa598ede55&nocache=62541e24b6e240f187ac920cab60f9da&dir=&wwwpkgdb=fc2733d6468c073f1dd738aa598ede55) | purM | phosphoribosylformylglycinamidine cyclo-ligase |
| [OCAR_6346](https://www.genoscope.cns.fr/agc/mage/wwwpkgdb/Info/getInfoLabel.php?id=3485095&wwwpkgdb=fc2733d6468c073f1dd738aa598ede55&nocache=62541e24b6e240f187ac920cab60f9da&dir=&wwwpkgdb=fc2733d6468c073f1dd738aa598ede55) | ndk | Nucleoside diphosphate kinase |
| [OCAR_6349](https://www.genoscope.cns.fr/agc/mage/wwwpkgdb/Info/getInfoLabel.php?id=3483438&wwwpkgdb=fc2733d6468c073f1dd738aa598ede55&nocache=62541e24b6e240f187ac920cab60f9da&dir=&wwwpkgdb=fc2733d6468c073f1dd738aa598ede55) | _ | ABC transporter, ATPase subunit |
| [OCAR_6351](https://www.genoscope.cns.fr/agc/mage/wwwpkgdb/Info/getInfoLabel.php?id=3485097&wwwpkgdb=fc2733d6468c073f1dd738aa598ede55&nocache=62541e24b6e240f187ac920cab60f9da&dir=&wwwpkgdb=fc2733d6468c073f1dd738aa598ede55) | _ | DNA polymerase III subunit chi |
| [OCAR_6352](https://www.genoscope.cns.fr/agc/mage/wwwpkgdb/Info/getInfoLabel.php?id=3485098&wwwpkgdb=fc2733d6468c073f1dd738aa598ede55&nocache=62541e24b6e240f187ac920cab60f9da&dir=&wwwpkgdb=fc2733d6468c073f1dd738aa598ede55) | pepA | Probable cytosol aminopeptidase |
| [OCAR_6354](https://www.genoscope.cns.fr/agc/mage/wwwpkgdb/Info/getInfoLabel.php?id=3483440&wwwpkgdb=fc2733d6468c073f1dd738aa598ede55&nocache=62541e24b6e240f187ac920cab60f9da&dir=&wwwpkgdb=fc2733d6468c073f1dd738aa598ede55) | _ | permease YjgP/YjgQ |
| [OCAR_6358](https://www.genoscope.cns.fr/agc/mage/wwwpkgdb/Info/getInfoLabel.php?id=3483444&wwwpkgdb=fc2733d6468c073f1dd738aa598ede55&nocache=62541e24b6e240f187ac920cab60f9da&dir=&wwwpkgdb=fc2733d6468c073f1dd738aa598ede55) | ksgA | Dimethyladenosine transferase |
| [OCAR_6363](https://www.genoscope.cns.fr/agc/mage/wwwpkgdb/Info/getInfoLabel.php?id=3485101&wwwpkgdb=fc2733d6468c073f1dd738aa598ede55&nocache=62541e24b6e240f187ac920cab60f9da&dir=&wwwpkgdb=fc2733d6468c073f1dd738aa598ede55) | gmk | guanylate kinase |
| [OCAR_6364](https://www.genoscope.cns.fr/agc/mage/wwwpkgdb/Info/getInfoLabel.php?id=3485102&wwwpkgdb=fc2733d6468c073f1dd738aa598ede55&nocache=62541e24b6e240f187ac920cab60f9da&dir=&wwwpkgdb=fc2733d6468c073f1dd738aa598ede55) | _ | hypothetical protein |
| [OCAR_6365](https://www.genoscope.cns.fr/agc/mage/wwwpkgdb/Info/getInfoLabel.php?id=3485103&wwwpkgdb=fc2733d6468c073f1dd738aa598ede55&nocache=62541e24b6e240f187ac920cab60f9da&dir=&wwwpkgdb=fc2733d6468c073f1dd738aa598ede55) | _ | hypothetical protein |
| [OCAR_6366](https://www.genoscope.cns.fr/agc/mage/wwwpkgdb/Info/getInfoLabel.php?id=3485104&wwwpkgdb=fc2733d6468c073f1dd738aa598ede55&nocache=62541e24b6e240f187ac920cab60f9da&dir=&wwwpkgdb=fc2733d6468c073f1dd738aa598ede55) | fabF | 3-oxoacyl-[acyl-carrier-protein] synthase 2 |
| [OCAR_6367](https://www.genoscope.cns.fr/agc/mage/wwwpkgdb/Info/getInfoLabel.php?id=3485105&wwwpkgdb=fc2733d6468c073f1dd738aa598ede55&nocache=62541e24b6e240f187ac920cab60f9da&dir=&wwwpkgdb=fc2733d6468c073f1dd738aa598ede55) | acpP | Acyl carrier protein |
| [OCAR_6369](https://www.genoscope.cns.fr/agc/mage/wwwpkgdb/Info/getInfoLabel.php?id=3485106&wwwpkgdb=fc2733d6468c073f1dd738aa598ede55&nocache=62541e24b6e240f187ac920cab60f9da&dir=&wwwpkgdb=fc2733d6468c073f1dd738aa598ede55) | fabG | 3-oxoacyl-(acyl-carrier-protein) reductase |
| [OCAR_6370](https://www.genoscope.cns.fr/agc/mage/wwwpkgdb/Info/getInfoLabel.php?id=3485107&wwwpkgdb=fc2733d6468c073f1dd738aa598ede55&nocache=62541e24b6e240f187ac920cab60f9da&dir=&wwwpkgdb=fc2733d6468c073f1dd738aa598ede55) | fabD | malonyl CoA-acyl carrier protein transacylase |
| [OCAR_6372](https://www.genoscope.cns.fr/agc/mage/wwwpkgdb/Info/getInfoLabel.php?id=3483449&wwwpkgdb=fc2733d6468c073f1dd738aa598ede55&nocache=62541e24b6e240f187ac920cab60f9da&dir=&wwwpkgdb=fc2733d6468c073f1dd738aa598ede55) | rpsR | 30S ribosomal protein S18 |
| [OCAR_6374](https://www.genoscope.cns.fr/agc/mage/wwwpkgdb/Info/getInfoLabel.php?id=3483451&wwwpkgdb=fc2733d6468c073f1dd738aa598ede55&nocache=62541e24b6e240f187ac920cab60f9da&dir=&wwwpkgdb=fc2733d6468c073f1dd738aa598ede55) | rplI | 50S ribosomal protein L9 |
| [OCAR_6377](https://www.genoscope.cns.fr/agc/mage/wwwpkgdb/Info/getInfoLabel.php?id=3483453&wwwpkgdb=fc2733d6468c073f1dd738aa598ede55&nocache=62541e24b6e240f187ac920cab60f9da&dir=&wwwpkgdb=fc2733d6468c073f1dd738aa598ede55) | dnaB | replicative DNA helicase |
| [OCAR_6379](https://www.genoscope.cns.fr/agc/mage/wwwpkgdb/Info/getInfoLabel.php?id=3483455&wwwpkgdb=fc2733d6468c073f1dd738aa598ede55&nocache=62541e24b6e240f187ac920cab60f9da&dir=&wwwpkgdb=fc2733d6468c073f1dd738aa598ede55) | radA | DNA repair protein RadA |
| [OCAR_6382](https://www.genoscope.cns.fr/agc/mage/wwwpkgdb/Info/getInfoLabel.php?id=3483457&wwwpkgdb=fc2733d6468c073f1dd738aa598ede55&nocache=62541e24b6e240f187ac920cab60f9da&dir=&wwwpkgdb=fc2733d6468c073f1dd738aa598ede55) | purF | amidophosphoribosyltransferase |
| [OCAR_6384](https://www.genoscope.cns.fr/agc/mage/wwwpkgdb/Info/getInfoLabel.php?id=3483458&wwwpkgdb=fc2733d6468c073f1dd738aa598ede55&nocache=62541e24b6e240f187ac920cab60f9da&dir=&wwwpkgdb=fc2733d6468c073f1dd738aa598ede55) | _ | short-chain dehydrogenase/reductase SDR |
| [OCAR_6385](https://www.genoscope.cns.fr/agc/mage/wwwpkgdb/Info/getInfoLabel.php?id=3485111&wwwpkgdb=fc2733d6468c073f1dd738aa598ede55&nocache=62541e24b6e240f187ac920cab60f9da&dir=&wwwpkgdb=fc2733d6468c073f1dd738aa598ede55) | _ | small GTP-binding protein domain |
| [OCAR_6388](https://www.genoscope.cns.fr/agc/mage/wwwpkgdb/Info/getInfoLabel.php?id=3485114&wwwpkgdb=fc2733d6468c073f1dd738aa598ede55&nocache=62541e24b6e240f187ac920cab60f9da&dir=&wwwpkgdb=fc2733d6468c073f1dd738aa598ede55) | panB | 3-methyl-2-oxobutanoate hydroxymethyltransferase |
| [OCAR_6390](https://www.genoscope.cns.fr/agc/mage/wwwpkgdb/Info/getInfoLabel.php?id=3485116&wwwpkgdb=fc2733d6468c073f1dd738aa598ede55&nocache=62541e24b6e240f187ac920cab60f9da&dir=&wwwpkgdb=fc2733d6468c073f1dd738aa598ede55) | prfC | peptide chain release factor 3 |
| [OCAR_6391](https://www.genoscope.cns.fr/agc/mage/wwwpkgdb/Info/getInfoLabel.php?id=3483459&wwwpkgdb=fc2733d6468c073f1dd738aa598ede55&nocache=62541e24b6e240f187ac920cab60f9da&dir=&wwwpkgdb=fc2733d6468c073f1dd738aa598ede55) | _ | small multidrug resistance protein |
| [OCAR_6392](https://www.genoscope.cns.fr/agc/mage/wwwpkgdb/Info/getInfoLabel.php?id=3485117&wwwpkgdb=fc2733d6468c073f1dd738aa598ede55&nocache=62541e24b6e240f187ac920cab60f9da&dir=&wwwpkgdb=fc2733d6468c073f1dd738aa598ede55) | _ | inner-membrane translocator |
| [OCAR_6393](https://www.genoscope.cns.fr/agc/mage/wwwpkgdb/Info/getInfoLabel.php?id=3485118&wwwpkgdb=fc2733d6468c073f1dd738aa598ede55&nocache=62541e24b6e240f187ac920cab60f9da&dir=&wwwpkgdb=fc2733d6468c073f1dd738aa598ede55) | _ | inner-membrane translocator |
| [OCAR_6394](https://www.genoscope.cns.fr/agc/mage/wwwpkgdb/Info/getInfoLabel.php?id=3485119&wwwpkgdb=fc2733d6468c073f1dd738aa598ede55&nocache=62541e24b6e240f187ac920cab60f9da&dir=&wwwpkgdb=fc2733d6468c073f1dd738aa598ede55) | _ | ABC transporter ATP-binding protein |
| [OCAR_6395](https://www.genoscope.cns.fr/agc/mage/wwwpkgdb/Info/getInfoLabel.php?id=3485120&wwwpkgdb=fc2733d6468c073f1dd738aa598ede55&nocache=62541e24b6e240f187ac920cab60f9da&dir=&wwwpkgdb=fc2733d6468c073f1dd738aa598ede55) | _ | ABC transporter ATP-binding protein |
| [OCAR_6437](https://www.genoscope.cns.fr/agc/mage/wwwpkgdb/Info/getInfoLabel.php?id=3485153&wwwpkgdb=fc2733d6468c073f1dd738aa598ede55&nocache=62541e24b6e240f187ac920cab60f9da&dir=&wwwpkgdb=fc2733d6468c073f1dd738aa598ede55) | _ | cation efflux system protein CzcA |
| [OCAR_6439](https://www.genoscope.cns.fr/agc/mage/wwwpkgdb/Info/getInfoLabel.php?id=3485155&wwwpkgdb=fc2733d6468c073f1dd738aa598ede55&nocache=62541e24b6e240f187ac920cab60f9da&dir=&wwwpkgdb=fc2733d6468c073f1dd738aa598ede55) | _ | outer membrane efflux protein |
| [OCAR_6467](https://www.genoscope.cns.fr/agc/mage/wwwpkgdb/Info/getInfoLabel.php?id=3485177&wwwpkgdb=fc2733d6468c073f1dd738aa598ede55&nocache=62541e24b6e240f187ac920cab60f9da&dir=&wwwpkgdb=fc2733d6468c073f1dd738aa598ede55) | guaA | GMP synthase [glutamine-hydrolyzing] |
| [OCAR_6468](https://www.genoscope.cns.fr/agc/mage/wwwpkgdb/Info/getInfoLabel.php?id=3485178&wwwpkgdb=fc2733d6468c073f1dd738aa598ede55&nocache=62541e24b6e240f187ac920cab60f9da&dir=&wwwpkgdb=fc2733d6468c073f1dd738aa598ede55) | _ | Fmu |
| [OCAR_6470](https://www.genoscope.cns.fr/agc/mage/wwwpkgdb/Info/getInfoLabel.php?id=3485180&wwwpkgdb=fc2733d6468c073f1dd738aa598ede55&nocache=62541e24b6e240f187ac920cab60f9da&dir=&wwwpkgdb=fc2733d6468c073f1dd738aa598ede55) | guaB | inosine-5'-monophosphate dehydrogenase |
| [OCAR_6471](https://www.genoscope.cns.fr/agc/mage/wwwpkgdb/Info/getInfoLabel.php?id=3483475&wwwpkgdb=fc2733d6468c073f1dd738aa598ede55&nocache=62541e24b6e240f187ac920cab60f9da&dir=&wwwpkgdb=fc2733d6468c073f1dd738aa598ede55) | _ | inner membrane transport protein YieO |
| [OCAR_6472](https://www.genoscope.cns.fr/agc/mage/wwwpkgdb/Info/getInfoLabel.php?id=3485181&wwwpkgdb=fc2733d6468c073f1dd738aa598ede55&nocache=62541e24b6e240f187ac920cab60f9da&dir=&wwwpkgdb=fc2733d6468c073f1dd738aa598ede55) | rrmJ | Ribosomal RNA large subunit methyltransferase J |
| [OCAR_6475](https://www.genoscope.cns.fr/agc/mage/wwwpkgdb/Info/getInfoLabel.php?id=3483477&wwwpkgdb=fc2733d6468c073f1dd738aa598ede55&nocache=62541e24b6e240f187ac920cab60f9da&dir=&wwwpkgdb=fc2733d6468c073f1dd738aa598ede55) | _ | hypothetical protein |
| [OCAR_6476](https://www.genoscope.cns.fr/agc/mage/wwwpkgdb/Info/getInfoLabel.php?id=3483478&wwwpkgdb=fc2733d6468c073f1dd738aa598ede55&nocache=62541e24b6e240f187ac920cab60f9da&dir=&wwwpkgdb=fc2733d6468c073f1dd738aa598ede55) | _ | holdfast attachment protein C (Protein HfaC) |
| [OCAR_6482](https://www.genoscope.cns.fr/agc/mage/wwwpkgdb/Info/getInfoLabel.php?id=3485186&wwwpkgdb=fc2733d6468c073f1dd738aa598ede55&nocache=62541e24b6e240f187ac920cab60f9da&dir=&wwwpkgdb=fc2733d6468c073f1dd738aa598ede55) | mmsB | 3-hydroxyisobutyrate dehydrogenase |
| [OCAR_6485](https://www.genoscope.cns.fr/agc/mage/wwwpkgdb/Info/getInfoLabel.php?id=3485187&wwwpkgdb=fc2733d6468c073f1dd738aa598ede55&nocache=62541e24b6e240f187ac920cab60f9da&dir=&wwwpkgdb=fc2733d6468c073f1dd738aa598ede55) | _ | multidrug resistance protein MdtB; Multidrug transporter mdtB |
| [OCAR_6496](https://www.genoscope.cns.fr/agc/mage/wwwpkgdb/Info/getInfoLabel.php?id=3483485&wwwpkgdb=fc2733d6468c073f1dd738aa598ede55&nocache=62541e24b6e240f187ac920cab60f9da&dir=&wwwpkgdb=fc2733d6468c073f1dd738aa598ede55) | _ | protein of unknown function DUF87 |
| [OCAR_6498](https://www.genoscope.cns.fr/agc/mage/wwwpkgdb/Info/getInfoLabel.php?id=3483487&wwwpkgdb=fc2733d6468c073f1dd738aa598ede55&nocache=62541e24b6e240f187ac920cab60f9da&dir=&wwwpkgdb=fc2733d6468c073f1dd738aa598ede55) | _ | acyl carrier protein |
| [OCAR_6500](https://www.genoscope.cns.fr/agc/mage/wwwpkgdb/Info/getInfoLabel.php?id=3483489&wwwpkgdb=fc2733d6468c073f1dd738aa598ede55&nocache=62541e24b6e240f187ac920cab60f9da&dir=&wwwpkgdb=fc2733d6468c073f1dd738aa598ede55) | _ | beta-ketoacyl synthase |
| [OCAR_6501](https://www.genoscope.cns.fr/agc/mage/wwwpkgdb/Info/getInfoLabel.php?id=3483490&wwwpkgdb=fc2733d6468c073f1dd738aa598ede55&nocache=62541e24b6e240f187ac920cab60f9da&dir=&wwwpkgdb=fc2733d6468c073f1dd738aa598ede55) | _ | 3-oxoacyl-[acyl-carrier-protein] synthase 2 |
| [OCAR_6504](https://www.genoscope.cns.fr/agc/mage/wwwpkgdb/Info/getInfoLabel.php?id=3485196&wwwpkgdb=fc2733d6468c073f1dd738aa598ede55&nocache=62541e24b6e240f187ac920cab60f9da&dir=&wwwpkgdb=fc2733d6468c073f1dd738aa598ede55) | _ | putrescine-binding periplasmic protein |
| [OCAR_6505](https://www.genoscope.cns.fr/agc/mage/wwwpkgdb/Info/getInfoLabel.php?id=3485197&wwwpkgdb=fc2733d6468c073f1dd738aa598ede55&nocache=62541e24b6e240f187ac920cab60f9da&dir=&wwwpkgdb=fc2733d6468c073f1dd738aa598ede55) | _ | aminotransferase YbdL |
| [OCAR_6507](https://www.genoscope.cns.fr/agc/mage/wwwpkgdb/Info/getInfoLabel.php?id=3485199&wwwpkgdb=fc2733d6468c073f1dd738aa598ede55&nocache=62541e24b6e240f187ac920cab60f9da&dir=&wwwpkgdb=fc2733d6468c073f1dd738aa598ede55) | _ | low affinity potassium transporter |
| [OCAR_6508](https://www.genoscope.cns.fr/agc/mage/wwwpkgdb/Info/getInfoLabel.php?id=3485200&wwwpkgdb=fc2733d6468c073f1dd738aa598ede55&nocache=62541e24b6e240f187ac920cab60f9da&dir=&wwwpkgdb=fc2733d6468c073f1dd738aa598ede55) | _ | OmpA/MotB |
| [OCAR_6509](https://www.genoscope.cns.fr/agc/mage/wwwpkgdb/Info/getInfoLabel.php?id=3485201&wwwpkgdb=fc2733d6468c073f1dd738aa598ede55&nocache=62541e24b6e240f187ac920cab60f9da&dir=&wwwpkgdb=fc2733d6468c073f1dd738aa598ede55) | _ | chemotaxis protein PomA |
| [OCAR_6512](https://www.genoscope.cns.fr/agc/mage/wwwpkgdb/Info/getInfoLabel.php?id=3485204&wwwpkgdb=fc2733d6468c073f1dd738aa598ede55&nocache=62541e24b6e240f187ac920cab60f9da&dir=&wwwpkgdb=fc2733d6468c073f1dd738aa598ede55) | psd | Phosphatidylserine decarboxylase proenzyme |
| [OCAR_6514](https://www.genoscope.cns.fr/agc/mage/wwwpkgdb/Info/getInfoLabel.php?id=3485206&wwwpkgdb=fc2733d6468c073f1dd738aa598ede55&nocache=62541e24b6e240f187ac920cab60f9da&dir=&wwwpkgdb=fc2733d6468c073f1dd738aa598ede55) | _ | ABC transporter, transmembrane region |
| [OCAR_6516](https://www.genoscope.cns.fr/agc/mage/wwwpkgdb/Info/getInfoLabel.php?id=3485207&wwwpkgdb=fc2733d6468c073f1dd738aa598ede55&nocache=62541e24b6e240f187ac920cab60f9da&dir=&wwwpkgdb=fc2733d6468c073f1dd738aa598ede55) | _ | 2-isopropylmalate synthase/homocitrate synthase family protein |
| [OCAR_6522](https://www.genoscope.cns.fr/agc/mage/wwwpkgdb/Info/getInfoLabel.php?id=3485211&wwwpkgdb=fc2733d6468c073f1dd738aa598ede55&nocache=62541e24b6e240f187ac920cab60f9da&dir=&wwwpkgdb=fc2733d6468c073f1dd738aa598ede55) | _ | glutamine-dependent NAD(+) synthetase |
| [OCAR_6523](https://www.genoscope.cns.fr/agc/mage/wwwpkgdb/Info/getInfoLabel.php?id=3485212&wwwpkgdb=fc2733d6468c073f1dd738aa598ede55&nocache=62541e24b6e240f187ac920cab60f9da&dir=&wwwpkgdb=fc2733d6468c073f1dd738aa598ede55) | _ | 3-deoxy-7-phosphoheptulonate synthase |
| [OCAR_6527](https://www.genoscope.cns.fr/agc/mage/wwwpkgdb/Info/getInfoLabel.php?id=3485215&wwwpkgdb=fc2733d6468c073f1dd738aa598ede55&nocache=62541e24b6e240f187ac920cab60f9da&dir=&wwwpkgdb=fc2733d6468c073f1dd738aa598ede55) | gor | glutathione-disulfide reductase |
| [OCAR_6529](https://www.genoscope.cns.fr/agc/mage/wwwpkgdb/Info/getInfoLabel.php?id=3485217&wwwpkgdb=fc2733d6468c073f1dd738aa598ede55&nocache=62541e24b6e240f187ac920cab60f9da&dir=&wwwpkgdb=fc2733d6468c073f1dd738aa598ede55) | rpiA | Ribose-5-phosphate isomerase A |
| [OCAR_6532](https://www.genoscope.cns.fr/agc/mage/wwwpkgdb/Info/getInfoLabel.php?id=3483499&wwwpkgdb=fc2733d6468c073f1dd738aa598ede55&nocache=62541e24b6e240f187ac920cab60f9da&dir=&wwwpkgdb=fc2733d6468c073f1dd738aa598ede55) | moaA | molybdenum cofactor biosynthesis protein A |
| [OCAR_6535](https://www.genoscope.cns.fr/agc/mage/wwwpkgdb/Info/getInfoLabel.php?id=3485218&wwwpkgdb=fc2733d6468c073f1dd738aa598ede55&nocache=62541e24b6e240f187ac920cab60f9da&dir=&wwwpkgdb=fc2733d6468c073f1dd738aa598ede55) | _ | twin-arginine translocation pathway signal |
| [OCAR_6537](https://www.genoscope.cns.fr/agc/mage/wwwpkgdb/Info/getInfoLabel.php?id=3485219&wwwpkgdb=fc2733d6468c073f1dd738aa598ede55&nocache=62541e24b6e240f187ac920cab60f9da&dir=&wwwpkgdb=fc2733d6468c073f1dd738aa598ede55) | _ | periplasmic mannitol-binding protein |
| [OCAR_6539](https://www.genoscope.cns.fr/agc/mage/wwwpkgdb/Info/getInfoLabel.php?id=3483503&wwwpkgdb=fc2733d6468c073f1dd738aa598ede55&nocache=62541e24b6e240f187ac920cab60f9da&dir=&wwwpkgdb=fc2733d6468c073f1dd738aa598ede55) | _ | hypothetical protein |
| [OCAR_6540](https://www.genoscope.cns.fr/agc/mage/wwwpkgdb/Info/getInfoLabel.php?id=3483504&wwwpkgdb=fc2733d6468c073f1dd738aa598ede55&nocache=62541e24b6e240f187ac920cab60f9da&dir=&wwwpkgdb=fc2733d6468c073f1dd738aa598ede55) | _ | phosphate transporter |
| [OCAR_6542](https://www.genoscope.cns.fr/agc/mage/wwwpkgdb/Info/getInfoLabel.php?id=3483506&wwwpkgdb=fc2733d6468c073f1dd738aa598ede55&nocache=62541e24b6e240f187ac920cab60f9da&dir=&wwwpkgdb=fc2733d6468c073f1dd738aa598ede55) | _ | periplasmic sensor signal transduction histidine kinase |
| [OCAR_6545](https://www.genoscope.cns.fr/agc/mage/wwwpkgdb/Info/getInfoLabel.php?id=3485221&wwwpkgdb=fc2733d6468c073f1dd738aa598ede55&nocache=62541e24b6e240f187ac920cab60f9da&dir=&wwwpkgdb=fc2733d6468c073f1dd738aa598ede55) | _ | tRNA-dihydrouridine synthase A |
| [OCAR_6554](https://www.genoscope.cns.fr/agc/mage/wwwpkgdb/Info/getInfoLabel.php?id=3483514&wwwpkgdb=fc2733d6468c073f1dd738aa598ede55&nocache=62541e24b6e240f187ac920cab60f9da&dir=&wwwpkgdb=fc2733d6468c073f1dd738aa598ede55) | sucC | Succinyl-CoA ligase [ADP-forming] subunit beta |
| [OCAR_6555](https://www.genoscope.cns.fr/agc/mage/wwwpkgdb/Info/getInfoLabel.php?id=3483515&wwwpkgdb=fc2733d6468c073f1dd738aa598ede55&nocache=62541e24b6e240f187ac920cab60f9da&dir=&wwwpkgdb=fc2733d6468c073f1dd738aa598ede55) | _ | succinyl-CoA ligase |
| [OCAR_6569](https://www.genoscope.cns.fr/agc/mage/wwwpkgdb/Info/getInfoLabel.php?id=3485234&wwwpkgdb=fc2733d6468c073f1dd738aa598ede55&nocache=62541e24b6e240f187ac920cab60f9da&dir=&wwwpkgdb=fc2733d6468c073f1dd738aa598ede55) | _ | glutamate-ammonia-ligase adenylyltransferase |
| [OCAR_6570](https://www.genoscope.cns.fr/agc/mage/wwwpkgdb/Info/getInfoLabel.php?id=3485235&wwwpkgdb=fc2733d6468c073f1dd738aa598ede55&nocache=62541e24b6e240f187ac920cab60f9da&dir=&wwwpkgdb=fc2733d6468c073f1dd738aa598ede55) | _ | periplasmic sensor signal transduction histidine kinase |
| [OCAR_6571](https://www.genoscope.cns.fr/agc/mage/wwwpkgdb/Info/getInfoLabel.php?id=3485236&wwwpkgdb=fc2733d6468c073f1dd738aa598ede55&nocache=62541e24b6e240f187ac920cab60f9da&dir=&wwwpkgdb=fc2733d6468c073f1dd738aa598ede55) | _ | transcriptional activator protein CopR |
| [OCAR_6572](https://www.genoscope.cns.fr/agc/mage/wwwpkgdb/Info/getInfoLabel.php?id=3485237&wwwpkgdb=fc2733d6468c073f1dd738aa598ede55&nocache=62541e24b6e240f187ac920cab60f9da&dir=&wwwpkgdb=fc2733d6468c073f1dd738aa598ede55) | _ | protease Do subfamily |
| [OCAR_6574](https://www.genoscope.cns.fr/agc/mage/wwwpkgdb/Info/getInfoLabel.php?id=3485239&wwwpkgdb=fc2733d6468c073f1dd738aa598ede55&nocache=62541e24b6e240f187ac920cab60f9da&dir=&wwwpkgdb=fc2733d6468c073f1dd738aa598ede55) | ccmF | cytochrome c-type biogenesis protein CcmF |
| [OCAR_6575](https://www.genoscope.cns.fr/agc/mage/wwwpkgdb/Info/getInfoLabel.php?id=3485240&wwwpkgdb=fc2733d6468c073f1dd738aa598ede55&nocache=62541e24b6e240f187ac920cab60f9da&dir=&wwwpkgdb=fc2733d6468c073f1dd738aa598ede55) | _ | cytochrome c-type biogenesis protein CycJ |
| [OCAR_6578](https://www.genoscope.cns.fr/agc/mage/wwwpkgdb/Info/getInfoLabel.php?id=3485243&wwwpkgdb=fc2733d6468c073f1dd738aa598ede55&nocache=62541e24b6e240f187ac920cab60f9da&dir=&wwwpkgdb=fc2733d6468c073f1dd738aa598ede55) | _ | two-component sensor histidine kinase |
| [OCAR_6579](https://www.genoscope.cns.fr/agc/mage/wwwpkgdb/Info/getInfoLabel.php?id=3485244&wwwpkgdb=fc2733d6468c073f1dd738aa598ede55&nocache=62541e24b6e240f187ac920cab60f9da&dir=&wwwpkgdb=fc2733d6468c073f1dd738aa598ede55) | _ | transcriptional regulatory protein QseB |
| [OCAR_6616](https://www.genoscope.cns.fr/agc/mage/wwwpkgdb/Info/getInfoLabel.php?id=3485277&wwwpkgdb=fc2733d6468c073f1dd738aa598ede55&nocache=62541e24b6e240f187ac920cab60f9da&dir=&wwwpkgdb=fc2733d6468c073f1dd738aa598ede55) | _ | UPF0260 protein OCAR_6616 |
| [OCAR_6617](https://www.genoscope.cns.fr/agc/mage/wwwpkgdb/Info/getInfoLabel.php?id=3483524&wwwpkgdb=fc2733d6468c073f1dd738aa598ede55&nocache=62541e24b6e240f187ac920cab60f9da&dir=&wwwpkgdb=fc2733d6468c073f1dd738aa598ede55) | _ | penicillin-binding protein, 1A family |
| [OCAR_6619](https://www.genoscope.cns.fr/agc/mage/wwwpkgdb/Info/getInfoLabel.php?id=3483526&wwwpkgdb=fc2733d6468c073f1dd738aa598ede55&nocache=62541e24b6e240f187ac920cab60f9da&dir=&wwwpkgdb=fc2733d6468c073f1dd738aa598ede55) | _ | hypothetical protein |
| [OCAR_6632](https://www.genoscope.cns.fr/agc/mage/wwwpkgdb/Info/getInfoLabel.php?id=3483531&wwwpkgdb=fc2733d6468c073f1dd738aa598ede55&nocache=62541e24b6e240f187ac920cab60f9da&dir=&wwwpkgdb=fc2733d6468c073f1dd738aa598ede55) | _ | cysteine desulfuration protein SufE |
| [OCAR_6639](https://www.genoscope.cns.fr/agc/mage/wwwpkgdb/Info/getInfoLabel.php?id=3485290&wwwpkgdb=fc2733d6468c073f1dd738aa598ede55&nocache=62541e24b6e240f187ac920cab60f9da&dir=&wwwpkgdb=fc2733d6468c073f1dd738aa598ede55) | _ | peptidase dimerisation |
| [OCAR_6640](https://www.genoscope.cns.fr/agc/mage/wwwpkgdb/Info/getInfoLabel.php?id=3485291&wwwpkgdb=fc2733d6468c073f1dd738aa598ede55&nocache=62541e24b6e240f187ac920cab60f9da&dir=&wwwpkgdb=fc2733d6468c073f1dd738aa598ede55) | _ | ATPase |
| [OCAR_6643](https://www.genoscope.cns.fr/agc/mage/wwwpkgdb/Info/getInfoLabel.php?id=3483535&wwwpkgdb=fc2733d6468c073f1dd738aa598ede55&nocache=62541e24b6e240f187ac920cab60f9da&dir=&wwwpkgdb=fc2733d6468c073f1dd738aa598ede55) | folB | dihydroneopterin aldolase |
| [OCAR_6644](https://www.genoscope.cns.fr/agc/mage/wwwpkgdb/Info/getInfoLabel.php?id=3483536&wwwpkgdb=fc2733d6468c073f1dd738aa598ede55&nocache=62541e24b6e240f187ac920cab60f9da&dir=&wwwpkgdb=fc2733d6468c073f1dd738aa598ede55) | folK | 2-amino-4-hydroxy-6- hydroxymethyldihydropteridine pyrophosphokinase |
| [OCAR_6658](https://www.genoscope.cns.fr/agc/mage/wwwpkgdb/Info/getInfoLabel.php?id=3485297&wwwpkgdb=fc2733d6468c073f1dd738aa598ede55&nocache=62541e24b6e240f187ac920cab60f9da&dir=&wwwpkgdb=fc2733d6468c073f1dd738aa598ede55) | _ | cation efflux system protein CzcA |
| [OCAR_6660](https://www.genoscope.cns.fr/agc/mage/wwwpkgdb/Info/getInfoLabel.php?id=3485299&wwwpkgdb=fc2733d6468c073f1dd738aa598ede55&nocache=62541e24b6e240f187ac920cab60f9da&dir=&wwwpkgdb=fc2733d6468c073f1dd738aa598ede55) | _ | hippuricase |
| [OCAR_6663](https://www.genoscope.cns.fr/agc/mage/wwwpkgdb/Info/getInfoLabel.php?id=3485300&wwwpkgdb=fc2733d6468c073f1dd738aa598ede55&nocache=62541e24b6e240f187ac920cab60f9da&dir=&wwwpkgdb=fc2733d6468c073f1dd738aa598ede55) | _ | aminotransferase class-III |
| [OCAR_6666](https://www.genoscope.cns.fr/agc/mage/wwwpkgdb/Info/getInfoLabel.php?id=3483548&wwwpkgdb=fc2733d6468c073f1dd738aa598ede55&nocache=62541e24b6e240f187ac920cab60f9da&dir=&wwwpkgdb=fc2733d6468c073f1dd738aa598ede55) | dxs | 1-deoxy-D-xylulose-5-phosphate synthase |
| [OCAR_6667](https://www.genoscope.cns.fr/agc/mage/wwwpkgdb/Info/getInfoLabel.php?id=3483549&wwwpkgdb=fc2733d6468c073f1dd738aa598ede55&nocache=62541e24b6e240f187ac920cab60f9da&dir=&wwwpkgdb=fc2733d6468c073f1dd738aa598ede55) | ispH | 4-hydroxy-3-methylbut-2-enyl diphosphate reductase |
| [OCAR_6668](https://www.genoscope.cns.fr/agc/mage/wwwpkgdb/Info/getInfoLabel.php?id=3483550&wwwpkgdb=fc2733d6468c073f1dd738aa598ede55&nocache=62541e24b6e240f187ac920cab60f9da&dir=&wwwpkgdb=fc2733d6468c073f1dd738aa598ede55) | hpnH | hopanoid biosynthesis associated radical SAM protein HpnH |
| [OCAR_6684](https://www.genoscope.cns.fr/agc/mage/wwwpkgdb/Info/getInfoLabel.php?id=3485314&wwwpkgdb=fc2733d6468c073f1dd738aa598ede55&nocache=62541e24b6e240f187ac920cab60f9da&dir=&wwwpkgdb=fc2733d6468c073f1dd738aa598ede55) | _ | acriflavin resistance protein |
| [OCAR_6716](https://www.genoscope.cns.fr/agc/mage/wwwpkgdb/Info/getInfoLabel.php?id=3485334&wwwpkgdb=fc2733d6468c073f1dd738aa598ede55&nocache=62541e24b6e240f187ac920cab60f9da&dir=&wwwpkgdb=fc2733d6468c073f1dd738aa598ede55) | _ | molybdenum cofactor synthesis domain |
| [OCAR_6719](https://www.genoscope.cns.fr/agc/mage/wwwpkgdb/Info/getInfoLabel.php?id=3483566&wwwpkgdb=fc2733d6468c073f1dd738aa598ede55&nocache=62541e24b6e240f187ac920cab60f9da&dir=&wwwpkgdb=fc2733d6468c073f1dd738aa598ede55) | _ | Ku protein |
| [OCAR_6723](https://www.genoscope.cns.fr/agc/mage/wwwpkgdb/Info/getInfoLabel.php?id=3485340&wwwpkgdb=fc2733d6468c073f1dd738aa598ede55&nocache=62541e24b6e240f187ac920cab60f9da&dir=&wwwpkgdb=fc2733d6468c073f1dd738aa598ede55) | _ | PKHD-type hydroxylase OCAR_6723 |
| [OCAR_6725](https://www.genoscope.cns.fr/agc/mage/wwwpkgdb/Info/getInfoLabel.php?id=3485342&wwwpkgdb=fc2733d6468c073f1dd738aa598ede55&nocache=62541e24b6e240f187ac920cab60f9da&dir=&wwwpkgdb=fc2733d6468c073f1dd738aa598ede55) | _ | hypothetical protein |
| [OCAR_6751](https://www.genoscope.cns.fr/agc/mage/wwwpkgdb/Info/getInfoLabel.php?id=3485356&wwwpkgdb=fc2733d6468c073f1dd738aa598ede55&nocache=62541e24b6e240f187ac920cab60f9da&dir=&wwwpkgdb=fc2733d6468c073f1dd738aa598ede55) | metE | 5-methyltetrahydropteroyltriglutamate-- homocysteine S-methyltransferase |
| [OCAR_6765](https://www.genoscope.cns.fr/agc/mage/wwwpkgdb/Info/getInfoLabel.php?id=3485362&wwwpkgdb=fc2733d6468c073f1dd738aa598ede55&nocache=62541e24b6e240f187ac920cab60f9da&dir=&wwwpkgdb=fc2733d6468c073f1dd738aa598ede55) | _ | B12-dependent ribonucleoside diphosphate reductase |
| [OCAR_6826](https://www.genoscope.cns.fr/agc/mage/wwwpkgdb/Info/getInfoLabel.php?id=3485399&wwwpkgdb=fc2733d6468c073f1dd738aa598ede55&nocache=62541e24b6e240f187ac920cab60f9da&dir=&wwwpkgdb=fc2733d6468c073f1dd738aa598ede55) | _ | alcohol dehydrogenase, zinc-containing |
| [OCAR_6830](https://www.genoscope.cns.fr/agc/mage/wwwpkgdb/Info/getInfoLabel.php?id=3483613&wwwpkgdb=fc2733d6468c073f1dd738aa598ede55&nocache=62541e24b6e240f187ac920cab60f9da&dir=&wwwpkgdb=fc2733d6468c073f1dd738aa598ede55) | _ | ABC transporter ATP-binding protein |
| [OCAR_6839](https://www.genoscope.cns.fr/agc/mage/wwwpkgdb/Info/getInfoLabel.php?id=3485408&wwwpkgdb=fc2733d6468c073f1dd738aa598ede55&nocache=62541e24b6e240f187ac920cab60f9da&dir=&wwwpkgdb=fc2733d6468c073f1dd738aa598ede55) | _ | hypothetical protein |
| [OCAR_6894](https://www.genoscope.cns.fr/agc/mage/wwwpkgdb/Info/getInfoLabel.php?id=3483635&wwwpkgdb=fc2733d6468c073f1dd738aa598ede55&nocache=62541e24b6e240f187ac920cab60f9da&dir=&wwwpkgdb=fc2733d6468c073f1dd738aa598ede55) | xseB | Exodeoxyribonuclease 7 small subunit |
| [OCAR_6895](https://www.genoscope.cns.fr/agc/mage/wwwpkgdb/Info/getInfoLabel.php?id=3483636&wwwpkgdb=fc2733d6468c073f1dd738aa598ede55&nocache=62541e24b6e240f187ac920cab60f9da&dir=&wwwpkgdb=fc2733d6468c073f1dd738aa598ede55) | dxs | 1-deoxy-D-xylulose-5-phosphate synthase |
| [OCAR_6896](https://www.genoscope.cns.fr/agc/mage/wwwpkgdb/Info/getInfoLabel.php?id=3483637&wwwpkgdb=fc2733d6468c073f1dd738aa598ede55&nocache=62541e24b6e240f187ac920cab60f9da&dir=&wwwpkgdb=fc2733d6468c073f1dd738aa598ede55) | rrmJ | ribosomal RNA large subunit methyltransferase J |
| [OCAR_6901](https://www.genoscope.cns.fr/agc/mage/wwwpkgdb/Info/getInfoLabel.php?id=3485444&wwwpkgdb=fc2733d6468c073f1dd738aa598ede55&nocache=62541e24b6e240f187ac920cab60f9da&dir=&wwwpkgdb=fc2733d6468c073f1dd738aa598ede55) | aroC | Chorismate synthase |
| [OCAR_6908](https://www.genoscope.cns.fr/agc/mage/wwwpkgdb/Info/getInfoLabel.php?id=3485449&wwwpkgdb=fc2733d6468c073f1dd738aa598ede55&nocache=62541e24b6e240f187ac920cab60f9da&dir=&wwwpkgdb=fc2733d6468c073f1dd738aa598ede55) | _ | curved DNA-binding protein |
| [OCAR_6910](https://www.genoscope.cns.fr/agc/mage/wwwpkgdb/Info/getInfoLabel.php?id=3483643&wwwpkgdb=fc2733d6468c073f1dd738aa598ede55&nocache=62541e24b6e240f187ac920cab60f9da&dir=&wwwpkgdb=fc2733d6468c073f1dd738aa598ede55) | pdxH | pyridoxamine 5'-phosphate oxidase |
| [OCAR_6911](https://www.genoscope.cns.fr/agc/mage/wwwpkgdb/Info/getInfoLabel.php?id=3483644&wwwpkgdb=fc2733d6468c073f1dd738aa598ede55&nocache=62541e24b6e240f187ac920cab60f9da&dir=&wwwpkgdb=fc2733d6468c073f1dd738aa598ede55) | _ | short-chain dehydrogenase/reductase SDR |
| [OCAR_6914](https://www.genoscope.cns.fr/agc/mage/wwwpkgdb/Info/getInfoLabel.php?id=3485452&wwwpkgdb=fc2733d6468c073f1dd738aa598ede55&nocache=62541e24b6e240f187ac920cab60f9da&dir=&wwwpkgdb=fc2733d6468c073f1dd738aa598ede55) | _ | Mg2+ transporter protein, CorA family protein |
| [OCAR_6915](https://www.genoscope.cns.fr/agc/mage/wwwpkgdb/Info/getInfoLabel.php?id=3483646&wwwpkgdb=fc2733d6468c073f1dd738aa598ede55&nocache=62541e24b6e240f187ac920cab60f9da&dir=&wwwpkgdb=fc2733d6468c073f1dd738aa598ede55) | _ | ErfK/YbiS/YcfS/YnhG |
| [OCAR_6918](https://www.genoscope.cns.fr/agc/mage/wwwpkgdb/Info/getInfoLabel.php?id=3485454&wwwpkgdb=fc2733d6468c073f1dd738aa598ede55&nocache=62541e24b6e240f187ac920cab60f9da&dir=&wwwpkgdb=fc2733d6468c073f1dd738aa598ede55) | _ | metallo-beta-lactamase superfamily protein |
| [OCAR_6920](https://www.genoscope.cns.fr/agc/mage/wwwpkgdb/Info/getInfoLabel.php?id=3485455&wwwpkgdb=fc2733d6468c073f1dd738aa598ede55&nocache=62541e24b6e240f187ac920cab60f9da&dir=&wwwpkgdb=fc2733d6468c073f1dd738aa598ede55) | _ | glutaryl-CoA dehydrogenase, (GCD) |
| [OCAR_6921](https://www.genoscope.cns.fr/agc/mage/wwwpkgdb/Info/getInfoLabel.php?id=3485456&wwwpkgdb=fc2733d6468c073f1dd738aa598ede55&nocache=62541e24b6e240f187ac920cab60f9da&dir=&wwwpkgdb=fc2733d6468c073f1dd738aa598ede55) | ribBA | 3,4-dihydroxy-2-butanone 4-phosphate synthase/GTP cyclohydrolase II |
| [OCAR_6924](https://www.genoscope.cns.fr/agc/mage/wwwpkgdb/Info/getInfoLabel.php?id=3483650&wwwpkgdb=fc2733d6468c073f1dd738aa598ede55&nocache=62541e24b6e240f187ac920cab60f9da&dir=&wwwpkgdb=fc2733d6468c073f1dd738aa598ede55) | _ | Sua5/YciO/YrdC/YwlC family protein |
| [OCAR_6930](https://www.genoscope.cns.fr/agc/mage/wwwpkgdb/Info/getInfoLabel.php?id=3483655&wwwpkgdb=fc2733d6468c073f1dd738aa598ede55&nocache=62541e24b6e240f187ac920cab60f9da&dir=&wwwpkgdb=fc2733d6468c073f1dd738aa598ede55) | _ | hypothetical protein |
| [OCAR_6931](https://www.genoscope.cns.fr/agc/mage/wwwpkgdb/Info/getInfoLabel.php?id=3485459&wwwpkgdb=fc2733d6468c073f1dd738aa598ede55&nocache=62541e24b6e240f187ac920cab60f9da&dir=&wwwpkgdb=fc2733d6468c073f1dd738aa598ede55) | _ | dihydroorotase, multifunctional complex type |
| [OCAR_6933](https://www.genoscope.cns.fr/agc/mage/wwwpkgdb/Info/getInfoLabel.php?id=3483657&wwwpkgdb=fc2733d6468c073f1dd738aa598ede55&nocache=62541e24b6e240f187ac920cab60f9da&dir=&wwwpkgdb=fc2733d6468c073f1dd738aa598ede55) | _ | dna-3-methyladenine glycosylase 1 |
| [OCAR_6934](https://www.genoscope.cns.fr/agc/mage/wwwpkgdb/Info/getInfoLabel.php?id=3483658&wwwpkgdb=fc2733d6468c073f1dd738aa598ede55&nocache=62541e24b6e240f187ac920cab60f9da&dir=&wwwpkgdb=fc2733d6468c073f1dd738aa598ede55) | _ | metal dependent phosphohydrolase |
| [OCAR_6940](https://www.genoscope.cns.fr/agc/mage/wwwpkgdb/Info/getInfoLabel.php?id=3485461&wwwpkgdb=fc2733d6468c073f1dd738aa598ede55&nocache=62541e24b6e240f187ac920cab60f9da&dir=&wwwpkgdb=fc2733d6468c073f1dd738aa598ede55) | _ | protein YhdH |
| [OCAR_6946](https://www.genoscope.cns.fr/agc/mage/wwwpkgdb/Info/getInfoLabel.php?id=3485464&wwwpkgdb=fc2733d6468c073f1dd738aa598ede55&nocache=62541e24b6e240f187ac920cab60f9da&dir=&wwwpkgdb=fc2733d6468c073f1dd738aa598ede55) | ppdK | pyruvate, phosphate dikinase |
| [OCAR_6952](https://www.genoscope.cns.fr/agc/mage/wwwpkgdb/Info/getInfoLabel.php?id=3485468&wwwpkgdb=fc2733d6468c073f1dd738aa598ede55&nocache=62541e24b6e240f187ac920cab60f9da&dir=&wwwpkgdb=fc2733d6468c073f1dd738aa598ede55) | glyQ | Glycyl-tRNA synthetase alpha subunit |
| [OCAR_6955](https://www.genoscope.cns.fr/agc/mage/wwwpkgdb/Info/getInfoLabel.php?id=3485470&wwwpkgdb=fc2733d6468c073f1dd738aa598ede55&nocache=62541e24b6e240f187ac920cab60f9da&dir=&wwwpkgdb=fc2733d6468c073f1dd738aa598ede55) | _ | peptidase S49 |
| [OCAR_6957](https://www.genoscope.cns.fr/agc/mage/wwwpkgdb/Info/getInfoLabel.php?id=3485472&wwwpkgdb=fc2733d6468c073f1dd738aa598ede55&nocache=62541e24b6e240f187ac920cab60f9da&dir=&wwwpkgdb=fc2733d6468c073f1dd738aa598ede55) | _ | hypothetical protein |
| [OCAR_6958](https://www.genoscope.cns.fr/agc/mage/wwwpkgdb/Info/getInfoLabel.php?id=3483669&wwwpkgdb=fc2733d6468c073f1dd738aa598ede55&nocache=62541e24b6e240f187ac920cab60f9da&dir=&wwwpkgdb=fc2733d6468c073f1dd738aa598ede55) | _ | octaprenyl-diphosphate synthase |
| [OCAR_6963](https://www.genoscope.cns.fr/agc/mage/wwwpkgdb/Info/getInfoLabel.php?id=3485476&wwwpkgdb=fc2733d6468c073f1dd738aa598ede55&nocache=62541e24b6e240f187ac920cab60f9da&dir=&wwwpkgdb=fc2733d6468c073f1dd738aa598ede55) | _ | electron transfer flavoprotein-ubiquinone oxidoreductase, (ETF-QO) |
| [OCAR_6972](https://www.genoscope.cns.fr/agc/mage/wwwpkgdb/Info/getInfoLabel.php?id=3485479&wwwpkgdb=fc2733d6468c073f1dd738aa598ede55&nocache=62541e24b6e240f187ac920cab60f9da&dir=&wwwpkgdb=fc2733d6468c073f1dd738aa598ede55) | _ | ribonuclease HII (RNase HII) |
| [OCAR_6974](https://www.genoscope.cns.fr/agc/mage/wwwpkgdb/Info/getInfoLabel.php?id=3485481&wwwpkgdb=fc2733d6468c073f1dd738aa598ede55&nocache=62541e24b6e240f187ac920cab60f9da&dir=&wwwpkgdb=fc2733d6468c073f1dd738aa598ede55) | _ | radical SAM |
| [OCAR_6976](https://www.genoscope.cns.fr/agc/mage/wwwpkgdb/Info/getInfoLabel.php?id=3485482&wwwpkgdb=fc2733d6468c073f1dd738aa598ede55&nocache=62541e24b6e240f187ac920cab60f9da&dir=&wwwpkgdb=fc2733d6468c073f1dd738aa598ede55) | moaB | molybdenum cofactor biosynthesis protein B |
| [OCAR_6978](https://www.genoscope.cns.fr/agc/mage/wwwpkgdb/Info/getInfoLabel.php?id=3485484&wwwpkgdb=fc2733d6468c073f1dd738aa598ede55&nocache=62541e24b6e240f187ac920cab60f9da&dir=&wwwpkgdb=fc2733d6468c073f1dd738aa598ede55) | _ | zinc-dependent peptidase |
| [OCAR_6979](https://www.genoscope.cns.fr/agc/mage/wwwpkgdb/Info/getInfoLabel.php?id=3483678&wwwpkgdb=fc2733d6468c073f1dd738aa598ede55&nocache=62541e24b6e240f187ac920cab60f9da&dir=&wwwpkgdb=fc2733d6468c073f1dd738aa598ede55) | _ | modification methylase CcrMI |
| [OCAR_6982](https://www.genoscope.cns.fr/agc/mage/wwwpkgdb/Info/getInfoLabel.php?id=3485487&wwwpkgdb=fc2733d6468c073f1dd738aa598ede55&nocache=62541e24b6e240f187ac920cab60f9da&dir=&wwwpkgdb=fc2733d6468c073f1dd738aa598ede55) | mutY | A/G-specific adenine glycosylase |
| [OCAR_6985](https://www.genoscope.cns.fr/agc/mage/wwwpkgdb/Info/getInfoLabel.php?id=3483681&wwwpkgdb=fc2733d6468c073f1dd738aa598ede55&nocache=62541e24b6e240f187ac920cab60f9da&dir=&wwwpkgdb=fc2733d6468c073f1dd738aa598ede55) | smc | chromosome segregation protein SMC |
| [OCAR_6988](https://www.genoscope.cns.fr/agc/mage/wwwpkgdb/Info/getInfoLabel.php?id=3483684&wwwpkgdb=fc2733d6468c073f1dd738aa598ede55&nocache=62541e24b6e240f187ac920cab60f9da&dir=&wwwpkgdb=fc2733d6468c073f1dd738aa598ede55) | _ | LemA |
| [OCAR_6990](https://www.genoscope.cns.fr/agc/mage/wwwpkgdb/Info/getInfoLabel.php?id=3483686&wwwpkgdb=fc2733d6468c073f1dd738aa598ede55&nocache=62541e24b6e240f187ac920cab60f9da&dir=&wwwpkgdb=fc2733d6468c073f1dd738aa598ede55) | _ | hypothetical protein |
| [OCAR_6992](https://www.genoscope.cns.fr/agc/mage/wwwpkgdb/Info/getInfoLabel.php?id=3485488&wwwpkgdb=fc2733d6468c073f1dd738aa598ede55&nocache=62541e24b6e240f187ac920cab60f9da&dir=&wwwpkgdb=fc2733d6468c073f1dd738aa598ede55) | apt | adenine phosphoribosyltransferase |
| [OCAR_6993](https://www.genoscope.cns.fr/agc/mage/wwwpkgdb/Info/getInfoLabel.php?id=3485489&wwwpkgdb=fc2733d6468c073f1dd738aa598ede55&nocache=62541e24b6e240f187ac920cab60f9da&dir=&wwwpkgdb=fc2733d6468c073f1dd738aa598ede55) | _ | anthranilate synthase |
| [OCAR_6995](https://www.genoscope.cns.fr/agc/mage/wwwpkgdb/Info/getInfoLabel.php?id=3485491&wwwpkgdb=fc2733d6468c073f1dd738aa598ede55&nocache=62541e24b6e240f187ac920cab60f9da&dir=&wwwpkgdb=fc2733d6468c073f1dd738aa598ede55) | _ | cytochrome B/c1 |
| [OCAR_6996](https://www.genoscope.cns.fr/agc/mage/wwwpkgdb/Info/getInfoLabel.php?id=3485492&wwwpkgdb=fc2733d6468c073f1dd738aa598ede55&nocache=62541e24b6e240f187ac920cab60f9da&dir=&wwwpkgdb=fc2733d6468c073f1dd738aa598ede55) | petA | ubiquinol-cytochrome c reductase, iron-sulfur subunit |
| [OCAR_6997](https://www.genoscope.cns.fr/agc/mage/wwwpkgdb/Info/getInfoLabel.php?id=3483688&wwwpkgdb=fc2733d6468c073f1dd738aa598ede55&nocache=62541e24b6e240f187ac920cab60f9da&dir=&wwwpkgdb=fc2733d6468c073f1dd738aa598ede55) | _ | tRNA/rRNA methyltransferase |
| [OCAR_6998](https://www.genoscope.cns.fr/agc/mage/wwwpkgdb/Info/getInfoLabel.php?id=3483689&wwwpkgdb=fc2733d6468c073f1dd738aa598ede55&nocache=62541e24b6e240f187ac920cab60f9da&dir=&wwwpkgdb=fc2733d6468c073f1dd738aa598ede55) | _ | radical SAM |
| [OCAR_6999](https://www.genoscope.cns.fr/agc/mage/wwwpkgdb/Info/getInfoLabel.php?id=3483690&wwwpkgdb=fc2733d6468c073f1dd738aa598ede55&nocache=62541e24b6e240f187ac920cab60f9da&dir=&wwwpkgdb=fc2733d6468c073f1dd738aa598ede55) | _ | 6-pyruvoyl tetrahydrobiopterin synthase |
| [OCAR_7000](https://www.genoscope.cns.fr/agc/mage/wwwpkgdb/Info/getInfoLabel.php?id=3483691&wwwpkgdb=fc2733d6468c073f1dd738aa598ede55&nocache=62541e24b6e240f187ac920cab60f9da&dir=&wwwpkgdb=fc2733d6468c073f1dd738aa598ede55) | hemF | coproporphyrinogen III oxidase, aerobic |
| [OCAR_7008](https://www.genoscope.cns.fr/agc/mage/wwwpkgdb/Info/getInfoLabel.php?id=3483694&wwwpkgdb=fc2733d6468c073f1dd738aa598ede55&nocache=62541e24b6e240f187ac920cab60f9da&dir=&wwwpkgdb=fc2733d6468c073f1dd738aa598ede55) | _ | ATPase associated with various cellular activities, AAA_3 |
| [OCAR_7010](https://www.genoscope.cns.fr/agc/mage/wwwpkgdb/Info/getInfoLabel.php?id=3483696&wwwpkgdb=fc2733d6468c073f1dd738aa598ede55&nocache=62541e24b6e240f187ac920cab60f9da&dir=&wwwpkgdb=fc2733d6468c073f1dd738aa598ede55) | _ | double-transmembrane region |
| [OCAR_7011](https://www.genoscope.cns.fr/agc/mage/wwwpkgdb/Info/getInfoLabel.php?id=3483697&wwwpkgdb=fc2733d6468c073f1dd738aa598ede55&nocache=62541e24b6e240f187ac920cab60f9da&dir=&wwwpkgdb=fc2733d6468c073f1dd738aa598ede55) | _ | hypothetical protein |
| [OCAR_7017](https://www.genoscope.cns.fr/agc/mage/wwwpkgdb/Info/getInfoLabel.php?id=3485500&wwwpkgdb=fc2733d6468c073f1dd738aa598ede55&nocache=62541e24b6e240f187ac920cab60f9da&dir=&wwwpkgdb=fc2733d6468c073f1dd738aa598ede55) | _ | acetyltransferase |
| [OCAR_7021](https://www.genoscope.cns.fr/agc/mage/wwwpkgdb/Info/getInfoLabel.php?id=3485503&wwwpkgdb=fc2733d6468c073f1dd738aa598ede55&nocache=62541e24b6e240f187ac920cab60f9da&dir=&wwwpkgdb=fc2733d6468c073f1dd738aa598ede55) | _ | hypothetical protein |
| [OCAR_7023](https://www.genoscope.cns.fr/agc/mage/wwwpkgdb/Info/getInfoLabel.php?id=3483702&wwwpkgdb=fc2733d6468c073f1dd738aa598ede55&nocache=62541e24b6e240f187ac920cab60f9da&dir=&wwwpkgdb=fc2733d6468c073f1dd738aa598ede55) | _ | DNA-binding protein |
| [OCAR_7028](https://www.genoscope.cns.fr/agc/mage/wwwpkgdb/Info/getInfoLabel.php?id=3483704&wwwpkgdb=fc2733d6468c073f1dd738aa598ede55&nocache=62541e24b6e240f187ac920cab60f9da&dir=&wwwpkgdb=fc2733d6468c073f1dd738aa598ede55) | thiO | glycine oxidase ThiO |
| [OCAR_7030](https://www.genoscope.cns.fr/agc/mage/wwwpkgdb/Info/getInfoLabel.php?id=3483706&wwwpkgdb=fc2733d6468c073f1dd738aa598ede55&nocache=62541e24b6e240f187ac920cab60f9da&dir=&wwwpkgdb=fc2733d6468c073f1dd738aa598ede55) | thiG | thiazole biosynthesis protein ThiG |
| [OCAR_7031](https://www.genoscope.cns.fr/agc/mage/wwwpkgdb/Info/getInfoLabel.php?id=3483707&wwwpkgdb=fc2733d6468c073f1dd738aa598ede55&nocache=62541e24b6e240f187ac920cab60f9da&dir=&wwwpkgdb=fc2733d6468c073f1dd738aa598ede55) | _ | thiamine-phosphate pyrophosphorylase |
| [OCAR_7033](https://www.genoscope.cns.fr/agc/mage/wwwpkgdb/Info/getInfoLabel.php?id=3483709&wwwpkgdb=fc2733d6468c073f1dd738aa598ede55&nocache=62541e24b6e240f187ac920cab60f9da&dir=&wwwpkgdb=fc2733d6468c073f1dd738aa598ede55) | thiC | Thiamine biosynthesis protein thiC |
| [OCAR_7040](https://www.genoscope.cns.fr/agc/mage/wwwpkgdb/Info/getInfoLabel.php?id=3485509&wwwpkgdb=fc2733d6468c073f1dd738aa598ede55&nocache=62541e24b6e240f187ac920cab60f9da&dir=&wwwpkgdb=fc2733d6468c073f1dd738aa598ede55) | bfr | bacterioferritin |
| [OCAR_7045](https://www.genoscope.cns.fr/agc/mage/wwwpkgdb/Info/getInfoLabel.php?id=3485511&wwwpkgdb=fc2733d6468c073f1dd738aa598ede55&nocache=62541e24b6e240f187ac920cab60f9da&dir=&wwwpkgdb=fc2733d6468c073f1dd738aa598ede55) | _ | lytic murein transglycosylase |
| [OCAR_7046](https://www.genoscope.cns.fr/agc/mage/wwwpkgdb/Info/getInfoLabel.php?id=3485512&wwwpkgdb=fc2733d6468c073f1dd738aa598ede55&nocache=62541e24b6e240f187ac920cab60f9da&dir=&wwwpkgdb=fc2733d6468c073f1dd738aa598ede55) | _ | hypothetical protein |
| [OCAR_7048](https://www.genoscope.cns.fr/agc/mage/wwwpkgdb/Info/getInfoLabel.php?id=3485514&wwwpkgdb=fc2733d6468c073f1dd738aa598ede55&nocache=62541e24b6e240f187ac920cab60f9da&dir=&wwwpkgdb=fc2733d6468c073f1dd738aa598ede55) | _ | putative ABC transporter ATP-binding protein ChvD |
| [OCAR_7053](https://www.genoscope.cns.fr/agc/mage/wwwpkgdb/Info/getInfoLabel.php?id=3485517&wwwpkgdb=fc2733d6468c073f1dd738aa598ede55&nocache=62541e24b6e240f187ac920cab60f9da&dir=&wwwpkgdb=fc2733d6468c073f1dd738aa598ede55) | _ | rhodanese domain protein |
| [OCAR_7060](https://www.genoscope.cns.fr/agc/mage/wwwpkgdb/Info/getInfoLabel.php?id=3485523&wwwpkgdb=fc2733d6468c073f1dd738aa598ede55&nocache=62541e24b6e240f187ac920cab60f9da&dir=&wwwpkgdb=fc2733d6468c073f1dd738aa598ede55) | _ | hypothetical protein |
| [OCAR_7064](https://www.genoscope.cns.fr/agc/mage/wwwpkgdb/Info/getInfoLabel.php?id=3485917&wwwpkgdb=fc2733d6468c073f1dd738aa598ede55&nocache=62541e24b6e240f187ac920cab60f9da&dir=&wwwpkgdb=fc2733d6468c073f1dd738aa598ede55) | _ | trehalose synthase; nonfunctional due to frameshift; identified by match to protein family HMM PF00128; match to protein family HMM TIGR02456 |
| [OCAR_7065](https://www.genoscope.cns.fr/agc/mage/wwwpkgdb/Info/getInfoLabel.php?id=3483724&wwwpkgdb=fc2733d6468c073f1dd738aa598ede55&nocache=62541e24b6e240f187ac920cab60f9da&dir=&wwwpkgdb=fc2733d6468c073f1dd738aa598ede55) | glgB | 1,4-alpha-glucan branching enzyme |
| [OCAR_7066](https://www.genoscope.cns.fr/agc/mage/wwwpkgdb/Info/getInfoLabel.php?id=3483725&wwwpkgdb=fc2733d6468c073f1dd738aa598ede55&nocache=62541e24b6e240f187ac920cab60f9da&dir=&wwwpkgdb=fc2733d6468c073f1dd738aa598ede55) | glgX | glycogen debranching enzyme GlgX |
| [OCAR_7105](https://www.genoscope.cns.fr/agc/mage/wwwpkgdb/Info/getInfoLabel.php?id=3483737&wwwpkgdb=fc2733d6468c073f1dd738aa598ede55&nocache=62541e24b6e240f187ac920cab60f9da&dir=&wwwpkgdb=fc2733d6468c073f1dd738aa598ede55) | flhA | flagellar biosynthesis protein FlhA |
| [OCAR_7109](https://www.genoscope.cns.fr/agc/mage/wwwpkgdb/Info/getInfoLabel.php?id=3485554&wwwpkgdb=fc2733d6468c073f1dd738aa598ede55&nocache=62541e24b6e240f187ac920cab60f9da&dir=&wwwpkgdb=fc2733d6468c073f1dd738aa598ede55) | fliJ | flagellar export protein FliJ |
| [OCAR_7110](https://www.genoscope.cns.fr/agc/mage/wwwpkgdb/Info/getInfoLabel.php?id=3485555&wwwpkgdb=fc2733d6468c073f1dd738aa598ede55&nocache=62541e24b6e240f187ac920cab60f9da&dir=&wwwpkgdb=fc2733d6468c073f1dd738aa598ede55) | fliI | flagellar protein export ATPase FliI |
| [OCAR_7111](https://www.genoscope.cns.fr/agc/mage/wwwpkgdb/Info/getInfoLabel.php?id=3483739&wwwpkgdb=fc2733d6468c073f1dd738aa598ede55&nocache=62541e24b6e240f187ac920cab60f9da&dir=&wwwpkgdb=fc2733d6468c073f1dd738aa598ede55) | _ | cell cycle transcriptional regulator CtrA |
| [OCAR_7114](https://www.genoscope.cns.fr/agc/mage/wwwpkgdb/Info/getInfoLabel.php?id=3485558&wwwpkgdb=fc2733d6468c073f1dd738aa598ede55&nocache=62541e24b6e240f187ac920cab60f9da&dir=&wwwpkgdb=fc2733d6468c073f1dd738aa598ede55) | _ | response regulator receiver |
| [OCAR_7115](https://www.genoscope.cns.fr/agc/mage/wwwpkgdb/Info/getInfoLabel.php?id=3485559&wwwpkgdb=fc2733d6468c073f1dd738aa598ede55&nocache=62541e24b6e240f187ac920cab60f9da&dir=&wwwpkgdb=fc2733d6468c073f1dd738aa598ede55) | _ | CheW protein |
| [OCAR_7116](https://www.genoscope.cns.fr/agc/mage/wwwpkgdb/Info/getInfoLabel.php?id=3485560&wwwpkgdb=fc2733d6468c073f1dd738aa598ede55&nocache=62541e24b6e240f187ac920cab60f9da&dir=&wwwpkgdb=fc2733d6468c073f1dd738aa598ede55) | _ | CheA signal transduction histidine kinases |
| [OCAR_7120](https://www.genoscope.cns.fr/agc/mage/wwwpkgdb/Info/getInfoLabel.php?id=3483741&wwwpkgdb=fc2733d6468c073f1dd738aa598ede55&nocache=62541e24b6e240f187ac920cab60f9da&dir=&wwwpkgdb=fc2733d6468c073f1dd738aa598ede55) | _ | hypothetical protein |
| [OCAR_7123](https://www.genoscope.cns.fr/agc/mage/wwwpkgdb/Info/getInfoLabel.php?id=3483744&wwwpkgdb=fc2733d6468c073f1dd738aa598ede55&nocache=62541e24b6e240f187ac920cab60f9da&dir=&wwwpkgdb=fc2733d6468c073f1dd738aa598ede55) | _ | ErfK/YbiS/YcfS/YnhG |
| [OCAR_7126](https://www.genoscope.cns.fr/agc/mage/wwwpkgdb/Info/getInfoLabel.php?id=3483746&wwwpkgdb=fc2733d6468c073f1dd738aa598ede55&nocache=62541e24b6e240f187ac920cab60f9da&dir=&wwwpkgdb=fc2733d6468c073f1dd738aa598ede55) | hemE | uroporphyrinogen decarboxylase |
| [OCAR_7148](https://www.genoscope.cns.fr/agc/mage/wwwpkgdb/Info/getInfoLabel.php?id=3483762&wwwpkgdb=fc2733d6468c073f1dd738aa598ede55&nocache=62541e24b6e240f187ac920cab60f9da&dir=&wwwpkgdb=fc2733d6468c073f1dd738aa598ede55) | _ | ribosomal large subunit pseudouridine synthase D |
| [OCAR_7149](https://www.genoscope.cns.fr/agc/mage/wwwpkgdb/Info/getInfoLabel.php?id=3483763&wwwpkgdb=fc2733d6468c073f1dd738aa598ede55&nocache=62541e24b6e240f187ac920cab60f9da&dir=&wwwpkgdb=fc2733d6468c073f1dd738aa598ede55) | rpoH | alternative sigma factor RpoH |
| [OCAR_7155](https://www.genoscope.cns.fr/agc/mage/wwwpkgdb/Info/getInfoLabel.php?id=3485574&wwwpkgdb=fc2733d6468c073f1dd738aa598ede55&nocache=62541e24b6e240f187ac920cab60f9da&dir=&wwwpkgdb=fc2733d6468c073f1dd738aa598ede55) | purA | adenylosuccinate synthetase |
| [OCAR_7157](https://www.genoscope.cns.fr/agc/mage/wwwpkgdb/Info/getInfoLabel.php?id=3485575&wwwpkgdb=fc2733d6468c073f1dd738aa598ede55&nocache=62541e24b6e240f187ac920cab60f9da&dir=&wwwpkgdb=fc2733d6468c073f1dd738aa598ede55) | serA | phosphoglycerate dehydrogenase |
| [OCAR_7158](https://www.genoscope.cns.fr/agc/mage/wwwpkgdb/Info/getInfoLabel.php?id=3485576&wwwpkgdb=fc2733d6468c073f1dd738aa598ede55&nocache=62541e24b6e240f187ac920cab60f9da&dir=&wwwpkgdb=fc2733d6468c073f1dd738aa598ede55) | _ | phosphoserine aminotransferase |
| [OCAR_7164](https://www.genoscope.cns.fr/agc/mage/wwwpkgdb/Info/getInfoLabel.php?id=3485580&wwwpkgdb=fc2733d6468c073f1dd738aa598ede55&nocache=62541e24b6e240f187ac920cab60f9da&dir=&wwwpkgdb=fc2733d6468c073f1dd738aa598ede55) | glmM | Phosphoglucosamine mutase |
| [OCAR_7173](https://www.genoscope.cns.fr/agc/mage/wwwpkgdb/Info/getInfoLabel.php?id=3485587&wwwpkgdb=fc2733d6468c073f1dd738aa598ede55&nocache=62541e24b6e240f187ac920cab60f9da&dir=&wwwpkgdb=fc2733d6468c073f1dd738aa598ede55) | _ | aspartate aminotransferase A |
| [OCAR_7175](https://www.genoscope.cns.fr/agc/mage/wwwpkgdb/Info/getInfoLabel.php?id=3483771&wwwpkgdb=fc2733d6468c073f1dd738aa598ede55&nocache=62541e24b6e240f187ac920cab60f9da&dir=&wwwpkgdb=fc2733d6468c073f1dd738aa598ede55) | _ | glutathione S-transferase |
| [OCAR_7183](https://www.genoscope.cns.fr/agc/mage/wwwpkgdb/Info/getInfoLabel.php?id=3483776&wwwpkgdb=fc2733d6468c073f1dd738aa598ede55&nocache=62541e24b6e240f187ac920cab60f9da&dir=&wwwpkgdb=fc2733d6468c073f1dd738aa598ede55) | _ | excinuclease ABC subunit B |
| [OCAR_7184](https://www.genoscope.cns.fr/agc/mage/wwwpkgdb/Info/getInfoLabel.php?id=3485591&wwwpkgdb=fc2733d6468c073f1dd738aa598ede55&nocache=62541e24b6e240f187ac920cab60f9da&dir=&wwwpkgdb=fc2733d6468c073f1dd738aa598ede55) | _ | ATP-binding protein of ABC transporter, duplicated ATPase domains |
| [OCAR_7187](https://www.genoscope.cns.fr/agc/mage/wwwpkgdb/Info/getInfoLabel.php?id=3483779&wwwpkgdb=fc2733d6468c073f1dd738aa598ede55&nocache=62541e24b6e240f187ac920cab60f9da&dir=&wwwpkgdb=fc2733d6468c073f1dd738aa598ede55) | _ | hypothetical protein |
| [OCAR_7188](https://www.genoscope.cns.fr/agc/mage/wwwpkgdb/Info/getInfoLabel.php?id=3485592&wwwpkgdb=fc2733d6468c073f1dd738aa598ede55&nocache=62541e24b6e240f187ac920cab60f9da&dir=&wwwpkgdb=fc2733d6468c073f1dd738aa598ede55) | ychF | GTP-binding protein YchF |
| [OCAR_7189](https://www.genoscope.cns.fr/agc/mage/wwwpkgdb/Info/getInfoLabel.php?id=3485593&wwwpkgdb=fc2733d6468c073f1dd738aa598ede55&nocache=62541e24b6e240f187ac920cab60f9da&dir=&wwwpkgdb=fc2733d6468c073f1dd738aa598ede55) | pth | Peptidyl-tRNA hydrolase |
| [OCAR_7190](https://www.genoscope.cns.fr/agc/mage/wwwpkgdb/Info/getInfoLabel.php?id=3485594&wwwpkgdb=fc2733d6468c073f1dd738aa598ede55&nocache=62541e24b6e240f187ac920cab60f9da&dir=&wwwpkgdb=fc2733d6468c073f1dd738aa598ede55) | rplY | 50S ribosomal protein L25 |
| [OCAR_7192](https://www.genoscope.cns.fr/agc/mage/wwwpkgdb/Info/getInfoLabel.php?id=3485595&wwwpkgdb=fc2733d6468c073f1dd738aa598ede55&nocache=62541e24b6e240f187ac920cab60f9da&dir=&wwwpkgdb=fc2733d6468c073f1dd738aa598ede55) | _ | hypothetical protein |
| [OCAR_7193](https://www.genoscope.cns.fr/agc/mage/wwwpkgdb/Info/getInfoLabel.php?id=3483781&wwwpkgdb=fc2733d6468c073f1dd738aa598ede55&nocache=62541e24b6e240f187ac920cab60f9da&dir=&wwwpkgdb=fc2733d6468c073f1dd738aa598ede55) | lgt | prolipoprotein diacylglyceryl transferase |
| [OCAR_7197](https://www.genoscope.cns.fr/agc/mage/wwwpkgdb/Info/getInfoLabel.php?id=3483785&wwwpkgdb=fc2733d6468c073f1dd738aa598ede55&nocache=62541e24b6e240f187ac920cab60f9da&dir=&wwwpkgdb=fc2733d6468c073f1dd738aa598ede55) | _ | ribose-Phosphate pyrophosphokinase (rppk) |
| [OCAR_7201](https://www.genoscope.cns.fr/agc/mage/wwwpkgdb/Info/getInfoLabel.php?id=3485596&wwwpkgdb=fc2733d6468c073f1dd738aa598ede55&nocache=62541e24b6e240f187ac920cab60f9da&dir=&wwwpkgdb=fc2733d6468c073f1dd738aa598ede55) | _ | two-component hybrid sensor and regulator |
| [OCAR_7202](https://www.genoscope.cns.fr/agc/mage/wwwpkgdb/Info/getInfoLabel.php?id=3485597&wwwpkgdb=fc2733d6468c073f1dd738aa598ede55&nocache=62541e24b6e240f187ac920cab60f9da&dir=&wwwpkgdb=fc2733d6468c073f1dd738aa598ede55) | _ | protein PetR |
| [OCAR_7203](https://www.genoscope.cns.fr/agc/mage/wwwpkgdb/Info/getInfoLabel.php?id=3485598&wwwpkgdb=fc2733d6468c073f1dd738aa598ede55&nocache=62541e24b6e240f187ac920cab60f9da&dir=&wwwpkgdb=fc2733d6468c073f1dd738aa598ede55) | _ | transcriptional regulator of MarR family |
| [OCAR_7205](https://www.genoscope.cns.fr/agc/mage/wwwpkgdb/Info/getInfoLabel.php?id=3483789&wwwpkgdb=fc2733d6468c073f1dd738aa598ede55&nocache=62541e24b6e240f187ac920cab60f9da&dir=&wwwpkgdb=fc2733d6468c073f1dd738aa598ede55) | ilvE | branched-chain amino acid aminotransferase |
| [OCAR_7211](https://www.genoscope.cns.fr/agc/mage/wwwpkgdb/Info/getInfoLabel.php?id=3485605&wwwpkgdb=fc2733d6468c073f1dd738aa598ede55&nocache=62541e24b6e240f187ac920cab60f9da&dir=&wwwpkgdb=fc2733d6468c073f1dd738aa598ede55) | moaD | molybdopterin converting factor, subunit 1 |
| [OCAR_7212](https://www.genoscope.cns.fr/agc/mage/wwwpkgdb/Info/getInfoLabel.php?id=3485606&wwwpkgdb=fc2733d6468c073f1dd738aa598ede55&nocache=62541e24b6e240f187ac920cab60f9da&dir=&wwwpkgdb=fc2733d6468c073f1dd738aa598ede55) | pgsA | CDP-diacylglycerol--glycerol-3-phosphate 3-phosphatidyltransferase |
| [OCAR_7213](https://www.genoscope.cns.fr/agc/mage/wwwpkgdb/Info/getInfoLabel.php?id=3485607&wwwpkgdb=fc2733d6468c073f1dd738aa598ede55&nocache=62541e24b6e240f187ac920cab60f9da&dir=&wwwpkgdb=fc2733d6468c073f1dd738aa598ede55) | uvrC | excinuclease ABC, C subunit |
| [OCAR_7215](https://www.genoscope.cns.fr/agc/mage/wwwpkgdb/Info/getInfoLabel.php?id=3485608&wwwpkgdb=fc2733d6468c073f1dd738aa598ede55&nocache=62541e24b6e240f187ac920cab60f9da&dir=&wwwpkgdb=fc2733d6468c073f1dd738aa598ede55) | _ | putative cold-shock' DNA-binding domain |
| [OCAR_7216](https://www.genoscope.cns.fr/agc/mage/wwwpkgdb/Info/getInfoLabel.php?id=3485609&wwwpkgdb=fc2733d6468c073f1dd738aa598ede55&nocache=62541e24b6e240f187ac920cab60f9da&dir=&wwwpkgdb=fc2733d6468c073f1dd738aa598ede55) | _ | hypothetical protein |
| [OCAR_7217](https://www.genoscope.cns.fr/agc/mage/wwwpkgdb/Info/getInfoLabel.php?id=3485610&wwwpkgdb=fc2733d6468c073f1dd738aa598ede55&nocache=62541e24b6e240f187ac920cab60f9da&dir=&wwwpkgdb=fc2733d6468c073f1dd738aa598ede55) | _ | ribonuclease T2 family |
| [OCAR_7231](https://www.genoscope.cns.fr/agc/mage/wwwpkgdb/Info/getInfoLabel.php?id=3485619&wwwpkgdb=fc2733d6468c073f1dd738aa598ede55&nocache=62541e24b6e240f187ac920cab60f9da&dir=&wwwpkgdb=fc2733d6468c073f1dd738aa598ede55) | _ | two-component response regulator |
| [OCAR_7234](https://www.genoscope.cns.fr/agc/mage/wwwpkgdb/Info/getInfoLabel.php?id=3483797&wwwpkgdb=fc2733d6468c073f1dd738aa598ede55&nocache=62541e24b6e240f187ac920cab60f9da&dir=&wwwpkgdb=fc2733d6468c073f1dd738aa598ede55) | ileS | isoleucyl-tRNA synthetase |
| [OCAR_7238](https://www.genoscope.cns.fr/agc/mage/wwwpkgdb/Info/getInfoLabel.php?id=3483800&wwwpkgdb=fc2733d6468c073f1dd738aa598ede55&nocache=62541e24b6e240f187ac920cab60f9da&dir=&wwwpkgdb=fc2733d6468c073f1dd738aa598ede55) | _ | peptidase M16 domain protein |
| [OCAR_7240](https://www.genoscope.cns.fr/agc/mage/wwwpkgdb/Info/getInfoLabel.php?id=3483802&wwwpkgdb=fc2733d6468c073f1dd738aa598ede55&nocache=62541e24b6e240f187ac920cab60f9da&dir=&wwwpkgdb=fc2733d6468c073f1dd738aa598ede55) | _ | class I peptide chain release factor |
| [OCAR_7241](https://www.genoscope.cns.fr/agc/mage/wwwpkgdb/Info/getInfoLabel.php?id=3483803&wwwpkgdb=fc2733d6468c073f1dd738aa598ede55&nocache=62541e24b6e240f187ac920cab60f9da&dir=&wwwpkgdb=fc2733d6468c073f1dd738aa598ede55) | mutL | DNA mismatch repair protein mutL |
| [OCAR_7242](https://www.genoscope.cns.fr/agc/mage/wwwpkgdb/Info/getInfoLabel.php?id=3485622&wwwpkgdb=fc2733d6468c073f1dd738aa598ede55&nocache=62541e24b6e240f187ac920cab60f9da&dir=&wwwpkgdb=fc2733d6468c073f1dd738aa598ede55) | _ | putative methyltransferase |
| [OCAR_7244](https://www.genoscope.cns.fr/agc/mage/wwwpkgdb/Info/getInfoLabel.php?id=3483804&wwwpkgdb=fc2733d6468c073f1dd738aa598ede55&nocache=62541e24b6e240f187ac920cab60f9da&dir=&wwwpkgdb=fc2733d6468c073f1dd738aa598ede55) | _ | tRNA-specific adenosine deaminase |
| [OCAR_7246](https://www.genoscope.cns.fr/agc/mage/wwwpkgdb/Info/getInfoLabel.php?id=3485625&wwwpkgdb=fc2733d6468c073f1dd738aa598ede55&nocache=62541e24b6e240f187ac920cab60f9da&dir=&wwwpkgdb=fc2733d6468c073f1dd738aa598ede55) | purD | phosphoribosylamine--glycine ligase |
| [OCAR_7256](https://www.genoscope.cns.fr/agc/mage/wwwpkgdb/Info/getInfoLabel.php?id=3485632&wwwpkgdb=fc2733d6468c073f1dd738aa598ede55&nocache=62541e24b6e240f187ac920cab60f9da&dir=&wwwpkgdb=fc2733d6468c073f1dd738aa598ede55) | _ | TldD/PmbA family protein |
| [OCAR_7258](https://www.genoscope.cns.fr/agc/mage/wwwpkgdb/Info/getInfoLabel.php?id=3485633&wwwpkgdb=fc2733d6468c073f1dd738aa598ede55&nocache=62541e24b6e240f187ac920cab60f9da&dir=&wwwpkgdb=fc2733d6468c073f1dd738aa598ede55) | ubiA | 4-hydroxybenzoate polyprenyl transferase |
| [OCAR_7262](https://www.genoscope.cns.fr/agc/mage/wwwpkgdb/Info/getInfoLabel.php?id=3483811&wwwpkgdb=fc2733d6468c073f1dd738aa598ede55&nocache=62541e24b6e240f187ac920cab60f9da&dir=&wwwpkgdb=fc2733d6468c073f1dd738aa598ede55) | _ | tRNA synthetase, class II |
| [OCAR_7263](https://www.genoscope.cns.fr/agc/mage/wwwpkgdb/Info/getInfoLabel.php?id=3483812&wwwpkgdb=fc2733d6468c073f1dd738aa598ede55&nocache=62541e24b6e240f187ac920cab60f9da&dir=&wwwpkgdb=fc2733d6468c073f1dd738aa598ede55) | hisG | ATP phosphoribosyltransferase |
| [OCAR_7264](https://www.genoscope.cns.fr/agc/mage/wwwpkgdb/Info/getInfoLabel.php?id=3483813&wwwpkgdb=fc2733d6468c073f1dd738aa598ede55&nocache=62541e24b6e240f187ac920cab60f9da&dir=&wwwpkgdb=fc2733d6468c073f1dd738aa598ede55) | _ | bactoprenol glucosyl transferase |
| [OCAR_7271](https://www.genoscope.cns.fr/agc/mage/wwwpkgdb/Info/getInfoLabel.php?id=3483817&wwwpkgdb=fc2733d6468c073f1dd738aa598ede55&nocache=62541e24b6e240f187ac920cab60f9da&dir=&wwwpkgdb=fc2733d6468c073f1dd738aa598ede55) | groS | chaperonin GroS |
| [OCAR_7272](https://www.genoscope.cns.fr/agc/mage/wwwpkgdb/Info/getInfoLabel.php?id=3483818&wwwpkgdb=fc2733d6468c073f1dd738aa598ede55&nocache=62541e24b6e240f187ac920cab60f9da&dir=&wwwpkgdb=fc2733d6468c073f1dd738aa598ede55) | groL | chaperonin GroL |
| [OCAR_7281](https://www.genoscope.cns.fr/agc/mage/wwwpkgdb/Info/getInfoLabel.php?id=3485642&wwwpkgdb=fc2733d6468c073f1dd738aa598ede55&nocache=62541e24b6e240f187ac920cab60f9da&dir=&wwwpkgdb=fc2733d6468c073f1dd738aa598ede55) | _ | glycolate oxidase iron-sulfur subunit |
| [OCAR_7283](https://www.genoscope.cns.fr/agc/mage/wwwpkgdb/Info/getInfoLabel.php?id=3485644&wwwpkgdb=fc2733d6468c073f1dd738aa598ede55&nocache=62541e24b6e240f187ac920cab60f9da&dir=&wwwpkgdb=fc2733d6468c073f1dd738aa598ede55) | glcD | glycolate oxidase, subunit GlcD |
| [OCAR_7284](https://www.genoscope.cns.fr/agc/mage/wwwpkgdb/Info/getInfoLabel.php?id=3485645&wwwpkgdb=fc2733d6468c073f1dd738aa598ede55&nocache=62541e24b6e240f187ac920cab60f9da&dir=&wwwpkgdb=fc2733d6468c073f1dd738aa598ede55) | _ | transcriptional regulator, TetR family |
| [OCAR_7286](https://www.genoscope.cns.fr/agc/mage/wwwpkgdb/Info/getInfoLabel.php?id=3485647&wwwpkgdb=fc2733d6468c073f1dd738aa598ede55&nocache=62541e24b6e240f187ac920cab60f9da&dir=&wwwpkgdb=fc2733d6468c073f1dd738aa598ede55) | _ | putative Cell division protease FtsH -like protein |
| [OCAR_7289](https://www.genoscope.cns.fr/agc/mage/wwwpkgdb/Info/getInfoLabel.php?id=3485650&wwwpkgdb=fc2733d6468c073f1dd738aa598ede55&nocache=62541e24b6e240f187ac920cab60f9da&dir=&wwwpkgdb=fc2733d6468c073f1dd738aa598ede55) | _ | OmpA/MotB domain protein |
| [OCAR_7290](https://www.genoscope.cns.fr/agc/mage/wwwpkgdb/Info/getInfoLabel.php?id=3485651&wwwpkgdb=fc2733d6468c073f1dd738aa598ede55&nocache=62541e24b6e240f187ac920cab60f9da&dir=&wwwpkgdb=fc2733d6468c073f1dd738aa598ede55) | tolB | Tol-Pal system beta propeller repeat protein TolB |
| [OCAR_7292](https://www.genoscope.cns.fr/agc/mage/wwwpkgdb/Info/getInfoLabel.php?id=3485653&wwwpkgdb=fc2733d6468c073f1dd738aa598ede55&nocache=62541e24b6e240f187ac920cab60f9da&dir=&wwwpkgdb=fc2733d6468c073f1dd738aa598ede55) | tolR | protein TolR |
| [OCAR_7293](https://www.genoscope.cns.fr/agc/mage/wwwpkgdb/Info/getInfoLabel.php?id=3485654&wwwpkgdb=fc2733d6468c073f1dd738aa598ede55&nocache=62541e24b6e240f187ac920cab60f9da&dir=&wwwpkgdb=fc2733d6468c073f1dd738aa598ede55) | tolQ | protein TolQ |
| [OCAR_7296](https://www.genoscope.cns.fr/agc/mage/wwwpkgdb/Info/getInfoLabel.php?id=3485656&wwwpkgdb=fc2733d6468c073f1dd738aa598ede55&nocache=62541e24b6e240f187ac920cab60f9da&dir=&wwwpkgdb=fc2733d6468c073f1dd738aa598ede55) | _ | transcriptional regulator, TetR family |
| [OCAR_7299](https://www.genoscope.cns.fr/agc/mage/wwwpkgdb/Info/getInfoLabel.php?id=3485659&wwwpkgdb=fc2733d6468c073f1dd738aa598ede55&nocache=62541e24b6e240f187ac920cab60f9da&dir=&wwwpkgdb=fc2733d6468c073f1dd738aa598ede55) | ruvB | holliday junction DNA helicase RuvB |
| [OCAR_7301](https://www.genoscope.cns.fr/agc/mage/wwwpkgdb/Info/getInfoLabel.php?id=3485661&wwwpkgdb=fc2733d6468c073f1dd738aa598ede55&nocache=62541e24b6e240f187ac920cab60f9da&dir=&wwwpkgdb=fc2733d6468c073f1dd738aa598ede55) | ruvA | Holliday junction ATP-dependent DNA helicase ruvA |
| [OCAR_7302](https://www.genoscope.cns.fr/agc/mage/wwwpkgdb/Info/getInfoLabel.php?id=3485662&wwwpkgdb=fc2733d6468c073f1dd738aa598ede55&nocache=62541e24b6e240f187ac920cab60f9da&dir=&wwwpkgdb=fc2733d6468c073f1dd738aa598ede55) | ruvC | Crossover junction endodeoxyribonuclease ruvC |
| [OCAR_7305](https://www.genoscope.cns.fr/agc/mage/wwwpkgdb/Info/getInfoLabel.php?id=3485665&wwwpkgdb=fc2733d6468c073f1dd738aa598ede55&nocache=62541e24b6e240f187ac920cab60f9da&dir=&wwwpkgdb=fc2733d6468c073f1dd738aa598ede55) | _ | UPF0082 protein OCAR_7305 |
| [OCAR_7306](https://www.genoscope.cns.fr/agc/mage/wwwpkgdb/Info/getInfoLabel.php?id=3485666&wwwpkgdb=fc2733d6468c073f1dd738aa598ede55&nocache=62541e24b6e240f187ac920cab60f9da&dir=&wwwpkgdb=fc2733d6468c073f1dd738aa598ede55) | _ | hypothetical protein |
| [OCAR_7311](https://www.genoscope.cns.fr/agc/mage/wwwpkgdb/Info/getInfoLabel.php?id=3483825&wwwpkgdb=fc2733d6468c073f1dd738aa598ede55&nocache=62541e24b6e240f187ac920cab60f9da&dir=&wwwpkgdb=fc2733d6468c073f1dd738aa598ede55) | gap | glyceraldehyde-3-phosphate dehydrogenase, type I |
| [OCAR_7312](https://www.genoscope.cns.fr/agc/mage/wwwpkgdb/Info/getInfoLabel.php?id=3483826&wwwpkgdb=fc2733d6468c073f1dd738aa598ede55&nocache=62541e24b6e240f187ac920cab60f9da&dir=&wwwpkgdb=fc2733d6468c073f1dd738aa598ede55) | pgk | phosphoglycerate kinase |
| [OCAR_7313](https://www.genoscope.cns.fr/agc/mage/wwwpkgdb/Info/getInfoLabel.php?id=3483827&wwwpkgdb=fc2733d6468c073f1dd738aa598ede55&nocache=62541e24b6e240f187ac920cab60f9da&dir=&wwwpkgdb=fc2733d6468c073f1dd738aa598ede55) | _ | fructose-bisphosphate aldolase class-I |
| [OCAR_7316](https://www.genoscope.cns.fr/agc/mage/wwwpkgdb/Info/getInfoLabel.php?id=3483830&wwwpkgdb=fc2733d6468c073f1dd738aa598ede55&nocache=62541e24b6e240f187ac920cab60f9da&dir=&wwwpkgdb=fc2733d6468c073f1dd738aa598ede55) | _ | inositol-1-monophosphatase (IMPase) |
| [OCAR_7317](https://www.genoscope.cns.fr/agc/mage/wwwpkgdb/Info/getInfoLabel.php?id=3483831&wwwpkgdb=fc2733d6468c073f1dd738aa598ede55&nocache=62541e24b6e240f187ac920cab60f9da&dir=&wwwpkgdb=fc2733d6468c073f1dd738aa598ede55) | _ | MotA/TolQ/ExbB proton channel family |
| [OCAR_7318](https://www.genoscope.cns.fr/agc/mage/wwwpkgdb/Info/getInfoLabel.php?id=3483832&wwwpkgdb=fc2733d6468c073f1dd738aa598ede55&nocache=62541e24b6e240f187ac920cab60f9da&dir=&wwwpkgdb=fc2733d6468c073f1dd738aa598ede55) | _ | flagellar motor protein |
| [OCAR_7320](https://www.genoscope.cns.fr/agc/mage/wwwpkgdb/Info/getInfoLabel.php?id=3485670&wwwpkgdb=fc2733d6468c073f1dd738aa598ede55&nocache=62541e24b6e240f187ac920cab60f9da&dir=&wwwpkgdb=fc2733d6468c073f1dd738aa598ede55) | _ | ABC transporter, permease/ATP-binding protein |
| [OCAR_7321](https://www.genoscope.cns.fr/agc/mage/wwwpkgdb/Info/getInfoLabel.php?id=3483834&wwwpkgdb=fc2733d6468c073f1dd738aa598ede55&nocache=62541e24b6e240f187ac920cab60f9da&dir=&wwwpkgdb=fc2733d6468c073f1dd738aa598ede55) | rpmE | 50S ribosomal protein L31 |
| [OCAR_7324](https://www.genoscope.cns.fr/agc/mage/wwwpkgdb/Info/getInfoLabel.php?id=3483835&wwwpkgdb=fc2733d6468c073f1dd738aa598ede55&nocache=62541e24b6e240f187ac920cab60f9da&dir=&wwwpkgdb=fc2733d6468c073f1dd738aa598ede55) | _ | NAD |
| [OCAR_7325](https://www.genoscope.cns.fr/agc/mage/wwwpkgdb/Info/getInfoLabel.php?id=3483836&wwwpkgdb=fc2733d6468c073f1dd738aa598ede55&nocache=62541e24b6e240f187ac920cab60f9da&dir=&wwwpkgdb=fc2733d6468c073f1dd738aa598ede55) | _ | multi-sensor diguanylate cyclase/phophodiesterase |
| [OCAR_7326](https://www.genoscope.cns.fr/agc/mage/wwwpkgdb/Info/getInfoLabel.php?id=3485673&wwwpkgdb=fc2733d6468c073f1dd738aa598ede55&nocache=62541e24b6e240f187ac920cab60f9da&dir=&wwwpkgdb=fc2733d6468c073f1dd738aa598ede55) | _ | UPF0301 protein OCAR_7326 |
| [OCAR_7328](https://www.genoscope.cns.fr/agc/mage/wwwpkgdb/Info/getInfoLabel.php?id=3483838&wwwpkgdb=fc2733d6468c073f1dd738aa598ede55&nocache=62541e24b6e240f187ac920cab60f9da&dir=&wwwpkgdb=fc2733d6468c073f1dd738aa598ede55) | _ | hybrid peroxiredoxin hyPrx5 |
| [OCAR_7329](https://www.genoscope.cns.fr/agc/mage/wwwpkgdb/Info/getInfoLabel.php?id=3485674&wwwpkgdb=fc2733d6468c073f1dd738aa598ede55&nocache=62541e24b6e240f187ac920cab60f9da&dir=&wwwpkgdb=fc2733d6468c073f1dd738aa598ede55) | rnhA | Ribonuclease H |
| [OCAR_7330](https://www.genoscope.cns.fr/agc/mage/wwwpkgdb/Info/getInfoLabel.php?id=3485675&wwwpkgdb=fc2733d6468c073f1dd738aa598ede55&nocache=62541e24b6e240f187ac920cab60f9da&dir=&wwwpkgdb=fc2733d6468c073f1dd738aa598ede55) | thrB | Homoserine kinase |
| [OCAR_7331](https://www.genoscope.cns.fr/agc/mage/wwwpkgdb/Info/getInfoLabel.php?id=3485676&wwwpkgdb=fc2733d6468c073f1dd738aa598ede55&nocache=62541e24b6e240f187ac920cab60f9da&dir=&wwwpkgdb=fc2733d6468c073f1dd738aa598ede55) | ispH | 4-hydroxy-3-methylbut-2-enyl diphosphate reductase |
| [OCAR_7332](https://www.genoscope.cns.fr/agc/mage/wwwpkgdb/Info/getInfoLabel.php?id=3483839&wwwpkgdb=fc2733d6468c073f1dd738aa598ede55&nocache=62541e24b6e240f187ac920cab60f9da&dir=&wwwpkgdb=fc2733d6468c073f1dd738aa598ede55) | _ | hypothetical protein |
| [OCAR_7336](https://www.genoscope.cns.fr/agc/mage/wwwpkgdb/Info/getInfoLabel.php?id=3485680&wwwpkgdb=fc2733d6468c073f1dd738aa598ede55&nocache=62541e24b6e240f187ac920cab60f9da&dir=&wwwpkgdb=fc2733d6468c073f1dd738aa598ede55) | _ | propionate--CoA ligase (Propionyl-CoA synthetase) |
| [OCAR_7372](https://www.genoscope.cns.fr/agc/mage/wwwpkgdb/Info/getInfoLabel.php?id=3485699&wwwpkgdb=fc2733d6468c073f1dd738aa598ede55&nocache=62541e24b6e240f187ac920cab60f9da&dir=&wwwpkgdb=fc2733d6468c073f1dd738aa598ede55) | _ | DedA family |
| [OCAR_7375](https://www.genoscope.cns.fr/agc/mage/wwwpkgdb/Info/getInfoLabel.php?id=3483857&wwwpkgdb=fc2733d6468c073f1dd738aa598ede55&nocache=62541e24b6e240f187ac920cab60f9da&dir=&wwwpkgdb=fc2733d6468c073f1dd738aa598ede55) | _ | beta-(1-->2)glucan export ATP-binding/permease protein NdvA |
| [OCAR_7381](https://www.genoscope.cns.fr/agc/mage/wwwpkgdb/Info/getInfoLabel.php?id=3485703&wwwpkgdb=fc2733d6468c073f1dd738aa598ede55&nocache=62541e24b6e240f187ac920cab60f9da&dir=&wwwpkgdb=fc2733d6468c073f1dd738aa598ede55) | _ | D-beta-hydroxybutyrate dehydrogenase (bdh) |
| [OCAR_7383](https://www.genoscope.cns.fr/agc/mage/wwwpkgdb/Info/getInfoLabel.php?id=3485705&wwwpkgdb=fc2733d6468c073f1dd738aa598ede55&nocache=62541e24b6e240f187ac920cab60f9da&dir=&wwwpkgdb=fc2733d6468c073f1dd738aa598ede55) | _ | sulfate/thiosulfate import ATP-binding protein CysA |
| [OCAR_7384](https://www.genoscope.cns.fr/agc/mage/wwwpkgdb/Info/getInfoLabel.php?id=3485706&wwwpkgdb=fc2733d6468c073f1dd738aa598ede55&nocache=62541e24b6e240f187ac920cab60f9da&dir=&wwwpkgdb=fc2733d6468c073f1dd738aa598ede55) | cysW | sulfate ABC transporter, permease protein CysW |
| [OCAR_7385](https://www.genoscope.cns.fr/agc/mage/wwwpkgdb/Info/getInfoLabel.php?id=3485707&wwwpkgdb=fc2733d6468c073f1dd738aa598ede55&nocache=62541e24b6e240f187ac920cab60f9da&dir=&wwwpkgdb=fc2733d6468c073f1dd738aa598ede55) | cysT | sulfate ABC transporter, permease protein CysT |
| [OCAR_7386](https://www.genoscope.cns.fr/agc/mage/wwwpkgdb/Info/getInfoLabel.php?id=3485708&wwwpkgdb=fc2733d6468c073f1dd738aa598ede55&nocache=62541e24b6e240f187ac920cab60f9da&dir=&wwwpkgdb=fc2733d6468c073f1dd738aa598ede55) | _ | thiosulfate-binding protein |
| [OCAR_7387](https://www.genoscope.cns.fr/agc/mage/wwwpkgdb/Info/getInfoLabel.php?id=3485709&wwwpkgdb=fc2733d6468c073f1dd738aa598ede55&nocache=62541e24b6e240f187ac920cab60f9da&dir=&wwwpkgdb=fc2733d6468c073f1dd738aa598ede55) | _ | phosphoadenosine phosphosulfate reductase |
| [OCAR_7390](https://www.genoscope.cns.fr/agc/mage/wwwpkgdb/Info/getInfoLabel.php?id=3483863&wwwpkgdb=fc2733d6468c073f1dd738aa598ede55&nocache=62541e24b6e240f187ac920cab60f9da&dir=&wwwpkgdb=fc2733d6468c073f1dd738aa598ede55) | cysD | sulfate adenylyltransferase, small subunit |
| [OCAR_7391](https://www.genoscope.cns.fr/agc/mage/wwwpkgdb/Info/getInfoLabel.php?id=3483864&wwwpkgdb=fc2733d6468c073f1dd738aa598ede55&nocache=62541e24b6e240f187ac920cab60f9da&dir=&wwwpkgdb=fc2733d6468c073f1dd738aa598ede55) | _ | NodQ bifunctional enzyme; Nodulation protein Q |
| [OCAR_7393](https://www.genoscope.cns.fr/agc/mage/wwwpkgdb/Info/getInfoLabel.php?id=3483865&wwwpkgdb=fc2733d6468c073f1dd738aa598ede55&nocache=62541e24b6e240f187ac920cab60f9da&dir=&wwwpkgdb=fc2733d6468c073f1dd738aa598ede55) | glcB | malate synthase G |
| [OCAR_7405](https://www.genoscope.cns.fr/agc/mage/wwwpkgdb/Info/getInfoLabel.php?id=3485718&wwwpkgdb=fc2733d6468c073f1dd738aa598ede55&nocache=62541e24b6e240f187ac920cab60f9da&dir=&wwwpkgdb=fc2733d6468c073f1dd738aa598ede55) | _ | response regulator receiver |
| [OCAR_7407](https://www.genoscope.cns.fr/agc/mage/wwwpkgdb/Info/getInfoLabel.php?id=3483872&wwwpkgdb=fc2733d6468c073f1dd738aa598ede55&nocache=62541e24b6e240f187ac920cab60f9da&dir=&wwwpkgdb=fc2733d6468c073f1dd738aa598ede55) | _ | sigma-24 |
| [OCAR_7411](https://www.genoscope.cns.fr/agc/mage/wwwpkgdb/Info/getInfoLabel.php?id=3483873&wwwpkgdb=fc2733d6468c073f1dd738aa598ede55&nocache=62541e24b6e240f187ac920cab60f9da&dir=&wwwpkgdb=fc2733d6468c073f1dd738aa598ede55) | _ | putative ATP-dependent RNA helicase RhlE |
| [OCAR_7412](https://www.genoscope.cns.fr/agc/mage/wwwpkgdb/Info/getInfoLabel.php?id=3483874&wwwpkgdb=fc2733d6468c073f1dd738aa598ede55&nocache=62541e24b6e240f187ac920cab60f9da&dir=&wwwpkgdb=fc2733d6468c073f1dd738aa598ede55) | infA | translation initiation factor IF-1 |
| [OCAR_7413](https://www.genoscope.cns.fr/agc/mage/wwwpkgdb/Info/getInfoLabel.php?id=3483875&wwwpkgdb=fc2733d6468c073f1dd738aa598ede55&nocache=62541e24b6e240f187ac920cab60f9da&dir=&wwwpkgdb=fc2733d6468c073f1dd738aa598ede55) | _ | hypothetical protein |
| [OCAR_7421](https://www.genoscope.cns.fr/agc/mage/wwwpkgdb/Info/getInfoLabel.php?id=3483880&wwwpkgdb=fc2733d6468c073f1dd738aa598ede55&nocache=62541e24b6e240f187ac920cab60f9da&dir=&wwwpkgdb=fc2733d6468c073f1dd738aa598ede55) | _ | pilus assembly protein |
| [OCAR_7422](https://www.genoscope.cns.fr/agc/mage/wwwpkgdb/Info/getInfoLabel.php?id=3483881&wwwpkgdb=fc2733d6468c073f1dd738aa598ede55&nocache=62541e24b6e240f187ac920cab60f9da&dir=&wwwpkgdb=fc2733d6468c073f1dd738aa598ede55) | _ | CpaF |
| [OCAR_7424](https://www.genoscope.cns.fr/agc/mage/wwwpkgdb/Info/getInfoLabel.php?id=3483883&wwwpkgdb=fc2733d6468c073f1dd738aa598ede55&nocache=62541e24b6e240f187ac920cab60f9da&dir=&wwwpkgdb=fc2733d6468c073f1dd738aa598ede55) | _ | type II secretion system protein |
| [OCAR_7426](https://www.genoscope.cns.fr/agc/mage/wwwpkgdb/Info/getInfoLabel.php?id=3483884&wwwpkgdb=fc2733d6468c073f1dd738aa598ede55&nocache=62541e24b6e240f187ac920cab60f9da&dir=&wwwpkgdb=fc2733d6468c073f1dd738aa598ede55) | _ | leucine aminopeptidase |
| [OCAR_7428](https://www.genoscope.cns.fr/agc/mage/wwwpkgdb/Info/getInfoLabel.php?id=3485726&wwwpkgdb=fc2733d6468c073f1dd738aa598ede55&nocache=62541e24b6e240f187ac920cab60f9da&dir=&wwwpkgdb=fc2733d6468c073f1dd738aa598ede55) | _ | glutathione import ATP-binding protein GsiA |
| [OCAR_7429](https://www.genoscope.cns.fr/agc/mage/wwwpkgdb/Info/getInfoLabel.php?id=3485727&wwwpkgdb=fc2733d6468c073f1dd738aa598ede55&nocache=62541e24b6e240f187ac920cab60f9da&dir=&wwwpkgdb=fc2733d6468c073f1dd738aa598ede55) | _ | inner membrane ABC transporter permease protein YejE |
| [OCAR_7430](https://www.genoscope.cns.fr/agc/mage/wwwpkgdb/Info/getInfoLabel.php?id=3485728&wwwpkgdb=fc2733d6468c073f1dd738aa598ede55&nocache=62541e24b6e240f187ac920cab60f9da&dir=&wwwpkgdb=fc2733d6468c073f1dd738aa598ede55) | _ | binding-protein-dependent transport systems inner membrane component |
| [OCAR_7431](https://www.genoscope.cns.fr/agc/mage/wwwpkgdb/Info/getInfoLabel.php?id=3485729&wwwpkgdb=fc2733d6468c073f1dd738aa598ede55&nocache=62541e24b6e240f187ac920cab60f9da&dir=&wwwpkgdb=fc2733d6468c073f1dd738aa598ede55) | _ | bacterial extracellular solute-binding proteins, family 5 |
| [OCAR_7435](https://www.genoscope.cns.fr/agc/mage/wwwpkgdb/Info/getInfoLabel.php?id=3483887&wwwpkgdb=fc2733d6468c073f1dd738aa598ede55&nocache=62541e24b6e240f187ac920cab60f9da&dir=&wwwpkgdb=fc2733d6468c073f1dd738aa598ede55) | kdsB | 3-deoxy-manno-octulosonate cytidylyltransferase |
| [OCAR_7436](https://www.genoscope.cns.fr/agc/mage/wwwpkgdb/Info/getInfoLabel.php?id=3483888&wwwpkgdb=fc2733d6468c073f1dd738aa598ede55&nocache=62541e24b6e240f187ac920cab60f9da&dir=&wwwpkgdb=fc2733d6468c073f1dd738aa598ede55) | _ | prephenate dehydratase |
| [OCAR_7438](https://www.genoscope.cns.fr/agc/mage/wwwpkgdb/Info/getInfoLabel.php?id=3483889&wwwpkgdb=fc2733d6468c073f1dd738aa598ede55&nocache=62541e24b6e240f187ac920cab60f9da&dir=&wwwpkgdb=fc2733d6468c073f1dd738aa598ede55) | metF | 5,10-methylenetetrahydrofolate reductase |
| [OCAR_7439](https://www.genoscope.cns.fr/agc/mage/wwwpkgdb/Info/getInfoLabel.php?id=3483890&wwwpkgdb=fc2733d6468c073f1dd738aa598ede55&nocache=62541e24b6e240f187ac920cab60f9da&dir=&wwwpkgdb=fc2733d6468c073f1dd738aa598ede55) | metH | methionine synthase |
| [OCAR_7441](https://www.genoscope.cns.fr/agc/mage/wwwpkgdb/Info/getInfoLabel.php?id=3485733&wwwpkgdb=fc2733d6468c073f1dd738aa598ede55&nocache=62541e24b6e240f187ac920cab60f9da&dir=&wwwpkgdb=fc2733d6468c073f1dd738aa598ede55) | glpK | Glycerol kinase |
| [OCAR_7442](https://www.genoscope.cns.fr/agc/mage/wwwpkgdb/Info/getInfoLabel.php?id=3485734&wwwpkgdb=fc2733d6468c073f1dd738aa598ede55&nocache=62541e24b6e240f187ac920cab60f9da&dir=&wwwpkgdb=fc2733d6468c073f1dd738aa598ede55) | _ | 3-ketoacyl-CoA thiolase B, peroxisomal |
| [OCAR_7443](https://www.genoscope.cns.fr/agc/mage/wwwpkgdb/Info/getInfoLabel.php?id=3483892&wwwpkgdb=fc2733d6468c073f1dd738aa598ede55&nocache=62541e24b6e240f187ac920cab60f9da&dir=&wwwpkgdb=fc2733d6468c073f1dd738aa598ede55) | _ | putative hydroxypyruvate reductase |
| [OCAR_7461](https://www.genoscope.cns.fr/agc/mage/wwwpkgdb/Info/getInfoLabel.php?id=3485744&wwwpkgdb=fc2733d6468c073f1dd738aa598ede55&nocache=62541e24b6e240f187ac920cab60f9da&dir=&wwwpkgdb=fc2733d6468c073f1dd738aa598ede55) | _ | LemA |
| [OCAR_7527](https://www.genoscope.cns.fr/agc/mage/wwwpkgdb/Info/getInfoLabel.php?id=3485790&wwwpkgdb=fc2733d6468c073f1dd738aa598ede55&nocache=62541e24b6e240f187ac920cab60f9da&dir=&wwwpkgdb=fc2733d6468c073f1dd738aa598ede55) | _ | histidine kinase |
| [OCAR_7528](https://www.genoscope.cns.fr/agc/mage/wwwpkgdb/Info/getInfoLabel.php?id=3483921&wwwpkgdb=fc2733d6468c073f1dd738aa598ede55&nocache=62541e24b6e240f187ac920cab60f9da&dir=&wwwpkgdb=fc2733d6468c073f1dd738aa598ede55) | rpmH | 50S ribosomal protein L34 |
| [OCAR_7531](https://www.genoscope.cns.fr/agc/mage/wwwpkgdb/Info/getInfoLabel.php?id=3483924&wwwpkgdb=fc2733d6468c073f1dd738aa598ede55&nocache=62541e24b6e240f187ac920cab60f9da&dir=&wwwpkgdb=fc2733d6468c073f1dd738aa598ede55) | engB | Probable GTP-binding protein engB |
| [OCAR_7533](https://www.genoscope.cns.fr/agc/mage/wwwpkgdb/Info/getInfoLabel.php?id=3483926&wwwpkgdb=fc2733d6468c073f1dd738aa598ede55&nocache=62541e24b6e240f187ac920cab60f9da&dir=&wwwpkgdb=fc2733d6468c073f1dd738aa598ede55) | argB | Acetylglutamate kinase |
| [OCAR_7534](https://www.genoscope.cns.fr/agc/mage/wwwpkgdb/Info/getInfoLabel.php?id=3483927&wwwpkgdb=fc2733d6468c073f1dd738aa598ede55&nocache=62541e24b6e240f187ac920cab60f9da&dir=&wwwpkgdb=fc2733d6468c073f1dd738aa598ede55) | _ | pyrimidine 5'-nucleotidase |
| [OCAR_7535](https://www.genoscope.cns.fr/agc/mage/wwwpkgdb/Info/getInfoLabel.php?id=3483928&wwwpkgdb=fc2733d6468c073f1dd738aa598ede55&nocache=62541e24b6e240f187ac920cab60f9da&dir=&wwwpkgdb=fc2733d6468c073f1dd738aa598ede55) | dapD | 2,3,4,5-tetrahydropyridine-2,6-dicarboxylate N-succinyltransferase |
| [OCAR_7538](https://www.genoscope.cns.fr/agc/mage/wwwpkgdb/Info/getInfoLabel.php?id=3485791&wwwpkgdb=fc2733d6468c073f1dd738aa598ede55&nocache=62541e24b6e240f187ac920cab60f9da&dir=&wwwpkgdb=fc2733d6468c073f1dd738aa598ede55) | truA | tRNA pseudouridine synthase A |
| [OCAR_7539](https://www.genoscope.cns.fr/agc/mage/wwwpkgdb/Info/getInfoLabel.php?id=3485792&wwwpkgdb=fc2733d6468c073f1dd738aa598ede55&nocache=62541e24b6e240f187ac920cab60f9da&dir=&wwwpkgdb=fc2733d6468c073f1dd738aa598ede55) | fmt | Methionyl-tRNA formyltransferase |
| [OCAR_7540](https://www.genoscope.cns.fr/agc/mage/wwwpkgdb/Info/getInfoLabel.php?id=3485793&wwwpkgdb=fc2733d6468c073f1dd738aa598ede55&nocache=62541e24b6e240f187ac920cab60f9da&dir=&wwwpkgdb=fc2733d6468c073f1dd738aa598ede55) | def | Peptide deformylase |
| [OCAR_7541](https://www.genoscope.cns.fr/agc/mage/wwwpkgdb/Info/getInfoLabel.php?id=3483931&wwwpkgdb=fc2733d6468c073f1dd738aa598ede55&nocache=62541e24b6e240f187ac920cab60f9da&dir=&wwwpkgdb=fc2733d6468c073f1dd738aa598ede55) | _ | RmuC domain protein |
| [OCAR_7543](https://www.genoscope.cns.fr/agc/mage/wwwpkgdb/Info/getInfoLabel.php?id=3485794&wwwpkgdb=fc2733d6468c073f1dd738aa598ede55&nocache=62541e24b6e240f187ac920cab60f9da&dir=&wwwpkgdb=fc2733d6468c073f1dd738aa598ede55) | recR | recombination protein RecR |
| [OCAR_7544](https://www.genoscope.cns.fr/agc/mage/wwwpkgdb/Info/getInfoLabel.php?id=3485795&wwwpkgdb=fc2733d6468c073f1dd738aa598ede55&nocache=62541e24b6e240f187ac920cab60f9da&dir=&wwwpkgdb=fc2733d6468c073f1dd738aa598ede55) | _ | UPF0133 protein OCAR_7544 |
| [OCAR_7545](https://www.genoscope.cns.fr/agc/mage/wwwpkgdb/Info/getInfoLabel.php?id=3485796&wwwpkgdb=fc2733d6468c073f1dd738aa598ede55&nocache=62541e24b6e240f187ac920cab60f9da&dir=&wwwpkgdb=fc2733d6468c073f1dd738aa598ede55) | _ | DNA polymerase III, subunits gamma and tau |
| [OCAR_7548](https://www.genoscope.cns.fr/agc/mage/wwwpkgdb/Info/getInfoLabel.php?id=3483935&wwwpkgdb=fc2733d6468c073f1dd738aa598ede55&nocache=62541e24b6e240f187ac920cab60f9da&dir=&wwwpkgdb=fc2733d6468c073f1dd738aa598ede55) | _ | glutamate uptake regulatory protein |
| [OCAR_7560](https://www.genoscope.cns.fr/agc/mage/wwwpkgdb/Info/getInfoLabel.php?id=3485802&wwwpkgdb=fc2733d6468c073f1dd738aa598ede55&nocache=62541e24b6e240f187ac920cab60f9da&dir=&wwwpkgdb=fc2733d6468c073f1dd738aa598ede55) | _ | phosphoglucomutase/phosphomannomutase |
| [OCAR_7564](https://www.genoscope.cns.fr/agc/mage/wwwpkgdb/Info/getInfoLabel.php?id=3485804&wwwpkgdb=fc2733d6468c073f1dd738aa598ede55&nocache=62541e24b6e240f187ac920cab60f9da&dir=&wwwpkgdb=fc2733d6468c073f1dd738aa598ede55) | hrpB | ATP-dependent helicase HrpB |
| [OCAR_7565](https://www.genoscope.cns.fr/agc/mage/wwwpkgdb/Info/getInfoLabel.php?id=3485805&wwwpkgdb=fc2733d6468c073f1dd738aa598ede55&nocache=62541e24b6e240f187ac920cab60f9da&dir=&wwwpkgdb=fc2733d6468c073f1dd738aa598ede55) | _ | udp-glucose 6-dehydrogenase |
| [OCAR_7567](https://www.genoscope.cns.fr/agc/mage/wwwpkgdb/Info/getInfoLabel.php?id=3483944&wwwpkgdb=fc2733d6468c073f1dd738aa598ede55&nocache=62541e24b6e240f187ac920cab60f9da&dir=&wwwpkgdb=fc2733d6468c073f1dd738aa598ede55) | _ | DNA polymerase I (POL I) |
| [OCAR_7569](https://www.genoscope.cns.fr/agc/mage/wwwpkgdb/Info/getInfoLabel.php?id=3483946&wwwpkgdb=fc2733d6468c073f1dd738aa598ede55&nocache=62541e24b6e240f187ac920cab60f9da&dir=&wwwpkgdb=fc2733d6468c073f1dd738aa598ede55) | pyrE | orotate phosphoribosyltransferase |
| [OCAR_7573](https://www.genoscope.cns.fr/agc/mage/wwwpkgdb/Info/getInfoLabel.php?id=3485807&wwwpkgdb=fc2733d6468c073f1dd738aa598ede55&nocache=62541e24b6e240f187ac920cab60f9da&dir=&wwwpkgdb=fc2733d6468c073f1dd738aa598ede55) | pckA | Phosphoenolpyruvate carboxykinase [ATP] |
| [OCAR_7575](https://www.genoscope.cns.fr/agc/mage/wwwpkgdb/Info/getInfoLabel.php?id=3483949&wwwpkgdb=fc2733d6468c073f1dd738aa598ede55&nocache=62541e24b6e240f187ac920cab60f9da&dir=&wwwpkgdb=fc2733d6468c073f1dd738aa598ede55) | _ | transcriptional regulatory protein ChvI |
| [OCAR_7576](https://www.genoscope.cns.fr/agc/mage/wwwpkgdb/Info/getInfoLabel.php?id=3483950&wwwpkgdb=fc2733d6468c073f1dd738aa598ede55&nocache=62541e24b6e240f187ac920cab60f9da&dir=&wwwpkgdb=fc2733d6468c073f1dd738aa598ede55) | _ | sensor protein ChvG |
| [OCAR_7578](https://www.genoscope.cns.fr/agc/mage/wwwpkgdb/Info/getInfoLabel.php?id=3483952&wwwpkgdb=fc2733d6468c073f1dd738aa598ede55&nocache=62541e24b6e240f187ac920cab60f9da&dir=&wwwpkgdb=fc2733d6468c073f1dd738aa598ede55) | _ | PTS system permease |
| [OCAR_7580](https://www.genoscope.cns.fr/agc/mage/wwwpkgdb/Info/getInfoLabel.php?id=3483954&wwwpkgdb=fc2733d6468c073f1dd738aa598ede55&nocache=62541e24b6e240f187ac920cab60f9da&dir=&wwwpkgdb=fc2733d6468c073f1dd738aa598ede55) | lepA | GTP-binding protein lepA |
| [OCAR_7586](https://www.genoscope.cns.fr/agc/mage/wwwpkgdb/Info/getInfoLabel.php?id=3483960&wwwpkgdb=fc2733d6468c073f1dd738aa598ede55&nocache=62541e24b6e240f187ac920cab60f9da&dir=&wwwpkgdb=fc2733d6468c073f1dd738aa598ede55) | modA | molybdate ABC transporter, periplasmic molybdate-binding protein |
| [OCAR_7587](https://www.genoscope.cns.fr/agc/mage/wwwpkgdb/Info/getInfoLabel.php?id=3483961&wwwpkgdb=fc2733d6468c073f1dd738aa598ede55&nocache=62541e24b6e240f187ac920cab60f9da&dir=&wwwpkgdb=fc2733d6468c073f1dd738aa598ede55) | modB | molybdate ABC transporter, permease protein |
| [OCAR_7588](https://www.genoscope.cns.fr/agc/mage/wwwpkgdb/Info/getInfoLabel.php?id=3483962&wwwpkgdb=fc2733d6468c073f1dd738aa598ede55&nocache=62541e24b6e240f187ac920cab60f9da&dir=&wwwpkgdb=fc2733d6468c073f1dd738aa598ede55) | _ | molybdenum import ATP-binding protein ModC 1 |
| [OCAR_7592](https://www.genoscope.cns.fr/agc/mage/wwwpkgdb/Info/getInfoLabel.php?id=3485810&wwwpkgdb=fc2733d6468c073f1dd738aa598ede55&nocache=62541e24b6e240f187ac920cab60f9da&dir=&wwwpkgdb=fc2733d6468c073f1dd738aa598ede55) | _ | cell volume regulation protein A |
| [OCAR_7594](https://www.genoscope.cns.fr/agc/mage/wwwpkgdb/Info/getInfoLabel.php?id=3483966&wwwpkgdb=fc2733d6468c073f1dd738aa598ede55&nocache=62541e24b6e240f187ac920cab60f9da&dir=&wwwpkgdb=fc2733d6468c073f1dd738aa598ede55) | map | methionine aminopeptidase, type I |
| [OCAR_7595](https://www.genoscope.cns.fr/agc/mage/wwwpkgdb/Info/getInfoLabel.php?id=3483967&wwwpkgdb=fc2733d6468c073f1dd738aa598ede55&nocache=62541e24b6e240f187ac920cab60f9da&dir=&wwwpkgdb=fc2733d6468c073f1dd738aa598ede55) | _ | DNA repair protein RadC |
| [OCAR_7618](https://www.genoscope.cns.fr/agc/mage/wwwpkgdb/Info/getInfoLabel.php?id=3485923&wwwpkgdb=fc2733d6468c073f1dd738aa598ede55&nocache=62541e24b6e240f187ac920cab60f9da&dir=&wwwpkgdb=fc2733d6468c073f1dd738aa598ede55) | _ | heavy metal efflux pump; nonfunctional due to frameshift |
| [OCAR_7636](https://www.genoscope.cns.fr/agc/mage/wwwpkgdb/Info/getInfoLabel.php?id=3485838&wwwpkgdb=fc2733d6468c073f1dd738aa598ede55&nocache=62541e24b6e240f187ac920cab60f9da&dir=&wwwpkgdb=fc2733d6468c073f1dd738aa598ede55) | _ | hypothetical protein |
| [OCAR_7637](https://www.genoscope.cns.fr/agc/mage/wwwpkgdb/Info/getInfoLabel.php?id=3483978&wwwpkgdb=fc2733d6468c073f1dd738aa598ede55&nocache=62541e24b6e240f187ac920cab60f9da&dir=&wwwpkgdb=fc2733d6468c073f1dd738aa598ede55) | _ | cation-transporting ATPase PacS |
| [OCAR_7726](https://www.genoscope.cns.fr/agc/mage/wwwpkgdb/Info/getInfoLabel.php?id=3485890&wwwpkgdb=fc2733d6468c073f1dd738aa598ede55&nocache=62541e24b6e240f187ac920cab60f9da&dir=&wwwpkgdb=fc2733d6468c073f1dd738aa598ede55) | _ | cation efflux system protein CzcA |
| [OCAR_7728](https://www.genoscope.cns.fr/agc/mage/wwwpkgdb/Info/getInfoLabel.php?id=3485892&wwwpkgdb=fc2733d6468c073f1dd738aa598ede55&nocache=62541e24b6e240f187ac920cab60f9da&dir=&wwwpkgdb=fc2733d6468c073f1dd738aa598ede55) | _ | outer membrane efflux protein |
| [OCAR_7738](https://www.genoscope.cns.fr/agc/mage/wwwpkgdb/Info/getInfoLabel.php?id=3485900&wwwpkgdb=fc2733d6468c073f1dd738aa598ede55&nocache=62541e24b6e240f187ac920cab60f9da&dir=&wwwpkgdb=fc2733d6468c073f1dd738aa598ede55) | _ | heavy metal efflux pump, CzcA family |
| [OCAR_7777](https://www.genoscope.cns.fr/agc/mage/wwwpkgdb/Info/getInfoLabel.php?id=3484041&wwwpkgdb=fc2733d6468c073f1dd738aa598ede55&nocache=62541e24b6e240f187ac920cab60f9da&dir=&wwwpkgdb=fc2733d6468c073f1dd738aa598ede55) | _ | cation efflux system protein CusA |
