# Supplementary material for: Complete genome and comparative analysis of the chemolithoautotrophic bacterium Oligotropha carboxidovorans OM5
Source: BMC Genomics. 2010 Sep 23;11:511. doi: 10.1186/1471-2164-11-511 (PMC3091675; doi:10.1186/1471-2164-11-511)
Supplement: Additional file 2 — Proteins unique to O. carboxidovorans OM5 as compared to N. hamburgensis X14, and Bradyrhizobium spp. USDA110. [file 1471-2164-11-511-S2.DOC]

**Additional file 2.** Proteins unique to *O. carboxidovorans* OM5 as compared to *N. hamburgensis* X14, and *Bradyrhizobium* spp. USDA110 as shown by the Phyloprofile tool of Oligotroscope.

| **Label** | **Gene** | **Product** |
| --- | --- | --- |
| [OCAR_4009](https://www.genoscope.cns.fr/agc/mage/wwwpkgdb/Info/getInfoLabel.php?id=3484052&wwwpkgdb=fc2733d6468c073f1dd738aa598ede55&nocache=a9a895ad49f0f55c362d050dc1ad3fa3&dir=&wwwpkgdb=fc2733d6468c073f1dd738aa598ede55) | _ | phage transcriptional regulator, AlpA |
| [OCAR_4011](https://www.genoscope.cns.fr/agc/mage/wwwpkgdb/Info/getInfoLabel.php?id=3484053&wwwpkgdb=fc2733d6468c073f1dd738aa598ede55&nocache=a9a895ad49f0f55c362d050dc1ad3fa3&dir=&wwwpkgdb=fc2733d6468c073f1dd738aa598ede55) | _ | hypothetical protein |
| [OCAR_0012](https://www.genoscope.cns.fr/agc/mage/wwwpkgdb/Info/getInfoLabel.php?id=3482111&wwwpkgdb=fc2733d6468c073f1dd738aa598ede55&nocache=a9a895ad49f0f55c362d050dc1ad3fa3&dir=&wwwpkgdb=fc2733d6468c073f1dd738aa598ede55) | _ | _ |
| [OCAR_4012](https://www.genoscope.cns.fr/agc/mage/wwwpkgdb/Info/getInfoLabel.php?id=3484054&wwwpkgdb=fc2733d6468c073f1dd738aa598ede55&nocache=a9a895ad49f0f55c362d050dc1ad3fa3&dir=&wwwpkgdb=fc2733d6468c073f1dd738aa598ede55) | _ | hypothetical protein |
| [OCAR_4015](https://www.genoscope.cns.fr/agc/mage/wwwpkgdb/Info/getInfoLabel.php?id=3482140&wwwpkgdb=fc2733d6468c073f1dd738aa598ede55&nocache=a9a895ad49f0f55c362d050dc1ad3fa3&dir=&wwwpkgdb=fc2733d6468c073f1dd738aa598ede55) | _ | hypothetical protein |
| [OCAR_4016](https://www.genoscope.cns.fr/agc/mage/wwwpkgdb/Info/getInfoLabel.php?id=3484057&wwwpkgdb=fc2733d6468c073f1dd738aa598ede55&nocache=a9a895ad49f0f55c362d050dc1ad3fa3&dir=&wwwpkgdb=fc2733d6468c073f1dd738aa598ede55) | _ | peptidase S41 |
| [OCAR_4021](https://www.genoscope.cns.fr/agc/mage/wwwpkgdb/Info/getInfoLabel.php?id=3484061&wwwpkgdb=fc2733d6468c073f1dd738aa598ede55&nocache=a9a895ad49f0f55c362d050dc1ad3fa3&dir=&wwwpkgdb=fc2733d6468c073f1dd738aa598ede55) | _ | twin-arginine translocation pathway signal |
| [OCAR_4022](https://www.genoscope.cns.fr/agc/mage/wwwpkgdb/Info/getInfoLabel.php?id=3482142&wwwpkgdb=fc2733d6468c073f1dd738aa598ede55&nocache=a9a895ad49f0f55c362d050dc1ad3fa3&dir=&wwwpkgdb=fc2733d6468c073f1dd738aa598ede55) | _ | hypothetical protein |
| [OCAR_4027](https://www.genoscope.cns.fr/agc/mage/wwwpkgdb/Info/getInfoLabel.php?id=3484066&wwwpkgdb=fc2733d6468c073f1dd738aa598ede55&nocache=a9a895ad49f0f55c362d050dc1ad3fa3&dir=&wwwpkgdb=fc2733d6468c073f1dd738aa598ede55) | arsH | arsenical resistance protein ArsH |
| [OCAR_4032](https://www.genoscope.cns.fr/agc/mage/wwwpkgdb/Info/getInfoLabel.php?id=3484071&wwwpkgdb=fc2733d6468c073f1dd738aa598ede55&nocache=a9a895ad49f0f55c362d050dc1ad3fa3&dir=&wwwpkgdb=fc2733d6468c073f1dd738aa598ede55) | _ | hypothetical protein |
| [OCAR_4033](https://www.genoscope.cns.fr/agc/mage/wwwpkgdb/Info/getInfoLabel.php?id=3484072&wwwpkgdb=fc2733d6468c073f1dd738aa598ede55&nocache=a9a895ad49f0f55c362d050dc1ad3fa3&dir=&wwwpkgdb=fc2733d6468c073f1dd738aa598ede55) | _ | hypothetical protein |
| [OCAR_4035](https://www.genoscope.cns.fr/agc/mage/wwwpkgdb/Info/getInfoLabel.php?id=3484074&wwwpkgdb=fc2733d6468c073f1dd738aa598ede55&nocache=a9a895ad49f0f55c362d050dc1ad3fa3&dir=&wwwpkgdb=fc2733d6468c073f1dd738aa598ede55) | _ | hypothetical protein |
| [OCAR_4037](https://www.genoscope.cns.fr/agc/mage/wwwpkgdb/Info/getInfoLabel.php?id=3484075&wwwpkgdb=fc2733d6468c073f1dd738aa598ede55&nocache=a9a895ad49f0f55c362d050dc1ad3fa3&dir=&wwwpkgdb=fc2733d6468c073f1dd738aa598ede55) | _ | hypothetical protein |
| [OCAR_4038](https://www.genoscope.cns.fr/agc/mage/wwwpkgdb/Info/getInfoLabel.php?id=3484076&wwwpkgdb=fc2733d6468c073f1dd738aa598ede55&nocache=a9a895ad49f0f55c362d050dc1ad3fa3&dir=&wwwpkgdb=fc2733d6468c073f1dd738aa598ede55) | _ | hypothetical protein |
| [OCAR_4039](https://www.genoscope.cns.fr/agc/mage/wwwpkgdb/Info/getInfoLabel.php?id=3484077&wwwpkgdb=fc2733d6468c073f1dd738aa598ede55&nocache=a9a895ad49f0f55c362d050dc1ad3fa3&dir=&wwwpkgdb=fc2733d6468c073f1dd738aa598ede55) | _ | hypothetical protein |
| [OCAR_0046](https://www.genoscope.cns.fr/agc/mage/wwwpkgdb/Info/getInfoLabel.php?id=3482109&wwwpkgdb=fc2733d6468c073f1dd738aa598ede55&nocache=a9a895ad49f0f55c362d050dc1ad3fa3&dir=&wwwpkgdb=fc2733d6468c073f1dd738aa598ede55) | _ | _ |
| [OCAR_4067](https://www.genoscope.cns.fr/agc/mage/wwwpkgdb/Info/getInfoLabel.php?id=3484094&wwwpkgdb=fc2733d6468c073f1dd738aa598ede55&nocache=a9a895ad49f0f55c362d050dc1ad3fa3&dir=&wwwpkgdb=fc2733d6468c073f1dd738aa598ede55) | _ | hypothetical protein |
| [OCAR_0070](https://www.genoscope.cns.fr/agc/mage/wwwpkgdb/Info/getInfoLabel.php?id=3482108&wwwpkgdb=fc2733d6468c073f1dd738aa598ede55&nocache=a9a895ad49f0f55c362d050dc1ad3fa3&dir=&wwwpkgdb=fc2733d6468c073f1dd738aa598ede55) | _ | _ |
| [OCAR_4081](https://www.genoscope.cns.fr/agc/mage/wwwpkgdb/Info/getInfoLabel.php?id=3482160&wwwpkgdb=fc2733d6468c073f1dd738aa598ede55&nocache=a9a895ad49f0f55c362d050dc1ad3fa3&dir=&wwwpkgdb=fc2733d6468c073f1dd738aa598ede55) | _ | hypothetical protein |
| [OCAR_0081](https://www.genoscope.cns.fr/agc/mage/wwwpkgdb/Info/getInfoLabel.php?id=3482107&wwwpkgdb=fc2733d6468c073f1dd738aa598ede55&nocache=a9a895ad49f0f55c362d050dc1ad3fa3&dir=&wwwpkgdb=fc2733d6468c073f1dd738aa598ede55) | _ | _ |
| [OCAR_4084](https://www.genoscope.cns.fr/agc/mage/wwwpkgdb/Info/getInfoLabel.php?id=3484104&wwwpkgdb=fc2733d6468c073f1dd738aa598ede55&nocache=a9a895ad49f0f55c362d050dc1ad3fa3&dir=&wwwpkgdb=fc2733d6468c073f1dd738aa598ede55) | _ | hypothetical protein |
| [OCAR_0086](https://www.genoscope.cns.fr/agc/mage/wwwpkgdb/Info/getInfoLabel.php?id=3482106&wwwpkgdb=fc2733d6468c073f1dd738aa598ede55&nocache=a9a895ad49f0f55c362d050dc1ad3fa3&dir=&wwwpkgdb=fc2733d6468c073f1dd738aa598ede55) | _ | _ |
| [OCAR_4096](https://www.genoscope.cns.fr/agc/mage/wwwpkgdb/Info/getInfoLabel.php?id=3482169&wwwpkgdb=fc2733d6468c073f1dd738aa598ede55&nocache=a9a895ad49f0f55c362d050dc1ad3fa3&dir=&wwwpkgdb=fc2733d6468c073f1dd738aa598ede55) | _ | hypothetical protein |
| [OCAR_4109](https://www.genoscope.cns.fr/agc/mage/wwwpkgdb/Info/getInfoLabel.php?id=3482178&wwwpkgdb=fc2733d6468c073f1dd738aa598ede55&nocache=a9a895ad49f0f55c362d050dc1ad3fa3&dir=&wwwpkgdb=fc2733d6468c073f1dd738aa598ede55) | _ | methyltransferase, FkbM family |
| [OCAR_4111](https://www.genoscope.cns.fr/agc/mage/wwwpkgdb/Info/getInfoLabel.php?id=3484115&wwwpkgdb=fc2733d6468c073f1dd738aa598ede55&nocache=a9a895ad49f0f55c362d050dc1ad3fa3&dir=&wwwpkgdb=fc2733d6468c073f1dd738aa598ede55) | _ | hypothetical protein |
| [OCAR_4128](https://www.genoscope.cns.fr/agc/mage/wwwpkgdb/Info/getInfoLabel.php?id=3484126&wwwpkgdb=fc2733d6468c073f1dd738aa598ede55&nocache=a9a895ad49f0f55c362d050dc1ad3fa3&dir=&wwwpkgdb=fc2733d6468c073f1dd738aa598ede55) | _ | hypothetical protein |
| [OCAR_0129](https://www.genoscope.cns.fr/agc/mage/wwwpkgdb/Info/getInfoLabel.php?id=3482105&wwwpkgdb=fc2733d6468c073f1dd738aa598ede55&nocache=a9a895ad49f0f55c362d050dc1ad3fa3&dir=&wwwpkgdb=fc2733d6468c073f1dd738aa598ede55) | _ | _ |
| [OCAR_4135](https://www.genoscope.cns.fr/agc/mage/wwwpkgdb/Info/getInfoLabel.php?id=3482189&wwwpkgdb=fc2733d6468c073f1dd738aa598ede55&nocache=a9a895ad49f0f55c362d050dc1ad3fa3&dir=&wwwpkgdb=fc2733d6468c073f1dd738aa598ede55) | _ | hypothetical protein |
| [OCAR_4136](https://www.genoscope.cns.fr/agc/mage/wwwpkgdb/Info/getInfoLabel.php?id=3482190&wwwpkgdb=fc2733d6468c073f1dd738aa598ede55&nocache=a9a895ad49f0f55c362d050dc1ad3fa3&dir=&wwwpkgdb=fc2733d6468c073f1dd738aa598ede55) | _ | putative heme oxygenase |
| [OCAR_4140](https://www.genoscope.cns.fr/agc/mage/wwwpkgdb/Info/getInfoLabel.php?id=3482193&wwwpkgdb=fc2733d6468c073f1dd738aa598ede55&nocache=a9a895ad49f0f55c362d050dc1ad3fa3&dir=&wwwpkgdb=fc2733d6468c073f1dd738aa598ede55) | _ | hypothetical protein |
| [OCAR_4143](https://www.genoscope.cns.fr/agc/mage/wwwpkgdb/Info/getInfoLabel.php?id=3482196&wwwpkgdb=fc2733d6468c073f1dd738aa598ede55&nocache=a9a895ad49f0f55c362d050dc1ad3fa3&dir=&wwwpkgdb=fc2733d6468c073f1dd738aa598ede55) | _ | site-specific recombinase, phage integrase family |
| [OCAR_0140](https://www.genoscope.cns.fr/agc/mage/wwwpkgdb/Info/getInfoLabel.php?id=3482104&wwwpkgdb=fc2733d6468c073f1dd738aa598ede55&nocache=a9a895ad49f0f55c362d050dc1ad3fa3&dir=&wwwpkgdb=fc2733d6468c073f1dd738aa598ede55) | _ | _ |
| [OCAR_0141](https://www.genoscope.cns.fr/agc/mage/wwwpkgdb/Info/getInfoLabel.php?id=3482103&wwwpkgdb=fc2733d6468c073f1dd738aa598ede55&nocache=a9a895ad49f0f55c362d050dc1ad3fa3&dir=&wwwpkgdb=fc2733d6468c073f1dd738aa598ede55) | _ | _ |
| [OCAR_4144](https://www.genoscope.cns.fr/agc/mage/wwwpkgdb/Info/getInfoLabel.php?id=3482197&wwwpkgdb=fc2733d6468c073f1dd738aa598ede55&nocache=a9a895ad49f0f55c362d050dc1ad3fa3&dir=&wwwpkgdb=fc2733d6468c073f1dd738aa598ede55) | _ | hypothetical protein |
| [OCAR_4145](https://www.genoscope.cns.fr/agc/mage/wwwpkgdb/Info/getInfoLabel.php?id=3484130&wwwpkgdb=fc2733d6468c073f1dd738aa598ede55&nocache=a9a895ad49f0f55c362d050dc1ad3fa3&dir=&wwwpkgdb=fc2733d6468c073f1dd738aa598ede55) | _ | C-5 cytosine-specific DNA methylase |
| [OCAR_4146](https://www.genoscope.cns.fr/agc/mage/wwwpkgdb/Info/getInfoLabel.php?id=3482198&wwwpkgdb=fc2733d6468c073f1dd738aa598ede55&nocache=a9a895ad49f0f55c362d050dc1ad3fa3&dir=&wwwpkgdb=fc2733d6468c073f1dd738aa598ede55) | _ | helicase |
| [OCAR_0145](https://www.genoscope.cns.fr/agc/mage/wwwpkgdb/Info/getInfoLabel.php?id=3482102&wwwpkgdb=fc2733d6468c073f1dd738aa598ede55&nocache=a9a895ad49f0f55c362d050dc1ad3fa3&dir=&wwwpkgdb=fc2733d6468c073f1dd738aa598ede55) | _ | _ |
| [OCAR_4147](https://www.genoscope.cns.fr/agc/mage/wwwpkgdb/Info/getInfoLabel.php?id=3482199&wwwpkgdb=fc2733d6468c073f1dd738aa598ede55&nocache=a9a895ad49f0f55c362d050dc1ad3fa3&dir=&wwwpkgdb=fc2733d6468c073f1dd738aa598ede55) | _ | hypothetical protein |
| [OCAR_4148](https://www.genoscope.cns.fr/agc/mage/wwwpkgdb/Info/getInfoLabel.php?id=3484131&wwwpkgdb=fc2733d6468c073f1dd738aa598ede55&nocache=a9a895ad49f0f55c362d050dc1ad3fa3&dir=&wwwpkgdb=fc2733d6468c073f1dd738aa598ede55) | _ | nuclease-related domain family |
| [OCAR_0148](https://www.genoscope.cns.fr/agc/mage/wwwpkgdb/Info/getInfoLabel.php?id=3482101&wwwpkgdb=fc2733d6468c073f1dd738aa598ede55&nocache=a9a895ad49f0f55c362d050dc1ad3fa3&dir=&wwwpkgdb=fc2733d6468c073f1dd738aa598ede55) | _ | _ |
| [OCAR_4149](https://www.genoscope.cns.fr/agc/mage/wwwpkgdb/Info/getInfoLabel.php?id=3484132&wwwpkgdb=fc2733d6468c073f1dd738aa598ede55&nocache=a9a895ad49f0f55c362d050dc1ad3fa3&dir=&wwwpkgdb=fc2733d6468c073f1dd738aa598ede55) | _ | RNA polymerase sigma factor RpoD |
| [OCAR_0150](https://www.genoscope.cns.fr/agc/mage/wwwpkgdb/Info/getInfoLabel.php?id=3482100&wwwpkgdb=fc2733d6468c073f1dd738aa598ede55&nocache=a9a895ad49f0f55c362d050dc1ad3fa3&dir=&wwwpkgdb=fc2733d6468c073f1dd738aa598ede55) | _ | _ |
| [OCAR_4151](https://www.genoscope.cns.fr/agc/mage/wwwpkgdb/Info/getInfoLabel.php?id=3484133&wwwpkgdb=fc2733d6468c073f1dd738aa598ede55&nocache=a9a895ad49f0f55c362d050dc1ad3fa3&dir=&wwwpkgdb=fc2733d6468c073f1dd738aa598ede55) | _ | hypothetical protein |
| [OCAR_4152](https://www.genoscope.cns.fr/agc/mage/wwwpkgdb/Info/getInfoLabel.php?id=3484134&wwwpkgdb=fc2733d6468c073f1dd738aa598ede55&nocache=a9a895ad49f0f55c362d050dc1ad3fa3&dir=&wwwpkgdb=fc2733d6468c073f1dd738aa598ede55) | _ | hypothetical protein |
| [OCAR_4153](https://www.genoscope.cns.fr/agc/mage/wwwpkgdb/Info/getInfoLabel.php?id=3484135&wwwpkgdb=fc2733d6468c073f1dd738aa598ede55&nocache=a9a895ad49f0f55c362d050dc1ad3fa3&dir=&wwwpkgdb=fc2733d6468c073f1dd738aa598ede55) | _ | hypothetical protein |
| [OCAR_4154](https://www.genoscope.cns.fr/agc/mage/wwwpkgdb/Info/getInfoLabel.php?id=3484136&wwwpkgdb=fc2733d6468c073f1dd738aa598ede55&nocache=a9a895ad49f0f55c362d050dc1ad3fa3&dir=&wwwpkgdb=fc2733d6468c073f1dd738aa598ede55) | _ | hypothetical protein |
| [OCAR_4155](https://www.genoscope.cns.fr/agc/mage/wwwpkgdb/Info/getInfoLabel.php?id=3484137&wwwpkgdb=fc2733d6468c073f1dd738aa598ede55&nocache=a9a895ad49f0f55c362d050dc1ad3fa3&dir=&wwwpkgdb=fc2733d6468c073f1dd738aa598ede55) | _ | hypothetical protein |
| [OCAR_4156](https://www.genoscope.cns.fr/agc/mage/wwwpkgdb/Info/getInfoLabel.php?id=3484138&wwwpkgdb=fc2733d6468c073f1dd738aa598ede55&nocache=a9a895ad49f0f55c362d050dc1ad3fa3&dir=&wwwpkgdb=fc2733d6468c073f1dd738aa598ede55) | _ | UvrD/REP helicase |
| [OCAR_4158](https://www.genoscope.cns.fr/agc/mage/wwwpkgdb/Info/getInfoLabel.php?id=3484140&wwwpkgdb=fc2733d6468c073f1dd738aa598ede55&nocache=a9a895ad49f0f55c362d050dc1ad3fa3&dir=&wwwpkgdb=fc2733d6468c073f1dd738aa598ede55) | _ | BsuBIPstI restriction endonuclease domain protein |
| [OCAR_4159](https://www.genoscope.cns.fr/agc/mage/wwwpkgdb/Info/getInfoLabel.php?id=3484141&wwwpkgdb=fc2733d6468c073f1dd738aa598ede55&nocache=a9a895ad49f0f55c362d050dc1ad3fa3&dir=&wwwpkgdb=fc2733d6468c073f1dd738aa598ede55) | _ | restriction methylase |
| [OCAR_4162](https://www.genoscope.cns.fr/agc/mage/wwwpkgdb/Info/getInfoLabel.php?id=3484143&wwwpkgdb=fc2733d6468c073f1dd738aa598ede55&nocache=a9a895ad49f0f55c362d050dc1ad3fa3&dir=&wwwpkgdb=fc2733d6468c073f1dd738aa598ede55) | _ | hypothetical protein |
| [OCAR_4164](https://www.genoscope.cns.fr/agc/mage/wwwpkgdb/Info/getInfoLabel.php?id=3482203&wwwpkgdb=fc2733d6468c073f1dd738aa598ede55&nocache=a9a895ad49f0f55c362d050dc1ad3fa3&dir=&wwwpkgdb=fc2733d6468c073f1dd738aa598ede55) | _ | NADH-ubiquinone oxidoreductase 18 kd subunit |
| [OCAR_4165](https://www.genoscope.cns.fr/agc/mage/wwwpkgdb/Info/getInfoLabel.php?id=3484144&wwwpkgdb=fc2733d6468c073f1dd738aa598ede55&nocache=a9a895ad49f0f55c362d050dc1ad3fa3&dir=&wwwpkgdb=fc2733d6468c073f1dd738aa598ede55) | _ | hypothetical protein |
| [OCAR_0165](https://www.genoscope.cns.fr/agc/mage/wwwpkgdb/Info/getInfoLabel.php?id=3482099&wwwpkgdb=fc2733d6468c073f1dd738aa598ede55&nocache=a9a895ad49f0f55c362d050dc1ad3fa3&dir=&wwwpkgdb=fc2733d6468c073f1dd738aa598ede55) | _ | _ |
| [OCAR_4167](https://www.genoscope.cns.fr/agc/mage/wwwpkgdb/Info/getInfoLabel.php?id=3482205&wwwpkgdb=fc2733d6468c073f1dd738aa598ede55&nocache=a9a895ad49f0f55c362d050dc1ad3fa3&dir=&wwwpkgdb=fc2733d6468c073f1dd738aa598ede55) | _ | hypothetical protein |
| [OCAR_4168](https://www.genoscope.cns.fr/agc/mage/wwwpkgdb/Info/getInfoLabel.php?id=3484145&wwwpkgdb=fc2733d6468c073f1dd738aa598ede55&nocache=a9a895ad49f0f55c362d050dc1ad3fa3&dir=&wwwpkgdb=fc2733d6468c073f1dd738aa598ede55) | _ | CbzJ2 |
| [OCAR_4169](https://www.genoscope.cns.fr/agc/mage/wwwpkgdb/Info/getInfoLabel.php?id=3482206&wwwpkgdb=fc2733d6468c073f1dd738aa598ede55&nocache=a9a895ad49f0f55c362d050dc1ad3fa3&dir=&wwwpkgdb=fc2733d6468c073f1dd738aa598ede55) | _ | hypothetical protein |
| [OCAR_0169](https://www.genoscope.cns.fr/agc/mage/wwwpkgdb/Info/getInfoLabel.php?id=3482098&wwwpkgdb=fc2733d6468c073f1dd738aa598ede55&nocache=a9a895ad49f0f55c362d050dc1ad3fa3&dir=&wwwpkgdb=fc2733d6468c073f1dd738aa598ede55) | _ | _ |
| [OCAR_4170](https://www.genoscope.cns.fr/agc/mage/wwwpkgdb/Info/getInfoLabel.php?id=3482207&wwwpkgdb=fc2733d6468c073f1dd738aa598ede55&nocache=a9a895ad49f0f55c362d050dc1ad3fa3&dir=&wwwpkgdb=fc2733d6468c073f1dd738aa598ede55) | _ | hypothetical protein |
| [OCAR_4173](https://www.genoscope.cns.fr/agc/mage/wwwpkgdb/Info/getInfoLabel.php?id=3482210&wwwpkgdb=fc2733d6468c073f1dd738aa598ede55&nocache=a9a895ad49f0f55c362d050dc1ad3fa3&dir=&wwwpkgdb=fc2733d6468c073f1dd738aa598ede55) | _ | hypothetical protein |
| [OCAR_4193](https://www.genoscope.cns.fr/agc/mage/wwwpkgdb/Info/getInfoLabel.php?id=3484156&wwwpkgdb=fc2733d6468c073f1dd738aa598ede55&nocache=a9a895ad49f0f55c362d050dc1ad3fa3&dir=&wwwpkgdb=fc2733d6468c073f1dd738aa598ede55) | _ | hypothetical protein |
| [OCAR_4204](https://www.genoscope.cns.fr/agc/mage/wwwpkgdb/Info/getInfoLabel.php?id=3484167&wwwpkgdb=fc2733d6468c073f1dd738aa598ede55&nocache=a9a895ad49f0f55c362d050dc1ad3fa3&dir=&wwwpkgdb=fc2733d6468c073f1dd738aa598ede55) | _ | phage transcriptional regulator, AlpA |
| [OCAR_4205](https://www.genoscope.cns.fr/agc/mage/wwwpkgdb/Info/getInfoLabel.php?id=3484168&wwwpkgdb=fc2733d6468c073f1dd738aa598ede55&nocache=a9a895ad49f0f55c362d050dc1ad3fa3&dir=&wwwpkgdb=fc2733d6468c073f1dd738aa598ede55) | _ | hypothetical protein |
| [OCAR_4210](https://www.genoscope.cns.fr/agc/mage/wwwpkgdb/Info/getInfoLabel.php?id=3484173&wwwpkgdb=fc2733d6468c073f1dd738aa598ede55&nocache=a9a895ad49f0f55c362d050dc1ad3fa3&dir=&wwwpkgdb=fc2733d6468c073f1dd738aa598ede55) | _ | hypothetical protein |
| [OCAR_0212](https://www.genoscope.cns.fr/agc/mage/wwwpkgdb/Info/getInfoLabel.php?id=3482138&wwwpkgdb=fc2733d6468c073f1dd738aa598ede55&nocache=a9a895ad49f0f55c362d050dc1ad3fa3&dir=&wwwpkgdb=fc2733d6468c073f1dd738aa598ede55) | _ | _ |
| [OCAR_4211](https://www.genoscope.cns.fr/agc/mage/wwwpkgdb/Info/getInfoLabel.php?id=3482220&wwwpkgdb=fc2733d6468c073f1dd738aa598ede55&nocache=a9a895ad49f0f55c362d050dc1ad3fa3&dir=&wwwpkgdb=fc2733d6468c073f1dd738aa598ede55) | _ | hypothetical protein |
| [OCAR_4213](https://www.genoscope.cns.fr/agc/mage/wwwpkgdb/Info/getInfoLabel.php?id=3482221&wwwpkgdb=fc2733d6468c073f1dd738aa598ede55&nocache=a9a895ad49f0f55c362d050dc1ad3fa3&dir=&wwwpkgdb=fc2733d6468c073f1dd738aa598ede55) | _ | hypothetical protein |
| [OCAR_4216](https://www.genoscope.cns.fr/agc/mage/wwwpkgdb/Info/getInfoLabel.php?id=3484177&wwwpkgdb=fc2733d6468c073f1dd738aa598ede55&nocache=a9a895ad49f0f55c362d050dc1ad3fa3&dir=&wwwpkgdb=fc2733d6468c073f1dd738aa598ede55) | _ | hypothetical protein |
| [OCAR_4217](https://www.genoscope.cns.fr/agc/mage/wwwpkgdb/Info/getInfoLabel.php?id=3484178&wwwpkgdb=fc2733d6468c073f1dd738aa598ede55&nocache=a9a895ad49f0f55c362d050dc1ad3fa3&dir=&wwwpkgdb=fc2733d6468c073f1dd738aa598ede55) | _ | hypothetical protein |
| [OCAR_4218](https://www.genoscope.cns.fr/agc/mage/wwwpkgdb/Info/getInfoLabel.php?id=3484179&wwwpkgdb=fc2733d6468c073f1dd738aa598ede55&nocache=a9a895ad49f0f55c362d050dc1ad3fa3&dir=&wwwpkgdb=fc2733d6468c073f1dd738aa598ede55) | _ | hypothetical protein |
| [OCAR_4219](https://www.genoscope.cns.fr/agc/mage/wwwpkgdb/Info/getInfoLabel.php?id=3484180&wwwpkgdb=fc2733d6468c073f1dd738aa598ede55&nocache=a9a895ad49f0f55c362d050dc1ad3fa3&dir=&wwwpkgdb=fc2733d6468c073f1dd738aa598ede55) | _ | hypothetical protein |
| [OCAR_4221](https://www.genoscope.cns.fr/agc/mage/wwwpkgdb/Info/getInfoLabel.php?id=3484182&wwwpkgdb=fc2733d6468c073f1dd738aa598ede55&nocache=a9a895ad49f0f55c362d050dc1ad3fa3&dir=&wwwpkgdb=fc2733d6468c073f1dd738aa598ede55) | _ | hypothetical protein |
| [OCAR_4223](https://www.genoscope.cns.fr/agc/mage/wwwpkgdb/Info/getInfoLabel.php?id=3482222&wwwpkgdb=fc2733d6468c073f1dd738aa598ede55&nocache=a9a895ad49f0f55c362d050dc1ad3fa3&dir=&wwwpkgdb=fc2733d6468c073f1dd738aa598ede55) | _ | hypothetical protein |
| [OCAR_4224](https://www.genoscope.cns.fr/agc/mage/wwwpkgdb/Info/getInfoLabel.php?id=3482223&wwwpkgdb=fc2733d6468c073f1dd738aa598ede55&nocache=a9a895ad49f0f55c362d050dc1ad3fa3&dir=&wwwpkgdb=fc2733d6468c073f1dd738aa598ede55) | _ | hypothetical protein |
| [OCAR_4225](https://www.genoscope.cns.fr/agc/mage/wwwpkgdb/Info/getInfoLabel.php?id=3482224&wwwpkgdb=fc2733d6468c073f1dd738aa598ede55&nocache=a9a895ad49f0f55c362d050dc1ad3fa3&dir=&wwwpkgdb=fc2733d6468c073f1dd738aa598ede55) | _ | hypothetical protein |
| [OCAR_4226](https://www.genoscope.cns.fr/agc/mage/wwwpkgdb/Info/getInfoLabel.php?id=3482225&wwwpkgdb=fc2733d6468c073f1dd738aa598ede55&nocache=a9a895ad49f0f55c362d050dc1ad3fa3&dir=&wwwpkgdb=fc2733d6468c073f1dd738aa598ede55) | _ | dead/deah box helicase domain protein |
| [OCAR_4229](https://www.genoscope.cns.fr/agc/mage/wwwpkgdb/Info/getInfoLabel.php?id=3484185&wwwpkgdb=fc2733d6468c073f1dd738aa598ede55&nocache=a9a895ad49f0f55c362d050dc1ad3fa3&dir=&wwwpkgdb=fc2733d6468c073f1dd738aa598ede55) | arsH2 | arsenical resistance protein ArsH |
| [OCAR_4236](https://www.genoscope.cns.fr/agc/mage/wwwpkgdb/Info/getInfoLabel.php?id=3482227&wwwpkgdb=fc2733d6468c073f1dd738aa598ede55&nocache=a9a895ad49f0f55c362d050dc1ad3fa3&dir=&wwwpkgdb=fc2733d6468c073f1dd738aa598ede55) | _ | hypothetical protein |
| [OCAR_4235](https://www.genoscope.cns.fr/agc/mage/wwwpkgdb/Info/getInfoLabel.php?id=3484191&wwwpkgdb=fc2733d6468c073f1dd738aa598ede55&nocache=a9a895ad49f0f55c362d050dc1ad3fa3&dir=&wwwpkgdb=fc2733d6468c073f1dd738aa598ede55) | _ | hypothetical protein |
| [OCAR_4238](https://www.genoscope.cns.fr/agc/mage/wwwpkgdb/Info/getInfoLabel.php?id=3482229&wwwpkgdb=fc2733d6468c073f1dd738aa598ede55&nocache=a9a895ad49f0f55c362d050dc1ad3fa3&dir=&wwwpkgdb=fc2733d6468c073f1dd738aa598ede55) | _ | hypothetical protein |
| [OCAR_4239](https://www.genoscope.cns.fr/agc/mage/wwwpkgdb/Info/getInfoLabel.php?id=3484192&wwwpkgdb=fc2733d6468c073f1dd738aa598ede55&nocache=a9a895ad49f0f55c362d050dc1ad3fa3&dir=&wwwpkgdb=fc2733d6468c073f1dd738aa598ede55) | _ | hypothetical protein |
| [OCAR_4247](https://www.genoscope.cns.fr/agc/mage/wwwpkgdb/Info/getInfoLabel.php?id=3484200&wwwpkgdb=fc2733d6468c073f1dd738aa598ede55&nocache=a9a895ad49f0f55c362d050dc1ad3fa3&dir=&wwwpkgdb=fc2733d6468c073f1dd738aa598ede55) | _ | putative molybdate ABC transporter |
| [OCAR_4256](https://www.genoscope.cns.fr/agc/mage/wwwpkgdb/Info/getInfoLabel.php?id=3482233&wwwpkgdb=fc2733d6468c073f1dd738aa598ede55&nocache=a9a895ad49f0f55c362d050dc1ad3fa3&dir=&wwwpkgdb=fc2733d6468c073f1dd738aa598ede55) | _ | hypothetical protein |
| [OCAR_4258](https://www.genoscope.cns.fr/agc/mage/wwwpkgdb/Info/getInfoLabel.php?id=3482234&wwwpkgdb=fc2733d6468c073f1dd738aa598ede55&nocache=a9a895ad49f0f55c362d050dc1ad3fa3&dir=&wwwpkgdb=fc2733d6468c073f1dd738aa598ede55) | _ | transcriptional regulator, MarR family |
| [OCAR_4264](https://www.genoscope.cns.fr/agc/mage/wwwpkgdb/Info/getInfoLabel.php?id=3484211&wwwpkgdb=fc2733d6468c073f1dd738aa598ede55&nocache=a9a895ad49f0f55c362d050dc1ad3fa3&dir=&wwwpkgdb=fc2733d6468c073f1dd738aa598ede55) | _ | hypothetical protein |
| [OCAR_4272](https://www.genoscope.cns.fr/agc/mage/wwwpkgdb/Info/getInfoLabel.php?id=3484212&wwwpkgdb=fc2733d6468c073f1dd738aa598ede55&nocache=a9a895ad49f0f55c362d050dc1ad3fa3&dir=&wwwpkgdb=fc2733d6468c073f1dd738aa598ede55) | _ | glyoxalase family protein |
| [OCAR_4275](https://www.genoscope.cns.fr/agc/mage/wwwpkgdb/Info/getInfoLabel.php?id=3484214&wwwpkgdb=fc2733d6468c073f1dd738aa598ede55&nocache=a9a895ad49f0f55c362d050dc1ad3fa3&dir=&wwwpkgdb=fc2733d6468c073f1dd738aa598ede55) | _ | hypothetical protein |
| [OCAR_4280](https://www.genoscope.cns.fr/agc/mage/wwwpkgdb/Info/getInfoLabel.php?id=3484217&wwwpkgdb=fc2733d6468c073f1dd738aa598ede55&nocache=a9a895ad49f0f55c362d050dc1ad3fa3&dir=&wwwpkgdb=fc2733d6468c073f1dd738aa598ede55) | _ | hypothetical protein |
| [OCAR_4282](https://www.genoscope.cns.fr/agc/mage/wwwpkgdb/Info/getInfoLabel.php?id=3484218&wwwpkgdb=fc2733d6468c073f1dd738aa598ede55&nocache=a9a895ad49f0f55c362d050dc1ad3fa3&dir=&wwwpkgdb=fc2733d6468c073f1dd738aa598ede55) | _ | hypothetical protein |
| [OCAR_0285](https://www.genoscope.cns.fr/agc/mage/wwwpkgdb/Info/getInfoLabel.php?id=3482095&wwwpkgdb=fc2733d6468c073f1dd738aa598ede55&nocache=a9a895ad49f0f55c362d050dc1ad3fa3&dir=&wwwpkgdb=fc2733d6468c073f1dd738aa598ede55) | _ | _ |
| [OCAR_4292](https://www.genoscope.cns.fr/agc/mage/wwwpkgdb/Info/getInfoLabel.php?id=3484222&wwwpkgdb=fc2733d6468c073f1dd738aa598ede55&nocache=a9a895ad49f0f55c362d050dc1ad3fa3&dir=&wwwpkgdb=fc2733d6468c073f1dd738aa598ede55) | _ | hypothetical protein |
| [OCAR_4296](https://www.genoscope.cns.fr/agc/mage/wwwpkgdb/Info/getInfoLabel.php?id=3482254&wwwpkgdb=fc2733d6468c073f1dd738aa598ede55&nocache=a9a895ad49f0f55c362d050dc1ad3fa3&dir=&wwwpkgdb=fc2733d6468c073f1dd738aa598ede55) | _ | hypothetical protein |
| [OCAR_0296](https://www.genoscope.cns.fr/agc/mage/wwwpkgdb/Info/getInfoLabel.php?id=3482094&wwwpkgdb=fc2733d6468c073f1dd738aa598ede55&nocache=a9a895ad49f0f55c362d050dc1ad3fa3&dir=&wwwpkgdb=fc2733d6468c073f1dd738aa598ede55) | _ | _ |
| [OCAR_4300](https://www.genoscope.cns.fr/agc/mage/wwwpkgdb/Info/getInfoLabel.php?id=3482255&wwwpkgdb=fc2733d6468c073f1dd738aa598ede55&nocache=a9a895ad49f0f55c362d050dc1ad3fa3&dir=&wwwpkgdb=fc2733d6468c073f1dd738aa598ede55) | _ | hypothetical protein |
| [OCAR_4301](https://www.genoscope.cns.fr/agc/mage/wwwpkgdb/Info/getInfoLabel.php?id=3482256&wwwpkgdb=fc2733d6468c073f1dd738aa598ede55&nocache=a9a895ad49f0f55c362d050dc1ad3fa3&dir=&wwwpkgdb=fc2733d6468c073f1dd738aa598ede55) | _ | hypothetical protein |
| [OCAR_0300](https://www.genoscope.cns.fr/agc/mage/wwwpkgdb/Info/getInfoLabel.php?id=3482093&wwwpkgdb=fc2733d6468c073f1dd738aa598ede55&nocache=a9a895ad49f0f55c362d050dc1ad3fa3&dir=&wwwpkgdb=fc2733d6468c073f1dd738aa598ede55) | _ | _ |
| [OCAR_4319](https://www.genoscope.cns.fr/agc/mage/wwwpkgdb/Info/getInfoLabel.php?id=3484235&wwwpkgdb=fc2733d6468c073f1dd738aa598ede55&nocache=a9a895ad49f0f55c362d050dc1ad3fa3&dir=&wwwpkgdb=fc2733d6468c073f1dd738aa598ede55) | _ | hypothetical protein |
| [OCAR_4321](https://www.genoscope.cns.fr/agc/mage/wwwpkgdb/Info/getInfoLabel.php?id=3484237&wwwpkgdb=fc2733d6468c073f1dd738aa598ede55&nocache=a9a895ad49f0f55c362d050dc1ad3fa3&dir=&wwwpkgdb=fc2733d6468c073f1dd738aa598ede55) | _ | hypothetical protein |
| [OCAR_4322](https://www.genoscope.cns.fr/agc/mage/wwwpkgdb/Info/getInfoLabel.php?id=3482267&wwwpkgdb=fc2733d6468c073f1dd738aa598ede55&nocache=a9a895ad49f0f55c362d050dc1ad3fa3&dir=&wwwpkgdb=fc2733d6468c073f1dd738aa598ede55) | _ | diguanylate cyclase |
| [OCAR_4331](https://www.genoscope.cns.fr/agc/mage/wwwpkgdb/Info/getInfoLabel.php?id=3484242&wwwpkgdb=fc2733d6468c073f1dd738aa598ede55&nocache=a9a895ad49f0f55c362d050dc1ad3fa3&dir=&wwwpkgdb=fc2733d6468c073f1dd738aa598ede55) | _ | hypothetical protein |
| [OCAR_0329](https://www.genoscope.cns.fr/agc/mage/wwwpkgdb/Info/getInfoLabel.php?id=3482092&wwwpkgdb=fc2733d6468c073f1dd738aa598ede55&nocache=a9a895ad49f0f55c362d050dc1ad3fa3&dir=&wwwpkgdb=fc2733d6468c073f1dd738aa598ede55) | _ | _ |
| [OCAR_4345](https://www.genoscope.cns.fr/agc/mage/wwwpkgdb/Info/getInfoLabel.php?id=3484249&wwwpkgdb=fc2733d6468c073f1dd738aa598ede55&nocache=a9a895ad49f0f55c362d050dc1ad3fa3&dir=&wwwpkgdb=fc2733d6468c073f1dd738aa598ede55) | _ | hypothetical protein |
| [OCAR_4353](https://www.genoscope.cns.fr/agc/mage/wwwpkgdb/Info/getInfoLabel.php?id=3482285&wwwpkgdb=fc2733d6468c073f1dd738aa598ede55&nocache=a9a895ad49f0f55c362d050dc1ad3fa3&dir=&wwwpkgdb=fc2733d6468c073f1dd738aa598ede55) | _ | hypothetical protein |
| [OCAR_4371](https://www.genoscope.cns.fr/agc/mage/wwwpkgdb/Info/getInfoLabel.php?id=3482296&wwwpkgdb=fc2733d6468c073f1dd738aa598ede55&nocache=a9a895ad49f0f55c362d050dc1ad3fa3&dir=&wwwpkgdb=fc2733d6468c073f1dd738aa598ede55) | _ | hypothetical protein |
| [OCAR_4378](https://www.genoscope.cns.fr/agc/mage/wwwpkgdb/Info/getInfoLabel.php?id=3482298&wwwpkgdb=fc2733d6468c073f1dd738aa598ede55&nocache=a9a895ad49f0f55c362d050dc1ad3fa3&dir=&wwwpkgdb=fc2733d6468c073f1dd738aa598ede55) | _ | gentisate 1,2-dioxygenase |
| [OCAR_4383](https://www.genoscope.cns.fr/agc/mage/wwwpkgdb/Info/getInfoLabel.php?id=3482302&wwwpkgdb=fc2733d6468c073f1dd738aa598ede55&nocache=a9a895ad49f0f55c362d050dc1ad3fa3&dir=&wwwpkgdb=fc2733d6468c073f1dd738aa598ede55) | _ | hypothetical protein |
| [OCAR_4406](https://www.genoscope.cns.fr/agc/mage/wwwpkgdb/Info/getInfoLabel.php?id=3484272&wwwpkgdb=fc2733d6468c073f1dd738aa598ede55&nocache=a9a895ad49f0f55c362d050dc1ad3fa3&dir=&wwwpkgdb=fc2733d6468c073f1dd738aa598ede55) | _ | hypothetical protein |
| [OCAR_0400](https://www.genoscope.cns.fr/agc/mage/wwwpkgdb/Info/getInfoLabel.php?id=3482091&wwwpkgdb=fc2733d6468c073f1dd738aa598ede55&nocache=a9a895ad49f0f55c362d050dc1ad3fa3&dir=&wwwpkgdb=fc2733d6468c073f1dd738aa598ede55) | _ | _ |
| [OCAR_4434](https://www.genoscope.cns.fr/agc/mage/wwwpkgdb/Info/getInfoLabel.php?id=3484288&wwwpkgdb=fc2733d6468c073f1dd738aa598ede55&nocache=a9a895ad49f0f55c362d050dc1ad3fa3&dir=&wwwpkgdb=fc2733d6468c073f1dd738aa598ede55) | _ | extracellular solute-binding protein, family 5 |
| [OCAR_4435](https://www.genoscope.cns.fr/agc/mage/wwwpkgdb/Info/getInfoLabel.php?id=3482329&wwwpkgdb=fc2733d6468c073f1dd738aa598ede55&nocache=a9a895ad49f0f55c362d050dc1ad3fa3&dir=&wwwpkgdb=fc2733d6468c073f1dd738aa598ede55) | _ | putative transcriptional regulator of LysR family |
| [OCAR_0427](https://www.genoscope.cns.fr/agc/mage/wwwpkgdb/Info/getInfoLabel.php?id=3482090&wwwpkgdb=fc2733d6468c073f1dd738aa598ede55&nocache=a9a895ad49f0f55c362d050dc1ad3fa3&dir=&wwwpkgdb=fc2733d6468c073f1dd738aa598ede55) | _ | _ |
| [OCAR_4436](https://www.genoscope.cns.fr/agc/mage/wwwpkgdb/Info/getInfoLabel.php?id=3482330&wwwpkgdb=fc2733d6468c073f1dd738aa598ede55&nocache=a9a895ad49f0f55c362d050dc1ad3fa3&dir=&wwwpkgdb=fc2733d6468c073f1dd738aa598ede55) | _ | hypothetical protein |
| [OCAR_4437](https://www.genoscope.cns.fr/agc/mage/wwwpkgdb/Info/getInfoLabel.php?id=3482331&wwwpkgdb=fc2733d6468c073f1dd738aa598ede55&nocache=a9a895ad49f0f55c362d050dc1ad3fa3&dir=&wwwpkgdb=fc2733d6468c073f1dd738aa598ede55) | _ | putative poly(aspartic acid) Hydrolase |
| [OCAR_0434](https://www.genoscope.cns.fr/agc/mage/wwwpkgdb/Info/getInfoLabel.php?id=3482089&wwwpkgdb=fc2733d6468c073f1dd738aa598ede55&nocache=a9a895ad49f0f55c362d050dc1ad3fa3&dir=&wwwpkgdb=fc2733d6468c073f1dd738aa598ede55) | _ | _ |
| [OCAR_4444](https://www.genoscope.cns.fr/agc/mage/wwwpkgdb/Info/getInfoLabel.php?id=3482332&wwwpkgdb=fc2733d6468c073f1dd738aa598ede55&nocache=a9a895ad49f0f55c362d050dc1ad3fa3&dir=&wwwpkgdb=fc2733d6468c073f1dd738aa598ede55) | _ | hypothetical protein |
| [OCAR_4443](https://www.genoscope.cns.fr/agc/mage/wwwpkgdb/Info/getInfoLabel.php?id=3484294&wwwpkgdb=fc2733d6468c073f1dd738aa598ede55&nocache=a9a895ad49f0f55c362d050dc1ad3fa3&dir=&wwwpkgdb=fc2733d6468c073f1dd738aa598ede55) | _ | glycosyltransferase |
| [OCAR_4449](https://www.genoscope.cns.fr/agc/mage/wwwpkgdb/Info/getInfoLabel.php?id=3484297&wwwpkgdb=fc2733d6468c073f1dd738aa598ede55&nocache=a9a895ad49f0f55c362d050dc1ad3fa3&dir=&wwwpkgdb=fc2733d6468c073f1dd738aa598ede55) | _ | hypothetical protein |
| [OCAR_4454](https://www.genoscope.cns.fr/agc/mage/wwwpkgdb/Info/getInfoLabel.php?id=3482338&wwwpkgdb=fc2733d6468c073f1dd738aa598ede55&nocache=a9a895ad49f0f55c362d050dc1ad3fa3&dir=&wwwpkgdb=fc2733d6468c073f1dd738aa598ede55) | _ | hypothetical protein |
| [OCAR_4468](https://www.genoscope.cns.fr/agc/mage/wwwpkgdb/Info/getInfoLabel.php?id=3482347&wwwpkgdb=fc2733d6468c073f1dd738aa598ede55&nocache=a9a895ad49f0f55c362d050dc1ad3fa3&dir=&wwwpkgdb=fc2733d6468c073f1dd738aa598ede55) | _ | hypothetical protein |
| [OCAR_4469](https://www.genoscope.cns.fr/agc/mage/wwwpkgdb/Info/getInfoLabel.php?id=3482348&wwwpkgdb=fc2733d6468c073f1dd738aa598ede55&nocache=a9a895ad49f0f55c362d050dc1ad3fa3&dir=&wwwpkgdb=fc2733d6468c073f1dd738aa598ede55) | _ | hypothetical protein |
| [OCAR_0460](https://www.genoscope.cns.fr/agc/mage/wwwpkgdb/Info/getInfoLabel.php?id=3482088&wwwpkgdb=fc2733d6468c073f1dd738aa598ede55&nocache=a9a895ad49f0f55c362d050dc1ad3fa3&dir=&wwwpkgdb=fc2733d6468c073f1dd738aa598ede55) | _ | _ |
| [OCAR_4471](https://www.genoscope.cns.fr/agc/mage/wwwpkgdb/Info/getInfoLabel.php?id=3484304&wwwpkgdb=fc2733d6468c073f1dd738aa598ede55&nocache=a9a895ad49f0f55c362d050dc1ad3fa3&dir=&wwwpkgdb=fc2733d6468c073f1dd738aa598ede55) | _ | hypothetical protein |
| [OCAR_0491](https://www.genoscope.cns.fr/agc/mage/wwwpkgdb/Info/getInfoLabel.php?id=3482087&wwwpkgdb=fc2733d6468c073f1dd738aa598ede55&nocache=a9a895ad49f0f55c362d050dc1ad3fa3&dir=&wwwpkgdb=fc2733d6468c073f1dd738aa598ede55) | _ | _ |
| [OCAR_4507](https://www.genoscope.cns.fr/agc/mage/wwwpkgdb/Info/getInfoLabel.php?id=3484324&wwwpkgdb=fc2733d6468c073f1dd738aa598ede55&nocache=a9a895ad49f0f55c362d050dc1ad3fa3&dir=&wwwpkgdb=fc2733d6468c073f1dd738aa598ede55) | _ | hypothetical protein |
| [OCAR_4509](https://www.genoscope.cns.fr/agc/mage/wwwpkgdb/Info/getInfoLabel.php?id=3482366&wwwpkgdb=fc2733d6468c073f1dd738aa598ede55&nocache=a9a895ad49f0f55c362d050dc1ad3fa3&dir=&wwwpkgdb=fc2733d6468c073f1dd738aa598ede55) | _ | hypothetical protein |
| [OCAR_4516](https://www.genoscope.cns.fr/agc/mage/wwwpkgdb/Info/getInfoLabel.php?id=3482368&wwwpkgdb=fc2733d6468c073f1dd738aa598ede55&nocache=a9a895ad49f0f55c362d050dc1ad3fa3&dir=&wwwpkgdb=fc2733d6468c073f1dd738aa598ede55) | _ | hypothetical protein |
| [OCAR_4533](https://www.genoscope.cns.fr/agc/mage/wwwpkgdb/Info/getInfoLabel.php?id=3482371&wwwpkgdb=fc2733d6468c073f1dd738aa598ede55&nocache=a9a895ad49f0f55c362d050dc1ad3fa3&dir=&wwwpkgdb=fc2733d6468c073f1dd738aa598ede55) | _ | hypothetical protein |
| [OCAR_4537](https://www.genoscope.cns.fr/agc/mage/wwwpkgdb/Info/getInfoLabel.php?id=3482372&wwwpkgdb=fc2733d6468c073f1dd738aa598ede55&nocache=a9a895ad49f0f55c362d050dc1ad3fa3&dir=&wwwpkgdb=fc2733d6468c073f1dd738aa598ede55) | _ | hypothetical protein |
| [OCAR_4539](https://www.genoscope.cns.fr/agc/mage/wwwpkgdb/Info/getInfoLabel.php?id=3484349&wwwpkgdb=fc2733d6468c073f1dd738aa598ede55&nocache=a9a895ad49f0f55c362d050dc1ad3fa3&dir=&wwwpkgdb=fc2733d6468c073f1dd738aa598ede55) | _ | hypothetical protein |
| [OCAR_4546](https://www.genoscope.cns.fr/agc/mage/wwwpkgdb/Info/getInfoLabel.php?id=3484353&wwwpkgdb=fc2733d6468c073f1dd738aa598ede55&nocache=a9a895ad49f0f55c362d050dc1ad3fa3&dir=&wwwpkgdb=fc2733d6468c073f1dd738aa598ede55) | _ | hypothetical protein |
| [OCAR_0541](https://www.genoscope.cns.fr/agc/mage/wwwpkgdb/Info/getInfoLabel.php?id=3482085&wwwpkgdb=fc2733d6468c073f1dd738aa598ede55&nocache=a9a895ad49f0f55c362d050dc1ad3fa3&dir=&wwwpkgdb=fc2733d6468c073f1dd738aa598ede55) | _ | _ |
| [OCAR_4554](https://www.genoscope.cns.fr/agc/mage/wwwpkgdb/Info/getInfoLabel.php?id=3484358&wwwpkgdb=fc2733d6468c073f1dd738aa598ede55&nocache=a9a895ad49f0f55c362d050dc1ad3fa3&dir=&wwwpkgdb=fc2733d6468c073f1dd738aa598ede55) | _ | hypothetical protein |
| [OCAR_4556](https://www.genoscope.cns.fr/agc/mage/wwwpkgdb/Info/getInfoLabel.php?id=3484359&wwwpkgdb=fc2733d6468c073f1dd738aa598ede55&nocache=a9a895ad49f0f55c362d050dc1ad3fa3&dir=&wwwpkgdb=fc2733d6468c073f1dd738aa598ede55) | _ | hypothetical protein |
| [OCAR_4558](https://www.genoscope.cns.fr/agc/mage/wwwpkgdb/Info/getInfoLabel.php?id=3484360&wwwpkgdb=fc2733d6468c073f1dd738aa598ede55&nocache=a9a895ad49f0f55c362d050dc1ad3fa3&dir=&wwwpkgdb=fc2733d6468c073f1dd738aa598ede55) | _ | glycosyl transferase, family 2 |
| [OCAR_4559](https://www.genoscope.cns.fr/agc/mage/wwwpkgdb/Info/getInfoLabel.php?id=3482381&wwwpkgdb=fc2733d6468c073f1dd738aa598ede55&nocache=a9a895ad49f0f55c362d050dc1ad3fa3&dir=&wwwpkgdb=fc2733d6468c073f1dd738aa598ede55) | _ | hypothetical protein |
| [OCAR_4563](https://www.genoscope.cns.fr/agc/mage/wwwpkgdb/Info/getInfoLabel.php?id=3482385&wwwpkgdb=fc2733d6468c073f1dd738aa598ede55&nocache=a9a895ad49f0f55c362d050dc1ad3fa3&dir=&wwwpkgdb=fc2733d6468c073f1dd738aa598ede55) | _ | methyltransferase type 12 |
| [OCAR_4564](https://www.genoscope.cns.fr/agc/mage/wwwpkgdb/Info/getInfoLabel.php?id=3482386&wwwpkgdb=fc2733d6468c073f1dd738aa598ede55&nocache=a9a895ad49f0f55c362d050dc1ad3fa3&dir=&wwwpkgdb=fc2733d6468c073f1dd738aa598ede55) | _ | periplasmic binding protein |
| [OCAR_0576](https://www.genoscope.cns.fr/agc/mage/wwwpkgdb/Info/getInfoLabel.php?id=3482084&wwwpkgdb=fc2733d6468c073f1dd738aa598ede55&nocache=a9a895ad49f0f55c362d050dc1ad3fa3&dir=&wwwpkgdb=fc2733d6468c073f1dd738aa598ede55) | _ | _ |
| [OCAR_4590](https://www.genoscope.cns.fr/agc/mage/wwwpkgdb/Info/getInfoLabel.php?id=3484374&wwwpkgdb=fc2733d6468c073f1dd738aa598ede55&nocache=a9a895ad49f0f55c362d050dc1ad3fa3&dir=&wwwpkgdb=fc2733d6468c073f1dd738aa598ede55) | _ | hypothetical protein |
| [OCAR_4616](https://www.genoscope.cns.fr/agc/mage/wwwpkgdb/Info/getInfoLabel.php?id=3484389&wwwpkgdb=fc2733d6468c073f1dd738aa598ede55&nocache=a9a895ad49f0f55c362d050dc1ad3fa3&dir=&wwwpkgdb=fc2733d6468c073f1dd738aa598ede55) | _ | hypothetical protein |
| [OCAR_4617](https://www.genoscope.cns.fr/agc/mage/wwwpkgdb/Info/getInfoLabel.php?id=3484390&wwwpkgdb=fc2733d6468c073f1dd738aa598ede55&nocache=a9a895ad49f0f55c362d050dc1ad3fa3&dir=&wwwpkgdb=fc2733d6468c073f1dd738aa598ede55) | _ | hypothetical protein |
| [OCAR_4618](https://www.genoscope.cns.fr/agc/mage/wwwpkgdb/Info/getInfoLabel.php?id=3482410&wwwpkgdb=fc2733d6468c073f1dd738aa598ede55&nocache=a9a895ad49f0f55c362d050dc1ad3fa3&dir=&wwwpkgdb=fc2733d6468c073f1dd738aa598ede55) | _ | hypothetical protein |
| [OCAR_4622](https://www.genoscope.cns.fr/agc/mage/wwwpkgdb/Info/getInfoLabel.php?id=3482414&wwwpkgdb=fc2733d6468c073f1dd738aa598ede55&nocache=a9a895ad49f0f55c362d050dc1ad3fa3&dir=&wwwpkgdb=fc2733d6468c073f1dd738aa598ede55) | _ | NAD dependent epimerase/dehydratase family |
| [OCAR_0608](https://www.genoscope.cns.fr/agc/mage/wwwpkgdb/Info/getInfoLabel.php?id=3482083&wwwpkgdb=fc2733d6468c073f1dd738aa598ede55&nocache=a9a895ad49f0f55c362d050dc1ad3fa3&dir=&wwwpkgdb=fc2733d6468c073f1dd738aa598ede55) | _ | _ |
| [OCAR_4623](https://www.genoscope.cns.fr/agc/mage/wwwpkgdb/Info/getInfoLabel.php?id=3482415&wwwpkgdb=fc2733d6468c073f1dd738aa598ede55&nocache=a9a895ad49f0f55c362d050dc1ad3fa3&dir=&wwwpkgdb=fc2733d6468c073f1dd738aa598ede55) | _ | hypothetical protein |
| [OCAR_4624](https://www.genoscope.cns.fr/agc/mage/wwwpkgdb/Info/getInfoLabel.php?id=3482416&wwwpkgdb=fc2733d6468c073f1dd738aa598ede55&nocache=a9a895ad49f0f55c362d050dc1ad3fa3&dir=&wwwpkgdb=fc2733d6468c073f1dd738aa598ede55) | _ | signal transduction histidine kinase containing PAS/PAC sensor domain |
| [OCAR_4629](https://www.genoscope.cns.fr/agc/mage/wwwpkgdb/Info/getInfoLabel.php?id=3484391&wwwpkgdb=fc2733d6468c073f1dd738aa598ede55&nocache=a9a895ad49f0f55c362d050dc1ad3fa3&dir=&wwwpkgdb=fc2733d6468c073f1dd738aa598ede55) | _ | mekhla domain superfamily |
| [OCAR_0623](https://www.genoscope.cns.fr/agc/mage/wwwpkgdb/Info/getInfoLabel.php?id=3482082&wwwpkgdb=fc2733d6468c073f1dd738aa598ede55&nocache=a9a895ad49f0f55c362d050dc1ad3fa3&dir=&wwwpkgdb=fc2733d6468c073f1dd738aa598ede55) | _ | _ |
| [OCAR_4637](https://www.genoscope.cns.fr/agc/mage/wwwpkgdb/Info/getInfoLabel.php?id=3484395&wwwpkgdb=fc2733d6468c073f1dd738aa598ede55&nocache=a9a895ad49f0f55c362d050dc1ad3fa3&dir=&wwwpkgdb=fc2733d6468c073f1dd738aa598ede55) | _ | hypothetical protein |
| [OCAR_4640](https://www.genoscope.cns.fr/agc/mage/wwwpkgdb/Info/getInfoLabel.php?id=3482425&wwwpkgdb=fc2733d6468c073f1dd738aa598ede55&nocache=a9a895ad49f0f55c362d050dc1ad3fa3&dir=&wwwpkgdb=fc2733d6468c073f1dd738aa598ede55) | _ | hypothetical protein |
| [OCAR_4653](https://www.genoscope.cns.fr/agc/mage/wwwpkgdb/Info/getInfoLabel.php?id=3484400&wwwpkgdb=fc2733d6468c073f1dd738aa598ede55&nocache=a9a895ad49f0f55c362d050dc1ad3fa3&dir=&wwwpkgdb=fc2733d6468c073f1dd738aa598ede55) | _ | hypothetical protein |
| [OCAR_0647](https://www.genoscope.cns.fr/agc/mage/wwwpkgdb/Info/getInfoLabel.php?id=3482081&wwwpkgdb=fc2733d6468c073f1dd738aa598ede55&nocache=a9a895ad49f0f55c362d050dc1ad3fa3&dir=&wwwpkgdb=fc2733d6468c073f1dd738aa598ede55) | _ | _ |
| [OCAR_4670](https://www.genoscope.cns.fr/agc/mage/wwwpkgdb/Info/getInfoLabel.php?id=3484404&wwwpkgdb=fc2733d6468c073f1dd738aa598ede55&nocache=a9a895ad49f0f55c362d050dc1ad3fa3&dir=&wwwpkgdb=fc2733d6468c073f1dd738aa598ede55) | _ | conserved hypothetical secreted protein |
| [OCAR_0657](https://www.genoscope.cns.fr/agc/mage/wwwpkgdb/Info/getInfoLabel.php?id=3482080&wwwpkgdb=fc2733d6468c073f1dd738aa598ede55&nocache=a9a895ad49f0f55c362d050dc1ad3fa3&dir=&wwwpkgdb=fc2733d6468c073f1dd738aa598ede55) | _ | _ |
| [OCAR_4685](https://www.genoscope.cns.fr/agc/mage/wwwpkgdb/Info/getInfoLabel.php?id=3484412&wwwpkgdb=fc2733d6468c073f1dd738aa598ede55&nocache=a9a895ad49f0f55c362d050dc1ad3fa3&dir=&wwwpkgdb=fc2733d6468c073f1dd738aa598ede55) | _ | putative lipoprotein |
| [OCAR_4691](https://www.genoscope.cns.fr/agc/mage/wwwpkgdb/Info/getInfoLabel.php?id=3482461&wwwpkgdb=fc2733d6468c073f1dd738aa598ede55&nocache=a9a895ad49f0f55c362d050dc1ad3fa3&dir=&wwwpkgdb=fc2733d6468c073f1dd738aa598ede55) | _ | hypothetical protein |
| [OCAR_0680](https://www.genoscope.cns.fr/agc/mage/wwwpkgdb/Info/getInfoLabel.php?id=3482079&wwwpkgdb=fc2733d6468c073f1dd738aa598ede55&nocache=a9a895ad49f0f55c362d050dc1ad3fa3&dir=&wwwpkgdb=fc2733d6468c073f1dd738aa598ede55) | _ | _ |
| [OCAR_4709](https://www.genoscope.cns.fr/agc/mage/wwwpkgdb/Info/getInfoLabel.php?id=3482471&wwwpkgdb=fc2733d6468c073f1dd738aa598ede55&nocache=a9a895ad49f0f55c362d050dc1ad3fa3&dir=&wwwpkgdb=fc2733d6468c073f1dd738aa598ede55) | _ | class II aldolase/adducin family protein |
| [OCAR_4719](https://www.genoscope.cns.fr/agc/mage/wwwpkgdb/Info/getInfoLabel.php?id=3482477&wwwpkgdb=fc2733d6468c073f1dd738aa598ede55&nocache=a9a895ad49f0f55c362d050dc1ad3fa3&dir=&wwwpkgdb=fc2733d6468c073f1dd738aa598ede55) | _ | transcriptional regulator, TetR family |
| [OCAR_4722](https://www.genoscope.cns.fr/agc/mage/wwwpkgdb/Info/getInfoLabel.php?id=3482480&wwwpkgdb=fc2733d6468c073f1dd738aa598ede55&nocache=a9a895ad49f0f55c362d050dc1ad3fa3&dir=&wwwpkgdb=fc2733d6468c073f1dd738aa598ede55) | _ | hypothetical protein |
| [OCAR_4725](https://www.genoscope.cns.fr/agc/mage/wwwpkgdb/Info/getInfoLabel.php?id=3482483&wwwpkgdb=fc2733d6468c073f1dd738aa598ede55&nocache=a9a895ad49f0f55c362d050dc1ad3fa3&dir=&wwwpkgdb=fc2733d6468c073f1dd738aa598ede55) | _ | hypothetical protein |
| [OCAR_4738](https://www.genoscope.cns.fr/agc/mage/wwwpkgdb/Info/getInfoLabel.php?id=3482493&wwwpkgdb=fc2733d6468c073f1dd738aa598ede55&nocache=a9a895ad49f0f55c362d050dc1ad3fa3&dir=&wwwpkgdb=fc2733d6468c073f1dd738aa598ede55) | _ | methylase involved in ubiquinone/menaquinone biosynthesis |
| [OCAR_4739](https://www.genoscope.cns.fr/agc/mage/wwwpkgdb/Info/getInfoLabel.php?id=3482494&wwwpkgdb=fc2733d6468c073f1dd738aa598ede55&nocache=a9a895ad49f0f55c362d050dc1ad3fa3&dir=&wwwpkgdb=fc2733d6468c073f1dd738aa598ede55) | _ | membrane protein involved in aromatic hydrocarbon degradation |
| [OCAR_4744](https://www.genoscope.cns.fr/agc/mage/wwwpkgdb/Info/getInfoLabel.php?id=3482495&wwwpkgdb=fc2733d6468c073f1dd738aa598ede55&nocache=a9a895ad49f0f55c362d050dc1ad3fa3&dir=&wwwpkgdb=fc2733d6468c073f1dd738aa598ede55) | _ | hypothetical protein |
| [OCAR_4743](https://www.genoscope.cns.fr/agc/mage/wwwpkgdb/Info/getInfoLabel.php?id=3484431&wwwpkgdb=fc2733d6468c073f1dd738aa598ede55&nocache=a9a895ad49f0f55c362d050dc1ad3fa3&dir=&wwwpkgdb=fc2733d6468c073f1dd738aa598ede55) | _ | hypothetical protein |
| [OCAR_4750](https://www.genoscope.cns.fr/agc/mage/wwwpkgdb/Info/getInfoLabel.php?id=3482499&wwwpkgdb=fc2733d6468c073f1dd738aa598ede55&nocache=a9a895ad49f0f55c362d050dc1ad3fa3&dir=&wwwpkgdb=fc2733d6468c073f1dd738aa598ede55) | _ | hypothetical protein |
| [OCAR_4775](https://www.genoscope.cns.fr/agc/mage/wwwpkgdb/Info/getInfoLabel.php?id=3484443&wwwpkgdb=fc2733d6468c073f1dd738aa598ede55&nocache=a9a895ad49f0f55c362d050dc1ad3fa3&dir=&wwwpkgdb=fc2733d6468c073f1dd738aa598ede55) | _ | hypothetical protein |
| [OCAR_0760](https://www.genoscope.cns.fr/agc/mage/wwwpkgdb/Info/getInfoLabel.php?id=3482076&wwwpkgdb=fc2733d6468c073f1dd738aa598ede55&nocache=a9a895ad49f0f55c362d050dc1ad3fa3&dir=&wwwpkgdb=fc2733d6468c073f1dd738aa598ede55) | _ | _ |
| [OCAR_4784](https://www.genoscope.cns.fr/agc/mage/wwwpkgdb/Info/getInfoLabel.php?id=3484446&wwwpkgdb=fc2733d6468c073f1dd738aa598ede55&nocache=a9a895ad49f0f55c362d050dc1ad3fa3&dir=&wwwpkgdb=fc2733d6468c073f1dd738aa598ede55) | _ | hypothetical protein |
| [OCAR_0766](https://www.genoscope.cns.fr/agc/mage/wwwpkgdb/Info/getInfoLabel.php?id=3482075&wwwpkgdb=fc2733d6468c073f1dd738aa598ede55&nocache=a9a895ad49f0f55c362d050dc1ad3fa3&dir=&wwwpkgdb=fc2733d6468c073f1dd738aa598ede55) | _ | _ |
| [OCAR_0769](https://www.genoscope.cns.fr/agc/mage/wwwpkgdb/Info/getInfoLabel.php?id=3482074&wwwpkgdb=fc2733d6468c073f1dd738aa598ede55&nocache=a9a895ad49f0f55c362d050dc1ad3fa3&dir=&wwwpkgdb=fc2733d6468c073f1dd738aa598ede55) | _ | _ |
| [OCAR_4788](https://www.genoscope.cns.fr/agc/mage/wwwpkgdb/Info/getInfoLabel.php?id=3484447&wwwpkgdb=fc2733d6468c073f1dd738aa598ede55&nocache=a9a895ad49f0f55c362d050dc1ad3fa3&dir=&wwwpkgdb=fc2733d6468c073f1dd738aa598ede55) | _ | regulatory protein of LacI family |
| [OCAR_0773](https://www.genoscope.cns.fr/agc/mage/wwwpkgdb/Info/getInfoLabel.php?id=3482073&wwwpkgdb=fc2733d6468c073f1dd738aa598ede55&nocache=a9a895ad49f0f55c362d050dc1ad3fa3&dir=&wwwpkgdb=fc2733d6468c073f1dd738aa598ede55) | _ | _ |
| [OCAR_4791](https://www.genoscope.cns.fr/agc/mage/wwwpkgdb/Info/getInfoLabel.php?id=3484450&wwwpkgdb=fc2733d6468c073f1dd738aa598ede55&nocache=a9a895ad49f0f55c362d050dc1ad3fa3&dir=&wwwpkgdb=fc2733d6468c073f1dd738aa598ede55) | _ | hypothetical protein |
| [OCAR_4792](https://www.genoscope.cns.fr/agc/mage/wwwpkgdb/Info/getInfoLabel.php?id=3484451&wwwpkgdb=fc2733d6468c073f1dd738aa598ede55&nocache=a9a895ad49f0f55c362d050dc1ad3fa3&dir=&wwwpkgdb=fc2733d6468c073f1dd738aa598ede55) | _ | hypothetical protein |
| [OCAR_4793](https://www.genoscope.cns.fr/agc/mage/wwwpkgdb/Info/getInfoLabel.php?id=3484452&wwwpkgdb=fc2733d6468c073f1dd738aa598ede55&nocache=a9a895ad49f0f55c362d050dc1ad3fa3&dir=&wwwpkgdb=fc2733d6468c073f1dd738aa598ede55) | _ | hypothetical protein |
| [OCAR_4794](https://www.genoscope.cns.fr/agc/mage/wwwpkgdb/Info/getInfoLabel.php?id=3484453&wwwpkgdb=fc2733d6468c073f1dd738aa598ede55&nocache=a9a895ad49f0f55c362d050dc1ad3fa3&dir=&wwwpkgdb=fc2733d6468c073f1dd738aa598ede55) | _ | hypothetical protein |
| [OCAR_4795](https://www.genoscope.cns.fr/agc/mage/wwwpkgdb/Info/getInfoLabel.php?id=3484454&wwwpkgdb=fc2733d6468c073f1dd738aa598ede55&nocache=a9a895ad49f0f55c362d050dc1ad3fa3&dir=&wwwpkgdb=fc2733d6468c073f1dd738aa598ede55) | _ | putative lipoprotein |
| [OCAR_4798](https://www.genoscope.cns.fr/agc/mage/wwwpkgdb/Info/getInfoLabel.php?id=3482529&wwwpkgdb=fc2733d6468c073f1dd738aa598ede55&nocache=a9a895ad49f0f55c362d050dc1ad3fa3&dir=&wwwpkgdb=fc2733d6468c073f1dd738aa598ede55) | _ | transcriptional regulator of XRE family |
| [OCAR_4799](https://www.genoscope.cns.fr/agc/mage/wwwpkgdb/Info/getInfoLabel.php?id=3482530&wwwpkgdb=fc2733d6468c073f1dd738aa598ede55&nocache=a9a895ad49f0f55c362d050dc1ad3fa3&dir=&wwwpkgdb=fc2733d6468c073f1dd738aa598ede55) | _ | hypothetical protein |
| [OCAR_4800](https://www.genoscope.cns.fr/agc/mage/wwwpkgdb/Info/getInfoLabel.php?id=3482531&wwwpkgdb=fc2733d6468c073f1dd738aa598ede55&nocache=a9a895ad49f0f55c362d050dc1ad3fa3&dir=&wwwpkgdb=fc2733d6468c073f1dd738aa598ede55) | _ | hypothetical protein |
| [OCAR_4801](https://www.genoscope.cns.fr/agc/mage/wwwpkgdb/Info/getInfoLabel.php?id=3482532&wwwpkgdb=fc2733d6468c073f1dd738aa598ede55&nocache=a9a895ad49f0f55c362d050dc1ad3fa3&dir=&wwwpkgdb=fc2733d6468c073f1dd738aa598ede55) | _ | hypothetical protein |
| [OCAR_4802](https://www.genoscope.cns.fr/agc/mage/wwwpkgdb/Info/getInfoLabel.php?id=3482533&wwwpkgdb=fc2733d6468c073f1dd738aa598ede55&nocache=a9a895ad49f0f55c362d050dc1ad3fa3&dir=&wwwpkgdb=fc2733d6468c073f1dd738aa598ede55) | _ | hypothetical protein |
| [OCAR_4803](https://www.genoscope.cns.fr/agc/mage/wwwpkgdb/Info/getInfoLabel.php?id=3482534&wwwpkgdb=fc2733d6468c073f1dd738aa598ede55&nocache=a9a895ad49f0f55c362d050dc1ad3fa3&dir=&wwwpkgdb=fc2733d6468c073f1dd738aa598ede55) | _ | hypothetical protein |
| [OCAR_4804](https://www.genoscope.cns.fr/agc/mage/wwwpkgdb/Info/getInfoLabel.php?id=3482535&wwwpkgdb=fc2733d6468c073f1dd738aa598ede55&nocache=a9a895ad49f0f55c362d050dc1ad3fa3&dir=&wwwpkgdb=fc2733d6468c073f1dd738aa598ede55) | _ | hypothetical protein |
| [OCAR_4805](https://www.genoscope.cns.fr/agc/mage/wwwpkgdb/Info/getInfoLabel.php?id=3482536&wwwpkgdb=fc2733d6468c073f1dd738aa598ede55&nocache=a9a895ad49f0f55c362d050dc1ad3fa3&dir=&wwwpkgdb=fc2733d6468c073f1dd738aa598ede55) | _ | hypothetical protein |
| [OCAR_4806](https://www.genoscope.cns.fr/agc/mage/wwwpkgdb/Info/getInfoLabel.php?id=3482537&wwwpkgdb=fc2733d6468c073f1dd738aa598ede55&nocache=a9a895ad49f0f55c362d050dc1ad3fa3&dir=&wwwpkgdb=fc2733d6468c073f1dd738aa598ede55) | _ | hypothetical protein |
| [OCAR_4809](https://www.genoscope.cns.fr/agc/mage/wwwpkgdb/Info/getInfoLabel.php?id=3482540&wwwpkgdb=fc2733d6468c073f1dd738aa598ede55&nocache=a9a895ad49f0f55c362d050dc1ad3fa3&dir=&wwwpkgdb=fc2733d6468c073f1dd738aa598ede55) | _ | hypothetical protein |
| [OCAR_4810](https://www.genoscope.cns.fr/agc/mage/wwwpkgdb/Info/getInfoLabel.php?id=3482541&wwwpkgdb=fc2733d6468c073f1dd738aa598ede55&nocache=a9a895ad49f0f55c362d050dc1ad3fa3&dir=&wwwpkgdb=fc2733d6468c073f1dd738aa598ede55) | _ | hypothetical protein |
| [OCAR_4811](https://www.genoscope.cns.fr/agc/mage/wwwpkgdb/Info/getInfoLabel.php?id=3482542&wwwpkgdb=fc2733d6468c073f1dd738aa598ede55&nocache=a9a895ad49f0f55c362d050dc1ad3fa3&dir=&wwwpkgdb=fc2733d6468c073f1dd738aa598ede55) | _ | holliday junction resolvasome, endonuclease subunit |
| [OCAR_4813](https://www.genoscope.cns.fr/agc/mage/wwwpkgdb/Info/getInfoLabel.php?id=3482544&wwwpkgdb=fc2733d6468c073f1dd738aa598ede55&nocache=a9a895ad49f0f55c362d050dc1ad3fa3&dir=&wwwpkgdb=fc2733d6468c073f1dd738aa598ede55) | _ | hypothetical protein |
| [OCAR_4814](https://www.genoscope.cns.fr/agc/mage/wwwpkgdb/Info/getInfoLabel.php?id=3482545&wwwpkgdb=fc2733d6468c073f1dd738aa598ede55&nocache=a9a895ad49f0f55c362d050dc1ad3fa3&dir=&wwwpkgdb=fc2733d6468c073f1dd738aa598ede55) | _ | hypothetical protein |
| [OCAR_4815](https://www.genoscope.cns.fr/agc/mage/wwwpkgdb/Info/getInfoLabel.php?id=3482546&wwwpkgdb=fc2733d6468c073f1dd738aa598ede55&nocache=a9a895ad49f0f55c362d050dc1ad3fa3&dir=&wwwpkgdb=fc2733d6468c073f1dd738aa598ede55) | _ | hypothetical protein |
| [OCAR_4818](https://www.genoscope.cns.fr/agc/mage/wwwpkgdb/Info/getInfoLabel.php?id=3482547&wwwpkgdb=fc2733d6468c073f1dd738aa598ede55&nocache=a9a895ad49f0f55c362d050dc1ad3fa3&dir=&wwwpkgdb=fc2733d6468c073f1dd738aa598ede55) | _ | hypothetical protein |
| [OCAR_4820](https://www.genoscope.cns.fr/agc/mage/wwwpkgdb/Info/getInfoLabel.php?id=3482549&wwwpkgdb=fc2733d6468c073f1dd738aa598ede55&nocache=a9a895ad49f0f55c362d050dc1ad3fa3&dir=&wwwpkgdb=fc2733d6468c073f1dd738aa598ede55) | _ | site-specific DNA methylase |
| [OCAR_0802](https://www.genoscope.cns.fr/agc/mage/wwwpkgdb/Info/getInfoLabel.php?id=3482071&wwwpkgdb=fc2733d6468c073f1dd738aa598ede55&nocache=a9a895ad49f0f55c362d050dc1ad3fa3&dir=&wwwpkgdb=fc2733d6468c073f1dd738aa598ede55) | _ | _ |
| [OCAR_0803](https://www.genoscope.cns.fr/agc/mage/wwwpkgdb/Info/getInfoLabel.php?id=3482072&wwwpkgdb=fc2733d6468c073f1dd738aa598ede55&nocache=a9a895ad49f0f55c362d050dc1ad3fa3&dir=&wwwpkgdb=fc2733d6468c073f1dd738aa598ede55) | _ | _ |
| [OCAR_4822](https://www.genoscope.cns.fr/agc/mage/wwwpkgdb/Info/getInfoLabel.php?id=3484458&wwwpkgdb=fc2733d6468c073f1dd738aa598ede55&nocache=a9a895ad49f0f55c362d050dc1ad3fa3&dir=&wwwpkgdb=fc2733d6468c073f1dd738aa598ede55) | _ | hypothetical protein |
| [OCAR_4823](https://www.genoscope.cns.fr/agc/mage/wwwpkgdb/Info/getInfoLabel.php?id=3484459&wwwpkgdb=fc2733d6468c073f1dd738aa598ede55&nocache=a9a895ad49f0f55c362d050dc1ad3fa3&dir=&wwwpkgdb=fc2733d6468c073f1dd738aa598ede55) | _ | hypothetical protein |
| [OCAR_4824](https://www.genoscope.cns.fr/agc/mage/wwwpkgdb/Info/getInfoLabel.php?id=3482550&wwwpkgdb=fc2733d6468c073f1dd738aa598ede55&nocache=a9a895ad49f0f55c362d050dc1ad3fa3&dir=&wwwpkgdb=fc2733d6468c073f1dd738aa598ede55) | _ | hypothetical protein |
| [OCAR_4826](https://www.genoscope.cns.fr/agc/mage/wwwpkgdb/Info/getInfoLabel.php?id=3482552&wwwpkgdb=fc2733d6468c073f1dd738aa598ede55&nocache=a9a895ad49f0f55c362d050dc1ad3fa3&dir=&wwwpkgdb=fc2733d6468c073f1dd738aa598ede55) | _ | hypothetical protein |
| [OCAR_4827](https://www.genoscope.cns.fr/agc/mage/wwwpkgdb/Info/getInfoLabel.php?id=3482553&wwwpkgdb=fc2733d6468c073f1dd738aa598ede55&nocache=a9a895ad49f0f55c362d050dc1ad3fa3&dir=&wwwpkgdb=fc2733d6468c073f1dd738aa598ede55) | _ | phage portal protein of lambda family |
| [OCAR_4828](https://www.genoscope.cns.fr/agc/mage/wwwpkgdb/Info/getInfoLabel.php?id=3482554&wwwpkgdb=fc2733d6468c073f1dd738aa598ede55&nocache=a9a895ad49f0f55c362d050dc1ad3fa3&dir=&wwwpkgdb=fc2733d6468c073f1dd738aa598ede55) | _ | periplasmic serine protease |
| [OCAR_4829](https://www.genoscope.cns.fr/agc/mage/wwwpkgdb/Info/getInfoLabel.php?id=3482555&wwwpkgdb=fc2733d6468c073f1dd738aa598ede55&nocache=a9a895ad49f0f55c362d050dc1ad3fa3&dir=&wwwpkgdb=fc2733d6468c073f1dd738aa598ede55) | _ | hypothetical protein |
| [OCAR_4831](https://www.genoscope.cns.fr/agc/mage/wwwpkgdb/Info/getInfoLabel.php?id=3482557&wwwpkgdb=fc2733d6468c073f1dd738aa598ede55&nocache=a9a895ad49f0f55c362d050dc1ad3fa3&dir=&wwwpkgdb=fc2733d6468c073f1dd738aa598ede55) | _ | hypothetical protein |
| [OCAR_4832](https://www.genoscope.cns.fr/agc/mage/wwwpkgdb/Info/getInfoLabel.php?id=3482558&wwwpkgdb=fc2733d6468c073f1dd738aa598ede55&nocache=a9a895ad49f0f55c362d050dc1ad3fa3&dir=&wwwpkgdb=fc2733d6468c073f1dd738aa598ede55) | _ | hypothetical protein |
| [OCAR_4833](https://www.genoscope.cns.fr/agc/mage/wwwpkgdb/Info/getInfoLabel.php?id=3482559&wwwpkgdb=fc2733d6468c073f1dd738aa598ede55&nocache=a9a895ad49f0f55c362d050dc1ad3fa3&dir=&wwwpkgdb=fc2733d6468c073f1dd738aa598ede55) | _ | hypothetical protein |
| [OCAR_4834](https://www.genoscope.cns.fr/agc/mage/wwwpkgdb/Info/getInfoLabel.php?id=3482560&wwwpkgdb=fc2733d6468c073f1dd738aa598ede55&nocache=a9a895ad49f0f55c362d050dc1ad3fa3&dir=&wwwpkgdb=fc2733d6468c073f1dd738aa598ede55) | _ | hypothetical protein |
| [OCAR_4835](https://www.genoscope.cns.fr/agc/mage/wwwpkgdb/Info/getInfoLabel.php?id=3484460&wwwpkgdb=fc2733d6468c073f1dd738aa598ede55&nocache=a9a895ad49f0f55c362d050dc1ad3fa3&dir=&wwwpkgdb=fc2733d6468c073f1dd738aa598ede55) | _ | transcriptional regulator of XRE family |
| [OCAR_4836](https://www.genoscope.cns.fr/agc/mage/wwwpkgdb/Info/getInfoLabel.php?id=3484461&wwwpkgdb=fc2733d6468c073f1dd738aa598ede55&nocache=a9a895ad49f0f55c362d050dc1ad3fa3&dir=&wwwpkgdb=fc2733d6468c073f1dd738aa598ede55) | _ | hypothetical protein |
| [OCAR_4837](https://www.genoscope.cns.fr/agc/mage/wwwpkgdb/Info/getInfoLabel.php?id=3482561&wwwpkgdb=fc2733d6468c073f1dd738aa598ede55&nocache=a9a895ad49f0f55c362d050dc1ad3fa3&dir=&wwwpkgdb=fc2733d6468c073f1dd738aa598ede55) | _ | hypothetical protein |
| [OCAR_4838](https://www.genoscope.cns.fr/agc/mage/wwwpkgdb/Info/getInfoLabel.php?id=3482562&wwwpkgdb=fc2733d6468c073f1dd738aa598ede55&nocache=a9a895ad49f0f55c362d050dc1ad3fa3&dir=&wwwpkgdb=fc2733d6468c073f1dd738aa598ede55) | _ | hypothetical protein |
| [OCAR_4839](https://www.genoscope.cns.fr/agc/mage/wwwpkgdb/Info/getInfoLabel.php?id=3482563&wwwpkgdb=fc2733d6468c073f1dd738aa598ede55&nocache=a9a895ad49f0f55c362d050dc1ad3fa3&dir=&wwwpkgdb=fc2733d6468c073f1dd738aa598ede55) | _ | phage-related minor tail protein |
| [OCAR_4845](https://www.genoscope.cns.fr/agc/mage/wwwpkgdb/Info/getInfoLabel.php?id=3482569&wwwpkgdb=fc2733d6468c073f1dd738aa598ede55&nocache=a9a895ad49f0f55c362d050dc1ad3fa3&dir=&wwwpkgdb=fc2733d6468c073f1dd738aa598ede55) | _ | hypothetical protein |
| [OCAR_4846](https://www.genoscope.cns.fr/agc/mage/wwwpkgdb/Info/getInfoLabel.php?id=3482570&wwwpkgdb=fc2733d6468c073f1dd738aa598ede55&nocache=a9a895ad49f0f55c362d050dc1ad3fa3&dir=&wwwpkgdb=fc2733d6468c073f1dd738aa598ede55) | _ | hypothetical protein |
| [OCAR_4847](https://www.genoscope.cns.fr/agc/mage/wwwpkgdb/Info/getInfoLabel.php?id=3482571&wwwpkgdb=fc2733d6468c073f1dd738aa598ede55&nocache=a9a895ad49f0f55c362d050dc1ad3fa3&dir=&wwwpkgdb=fc2733d6468c073f1dd738aa598ede55) | _ | secretion activator protein |
| [OCAR_4848](https://www.genoscope.cns.fr/agc/mage/wwwpkgdb/Info/getInfoLabel.php?id=3482572&wwwpkgdb=fc2733d6468c073f1dd738aa598ede55&nocache=a9a895ad49f0f55c362d050dc1ad3fa3&dir=&wwwpkgdb=fc2733d6468c073f1dd738aa598ede55) | _ | hypothetical protein |
| [OCAR_4849](https://www.genoscope.cns.fr/agc/mage/wwwpkgdb/Info/getInfoLabel.php?id=3482573&wwwpkgdb=fc2733d6468c073f1dd738aa598ede55&nocache=a9a895ad49f0f55c362d050dc1ad3fa3&dir=&wwwpkgdb=fc2733d6468c073f1dd738aa598ede55) | _ | transcriptional regulator |
| [OCAR_4850](https://www.genoscope.cns.fr/agc/mage/wwwpkgdb/Info/getInfoLabel.php?id=3482574&wwwpkgdb=fc2733d6468c073f1dd738aa598ede55&nocache=a9a895ad49f0f55c362d050dc1ad3fa3&dir=&wwwpkgdb=fc2733d6468c073f1dd738aa598ede55) | _ | hypothetical protein |
| [OCAR_4851](https://www.genoscope.cns.fr/agc/mage/wwwpkgdb/Info/getInfoLabel.php?id=3482575&wwwpkgdb=fc2733d6468c073f1dd738aa598ede55&nocache=a9a895ad49f0f55c362d050dc1ad3fa3&dir=&wwwpkgdb=fc2733d6468c073f1dd738aa598ede55) | _ | efflux transporter, RND family, MFP subunit |
| [OCAR_4852](https://www.genoscope.cns.fr/agc/mage/wwwpkgdb/Info/getInfoLabel.php?id=3482576&wwwpkgdb=fc2733d6468c073f1dd738aa598ede55&nocache=a9a895ad49f0f55c362d050dc1ad3fa3&dir=&wwwpkgdb=fc2733d6468c073f1dd738aa598ede55) | _ | ABC transporter permease protein |
| [OCAR_0836](https://www.genoscope.cns.fr/agc/mage/wwwpkgdb/Info/getInfoLabel.php?id=3482069&wwwpkgdb=fc2733d6468c073f1dd738aa598ede55&nocache=a9a895ad49f0f55c362d050dc1ad3fa3&dir=&wwwpkgdb=fc2733d6468c073f1dd738aa598ede55) | _ | _ |
| [OCAR_0837](https://www.genoscope.cns.fr/agc/mage/wwwpkgdb/Info/getInfoLabel.php?id=3482070&wwwpkgdb=fc2733d6468c073f1dd738aa598ede55&nocache=a9a895ad49f0f55c362d050dc1ad3fa3&dir=&wwwpkgdb=fc2733d6468c073f1dd738aa598ede55) | _ | _ |
| [OCAR_4854](https://www.genoscope.cns.fr/agc/mage/wwwpkgdb/Info/getInfoLabel.php?id=3484462&wwwpkgdb=fc2733d6468c073f1dd738aa598ede55&nocache=a9a895ad49f0f55c362d050dc1ad3fa3&dir=&wwwpkgdb=fc2733d6468c073f1dd738aa598ede55) | _ | hypothetical protein |
| [OCAR_0838](https://www.genoscope.cns.fr/agc/mage/wwwpkgdb/Info/getInfoLabel.php?id=3482068&wwwpkgdb=fc2733d6468c073f1dd738aa598ede55&nocache=a9a895ad49f0f55c362d050dc1ad3fa3&dir=&wwwpkgdb=fc2733d6468c073f1dd738aa598ede55) | _ | _ |
| [OCAR_0839](https://www.genoscope.cns.fr/agc/mage/wwwpkgdb/Info/getInfoLabel.php?id=3482067&wwwpkgdb=fc2733d6468c073f1dd738aa598ede55&nocache=a9a895ad49f0f55c362d050dc1ad3fa3&dir=&wwwpkgdb=fc2733d6468c073f1dd738aa598ede55) | _ | _ |
| [OCAR_4855](https://www.genoscope.cns.fr/agc/mage/wwwpkgdb/Info/getInfoLabel.php?id=3484463&wwwpkgdb=fc2733d6468c073f1dd738aa598ede55&nocache=a9a895ad49f0f55c362d050dc1ad3fa3&dir=&wwwpkgdb=fc2733d6468c073f1dd738aa598ede55) | _ | hypothetical protein |
| [OCAR_4856](https://www.genoscope.cns.fr/agc/mage/wwwpkgdb/Info/getInfoLabel.php?id=3484464&wwwpkgdb=fc2733d6468c073f1dd738aa598ede55&nocache=a9a895ad49f0f55c362d050dc1ad3fa3&dir=&wwwpkgdb=fc2733d6468c073f1dd738aa598ede55) | _ | OsmC family protein |
| [OCAR_4857](https://www.genoscope.cns.fr/agc/mage/wwwpkgdb/Info/getInfoLabel.php?id=3484465&wwwpkgdb=fc2733d6468c073f1dd738aa598ede55&nocache=a9a895ad49f0f55c362d050dc1ad3fa3&dir=&wwwpkgdb=fc2733d6468c073f1dd738aa598ede55) | _ | putative ABC transporter, permease protein |
| [OCAR_4858](https://www.genoscope.cns.fr/agc/mage/wwwpkgdb/Info/getInfoLabel.php?id=3484466&wwwpkgdb=fc2733d6468c073f1dd738aa598ede55&nocache=a9a895ad49f0f55c362d050dc1ad3fa3&dir=&wwwpkgdb=fc2733d6468c073f1dd738aa598ede55) | _ | putative ABC transporter, permease protein |
| [OCAR_4860](https://www.genoscope.cns.fr/agc/mage/wwwpkgdb/Info/getInfoLabel.php?id=3484468&wwwpkgdb=fc2733d6468c073f1dd738aa598ede55&nocache=a9a895ad49f0f55c362d050dc1ad3fa3&dir=&wwwpkgdb=fc2733d6468c073f1dd738aa598ede55) | _ | secretion protein HlyD family protein |
| [OCAR_4861](https://www.genoscope.cns.fr/agc/mage/wwwpkgdb/Info/getInfoLabel.php?id=3484469&wwwpkgdb=fc2733d6468c073f1dd738aa598ede55&nocache=a9a895ad49f0f55c362d050dc1ad3fa3&dir=&wwwpkgdb=fc2733d6468c073f1dd738aa598ede55) | _ | transcriptional regulator of TetR family protein |
| [OCAR_0847](https://www.genoscope.cns.fr/agc/mage/wwwpkgdb/Info/getInfoLabel.php?id=3482066&wwwpkgdb=fc2733d6468c073f1dd738aa598ede55&nocache=a9a895ad49f0f55c362d050dc1ad3fa3&dir=&wwwpkgdb=fc2733d6468c073f1dd738aa598ede55) | _ | _ |
| [OCAR_0853](https://www.genoscope.cns.fr/agc/mage/wwwpkgdb/Info/getInfoLabel.php?id=3482065&wwwpkgdb=fc2733d6468c073f1dd738aa598ede55&nocache=a9a895ad49f0f55c362d050dc1ad3fa3&dir=&wwwpkgdb=fc2733d6468c073f1dd738aa598ede55) | _ | _ |
| [OCAR_4868](https://www.genoscope.cns.fr/agc/mage/wwwpkgdb/Info/getInfoLabel.php?id=3484470&wwwpkgdb=fc2733d6468c073f1dd738aa598ede55&nocache=a9a895ad49f0f55c362d050dc1ad3fa3&dir=&wwwpkgdb=fc2733d6468c073f1dd738aa598ede55) | _ | hypothetical protein |
| [OCAR_4869](https://www.genoscope.cns.fr/agc/mage/wwwpkgdb/Info/getInfoLabel.php?id=3482584&wwwpkgdb=fc2733d6468c073f1dd738aa598ede55&nocache=a9a895ad49f0f55c362d050dc1ad3fa3&dir=&wwwpkgdb=fc2733d6468c073f1dd738aa598ede55) | _ | hypothetical protein |
| [OCAR_0856](https://www.genoscope.cns.fr/agc/mage/wwwpkgdb/Info/getInfoLabel.php?id=3482064&wwwpkgdb=fc2733d6468c073f1dd738aa598ede55&nocache=a9a895ad49f0f55c362d050dc1ad3fa3&dir=&wwwpkgdb=fc2733d6468c073f1dd738aa598ede55) | _ | _ |
| [OCAR_4870](https://www.genoscope.cns.fr/agc/mage/wwwpkgdb/Info/getInfoLabel.php?id=3482585&wwwpkgdb=fc2733d6468c073f1dd738aa598ede55&nocache=a9a895ad49f0f55c362d050dc1ad3fa3&dir=&wwwpkgdb=fc2733d6468c073f1dd738aa598ede55) | _ | hypothetical protein |
| [OCAR_4872](https://www.genoscope.cns.fr/agc/mage/wwwpkgdb/Info/getInfoLabel.php?id=3482587&wwwpkgdb=fc2733d6468c073f1dd738aa598ede55&nocache=a9a895ad49f0f55c362d050dc1ad3fa3&dir=&wwwpkgdb=fc2733d6468c073f1dd738aa598ede55) | _ | H3U |
| [OCAR_4873](https://www.genoscope.cns.fr/agc/mage/wwwpkgdb/Info/getInfoLabel.php?id=3484471&wwwpkgdb=fc2733d6468c073f1dd738aa598ede55&nocache=a9a895ad49f0f55c362d050dc1ad3fa3&dir=&wwwpkgdb=fc2733d6468c073f1dd738aa598ede55) | _ | sensor histidine kinase |
| [OCAR_4875](https://www.genoscope.cns.fr/agc/mage/wwwpkgdb/Info/getInfoLabel.php?id=3482588&wwwpkgdb=fc2733d6468c073f1dd738aa598ede55&nocache=a9a895ad49f0f55c362d050dc1ad3fa3&dir=&wwwpkgdb=fc2733d6468c073f1dd738aa598ede55) | _ | hypothetical Cytosolic Protein |
| [OCAR_4877](https://www.genoscope.cns.fr/agc/mage/wwwpkgdb/Info/getInfoLabel.php?id=3482590&wwwpkgdb=fc2733d6468c073f1dd738aa598ede55&nocache=a9a895ad49f0f55c362d050dc1ad3fa3&dir=&wwwpkgdb=fc2733d6468c073f1dd738aa598ede55) | _ | hypothetical protein |
| [OCAR_4878](https://www.genoscope.cns.fr/agc/mage/wwwpkgdb/Info/getInfoLabel.php?id=3482591&wwwpkgdb=fc2733d6468c073f1dd738aa598ede55&nocache=a9a895ad49f0f55c362d050dc1ad3fa3&dir=&wwwpkgdb=fc2733d6468c073f1dd738aa598ede55) | _ | hypothetical protein |
| [OCAR_4879](https://www.genoscope.cns.fr/agc/mage/wwwpkgdb/Info/getInfoLabel.php?id=3482592&wwwpkgdb=fc2733d6468c073f1dd738aa598ede55&nocache=a9a895ad49f0f55c362d050dc1ad3fa3&dir=&wwwpkgdb=fc2733d6468c073f1dd738aa598ede55) | _ | sulfite reductase |
| [OCAR_4881](https://www.genoscope.cns.fr/agc/mage/wwwpkgdb/Info/getInfoLabel.php?id=3482594&wwwpkgdb=fc2733d6468c073f1dd738aa598ede55&nocache=a9a895ad49f0f55c362d050dc1ad3fa3&dir=&wwwpkgdb=fc2733d6468c073f1dd738aa598ede55) | _ | hypothetical protein |
| [OCAR_4883](https://www.genoscope.cns.fr/agc/mage/wwwpkgdb/Info/getInfoLabel.php?id=3482595&wwwpkgdb=fc2733d6468c073f1dd738aa598ede55&nocache=a9a895ad49f0f55c362d050dc1ad3fa3&dir=&wwwpkgdb=fc2733d6468c073f1dd738aa598ede55) | _ | hypothetical protein |
| [OCAR_4889](https://www.genoscope.cns.fr/agc/mage/wwwpkgdb/Info/getInfoLabel.php?id=3484478&wwwpkgdb=fc2733d6468c073f1dd738aa598ede55&nocache=a9a895ad49f0f55c362d050dc1ad3fa3&dir=&wwwpkgdb=fc2733d6468c073f1dd738aa598ede55) | _ | hypothetical protein |
| [OCAR_4893](https://www.genoscope.cns.fr/agc/mage/wwwpkgdb/Info/getInfoLabel.php?id=3484479&wwwpkgdb=fc2733d6468c073f1dd738aa598ede55&nocache=a9a895ad49f0f55c362d050dc1ad3fa3&dir=&wwwpkgdb=fc2733d6468c073f1dd738aa598ede55) | _ | hypothetical protein |
| [OCAR_4895](https://www.genoscope.cns.fr/agc/mage/wwwpkgdb/Info/getInfoLabel.php?id=3484480&wwwpkgdb=fc2733d6468c073f1dd738aa598ede55&nocache=a9a895ad49f0f55c362d050dc1ad3fa3&dir=&wwwpkgdb=fc2733d6468c073f1dd738aa598ede55) | _ | hypothetical protein |
| [OCAR_0886](https://www.genoscope.cns.fr/agc/mage/wwwpkgdb/Info/getInfoLabel.php?id=3482063&wwwpkgdb=fc2733d6468c073f1dd738aa598ede55&nocache=a9a895ad49f0f55c362d050dc1ad3fa3&dir=&wwwpkgdb=fc2733d6468c073f1dd738aa598ede55) | _ | _ |
| [OCAR_0889](https://www.genoscope.cns.fr/agc/mage/wwwpkgdb/Info/getInfoLabel.php?id=3482062&wwwpkgdb=fc2733d6468c073f1dd738aa598ede55&nocache=a9a895ad49f0f55c362d050dc1ad3fa3&dir=&wwwpkgdb=fc2733d6468c073f1dd738aa598ede55) | _ | _ |
| [OCAR_4914](https://www.genoscope.cns.fr/agc/mage/wwwpkgdb/Info/getInfoLabel.php?id=3482615&wwwpkgdb=fc2733d6468c073f1dd738aa598ede55&nocache=a9a895ad49f0f55c362d050dc1ad3fa3&dir=&wwwpkgdb=fc2733d6468c073f1dd738aa598ede55) | _ | hypothetical protein |
| [OCAR_4916](https://www.genoscope.cns.fr/agc/mage/wwwpkgdb/Info/getInfoLabel.php?id=3482616&wwwpkgdb=fc2733d6468c073f1dd738aa598ede55&nocache=a9a895ad49f0f55c362d050dc1ad3fa3&dir=&wwwpkgdb=fc2733d6468c073f1dd738aa598ede55) | _ | hypothetical protein |
| [OCAR_4919](https://www.genoscope.cns.fr/agc/mage/wwwpkgdb/Info/getInfoLabel.php?id=3482617&wwwpkgdb=fc2733d6468c073f1dd738aa598ede55&nocache=a9a895ad49f0f55c362d050dc1ad3fa3&dir=&wwwpkgdb=fc2733d6468c073f1dd738aa598ede55) | _ | hypothetical protein |
| [OCAR_0904](https://www.genoscope.cns.fr/agc/mage/wwwpkgdb/Info/getInfoLabel.php?id=3482061&wwwpkgdb=fc2733d6468c073f1dd738aa598ede55&nocache=a9a895ad49f0f55c362d050dc1ad3fa3&dir=&wwwpkgdb=fc2733d6468c073f1dd738aa598ede55) | _ | _ |
| [OCAR_4927](https://www.genoscope.cns.fr/agc/mage/wwwpkgdb/Info/getInfoLabel.php?id=3482624&wwwpkgdb=fc2733d6468c073f1dd738aa598ede55&nocache=a9a895ad49f0f55c362d050dc1ad3fa3&dir=&wwwpkgdb=fc2733d6468c073f1dd738aa598ede55) | _ | hypothetical protein |
| [OCAR_4929](https://www.genoscope.cns.fr/agc/mage/wwwpkgdb/Info/getInfoLabel.php?id=3482625&wwwpkgdb=fc2733d6468c073f1dd738aa598ede55&nocache=a9a895ad49f0f55c362d050dc1ad3fa3&dir=&wwwpkgdb=fc2733d6468c073f1dd738aa598ede55) | _ | hypothetical protein |
| [OCAR_0914](https://www.genoscope.cns.fr/agc/mage/wwwpkgdb/Info/getInfoLabel.php?id=3482060&wwwpkgdb=fc2733d6468c073f1dd738aa598ede55&nocache=a9a895ad49f0f55c362d050dc1ad3fa3&dir=&wwwpkgdb=fc2733d6468c073f1dd738aa598ede55) | _ | _ |
| [OCAR_4933](https://www.genoscope.cns.fr/agc/mage/wwwpkgdb/Info/getInfoLabel.php?id=3482629&wwwpkgdb=fc2733d6468c073f1dd738aa598ede55&nocache=a9a895ad49f0f55c362d050dc1ad3fa3&dir=&wwwpkgdb=fc2733d6468c073f1dd738aa598ede55) | _ | hypothetical protein |
| [OCAR_0918](https://www.genoscope.cns.fr/agc/mage/wwwpkgdb/Info/getInfoLabel.php?id=3482136&wwwpkgdb=fc2733d6468c073f1dd738aa598ede55&nocache=a9a895ad49f0f55c362d050dc1ad3fa3&dir=&wwwpkgdb=fc2733d6468c073f1dd738aa598ede55) | _ | _ |
| [OCAR_0933](https://www.genoscope.cns.fr/agc/mage/wwwpkgdb/Info/getInfoLabel.php?id=3482059&wwwpkgdb=fc2733d6468c073f1dd738aa598ede55&nocache=a9a895ad49f0f55c362d050dc1ad3fa3&dir=&wwwpkgdb=fc2733d6468c073f1dd738aa598ede55) | _ | _ |
| [OCAR_4949](https://www.genoscope.cns.fr/agc/mage/wwwpkgdb/Info/getInfoLabel.php?id=3482637&wwwpkgdb=fc2733d6468c073f1dd738aa598ede55&nocache=a9a895ad49f0f55c362d050dc1ad3fa3&dir=&wwwpkgdb=fc2733d6468c073f1dd738aa598ede55) | _ | hypothetical protein |
| [OCAR_4951](https://www.genoscope.cns.fr/agc/mage/wwwpkgdb/Info/getInfoLabel.php?id=3484498&wwwpkgdb=fc2733d6468c073f1dd738aa598ede55&nocache=a9a895ad49f0f55c362d050dc1ad3fa3&dir=&wwwpkgdb=fc2733d6468c073f1dd738aa598ede55) | _ | integral membrane protein |
| [OCAR_4953](https://www.genoscope.cns.fr/agc/mage/wwwpkgdb/Info/getInfoLabel.php?id=3484499&wwwpkgdb=fc2733d6468c073f1dd738aa598ede55&nocache=a9a895ad49f0f55c362d050dc1ad3fa3&dir=&wwwpkgdb=fc2733d6468c073f1dd738aa598ede55) | _ | hypothetical protein |
| [OCAR_0940](https://www.genoscope.cns.fr/agc/mage/wwwpkgdb/Info/getInfoLabel.php?id=3482135&wwwpkgdb=fc2733d6468c073f1dd738aa598ede55&nocache=a9a895ad49f0f55c362d050dc1ad3fa3&dir=&wwwpkgdb=fc2733d6468c073f1dd738aa598ede55) | _ | _ |
| [OCAR_4957](https://www.genoscope.cns.fr/agc/mage/wwwpkgdb/Info/getInfoLabel.php?id=3484503&wwwpkgdb=fc2733d6468c073f1dd738aa598ede55&nocache=a9a895ad49f0f55c362d050dc1ad3fa3&dir=&wwwpkgdb=fc2733d6468c073f1dd738aa598ede55) | _ | hypothetical protein |
| [OCAR_4958](https://www.genoscope.cns.fr/agc/mage/wwwpkgdb/Info/getInfoLabel.php?id=3484504&wwwpkgdb=fc2733d6468c073f1dd738aa598ede55&nocache=a9a895ad49f0f55c362d050dc1ad3fa3&dir=&wwwpkgdb=fc2733d6468c073f1dd738aa598ede55) | _ | hypothetical protein |
| [OCAR_4960](https://www.genoscope.cns.fr/agc/mage/wwwpkgdb/Info/getInfoLabel.php?id=3482640&wwwpkgdb=fc2733d6468c073f1dd738aa598ede55&nocache=a9a895ad49f0f55c362d050dc1ad3fa3&dir=&wwwpkgdb=fc2733d6468c073f1dd738aa598ede55) | _ | hypothetical protein |
| [OCAR_0944](https://www.genoscope.cns.fr/agc/mage/wwwpkgdb/Info/getInfoLabel.php?id=3482058&wwwpkgdb=fc2733d6468c073f1dd738aa598ede55&nocache=a9a895ad49f0f55c362d050dc1ad3fa3&dir=&wwwpkgdb=fc2733d6468c073f1dd738aa598ede55) | _ | _ |
| [OCAR_0948](https://www.genoscope.cns.fr/agc/mage/wwwpkgdb/Info/getInfoLabel.php?id=3482057&wwwpkgdb=fc2733d6468c073f1dd738aa598ede55&nocache=a9a895ad49f0f55c362d050dc1ad3fa3&dir=&wwwpkgdb=fc2733d6468c073f1dd738aa598ede55) | _ | _ |
| [OCAR_4964](https://www.genoscope.cns.fr/agc/mage/wwwpkgdb/Info/getInfoLabel.php?id=3482641&wwwpkgdb=fc2733d6468c073f1dd738aa598ede55&nocache=a9a895ad49f0f55c362d050dc1ad3fa3&dir=&wwwpkgdb=fc2733d6468c073f1dd738aa598ede55) | _ | hypothetical protein |
| [OCAR_4965](https://www.genoscope.cns.fr/agc/mage/wwwpkgdb/Info/getInfoLabel.php?id=3482642&wwwpkgdb=fc2733d6468c073f1dd738aa598ede55&nocache=a9a895ad49f0f55c362d050dc1ad3fa3&dir=&wwwpkgdb=fc2733d6468c073f1dd738aa598ede55) | _ | hypothetical protein |
| [OCAR_4966](https://www.genoscope.cns.fr/agc/mage/wwwpkgdb/Info/getInfoLabel.php?id=3482643&wwwpkgdb=fc2733d6468c073f1dd738aa598ede55&nocache=a9a895ad49f0f55c362d050dc1ad3fa3&dir=&wwwpkgdb=fc2733d6468c073f1dd738aa598ede55) | _ | hypothetical protein |
| [OCAR_4968](https://www.genoscope.cns.fr/agc/mage/wwwpkgdb/Info/getInfoLabel.php?id=3482645&wwwpkgdb=fc2733d6468c073f1dd738aa598ede55&nocache=a9a895ad49f0f55c362d050dc1ad3fa3&dir=&wwwpkgdb=fc2733d6468c073f1dd738aa598ede55) | _ | putative flavin-nucleotide-binding protein |
| [OCAR_4970](https://www.genoscope.cns.fr/agc/mage/wwwpkgdb/Info/getInfoLabel.php?id=3482646&wwwpkgdb=fc2733d6468c073f1dd738aa598ede55&nocache=a9a895ad49f0f55c362d050dc1ad3fa3&dir=&wwwpkgdb=fc2733d6468c073f1dd738aa598ede55) | _ | putative esterase |
| [OCAR_0956](https://www.genoscope.cns.fr/agc/mage/wwwpkgdb/Info/getInfoLabel.php?id=3482055&wwwpkgdb=fc2733d6468c073f1dd738aa598ede55&nocache=a9a895ad49f0f55c362d050dc1ad3fa3&dir=&wwwpkgdb=fc2733d6468c073f1dd738aa598ede55) | _ | _ |
| [OCAR_0957](https://www.genoscope.cns.fr/agc/mage/wwwpkgdb/Info/getInfoLabel.php?id=3482054&wwwpkgdb=fc2733d6468c073f1dd738aa598ede55&nocache=a9a895ad49f0f55c362d050dc1ad3fa3&dir=&wwwpkgdb=fc2733d6468c073f1dd738aa598ede55) | _ | _ |
| [OCAR_4971](https://www.genoscope.cns.fr/agc/mage/wwwpkgdb/Info/getInfoLabel.php?id=3482647&wwwpkgdb=fc2733d6468c073f1dd738aa598ede55&nocache=a9a895ad49f0f55c362d050dc1ad3fa3&dir=&wwwpkgdb=fc2733d6468c073f1dd738aa598ede55) | _ | trap-type c4-dicarboxylate transport system |
| [OCAR_0959](https://www.genoscope.cns.fr/agc/mage/wwwpkgdb/Info/getInfoLabel.php?id=3482053&wwwpkgdb=fc2733d6468c073f1dd738aa598ede55&nocache=a9a895ad49f0f55c362d050dc1ad3fa3&dir=&wwwpkgdb=fc2733d6468c073f1dd738aa598ede55) | _ | _ |
| [OCAR_0961](https://www.genoscope.cns.fr/agc/mage/wwwpkgdb/Info/getInfoLabel.php?id=3482052&wwwpkgdb=fc2733d6468c073f1dd738aa598ede55&nocache=a9a895ad49f0f55c362d050dc1ad3fa3&dir=&wwwpkgdb=fc2733d6468c073f1dd738aa598ede55) | _ | _ |
| [OCAR_0962](https://www.genoscope.cns.fr/agc/mage/wwwpkgdb/Info/getInfoLabel.php?id=3482051&wwwpkgdb=fc2733d6468c073f1dd738aa598ede55&nocache=a9a895ad49f0f55c362d050dc1ad3fa3&dir=&wwwpkgdb=fc2733d6468c073f1dd738aa598ede55) | _ | _ |
| [OCAR_4973](https://www.genoscope.cns.fr/agc/mage/wwwpkgdb/Info/getInfoLabel.php?id=3484510&wwwpkgdb=fc2733d6468c073f1dd738aa598ede55&nocache=a9a895ad49f0f55c362d050dc1ad3fa3&dir=&wwwpkgdb=fc2733d6468c073f1dd738aa598ede55) | _ | hypothetical protein |
| [OCAR_0963](https://www.genoscope.cns.fr/agc/mage/wwwpkgdb/Info/getInfoLabel.php?id=3482050&wwwpkgdb=fc2733d6468c073f1dd738aa598ede55&nocache=a9a895ad49f0f55c362d050dc1ad3fa3&dir=&wwwpkgdb=fc2733d6468c073f1dd738aa598ede55) | _ | _ |
| [OCAR_0966](https://www.genoscope.cns.fr/agc/mage/wwwpkgdb/Info/getInfoLabel.php?id=3482049&wwwpkgdb=fc2733d6468c073f1dd738aa598ede55&nocache=a9a895ad49f0f55c362d050dc1ad3fa3&dir=&wwwpkgdb=fc2733d6468c073f1dd738aa598ede55) | _ | _ |
| [OCAR_4976](https://www.genoscope.cns.fr/agc/mage/wwwpkgdb/Info/getInfoLabel.php?id=3484512&wwwpkgdb=fc2733d6468c073f1dd738aa598ede55&nocache=a9a895ad49f0f55c362d050dc1ad3fa3&dir=&wwwpkgdb=fc2733d6468c073f1dd738aa598ede55) | _ | hypothetical protein |
| [OCAR_4977](https://www.genoscope.cns.fr/agc/mage/wwwpkgdb/Info/getInfoLabel.php?id=3482650&wwwpkgdb=fc2733d6468c073f1dd738aa598ede55&nocache=a9a895ad49f0f55c362d050dc1ad3fa3&dir=&wwwpkgdb=fc2733d6468c073f1dd738aa598ede55) | _ | Na-Ca exchanger/integrin-beta4 |
| [OCAR_4980](https://www.genoscope.cns.fr/agc/mage/wwwpkgdb/Info/getInfoLabel.php?id=3484513&wwwpkgdb=fc2733d6468c073f1dd738aa598ede55&nocache=a9a895ad49f0f55c362d050dc1ad3fa3&dir=&wwwpkgdb=fc2733d6468c073f1dd738aa598ede55) | _ | phage integrase |
| [OCAR_4982](https://www.genoscope.cns.fr/agc/mage/wwwpkgdb/Info/getInfoLabel.php?id=3482653&wwwpkgdb=fc2733d6468c073f1dd738aa598ede55&nocache=a9a895ad49f0f55c362d050dc1ad3fa3&dir=&wwwpkgdb=fc2733d6468c073f1dd738aa598ede55) | _ | hypothetical protein |
| [OCAR_4983](https://www.genoscope.cns.fr/agc/mage/wwwpkgdb/Info/getInfoLabel.php?id=3484515&wwwpkgdb=fc2733d6468c073f1dd738aa598ede55&nocache=a9a895ad49f0f55c362d050dc1ad3fa3&dir=&wwwpkgdb=fc2733d6468c073f1dd738aa598ede55) | _ | hypothetical protein |
| [OCAR_4985](https://www.genoscope.cns.fr/agc/mage/wwwpkgdb/Info/getInfoLabel.php?id=3485938&wwwpkgdb=fc2733d6468c073f1dd738aa598ede55&nocache=a9a895ad49f0f55c362d050dc1ad3fa3&dir=&wwwpkgdb=fc2733d6468c073f1dd738aa598ede55) | _ | DNA methylase N-4/N-6; nonfunctional due to frameshift |
| [OCAR_4986](https://www.genoscope.cns.fr/agc/mage/wwwpkgdb/Info/getInfoLabel.php?id=3482655&wwwpkgdb=fc2733d6468c073f1dd738aa598ede55&nocache=a9a895ad49f0f55c362d050dc1ad3fa3&dir=&wwwpkgdb=fc2733d6468c073f1dd738aa598ede55) | _ | ankyrin repeat protein containing four repeats |
| [OCAR_4989](https://www.genoscope.cns.fr/agc/mage/wwwpkgdb/Info/getInfoLabel.php?id=3482656&wwwpkgdb=fc2733d6468c073f1dd738aa598ede55&nocache=a9a895ad49f0f55c362d050dc1ad3fa3&dir=&wwwpkgdb=fc2733d6468c073f1dd738aa598ede55) | _ | hypothetical protein |
| [OCAR_4999](https://www.genoscope.cns.fr/agc/mage/wwwpkgdb/Info/getInfoLabel.php?id=3482662&wwwpkgdb=fc2733d6468c073f1dd738aa598ede55&nocache=a9a895ad49f0f55c362d050dc1ad3fa3&dir=&wwwpkgdb=fc2733d6468c073f1dd738aa598ede55) | _ | hypothetical protein |
| [OCAR_5001](https://www.genoscope.cns.fr/agc/mage/wwwpkgdb/Info/getInfoLabel.php?id=3482664&wwwpkgdb=fc2733d6468c073f1dd738aa598ede55&nocache=a9a895ad49f0f55c362d050dc1ad3fa3&dir=&wwwpkgdb=fc2733d6468c073f1dd738aa598ede55) | npdG | NADPH-dependent f420 reductase |
| [OCAR_5004](https://www.genoscope.cns.fr/agc/mage/wwwpkgdb/Info/getInfoLabel.php?id=3482667&wwwpkgdb=fc2733d6468c073f1dd738aa598ede55&nocache=a9a895ad49f0f55c362d050dc1ad3fa3&dir=&wwwpkgdb=fc2733d6468c073f1dd738aa598ede55) | _ | putative F420-dependent oxidoreductase |
| [OCAR_5005](https://www.genoscope.cns.fr/agc/mage/wwwpkgdb/Info/getInfoLabel.php?id=3482668&wwwpkgdb=fc2733d6468c073f1dd738aa598ede55&nocache=a9a895ad49f0f55c362d050dc1ad3fa3&dir=&wwwpkgdb=fc2733d6468c073f1dd738aa598ede55) | cofD | lppg:fo 2-phospho-l-lactate transferase |
| [OCAR_5006](https://www.genoscope.cns.fr/agc/mage/wwwpkgdb/Info/getInfoLabel.php?id=3482669&wwwpkgdb=fc2733d6468c073f1dd738aa598ede55&nocache=a9a895ad49f0f55c362d050dc1ad3fa3&dir=&wwwpkgdb=fc2733d6468c073f1dd738aa598ede55) | _ | hypothetical protein |
| [OCAR_5007](https://www.genoscope.cns.fr/agc/mage/wwwpkgdb/Info/getInfoLabel.php?id=3482670&wwwpkgdb=fc2733d6468c073f1dd738aa598ede55&nocache=a9a895ad49f0f55c362d050dc1ad3fa3&dir=&wwwpkgdb=fc2733d6468c073f1dd738aa598ede55) | _ | FO synthase |
| [OCAR_5008](https://www.genoscope.cns.fr/agc/mage/wwwpkgdb/Info/getInfoLabel.php?id=3482671&wwwpkgdb=fc2733d6468c073f1dd738aa598ede55&nocache=a9a895ad49f0f55c362d050dc1ad3fa3&dir=&wwwpkgdb=fc2733d6468c073f1dd738aa598ede55) | _ | transcriptional regulator, MarR family |
| [OCAR_1001](https://www.genoscope.cns.fr/agc/mage/wwwpkgdb/Info/getInfoLabel.php?id=3482048&wwwpkgdb=fc2733d6468c073f1dd738aa598ede55&nocache=a9a895ad49f0f55c362d050dc1ad3fa3&dir=&wwwpkgdb=fc2733d6468c073f1dd738aa598ede55) | _ | _ |
| [OCAR_5011](https://www.genoscope.cns.fr/agc/mage/wwwpkgdb/Info/getInfoLabel.php?id=3482673&wwwpkgdb=fc2733d6468c073f1dd738aa598ede55&nocache=a9a895ad49f0f55c362d050dc1ad3fa3&dir=&wwwpkgdb=fc2733d6468c073f1dd738aa598ede55) | _ | hypothetical protein |
| [OCAR_5012](https://www.genoscope.cns.fr/agc/mage/wwwpkgdb/Info/getInfoLabel.php?id=3482674&wwwpkgdb=fc2733d6468c073f1dd738aa598ede55&nocache=a9a895ad49f0f55c362d050dc1ad3fa3&dir=&wwwpkgdb=fc2733d6468c073f1dd738aa598ede55) | _ | hypothetical protein |
| [OCAR_5014](https://www.genoscope.cns.fr/agc/mage/wwwpkgdb/Info/getInfoLabel.php?id=3484523&wwwpkgdb=fc2733d6468c073f1dd738aa598ede55&nocache=a9a895ad49f0f55c362d050dc1ad3fa3&dir=&wwwpkgdb=fc2733d6468c073f1dd738aa598ede55) | _ | activator of Hsp90 ATPase 1 family protein |
| [OCAR_5019](https://www.genoscope.cns.fr/agc/mage/wwwpkgdb/Info/getInfoLabel.php?id=3484527&wwwpkgdb=fc2733d6468c073f1dd738aa598ede55&nocache=a9a895ad49f0f55c362d050dc1ad3fa3&dir=&wwwpkgdb=fc2733d6468c073f1dd738aa598ede55) | _ | dihydrolipoyllysine-residue succinyltransferase component of 2-oxoglutarate dehydrogenase complex (E2) |
| [OCAR_1024](https://www.genoscope.cns.fr/agc/mage/wwwpkgdb/Info/getInfoLabel.php?id=3482047&wwwpkgdb=fc2733d6468c073f1dd738aa598ede55&nocache=a9a895ad49f0f55c362d050dc1ad3fa3&dir=&wwwpkgdb=fc2733d6468c073f1dd738aa598ede55) | _ | _ |
| [OCAR_1029](https://www.genoscope.cns.fr/agc/mage/wwwpkgdb/Info/getInfoLabel.php?id=3482046&wwwpkgdb=fc2733d6468c073f1dd738aa598ede55&nocache=a9a895ad49f0f55c362d050dc1ad3fa3&dir=&wwwpkgdb=fc2733d6468c073f1dd738aa598ede55) | _ | _ |
| [OCAR_5039](https://www.genoscope.cns.fr/agc/mage/wwwpkgdb/Info/getInfoLabel.php?id=3482686&wwwpkgdb=fc2733d6468c073f1dd738aa598ede55&nocache=a9a895ad49f0f55c362d050dc1ad3fa3&dir=&wwwpkgdb=fc2733d6468c073f1dd738aa598ede55) | _ | hypothetical protein |
| [OCAR_5041](https://www.genoscope.cns.fr/agc/mage/wwwpkgdb/Info/getInfoLabel.php?id=3484539&wwwpkgdb=fc2733d6468c073f1dd738aa598ede55&nocache=a9a895ad49f0f55c362d050dc1ad3fa3&dir=&wwwpkgdb=fc2733d6468c073f1dd738aa598ede55) | _ | cyclic nucleotide-binding protein |
| [OCAR_5045](https://www.genoscope.cns.fr/agc/mage/wwwpkgdb/Info/getInfoLabel.php?id=3482690&wwwpkgdb=fc2733d6468c073f1dd738aa598ede55&nocache=a9a895ad49f0f55c362d050dc1ad3fa3&dir=&wwwpkgdb=fc2733d6468c073f1dd738aa598ede55) | _ | respiratory nitrate reductase 2 delta chain |
| [OCAR_5047](https://www.genoscope.cns.fr/agc/mage/wwwpkgdb/Info/getInfoLabel.php?id=3482692&wwwpkgdb=fc2733d6468c073f1dd738aa598ede55&nocache=a9a895ad49f0f55c362d050dc1ad3fa3&dir=&wwwpkgdb=fc2733d6468c073f1dd738aa598ede55) | _ | PpiC-type peptidyl-prolyl cis-trans isomerase |
| [OCAR_5048](https://www.genoscope.cns.fr/agc/mage/wwwpkgdb/Info/getInfoLabel.php?id=3482693&wwwpkgdb=fc2733d6468c073f1dd738aa598ede55&nocache=a9a895ad49f0f55c362d050dc1ad3fa3&dir=&wwwpkgdb=fc2733d6468c073f1dd738aa598ede55) | _ | hypothetical protein |
| [OCAR_5049](https://www.genoscope.cns.fr/agc/mage/wwwpkgdb/Info/getInfoLabel.php?id=3482694&wwwpkgdb=fc2733d6468c073f1dd738aa598ede55&nocache=a9a895ad49f0f55c362d050dc1ad3fa3&dir=&wwwpkgdb=fc2733d6468c073f1dd738aa598ede55) | _ | molybdopterin biosynthesis mog protein |
| [OCAR_5050](https://www.genoscope.cns.fr/agc/mage/wwwpkgdb/Info/getInfoLabel.php?id=3484540&wwwpkgdb=fc2733d6468c073f1dd738aa598ede55&nocache=a9a895ad49f0f55c362d050dc1ad3fa3&dir=&wwwpkgdb=fc2733d6468c073f1dd738aa598ede55) | _ | hypothetical protein |
| [OCAR_5055](https://www.genoscope.cns.fr/agc/mage/wwwpkgdb/Info/getInfoLabel.php?id=3484543&wwwpkgdb=fc2733d6468c073f1dd738aa598ede55&nocache=a9a895ad49f0f55c362d050dc1ad3fa3&dir=&wwwpkgdb=fc2733d6468c073f1dd738aa598ede55) | _ | periplasmic binding protein |
| [OCAR_5057](https://www.genoscope.cns.fr/agc/mage/wwwpkgdb/Info/getInfoLabel.php?id=3484544&wwwpkgdb=fc2733d6468c073f1dd738aa598ede55&nocache=a9a895ad49f0f55c362d050dc1ad3fa3&dir=&wwwpkgdb=fc2733d6468c073f1dd738aa598ede55) | _ | hypothetical protein |
| [OCAR_5059](https://www.genoscope.cns.fr/agc/mage/wwwpkgdb/Info/getInfoLabel.php?id=3482699&wwwpkgdb=fc2733d6468c073f1dd738aa598ede55&nocache=a9a895ad49f0f55c362d050dc1ad3fa3&dir=&wwwpkgdb=fc2733d6468c073f1dd738aa598ede55) | _ | hypothetical protein |
| [OCAR_5072](https://www.genoscope.cns.fr/agc/mage/wwwpkgdb/Info/getInfoLabel.php?id=3482702&wwwpkgdb=fc2733d6468c073f1dd738aa598ede55&nocache=a9a895ad49f0f55c362d050dc1ad3fa3&dir=&wwwpkgdb=fc2733d6468c073f1dd738aa598ede55) | _ | hypothetical protein |
| [OCAR_5076](https://www.genoscope.cns.fr/agc/mage/wwwpkgdb/Info/getInfoLabel.php?id=3482705&wwwpkgdb=fc2733d6468c073f1dd738aa598ede55&nocache=a9a895ad49f0f55c362d050dc1ad3fa3&dir=&wwwpkgdb=fc2733d6468c073f1dd738aa598ede55) | _ | hypothetical protein |
| [OCAR_1078](https://www.genoscope.cns.fr/agc/mage/wwwpkgdb/Info/getInfoLabel.php?id=3482134&wwwpkgdb=fc2733d6468c073f1dd738aa598ede55&nocache=a9a895ad49f0f55c362d050dc1ad3fa3&dir=&wwwpkgdb=fc2733d6468c073f1dd738aa598ede55) | _ | _ |
| [OCAR_1080](https://www.genoscope.cns.fr/agc/mage/wwwpkgdb/Info/getInfoLabel.php?id=3482045&wwwpkgdb=fc2733d6468c073f1dd738aa598ede55&nocache=a9a895ad49f0f55c362d050dc1ad3fa3&dir=&wwwpkgdb=fc2733d6468c073f1dd738aa598ede55) | _ | _ |
| [OCAR_5091](https://www.genoscope.cns.fr/agc/mage/wwwpkgdb/Info/getInfoLabel.php?id=3482713&wwwpkgdb=fc2733d6468c073f1dd738aa598ede55&nocache=a9a895ad49f0f55c362d050dc1ad3fa3&dir=&wwwpkgdb=fc2733d6468c073f1dd738aa598ede55) | _ | transcriptional regulator/antitoxin, MazE |
| [OCAR_5092](https://www.genoscope.cns.fr/agc/mage/wwwpkgdb/Info/getInfoLabel.php?id=3482714&wwwpkgdb=fc2733d6468c073f1dd738aa598ede55&nocache=a9a895ad49f0f55c362d050dc1ad3fa3&dir=&wwwpkgdb=fc2733d6468c073f1dd738aa598ede55) | _ | death-on-curing protein |
| [OCAR_5097](https://www.genoscope.cns.fr/agc/mage/wwwpkgdb/Info/getInfoLabel.php?id=3482719&wwwpkgdb=fc2733d6468c073f1dd738aa598ede55&nocache=a9a895ad49f0f55c362d050dc1ad3fa3&dir=&wwwpkgdb=fc2733d6468c073f1dd738aa598ede55) | _ | hypothetical protein |
| [OCAR_5106](https://www.genoscope.cns.fr/agc/mage/wwwpkgdb/Info/getInfoLabel.php?id=3482723&wwwpkgdb=fc2733d6468c073f1dd738aa598ede55&nocache=a9a895ad49f0f55c362d050dc1ad3fa3&dir=&wwwpkgdb=fc2733d6468c073f1dd738aa598ede55) | _ | hypothetical protein |
| [OCAR_5107](https://www.genoscope.cns.fr/agc/mage/wwwpkgdb/Info/getInfoLabel.php?id=3484568&wwwpkgdb=fc2733d6468c073f1dd738aa598ede55&nocache=a9a895ad49f0f55c362d050dc1ad3fa3&dir=&wwwpkgdb=fc2733d6468c073f1dd738aa598ede55) | _ | hypothetical protein |
| [OCAR_5115](https://www.genoscope.cns.fr/agc/mage/wwwpkgdb/Info/getInfoLabel.php?id=3482729&wwwpkgdb=fc2733d6468c073f1dd738aa598ede55&nocache=a9a895ad49f0f55c362d050dc1ad3fa3&dir=&wwwpkgdb=fc2733d6468c073f1dd738aa598ede55) | _ | hypothetical protein |
| [OCAR_5118](https://www.genoscope.cns.fr/agc/mage/wwwpkgdb/Info/getInfoLabel.php?id=3484571&wwwpkgdb=fc2733d6468c073f1dd738aa598ede55&nocache=a9a895ad49f0f55c362d050dc1ad3fa3&dir=&wwwpkgdb=fc2733d6468c073f1dd738aa598ede55) | _ | ferric iron uptake ABC transporter (FeT) family, permease protein |
| [OCAR_5119](https://www.genoscope.cns.fr/agc/mage/wwwpkgdb/Info/getInfoLabel.php?id=3484572&wwwpkgdb=fc2733d6468c073f1dd738aa598ede55&nocache=a9a895ad49f0f55c362d050dc1ad3fa3&dir=&wwwpkgdb=fc2733d6468c073f1dd738aa598ede55) | _ | extracellular solute-binding protein, family 1 |
| [OCAR_5127](https://www.genoscope.cns.fr/agc/mage/wwwpkgdb/Info/getInfoLabel.php?id=3482736&wwwpkgdb=fc2733d6468c073f1dd738aa598ede55&nocache=a9a895ad49f0f55c362d050dc1ad3fa3&dir=&wwwpkgdb=fc2733d6468c073f1dd738aa598ede55) | _ | hypothetical protein |
| [OCAR_5129](https://www.genoscope.cns.fr/agc/mage/wwwpkgdb/Info/getInfoLabel.php?id=3484577&wwwpkgdb=fc2733d6468c073f1dd738aa598ede55&nocache=a9a895ad49f0f55c362d050dc1ad3fa3&dir=&wwwpkgdb=fc2733d6468c073f1dd738aa598ede55) | _ | hypothetical protein |
| [OCAR_5149](https://www.genoscope.cns.fr/agc/mage/wwwpkgdb/Info/getInfoLabel.php?id=3482744&wwwpkgdb=fc2733d6468c073f1dd738aa598ede55&nocache=a9a895ad49f0f55c362d050dc1ad3fa3&dir=&wwwpkgdb=fc2733d6468c073f1dd738aa598ede55) | _ | NADPH-dependent fmn reductase |
| [OCAR_5151](https://www.genoscope.cns.fr/agc/mage/wwwpkgdb/Info/getInfoLabel.php?id=3482745&wwwpkgdb=fc2733d6468c073f1dd738aa598ede55&nocache=a9a895ad49f0f55c362d050dc1ad3fa3&dir=&wwwpkgdb=fc2733d6468c073f1dd738aa598ede55) | _ | hypothetical protein |
| [OCAR_5152](https://www.genoscope.cns.fr/agc/mage/wwwpkgdb/Info/getInfoLabel.php?id=3482746&wwwpkgdb=fc2733d6468c073f1dd738aa598ede55&nocache=a9a895ad49f0f55c362d050dc1ad3fa3&dir=&wwwpkgdb=fc2733d6468c073f1dd738aa598ede55) | _ | hypothetical protein |
| [OCAR_5156](https://www.genoscope.cns.fr/agc/mage/wwwpkgdb/Info/getInfoLabel.php?id=3484592&wwwpkgdb=fc2733d6468c073f1dd738aa598ede55&nocache=a9a895ad49f0f55c362d050dc1ad3fa3&dir=&wwwpkgdb=fc2733d6468c073f1dd738aa598ede55) | _ | transglutaminase domain protein |
| [OCAR_1147](https://www.genoscope.cns.fr/agc/mage/wwwpkgdb/Info/getInfoLabel.php?id=3482133&wwwpkgdb=fc2733d6468c073f1dd738aa598ede55&nocache=a9a895ad49f0f55c362d050dc1ad3fa3&dir=&wwwpkgdb=fc2733d6468c073f1dd738aa598ede55) | _ | _ |
| [OCAR_1148](https://www.genoscope.cns.fr/agc/mage/wwwpkgdb/Info/getInfoLabel.php?id=3482044&wwwpkgdb=fc2733d6468c073f1dd738aa598ede55&nocache=a9a895ad49f0f55c362d050dc1ad3fa3&dir=&wwwpkgdb=fc2733d6468c073f1dd738aa598ede55) | _ | _ |
| [OCAR_5158](https://www.genoscope.cns.fr/agc/mage/wwwpkgdb/Info/getInfoLabel.php?id=3482749&wwwpkgdb=fc2733d6468c073f1dd738aa598ede55&nocache=a9a895ad49f0f55c362d050dc1ad3fa3&dir=&wwwpkgdb=fc2733d6468c073f1dd738aa598ede55) | _ | major subunit of formate dehydrogenase-O |
| [OCAR_5159](https://www.genoscope.cns.fr/agc/mage/wwwpkgdb/Info/getInfoLabel.php?id=3482750&wwwpkgdb=fc2733d6468c073f1dd738aa598ede55&nocache=a9a895ad49f0f55c362d050dc1ad3fa3&dir=&wwwpkgdb=fc2733d6468c073f1dd738aa598ede55) | _ | alpha subunit formate dehydrogenase |
| [OCAR_5162](https://www.genoscope.cns.fr/agc/mage/wwwpkgdb/Info/getInfoLabel.php?id=3482753&wwwpkgdb=fc2733d6468c073f1dd738aa598ede55&nocache=a9a895ad49f0f55c362d050dc1ad3fa3&dir=&wwwpkgdb=fc2733d6468c073f1dd738aa598ede55) | fdhE | formate dehydrogenase accessory protein FdhE |
| [OCAR_5163](https://www.genoscope.cns.fr/agc/mage/wwwpkgdb/Info/getInfoLabel.php?id=3482754&wwwpkgdb=fc2733d6468c073f1dd738aa598ede55&nocache=a9a895ad49f0f55c362d050dc1ad3fa3&dir=&wwwpkgdb=fc2733d6468c073f1dd738aa598ede55) | selA | L-seryl-tRNA selenium transferase |
| [OCAR_5165](https://www.genoscope.cns.fr/agc/mage/wwwpkgdb/Info/getInfoLabel.php?id=3482756&wwwpkgdb=fc2733d6468c073f1dd738aa598ede55&nocache=a9a895ad49f0f55c362d050dc1ad3fa3&dir=&wwwpkgdb=fc2733d6468c073f1dd738aa598ede55) | selB | selenocysteine-specific translation elongation factor |
| [OCAR_5166](https://www.genoscope.cns.fr/agc/mage/wwwpkgdb/Info/getInfoLabel.php?id=3482757&wwwpkgdb=fc2733d6468c073f1dd738aa598ede55&nocache=a9a895ad49f0f55c362d050dc1ad3fa3&dir=&wwwpkgdb=fc2733d6468c073f1dd738aa598ede55) | _ | hypothetical protein |
| [OCAR_5167](https://www.genoscope.cns.fr/agc/mage/wwwpkgdb/Info/getInfoLabel.php?id=3482758&wwwpkgdb=fc2733d6468c073f1dd738aa598ede55&nocache=a9a895ad49f0f55c362d050dc1ad3fa3&dir=&wwwpkgdb=fc2733d6468c073f1dd738aa598ede55) | _ | hypothetical protein |
| [OCAR_5168](https://www.genoscope.cns.fr/agc/mage/wwwpkgdb/Info/getInfoLabel.php?id=3484594&wwwpkgdb=fc2733d6468c073f1dd738aa598ede55&nocache=a9a895ad49f0f55c362d050dc1ad3fa3&dir=&wwwpkgdb=fc2733d6468c073f1dd738aa598ede55) | selD | selenide, water dikinase |
| [OCAR_1161](https://www.genoscope.cns.fr/agc/mage/wwwpkgdb/Info/getInfoLabel.php?id=3482043&wwwpkgdb=fc2733d6468c073f1dd738aa598ede55&nocache=a9a895ad49f0f55c362d050dc1ad3fa3&dir=&wwwpkgdb=fc2733d6468c073f1dd738aa598ede55) | _ | _ |
| [OCAR_5178](https://www.genoscope.cns.fr/agc/mage/wwwpkgdb/Info/getInfoLabel.php?id=3484599&wwwpkgdb=fc2733d6468c073f1dd738aa598ede55&nocache=a9a895ad49f0f55c362d050dc1ad3fa3&dir=&wwwpkgdb=fc2733d6468c073f1dd738aa598ede55) | _ | beta lactamase |
| [OCAR_5193](https://www.genoscope.cns.fr/agc/mage/wwwpkgdb/Info/getInfoLabel.php?id=3484602&wwwpkgdb=fc2733d6468c073f1dd738aa598ede55&nocache=a9a895ad49f0f55c362d050dc1ad3fa3&dir=&wwwpkgdb=fc2733d6468c073f1dd738aa598ede55) | _ | universal stress protein |
| [OCAR_1192](https://www.genoscope.cns.fr/agc/mage/wwwpkgdb/Info/getInfoLabel.php?id=3482041&wwwpkgdb=fc2733d6468c073f1dd738aa598ede55&nocache=a9a895ad49f0f55c362d050dc1ad3fa3&dir=&wwwpkgdb=fc2733d6468c073f1dd738aa598ede55) | _ | _ |
| [OCAR_1194](https://www.genoscope.cns.fr/agc/mage/wwwpkgdb/Info/getInfoLabel.php?id=3482040&wwwpkgdb=fc2733d6468c073f1dd738aa598ede55&nocache=a9a895ad49f0f55c362d050dc1ad3fa3&dir=&wwwpkgdb=fc2733d6468c073f1dd738aa598ede55) | _ | _ |
| [OCAR_5200](https://www.genoscope.cns.fr/agc/mage/wwwpkgdb/Info/getInfoLabel.php?id=3482776&wwwpkgdb=fc2733d6468c073f1dd738aa598ede55&nocache=a9a895ad49f0f55c362d050dc1ad3fa3&dir=&wwwpkgdb=fc2733d6468c073f1dd738aa598ede55) | _ | hypothetical protein |
| [OCAR_5204](https://www.genoscope.cns.fr/agc/mage/wwwpkgdb/Info/getInfoLabel.php?id=3484609&wwwpkgdb=fc2733d6468c073f1dd738aa598ede55&nocache=a9a895ad49f0f55c362d050dc1ad3fa3&dir=&wwwpkgdb=fc2733d6468c073f1dd738aa598ede55) | _ | 3-isopropylmalate dehydrogenase |
| [OCAR_5211](https://www.genoscope.cns.fr/agc/mage/wwwpkgdb/Info/getInfoLabel.php?id=3484613&wwwpkgdb=fc2733d6468c073f1dd738aa598ede55&nocache=a9a895ad49f0f55c362d050dc1ad3fa3&dir=&wwwpkgdb=fc2733d6468c073f1dd738aa598ede55) | _ | hypothetical protein |
| [OCAR_5218](https://www.genoscope.cns.fr/agc/mage/wwwpkgdb/Info/getInfoLabel.php?id=3482789&wwwpkgdb=fc2733d6468c073f1dd738aa598ede55&nocache=a9a895ad49f0f55c362d050dc1ad3fa3&dir=&wwwpkgdb=fc2733d6468c073f1dd738aa598ede55) | _ | aminotransferase, class IV |
| [OCAR_1213](https://www.genoscope.cns.fr/agc/mage/wwwpkgdb/Info/getInfoLabel.php?id=3482039&wwwpkgdb=fc2733d6468c073f1dd738aa598ede55&nocache=a9a895ad49f0f55c362d050dc1ad3fa3&dir=&wwwpkgdb=fc2733d6468c073f1dd738aa598ede55) | _ | _ |
| [OCAR_5228](https://www.genoscope.cns.fr/agc/mage/wwwpkgdb/Info/getInfoLabel.php?id=3484617&wwwpkgdb=fc2733d6468c073f1dd738aa598ede55&nocache=a9a895ad49f0f55c362d050dc1ad3fa3&dir=&wwwpkgdb=fc2733d6468c073f1dd738aa598ede55) | _ | hypothetical protein |
| [OCAR_1225](https://www.genoscope.cns.fr/agc/mage/wwwpkgdb/Info/getInfoLabel.php?id=3482038&wwwpkgdb=fc2733d6468c073f1dd738aa598ede55&nocache=a9a895ad49f0f55c362d050dc1ad3fa3&dir=&wwwpkgdb=fc2733d6468c073f1dd738aa598ede55) | _ | _ |
| [OCAR_5233](https://www.genoscope.cns.fr/agc/mage/wwwpkgdb/Info/getInfoLabel.php?id=3484620&wwwpkgdb=fc2733d6468c073f1dd738aa598ede55&nocache=a9a895ad49f0f55c362d050dc1ad3fa3&dir=&wwwpkgdb=fc2733d6468c073f1dd738aa598ede55) | _ | putative tmprss13 protein |
| [OCAR_5246](https://www.genoscope.cns.fr/agc/mage/wwwpkgdb/Info/getInfoLabel.php?id=3484622&wwwpkgdb=fc2733d6468c073f1dd738aa598ede55&nocache=a9a895ad49f0f55c362d050dc1ad3fa3&dir=&wwwpkgdb=fc2733d6468c073f1dd738aa598ede55) | _ | hypothetical protein |
| [OCAR_5255](https://www.genoscope.cns.fr/agc/mage/wwwpkgdb/Info/getInfoLabel.php?id=3482817&wwwpkgdb=fc2733d6468c073f1dd738aa598ede55&nocache=a9a895ad49f0f55c362d050dc1ad3fa3&dir=&wwwpkgdb=fc2733d6468c073f1dd738aa598ede55) | _ | hypothetical protein |
| [OCAR_5258](https://www.genoscope.cns.fr/agc/mage/wwwpkgdb/Info/getInfoLabel.php?id=3482818&wwwpkgdb=fc2733d6468c073f1dd738aa598ede55&nocache=a9a895ad49f0f55c362d050dc1ad3fa3&dir=&wwwpkgdb=fc2733d6468c073f1dd738aa598ede55) | _ | hypothetical protein |
| [OCAR_5267](https://www.genoscope.cns.fr/agc/mage/wwwpkgdb/Info/getInfoLabel.php?id=3484630&wwwpkgdb=fc2733d6468c073f1dd738aa598ede55&nocache=a9a895ad49f0f55c362d050dc1ad3fa3&dir=&wwwpkgdb=fc2733d6468c073f1dd738aa598ede55) | _ | hypothetical protein |
| [OCAR_5268](https://www.genoscope.cns.fr/agc/mage/wwwpkgdb/Info/getInfoLabel.php?id=3484631&wwwpkgdb=fc2733d6468c073f1dd738aa598ede55&nocache=a9a895ad49f0f55c362d050dc1ad3fa3&dir=&wwwpkgdb=fc2733d6468c073f1dd738aa598ede55) | _ | hypothetical protein |
| [OCAR_1262](https://www.genoscope.cns.fr/agc/mage/wwwpkgdb/Info/getInfoLabel.php?id=3482037&wwwpkgdb=fc2733d6468c073f1dd738aa598ede55&nocache=a9a895ad49f0f55c362d050dc1ad3fa3&dir=&wwwpkgdb=fc2733d6468c073f1dd738aa598ede55) | _ | _ |
| [OCAR_5273](https://www.genoscope.cns.fr/agc/mage/wwwpkgdb/Info/getInfoLabel.php?id=3482824&wwwpkgdb=fc2733d6468c073f1dd738aa598ede55&nocache=a9a895ad49f0f55c362d050dc1ad3fa3&dir=&wwwpkgdb=fc2733d6468c073f1dd738aa598ede55) | _ | hypothetical protein |
| [OCAR_1267](https://www.genoscope.cns.fr/agc/mage/wwwpkgdb/Info/getInfoLabel.php?id=3482035&wwwpkgdb=fc2733d6468c073f1dd738aa598ede55&nocache=a9a895ad49f0f55c362d050dc1ad3fa3&dir=&wwwpkgdb=fc2733d6468c073f1dd738aa598ede55) | _ | _ |
| [OCAR_5284](https://www.genoscope.cns.fr/agc/mage/wwwpkgdb/Info/getInfoLabel.php?id=3484637&wwwpkgdb=fc2733d6468c073f1dd738aa598ede55&nocache=a9a895ad49f0f55c362d050dc1ad3fa3&dir=&wwwpkgdb=fc2733d6468c073f1dd738aa598ede55) | _ | hypothetical protein |
| [OCAR_5285](https://www.genoscope.cns.fr/agc/mage/wwwpkgdb/Info/getInfoLabel.php?id=3482832&wwwpkgdb=fc2733d6468c073f1dd738aa598ede55&nocache=a9a895ad49f0f55c362d050dc1ad3fa3&dir=&wwwpkgdb=fc2733d6468c073f1dd738aa598ede55) | selB | selenocysteine-specific translation elongation factor |
| [OCAR_1281](https://www.genoscope.cns.fr/agc/mage/wwwpkgdb/Info/getInfoLabel.php?id=3482034&wwwpkgdb=fc2733d6468c073f1dd738aa598ede55&nocache=a9a895ad49f0f55c362d050dc1ad3fa3&dir=&wwwpkgdb=fc2733d6468c073f1dd738aa598ede55) | _ | _ |
| [OCAR_1282](https://www.genoscope.cns.fr/agc/mage/wwwpkgdb/Info/getInfoLabel.php?id=3482033&wwwpkgdb=fc2733d6468c073f1dd738aa598ede55&nocache=a9a895ad49f0f55c362d050dc1ad3fa3&dir=&wwwpkgdb=fc2733d6468c073f1dd738aa598ede55) | _ | _ |
| [OCAR_5293](https://www.genoscope.cns.fr/agc/mage/wwwpkgdb/Info/getInfoLabel.php?id=3482836&wwwpkgdb=fc2733d6468c073f1dd738aa598ede55&nocache=a9a895ad49f0f55c362d050dc1ad3fa3&dir=&wwwpkgdb=fc2733d6468c073f1dd738aa598ede55) | _ | hypothetical protein |
| [OCAR_5295](https://www.genoscope.cns.fr/agc/mage/wwwpkgdb/Info/getInfoLabel.php?id=3484642&wwwpkgdb=fc2733d6468c073f1dd738aa598ede55&nocache=a9a895ad49f0f55c362d050dc1ad3fa3&dir=&wwwpkgdb=fc2733d6468c073f1dd738aa598ede55) | _ | hypothetical protein |
| [OCAR_5300](https://www.genoscope.cns.fr/agc/mage/wwwpkgdb/Info/getInfoLabel.php?id=3482842&wwwpkgdb=fc2733d6468c073f1dd738aa598ede55&nocache=a9a895ad49f0f55c362d050dc1ad3fa3&dir=&wwwpkgdb=fc2733d6468c073f1dd738aa598ede55) | _ | hypothetical protein |
| [OCAR_5302](https://www.genoscope.cns.fr/agc/mage/wwwpkgdb/Info/getInfoLabel.php?id=3482844&wwwpkgdb=fc2733d6468c073f1dd738aa598ede55&nocache=a9a895ad49f0f55c362d050dc1ad3fa3&dir=&wwwpkgdb=fc2733d6468c073f1dd738aa598ede55) | _ | FAD linked oxidase, C-:FAD linked oxidase |
| [OCAR_5306](https://www.genoscope.cns.fr/agc/mage/wwwpkgdb/Info/getInfoLabel.php?id=3482848&wwwpkgdb=fc2733d6468c073f1dd738aa598ede55&nocache=a9a895ad49f0f55c362d050dc1ad3fa3&dir=&wwwpkgdb=fc2733d6468c073f1dd738aa598ede55) | _ | AMP-dependent synthetase and ligase |
| [OCAR_5307](https://www.genoscope.cns.fr/agc/mage/wwwpkgdb/Info/getInfoLabel.php?id=3482849&wwwpkgdb=fc2733d6468c073f1dd738aa598ede55&nocache=a9a895ad49f0f55c362d050dc1ad3fa3&dir=&wwwpkgdb=fc2733d6468c073f1dd738aa598ede55) | _ | hypothetical protein |
| [OCAR_5308](https://www.genoscope.cns.fr/agc/mage/wwwpkgdb/Info/getInfoLabel.php?id=3482850&wwwpkgdb=fc2733d6468c073f1dd738aa598ede55&nocache=a9a895ad49f0f55c362d050dc1ad3fa3&dir=&wwwpkgdb=fc2733d6468c073f1dd738aa598ede55) | _ | putative 2-oxo acid dehydrogenase acyltransferase catalytic domain protein |
| [OCAR_1301](https://www.genoscope.cns.fr/agc/mage/wwwpkgdb/Info/getInfoLabel.php?id=3482032&wwwpkgdb=fc2733d6468c073f1dd738aa598ede55&nocache=a9a895ad49f0f55c362d050dc1ad3fa3&dir=&wwwpkgdb=fc2733d6468c073f1dd738aa598ede55) | _ | _ |
| [OCAR_5314](https://www.genoscope.cns.fr/agc/mage/wwwpkgdb/Info/getInfoLabel.php?id=3484645&wwwpkgdb=fc2733d6468c073f1dd738aa598ede55&nocache=a9a895ad49f0f55c362d050dc1ad3fa3&dir=&wwwpkgdb=fc2733d6468c073f1dd738aa598ede55) | _ | hypothetical protein |
| [OCAR_5320](https://www.genoscope.cns.fr/agc/mage/wwwpkgdb/Info/getInfoLabel.php?id=3484646&wwwpkgdb=fc2733d6468c073f1dd738aa598ede55&nocache=a9a895ad49f0f55c362d050dc1ad3fa3&dir=&wwwpkgdb=fc2733d6468c073f1dd738aa598ede55) | _ | hypothetical protein |
| [OCAR_1313](https://www.genoscope.cns.fr/agc/mage/wwwpkgdb/Info/getInfoLabel.php?id=3482031&wwwpkgdb=fc2733d6468c073f1dd738aa598ede55&nocache=a9a895ad49f0f55c362d050dc1ad3fa3&dir=&wwwpkgdb=fc2733d6468c073f1dd738aa598ede55) | _ | _ |
| [OCAR_1315](https://www.genoscope.cns.fr/agc/mage/wwwpkgdb/Info/getInfoLabel.php?id=3482030&wwwpkgdb=fc2733d6468c073f1dd738aa598ede55&nocache=a9a895ad49f0f55c362d050dc1ad3fa3&dir=&wwwpkgdb=fc2733d6468c073f1dd738aa598ede55) | _ | _ |
| [OCAR_5323](https://www.genoscope.cns.fr/agc/mage/wwwpkgdb/Info/getInfoLabel.php?id=3482861&wwwpkgdb=fc2733d6468c073f1dd738aa598ede55&nocache=a9a895ad49f0f55c362d050dc1ad3fa3&dir=&wwwpkgdb=fc2733d6468c073f1dd738aa598ede55) | _ | hypothetical protein |
| [OCAR_5324](https://www.genoscope.cns.fr/agc/mage/wwwpkgdb/Info/getInfoLabel.php?id=3482862&wwwpkgdb=fc2733d6468c073f1dd738aa598ede55&nocache=a9a895ad49f0f55c362d050dc1ad3fa3&dir=&wwwpkgdb=fc2733d6468c073f1dd738aa598ede55) | _ | integral membrane protein |
| [OCAR_5326](https://www.genoscope.cns.fr/agc/mage/wwwpkgdb/Info/getInfoLabel.php?id=3482864&wwwpkgdb=fc2733d6468c073f1dd738aa598ede55&nocache=a9a895ad49f0f55c362d050dc1ad3fa3&dir=&wwwpkgdb=fc2733d6468c073f1dd738aa598ede55) | _ | hypothetical protein |
| [OCAR_1319](https://www.genoscope.cns.fr/agc/mage/wwwpkgdb/Info/getInfoLabel.php?id=3482029&wwwpkgdb=fc2733d6468c073f1dd738aa598ede55&nocache=a9a895ad49f0f55c362d050dc1ad3fa3&dir=&wwwpkgdb=fc2733d6468c073f1dd738aa598ede55) | _ | _ |
| [OCAR_1333](https://www.genoscope.cns.fr/agc/mage/wwwpkgdb/Info/getInfoLabel.php?id=3482027&wwwpkgdb=fc2733d6468c073f1dd738aa598ede55&nocache=a9a895ad49f0f55c362d050dc1ad3fa3&dir=&wwwpkgdb=fc2733d6468c073f1dd738aa598ede55) | _ | _ |
| [OCAR_5349](https://www.genoscope.cns.fr/agc/mage/wwwpkgdb/Info/getInfoLabel.php?id=3482878&wwwpkgdb=fc2733d6468c073f1dd738aa598ede55&nocache=a9a895ad49f0f55c362d050dc1ad3fa3&dir=&wwwpkgdb=fc2733d6468c073f1dd738aa598ede55) | _ | hypothetical protein |
| [OCAR_1352](https://www.genoscope.cns.fr/agc/mage/wwwpkgdb/Info/getInfoLabel.php?id=3482026&wwwpkgdb=fc2733d6468c073f1dd738aa598ede55&nocache=a9a895ad49f0f55c362d050dc1ad3fa3&dir=&wwwpkgdb=fc2733d6468c073f1dd738aa598ede55) | _ | _ |
| [OCAR_5361](https://www.genoscope.cns.fr/agc/mage/wwwpkgdb/Info/getInfoLabel.php?id=3482886&wwwpkgdb=fc2733d6468c073f1dd738aa598ede55&nocache=a9a895ad49f0f55c362d050dc1ad3fa3&dir=&wwwpkgdb=fc2733d6468c073f1dd738aa598ede55) | _ | hypothetical protein |
| [OCAR_5374](https://www.genoscope.cns.fr/agc/mage/wwwpkgdb/Info/getInfoLabel.php?id=3482894&wwwpkgdb=fc2733d6468c073f1dd738aa598ede55&nocache=a9a895ad49f0f55c362d050dc1ad3fa3&dir=&wwwpkgdb=fc2733d6468c073f1dd738aa598ede55) | _ | TPR domain protein |
| [OCAR_5383](https://www.genoscope.cns.fr/agc/mage/wwwpkgdb/Info/getInfoLabel.php?id=3484669&wwwpkgdb=fc2733d6468c073f1dd738aa598ede55&nocache=a9a895ad49f0f55c362d050dc1ad3fa3&dir=&wwwpkgdb=fc2733d6468c073f1dd738aa598ede55) | _ | hypothetical protein |
| [OCAR_5405](https://www.genoscope.cns.fr/agc/mage/wwwpkgdb/Info/getInfoLabel.php?id=3482914&wwwpkgdb=fc2733d6468c073f1dd738aa598ede55&nocache=a9a895ad49f0f55c362d050dc1ad3fa3&dir=&wwwpkgdb=fc2733d6468c073f1dd738aa598ede55) | _ | hypothetical protein |
| [OCAR_5412](https://www.genoscope.cns.fr/agc/mage/wwwpkgdb/Info/getInfoLabel.php?id=3482919&wwwpkgdb=fc2733d6468c073f1dd738aa598ede55&nocache=a9a895ad49f0f55c362d050dc1ad3fa3&dir=&wwwpkgdb=fc2733d6468c073f1dd738aa598ede55) | _ | activator of Hsp90 ATPase 1 family protein |
| [OCAR_5413](https://www.genoscope.cns.fr/agc/mage/wwwpkgdb/Info/getInfoLabel.php?id=3482920&wwwpkgdb=fc2733d6468c073f1dd738aa598ede55&nocache=a9a895ad49f0f55c362d050dc1ad3fa3&dir=&wwwpkgdb=fc2733d6468c073f1dd738aa598ede55) | _ | hypothetical protein |
| [OCAR_1414](https://www.genoscope.cns.fr/agc/mage/wwwpkgdb/Info/getInfoLabel.php?id=3482025&wwwpkgdb=fc2733d6468c073f1dd738aa598ede55&nocache=a9a895ad49f0f55c362d050dc1ad3fa3&dir=&wwwpkgdb=fc2733d6468c073f1dd738aa598ede55) | _ | _ |
| [OCAR_5422](https://www.genoscope.cns.fr/agc/mage/wwwpkgdb/Info/getInfoLabel.php?id=3484680&wwwpkgdb=fc2733d6468c073f1dd738aa598ede55&nocache=a9a895ad49f0f55c362d050dc1ad3fa3&dir=&wwwpkgdb=fc2733d6468c073f1dd738aa598ede55) | _ | hypothetical protein |
| [OCAR_5423](https://www.genoscope.cns.fr/agc/mage/wwwpkgdb/Info/getInfoLabel.php?id=3484681&wwwpkgdb=fc2733d6468c073f1dd738aa598ede55&nocache=a9a895ad49f0f55c362d050dc1ad3fa3&dir=&wwwpkgdb=fc2733d6468c073f1dd738aa598ede55) | _ | hypothetical protein |
| [OCAR_5424](https://www.genoscope.cns.fr/agc/mage/wwwpkgdb/Info/getInfoLabel.php?id=3484682&wwwpkgdb=fc2733d6468c073f1dd738aa598ede55&nocache=a9a895ad49f0f55c362d050dc1ad3fa3&dir=&wwwpkgdb=fc2733d6468c073f1dd738aa598ede55) | _ | hypothetical protein |
| [OCAR_1420](https://www.genoscope.cns.fr/agc/mage/wwwpkgdb/Info/getInfoLabel.php?id=3482024&wwwpkgdb=fc2733d6468c073f1dd738aa598ede55&nocache=a9a895ad49f0f55c362d050dc1ad3fa3&dir=&wwwpkgdb=fc2733d6468c073f1dd738aa598ede55) | _ | _ |
| [OCAR_1423](https://www.genoscope.cns.fr/agc/mage/wwwpkgdb/Info/getInfoLabel.php?id=3482023&wwwpkgdb=fc2733d6468c073f1dd738aa598ede55&nocache=a9a895ad49f0f55c362d050dc1ad3fa3&dir=&wwwpkgdb=fc2733d6468c073f1dd738aa598ede55) | _ | _ |
| [OCAR_5429](https://www.genoscope.cns.fr/agc/mage/wwwpkgdb/Info/getInfoLabel.php?id=3484684&wwwpkgdb=fc2733d6468c073f1dd738aa598ede55&nocache=a9a895ad49f0f55c362d050dc1ad3fa3&dir=&wwwpkgdb=fc2733d6468c073f1dd738aa598ede55) | _ | hypothetical protein |
| [OCAR_5430](https://www.genoscope.cns.fr/agc/mage/wwwpkgdb/Info/getInfoLabel.php?id=3484685&wwwpkgdb=fc2733d6468c073f1dd738aa598ede55&nocache=a9a895ad49f0f55c362d050dc1ad3fa3&dir=&wwwpkgdb=fc2733d6468c073f1dd738aa598ede55) | _ | hypothetical protein |
| [OCAR_5438](https://www.genoscope.cns.fr/agc/mage/wwwpkgdb/Info/getInfoLabel.php?id=3482932&wwwpkgdb=fc2733d6468c073f1dd738aa598ede55&nocache=a9a895ad49f0f55c362d050dc1ad3fa3&dir=&wwwpkgdb=fc2733d6468c073f1dd738aa598ede55) | _ | inner membrane protein YbaL |
| [OCAR_5437](https://www.genoscope.cns.fr/agc/mage/wwwpkgdb/Info/getInfoLabel.php?id=3484690&wwwpkgdb=fc2733d6468c073f1dd738aa598ede55&nocache=a9a895ad49f0f55c362d050dc1ad3fa3&dir=&wwwpkgdb=fc2733d6468c073f1dd738aa598ede55) | _ | hypothetical protein |
| [OCAR_5439](https://www.genoscope.cns.fr/agc/mage/wwwpkgdb/Info/getInfoLabel.php?id=3482933&wwwpkgdb=fc2733d6468c073f1dd738aa598ede55&nocache=a9a895ad49f0f55c362d050dc1ad3fa3&dir=&wwwpkgdb=fc2733d6468c073f1dd738aa598ede55) | _ | hypothetical protein |
| [OCAR_5442](https://www.genoscope.cns.fr/agc/mage/wwwpkgdb/Info/getInfoLabel.php?id=3482934&wwwpkgdb=fc2733d6468c073f1dd738aa598ede55&nocache=a9a895ad49f0f55c362d050dc1ad3fa3&dir=&wwwpkgdb=fc2733d6468c073f1dd738aa598ede55) | _ | hypothetical protein |
| [OCAR_5441](https://www.genoscope.cns.fr/agc/mage/wwwpkgdb/Info/getInfoLabel.php?id=3484692&wwwpkgdb=fc2733d6468c073f1dd738aa598ede55&nocache=a9a895ad49f0f55c362d050dc1ad3fa3&dir=&wwwpkgdb=fc2733d6468c073f1dd738aa598ede55) | _ | hypothetical protein |
| [OCAR_5445](https://www.genoscope.cns.fr/agc/mage/wwwpkgdb/Info/getInfoLabel.php?id=3482936&wwwpkgdb=fc2733d6468c073f1dd738aa598ede55&nocache=a9a895ad49f0f55c362d050dc1ad3fa3&dir=&wwwpkgdb=fc2733d6468c073f1dd738aa598ede55) | _ | hypothetical protein |
| [OCAR_5448](https://www.genoscope.cns.fr/agc/mage/wwwpkgdb/Info/getInfoLabel.php?id=3484696&wwwpkgdb=fc2733d6468c073f1dd738aa598ede55&nocache=a9a895ad49f0f55c362d050dc1ad3fa3&dir=&wwwpkgdb=fc2733d6468c073f1dd738aa598ede55) | _ | hypothetical protein |
| [OCAR_5461](https://www.genoscope.cns.fr/agc/mage/wwwpkgdb/Info/getInfoLabel.php?id=3484702&wwwpkgdb=fc2733d6468c073f1dd738aa598ede55&nocache=a9a895ad49f0f55c362d050dc1ad3fa3&dir=&wwwpkgdb=fc2733d6468c073f1dd738aa598ede55) | _ | hydrolase, Alpha/Beta family |
| [OCAR_1454](https://www.genoscope.cns.fr/agc/mage/wwwpkgdb/Info/getInfoLabel.php?id=3482022&wwwpkgdb=fc2733d6468c073f1dd738aa598ede55&nocache=a9a895ad49f0f55c362d050dc1ad3fa3&dir=&wwwpkgdb=fc2733d6468c073f1dd738aa598ede55) | _ | _ |
| [OCAR_5476](https://www.genoscope.cns.fr/agc/mage/wwwpkgdb/Info/getInfoLabel.php?id=3484714&wwwpkgdb=fc2733d6468c073f1dd738aa598ede55&nocache=a9a895ad49f0f55c362d050dc1ad3fa3&dir=&wwwpkgdb=fc2733d6468c073f1dd738aa598ede55) | _ | hypothetical protein |
| [OCAR_1472](https://www.genoscope.cns.fr/agc/mage/wwwpkgdb/Info/getInfoLabel.php?id=3482021&wwwpkgdb=fc2733d6468c073f1dd738aa598ede55&nocache=a9a895ad49f0f55c362d050dc1ad3fa3&dir=&wwwpkgdb=fc2733d6468c073f1dd738aa598ede55) | _ | _ |
| [OCAR_5481](https://www.genoscope.cns.fr/agc/mage/wwwpkgdb/Info/getInfoLabel.php?id=3482947&wwwpkgdb=fc2733d6468c073f1dd738aa598ede55&nocache=a9a895ad49f0f55c362d050dc1ad3fa3&dir=&wwwpkgdb=fc2733d6468c073f1dd738aa598ede55) | _ | hypothetical protein |
| [OCAR_1482](https://www.genoscope.cns.fr/agc/mage/wwwpkgdb/Info/getInfoLabel.php?id=3482020&wwwpkgdb=fc2733d6468c073f1dd738aa598ede55&nocache=a9a895ad49f0f55c362d050dc1ad3fa3&dir=&wwwpkgdb=fc2733d6468c073f1dd738aa598ede55) | _ | _ |
| [OCAR_5495](https://www.genoscope.cns.fr/agc/mage/wwwpkgdb/Info/getInfoLabel.php?id=3484725&wwwpkgdb=fc2733d6468c073f1dd738aa598ede55&nocache=a9a895ad49f0f55c362d050dc1ad3fa3&dir=&wwwpkgdb=fc2733d6468c073f1dd738aa598ede55) | _ | exopolysaccharide production protein ExoZ |
| [OCAR_1488](https://www.genoscope.cns.fr/agc/mage/wwwpkgdb/Info/getInfoLabel.php?id=3482019&wwwpkgdb=fc2733d6468c073f1dd738aa598ede55&nocache=a9a895ad49f0f55c362d050dc1ad3fa3&dir=&wwwpkgdb=fc2733d6468c073f1dd738aa598ede55) | _ | _ |
| [OCAR_5496](https://www.genoscope.cns.fr/agc/mage/wwwpkgdb/Info/getInfoLabel.php?id=3482955&wwwpkgdb=fc2733d6468c073f1dd738aa598ede55&nocache=a9a895ad49f0f55c362d050dc1ad3fa3&dir=&wwwpkgdb=fc2733d6468c073f1dd738aa598ede55) | _ | extracellular solute-binding protein, family 3 |
| [OCAR_1490](https://www.genoscope.cns.fr/agc/mage/wwwpkgdb/Info/getInfoLabel.php?id=3482017&wwwpkgdb=fc2733d6468c073f1dd738aa598ede55&nocache=a9a895ad49f0f55c362d050dc1ad3fa3&dir=&wwwpkgdb=fc2733d6468c073f1dd738aa598ede55) | _ | _ |
| [OCAR_5498](https://www.genoscope.cns.fr/agc/mage/wwwpkgdb/Info/getInfoLabel.php?id=3482957&wwwpkgdb=fc2733d6468c073f1dd738aa598ede55&nocache=a9a895ad49f0f55c362d050dc1ad3fa3&dir=&wwwpkgdb=fc2733d6468c073f1dd738aa598ede55) | _ | hypothetical protein |
| [OCAR_5499](https://www.genoscope.cns.fr/agc/mage/wwwpkgdb/Info/getInfoLabel.php?id=3482958&wwwpkgdb=fc2733d6468c073f1dd738aa598ede55&nocache=a9a895ad49f0f55c362d050dc1ad3fa3&dir=&wwwpkgdb=fc2733d6468c073f1dd738aa598ede55) | _ | putative lipoprotein |
| [OCAR_5500](https://www.genoscope.cns.fr/agc/mage/wwwpkgdb/Info/getInfoLabel.php?id=3482959&wwwpkgdb=fc2733d6468c073f1dd738aa598ede55&nocache=a9a895ad49f0f55c362d050dc1ad3fa3&dir=&wwwpkgdb=fc2733d6468c073f1dd738aa598ede55) | _ | hypothetical protein |
| [OCAR_5501](https://www.genoscope.cns.fr/agc/mage/wwwpkgdb/Info/getInfoLabel.php?id=3482960&wwwpkgdb=fc2733d6468c073f1dd738aa598ede55&nocache=a9a895ad49f0f55c362d050dc1ad3fa3&dir=&wwwpkgdb=fc2733d6468c073f1dd738aa598ede55) | _ | hypothetical protein |
| [OCAR_1501](https://www.genoscope.cns.fr/agc/mage/wwwpkgdb/Info/getInfoLabel.php?id=3482016&wwwpkgdb=fc2733d6468c073f1dd738aa598ede55&nocache=a9a895ad49f0f55c362d050dc1ad3fa3&dir=&wwwpkgdb=fc2733d6468c073f1dd738aa598ede55) | _ | _ |
| [OCAR_5511](https://www.genoscope.cns.fr/agc/mage/wwwpkgdb/Info/getInfoLabel.php?id=3482966&wwwpkgdb=fc2733d6468c073f1dd738aa598ede55&nocache=a9a895ad49f0f55c362d050dc1ad3fa3&dir=&wwwpkgdb=fc2733d6468c073f1dd738aa598ede55) | _ | glycosyl transferase |
| [OCAR_5523](https://www.genoscope.cns.fr/agc/mage/wwwpkgdb/Info/getInfoLabel.php?id=3484733&wwwpkgdb=fc2733d6468c073f1dd738aa598ede55&nocache=a9a895ad49f0f55c362d050dc1ad3fa3&dir=&wwwpkgdb=fc2733d6468c073f1dd738aa598ede55) | _ | glycosyl transferase, family 9 |
| [OCAR_5525](https://www.genoscope.cns.fr/agc/mage/wwwpkgdb/Info/getInfoLabel.php?id=3482975&wwwpkgdb=fc2733d6468c073f1dd738aa598ede55&nocache=a9a895ad49f0f55c362d050dc1ad3fa3&dir=&wwwpkgdb=fc2733d6468c073f1dd738aa598ede55) | _ | hypothetical protein |
| [OCAR_5528](https://www.genoscope.cns.fr/agc/mage/wwwpkgdb/Info/getInfoLabel.php?id=3484735&wwwpkgdb=fc2733d6468c073f1dd738aa598ede55&nocache=a9a895ad49f0f55c362d050dc1ad3fa3&dir=&wwwpkgdb=fc2733d6468c073f1dd738aa598ede55) | _ | hypothetical protein |
| [OCAR_5530](https://www.genoscope.cns.fr/agc/mage/wwwpkgdb/Info/getInfoLabel.php?id=3484737&wwwpkgdb=fc2733d6468c073f1dd738aa598ede55&nocache=a9a895ad49f0f55c362d050dc1ad3fa3&dir=&wwwpkgdb=fc2733d6468c073f1dd738aa598ede55) | _ | phage integrase |
| [OCAR_1526](https://www.genoscope.cns.fr/agc/mage/wwwpkgdb/Info/getInfoLabel.php?id=3482015&wwwpkgdb=fc2733d6468c073f1dd738aa598ede55&nocache=a9a895ad49f0f55c362d050dc1ad3fa3&dir=&wwwpkgdb=fc2733d6468c073f1dd738aa598ede55) | _ | _ |
| [OCAR_5531](https://www.genoscope.cns.fr/agc/mage/wwwpkgdb/Info/getInfoLabel.php?id=3484738&wwwpkgdb=fc2733d6468c073f1dd738aa598ede55&nocache=a9a895ad49f0f55c362d050dc1ad3fa3&dir=&wwwpkgdb=fc2733d6468c073f1dd738aa598ede55) | _ | hypothetical protein |
| [OCAR_1527](https://www.genoscope.cns.fr/agc/mage/wwwpkgdb/Info/getInfoLabel.php?id=3482014&wwwpkgdb=fc2733d6468c073f1dd738aa598ede55&nocache=a9a895ad49f0f55c362d050dc1ad3fa3&dir=&wwwpkgdb=fc2733d6468c073f1dd738aa598ede55) | _ | _ |
| [OCAR_5532](https://www.genoscope.cns.fr/agc/mage/wwwpkgdb/Info/getInfoLabel.php?id=3484739&wwwpkgdb=fc2733d6468c073f1dd738aa598ede55&nocache=a9a895ad49f0f55c362d050dc1ad3fa3&dir=&wwwpkgdb=fc2733d6468c073f1dd738aa598ede55) | _ | hypothetical protein |
| [OCAR_5533](https://www.genoscope.cns.fr/agc/mage/wwwpkgdb/Info/getInfoLabel.php?id=3484740&wwwpkgdb=fc2733d6468c073f1dd738aa598ede55&nocache=a9a895ad49f0f55c362d050dc1ad3fa3&dir=&wwwpkgdb=fc2733d6468c073f1dd738aa598ede55) | _ | hypothetical protein |
| [OCAR_5534](https://www.genoscope.cns.fr/agc/mage/wwwpkgdb/Info/getInfoLabel.php?id=3484741&wwwpkgdb=fc2733d6468c073f1dd738aa598ede55&nocache=a9a895ad49f0f55c362d050dc1ad3fa3&dir=&wwwpkgdb=fc2733d6468c073f1dd738aa598ede55) | _ | hypothetical protein |
| [OCAR_5535](https://www.genoscope.cns.fr/agc/mage/wwwpkgdb/Info/getInfoLabel.php?id=3484742&wwwpkgdb=fc2733d6468c073f1dd738aa598ede55&nocache=a9a895ad49f0f55c362d050dc1ad3fa3&dir=&wwwpkgdb=fc2733d6468c073f1dd738aa598ede55) | _ | hypothetical protein |
| [OCAR_5536](https://www.genoscope.cns.fr/agc/mage/wwwpkgdb/Info/getInfoLabel.php?id=3482978&wwwpkgdb=fc2733d6468c073f1dd738aa598ede55&nocache=a9a895ad49f0f55c362d050dc1ad3fa3&dir=&wwwpkgdb=fc2733d6468c073f1dd738aa598ede55) | _ | hypothetical protein |
| [OCAR_1532](https://www.genoscope.cns.fr/agc/mage/wwwpkgdb/Info/getInfoLabel.php?id=3482132&wwwpkgdb=fc2733d6468c073f1dd738aa598ede55&nocache=a9a895ad49f0f55c362d050dc1ad3fa3&dir=&wwwpkgdb=fc2733d6468c073f1dd738aa598ede55) | _ | _ |
| [OCAR_5538](https://www.genoscope.cns.fr/agc/mage/wwwpkgdb/Info/getInfoLabel.php?id=3484744&wwwpkgdb=fc2733d6468c073f1dd738aa598ede55&nocache=a9a895ad49f0f55c362d050dc1ad3fa3&dir=&wwwpkgdb=fc2733d6468c073f1dd738aa598ede55) | _ | hypothetical protein |
| [OCAR_5541](https://www.genoscope.cns.fr/agc/mage/wwwpkgdb/Info/getInfoLabel.php?id=3484747&wwwpkgdb=fc2733d6468c073f1dd738aa598ede55&nocache=a9a895ad49f0f55c362d050dc1ad3fa3&dir=&wwwpkgdb=fc2733d6468c073f1dd738aa598ede55) | _ | rect protein |
| [OCAR_5542](https://www.genoscope.cns.fr/agc/mage/wwwpkgdb/Info/getInfoLabel.php?id=3484748&wwwpkgdb=fc2733d6468c073f1dd738aa598ede55&nocache=a9a895ad49f0f55c362d050dc1ad3fa3&dir=&wwwpkgdb=fc2733d6468c073f1dd738aa598ede55) | _ | hypothetical protein |
| [OCAR_5543](https://www.genoscope.cns.fr/agc/mage/wwwpkgdb/Info/getInfoLabel.php?id=3484749&wwwpkgdb=fc2733d6468c073f1dd738aa598ede55&nocache=a9a895ad49f0f55c362d050dc1ad3fa3&dir=&wwwpkgdb=fc2733d6468c073f1dd738aa598ede55) | _ | putative conserved phage protein |
| [OCAR_5544](https://www.genoscope.cns.fr/agc/mage/wwwpkgdb/Info/getInfoLabel.php?id=3484750&wwwpkgdb=fc2733d6468c073f1dd738aa598ede55&nocache=a9a895ad49f0f55c362d050dc1ad3fa3&dir=&wwwpkgdb=fc2733d6468c073f1dd738aa598ede55) | _ | hypothetical protein |
| [OCAR_5545](https://www.genoscope.cns.fr/agc/mage/wwwpkgdb/Info/getInfoLabel.php?id=3484751&wwwpkgdb=fc2733d6468c073f1dd738aa598ede55&nocache=a9a895ad49f0f55c362d050dc1ad3fa3&dir=&wwwpkgdb=fc2733d6468c073f1dd738aa598ede55) | _ | hypothetical protein |
| [OCAR_1541](https://www.genoscope.cns.fr/agc/mage/wwwpkgdb/Info/getInfoLabel.php?id=3482013&wwwpkgdb=fc2733d6468c073f1dd738aa598ede55&nocache=a9a895ad49f0f55c362d050dc1ad3fa3&dir=&wwwpkgdb=fc2733d6468c073f1dd738aa598ede55) | _ | _ |
| [OCAR_5546](https://www.genoscope.cns.fr/agc/mage/wwwpkgdb/Info/getInfoLabel.php?id=3484752&wwwpkgdb=fc2733d6468c073f1dd738aa598ede55&nocache=a9a895ad49f0f55c362d050dc1ad3fa3&dir=&wwwpkgdb=fc2733d6468c073f1dd738aa598ede55) | _ | hypothetical protein |
| [OCAR_5547](https://www.genoscope.cns.fr/agc/mage/wwwpkgdb/Info/getInfoLabel.php?id=3484753&wwwpkgdb=fc2733d6468c073f1dd738aa598ede55&nocache=a9a895ad49f0f55c362d050dc1ad3fa3&dir=&wwwpkgdb=fc2733d6468c073f1dd738aa598ede55) | _ | hypothetical protein |
| [OCAR_5548](https://www.genoscope.cns.fr/agc/mage/wwwpkgdb/Info/getInfoLabel.php?id=3482979&wwwpkgdb=fc2733d6468c073f1dd738aa598ede55&nocache=a9a895ad49f0f55c362d050dc1ad3fa3&dir=&wwwpkgdb=fc2733d6468c073f1dd738aa598ede55) | _ | hypothetical protein |
| [OCAR_1545](https://www.genoscope.cns.fr/agc/mage/wwwpkgdb/Info/getInfoLabel.php?id=3482131&wwwpkgdb=fc2733d6468c073f1dd738aa598ede55&nocache=a9a895ad49f0f55c362d050dc1ad3fa3&dir=&wwwpkgdb=fc2733d6468c073f1dd738aa598ede55) | _ | _ |
| [OCAR_5549](https://www.genoscope.cns.fr/agc/mage/wwwpkgdb/Info/getInfoLabel.php?id=3484754&wwwpkgdb=fc2733d6468c073f1dd738aa598ede55&nocache=a9a895ad49f0f55c362d050dc1ad3fa3&dir=&wwwpkgdb=fc2733d6468c073f1dd738aa598ede55) | _ | hypothetical protein |
| [OCAR_5553](https://www.genoscope.cns.fr/agc/mage/wwwpkgdb/Info/getInfoLabel.php?id=3482982&wwwpkgdb=fc2733d6468c073f1dd738aa598ede55&nocache=a9a895ad49f0f55c362d050dc1ad3fa3&dir=&wwwpkgdb=fc2733d6468c073f1dd738aa598ede55) | _ | hypothetical protein |
| [OCAR_5554](https://www.genoscope.cns.fr/agc/mage/wwwpkgdb/Info/getInfoLabel.php?id=3482983&wwwpkgdb=fc2733d6468c073f1dd738aa598ede55&nocache=a9a895ad49f0f55c362d050dc1ad3fa3&dir=&wwwpkgdb=fc2733d6468c073f1dd738aa598ede55) | _ | hypothetical protein |
| [OCAR_5555](https://www.genoscope.cns.fr/agc/mage/wwwpkgdb/Info/getInfoLabel.php?id=3482984&wwwpkgdb=fc2733d6468c073f1dd738aa598ede55&nocache=a9a895ad49f0f55c362d050dc1ad3fa3&dir=&wwwpkgdb=fc2733d6468c073f1dd738aa598ede55) | _ | holliday junction resolvasome, endonuclease subunit |
| [OCAR_5556](https://www.genoscope.cns.fr/agc/mage/wwwpkgdb/Info/getInfoLabel.php?id=3482985&wwwpkgdb=fc2733d6468c073f1dd738aa598ede55&nocache=a9a895ad49f0f55c362d050dc1ad3fa3&dir=&wwwpkgdb=fc2733d6468c073f1dd738aa598ede55) | _ | DNA methylase N-4/N-6 domain protein |
| [OCAR_5557](https://www.genoscope.cns.fr/agc/mage/wwwpkgdb/Info/getInfoLabel.php?id=3482986&wwwpkgdb=fc2733d6468c073f1dd738aa598ede55&nocache=a9a895ad49f0f55c362d050dc1ad3fa3&dir=&wwwpkgdb=fc2733d6468c073f1dd738aa598ede55) | _ | hypothetical protein |
| [OCAR_5558](https://www.genoscope.cns.fr/agc/mage/wwwpkgdb/Info/getInfoLabel.php?id=3482987&wwwpkgdb=fc2733d6468c073f1dd738aa598ede55&nocache=a9a895ad49f0f55c362d050dc1ad3fa3&dir=&wwwpkgdb=fc2733d6468c073f1dd738aa598ede55) | _ | putative replicative DNA helicase |
| [OCAR_5559](https://www.genoscope.cns.fr/agc/mage/wwwpkgdb/Info/getInfoLabel.php?id=3482988&wwwpkgdb=fc2733d6468c073f1dd738aa598ede55&nocache=a9a895ad49f0f55c362d050dc1ad3fa3&dir=&wwwpkgdb=fc2733d6468c073f1dd738aa598ede55) | _ | hypothetical protein |
| [OCAR_5560](https://www.genoscope.cns.fr/agc/mage/wwwpkgdb/Info/getInfoLabel.php?id=3482989&wwwpkgdb=fc2733d6468c073f1dd738aa598ede55&nocache=a9a895ad49f0f55c362d050dc1ad3fa3&dir=&wwwpkgdb=fc2733d6468c073f1dd738aa598ede55) | _ | hypothetical protein |
| [OCAR_5561](https://www.genoscope.cns.fr/agc/mage/wwwpkgdb/Info/getInfoLabel.php?id=3482990&wwwpkgdb=fc2733d6468c073f1dd738aa598ede55&nocache=a9a895ad49f0f55c362d050dc1ad3fa3&dir=&wwwpkgdb=fc2733d6468c073f1dd738aa598ede55) | _ | hypothetical protein |
| [OCAR_5562](https://www.genoscope.cns.fr/agc/mage/wwwpkgdb/Info/getInfoLabel.php?id=3482991&wwwpkgdb=fc2733d6468c073f1dd738aa598ede55&nocache=a9a895ad49f0f55c362d050dc1ad3fa3&dir=&wwwpkgdb=fc2733d6468c073f1dd738aa598ede55) | _ | hypothetical protein |
| [OCAR_5563](https://www.genoscope.cns.fr/agc/mage/wwwpkgdb/Info/getInfoLabel.php?id=3482992&wwwpkgdb=fc2733d6468c073f1dd738aa598ede55&nocache=a9a895ad49f0f55c362d050dc1ad3fa3&dir=&wwwpkgdb=fc2733d6468c073f1dd738aa598ede55) | _ | hypothetical protein |
| [OCAR_5564](https://www.genoscope.cns.fr/agc/mage/wwwpkgdb/Info/getInfoLabel.php?id=3482993&wwwpkgdb=fc2733d6468c073f1dd738aa598ede55&nocache=a9a895ad49f0f55c362d050dc1ad3fa3&dir=&wwwpkgdb=fc2733d6468c073f1dd738aa598ede55) | _ | hypothetical protein |
| [OCAR_5565](https://www.genoscope.cns.fr/agc/mage/wwwpkgdb/Info/getInfoLabel.php?id=3482994&wwwpkgdb=fc2733d6468c073f1dd738aa598ede55&nocache=a9a895ad49f0f55c362d050dc1ad3fa3&dir=&wwwpkgdb=fc2733d6468c073f1dd738aa598ede55) | _ | C-5 cytosine-specific DNA methylase |
| [OCAR_5566](https://www.genoscope.cns.fr/agc/mage/wwwpkgdb/Info/getInfoLabel.php?id=3482995&wwwpkgdb=fc2733d6468c073f1dd738aa598ede55&nocache=a9a895ad49f0f55c362d050dc1ad3fa3&dir=&wwwpkgdb=fc2733d6468c073f1dd738aa598ede55) | _ | hypothetical protein |
| [OCAR_1564](https://www.genoscope.cns.fr/agc/mage/wwwpkgdb/Info/getInfoLabel.php?id=3482012&wwwpkgdb=fc2733d6468c073f1dd738aa598ede55&nocache=a9a895ad49f0f55c362d050dc1ad3fa3&dir=&wwwpkgdb=fc2733d6468c073f1dd738aa598ede55) | _ | _ |
| [OCAR_5567](https://www.genoscope.cns.fr/agc/mage/wwwpkgdb/Info/getInfoLabel.php?id=3482996&wwwpkgdb=fc2733d6468c073f1dd738aa598ede55&nocache=a9a895ad49f0f55c362d050dc1ad3fa3&dir=&wwwpkgdb=fc2733d6468c073f1dd738aa598ede55) | _ | hypothetical protein |
| [OCAR_5568](https://www.genoscope.cns.fr/agc/mage/wwwpkgdb/Info/getInfoLabel.php?id=3482997&wwwpkgdb=fc2733d6468c073f1dd738aa598ede55&nocache=a9a895ad49f0f55c362d050dc1ad3fa3&dir=&wwwpkgdb=fc2733d6468c073f1dd738aa598ede55) | _ | hypothetical protein |
| [OCAR_5569](https://www.genoscope.cns.fr/agc/mage/wwwpkgdb/Info/getInfoLabel.php?id=3482998&wwwpkgdb=fc2733d6468c073f1dd738aa598ede55&nocache=a9a895ad49f0f55c362d050dc1ad3fa3&dir=&wwwpkgdb=fc2733d6468c073f1dd738aa598ede55) | _ | hypothetical protein |
| [OCAR_1568](https://www.genoscope.cns.fr/agc/mage/wwwpkgdb/Info/getInfoLabel.php?id=3482011&wwwpkgdb=fc2733d6468c073f1dd738aa598ede55&nocache=a9a895ad49f0f55c362d050dc1ad3fa3&dir=&wwwpkgdb=fc2733d6468c073f1dd738aa598ede55) | _ | _ |
| [OCAR_5570](https://www.genoscope.cns.fr/agc/mage/wwwpkgdb/Info/getInfoLabel.php?id=3482999&wwwpkgdb=fc2733d6468c073f1dd738aa598ede55&nocache=a9a895ad49f0f55c362d050dc1ad3fa3&dir=&wwwpkgdb=fc2733d6468c073f1dd738aa598ede55) | _ | hypothetical protein |
| [OCAR_5571](https://www.genoscope.cns.fr/agc/mage/wwwpkgdb/Info/getInfoLabel.php?id=3483000&wwwpkgdb=fc2733d6468c073f1dd738aa598ede55&nocache=a9a895ad49f0f55c362d050dc1ad3fa3&dir=&wwwpkgdb=fc2733d6468c073f1dd738aa598ede55) | _ | hypothetical protein |
| [OCAR_1572](https://www.genoscope.cns.fr/agc/mage/wwwpkgdb/Info/getInfoLabel.php?id=3482010&wwwpkgdb=fc2733d6468c073f1dd738aa598ede55&nocache=a9a895ad49f0f55c362d050dc1ad3fa3&dir=&wwwpkgdb=fc2733d6468c073f1dd738aa598ede55) | _ | _ |
| [OCAR_5573](https://www.genoscope.cns.fr/agc/mage/wwwpkgdb/Info/getInfoLabel.php?id=3484757&wwwpkgdb=fc2733d6468c073f1dd738aa598ede55&nocache=a9a895ad49f0f55c362d050dc1ad3fa3&dir=&wwwpkgdb=fc2733d6468c073f1dd738aa598ede55) | _ | hypothetical protein |
| [OCAR_5575](https://www.genoscope.cns.fr/agc/mage/wwwpkgdb/Info/getInfoLabel.php?id=3483002&wwwpkgdb=fc2733d6468c073f1dd738aa598ede55&nocache=a9a895ad49f0f55c362d050dc1ad3fa3&dir=&wwwpkgdb=fc2733d6468c073f1dd738aa598ede55) | _ | hypothetical protein |
| [OCAR_5576](https://www.genoscope.cns.fr/agc/mage/wwwpkgdb/Info/getInfoLabel.php?id=3483003&wwwpkgdb=fc2733d6468c073f1dd738aa598ede55&nocache=a9a895ad49f0f55c362d050dc1ad3fa3&dir=&wwwpkgdb=fc2733d6468c073f1dd738aa598ede55) | _ | phage terminase large subunit (GpA) |
| [OCAR_5579](https://www.genoscope.cns.fr/agc/mage/wwwpkgdb/Info/getInfoLabel.php?id=3483006&wwwpkgdb=fc2733d6468c073f1dd738aa598ede55&nocache=a9a895ad49f0f55c362d050dc1ad3fa3&dir=&wwwpkgdb=fc2733d6468c073f1dd738aa598ede55) | _ | hypothetical protein |
| [OCAR_5580](https://www.genoscope.cns.fr/agc/mage/wwwpkgdb/Info/getInfoLabel.php?id=3483007&wwwpkgdb=fc2733d6468c073f1dd738aa598ede55&nocache=a9a895ad49f0f55c362d050dc1ad3fa3&dir=&wwwpkgdb=fc2733d6468c073f1dd738aa598ede55) | _ | peptidase S49 |
| [OCAR_5587](https://www.genoscope.cns.fr/agc/mage/wwwpkgdb/Info/getInfoLabel.php?id=3483014&wwwpkgdb=fc2733d6468c073f1dd738aa598ede55&nocache=a9a895ad49f0f55c362d050dc1ad3fa3&dir=&wwwpkgdb=fc2733d6468c073f1dd738aa598ede55) | _ | phage tail protein I |
| [OCAR_5588](https://www.genoscope.cns.fr/agc/mage/wwwpkgdb/Info/getInfoLabel.php?id=3483015&wwwpkgdb=fc2733d6468c073f1dd738aa598ede55&nocache=a9a895ad49f0f55c362d050dc1ad3fa3&dir=&wwwpkgdb=fc2733d6468c073f1dd738aa598ede55) | _ | hypothetical protein |
| [OCAR_5589](https://www.genoscope.cns.fr/agc/mage/wwwpkgdb/Info/getInfoLabel.php?id=3483016&wwwpkgdb=fc2733d6468c073f1dd738aa598ede55&nocache=a9a895ad49f0f55c362d050dc1ad3fa3&dir=&wwwpkgdb=fc2733d6468c073f1dd738aa598ede55) | _ | hypothetical protein |
| [OCAR_5590](https://www.genoscope.cns.fr/agc/mage/wwwpkgdb/Info/getInfoLabel.php?id=3483017&wwwpkgdb=fc2733d6468c073f1dd738aa598ede55&nocache=a9a895ad49f0f55c362d050dc1ad3fa3&dir=&wwwpkgdb=fc2733d6468c073f1dd738aa598ede55) | _ | putative tail fiber protein H |
| [OCAR_5591](https://www.genoscope.cns.fr/agc/mage/wwwpkgdb/Info/getInfoLabel.php?id=3483018&wwwpkgdb=fc2733d6468c073f1dd738aa598ede55&nocache=a9a895ad49f0f55c362d050dc1ad3fa3&dir=&wwwpkgdb=fc2733d6468c073f1dd738aa598ede55) | _ | hypothetical protein |
| [OCAR_5592](https://www.genoscope.cns.fr/agc/mage/wwwpkgdb/Info/getInfoLabel.php?id=3483019&wwwpkgdb=fc2733d6468c073f1dd738aa598ede55&nocache=a9a895ad49f0f55c362d050dc1ad3fa3&dir=&wwwpkgdb=fc2733d6468c073f1dd738aa598ede55) | _ | putative major tail sheath protein FI |
| [OCAR_5593](https://www.genoscope.cns.fr/agc/mage/wwwpkgdb/Info/getInfoLabel.php?id=3483020&wwwpkgdb=fc2733d6468c073f1dd738aa598ede55&nocache=a9a895ad49f0f55c362d050dc1ad3fa3&dir=&wwwpkgdb=fc2733d6468c073f1dd738aa598ede55) | _ | putative phage major tail tube protein |
| [OCAR_5594](https://www.genoscope.cns.fr/agc/mage/wwwpkgdb/Info/getInfoLabel.php?id=3483021&wwwpkgdb=fc2733d6468c073f1dd738aa598ede55&nocache=a9a895ad49f0f55c362d050dc1ad3fa3&dir=&wwwpkgdb=fc2733d6468c073f1dd738aa598ede55) | _ | hypothetical protein |
| [OCAR_5595](https://www.genoscope.cns.fr/agc/mage/wwwpkgdb/Info/getInfoLabel.php?id=3483022&wwwpkgdb=fc2733d6468c073f1dd738aa598ede55&nocache=a9a895ad49f0f55c362d050dc1ad3fa3&dir=&wwwpkgdb=fc2733d6468c073f1dd738aa598ede55) | _ | hypothetical protein |
| [OCAR_5596](https://www.genoscope.cns.fr/agc/mage/wwwpkgdb/Info/getInfoLabel.php?id=3483023&wwwpkgdb=fc2733d6468c073f1dd738aa598ede55&nocache=a9a895ad49f0f55c362d050dc1ad3fa3&dir=&wwwpkgdb=fc2733d6468c073f1dd738aa598ede55) | _ | putative tail protein U |
| [OCAR_5600](https://www.genoscope.cns.fr/agc/mage/wwwpkgdb/Info/getInfoLabel.php?id=3483027&wwwpkgdb=fc2733d6468c073f1dd738aa598ede55&nocache=a9a895ad49f0f55c362d050dc1ad3fa3&dir=&wwwpkgdb=fc2733d6468c073f1dd738aa598ede55) | _ | hypothetical protein |
| [OCAR_5601](https://www.genoscope.cns.fr/agc/mage/wwwpkgdb/Info/getInfoLabel.php?id=3483028&wwwpkgdb=fc2733d6468c073f1dd738aa598ede55&nocache=a9a895ad49f0f55c362d050dc1ad3fa3&dir=&wwwpkgdb=fc2733d6468c073f1dd738aa598ede55) | _ | hypothetical protein |
| [OCAR_5602](https://www.genoscope.cns.fr/agc/mage/wwwpkgdb/Info/getInfoLabel.php?id=3483029&wwwpkgdb=fc2733d6468c073f1dd738aa598ede55&nocache=a9a895ad49f0f55c362d050dc1ad3fa3&dir=&wwwpkgdb=fc2733d6468c073f1dd738aa598ede55) | _ | hypothetical protein |
| [OCAR_5603](https://www.genoscope.cns.fr/agc/mage/wwwpkgdb/Info/getInfoLabel.php?id=3483030&wwwpkgdb=fc2733d6468c073f1dd738aa598ede55&nocache=a9a895ad49f0f55c362d050dc1ad3fa3&dir=&wwwpkgdb=fc2733d6468c073f1dd738aa598ede55) | _ | hypothetical protein |
| [OCAR_5606](https://www.genoscope.cns.fr/agc/mage/wwwpkgdb/Info/getInfoLabel.php?id=3483033&wwwpkgdb=fc2733d6468c073f1dd738aa598ede55&nocache=a9a895ad49f0f55c362d050dc1ad3fa3&dir=&wwwpkgdb=fc2733d6468c073f1dd738aa598ede55) | _ | hypothetical protein |
| [OCAR_5611](https://www.genoscope.cns.fr/agc/mage/wwwpkgdb/Info/getInfoLabel.php?id=3483036&wwwpkgdb=fc2733d6468c073f1dd738aa598ede55&nocache=a9a895ad49f0f55c362d050dc1ad3fa3&dir=&wwwpkgdb=fc2733d6468c073f1dd738aa598ede55) | _ | hypothetical protein |
| [OCAR_5614](https://www.genoscope.cns.fr/agc/mage/wwwpkgdb/Info/getInfoLabel.php?id=3483038&wwwpkgdb=fc2733d6468c073f1dd738aa598ede55&nocache=a9a895ad49f0f55c362d050dc1ad3fa3&dir=&wwwpkgdb=fc2733d6468c073f1dd738aa598ede55) | _ | hypothetical protein |
| [OCAR_5620](https://www.genoscope.cns.fr/agc/mage/wwwpkgdb/Info/getInfoLabel.php?id=3483039&wwwpkgdb=fc2733d6468c073f1dd738aa598ede55&nocache=a9a895ad49f0f55c362d050dc1ad3fa3&dir=&wwwpkgdb=fc2733d6468c073f1dd738aa598ede55) | _ | hypothetical protein |
| [OCAR_5623](https://www.genoscope.cns.fr/agc/mage/wwwpkgdb/Info/getInfoLabel.php?id=3484767&wwwpkgdb=fc2733d6468c073f1dd738aa598ede55&nocache=a9a895ad49f0f55c362d050dc1ad3fa3&dir=&wwwpkgdb=fc2733d6468c073f1dd738aa598ede55) | _ | hypothetical protein |
| [OCAR_5635](https://www.genoscope.cns.fr/agc/mage/wwwpkgdb/Info/getInfoLabel.php?id=3483048&wwwpkgdb=fc2733d6468c073f1dd738aa598ede55&nocache=a9a895ad49f0f55c362d050dc1ad3fa3&dir=&wwwpkgdb=fc2733d6468c073f1dd738aa598ede55) | _ | hypothetical protein |
| [OCAR_1631](https://www.genoscope.cns.fr/agc/mage/wwwpkgdb/Info/getInfoLabel.php?id=3482008&wwwpkgdb=fc2733d6468c073f1dd738aa598ede55&nocache=a9a895ad49f0f55c362d050dc1ad3fa3&dir=&wwwpkgdb=fc2733d6468c073f1dd738aa598ede55) | _ | _ |
| [OCAR_1633](https://www.genoscope.cns.fr/agc/mage/wwwpkgdb/Info/getInfoLabel.php?id=3482007&wwwpkgdb=fc2733d6468c073f1dd738aa598ede55&nocache=a9a895ad49f0f55c362d050dc1ad3fa3&dir=&wwwpkgdb=fc2733d6468c073f1dd738aa598ede55) | _ | _ |
| [OCAR_5642](https://www.genoscope.cns.fr/agc/mage/wwwpkgdb/Info/getInfoLabel.php?id=3484776&wwwpkgdb=fc2733d6468c073f1dd738aa598ede55&nocache=a9a895ad49f0f55c362d050dc1ad3fa3&dir=&wwwpkgdb=fc2733d6468c073f1dd738aa598ede55) | _ | hypothetical protein |
| [OCAR_5644](https://www.genoscope.cns.fr/agc/mage/wwwpkgdb/Info/getInfoLabel.php?id=3484777&wwwpkgdb=fc2733d6468c073f1dd738aa598ede55&nocache=a9a895ad49f0f55c362d050dc1ad3fa3&dir=&wwwpkgdb=fc2733d6468c073f1dd738aa598ede55) | _ | transcriptional regulator, IclR family |
| [OCAR_5646](https://www.genoscope.cns.fr/agc/mage/wwwpkgdb/Info/getInfoLabel.php?id=3484778&wwwpkgdb=fc2733d6468c073f1dd738aa598ede55&nocache=a9a895ad49f0f55c362d050dc1ad3fa3&dir=&wwwpkgdb=fc2733d6468c073f1dd738aa598ede55) | _ | hypothetical protein |
| [OCAR_5647](https://www.genoscope.cns.fr/agc/mage/wwwpkgdb/Info/getInfoLabel.php?id=3484779&wwwpkgdb=fc2733d6468c073f1dd738aa598ede55&nocache=a9a895ad49f0f55c362d050dc1ad3fa3&dir=&wwwpkgdb=fc2733d6468c073f1dd738aa598ede55) | _ | putative protein of unknown function |
| [OCAR_5648](https://www.genoscope.cns.fr/agc/mage/wwwpkgdb/Info/getInfoLabel.php?id=3483053&wwwpkgdb=fc2733d6468c073f1dd738aa598ede55&nocache=a9a895ad49f0f55c362d050dc1ad3fa3&dir=&wwwpkgdb=fc2733d6468c073f1dd738aa598ede55) | _ | hypothetical protein |
| [OCAR_5651](https://www.genoscope.cns.fr/agc/mage/wwwpkgdb/Info/getInfoLabel.php?id=3483056&wwwpkgdb=fc2733d6468c073f1dd738aa598ede55&nocache=a9a895ad49f0f55c362d050dc1ad3fa3&dir=&wwwpkgdb=fc2733d6468c073f1dd738aa598ede55) | _ | thermosome alpha subunit |
| [OCAR_5652](https://www.genoscope.cns.fr/agc/mage/wwwpkgdb/Info/getInfoLabel.php?id=3483057&wwwpkgdb=fc2733d6468c073f1dd738aa598ede55&nocache=a9a895ad49f0f55c362d050dc1ad3fa3&dir=&wwwpkgdb=fc2733d6468c073f1dd738aa598ede55) | _ | hypothetical protein |
| [OCAR_5658](https://www.genoscope.cns.fr/agc/mage/wwwpkgdb/Info/getInfoLabel.php?id=3484781&wwwpkgdb=fc2733d6468c073f1dd738aa598ede55&nocache=a9a895ad49f0f55c362d050dc1ad3fa3&dir=&wwwpkgdb=fc2733d6468c073f1dd738aa598ede55) | _ | hypothetical protein |
| [OCAR_5664](https://www.genoscope.cns.fr/agc/mage/wwwpkgdb/Info/getInfoLabel.php?id=3484782&wwwpkgdb=fc2733d6468c073f1dd738aa598ede55&nocache=a9a895ad49f0f55c362d050dc1ad3fa3&dir=&wwwpkgdb=fc2733d6468c073f1dd738aa598ede55) | _ | hypothetical protein |
| [OCAR_5668](https://www.genoscope.cns.fr/agc/mage/wwwpkgdb/Info/getInfoLabel.php?id=3483070&wwwpkgdb=fc2733d6468c073f1dd738aa598ede55&nocache=a9a895ad49f0f55c362d050dc1ad3fa3&dir=&wwwpkgdb=fc2733d6468c073f1dd738aa598ede55) | _ | glycosyl transferase, family 2 |
| [OCAR_1661](https://www.genoscope.cns.fr/agc/mage/wwwpkgdb/Info/getInfoLabel.php?id=3482005&wwwpkgdb=fc2733d6468c073f1dd738aa598ede55&nocache=a9a895ad49f0f55c362d050dc1ad3fa3&dir=&wwwpkgdb=fc2733d6468c073f1dd738aa598ede55) | _ | _ |
| [OCAR_5671](https://www.genoscope.cns.fr/agc/mage/wwwpkgdb/Info/getInfoLabel.php?id=3484784&wwwpkgdb=fc2733d6468c073f1dd738aa598ede55&nocache=a9a895ad49f0f55c362d050dc1ad3fa3&dir=&wwwpkgdb=fc2733d6468c073f1dd738aa598ede55) | _ | hypothetical protein |
| [OCAR_5703](https://www.genoscope.cns.fr/agc/mage/wwwpkgdb/Info/getInfoLabel.php?id=3483103&wwwpkgdb=fc2733d6468c073f1dd738aa598ede55&nocache=a9a895ad49f0f55c362d050dc1ad3fa3&dir=&wwwpkgdb=fc2733d6468c073f1dd738aa598ede55) | _ | ankyrin repeat protein containing four repeats |
| [OCAR_5705](https://www.genoscope.cns.fr/agc/mage/wwwpkgdb/Info/getInfoLabel.php?id=3484785&wwwpkgdb=fc2733d6468c073f1dd738aa598ede55&nocache=a9a895ad49f0f55c362d050dc1ad3fa3&dir=&wwwpkgdb=fc2733d6468c073f1dd738aa598ede55) | _ | exopolysaccharide production |
| [OCAR_5711](https://www.genoscope.cns.fr/agc/mage/wwwpkgdb/Info/getInfoLabel.php?id=3483110&wwwpkgdb=fc2733d6468c073f1dd738aa598ede55&nocache=a9a895ad49f0f55c362d050dc1ad3fa3&dir=&wwwpkgdb=fc2733d6468c073f1dd738aa598ede55) | _ | hypothetical protein |
| [OCAR_5716](https://www.genoscope.cns.fr/agc/mage/wwwpkgdb/Info/getInfoLabel.php?id=3484787&wwwpkgdb=fc2733d6468c073f1dd738aa598ede55&nocache=a9a895ad49f0f55c362d050dc1ad3fa3&dir=&wwwpkgdb=fc2733d6468c073f1dd738aa598ede55) | _ | hypothetical protein |
| [OCAR_1711](https://www.genoscope.cns.fr/agc/mage/wwwpkgdb/Info/getInfoLabel.php?id=3482004&wwwpkgdb=fc2733d6468c073f1dd738aa598ede55&nocache=a9a895ad49f0f55c362d050dc1ad3fa3&dir=&wwwpkgdb=fc2733d6468c073f1dd738aa598ede55) | _ | _ |
| [OCAR_1712](https://www.genoscope.cns.fr/agc/mage/wwwpkgdb/Info/getInfoLabel.php?id=3482003&wwwpkgdb=fc2733d6468c073f1dd738aa598ede55&nocache=a9a895ad49f0f55c362d050dc1ad3fa3&dir=&wwwpkgdb=fc2733d6468c073f1dd738aa598ede55) | _ | _ |
| [OCAR_5722](https://www.genoscope.cns.fr/agc/mage/wwwpkgdb/Info/getInfoLabel.php?id=3484790&wwwpkgdb=fc2733d6468c073f1dd738aa598ede55&nocache=a9a895ad49f0f55c362d050dc1ad3fa3&dir=&wwwpkgdb=fc2733d6468c073f1dd738aa598ede55) | _ | NAD-dependent epimerase/dehydratase |
| [OCAR_5723](https://www.genoscope.cns.fr/agc/mage/wwwpkgdb/Info/getInfoLabel.php?id=3484791&wwwpkgdb=fc2733d6468c073f1dd738aa598ede55&nocache=a9a895ad49f0f55c362d050dc1ad3fa3&dir=&wwwpkgdb=fc2733d6468c073f1dd738aa598ede55) | _ | alpha/beta superfamily hydrolase |
| [OCAR_5724](https://www.genoscope.cns.fr/agc/mage/wwwpkgdb/Info/getInfoLabel.php?id=3484792&wwwpkgdb=fc2733d6468c073f1dd738aa598ede55&nocache=a9a895ad49f0f55c362d050dc1ad3fa3&dir=&wwwpkgdb=fc2733d6468c073f1dd738aa598ede55) | _ | alpha/beta superfamily hydrolase |
| [OCAR_5726](https://www.genoscope.cns.fr/agc/mage/wwwpkgdb/Info/getInfoLabel.php?id=3483119&wwwpkgdb=fc2733d6468c073f1dd738aa598ede55&nocache=a9a895ad49f0f55c362d050dc1ad3fa3&dir=&wwwpkgdb=fc2733d6468c073f1dd738aa598ede55) | _ | hypothetical protein |
| [OCAR_5753](https://www.genoscope.cns.fr/agc/mage/wwwpkgdb/Info/getInfoLabel.php?id=3484803&wwwpkgdb=fc2733d6468c073f1dd738aa598ede55&nocache=a9a895ad49f0f55c362d050dc1ad3fa3&dir=&wwwpkgdb=fc2733d6468c073f1dd738aa598ede55) | _ | probable glycin-rich signal peptide protein |
| [OCAR_1751](https://www.genoscope.cns.fr/agc/mage/wwwpkgdb/Info/getInfoLabel.php?id=3482002&wwwpkgdb=fc2733d6468c073f1dd738aa598ede55&nocache=a9a895ad49f0f55c362d050dc1ad3fa3&dir=&wwwpkgdb=fc2733d6468c073f1dd738aa598ede55) | _ | _ |
| [OCAR_5764](https://www.genoscope.cns.fr/agc/mage/wwwpkgdb/Info/getInfoLabel.php?id=3484807&wwwpkgdb=fc2733d6468c073f1dd738aa598ede55&nocache=a9a895ad49f0f55c362d050dc1ad3fa3&dir=&wwwpkgdb=fc2733d6468c073f1dd738aa598ede55) | _ | hypothetical protein |
| [OCAR_1758](https://www.genoscope.cns.fr/agc/mage/wwwpkgdb/Info/getInfoLabel.php?id=3482001&wwwpkgdb=fc2733d6468c073f1dd738aa598ede55&nocache=a9a895ad49f0f55c362d050dc1ad3fa3&dir=&wwwpkgdb=fc2733d6468c073f1dd738aa598ede55) | _ | _ |
| [OCAR_5766](https://www.genoscope.cns.fr/agc/mage/wwwpkgdb/Info/getInfoLabel.php?id=3484808&wwwpkgdb=fc2733d6468c073f1dd738aa598ede55&nocache=a9a895ad49f0f55c362d050dc1ad3fa3&dir=&wwwpkgdb=fc2733d6468c073f1dd738aa598ede55) | _ | hypothetical protein |
| [OCAR_5768](https://www.genoscope.cns.fr/agc/mage/wwwpkgdb/Info/getInfoLabel.php?id=3484809&wwwpkgdb=fc2733d6468c073f1dd738aa598ede55&nocache=a9a895ad49f0f55c362d050dc1ad3fa3&dir=&wwwpkgdb=fc2733d6468c073f1dd738aa598ede55) | _ | hypothetical protein |
| [OCAR_5789](https://www.genoscope.cns.fr/agc/mage/wwwpkgdb/Info/getInfoLabel.php?id=3484825&wwwpkgdb=fc2733d6468c073f1dd738aa598ede55&nocache=a9a895ad49f0f55c362d050dc1ad3fa3&dir=&wwwpkgdb=fc2733d6468c073f1dd738aa598ede55) | _ | hypothetical protein |
| [OCAR_5790](https://www.genoscope.cns.fr/agc/mage/wwwpkgdb/Info/getInfoLabel.php?id=3483150&wwwpkgdb=fc2733d6468c073f1dd738aa598ede55&nocache=a9a895ad49f0f55c362d050dc1ad3fa3&dir=&wwwpkgdb=fc2733d6468c073f1dd738aa598ede55) | _ | hypothetical protein |
| [OCAR_5798](https://www.genoscope.cns.fr/agc/mage/wwwpkgdb/Info/getInfoLabel.php?id=3483157&wwwpkgdb=fc2733d6468c073f1dd738aa598ede55&nocache=a9a895ad49f0f55c362d050dc1ad3fa3&dir=&wwwpkgdb=fc2733d6468c073f1dd738aa598ede55) | _ | T-protein |
| [OCAR_5801](https://www.genoscope.cns.fr/agc/mage/wwwpkgdb/Info/getInfoLabel.php?id=3484827&wwwpkgdb=fc2733d6468c073f1dd738aa598ede55&nocache=a9a895ad49f0f55c362d050dc1ad3fa3&dir=&wwwpkgdb=fc2733d6468c073f1dd738aa598ede55) | _ | hypothetical protein |
| [OCAR_5802](https://www.genoscope.cns.fr/agc/mage/wwwpkgdb/Info/getInfoLabel.php?id=3484828&wwwpkgdb=fc2733d6468c073f1dd738aa598ede55&nocache=a9a895ad49f0f55c362d050dc1ad3fa3&dir=&wwwpkgdb=fc2733d6468c073f1dd738aa598ede55) | _ | hypothetical protein |
| [OCAR_5810](https://www.genoscope.cns.fr/agc/mage/wwwpkgdb/Info/getInfoLabel.php?id=3484830&wwwpkgdb=fc2733d6468c073f1dd738aa598ede55&nocache=a9a895ad49f0f55c362d050dc1ad3fa3&dir=&wwwpkgdb=fc2733d6468c073f1dd738aa598ede55) | _ | UDP-N-acetylglucosamine--peptide N-acetylglucosaminyltransferase |
| [OCAR_5811](https://www.genoscope.cns.fr/agc/mage/wwwpkgdb/Info/getInfoLabel.php?id=3483166&wwwpkgdb=fc2733d6468c073f1dd738aa598ede55&nocache=a9a895ad49f0f55c362d050dc1ad3fa3&dir=&wwwpkgdb=fc2733d6468c073f1dd738aa598ede55) | _ | hypothetical protein |
| [OCAR_5814](https://www.genoscope.cns.fr/agc/mage/wwwpkgdb/Info/getInfoLabel.php?id=3483167&wwwpkgdb=fc2733d6468c073f1dd738aa598ede55&nocache=a9a895ad49f0f55c362d050dc1ad3fa3&dir=&wwwpkgdb=fc2733d6468c073f1dd738aa598ede55) | _ | hypothetical protein |
| [OCAR_1806](https://www.genoscope.cns.fr/agc/mage/wwwpkgdb/Info/getInfoLabel.php?id=3482000&wwwpkgdb=fc2733d6468c073f1dd738aa598ede55&nocache=a9a895ad49f0f55c362d050dc1ad3fa3&dir=&wwwpkgdb=fc2733d6468c073f1dd738aa598ede55) | _ | _ |
| [OCAR_5816](https://www.genoscope.cns.fr/agc/mage/wwwpkgdb/Info/getInfoLabel.php?id=3483168&wwwpkgdb=fc2733d6468c073f1dd738aa598ede55&nocache=a9a895ad49f0f55c362d050dc1ad3fa3&dir=&wwwpkgdb=fc2733d6468c073f1dd738aa598ede55) | _ | hypothetical protein |
| [OCAR_5819](https://www.genoscope.cns.fr/agc/mage/wwwpkgdb/Info/getInfoLabel.php?id=3484835&wwwpkgdb=fc2733d6468c073f1dd738aa598ede55&nocache=a9a895ad49f0f55c362d050dc1ad3fa3&dir=&wwwpkgdb=fc2733d6468c073f1dd738aa598ede55) | _ | hypothetical protein |
| [OCAR_1815](https://www.genoscope.cns.fr/agc/mage/wwwpkgdb/Info/getInfoLabel.php?id=3481999&wwwpkgdb=fc2733d6468c073f1dd738aa598ede55&nocache=a9a895ad49f0f55c362d050dc1ad3fa3&dir=&wwwpkgdb=fc2733d6468c073f1dd738aa598ede55) | _ | _ |
| [OCAR_5824](https://www.genoscope.cns.fr/agc/mage/wwwpkgdb/Info/getInfoLabel.php?id=3483172&wwwpkgdb=fc2733d6468c073f1dd738aa598ede55&nocache=a9a895ad49f0f55c362d050dc1ad3fa3&dir=&wwwpkgdb=fc2733d6468c073f1dd738aa598ede55) | _ | hypothetical protein |
| [OCAR_5823](https://www.genoscope.cns.fr/agc/mage/wwwpkgdb/Info/getInfoLabel.php?id=3484837&wwwpkgdb=fc2733d6468c073f1dd738aa598ede55&nocache=a9a895ad49f0f55c362d050dc1ad3fa3&dir=&wwwpkgdb=fc2733d6468c073f1dd738aa598ede55) | _ | hypothetical protein |
| [OCAR_1817](https://www.genoscope.cns.fr/agc/mage/wwwpkgdb/Info/getInfoLabel.php?id=3481998&wwwpkgdb=fc2733d6468c073f1dd738aa598ede55&nocache=a9a895ad49f0f55c362d050dc1ad3fa3&dir=&wwwpkgdb=fc2733d6468c073f1dd738aa598ede55) | _ | _ |
| [OCAR_5825](https://www.genoscope.cns.fr/agc/mage/wwwpkgdb/Info/getInfoLabel.php?id=3483173&wwwpkgdb=fc2733d6468c073f1dd738aa598ede55&nocache=a9a895ad49f0f55c362d050dc1ad3fa3&dir=&wwwpkgdb=fc2733d6468c073f1dd738aa598ede55) | _ | hypothetical protein |
| [OCAR_1819](https://www.genoscope.cns.fr/agc/mage/wwwpkgdb/Info/getInfoLabel.php?id=3481997&wwwpkgdb=fc2733d6468c073f1dd738aa598ede55&nocache=a9a895ad49f0f55c362d050dc1ad3fa3&dir=&wwwpkgdb=fc2733d6468c073f1dd738aa598ede55) | _ | _ |
| [OCAR_5828](https://www.genoscope.cns.fr/agc/mage/wwwpkgdb/Info/getInfoLabel.php?id=3484839&wwwpkgdb=fc2733d6468c073f1dd738aa598ede55&nocache=a9a895ad49f0f55c362d050dc1ad3fa3&dir=&wwwpkgdb=fc2733d6468c073f1dd738aa598ede55) | _ | putative hydrolase |
| [OCAR_5836](https://www.genoscope.cns.fr/agc/mage/wwwpkgdb/Info/getInfoLabel.php?id=3484843&wwwpkgdb=fc2733d6468c073f1dd738aa598ede55&nocache=a9a895ad49f0f55c362d050dc1ad3fa3&dir=&wwwpkgdb=fc2733d6468c073f1dd738aa598ede55) | _ | hypothetical protein |
| [OCAR_5845](https://www.genoscope.cns.fr/agc/mage/wwwpkgdb/Info/getInfoLabel.php?id=3483187&wwwpkgdb=fc2733d6468c073f1dd738aa598ede55&nocache=a9a895ad49f0f55c362d050dc1ad3fa3&dir=&wwwpkgdb=fc2733d6468c073f1dd738aa598ede55) | _ | hypothetical protein |
| [OCAR_5851](https://www.genoscope.cns.fr/agc/mage/wwwpkgdb/Info/getInfoLabel.php?id=3484845&wwwpkgdb=fc2733d6468c073f1dd738aa598ede55&nocache=a9a895ad49f0f55c362d050dc1ad3fa3&dir=&wwwpkgdb=fc2733d6468c073f1dd738aa598ede55) | _ | hypothetical protein |
| [OCAR_5852](https://www.genoscope.cns.fr/agc/mage/wwwpkgdb/Info/getInfoLabel.php?id=3483192&wwwpkgdb=fc2733d6468c073f1dd738aa598ede55&nocache=a9a895ad49f0f55c362d050dc1ad3fa3&dir=&wwwpkgdb=fc2733d6468c073f1dd738aa598ede55) | _ | hypothetical protein |
| [OCAR_1848](https://www.genoscope.cns.fr/agc/mage/wwwpkgdb/Info/getInfoLabel.php?id=3481996&wwwpkgdb=fc2733d6468c073f1dd738aa598ede55&nocache=a9a895ad49f0f55c362d050dc1ad3fa3&dir=&wwwpkgdb=fc2733d6468c073f1dd738aa598ede55) | _ | _ |
| [OCAR_5858](https://www.genoscope.cns.fr/agc/mage/wwwpkgdb/Info/getInfoLabel.php?id=3483195&wwwpkgdb=fc2733d6468c073f1dd738aa598ede55&nocache=a9a895ad49f0f55c362d050dc1ad3fa3&dir=&wwwpkgdb=fc2733d6468c073f1dd738aa598ede55) | _ | hypothetical protein |
| [OCAR_5859](https://www.genoscope.cns.fr/agc/mage/wwwpkgdb/Info/getInfoLabel.php?id=3483196&wwwpkgdb=fc2733d6468c073f1dd738aa598ede55&nocache=a9a895ad49f0f55c362d050dc1ad3fa3&dir=&wwwpkgdb=fc2733d6468c073f1dd738aa598ede55) | _ | putative lipoprotein |
| [OCAR_5868](https://www.genoscope.cns.fr/agc/mage/wwwpkgdb/Info/getInfoLabel.php?id=3483205&wwwpkgdb=fc2733d6468c073f1dd738aa598ede55&nocache=a9a895ad49f0f55c362d050dc1ad3fa3&dir=&wwwpkgdb=fc2733d6468c073f1dd738aa598ede55) | _ | hypothetical protein |
| [OCAR_5871](https://www.genoscope.cns.fr/agc/mage/wwwpkgdb/Info/getInfoLabel.php?id=3483206&wwwpkgdb=fc2733d6468c073f1dd738aa598ede55&nocache=a9a895ad49f0f55c362d050dc1ad3fa3&dir=&wwwpkgdb=fc2733d6468c073f1dd738aa598ede55) | _ | transcriptional regulator, XRE family with cupin sensor |
| [OCAR_5879](https://www.genoscope.cns.fr/agc/mage/wwwpkgdb/Info/getInfoLabel.php?id=3483213&wwwpkgdb=fc2733d6468c073f1dd738aa598ede55&nocache=a9a895ad49f0f55c362d050dc1ad3fa3&dir=&wwwpkgdb=fc2733d6468c073f1dd738aa598ede55) | _ | acyltransferase 3 |
| [OCAR_5882](https://www.genoscope.cns.fr/agc/mage/wwwpkgdb/Info/getInfoLabel.php?id=3484853&wwwpkgdb=fc2733d6468c073f1dd738aa598ede55&nocache=a9a895ad49f0f55c362d050dc1ad3fa3&dir=&wwwpkgdb=fc2733d6468c073f1dd738aa598ede55) | _ | dolichyl-phosphate-mannose-protein mannosyltransferase |
| [OCAR_5883](https://www.genoscope.cns.fr/agc/mage/wwwpkgdb/Info/getInfoLabel.php?id=3483215&wwwpkgdb=fc2733d6468c073f1dd738aa598ede55&nocache=a9a895ad49f0f55c362d050dc1ad3fa3&dir=&wwwpkgdb=fc2733d6468c073f1dd738aa598ede55) | _ | glycosyl transferase, family 9 |
| [OCAR_1881](https://www.genoscope.cns.fr/agc/mage/wwwpkgdb/Info/getInfoLabel.php?id=3481995&wwwpkgdb=fc2733d6468c073f1dd738aa598ede55&nocache=a9a895ad49f0f55c362d050dc1ad3fa3&dir=&wwwpkgdb=fc2733d6468c073f1dd738aa598ede55) | _ | _ |
| [OCAR_5891](https://www.genoscope.cns.fr/agc/mage/wwwpkgdb/Info/getInfoLabel.php?id=3483219&wwwpkgdb=fc2733d6468c073f1dd738aa598ede55&nocache=a9a895ad49f0f55c362d050dc1ad3fa3&dir=&wwwpkgdb=fc2733d6468c073f1dd738aa598ede55) | _ | iron permease FTR1 |
| [OCAR_5906](https://www.genoscope.cns.fr/agc/mage/wwwpkgdb/Info/getInfoLabel.php?id=3484864&wwwpkgdb=fc2733d6468c073f1dd738aa598ede55&nocache=a9a895ad49f0f55c362d050dc1ad3fa3&dir=&wwwpkgdb=fc2733d6468c073f1dd738aa598ede55) | _ | hypothetical protein |
| [OCAR_5910](https://www.genoscope.cns.fr/agc/mage/wwwpkgdb/Info/getInfoLabel.php?id=3484865&wwwpkgdb=fc2733d6468c073f1dd738aa598ede55&nocache=a9a895ad49f0f55c362d050dc1ad3fa3&dir=&wwwpkgdb=fc2733d6468c073f1dd738aa598ede55) | _ | hypothetical protein |
| [OCAR_1901](https://www.genoscope.cns.fr/agc/mage/wwwpkgdb/Info/getInfoLabel.php?id=3481994&wwwpkgdb=fc2733d6468c073f1dd738aa598ede55&nocache=a9a895ad49f0f55c362d050dc1ad3fa3&dir=&wwwpkgdb=fc2733d6468c073f1dd738aa598ede55) | _ | _ |
| [OCAR_5913](https://www.genoscope.cns.fr/agc/mage/wwwpkgdb/Info/getInfoLabel.php?id=3484866&wwwpkgdb=fc2733d6468c073f1dd738aa598ede55&nocache=a9a895ad49f0f55c362d050dc1ad3fa3&dir=&wwwpkgdb=fc2733d6468c073f1dd738aa598ede55) | _ | putative acyltransferase |
| [OCAR_5914](https://www.genoscope.cns.fr/agc/mage/wwwpkgdb/Info/getInfoLabel.php?id=3484867&wwwpkgdb=fc2733d6468c073f1dd738aa598ede55&nocache=a9a895ad49f0f55c362d050dc1ad3fa3&dir=&wwwpkgdb=fc2733d6468c073f1dd738aa598ede55) | _ | methyltransferase |
| [OCAR_1904](https://www.genoscope.cns.fr/agc/mage/wwwpkgdb/Info/getInfoLabel.php?id=3481993&wwwpkgdb=fc2733d6468c073f1dd738aa598ede55&nocache=a9a895ad49f0f55c362d050dc1ad3fa3&dir=&wwwpkgdb=fc2733d6468c073f1dd738aa598ede55) | _ | _ |
| [OCAR_5915](https://www.genoscope.cns.fr/agc/mage/wwwpkgdb/Info/getInfoLabel.php?id=3484868&wwwpkgdb=fc2733d6468c073f1dd738aa598ede55&nocache=a9a895ad49f0f55c362d050dc1ad3fa3&dir=&wwwpkgdb=fc2733d6468c073f1dd738aa598ede55) | _ | hypothetical protein |
| [OCAR_5941](https://www.genoscope.cns.fr/agc/mage/wwwpkgdb/Info/getInfoLabel.php?id=3483256&wwwpkgdb=fc2733d6468c073f1dd738aa598ede55&nocache=a9a895ad49f0f55c362d050dc1ad3fa3&dir=&wwwpkgdb=fc2733d6468c073f1dd738aa598ede55) | _ | hypothetical protein |
| [OCAR_5952](https://www.genoscope.cns.fr/agc/mage/wwwpkgdb/Info/getInfoLabel.php?id=3484873&wwwpkgdb=fc2733d6468c073f1dd738aa598ede55&nocache=a9a895ad49f0f55c362d050dc1ad3fa3&dir=&wwwpkgdb=fc2733d6468c073f1dd738aa598ede55) | _ | hypothetical protein |
| [OCAR_5964](https://www.genoscope.cns.fr/agc/mage/wwwpkgdb/Info/getInfoLabel.php?id=3483276&wwwpkgdb=fc2733d6468c073f1dd738aa598ede55&nocache=a9a895ad49f0f55c362d050dc1ad3fa3&dir=&wwwpkgdb=fc2733d6468c073f1dd738aa598ede55) | _ | hypothetical protein |
| [OCAR_1970](https://www.genoscope.cns.fr/agc/mage/wwwpkgdb/Info/getInfoLabel.php?id=3481991&wwwpkgdb=fc2733d6468c073f1dd738aa598ede55&nocache=a9a895ad49f0f55c362d050dc1ad3fa3&dir=&wwwpkgdb=fc2733d6468c073f1dd738aa598ede55) | _ | _ |
| [OCAR_1976](https://www.genoscope.cns.fr/agc/mage/wwwpkgdb/Info/getInfoLabel.php?id=3481990&wwwpkgdb=fc2733d6468c073f1dd738aa598ede55&nocache=a9a895ad49f0f55c362d050dc1ad3fa3&dir=&wwwpkgdb=fc2733d6468c073f1dd738aa598ede55) | _ | _ |
| [OCAR_5990](https://www.genoscope.cns.fr/agc/mage/wwwpkgdb/Info/getInfoLabel.php?id=3483288&wwwpkgdb=fc2733d6468c073f1dd738aa598ede55&nocache=a9a895ad49f0f55c362d050dc1ad3fa3&dir=&wwwpkgdb=fc2733d6468c073f1dd738aa598ede55) | _ | hypothetical protein |
| [OCAR_5993](https://www.genoscope.cns.fr/agc/mage/wwwpkgdb/Info/getInfoLabel.php?id=3484889&wwwpkgdb=fc2733d6468c073f1dd738aa598ede55&nocache=a9a895ad49f0f55c362d050dc1ad3fa3&dir=&wwwpkgdb=fc2733d6468c073f1dd738aa598ede55) | _ | hypothetical protein |
| [OCAR_6013](https://www.genoscope.cns.fr/agc/mage/wwwpkgdb/Info/getInfoLabel.php?id=3484897&wwwpkgdb=fc2733d6468c073f1dd738aa598ede55&nocache=a9a895ad49f0f55c362d050dc1ad3fa3&dir=&wwwpkgdb=fc2733d6468c073f1dd738aa598ede55) | _ | hypothetical protein |
| [OCAR_6014](https://www.genoscope.cns.fr/agc/mage/wwwpkgdb/Info/getInfoLabel.php?id=3483301&wwwpkgdb=fc2733d6468c073f1dd738aa598ede55&nocache=a9a895ad49f0f55c362d050dc1ad3fa3&dir=&wwwpkgdb=fc2733d6468c073f1dd738aa598ede55) | _ | hypothetical protein |
| [OCAR_6021](https://www.genoscope.cns.fr/agc/mage/wwwpkgdb/Info/getInfoLabel.php?id=3484899&wwwpkgdb=fc2733d6468c073f1dd738aa598ede55&nocache=a9a895ad49f0f55c362d050dc1ad3fa3&dir=&wwwpkgdb=fc2733d6468c073f1dd738aa598ede55) | speE | spermidine synthase |
| [OCAR_6022](https://www.genoscope.cns.fr/agc/mage/wwwpkgdb/Info/getInfoLabel.php?id=3484900&wwwpkgdb=fc2733d6468c073f1dd738aa598ede55&nocache=a9a895ad49f0f55c362d050dc1ad3fa3&dir=&wwwpkgdb=fc2733d6468c073f1dd738aa598ede55) | _ | S-adenosylmethionine decarboxylase related |
| [OCAR_6023](https://www.genoscope.cns.fr/agc/mage/wwwpkgdb/Info/getInfoLabel.php?id=3484901&wwwpkgdb=fc2733d6468c073f1dd738aa598ede55&nocache=a9a895ad49f0f55c362d050dc1ad3fa3&dir=&wwwpkgdb=fc2733d6468c073f1dd738aa598ede55) | _ | hypothetical protein |
| [OCAR_2012](https://www.genoscope.cns.fr/agc/mage/wwwpkgdb/Info/getInfoLabel.php?id=3481989&wwwpkgdb=fc2733d6468c073f1dd738aa598ede55&nocache=a9a895ad49f0f55c362d050dc1ad3fa3&dir=&wwwpkgdb=fc2733d6468c073f1dd738aa598ede55) | _ | _ |
| [OCAR_6030](https://www.genoscope.cns.fr/agc/mage/wwwpkgdb/Info/getInfoLabel.php?id=3483312&wwwpkgdb=fc2733d6468c073f1dd738aa598ede55&nocache=a9a895ad49f0f55c362d050dc1ad3fa3&dir=&wwwpkgdb=fc2733d6468c073f1dd738aa598ede55) | _ | hypothetical protein |
| [OCAR_6036](https://www.genoscope.cns.fr/agc/mage/wwwpkgdb/Info/getInfoLabel.php?id=3484905&wwwpkgdb=fc2733d6468c073f1dd738aa598ede55&nocache=a9a895ad49f0f55c362d050dc1ad3fa3&dir=&wwwpkgdb=fc2733d6468c073f1dd738aa598ede55) | _ | glycosyl transferase, group 1 |
| [OCAR_6037](https://www.genoscope.cns.fr/agc/mage/wwwpkgdb/Info/getInfoLabel.php?id=3484906&wwwpkgdb=fc2733d6468c073f1dd738aa598ede55&nocache=a9a895ad49f0f55c362d050dc1ad3fa3&dir=&wwwpkgdb=fc2733d6468c073f1dd738aa598ede55) | _ | putative lipoprotein |
| [OCAR_6038](https://www.genoscope.cns.fr/agc/mage/wwwpkgdb/Info/getInfoLabel.php?id=3483316&wwwpkgdb=fc2733d6468c073f1dd738aa598ede55&nocache=a9a895ad49f0f55c362d050dc1ad3fa3&dir=&wwwpkgdb=fc2733d6468c073f1dd738aa598ede55) | _ | vcbS |
| [OCAR_2026](https://www.genoscope.cns.fr/agc/mage/wwwpkgdb/Info/getInfoLabel.php?id=3481988&wwwpkgdb=fc2733d6468c073f1dd738aa598ede55&nocache=a9a895ad49f0f55c362d050dc1ad3fa3&dir=&wwwpkgdb=fc2733d6468c073f1dd738aa598ede55) | _ | _ |
| [OCAR_2034](https://www.genoscope.cns.fr/agc/mage/wwwpkgdb/Info/getInfoLabel.php?id=3481987&wwwpkgdb=fc2733d6468c073f1dd738aa598ede55&nocache=a9a895ad49f0f55c362d050dc1ad3fa3&dir=&wwwpkgdb=fc2733d6468c073f1dd738aa598ede55) | _ | _ |
| [OCAR_2035](https://www.genoscope.cns.fr/agc/mage/wwwpkgdb/Info/getInfoLabel.php?id=3481986&wwwpkgdb=fc2733d6468c073f1dd738aa598ede55&nocache=a9a895ad49f0f55c362d050dc1ad3fa3&dir=&wwwpkgdb=fc2733d6468c073f1dd738aa598ede55) | _ | _ |
| [OCAR_6048](https://www.genoscope.cns.fr/agc/mage/wwwpkgdb/Info/getInfoLabel.php?id=3483320&wwwpkgdb=fc2733d6468c073f1dd738aa598ede55&nocache=a9a895ad49f0f55c362d050dc1ad3fa3&dir=&wwwpkgdb=fc2733d6468c073f1dd738aa598ede55) | _ | O-methyltransferase I |
| [OCAR_6049](https://www.genoscope.cns.fr/agc/mage/wwwpkgdb/Info/getInfoLabel.php?id=3484913&wwwpkgdb=fc2733d6468c073f1dd738aa598ede55&nocache=a9a895ad49f0f55c362d050dc1ad3fa3&dir=&wwwpkgdb=fc2733d6468c073f1dd738aa598ede55) | _ | hypothetical protein |
| [OCAR_6050](https://www.genoscope.cns.fr/agc/mage/wwwpkgdb/Info/getInfoLabel.php?id=3484914&wwwpkgdb=fc2733d6468c073f1dd738aa598ede55&nocache=a9a895ad49f0f55c362d050dc1ad3fa3&dir=&wwwpkgdb=fc2733d6468c073f1dd738aa598ede55) | _ | hypothetical protein |
| [OCAR_6060](https://www.genoscope.cns.fr/agc/mage/wwwpkgdb/Info/getInfoLabel.php?id=3483329&wwwpkgdb=fc2733d6468c073f1dd738aa598ede55&nocache=a9a895ad49f0f55c362d050dc1ad3fa3&dir=&wwwpkgdb=fc2733d6468c073f1dd738aa598ede55) | _ | hypothetical protein |
| [OCAR_6071](https://www.genoscope.cns.fr/agc/mage/wwwpkgdb/Info/getInfoLabel.php?id=3483338&wwwpkgdb=fc2733d6468c073f1dd738aa598ede55&nocache=a9a895ad49f0f55c362d050dc1ad3fa3&dir=&wwwpkgdb=fc2733d6468c073f1dd738aa598ede55) | _ | hypothetical protein |
| [OCAR_6072](https://www.genoscope.cns.fr/agc/mage/wwwpkgdb/Info/getInfoLabel.php?id=3484918&wwwpkgdb=fc2733d6468c073f1dd738aa598ede55&nocache=a9a895ad49f0f55c362d050dc1ad3fa3&dir=&wwwpkgdb=fc2733d6468c073f1dd738aa598ede55) | _ | hypothetical protein |
| [OCAR_6083](https://www.genoscope.cns.fr/agc/mage/wwwpkgdb/Info/getInfoLabel.php?id=3484919&wwwpkgdb=fc2733d6468c073f1dd738aa598ede55&nocache=a9a895ad49f0f55c362d050dc1ad3fa3&dir=&wwwpkgdb=fc2733d6468c073f1dd738aa598ede55) | _ | hypothetical protein |
| [OCAR_6084](https://www.genoscope.cns.fr/agc/mage/wwwpkgdb/Info/getInfoLabel.php?id=3484920&wwwpkgdb=fc2733d6468c073f1dd738aa598ede55&nocache=a9a895ad49f0f55c362d050dc1ad3fa3&dir=&wwwpkgdb=fc2733d6468c073f1dd738aa598ede55) | _ | methyltransferase domain family |
| [OCAR_6085](https://www.genoscope.cns.fr/agc/mage/wwwpkgdb/Info/getInfoLabel.php?id=3484921&wwwpkgdb=fc2733d6468c073f1dd738aa598ede55&nocache=a9a895ad49f0f55c362d050dc1ad3fa3&dir=&wwwpkgdb=fc2733d6468c073f1dd738aa598ede55) | _ | methyl-accepting chemotaxis protein |
| [OCAR_6104](https://www.genoscope.cns.fr/agc/mage/wwwpkgdb/Info/getInfoLabel.php?id=3484926&wwwpkgdb=fc2733d6468c073f1dd738aa598ede55&nocache=a9a895ad49f0f55c362d050dc1ad3fa3&dir=&wwwpkgdb=fc2733d6468c073f1dd738aa598ede55) | _ | methyltransferase type 11 |
| [OCAR_6105](https://www.genoscope.cns.fr/agc/mage/wwwpkgdb/Info/getInfoLabel.php?id=3484927&wwwpkgdb=fc2733d6468c073f1dd738aa598ede55&nocache=a9a895ad49f0f55c362d050dc1ad3fa3&dir=&wwwpkgdb=fc2733d6468c073f1dd738aa598ede55) | _ | putative lipoprotein |
| [OCAR_6106](https://www.genoscope.cns.fr/agc/mage/wwwpkgdb/Info/getInfoLabel.php?id=3484928&wwwpkgdb=fc2733d6468c073f1dd738aa598ede55&nocache=a9a895ad49f0f55c362d050dc1ad3fa3&dir=&wwwpkgdb=fc2733d6468c073f1dd738aa598ede55) | _ | putative glycosyl transferase |
| [OCAR_6107](https://www.genoscope.cns.fr/agc/mage/wwwpkgdb/Info/getInfoLabel.php?id=3484929&wwwpkgdb=fc2733d6468c073f1dd738aa598ede55&nocache=a9a895ad49f0f55c362d050dc1ad3fa3&dir=&wwwpkgdb=fc2733d6468c073f1dd738aa598ede55) | _ | putative glycosyl transferase, group 1 |
| [OCAR_6108](https://www.genoscope.cns.fr/agc/mage/wwwpkgdb/Info/getInfoLabel.php?id=3484930&wwwpkgdb=fc2733d6468c073f1dd738aa598ede55&nocache=a9a895ad49f0f55c362d050dc1ad3fa3&dir=&wwwpkgdb=fc2733d6468c073f1dd738aa598ede55) | _ | EpsP |
| [OCAR_6109](https://www.genoscope.cns.fr/agc/mage/wwwpkgdb/Info/getInfoLabel.php?id=3483363&wwwpkgdb=fc2733d6468c073f1dd738aa598ede55&nocache=a9a895ad49f0f55c362d050dc1ad3fa3&dir=&wwwpkgdb=fc2733d6468c073f1dd738aa598ede55) | _ | glycosyl hydrolase family 10 |
| [OCAR_6110](https://www.genoscope.cns.fr/agc/mage/wwwpkgdb/Info/getInfoLabel.php?id=3484931&wwwpkgdb=fc2733d6468c073f1dd738aa598ede55&nocache=a9a895ad49f0f55c362d050dc1ad3fa3&dir=&wwwpkgdb=fc2733d6468c073f1dd738aa598ede55) | _ | putative polysaccharide biosynthesis protein |
| [OCAR_6111](https://www.genoscope.cns.fr/agc/mage/wwwpkgdb/Info/getInfoLabel.php?id=3484932&wwwpkgdb=fc2733d6468c073f1dd738aa598ede55&nocache=a9a895ad49f0f55c362d050dc1ad3fa3&dir=&wwwpkgdb=fc2733d6468c073f1dd738aa598ede55) | _ | putative acyltransferase |
| [OCAR_6112](https://www.genoscope.cns.fr/agc/mage/wwwpkgdb/Info/getInfoLabel.php?id=3484933&wwwpkgdb=fc2733d6468c073f1dd738aa598ede55&nocache=a9a895ad49f0f55c362d050dc1ad3fa3&dir=&wwwpkgdb=fc2733d6468c073f1dd738aa598ede55) | _ | putative mannosyltransferase |
| [OCAR_6113](https://www.genoscope.cns.fr/agc/mage/wwwpkgdb/Info/getInfoLabel.php?id=3484934&wwwpkgdb=fc2733d6468c073f1dd738aa598ede55&nocache=a9a895ad49f0f55c362d050dc1ad3fa3&dir=&wwwpkgdb=fc2733d6468c073f1dd738aa598ede55) | _ | hypothetical protein |
| [OCAR_6115](https://www.genoscope.cns.fr/agc/mage/wwwpkgdb/Info/getInfoLabel.php?id=3484936&wwwpkgdb=fc2733d6468c073f1dd738aa598ede55&nocache=a9a895ad49f0f55c362d050dc1ad3fa3&dir=&wwwpkgdb=fc2733d6468c073f1dd738aa598ede55) | _ | hypothetical protein |
| [OCAR_6118](https://www.genoscope.cns.fr/agc/mage/wwwpkgdb/Info/getInfoLabel.php?id=3484939&wwwpkgdb=fc2733d6468c073f1dd738aa598ede55&nocache=a9a895ad49f0f55c362d050dc1ad3fa3&dir=&wwwpkgdb=fc2733d6468c073f1dd738aa598ede55) | _ | hypothetical protein |
| [OCAR_2102](https://www.genoscope.cns.fr/agc/mage/wwwpkgdb/Info/getInfoLabel.php?id=3482129&wwwpkgdb=fc2733d6468c073f1dd738aa598ede55&nocache=a9a895ad49f0f55c362d050dc1ad3fa3&dir=&wwwpkgdb=fc2733d6468c073f1dd738aa598ede55) | _ | _ |
| [OCAR_6119](https://www.genoscope.cns.fr/agc/mage/wwwpkgdb/Info/getInfoLabel.php?id=3483364&wwwpkgdb=fc2733d6468c073f1dd738aa598ede55&nocache=a9a895ad49f0f55c362d050dc1ad3fa3&dir=&wwwpkgdb=fc2733d6468c073f1dd738aa598ede55) | _ | glucosyltransferase EpsB |
| [OCAR_6121](https://www.genoscope.cns.fr/agc/mage/wwwpkgdb/Info/getInfoLabel.php?id=3484940&wwwpkgdb=fc2733d6468c073f1dd738aa598ede55&nocache=a9a895ad49f0f55c362d050dc1ad3fa3&dir=&wwwpkgdb=fc2733d6468c073f1dd738aa598ede55) | _ | hypothetical protein |
| [OCAR_6130](https://www.genoscope.cns.fr/agc/mage/wwwpkgdb/Info/getInfoLabel.php?id=3483367&wwwpkgdb=fc2733d6468c073f1dd738aa598ede55&nocache=a9a895ad49f0f55c362d050dc1ad3fa3&dir=&wwwpkgdb=fc2733d6468c073f1dd738aa598ede55) | _ | hypothetical protein |
| [OCAR_6132](https://www.genoscope.cns.fr/agc/mage/wwwpkgdb/Info/getInfoLabel.php?id=3484948&wwwpkgdb=fc2733d6468c073f1dd738aa598ede55&nocache=a9a895ad49f0f55c362d050dc1ad3fa3&dir=&wwwpkgdb=fc2733d6468c073f1dd738aa598ede55) | _ | hypothetical protein |
| [OCAR_6133](https://www.genoscope.cns.fr/agc/mage/wwwpkgdb/Info/getInfoLabel.php?id=3484949&wwwpkgdb=fc2733d6468c073f1dd738aa598ede55&nocache=a9a895ad49f0f55c362d050dc1ad3fa3&dir=&wwwpkgdb=fc2733d6468c073f1dd738aa598ede55) | _ | hypothetical protein |
| [OCAR_2118](https://www.genoscope.cns.fr/agc/mage/wwwpkgdb/Info/getInfoLabel.php?id=3481984&wwwpkgdb=fc2733d6468c073f1dd738aa598ede55&nocache=a9a895ad49f0f55c362d050dc1ad3fa3&dir=&wwwpkgdb=fc2733d6468c073f1dd738aa598ede55) | _ | _ |
| [OCAR_6135](https://www.genoscope.cns.fr/agc/mage/wwwpkgdb/Info/getInfoLabel.php?id=3484951&wwwpkgdb=fc2733d6468c073f1dd738aa598ede55&nocache=a9a895ad49f0f55c362d050dc1ad3fa3&dir=&wwwpkgdb=fc2733d6468c073f1dd738aa598ede55) | _ | hypothetical protein |
| [OCAR_2123](https://www.genoscope.cns.fr/agc/mage/wwwpkgdb/Info/getInfoLabel.php?id=3481983&wwwpkgdb=fc2733d6468c073f1dd738aa598ede55&nocache=a9a895ad49f0f55c362d050dc1ad3fa3&dir=&wwwpkgdb=fc2733d6468c073f1dd738aa598ede55) | _ | _ |
| [OCAR_6139](https://www.genoscope.cns.fr/agc/mage/wwwpkgdb/Info/getInfoLabel.php?id=3484955&wwwpkgdb=fc2733d6468c073f1dd738aa598ede55&nocache=a9a895ad49f0f55c362d050dc1ad3fa3&dir=&wwwpkgdb=fc2733d6468c073f1dd738aa598ede55) | _ | hypothetical protein |
| [OCAR_2124](https://www.genoscope.cns.fr/agc/mage/wwwpkgdb/Info/getInfoLabel.php?id=3481982&wwwpkgdb=fc2733d6468c073f1dd738aa598ede55&nocache=a9a895ad49f0f55c362d050dc1ad3fa3&dir=&wwwpkgdb=fc2733d6468c073f1dd738aa598ede55) | _ | _ |
| [OCAR_6140](https://www.genoscope.cns.fr/agc/mage/wwwpkgdb/Info/getInfoLabel.php?id=3484956&wwwpkgdb=fc2733d6468c073f1dd738aa598ede55&nocache=a9a895ad49f0f55c362d050dc1ad3fa3&dir=&wwwpkgdb=fc2733d6468c073f1dd738aa598ede55) | _ | hypothetical protein |
| [OCAR_6141](https://www.genoscope.cns.fr/agc/mage/wwwpkgdb/Info/getInfoLabel.php?id=3484957&wwwpkgdb=fc2733d6468c073f1dd738aa598ede55&nocache=a9a895ad49f0f55c362d050dc1ad3fa3&dir=&wwwpkgdb=fc2733d6468c073f1dd738aa598ede55) | _ | hypothetical protein |
| [OCAR_6143](https://www.genoscope.cns.fr/agc/mage/wwwpkgdb/Info/getInfoLabel.php?id=3484959&wwwpkgdb=fc2733d6468c073f1dd738aa598ede55&nocache=a9a895ad49f0f55c362d050dc1ad3fa3&dir=&wwwpkgdb=fc2733d6468c073f1dd738aa598ede55) | _ | phage capsid family |
| [OCAR_6144](https://www.genoscope.cns.fr/agc/mage/wwwpkgdb/Info/getInfoLabel.php?id=3484960&wwwpkgdb=fc2733d6468c073f1dd738aa598ede55&nocache=a9a895ad49f0f55c362d050dc1ad3fa3&dir=&wwwpkgdb=fc2733d6468c073f1dd738aa598ede55) | _ | hypothetical protein |
| [OCAR_6145](https://www.genoscope.cns.fr/agc/mage/wwwpkgdb/Info/getInfoLabel.php?id=3484961&wwwpkgdb=fc2733d6468c073f1dd738aa598ede55&nocache=a9a895ad49f0f55c362d050dc1ad3fa3&dir=&wwwpkgdb=fc2733d6468c073f1dd738aa598ede55) | _ | hypothetical protein |
| [OCAR_6146](https://www.genoscope.cns.fr/agc/mage/wwwpkgdb/Info/getInfoLabel.php?id=3484962&wwwpkgdb=fc2733d6468c073f1dd738aa598ede55&nocache=a9a895ad49f0f55c362d050dc1ad3fa3&dir=&wwwpkgdb=fc2733d6468c073f1dd738aa598ede55) | _ | hypothetical protein |
| [OCAR_6147](https://www.genoscope.cns.fr/agc/mage/wwwpkgdb/Info/getInfoLabel.php?id=3484963&wwwpkgdb=fc2733d6468c073f1dd738aa598ede55&nocache=a9a895ad49f0f55c362d050dc1ad3fa3&dir=&wwwpkgdb=fc2733d6468c073f1dd738aa598ede55) | _ | RecA-family ATPase |
| [OCAR_6148](https://www.genoscope.cns.fr/agc/mage/wwwpkgdb/Info/getInfoLabel.php?id=3484964&wwwpkgdb=fc2733d6468c073f1dd738aa598ede55&nocache=a9a895ad49f0f55c362d050dc1ad3fa3&dir=&wwwpkgdb=fc2733d6468c073f1dd738aa598ede55) | _ | virulence-associated protein E |
| [OCAR_6149](https://www.genoscope.cns.fr/agc/mage/wwwpkgdb/Info/getInfoLabel.php?id=3484965&wwwpkgdb=fc2733d6468c073f1dd738aa598ede55&nocache=a9a895ad49f0f55c362d050dc1ad3fa3&dir=&wwwpkgdb=fc2733d6468c073f1dd738aa598ede55) | _ | hypothetical protein |
| [OCAR_6150](https://www.genoscope.cns.fr/agc/mage/wwwpkgdb/Info/getInfoLabel.php?id=3484966&wwwpkgdb=fc2733d6468c073f1dd738aa598ede55&nocache=a9a895ad49f0f55c362d050dc1ad3fa3&dir=&wwwpkgdb=fc2733d6468c073f1dd738aa598ede55) | _ | hypothetical protein |
| [OCAR_6152](https://www.genoscope.cns.fr/agc/mage/wwwpkgdb/Info/getInfoLabel.php?id=3484968&wwwpkgdb=fc2733d6468c073f1dd738aa598ede55&nocache=a9a895ad49f0f55c362d050dc1ad3fa3&dir=&wwwpkgdb=fc2733d6468c073f1dd738aa598ede55) | _ | hypothetical protein |
| [OCAR_6154](https://www.genoscope.cns.fr/agc/mage/wwwpkgdb/Info/getInfoLabel.php?id=3483370&wwwpkgdb=fc2733d6468c073f1dd738aa598ede55&nocache=a9a895ad49f0f55c362d050dc1ad3fa3&dir=&wwwpkgdb=fc2733d6468c073f1dd738aa598ede55) | _ | hypothetical protein |
| [OCAR_6155](https://www.genoscope.cns.fr/agc/mage/wwwpkgdb/Info/getInfoLabel.php?id=3483371&wwwpkgdb=fc2733d6468c073f1dd738aa598ede55&nocache=a9a895ad49f0f55c362d050dc1ad3fa3&dir=&wwwpkgdb=fc2733d6468c073f1dd738aa598ede55) | _ | hypothetical protein |
| [OCAR_6156](https://www.genoscope.cns.fr/agc/mage/wwwpkgdb/Info/getInfoLabel.php?id=3483372&wwwpkgdb=fc2733d6468c073f1dd738aa598ede55&nocache=a9a895ad49f0f55c362d050dc1ad3fa3&dir=&wwwpkgdb=fc2733d6468c073f1dd738aa598ede55) | _ | hypothetical protein |
| [OCAR_6162](https://www.genoscope.cns.fr/agc/mage/wwwpkgdb/Info/getInfoLabel.php?id=3484974&wwwpkgdb=fc2733d6468c073f1dd738aa598ede55&nocache=a9a895ad49f0f55c362d050dc1ad3fa3&dir=&wwwpkgdb=fc2733d6468c073f1dd738aa598ede55) | _ | hypothetical protein |
| [OCAR_6170](https://www.genoscope.cns.fr/agc/mage/wwwpkgdb/Info/getInfoLabel.php?id=3484982&wwwpkgdb=fc2733d6468c073f1dd738aa598ede55&nocache=a9a895ad49f0f55c362d050dc1ad3fa3&dir=&wwwpkgdb=fc2733d6468c073f1dd738aa598ede55) | _ | hypothetical protein |
| [OCAR_6175](https://www.genoscope.cns.fr/agc/mage/wwwpkgdb/Info/getInfoLabel.php?id=3483373&wwwpkgdb=fc2733d6468c073f1dd738aa598ede55&nocache=a9a895ad49f0f55c362d050dc1ad3fa3&dir=&wwwpkgdb=fc2733d6468c073f1dd738aa598ede55) | _ | hypothetical protein |
| [OCAR_6176](https://www.genoscope.cns.fr/agc/mage/wwwpkgdb/Info/getInfoLabel.php?id=3483374&wwwpkgdb=fc2733d6468c073f1dd738aa598ede55&nocache=a9a895ad49f0f55c362d050dc1ad3fa3&dir=&wwwpkgdb=fc2733d6468c073f1dd738aa598ede55) | _ | hypothetical protein |
| [OCAR_6179](https://www.genoscope.cns.fr/agc/mage/wwwpkgdb/Info/getInfoLabel.php?id=3483376&wwwpkgdb=fc2733d6468c073f1dd738aa598ede55&nocache=a9a895ad49f0f55c362d050dc1ad3fa3&dir=&wwwpkgdb=fc2733d6468c073f1dd738aa598ede55) | _ | hypothetical protein |
| [OCAR_6181](https://www.genoscope.cns.fr/agc/mage/wwwpkgdb/Info/getInfoLabel.php?id=3484988&wwwpkgdb=fc2733d6468c073f1dd738aa598ede55&nocache=a9a895ad49f0f55c362d050dc1ad3fa3&dir=&wwwpkgdb=fc2733d6468c073f1dd738aa598ede55) | _ | CumA |
| [OCAR_6184](https://www.genoscope.cns.fr/agc/mage/wwwpkgdb/Info/getInfoLabel.php?id=3484991&wwwpkgdb=fc2733d6468c073f1dd738aa598ede55&nocache=a9a895ad49f0f55c362d050dc1ad3fa3&dir=&wwwpkgdb=fc2733d6468c073f1dd738aa598ede55) | _ | extracellular solute-binding protein, family 1 |
| [OCAR_2170](https://www.genoscope.cns.fr/agc/mage/wwwpkgdb/Info/getInfoLabel.php?id=3481981&wwwpkgdb=fc2733d6468c073f1dd738aa598ede55&nocache=a9a895ad49f0f55c362d050dc1ad3fa3&dir=&wwwpkgdb=fc2733d6468c073f1dd738aa598ede55) | _ | _ |
| [OCAR_6190](https://www.genoscope.cns.fr/agc/mage/wwwpkgdb/Info/getInfoLabel.php?id=3484996&wwwpkgdb=fc2733d6468c073f1dd738aa598ede55&nocache=a9a895ad49f0f55c362d050dc1ad3fa3&dir=&wwwpkgdb=fc2733d6468c073f1dd738aa598ede55) | _ | metal-dependent hydrolase of the beta-lactamase superfamily I |
| [OCAR_6191](https://www.genoscope.cns.fr/agc/mage/wwwpkgdb/Info/getInfoLabel.php?id=3483379&wwwpkgdb=fc2733d6468c073f1dd738aa598ede55&nocache=a9a895ad49f0f55c362d050dc1ad3fa3&dir=&wwwpkgdb=fc2733d6468c073f1dd738aa598ede55) | _ | hypothetical protein |
| [OCAR_6212](https://www.genoscope.cns.fr/agc/mage/wwwpkgdb/Info/getInfoLabel.php?id=3485006&wwwpkgdb=fc2733d6468c073f1dd738aa598ede55&nocache=a9a895ad49f0f55c362d050dc1ad3fa3&dir=&wwwpkgdb=fc2733d6468c073f1dd738aa598ede55) | _ | hypothetical protein |
| [OCAR_6214](https://www.genoscope.cns.fr/agc/mage/wwwpkgdb/Info/getInfoLabel.php?id=3485007&wwwpkgdb=fc2733d6468c073f1dd738aa598ede55&nocache=a9a895ad49f0f55c362d050dc1ad3fa3&dir=&wwwpkgdb=fc2733d6468c073f1dd738aa598ede55) | _ | hypothetical protein |
| [OCAR_2204](https://www.genoscope.cns.fr/agc/mage/wwwpkgdb/Info/getInfoLabel.php?id=3481979&wwwpkgdb=fc2733d6468c073f1dd738aa598ede55&nocache=a9a895ad49f0f55c362d050dc1ad3fa3&dir=&wwwpkgdb=fc2733d6468c073f1dd738aa598ede55) | _ | _ |
| [OCAR_6227](https://www.genoscope.cns.fr/agc/mage/wwwpkgdb/Info/getInfoLabel.php?id=3483395&wwwpkgdb=fc2733d6468c073f1dd738aa598ede55&nocache=a9a895ad49f0f55c362d050dc1ad3fa3&dir=&wwwpkgdb=fc2733d6468c073f1dd738aa598ede55) | _ | hypothetical protein |
| [OCAR_6232](https://www.genoscope.cns.fr/agc/mage/wwwpkgdb/Info/getInfoLabel.php?id=3483396&wwwpkgdb=fc2733d6468c073f1dd738aa598ede55&nocache=a9a895ad49f0f55c362d050dc1ad3fa3&dir=&wwwpkgdb=fc2733d6468c073f1dd738aa598ede55) | _ | hypothetical protein |
| [OCAR_6236](https://www.genoscope.cns.fr/agc/mage/wwwpkgdb/Info/getInfoLabel.php?id=3483398&wwwpkgdb=fc2733d6468c073f1dd738aa598ede55&nocache=a9a895ad49f0f55c362d050dc1ad3fa3&dir=&wwwpkgdb=fc2733d6468c073f1dd738aa598ede55) | _ | hypothetical protein |
| [OCAR_6243](https://www.genoscope.cns.fr/agc/mage/wwwpkgdb/Info/getInfoLabel.php?id=3485025&wwwpkgdb=fc2733d6468c073f1dd738aa598ede55&nocache=a9a895ad49f0f55c362d050dc1ad3fa3&dir=&wwwpkgdb=fc2733d6468c073f1dd738aa598ede55) | _ | isochorismatase hydrolase |
| [OCAR_6245](https://www.genoscope.cns.fr/agc/mage/wwwpkgdb/Info/getInfoLabel.php?id=3483403&wwwpkgdb=fc2733d6468c073f1dd738aa598ede55&nocache=a9a895ad49f0f55c362d050dc1ad3fa3&dir=&wwwpkgdb=fc2733d6468c073f1dd738aa598ede55) | _ | gamma-aminobutyrate metabolism dehydratase/isomerase |
| [OCAR_6258](https://www.genoscope.cns.fr/agc/mage/wwwpkgdb/Info/getInfoLabel.php?id=3485034&wwwpkgdb=fc2733d6468c073f1dd738aa598ede55&nocache=a9a895ad49f0f55c362d050dc1ad3fa3&dir=&wwwpkgdb=fc2733d6468c073f1dd738aa598ede55) | _ | hypothetical protein |
| [OCAR_6259](https://www.genoscope.cns.fr/agc/mage/wwwpkgdb/Info/getInfoLabel.php?id=3483409&wwwpkgdb=fc2733d6468c073f1dd738aa598ede55&nocache=a9a895ad49f0f55c362d050dc1ad3fa3&dir=&wwwpkgdb=fc2733d6468c073f1dd738aa598ede55) | _ | hypothetical protein |
| [OCAR_6279](https://www.genoscope.cns.fr/agc/mage/wwwpkgdb/Info/getInfoLabel.php?id=3485051&wwwpkgdb=fc2733d6468c073f1dd738aa598ede55&nocache=a9a895ad49f0f55c362d050dc1ad3fa3&dir=&wwwpkgdb=fc2733d6468c073f1dd738aa598ede55) | _ | hypothetical protein |
| [OCAR_2261](https://www.genoscope.cns.fr/agc/mage/wwwpkgdb/Info/getInfoLabel.php?id=3481978&wwwpkgdb=fc2733d6468c073f1dd738aa598ede55&nocache=a9a895ad49f0f55c362d050dc1ad3fa3&dir=&wwwpkgdb=fc2733d6468c073f1dd738aa598ede55) | _ | _ |
| [OCAR_6283](https://www.genoscope.cns.fr/agc/mage/wwwpkgdb/Info/getInfoLabel.php?id=3485054&wwwpkgdb=fc2733d6468c073f1dd738aa598ede55&nocache=a9a895ad49f0f55c362d050dc1ad3fa3&dir=&wwwpkgdb=fc2733d6468c073f1dd738aa598ede55) | _ | putative lipoprotein |
| [OCAR_6289](https://www.genoscope.cns.fr/agc/mage/wwwpkgdb/Info/getInfoLabel.php?id=3483414&wwwpkgdb=fc2733d6468c073f1dd738aa598ede55&nocache=a9a895ad49f0f55c362d050dc1ad3fa3&dir=&wwwpkgdb=fc2733d6468c073f1dd738aa598ede55) | _ | hypothetical protein |
| [OCAR_6294](https://www.genoscope.cns.fr/agc/mage/wwwpkgdb/Info/getInfoLabel.php?id=3485063&wwwpkgdb=fc2733d6468c073f1dd738aa598ede55&nocache=a9a895ad49f0f55c362d050dc1ad3fa3&dir=&wwwpkgdb=fc2733d6468c073f1dd738aa598ede55) | _ | hypothetical protein |
| [OCAR_6295](https://www.genoscope.cns.fr/agc/mage/wwwpkgdb/Info/getInfoLabel.php?id=3485064&wwwpkgdb=fc2733d6468c073f1dd738aa598ede55&nocache=a9a895ad49f0f55c362d050dc1ad3fa3&dir=&wwwpkgdb=fc2733d6468c073f1dd738aa598ede55) | _ | hypothetical protein |
| [OCAR_6297](https://www.genoscope.cns.fr/agc/mage/wwwpkgdb/Info/getInfoLabel.php?id=3485066&wwwpkgdb=fc2733d6468c073f1dd738aa598ede55&nocache=a9a895ad49f0f55c362d050dc1ad3fa3&dir=&wwwpkgdb=fc2733d6468c073f1dd738aa598ede55) | _ | hypothetical protein |
| [OCAR_6299](https://www.genoscope.cns.fr/agc/mage/wwwpkgdb/Info/getInfoLabel.php?id=3485068&wwwpkgdb=fc2733d6468c073f1dd738aa598ede55&nocache=a9a895ad49f0f55c362d050dc1ad3fa3&dir=&wwwpkgdb=fc2733d6468c073f1dd738aa598ede55) | _ | hypothetical protein |
| [OCAR_6308](https://www.genoscope.cns.fr/agc/mage/wwwpkgdb/Info/getInfoLabel.php?id=3485072&wwwpkgdb=fc2733d6468c073f1dd738aa598ede55&nocache=a9a895ad49f0f55c362d050dc1ad3fa3&dir=&wwwpkgdb=fc2733d6468c073f1dd738aa598ede55) | _ | hypothetical protein |
| [OCAR_6309](https://www.genoscope.cns.fr/agc/mage/wwwpkgdb/Info/getInfoLabel.php?id=3485073&wwwpkgdb=fc2733d6468c073f1dd738aa598ede55&nocache=a9a895ad49f0f55c362d050dc1ad3fa3&dir=&wwwpkgdb=fc2733d6468c073f1dd738aa598ede55) | _ | hypothetical protein |
| [OCAR_6317](https://www.genoscope.cns.fr/agc/mage/wwwpkgdb/Info/getInfoLabel.php?id=3483424&wwwpkgdb=fc2733d6468c073f1dd738aa598ede55&nocache=a9a895ad49f0f55c362d050dc1ad3fa3&dir=&wwwpkgdb=fc2733d6468c073f1dd738aa598ede55) | _ | hypothetical protein |
| [OCAR_6325](https://www.genoscope.cns.fr/agc/mage/wwwpkgdb/Info/getInfoLabel.php?id=3483427&wwwpkgdb=fc2733d6468c073f1dd738aa598ede55&nocache=a9a895ad49f0f55c362d050dc1ad3fa3&dir=&wwwpkgdb=fc2733d6468c073f1dd738aa598ede55) | _ | hypothetical protein |
| [OCAR_6328](https://www.genoscope.cns.fr/agc/mage/wwwpkgdb/Info/getInfoLabel.php?id=3485084&wwwpkgdb=fc2733d6468c073f1dd738aa598ede55&nocache=a9a895ad49f0f55c362d050dc1ad3fa3&dir=&wwwpkgdb=fc2733d6468c073f1dd738aa598ede55) | _ | hypothetical protein |
| [OCAR_6335](https://www.genoscope.cns.fr/agc/mage/wwwpkgdb/Info/getInfoLabel.php?id=3483432&wwwpkgdb=fc2733d6468c073f1dd738aa598ede55&nocache=a9a895ad49f0f55c362d050dc1ad3fa3&dir=&wwwpkgdb=fc2733d6468c073f1dd738aa598ede55) | _ | hypothetical protein |
| [OCAR_6336](https://www.genoscope.cns.fr/agc/mage/wwwpkgdb/Info/getInfoLabel.php?id=3485088&wwwpkgdb=fc2733d6468c073f1dd738aa598ede55&nocache=a9a895ad49f0f55c362d050dc1ad3fa3&dir=&wwwpkgdb=fc2733d6468c073f1dd738aa598ede55) | _ | hypothetical protein |
| [OCAR_6342](https://www.genoscope.cns.fr/agc/mage/wwwpkgdb/Info/getInfoLabel.php?id=3485093&wwwpkgdb=fc2733d6468c073f1dd738aa598ede55&nocache=a9a895ad49f0f55c362d050dc1ad3fa3&dir=&wwwpkgdb=fc2733d6468c073f1dd738aa598ede55) | _ | hypothetical protein |
| [OCAR_2321](https://www.genoscope.cns.fr/agc/mage/wwwpkgdb/Info/getInfoLabel.php?id=3481977&wwwpkgdb=fc2733d6468c073f1dd738aa598ede55&nocache=a9a895ad49f0f55c362d050dc1ad3fa3&dir=&wwwpkgdb=fc2733d6468c073f1dd738aa598ede55) | _ | _ |
| [OCAR_6348](https://www.genoscope.cns.fr/agc/mage/wwwpkgdb/Info/getInfoLabel.php?id=3483437&wwwpkgdb=fc2733d6468c073f1dd738aa598ede55&nocache=a9a895ad49f0f55c362d050dc1ad3fa3&dir=&wwwpkgdb=fc2733d6468c073f1dd738aa598ede55) | _ | hypothetical protein |
| [OCAR_6353](https://www.genoscope.cns.fr/agc/mage/wwwpkgdb/Info/getInfoLabel.php?id=3483439&wwwpkgdb=fc2733d6468c073f1dd738aa598ede55&nocache=a9a895ad49f0f55c362d050dc1ad3fa3&dir=&wwwpkgdb=fc2733d6468c073f1dd738aa598ede55) | _ | hypothetical protein |
| [OCAR_6360](https://www.genoscope.cns.fr/agc/mage/wwwpkgdb/Info/getInfoLabel.php?id=3483446&wwwpkgdb=fc2733d6468c073f1dd738aa598ede55&nocache=a9a895ad49f0f55c362d050dc1ad3fa3&dir=&wwwpkgdb=fc2733d6468c073f1dd738aa598ede55) | _ | hypothetical protein |
| [OCAR_6361](https://www.genoscope.cns.fr/agc/mage/wwwpkgdb/Info/getInfoLabel.php?id=3485099&wwwpkgdb=fc2733d6468c073f1dd738aa598ede55&nocache=a9a895ad49f0f55c362d050dc1ad3fa3&dir=&wwwpkgdb=fc2733d6468c073f1dd738aa598ede55) | _ | 2-Hydroxychromene-2-carboxylate isomerase |
| [OCAR_6368](https://www.genoscope.cns.fr/agc/mage/wwwpkgdb/Info/getInfoLabel.php?id=3483447&wwwpkgdb=fc2733d6468c073f1dd738aa598ede55&nocache=a9a895ad49f0f55c362d050dc1ad3fa3&dir=&wwwpkgdb=fc2733d6468c073f1dd738aa598ede55) | _ | hypothetical protein |
| [OCAR_6380](https://www.genoscope.cns.fr/agc/mage/wwwpkgdb/Info/getInfoLabel.php?id=3485109&wwwpkgdb=fc2733d6468c073f1dd738aa598ede55&nocache=a9a895ad49f0f55c362d050dc1ad3fa3&dir=&wwwpkgdb=fc2733d6468c073f1dd738aa598ede55) | _ | hypothetical protein |
| [OCAR_2370](https://www.genoscope.cns.fr/agc/mage/wwwpkgdb/Info/getInfoLabel.php?id=3481976&wwwpkgdb=fc2733d6468c073f1dd738aa598ede55&nocache=a9a895ad49f0f55c362d050dc1ad3fa3&dir=&wwwpkgdb=fc2733d6468c073f1dd738aa598ede55) | _ | _ |
| [OCAR_6396](https://www.genoscope.cns.fr/agc/mage/wwwpkgdb/Info/getInfoLabel.php?id=3485121&wwwpkgdb=fc2733d6468c073f1dd738aa598ede55&nocache=a9a895ad49f0f55c362d050dc1ad3fa3&dir=&wwwpkgdb=fc2733d6468c073f1dd738aa598ede55) | _ | hypothetical protein |
| [OCAR_2375](https://www.genoscope.cns.fr/agc/mage/wwwpkgdb/Info/getInfoLabel.php?id=3481975&wwwpkgdb=fc2733d6468c073f1dd738aa598ede55&nocache=a9a895ad49f0f55c362d050dc1ad3fa3&dir=&wwwpkgdb=fc2733d6468c073f1dd738aa598ede55) | _ | _ |
| [OCAR_6400](https://www.genoscope.cns.fr/agc/mage/wwwpkgdb/Info/getInfoLabel.php?id=3483461&wwwpkgdb=fc2733d6468c073f1dd738aa598ede55&nocache=a9a895ad49f0f55c362d050dc1ad3fa3&dir=&wwwpkgdb=fc2733d6468c073f1dd738aa598ede55) | _ | voltage-gated chloride channel |
| [OCAR_6401](https://www.genoscope.cns.fr/agc/mage/wwwpkgdb/Info/getInfoLabel.php?id=3483462&wwwpkgdb=fc2733d6468c073f1dd738aa598ede55&nocache=a9a895ad49f0f55c362d050dc1ad3fa3&dir=&wwwpkgdb=fc2733d6468c073f1dd738aa598ede55) | _ | hypothetical protein |
| [OCAR_6402](https://www.genoscope.cns.fr/agc/mage/wwwpkgdb/Info/getInfoLabel.php?id=3485124&wwwpkgdb=fc2733d6468c073f1dd738aa598ede55&nocache=a9a895ad49f0f55c362d050dc1ad3fa3&dir=&wwwpkgdb=fc2733d6468c073f1dd738aa598ede55) | _ | hypothetical protein |
| [OCAR_6403](https://www.genoscope.cns.fr/agc/mage/wwwpkgdb/Info/getInfoLabel.php?id=3485125&wwwpkgdb=fc2733d6468c073f1dd738aa598ede55&nocache=a9a895ad49f0f55c362d050dc1ad3fa3&dir=&wwwpkgdb=fc2733d6468c073f1dd738aa598ede55) | _ | hypothetical protein |
| [OCAR_6404](https://www.genoscope.cns.fr/agc/mage/wwwpkgdb/Info/getInfoLabel.php?id=3485126&wwwpkgdb=fc2733d6468c073f1dd738aa598ede55&nocache=a9a895ad49f0f55c362d050dc1ad3fa3&dir=&wwwpkgdb=fc2733d6468c073f1dd738aa598ede55) | _ | hypothetical protein |
| [OCAR_6405](https://www.genoscope.cns.fr/agc/mage/wwwpkgdb/Info/getInfoLabel.php?id=3485127&wwwpkgdb=fc2733d6468c073f1dd738aa598ede55&nocache=a9a895ad49f0f55c362d050dc1ad3fa3&dir=&wwwpkgdb=fc2733d6468c073f1dd738aa598ede55) | _ | hypothetical protein |
| [OCAR_6406](https://www.genoscope.cns.fr/agc/mage/wwwpkgdb/Info/getInfoLabel.php?id=3485128&wwwpkgdb=fc2733d6468c073f1dd738aa598ede55&nocache=a9a895ad49f0f55c362d050dc1ad3fa3&dir=&wwwpkgdb=fc2733d6468c073f1dd738aa598ede55) | _ | hypothetical protein |
| [OCAR_6407](https://www.genoscope.cns.fr/agc/mage/wwwpkgdb/Info/getInfoLabel.php?id=3485129&wwwpkgdb=fc2733d6468c073f1dd738aa598ede55&nocache=a9a895ad49f0f55c362d050dc1ad3fa3&dir=&wwwpkgdb=fc2733d6468c073f1dd738aa598ede55) | _ | exonuclease, RNase T and DNA polymerase III |
| [OCAR_6408](https://www.genoscope.cns.fr/agc/mage/wwwpkgdb/Info/getInfoLabel.php?id=3485130&wwwpkgdb=fc2733d6468c073f1dd738aa598ede55&nocache=a9a895ad49f0f55c362d050dc1ad3fa3&dir=&wwwpkgdb=fc2733d6468c073f1dd738aa598ede55) | _ | hypothetical protein |
| [OCAR_6409](https://www.genoscope.cns.fr/agc/mage/wwwpkgdb/Info/getInfoLabel.php?id=3485131&wwwpkgdb=fc2733d6468c073f1dd738aa598ede55&nocache=a9a895ad49f0f55c362d050dc1ad3fa3&dir=&wwwpkgdb=fc2733d6468c073f1dd738aa598ede55) | _ | AAA ATPase, central domain protein |
| [OCAR_6410](https://www.genoscope.cns.fr/agc/mage/wwwpkgdb/Info/getInfoLabel.php?id=3485132&wwwpkgdb=fc2733d6468c073f1dd738aa598ede55&nocache=a9a895ad49f0f55c362d050dc1ad3fa3&dir=&wwwpkgdb=fc2733d6468c073f1dd738aa598ede55) | _ | hypothetical protein |
| [OCAR_6411](https://www.genoscope.cns.fr/agc/mage/wwwpkgdb/Info/getInfoLabel.php?id=3485133&wwwpkgdb=fc2733d6468c073f1dd738aa598ede55&nocache=a9a895ad49f0f55c362d050dc1ad3fa3&dir=&wwwpkgdb=fc2733d6468c073f1dd738aa598ede55) | _ | hypothetical protein |
| [OCAR_6412](https://www.genoscope.cns.fr/agc/mage/wwwpkgdb/Info/getInfoLabel.php?id=3483463&wwwpkgdb=fc2733d6468c073f1dd738aa598ede55&nocache=a9a895ad49f0f55c362d050dc1ad3fa3&dir=&wwwpkgdb=fc2733d6468c073f1dd738aa598ede55) | _ | hypothetical protein |
| [OCAR_6413](https://www.genoscope.cns.fr/agc/mage/wwwpkgdb/Info/getInfoLabel.php?id=3483464&wwwpkgdb=fc2733d6468c073f1dd738aa598ede55&nocache=a9a895ad49f0f55c362d050dc1ad3fa3&dir=&wwwpkgdb=fc2733d6468c073f1dd738aa598ede55) | _ | helix-turn-helix domain protein |
| [OCAR_6414](https://www.genoscope.cns.fr/agc/mage/wwwpkgdb/Info/getInfoLabel.php?id=3483465&wwwpkgdb=fc2733d6468c073f1dd738aa598ede55&nocache=a9a895ad49f0f55c362d050dc1ad3fa3&dir=&wwwpkgdb=fc2733d6468c073f1dd738aa598ede55) | _ | hypothetical protein |
| [OCAR_6415](https://www.genoscope.cns.fr/agc/mage/wwwpkgdb/Info/getInfoLabel.php?id=3483466&wwwpkgdb=fc2733d6468c073f1dd738aa598ede55&nocache=a9a895ad49f0f55c362d050dc1ad3fa3&dir=&wwwpkgdb=fc2733d6468c073f1dd738aa598ede55) | _ | hypothetical protein |
| [OCAR_6416](https://www.genoscope.cns.fr/agc/mage/wwwpkgdb/Info/getInfoLabel.php?id=3483467&wwwpkgdb=fc2733d6468c073f1dd738aa598ede55&nocache=a9a895ad49f0f55c362d050dc1ad3fa3&dir=&wwwpkgdb=fc2733d6468c073f1dd738aa598ede55) | _ | hypothetical protein |
| [OCAR_6417](https://www.genoscope.cns.fr/agc/mage/wwwpkgdb/Info/getInfoLabel.php?id=3485134&wwwpkgdb=fc2733d6468c073f1dd738aa598ede55&nocache=a9a895ad49f0f55c362d050dc1ad3fa3&dir=&wwwpkgdb=fc2733d6468c073f1dd738aa598ede55) | _ | hypothetical protein |
| [OCAR_6418](https://www.genoscope.cns.fr/agc/mage/wwwpkgdb/Info/getInfoLabel.php?id=3485135&wwwpkgdb=fc2733d6468c073f1dd738aa598ede55&nocache=a9a895ad49f0f55c362d050dc1ad3fa3&dir=&wwwpkgdb=fc2733d6468c073f1dd738aa598ede55) | _ | Y4BN |
| [OCAR_6420](https://www.genoscope.cns.fr/agc/mage/wwwpkgdb/Info/getInfoLabel.php?id=3483468&wwwpkgdb=fc2733d6468c073f1dd738aa598ede55&nocache=a9a895ad49f0f55c362d050dc1ad3fa3&dir=&wwwpkgdb=fc2733d6468c073f1dd738aa598ede55) | _ | hypothetical protein |
| [OCAR_6433](https://www.genoscope.cns.fr/agc/mage/wwwpkgdb/Info/getInfoLabel.php?id=3485149&wwwpkgdb=fc2733d6468c073f1dd738aa598ede55&nocache=a9a895ad49f0f55c362d050dc1ad3fa3&dir=&wwwpkgdb=fc2733d6468c073f1dd738aa598ede55) | _ | putative membrane protein of unknown function |
| [OCAR_6434](https://www.genoscope.cns.fr/agc/mage/wwwpkgdb/Info/getInfoLabel.php?id=3485150&wwwpkgdb=fc2733d6468c073f1dd738aa598ede55&nocache=a9a895ad49f0f55c362d050dc1ad3fa3&dir=&wwwpkgdb=fc2733d6468c073f1dd738aa598ede55) | _ | hypothetical protein |
| [OCAR_6445](https://www.genoscope.cns.fr/agc/mage/wwwpkgdb/Info/getInfoLabel.php?id=3483469&wwwpkgdb=fc2733d6468c073f1dd738aa598ede55&nocache=a9a895ad49f0f55c362d050dc1ad3fa3&dir=&wwwpkgdb=fc2733d6468c073f1dd738aa598ede55) | _ | hypothetical protein |
| [OCAR_6446](https://www.genoscope.cns.fr/agc/mage/wwwpkgdb/Info/getInfoLabel.php?id=3485161&wwwpkgdb=fc2733d6468c073f1dd738aa598ede55&nocache=a9a895ad49f0f55c362d050dc1ad3fa3&dir=&wwwpkgdb=fc2733d6468c073f1dd738aa598ede55) | _ | hypothetical protein |
| [OCAR_6450](https://www.genoscope.cns.fr/agc/mage/wwwpkgdb/Info/getInfoLabel.php?id=3485165&wwwpkgdb=fc2733d6468c073f1dd738aa598ede55&nocache=a9a895ad49f0f55c362d050dc1ad3fa3&dir=&wwwpkgdb=fc2733d6468c073f1dd738aa598ede55) | _ | DNA methylase |
| [OCAR_2429](https://www.genoscope.cns.fr/agc/mage/wwwpkgdb/Info/getInfoLabel.php?id=3481972&wwwpkgdb=fc2733d6468c073f1dd738aa598ede55&nocache=a9a895ad49f0f55c362d050dc1ad3fa3&dir=&wwwpkgdb=fc2733d6468c073f1dd738aa598ede55) | _ | _ |
| [OCAR_6457](https://www.genoscope.cns.fr/agc/mage/wwwpkgdb/Info/getInfoLabel.php?id=3483471&wwwpkgdb=fc2733d6468c073f1dd738aa598ede55&nocache=a9a895ad49f0f55c362d050dc1ad3fa3&dir=&wwwpkgdb=fc2733d6468c073f1dd738aa598ede55) | _ | hypothetical protein |
| [OCAR_6459](https://www.genoscope.cns.fr/agc/mage/wwwpkgdb/Info/getInfoLabel.php?id=3485172&wwwpkgdb=fc2733d6468c073f1dd738aa598ede55&nocache=a9a895ad49f0f55c362d050dc1ad3fa3&dir=&wwwpkgdb=fc2733d6468c073f1dd738aa598ede55) | _ | hypothetical protein |
| [OCAR_6460](https://www.genoscope.cns.fr/agc/mage/wwwpkgdb/Info/getInfoLabel.php?id=3485173&wwwpkgdb=fc2733d6468c073f1dd738aa598ede55&nocache=a9a895ad49f0f55c362d050dc1ad3fa3&dir=&wwwpkgdb=fc2733d6468c073f1dd738aa598ede55) | _ | hypothetical protein |
| [OCAR_6461](https://www.genoscope.cns.fr/agc/mage/wwwpkgdb/Info/getInfoLabel.php?id=3485174&wwwpkgdb=fc2733d6468c073f1dd738aa598ede55&nocache=a9a895ad49f0f55c362d050dc1ad3fa3&dir=&wwwpkgdb=fc2733d6468c073f1dd738aa598ede55) | _ | hypothetical protein |
| [OCAR_6462](https://www.genoscope.cns.fr/agc/mage/wwwpkgdb/Info/getInfoLabel.php?id=3483472&wwwpkgdb=fc2733d6468c073f1dd738aa598ede55&nocache=a9a895ad49f0f55c362d050dc1ad3fa3&dir=&wwwpkgdb=fc2733d6468c073f1dd738aa598ede55) | _ | hypothetical protein |
| [OCAR_6464](https://www.genoscope.cns.fr/agc/mage/wwwpkgdb/Info/getInfoLabel.php?id=3483473&wwwpkgdb=fc2733d6468c073f1dd738aa598ede55&nocache=a9a895ad49f0f55c362d050dc1ad3fa3&dir=&wwwpkgdb=fc2733d6468c073f1dd738aa598ede55) | _ | hypothetical protein |
| [OCAR_2441](https://www.genoscope.cns.fr/agc/mage/wwwpkgdb/Info/getInfoLabel.php?id=3481971&wwwpkgdb=fc2733d6468c073f1dd738aa598ede55&nocache=a9a895ad49f0f55c362d050dc1ad3fa3&dir=&wwwpkgdb=fc2733d6468c073f1dd738aa598ede55) | _ | _ |
| [OCAR_6466](https://www.genoscope.cns.fr/agc/mage/wwwpkgdb/Info/getInfoLabel.php?id=3483474&wwwpkgdb=fc2733d6468c073f1dd738aa598ede55&nocache=a9a895ad49f0f55c362d050dc1ad3fa3&dir=&wwwpkgdb=fc2733d6468c073f1dd738aa598ede55) | _ | hypothetical protein |
| [OCAR_2444](https://www.genoscope.cns.fr/agc/mage/wwwpkgdb/Info/getInfoLabel.php?id=3481970&wwwpkgdb=fc2733d6468c073f1dd738aa598ede55&nocache=a9a895ad49f0f55c362d050dc1ad3fa3&dir=&wwwpkgdb=fc2733d6468c073f1dd738aa598ede55) | _ | _ |
| [OCAR_6474](https://www.genoscope.cns.fr/agc/mage/wwwpkgdb/Info/getInfoLabel.php?id=3483476&wwwpkgdb=fc2733d6468c073f1dd738aa598ede55&nocache=a9a895ad49f0f55c362d050dc1ad3fa3&dir=&wwwpkgdb=fc2733d6468c073f1dd738aa598ede55) | _ | hypothetical protein |
| [OCAR_2461](https://www.genoscope.cns.fr/agc/mage/wwwpkgdb/Info/getInfoLabel.php?id=3481969&wwwpkgdb=fc2733d6468c073f1dd738aa598ede55&nocache=a9a895ad49f0f55c362d050dc1ad3fa3&dir=&wwwpkgdb=fc2733d6468c073f1dd738aa598ede55) | _ | _ |
| [OCAR_6488](https://www.genoscope.cns.fr/agc/mage/wwwpkgdb/Info/getInfoLabel.php?id=3485189&wwwpkgdb=fc2733d6468c073f1dd738aa598ede55&nocache=a9a895ad49f0f55c362d050dc1ad3fa3&dir=&wwwpkgdb=fc2733d6468c073f1dd738aa598ede55) | _ | hypothetical protein |
| [OCAR_6491](https://www.genoscope.cns.fr/agc/mage/wwwpkgdb/Info/getInfoLabel.php?id=3485192&wwwpkgdb=fc2733d6468c073f1dd738aa598ede55&nocache=a9a895ad49f0f55c362d050dc1ad3fa3&dir=&wwwpkgdb=fc2733d6468c073f1dd738aa598ede55) | _ | hypothetical protein |
| [OCAR_6492](https://www.genoscope.cns.fr/agc/mage/wwwpkgdb/Info/getInfoLabel.php?id=3485193&wwwpkgdb=fc2733d6468c073f1dd738aa598ede55&nocache=a9a895ad49f0f55c362d050dc1ad3fa3&dir=&wwwpkgdb=fc2733d6468c073f1dd738aa598ede55) | _ | hypothetical protein |
| [OCAR_6493](https://www.genoscope.cns.fr/agc/mage/wwwpkgdb/Info/getInfoLabel.php?id=3485194&wwwpkgdb=fc2733d6468c073f1dd738aa598ede55&nocache=a9a895ad49f0f55c362d050dc1ad3fa3&dir=&wwwpkgdb=fc2733d6468c073f1dd738aa598ede55) | _ | hypothetical protein |
| [OCAR_2473](https://www.genoscope.cns.fr/agc/mage/wwwpkgdb/Info/getInfoLabel.php?id=3481968&wwwpkgdb=fc2733d6468c073f1dd738aa598ede55&nocache=a9a895ad49f0f55c362d050dc1ad3fa3&dir=&wwwpkgdb=fc2733d6468c073f1dd738aa598ede55) | _ | _ |
| [OCAR_6510](https://www.genoscope.cns.fr/agc/mage/wwwpkgdb/Info/getInfoLabel.php?id=3485202&wwwpkgdb=fc2733d6468c073f1dd738aa598ede55&nocache=a9a895ad49f0f55c362d050dc1ad3fa3&dir=&wwwpkgdb=fc2733d6468c073f1dd738aa598ede55) | _ | hypothetical protein |
| [OCAR_6513](https://www.genoscope.cns.fr/agc/mage/wwwpkgdb/Info/getInfoLabel.php?id=3485205&wwwpkgdb=fc2733d6468c073f1dd738aa598ede55&nocache=a9a895ad49f0f55c362d050dc1ad3fa3&dir=&wwwpkgdb=fc2733d6468c073f1dd738aa598ede55) | _ | hypothetical protein |
| [OCAR_2494](https://www.genoscope.cns.fr/agc/mage/wwwpkgdb/Info/getInfoLabel.php?id=3481967&wwwpkgdb=fc2733d6468c073f1dd738aa598ede55&nocache=a9a895ad49f0f55c362d050dc1ad3fa3&dir=&wwwpkgdb=fc2733d6468c073f1dd738aa598ede55) | _ | _ |
| [OCAR_2498](https://www.genoscope.cns.fr/agc/mage/wwwpkgdb/Info/getInfoLabel.php?id=3481966&wwwpkgdb=fc2733d6468c073f1dd738aa598ede55&nocache=a9a895ad49f0f55c362d050dc1ad3fa3&dir=&wwwpkgdb=fc2733d6468c073f1dd738aa598ede55) | _ | _ |
| [OCAR_2501](https://www.genoscope.cns.fr/agc/mage/wwwpkgdb/Info/getInfoLabel.php?id=3481965&wwwpkgdb=fc2733d6468c073f1dd738aa598ede55&nocache=a9a895ad49f0f55c362d050dc1ad3fa3&dir=&wwwpkgdb=fc2733d6468c073f1dd738aa598ede55) | _ | _ |
| [OCAR_6524](https://www.genoscope.cns.fr/agc/mage/wwwpkgdb/Info/getInfoLabel.php?id=3485213&wwwpkgdb=fc2733d6468c073f1dd738aa598ede55&nocache=a9a895ad49f0f55c362d050dc1ad3fa3&dir=&wwwpkgdb=fc2733d6468c073f1dd738aa598ede55) | _ | hypothetical protein |
| [OCAR_6534](https://www.genoscope.cns.fr/agc/mage/wwwpkgdb/Info/getInfoLabel.php?id=3483501&wwwpkgdb=fc2733d6468c073f1dd738aa598ede55&nocache=a9a895ad49f0f55c362d050dc1ad3fa3&dir=&wwwpkgdb=fc2733d6468c073f1dd738aa598ede55) | _ | trap dicarboxylate transporter- dctm subunit |
| [OCAR_6536](https://www.genoscope.cns.fr/agc/mage/wwwpkgdb/Info/getInfoLabel.php?id=3483502&wwwpkgdb=fc2733d6468c073f1dd738aa598ede55&nocache=a9a895ad49f0f55c362d050dc1ad3fa3&dir=&wwwpkgdb=fc2733d6468c073f1dd738aa598ede55) | _ | hypothetical protein |
| [OCAR_6546](https://www.genoscope.cns.fr/agc/mage/wwwpkgdb/Info/getInfoLabel.php?id=3485222&wwwpkgdb=fc2733d6468c073f1dd738aa598ede55&nocache=a9a895ad49f0f55c362d050dc1ad3fa3&dir=&wwwpkgdb=fc2733d6468c073f1dd738aa598ede55) | _ | hypothetical protein |
| [OCAR_2528](https://www.genoscope.cns.fr/agc/mage/wwwpkgdb/Info/getInfoLabel.php?id=3481964&wwwpkgdb=fc2733d6468c073f1dd738aa598ede55&nocache=a9a895ad49f0f55c362d050dc1ad3fa3&dir=&wwwpkgdb=fc2733d6468c073f1dd738aa598ede55) | _ | _ |
| [OCAR_2530](https://www.genoscope.cns.fr/agc/mage/wwwpkgdb/Info/getInfoLabel.php?id=3481963&wwwpkgdb=fc2733d6468c073f1dd738aa598ede55&nocache=a9a895ad49f0f55c362d050dc1ad3fa3&dir=&wwwpkgdb=fc2733d6468c073f1dd738aa598ede55) | _ | _ |
| [OCAR_6567](https://www.genoscope.cns.fr/agc/mage/wwwpkgdb/Info/getInfoLabel.php?id=3483519&wwwpkgdb=fc2733d6468c073f1dd738aa598ede55&nocache=a9a895ad49f0f55c362d050dc1ad3fa3&dir=&wwwpkgdb=fc2733d6468c073f1dd738aa598ede55) | _ | hypothetical protein |
| [OCAR_6568](https://www.genoscope.cns.fr/agc/mage/wwwpkgdb/Info/getInfoLabel.php?id=3485233&wwwpkgdb=fc2733d6468c073f1dd738aa598ede55&nocache=a9a895ad49f0f55c362d050dc1ad3fa3&dir=&wwwpkgdb=fc2733d6468c073f1dd738aa598ede55) | _ | hypothetical protein |
| [OCAR_6583](https://www.genoscope.cns.fr/agc/mage/wwwpkgdb/Info/getInfoLabel.php?id=3485248&wwwpkgdb=fc2733d6468c073f1dd738aa598ede55&nocache=a9a895ad49f0f55c362d050dc1ad3fa3&dir=&wwwpkgdb=fc2733d6468c073f1dd738aa598ede55) | _ | hypothetical protein |
| [OCAR_6584](https://www.genoscope.cns.fr/agc/mage/wwwpkgdb/Info/getInfoLabel.php?id=3485249&wwwpkgdb=fc2733d6468c073f1dd738aa598ede55&nocache=a9a895ad49f0f55c362d050dc1ad3fa3&dir=&wwwpkgdb=fc2733d6468c073f1dd738aa598ede55) | _ | lysozyme |
| [OCAR_6585](https://www.genoscope.cns.fr/agc/mage/wwwpkgdb/Info/getInfoLabel.php?id=3483520&wwwpkgdb=fc2733d6468c073f1dd738aa598ede55&nocache=a9a895ad49f0f55c362d050dc1ad3fa3&dir=&wwwpkgdb=fc2733d6468c073f1dd738aa598ede55) | _ | transferase hexapeptide repeat protein |
| [OCAR_6601](https://www.genoscope.cns.fr/agc/mage/wwwpkgdb/Info/getInfoLabel.php?id=3485263&wwwpkgdb=fc2733d6468c073f1dd738aa598ede55&nocache=a9a895ad49f0f55c362d050dc1ad3fa3&dir=&wwwpkgdb=fc2733d6468c073f1dd738aa598ede55) | _ | hypothetical protein |
| [OCAR_6603](https://www.genoscope.cns.fr/agc/mage/wwwpkgdb/Info/getInfoLabel.php?id=3485265&wwwpkgdb=fc2733d6468c073f1dd738aa598ede55&nocache=a9a895ad49f0f55c362d050dc1ad3fa3&dir=&wwwpkgdb=fc2733d6468c073f1dd738aa598ede55) | _ | hypothetical protein |
| [OCAR_6604](https://www.genoscope.cns.fr/agc/mage/wwwpkgdb/Info/getInfoLabel.php?id=3485266&wwwpkgdb=fc2733d6468c073f1dd738aa598ede55&nocache=a9a895ad49f0f55c362d050dc1ad3fa3&dir=&wwwpkgdb=fc2733d6468c073f1dd738aa598ede55) | _ | hypothetical protein |
| [OCAR_6605](https://www.genoscope.cns.fr/agc/mage/wwwpkgdb/Info/getInfoLabel.php?id=3485267&wwwpkgdb=fc2733d6468c073f1dd738aa598ede55&nocache=a9a895ad49f0f55c362d050dc1ad3fa3&dir=&wwwpkgdb=fc2733d6468c073f1dd738aa598ede55) | _ | hypothetical protein |
| [OCAR_2591](https://www.genoscope.cns.fr/agc/mage/wwwpkgdb/Info/getInfoLabel.php?id=3481961&wwwpkgdb=fc2733d6468c073f1dd738aa598ede55&nocache=a9a895ad49f0f55c362d050dc1ad3fa3&dir=&wwwpkgdb=fc2733d6468c073f1dd738aa598ede55) | _ | _ |
| [OCAR_6614](https://www.genoscope.cns.fr/agc/mage/wwwpkgdb/Info/getInfoLabel.php?id=3485275&wwwpkgdb=fc2733d6468c073f1dd738aa598ede55&nocache=a9a895ad49f0f55c362d050dc1ad3fa3&dir=&wwwpkgdb=fc2733d6468c073f1dd738aa598ede55) | _ | hypothetical protein |
| [OCAR_6620](https://www.genoscope.cns.fr/agc/mage/wwwpkgdb/Info/getInfoLabel.php?id=3485278&wwwpkgdb=fc2733d6468c073f1dd738aa598ede55&nocache=a9a895ad49f0f55c362d050dc1ad3fa3&dir=&wwwpkgdb=fc2733d6468c073f1dd738aa598ede55) | _ | hypothetical protein |
| [OCAR_2599](https://www.genoscope.cns.fr/agc/mage/wwwpkgdb/Info/getInfoLabel.php?id=3482127&wwwpkgdb=fc2733d6468c073f1dd738aa598ede55&nocache=a9a895ad49f0f55c362d050dc1ad3fa3&dir=&wwwpkgdb=fc2733d6468c073f1dd738aa598ede55) | _ | _ |
| [OCAR_6623](https://www.genoscope.cns.fr/agc/mage/wwwpkgdb/Info/getInfoLabel.php?id=3485280&wwwpkgdb=fc2733d6468c073f1dd738aa598ede55&nocache=a9a895ad49f0f55c362d050dc1ad3fa3&dir=&wwwpkgdb=fc2733d6468c073f1dd738aa598ede55) | _ | hypothetical protein |
| [OCAR_2605](https://www.genoscope.cns.fr/agc/mage/wwwpkgdb/Info/getInfoLabel.php?id=3481960&wwwpkgdb=fc2733d6468c073f1dd738aa598ede55&nocache=a9a895ad49f0f55c362d050dc1ad3fa3&dir=&wwwpkgdb=fc2733d6468c073f1dd738aa598ede55) | _ | _ |
| [OCAR_6627](https://www.genoscope.cns.fr/agc/mage/wwwpkgdb/Info/getInfoLabel.php?id=3483529&wwwpkgdb=fc2733d6468c073f1dd738aa598ede55&nocache=a9a895ad49f0f55c362d050dc1ad3fa3&dir=&wwwpkgdb=fc2733d6468c073f1dd738aa598ede55) | _ | hypothetical protein |
| [OCAR_2609](https://www.genoscope.cns.fr/agc/mage/wwwpkgdb/Info/getInfoLabel.php?id=3481959&wwwpkgdb=fc2733d6468c073f1dd738aa598ede55&nocache=a9a895ad49f0f55c362d050dc1ad3fa3&dir=&wwwpkgdb=fc2733d6468c073f1dd738aa598ede55) | _ | _ |
| [OCAR_6633](https://www.genoscope.cns.fr/agc/mage/wwwpkgdb/Info/getInfoLabel.php?id=3485286&wwwpkgdb=fc2733d6468c073f1dd738aa598ede55&nocache=a9a895ad49f0f55c362d050dc1ad3fa3&dir=&wwwpkgdb=fc2733d6468c073f1dd738aa598ede55) | _ | hypothetical protein |
| [OCAR_6634](https://www.genoscope.cns.fr/agc/mage/wwwpkgdb/Info/getInfoLabel.php?id=3485287&wwwpkgdb=fc2733d6468c073f1dd738aa598ede55&nocache=a9a895ad49f0f55c362d050dc1ad3fa3&dir=&wwwpkgdb=fc2733d6468c073f1dd738aa598ede55) | _ | hypothetical protein |
| [OCAR_2615](https://www.genoscope.cns.fr/agc/mage/wwwpkgdb/Info/getInfoLabel.php?id=3481958&wwwpkgdb=fc2733d6468c073f1dd738aa598ede55&nocache=a9a895ad49f0f55c362d050dc1ad3fa3&dir=&wwwpkgdb=fc2733d6468c073f1dd738aa598ede55) | _ | _ |
| [OCAR_2618](https://www.genoscope.cns.fr/agc/mage/wwwpkgdb/Info/getInfoLabel.php?id=3481957&wwwpkgdb=fc2733d6468c073f1dd738aa598ede55&nocache=a9a895ad49f0f55c362d050dc1ad3fa3&dir=&wwwpkgdb=fc2733d6468c073f1dd738aa598ede55) | _ | _ |
| [OCAR_6645](https://www.genoscope.cns.fr/agc/mage/wwwpkgdb/Info/getInfoLabel.php?id=3485292&wwwpkgdb=fc2733d6468c073f1dd738aa598ede55&nocache=a9a895ad49f0f55c362d050dc1ad3fa3&dir=&wwwpkgdb=fc2733d6468c073f1dd738aa598ede55) | _ | hypothetical protein |
| [OCAR_6648](https://www.genoscope.cns.fr/agc/mage/wwwpkgdb/Info/getInfoLabel.php?id=3485293&wwwpkgdb=fc2733d6468c073f1dd738aa598ede55&nocache=a9a895ad49f0f55c362d050dc1ad3fa3&dir=&wwwpkgdb=fc2733d6468c073f1dd738aa598ede55) | _ | hypothetical protein |
| [OCAR_6656](https://www.genoscope.cns.fr/agc/mage/wwwpkgdb/Info/getInfoLabel.php?id=3483543&wwwpkgdb=fc2733d6468c073f1dd738aa598ede55&nocache=a9a895ad49f0f55c362d050dc1ad3fa3&dir=&wwwpkgdb=fc2733d6468c073f1dd738aa598ede55) | _ | hypothetical protein |
| [OCAR_2640](https://www.genoscope.cns.fr/agc/mage/wwwpkgdb/Info/getInfoLabel.php?id=3481956&wwwpkgdb=fc2733d6468c073f1dd738aa598ede55&nocache=a9a895ad49f0f55c362d050dc1ad3fa3&dir=&wwwpkgdb=fc2733d6468c073f1dd738aa598ede55) | _ | _ |
| [OCAR_6669](https://www.genoscope.cns.fr/agc/mage/wwwpkgdb/Info/getInfoLabel.php?id=3485302&wwwpkgdb=fc2733d6468c073f1dd738aa598ede55&nocache=a9a895ad49f0f55c362d050dc1ad3fa3&dir=&wwwpkgdb=fc2733d6468c073f1dd738aa598ede55) | _ | hypothetical protein |
| [OCAR_2651](https://www.genoscope.cns.fr/agc/mage/wwwpkgdb/Info/getInfoLabel.php?id=3481955&wwwpkgdb=fc2733d6468c073f1dd738aa598ede55&nocache=a9a895ad49f0f55c362d050dc1ad3fa3&dir=&wwwpkgdb=fc2733d6468c073f1dd738aa598ede55) | _ | _ |
| [OCAR_6683](https://www.genoscope.cns.fr/agc/mage/wwwpkgdb/Info/getInfoLabel.php?id=3485313&wwwpkgdb=fc2733d6468c073f1dd738aa598ede55&nocache=a9a895ad49f0f55c362d050dc1ad3fa3&dir=&wwwpkgdb=fc2733d6468c073f1dd738aa598ede55) | _ | hypothetical protein |
| [OCAR_2676](https://www.genoscope.cns.fr/agc/mage/wwwpkgdb/Info/getInfoLabel.php?id=3481954&wwwpkgdb=fc2733d6468c073f1dd738aa598ede55&nocache=a9a895ad49f0f55c362d050dc1ad3fa3&dir=&wwwpkgdb=fc2733d6468c073f1dd738aa598ede55) | _ | _ |
| [OCAR_6700](https://www.genoscope.cns.fr/agc/mage/wwwpkgdb/Info/getInfoLabel.php?id=3485323&wwwpkgdb=fc2733d6468c073f1dd738aa598ede55&nocache=a9a895ad49f0f55c362d050dc1ad3fa3&dir=&wwwpkgdb=fc2733d6468c073f1dd738aa598ede55) | _ | hypothetical protein |
| [OCAR_2684](https://www.genoscope.cns.fr/agc/mage/wwwpkgdb/Info/getInfoLabel.php?id=3481953&wwwpkgdb=fc2733d6468c073f1dd738aa598ede55&nocache=a9a895ad49f0f55c362d050dc1ad3fa3&dir=&wwwpkgdb=fc2733d6468c073f1dd738aa598ede55) | _ | _ |
| [OCAR_6704](https://www.genoscope.cns.fr/agc/mage/wwwpkgdb/Info/getInfoLabel.php?id=3483561&wwwpkgdb=fc2733d6468c073f1dd738aa598ede55&nocache=a9a895ad49f0f55c362d050dc1ad3fa3&dir=&wwwpkgdb=fc2733d6468c073f1dd738aa598ede55) | _ | hypothetical protein |
| [OCAR_6707](https://www.genoscope.cns.fr/agc/mage/wwwpkgdb/Info/getInfoLabel.php?id=3483563&wwwpkgdb=fc2733d6468c073f1dd738aa598ede55&nocache=a9a895ad49f0f55c362d050dc1ad3fa3&dir=&wwwpkgdb=fc2733d6468c073f1dd738aa598ede55) | _ | ATPase component of tungstate ABC transporter |
| [OCAR_6710](https://www.genoscope.cns.fr/agc/mage/wwwpkgdb/Info/getInfoLabel.php?id=3485329&wwwpkgdb=fc2733d6468c073f1dd738aa598ede55&nocache=a9a895ad49f0f55c362d050dc1ad3fa3&dir=&wwwpkgdb=fc2733d6468c073f1dd738aa598ede55) | _ | hypothetical protein |
| [OCAR_2695](https://www.genoscope.cns.fr/agc/mage/wwwpkgdb/Info/getInfoLabel.php?id=3481952&wwwpkgdb=fc2733d6468c073f1dd738aa598ede55&nocache=a9a895ad49f0f55c362d050dc1ad3fa3&dir=&wwwpkgdb=fc2733d6468c073f1dd738aa598ede55) | _ | _ |
| [OCAR_6730](https://www.genoscope.cns.fr/agc/mage/wwwpkgdb/Info/getInfoLabel.php?id=3485344&wwwpkgdb=fc2733d6468c073f1dd738aa598ede55&nocache=a9a895ad49f0f55c362d050dc1ad3fa3&dir=&wwwpkgdb=fc2733d6468c073f1dd738aa598ede55) | _ | hypothetical protein |
| [OCAR_6733](https://www.genoscope.cns.fr/agc/mage/wwwpkgdb/Info/getInfoLabel.php?id=3483571&wwwpkgdb=fc2733d6468c073f1dd738aa598ede55&nocache=a9a895ad49f0f55c362d050dc1ad3fa3&dir=&wwwpkgdb=fc2733d6468c073f1dd738aa598ede55) | _ | hypothetical protein |
| [OCAR_6735](https://www.genoscope.cns.fr/agc/mage/wwwpkgdb/Info/getInfoLabel.php?id=3483572&wwwpkgdb=fc2733d6468c073f1dd738aa598ede55&nocache=a9a895ad49f0f55c362d050dc1ad3fa3&dir=&wwwpkgdb=fc2733d6468c073f1dd738aa598ede55) | _ | hypothetical protein |
| [OCAR_6738](https://www.genoscope.cns.fr/agc/mage/wwwpkgdb/Info/getInfoLabel.php?id=3485349&wwwpkgdb=fc2733d6468c073f1dd738aa598ede55&nocache=a9a895ad49f0f55c362d050dc1ad3fa3&dir=&wwwpkgdb=fc2733d6468c073f1dd738aa598ede55) | _ | succinate-semialdehyde dehydrogenase |
| [OCAR_6739](https://www.genoscope.cns.fr/agc/mage/wwwpkgdb/Info/getInfoLabel.php?id=3483573&wwwpkgdb=fc2733d6468c073f1dd738aa598ede55&nocache=a9a895ad49f0f55c362d050dc1ad3fa3&dir=&wwwpkgdb=fc2733d6468c073f1dd738aa598ede55) | _ | hypothetical protein |
| [OCAR_6741](https://www.genoscope.cns.fr/agc/mage/wwwpkgdb/Info/getInfoLabel.php?id=3485351&wwwpkgdb=fc2733d6468c073f1dd738aa598ede55&nocache=a9a895ad49f0f55c362d050dc1ad3fa3&dir=&wwwpkgdb=fc2733d6468c073f1dd738aa598ede55) | _ | hypothetical protein |
| [OCAR_6744](https://www.genoscope.cns.fr/agc/mage/wwwpkgdb/Info/getInfoLabel.php?id=3485353&wwwpkgdb=fc2733d6468c073f1dd738aa598ede55&nocache=a9a895ad49f0f55c362d050dc1ad3fa3&dir=&wwwpkgdb=fc2733d6468c073f1dd738aa598ede55) | xsc | sulfoacetaldehyde acetyltransferase |
| [OCAR_2728](https://www.genoscope.cns.fr/agc/mage/wwwpkgdb/Info/getInfoLabel.php?id=3481951&wwwpkgdb=fc2733d6468c073f1dd738aa598ede55&nocache=a9a895ad49f0f55c362d050dc1ad3fa3&dir=&wwwpkgdb=fc2733d6468c073f1dd738aa598ede55) | _ | _ |
| [OCAR_6750](https://www.genoscope.cns.fr/agc/mage/wwwpkgdb/Info/getInfoLabel.php?id=3483578&wwwpkgdb=fc2733d6468c073f1dd738aa598ede55&nocache=a9a895ad49f0f55c362d050dc1ad3fa3&dir=&wwwpkgdb=fc2733d6468c073f1dd738aa598ede55) | _ | hypothetical protein |
| [OCAR_6753](https://www.genoscope.cns.fr/agc/mage/wwwpkgdb/Info/getInfoLabel.php?id=3485358&wwwpkgdb=fc2733d6468c073f1dd738aa598ede55&nocache=a9a895ad49f0f55c362d050dc1ad3fa3&dir=&wwwpkgdb=fc2733d6468c073f1dd738aa598ede55) | _ | hypothetical protein |
| [OCAR_6756](https://www.genoscope.cns.fr/agc/mage/wwwpkgdb/Info/getInfoLabel.php?id=3483581&wwwpkgdb=fc2733d6468c073f1dd738aa598ede55&nocache=a9a895ad49f0f55c362d050dc1ad3fa3&dir=&wwwpkgdb=fc2733d6468c073f1dd738aa598ede55) | _ | ggdef domain protein |
| [OCAR_6757](https://www.genoscope.cns.fr/agc/mage/wwwpkgdb/Info/getInfoLabel.php?id=3483582&wwwpkgdb=fc2733d6468c073f1dd738aa598ede55&nocache=a9a895ad49f0f55c362d050dc1ad3fa3&dir=&wwwpkgdb=fc2733d6468c073f1dd738aa598ede55) | _ | hypothetical protein |
| [OCAR_6764](https://www.genoscope.cns.fr/agc/mage/wwwpkgdb/Info/getInfoLabel.php?id=3483587&wwwpkgdb=fc2733d6468c073f1dd738aa598ede55&nocache=a9a895ad49f0f55c362d050dc1ad3fa3&dir=&wwwpkgdb=fc2733d6468c073f1dd738aa598ede55) | _ | hypothetical protein |
| [OCAR_6767](https://www.genoscope.cns.fr/agc/mage/wwwpkgdb/Info/getInfoLabel.php?id=3485363&wwwpkgdb=fc2733d6468c073f1dd738aa598ede55&nocache=a9a895ad49f0f55c362d050dc1ad3fa3&dir=&wwwpkgdb=fc2733d6468c073f1dd738aa598ede55) | fdxH | formate dehydrogenase, beta subunit |
| [OCAR_6768](https://www.genoscope.cns.fr/agc/mage/wwwpkgdb/Info/getInfoLabel.php?id=3485364&wwwpkgdb=fc2733d6468c073f1dd738aa598ede55&nocache=a9a895ad49f0f55c362d050dc1ad3fa3&dir=&wwwpkgdb=fc2733d6468c073f1dd738aa598ede55) | _ | formate dehydrogenase, alpha subunit |
| [OCAR_2751](https://www.genoscope.cns.fr/agc/mage/wwwpkgdb/Info/getInfoLabel.php?id=3481950&wwwpkgdb=fc2733d6468c073f1dd738aa598ede55&nocache=a9a895ad49f0f55c362d050dc1ad3fa3&dir=&wwwpkgdb=fc2733d6468c073f1dd738aa598ede55) | _ | _ |
| [OCAR_6770](https://www.genoscope.cns.fr/agc/mage/wwwpkgdb/Info/getInfoLabel.php?id=3483589&wwwpkgdb=fc2733d6468c073f1dd738aa598ede55&nocache=a9a895ad49f0f55c362d050dc1ad3fa3&dir=&wwwpkgdb=fc2733d6468c073f1dd738aa598ede55) | _ | hypothetical protein |
| [OCAR_6775](https://www.genoscope.cns.fr/agc/mage/wwwpkgdb/Info/getInfoLabel.php?id=3485370&wwwpkgdb=fc2733d6468c073f1dd738aa598ede55&nocache=a9a895ad49f0f55c362d050dc1ad3fa3&dir=&wwwpkgdb=fc2733d6468c073f1dd738aa598ede55) | _ | 3-oxoadipate:succinyl-CoA transferase subunit B |
| [OCAR_6777](https://www.genoscope.cns.fr/agc/mage/wwwpkgdb/Info/getInfoLabel.php?id=3485372&wwwpkgdb=fc2733d6468c073f1dd738aa598ede55&nocache=a9a895ad49f0f55c362d050dc1ad3fa3&dir=&wwwpkgdb=fc2733d6468c073f1dd738aa598ede55) | _ | coenzyme A transferase |
| [OCAR_6782](https://www.genoscope.cns.fr/agc/mage/wwwpkgdb/Info/getInfoLabel.php?id=3485377&wwwpkgdb=fc2733d6468c073f1dd738aa598ede55&nocache=a9a895ad49f0f55c362d050dc1ad3fa3&dir=&wwwpkgdb=fc2733d6468c073f1dd738aa598ede55) | _ | transcriptional regulator, IclR family |
| [OCAR_6783](https://www.genoscope.cns.fr/agc/mage/wwwpkgdb/Info/getInfoLabel.php?id=3483590&wwwpkgdb=fc2733d6468c073f1dd738aa598ede55&nocache=a9a895ad49f0f55c362d050dc1ad3fa3&dir=&wwwpkgdb=fc2733d6468c073f1dd738aa598ede55) | _ | hypothetical protein |
| [OCAR_6784](https://www.genoscope.cns.fr/agc/mage/wwwpkgdb/Info/getInfoLabel.php?id=3485378&wwwpkgdb=fc2733d6468c073f1dd738aa598ede55&nocache=a9a895ad49f0f55c362d050dc1ad3fa3&dir=&wwwpkgdb=fc2733d6468c073f1dd738aa598ede55) | _ | cysteine dioxygenase type I |
| [OCAR_6786](https://www.genoscope.cns.fr/agc/mage/wwwpkgdb/Info/getInfoLabel.php?id=3485380&wwwpkgdb=fc2733d6468c073f1dd738aa598ede55&nocache=a9a895ad49f0f55c362d050dc1ad3fa3&dir=&wwwpkgdb=fc2733d6468c073f1dd738aa598ede55) | _ | putative protein of unknown function DUF81 |
| [OCAR_6789](https://www.genoscope.cns.fr/agc/mage/wwwpkgdb/Info/getInfoLabel.php?id=3485383&wwwpkgdb=fc2733d6468c073f1dd738aa598ede55&nocache=a9a895ad49f0f55c362d050dc1ad3fa3&dir=&wwwpkgdb=fc2733d6468c073f1dd738aa598ede55) | _ | methanesulfonate monooxygenase component; reductase |
| [OCAR_6793](https://www.genoscope.cns.fr/agc/mage/wwwpkgdb/Info/getInfoLabel.php?id=3485387&wwwpkgdb=fc2733d6468c073f1dd738aa598ede55&nocache=a9a895ad49f0f55c362d050dc1ad3fa3&dir=&wwwpkgdb=fc2733d6468c073f1dd738aa598ede55) | _ | hypothetical protein |
| [OCAR_6794](https://www.genoscope.cns.fr/agc/mage/wwwpkgdb/Info/getInfoLabel.php?id=3485388&wwwpkgdb=fc2733d6468c073f1dd738aa598ede55&nocache=a9a895ad49f0f55c362d050dc1ad3fa3&dir=&wwwpkgdb=fc2733d6468c073f1dd738aa598ede55) | _ | hypothetical protein |
| [OCAR_6795](https://www.genoscope.cns.fr/agc/mage/wwwpkgdb/Info/getInfoLabel.php?id=3483591&wwwpkgdb=fc2733d6468c073f1dd738aa598ede55&nocache=a9a895ad49f0f55c362d050dc1ad3fa3&dir=&wwwpkgdb=fc2733d6468c073f1dd738aa598ede55) | _ | O-succinylbenzoate-CoA synthase |
| [OCAR_6796](https://www.genoscope.cns.fr/agc/mage/wwwpkgdb/Info/getInfoLabel.php?id=3483592&wwwpkgdb=fc2733d6468c073f1dd738aa598ede55&nocache=a9a895ad49f0f55c362d050dc1ad3fa3&dir=&wwwpkgdb=fc2733d6468c073f1dd738aa598ede55) | _ | transcriptional regulator, IclR family |
| [OCAR_6797](https://www.genoscope.cns.fr/agc/mage/wwwpkgdb/Info/getInfoLabel.php?id=3483593&wwwpkgdb=fc2733d6468c073f1dd738aa598ede55&nocache=a9a895ad49f0f55c362d050dc1ad3fa3&dir=&wwwpkgdb=fc2733d6468c073f1dd738aa598ede55) | _ | glycosyl transferase, family 2 |
| [OCAR_6800](https://www.genoscope.cns.fr/agc/mage/wwwpkgdb/Info/getInfoLabel.php?id=3485389&wwwpkgdb=fc2733d6468c073f1dd738aa598ede55&nocache=a9a895ad49f0f55c362d050dc1ad3fa3&dir=&wwwpkgdb=fc2733d6468c073f1dd738aa598ede55) | _ | putative ABC branched chain amino acid family transporter |
| [OCAR_6804](https://www.genoscope.cns.fr/agc/mage/wwwpkgdb/Info/getInfoLabel.php?id=3483596&wwwpkgdb=fc2733d6468c073f1dd738aa598ede55&nocache=a9a895ad49f0f55c362d050dc1ad3fa3&dir=&wwwpkgdb=fc2733d6468c073f1dd738aa598ede55) | _ | hypothetical protein |
| [OCAR_6808](https://www.genoscope.cns.fr/agc/mage/wwwpkgdb/Info/getInfoLabel.php?id=3485392&wwwpkgdb=fc2733d6468c073f1dd738aa598ede55&nocache=a9a895ad49f0f55c362d050dc1ad3fa3&dir=&wwwpkgdb=fc2733d6468c073f1dd738aa598ede55) | _ | phosophohydrolase |
| [OCAR_2790](https://www.genoscope.cns.fr/agc/mage/wwwpkgdb/Info/getInfoLabel.php?id=3481949&wwwpkgdb=fc2733d6468c073f1dd738aa598ede55&nocache=a9a895ad49f0f55c362d050dc1ad3fa3&dir=&wwwpkgdb=fc2733d6468c073f1dd738aa598ede55) | _ | _ |
| [OCAR_6812](https://www.genoscope.cns.fr/agc/mage/wwwpkgdb/Info/getInfoLabel.php?id=3483601&wwwpkgdb=fc2733d6468c073f1dd738aa598ede55&nocache=a9a895ad49f0f55c362d050dc1ad3fa3&dir=&wwwpkgdb=fc2733d6468c073f1dd738aa598ede55) | _ | GntR-family transcriptional regulator |
| [OCAR_6813](https://www.genoscope.cns.fr/agc/mage/wwwpkgdb/Info/getInfoLabel.php?id=3485395&wwwpkgdb=fc2733d6468c073f1dd738aa598ede55&nocache=a9a895ad49f0f55c362d050dc1ad3fa3&dir=&wwwpkgdb=fc2733d6468c073f1dd738aa598ede55) | _ | putative ggdef family protein |
| [OCAR_2796](https://www.genoscope.cns.fr/agc/mage/wwwpkgdb/Info/getInfoLabel.php?id=3481948&wwwpkgdb=fc2733d6468c073f1dd738aa598ede55&nocache=a9a895ad49f0f55c362d050dc1ad3fa3&dir=&wwwpkgdb=fc2733d6468c073f1dd738aa598ede55) | _ | _ |
| [OCAR_6819](https://www.genoscope.cns.fr/agc/mage/wwwpkgdb/Info/getInfoLabel.php?id=3485396&wwwpkgdb=fc2733d6468c073f1dd738aa598ede55&nocache=a9a895ad49f0f55c362d050dc1ad3fa3&dir=&wwwpkgdb=fc2733d6468c073f1dd738aa598ede55) | _ | hypothetical protein |
| [OCAR_2802](https://www.genoscope.cns.fr/agc/mage/wwwpkgdb/Info/getInfoLabel.php?id=3481947&wwwpkgdb=fc2733d6468c073f1dd738aa598ede55&nocache=a9a895ad49f0f55c362d050dc1ad3fa3&dir=&wwwpkgdb=fc2733d6468c073f1dd738aa598ede55) | _ | _ |
| [OCAR_2806](https://www.genoscope.cns.fr/agc/mage/wwwpkgdb/Info/getInfoLabel.php?id=3481946&wwwpkgdb=fc2733d6468c073f1dd738aa598ede55&nocache=a9a895ad49f0f55c362d050dc1ad3fa3&dir=&wwwpkgdb=fc2733d6468c073f1dd738aa598ede55) | _ | _ |
| [OCAR_6823](https://www.genoscope.cns.fr/agc/mage/wwwpkgdb/Info/getInfoLabel.php?id=3483609&wwwpkgdb=fc2733d6468c073f1dd738aa598ede55&nocache=a9a895ad49f0f55c362d050dc1ad3fa3&dir=&wwwpkgdb=fc2733d6468c073f1dd738aa598ede55) | _ | hypothetical protein |
| [OCAR_6824](https://www.genoscope.cns.fr/agc/mage/wwwpkgdb/Info/getInfoLabel.php?id=3483610&wwwpkgdb=fc2733d6468c073f1dd738aa598ede55&nocache=a9a895ad49f0f55c362d050dc1ad3fa3&dir=&wwwpkgdb=fc2733d6468c073f1dd738aa598ede55) | _ | hypothetical protein |
| [OCAR_2809](https://www.genoscope.cns.fr/agc/mage/wwwpkgdb/Info/getInfoLabel.php?id=3481945&wwwpkgdb=fc2733d6468c073f1dd738aa598ede55&nocache=a9a895ad49f0f55c362d050dc1ad3fa3&dir=&wwwpkgdb=fc2733d6468c073f1dd738aa598ede55) | _ | _ |
| [OCAR_6842](https://www.genoscope.cns.fr/agc/mage/wwwpkgdb/Info/getInfoLabel.php?id=3485411&wwwpkgdb=fc2733d6468c073f1dd738aa598ede55&nocache=a9a895ad49f0f55c362d050dc1ad3fa3&dir=&wwwpkgdb=fc2733d6468c073f1dd738aa598ede55) | _ | hypothetical protein |
| [OCAR_6846](https://www.genoscope.cns.fr/agc/mage/wwwpkgdb/Info/getInfoLabel.php?id=3485415&wwwpkgdb=fc2733d6468c073f1dd738aa598ede55&nocache=a9a895ad49f0f55c362d050dc1ad3fa3&dir=&wwwpkgdb=fc2733d6468c073f1dd738aa598ede55) | _ | periplasmic binding protein |
| [OCAR_6849](https://www.genoscope.cns.fr/agc/mage/wwwpkgdb/Info/getInfoLabel.php?id=3485418&wwwpkgdb=fc2733d6468c073f1dd738aa598ede55&nocache=a9a895ad49f0f55c362d050dc1ad3fa3&dir=&wwwpkgdb=fc2733d6468c073f1dd738aa598ede55) | _ | binding-protein-dependent transport systems inner membrane component |
| [OCAR_6850](https://www.genoscope.cns.fr/agc/mage/wwwpkgdb/Info/getInfoLabel.php?id=3485419&wwwpkgdb=fc2733d6468c073f1dd738aa598ede55&nocache=a9a895ad49f0f55c362d050dc1ad3fa3&dir=&wwwpkgdb=fc2733d6468c073f1dd738aa598ede55) | _ | extracellular solute-binding protein, family 1 |
| [OCAR_6851](https://www.genoscope.cns.fr/agc/mage/wwwpkgdb/Info/getInfoLabel.php?id=3485420&wwwpkgdb=fc2733d6468c073f1dd738aa598ede55&nocache=a9a895ad49f0f55c362d050dc1ad3fa3&dir=&wwwpkgdb=fc2733d6468c073f1dd738aa598ede55) | phnA | phosphonoacetate hydrolase |
| [OCAR_6854](https://www.genoscope.cns.fr/agc/mage/wwwpkgdb/Info/getInfoLabel.php?id=3485421&wwwpkgdb=fc2733d6468c073f1dd738aa598ede55&nocache=a9a895ad49f0f55c362d050dc1ad3fa3&dir=&wwwpkgdb=fc2733d6468c073f1dd738aa598ede55) | _ | hypothetical protein |
| [OCAR_6857](https://www.genoscope.cns.fr/agc/mage/wwwpkgdb/Info/getInfoLabel.php?id=3485423&wwwpkgdb=fc2733d6468c073f1dd738aa598ede55&nocache=a9a895ad49f0f55c362d050dc1ad3fa3&dir=&wwwpkgdb=fc2733d6468c073f1dd738aa598ede55) | _ | hypothetical protein |
| [OCAR_6862](https://www.genoscope.cns.fr/agc/mage/wwwpkgdb/Info/getInfoLabel.php?id=3485428&wwwpkgdb=fc2733d6468c073f1dd738aa598ede55&nocache=a9a895ad49f0f55c362d050dc1ad3fa3&dir=&wwwpkgdb=fc2733d6468c073f1dd738aa598ede55) | _ | twin-arginine translocation pathway signal |
| [OCAR_6863](https://www.genoscope.cns.fr/agc/mage/wwwpkgdb/Info/getInfoLabel.php?id=3483618&wwwpkgdb=fc2733d6468c073f1dd738aa598ede55&nocache=a9a895ad49f0f55c362d050dc1ad3fa3&dir=&wwwpkgdb=fc2733d6468c073f1dd738aa598ede55) | _ | fatty acid hydroxylase |
| [OCAR_6864](https://www.genoscope.cns.fr/agc/mage/wwwpkgdb/Info/getInfoLabel.php?id=3483619&wwwpkgdb=fc2733d6468c073f1dd738aa598ede55&nocache=a9a895ad49f0f55c362d050dc1ad3fa3&dir=&wwwpkgdb=fc2733d6468c073f1dd738aa598ede55) | _ | hypothetical protein |
| [OCAR_6865](https://www.genoscope.cns.fr/agc/mage/wwwpkgdb/Info/getInfoLabel.php?id=3483620&wwwpkgdb=fc2733d6468c073f1dd738aa598ede55&nocache=a9a895ad49f0f55c362d050dc1ad3fa3&dir=&wwwpkgdb=fc2733d6468c073f1dd738aa598ede55) | _ | hypothetical protein |
| [OCAR_2858](https://www.genoscope.cns.fr/agc/mage/wwwpkgdb/Info/getInfoLabel.php?id=3481944&wwwpkgdb=fc2733d6468c073f1dd738aa598ede55&nocache=a9a895ad49f0f55c362d050dc1ad3fa3&dir=&wwwpkgdb=fc2733d6468c073f1dd738aa598ede55) | _ | _ |
| [OCAR_2859](https://www.genoscope.cns.fr/agc/mage/wwwpkgdb/Info/getInfoLabel.php?id=3481943&wwwpkgdb=fc2733d6468c073f1dd738aa598ede55&nocache=a9a895ad49f0f55c362d050dc1ad3fa3&dir=&wwwpkgdb=fc2733d6468c073f1dd738aa598ede55) | _ | _ |
| [OCAR_6876](https://www.genoscope.cns.fr/agc/mage/wwwpkgdb/Info/getInfoLabel.php?id=3483621&wwwpkgdb=fc2733d6468c073f1dd738aa598ede55&nocache=a9a895ad49f0f55c362d050dc1ad3fa3&dir=&wwwpkgdb=fc2733d6468c073f1dd738aa598ede55) | _ | hypothetical protein |
| [OCAR_6877](https://www.genoscope.cns.fr/agc/mage/wwwpkgdb/Info/getInfoLabel.php?id=3483622&wwwpkgdb=fc2733d6468c073f1dd738aa598ede55&nocache=a9a895ad49f0f55c362d050dc1ad3fa3&dir=&wwwpkgdb=fc2733d6468c073f1dd738aa598ede55) | _ | carboxymuconolactone decarboxylase |
| [OCAR_6880](https://www.genoscope.cns.fr/agc/mage/wwwpkgdb/Info/getInfoLabel.php?id=3483625&wwwpkgdb=fc2733d6468c073f1dd738aa598ede55&nocache=a9a895ad49f0f55c362d050dc1ad3fa3&dir=&wwwpkgdb=fc2733d6468c073f1dd738aa598ede55) | _ | ABC-type nitrate/sulfonate/bicarbonate transport systems periplasmic components |
| [OCAR_6881](https://www.genoscope.cns.fr/agc/mage/wwwpkgdb/Info/getInfoLabel.php?id=3483626&wwwpkgdb=fc2733d6468c073f1dd738aa598ede55&nocache=a9a895ad49f0f55c362d050dc1ad3fa3&dir=&wwwpkgdb=fc2733d6468c073f1dd738aa598ede55) | _ | binding-protein-dependent transport systems inner membrane component |
| [OCAR_2866](https://www.genoscope.cns.fr/agc/mage/wwwpkgdb/Info/getInfoLabel.php?id=3481942&wwwpkgdb=fc2733d6468c073f1dd738aa598ede55&nocache=a9a895ad49f0f55c362d050dc1ad3fa3&dir=&wwwpkgdb=fc2733d6468c073f1dd738aa598ede55) | _ | _ |
| [OCAR_6883](https://www.genoscope.cns.fr/agc/mage/wwwpkgdb/Info/getInfoLabel.php?id=3483627&wwwpkgdb=fc2733d6468c073f1dd738aa598ede55&nocache=a9a895ad49f0f55c362d050dc1ad3fa3&dir=&wwwpkgdb=fc2733d6468c073f1dd738aa598ede55) | _ | hypothetical protein |
| [OCAR_6886](https://www.genoscope.cns.fr/agc/mage/wwwpkgdb/Info/getInfoLabel.php?id=3483629&wwwpkgdb=fc2733d6468c073f1dd738aa598ede55&nocache=a9a895ad49f0f55c362d050dc1ad3fa3&dir=&wwwpkgdb=fc2733d6468c073f1dd738aa598ede55) | _ | hypothetical protein |
| [OCAR_6887](https://www.genoscope.cns.fr/agc/mage/wwwpkgdb/Info/getInfoLabel.php?id=3483630&wwwpkgdb=fc2733d6468c073f1dd738aa598ede55&nocache=a9a895ad49f0f55c362d050dc1ad3fa3&dir=&wwwpkgdb=fc2733d6468c073f1dd738aa598ede55) | _ | glutamine amidotransferase class-I |
| [OCAR_2878](https://www.genoscope.cns.fr/agc/mage/wwwpkgdb/Info/getInfoLabel.php?id=3481941&wwwpkgdb=fc2733d6468c073f1dd738aa598ede55&nocache=a9a895ad49f0f55c362d050dc1ad3fa3&dir=&wwwpkgdb=fc2733d6468c073f1dd738aa598ede55) | _ | _ |
| [OCAR_6900](https://www.genoscope.cns.fr/agc/mage/wwwpkgdb/Info/getInfoLabel.php?id=3483640&wwwpkgdb=fc2733d6468c073f1dd738aa598ede55&nocache=a9a895ad49f0f55c362d050dc1ad3fa3&dir=&wwwpkgdb=fc2733d6468c073f1dd738aa598ede55) | _ | hypothetical protein |
| [OCAR_6913](https://www.genoscope.cns.fr/agc/mage/wwwpkgdb/Info/getInfoLabel.php?id=3485451&wwwpkgdb=fc2733d6468c073f1dd738aa598ede55&nocache=a9a895ad49f0f55c362d050dc1ad3fa3&dir=&wwwpkgdb=fc2733d6468c073f1dd738aa598ede55) | _ | CutA1 divalent ion tolerance protein |
| [OCAR_2905](https://www.genoscope.cns.fr/agc/mage/wwwpkgdb/Info/getInfoLabel.php?id=3481940&wwwpkgdb=fc2733d6468c073f1dd738aa598ede55&nocache=a9a895ad49f0f55c362d050dc1ad3fa3&dir=&wwwpkgdb=fc2733d6468c073f1dd738aa598ede55) | _ | _ |
| [OCAR_6937](https://www.genoscope.cns.fr/agc/mage/wwwpkgdb/Info/getInfoLabel.php?id=3483661&wwwpkgdb=fc2733d6468c073f1dd738aa598ede55&nocache=a9a895ad49f0f55c362d050dc1ad3fa3&dir=&wwwpkgdb=fc2733d6468c073f1dd738aa598ede55) | _ | hypothetical protein |
| [OCAR_6941](https://www.genoscope.cns.fr/agc/mage/wwwpkgdb/Info/getInfoLabel.php?id=3485462&wwwpkgdb=fc2733d6468c073f1dd738aa598ede55&nocache=a9a895ad49f0f55c362d050dc1ad3fa3&dir=&wwwpkgdb=fc2733d6468c073f1dd738aa598ede55) | _ | hypothetical protein |
| [OCAR_6950](https://www.genoscope.cns.fr/agc/mage/wwwpkgdb/Info/getInfoLabel.php?id=3485467&wwwpkgdb=fc2733d6468c073f1dd738aa598ede55&nocache=a9a895ad49f0f55c362d050dc1ad3fa3&dir=&wwwpkgdb=fc2733d6468c073f1dd738aa598ede55) | _ | hypothetical protein |
| [OCAR_6951](https://www.genoscope.cns.fr/agc/mage/wwwpkgdb/Info/getInfoLabel.php?id=3483667&wwwpkgdb=fc2733d6468c073f1dd738aa598ede55&nocache=a9a895ad49f0f55c362d050dc1ad3fa3&dir=&wwwpkgdb=fc2733d6468c073f1dd738aa598ede55) | _ | hypothetical protein |
| [OCAR_6953](https://www.genoscope.cns.fr/agc/mage/wwwpkgdb/Info/getInfoLabel.php?id=3483668&wwwpkgdb=fc2733d6468c073f1dd738aa598ede55&nocache=a9a895ad49f0f55c362d050dc1ad3fa3&dir=&wwwpkgdb=fc2733d6468c073f1dd738aa598ede55) | _ | hypothetical protein |
| [OCAR_6961](https://www.genoscope.cns.fr/agc/mage/wwwpkgdb/Info/getInfoLabel.php?id=3483670&wwwpkgdb=fc2733d6468c073f1dd738aa598ede55&nocache=a9a895ad49f0f55c362d050dc1ad3fa3&dir=&wwwpkgdb=fc2733d6468c073f1dd738aa598ede55) | _ | hypothetical protein |
| [OCAR_6962](https://www.genoscope.cns.fr/agc/mage/wwwpkgdb/Info/getInfoLabel.php?id=3485475&wwwpkgdb=fc2733d6468c073f1dd738aa598ede55&nocache=a9a895ad49f0f55c362d050dc1ad3fa3&dir=&wwwpkgdb=fc2733d6468c073f1dd738aa598ede55) | _ | putative domain of unknown function |
| [OCAR_6964](https://www.genoscope.cns.fr/agc/mage/wwwpkgdb/Info/getInfoLabel.php?id=3483671&wwwpkgdb=fc2733d6468c073f1dd738aa598ede55&nocache=a9a895ad49f0f55c362d050dc1ad3fa3&dir=&wwwpkgdb=fc2733d6468c073f1dd738aa598ede55) | _ | hypothetical protein |
| [OCAR_6968](https://www.genoscope.cns.fr/agc/mage/wwwpkgdb/Info/getInfoLabel.php?id=3485477&wwwpkgdb=fc2733d6468c073f1dd738aa598ede55&nocache=a9a895ad49f0f55c362d050dc1ad3fa3&dir=&wwwpkgdb=fc2733d6468c073f1dd738aa598ede55) | _ | transcriptional regulator, GntR family |
| [OCAR_6977](https://www.genoscope.cns.fr/agc/mage/wwwpkgdb/Info/getInfoLabel.php?id=3485483&wwwpkgdb=fc2733d6468c073f1dd738aa598ede55&nocache=a9a895ad49f0f55c362d050dc1ad3fa3&dir=&wwwpkgdb=fc2733d6468c073f1dd738aa598ede55) | _ | hypothetical protein |
| [OCAR_2956](https://www.genoscope.cns.fr/agc/mage/wwwpkgdb/Info/getInfoLabel.php?id=3481939&wwwpkgdb=fc2733d6468c073f1dd738aa598ede55&nocache=a9a895ad49f0f55c362d050dc1ad3fa3&dir=&wwwpkgdb=fc2733d6468c073f1dd738aa598ede55) | _ | _ |
| [OCAR_2957](https://www.genoscope.cns.fr/agc/mage/wwwpkgdb/Info/getInfoLabel.php?id=3481938&wwwpkgdb=fc2733d6468c073f1dd738aa598ede55&nocache=a9a895ad49f0f55c362d050dc1ad3fa3&dir=&wwwpkgdb=fc2733d6468c073f1dd738aa598ede55) | _ | _ |
| [OCAR_7001](https://www.genoscope.cns.fr/agc/mage/wwwpkgdb/Info/getInfoLabel.php?id=3485493&wwwpkgdb=fc2733d6468c073f1dd738aa598ede55&nocache=a9a895ad49f0f55c362d050dc1ad3fa3&dir=&wwwpkgdb=fc2733d6468c073f1dd738aa598ede55) | _ | hypothetical protein |
| [OCAR_7013](https://www.genoscope.cns.fr/agc/mage/wwwpkgdb/Info/getInfoLabel.php?id=3485498&wwwpkgdb=fc2733d6468c073f1dd738aa598ede55&nocache=a9a895ad49f0f55c362d050dc1ad3fa3&dir=&wwwpkgdb=fc2733d6468c073f1dd738aa598ede55) | _ | hypothetical protein |
| [OCAR_7015](https://www.genoscope.cns.fr/agc/mage/wwwpkgdb/Info/getInfoLabel.php?id=3485499&wwwpkgdb=fc2733d6468c073f1dd738aa598ede55&nocache=a9a895ad49f0f55c362d050dc1ad3fa3&dir=&wwwpkgdb=fc2733d6468c073f1dd738aa598ede55) | _ | hypothetical protein |
| [OCAR_7032](https://www.genoscope.cns.fr/agc/mage/wwwpkgdb/Info/getInfoLabel.php?id=3483708&wwwpkgdb=fc2733d6468c073f1dd738aa598ede55&nocache=a9a895ad49f0f55c362d050dc1ad3fa3&dir=&wwwpkgdb=fc2733d6468c073f1dd738aa598ede55) | _ | hypothetical protein |
| [OCAR_3009](https://www.genoscope.cns.fr/agc/mage/wwwpkgdb/Info/getInfoLabel.php?id=3481937&wwwpkgdb=fc2733d6468c073f1dd738aa598ede55&nocache=a9a895ad49f0f55c362d050dc1ad3fa3&dir=&wwwpkgdb=fc2733d6468c073f1dd738aa598ede55) | _ | _ |
| [OCAR_7036](https://www.genoscope.cns.fr/agc/mage/wwwpkgdb/Info/getInfoLabel.php?id=3485508&wwwpkgdb=fc2733d6468c073f1dd738aa598ede55&nocache=a9a895ad49f0f55c362d050dc1ad3fa3&dir=&wwwpkgdb=fc2733d6468c073f1dd738aa598ede55) | _ | choloylglycine hydrolase |
| [OCAR_7037](https://www.genoscope.cns.fr/agc/mage/wwwpkgdb/Info/getInfoLabel.php?id=3483712&wwwpkgdb=fc2733d6468c073f1dd738aa598ede55&nocache=a9a895ad49f0f55c362d050dc1ad3fa3&dir=&wwwpkgdb=fc2733d6468c073f1dd738aa598ede55) | _ | hypothetical protein |
| [OCAR_3013](https://www.genoscope.cns.fr/agc/mage/wwwpkgdb/Info/getInfoLabel.php?id=3481936&wwwpkgdb=fc2733d6468c073f1dd738aa598ede55&nocache=a9a895ad49f0f55c362d050dc1ad3fa3&dir=&wwwpkgdb=fc2733d6468c073f1dd738aa598ede55) | _ | _ |
| [OCAR_3016](https://www.genoscope.cns.fr/agc/mage/wwwpkgdb/Info/getInfoLabel.php?id=3481935&wwwpkgdb=fc2733d6468c073f1dd738aa598ede55&nocache=a9a895ad49f0f55c362d050dc1ad3fa3&dir=&wwwpkgdb=fc2733d6468c073f1dd738aa598ede55) | _ | _ |
| [OCAR_7051](https://www.genoscope.cns.fr/agc/mage/wwwpkgdb/Info/getInfoLabel.php?id=3483719&wwwpkgdb=fc2733d6468c073f1dd738aa598ede55&nocache=a9a895ad49f0f55c362d050dc1ad3fa3&dir=&wwwpkgdb=fc2733d6468c073f1dd738aa598ede55) | _ | hypothetical protein |
| [OCAR_7056](https://www.genoscope.cns.fr/agc/mage/wwwpkgdb/Info/getInfoLabel.php?id=3485519&wwwpkgdb=fc2733d6468c073f1dd738aa598ede55&nocache=a9a895ad49f0f55c362d050dc1ad3fa3&dir=&wwwpkgdb=fc2733d6468c073f1dd738aa598ede55) | _ | excinuclease ABC, C subunit domain protein |
| [OCAR_7058](https://www.genoscope.cns.fr/agc/mage/wwwpkgdb/Info/getInfoLabel.php?id=3483721&wwwpkgdb=fc2733d6468c073f1dd738aa598ede55&nocache=a9a895ad49f0f55c362d050dc1ad3fa3&dir=&wwwpkgdb=fc2733d6468c073f1dd738aa598ede55) | _ | hypothetical protein |
| [OCAR_7062](https://www.genoscope.cns.fr/agc/mage/wwwpkgdb/Info/getInfoLabel.php?id=3483722&wwwpkgdb=fc2733d6468c073f1dd738aa598ede55&nocache=a9a895ad49f0f55c362d050dc1ad3fa3&dir=&wwwpkgdb=fc2733d6468c073f1dd738aa598ede55) | _ | hypothetical protein |
| [OCAR_7069](https://www.genoscope.cns.fr/agc/mage/wwwpkgdb/Info/getInfoLabel.php?id=3485525&wwwpkgdb=fc2733d6468c073f1dd738aa598ede55&nocache=a9a895ad49f0f55c362d050dc1ad3fa3&dir=&wwwpkgdb=fc2733d6468c073f1dd738aa598ede55) | _ | hypothetical protein |
| [OCAR_7073](https://www.genoscope.cns.fr/agc/mage/wwwpkgdb/Info/getInfoLabel.php?id=3485527&wwwpkgdb=fc2733d6468c073f1dd738aa598ede55&nocache=a9a895ad49f0f55c362d050dc1ad3fa3&dir=&wwwpkgdb=fc2733d6468c073f1dd738aa598ede55) | _ | hypothetical protein |
| [OCAR_7074](https://www.genoscope.cns.fr/agc/mage/wwwpkgdb/Info/getInfoLabel.php?id=3485528&wwwpkgdb=fc2733d6468c073f1dd738aa598ede55&nocache=a9a895ad49f0f55c362d050dc1ad3fa3&dir=&wwwpkgdb=fc2733d6468c073f1dd738aa598ede55) | prpB | methylisocitrate lyase |
| [OCAR_7076](https://www.genoscope.cns.fr/agc/mage/wwwpkgdb/Info/getInfoLabel.php?id=3485530&wwwpkgdb=fc2733d6468c073f1dd738aa598ede55&nocache=a9a895ad49f0f55c362d050dc1ad3fa3&dir=&wwwpkgdb=fc2733d6468c073f1dd738aa598ede55) | _ | 2-methylcitrate dehydratase 2 |
| [OCAR_7077](https://www.genoscope.cns.fr/agc/mage/wwwpkgdb/Info/getInfoLabel.php?id=3485531&wwwpkgdb=fc2733d6468c073f1dd738aa598ede55&nocache=a9a895ad49f0f55c362d050dc1ad3fa3&dir=&wwwpkgdb=fc2733d6468c073f1dd738aa598ede55) | _ | thioesterase superfamily |
| [OCAR_7078](https://www.genoscope.cns.fr/agc/mage/wwwpkgdb/Info/getInfoLabel.php?id=3485532&wwwpkgdb=fc2733d6468c073f1dd738aa598ede55&nocache=a9a895ad49f0f55c362d050dc1ad3fa3&dir=&wwwpkgdb=fc2733d6468c073f1dd738aa598ede55) | _ | auxin Efflux Carrier |
| [OCAR_7079](https://www.genoscope.cns.fr/agc/mage/wwwpkgdb/Info/getInfoLabel.php?id=3483730&wwwpkgdb=fc2733d6468c073f1dd738aa598ede55&nocache=a9a895ad49f0f55c362d050dc1ad3fa3&dir=&wwwpkgdb=fc2733d6468c073f1dd738aa598ede55) | _ | major facilitator family transporter |
| [OCAR_7080](https://www.genoscope.cns.fr/agc/mage/wwwpkgdb/Info/getInfoLabel.php?id=3485533&wwwpkgdb=fc2733d6468c073f1dd738aa598ede55&nocache=a9a895ad49f0f55c362d050dc1ad3fa3&dir=&wwwpkgdb=fc2733d6468c073f1dd738aa598ede55) | _ | glycosyltransferase |
| [OCAR_7081](https://www.genoscope.cns.fr/agc/mage/wwwpkgdb/Info/getInfoLabel.php?id=3485534&wwwpkgdb=fc2733d6468c073f1dd738aa598ede55&nocache=a9a895ad49f0f55c362d050dc1ad3fa3&dir=&wwwpkgdb=fc2733d6468c073f1dd738aa598ede55) | _ | polysaccharide biosynthesis protein |
| [OCAR_7082](https://www.genoscope.cns.fr/agc/mage/wwwpkgdb/Info/getInfoLabel.php?id=3485535&wwwpkgdb=fc2733d6468c073f1dd738aa598ede55&nocache=a9a895ad49f0f55c362d050dc1ad3fa3&dir=&wwwpkgdb=fc2733d6468c073f1dd738aa598ede55) | _ | cellulose synthesis regulatory protein |
| [OCAR_7083](https://www.genoscope.cns.fr/agc/mage/wwwpkgdb/Info/getInfoLabel.php?id=3485536&wwwpkgdb=fc2733d6468c073f1dd738aa598ede55&nocache=a9a895ad49f0f55c362d050dc1ad3fa3&dir=&wwwpkgdb=fc2733d6468c073f1dd738aa598ede55) | _ | hypothetical protein |
| [OCAR_7084](https://www.genoscope.cns.fr/agc/mage/wwwpkgdb/Info/getInfoLabel.php?id=3485537&wwwpkgdb=fc2733d6468c073f1dd738aa598ede55&nocache=a9a895ad49f0f55c362d050dc1ad3fa3&dir=&wwwpkgdb=fc2733d6468c073f1dd738aa598ede55) | _ | hypothetical protein |
| [OCAR_7087](https://www.genoscope.cns.fr/agc/mage/wwwpkgdb/Info/getInfoLabel.php?id=3485540&wwwpkgdb=fc2733d6468c073f1dd738aa598ede55&nocache=a9a895ad49f0f55c362d050dc1ad3fa3&dir=&wwwpkgdb=fc2733d6468c073f1dd738aa598ede55) | _ | AmiS/UreI transporter |
| [OCAR_7093](https://www.genoscope.cns.fr/agc/mage/wwwpkgdb/Info/getInfoLabel.php?id=3485545&wwwpkgdb=fc2733d6468c073f1dd738aa598ede55&nocache=a9a895ad49f0f55c362d050dc1ad3fa3&dir=&wwwpkgdb=fc2733d6468c073f1dd738aa598ede55) | _ | alpha/beta hydrolase fold protein |
| [OCAR_7094](https://www.genoscope.cns.fr/agc/mage/wwwpkgdb/Info/getInfoLabel.php?id=3485546&wwwpkgdb=fc2733d6468c073f1dd738aa598ede55&nocache=a9a895ad49f0f55c362d050dc1ad3fa3&dir=&wwwpkgdb=fc2733d6468c073f1dd738aa598ede55) | _ | hypothetical protein |
| [OCAR_7099](https://www.genoscope.cns.fr/agc/mage/wwwpkgdb/Info/getInfoLabel.php?id=3485550&wwwpkgdb=fc2733d6468c073f1dd738aa598ede55&nocache=a9a895ad49f0f55c362d050dc1ad3fa3&dir=&wwwpkgdb=fc2733d6468c073f1dd738aa598ede55) | _ | hypothetical protein |
| [OCAR_7102](https://www.genoscope.cns.fr/agc/mage/wwwpkgdb/Info/getInfoLabel.php?id=3483734&wwwpkgdb=fc2733d6468c073f1dd738aa598ede55&nocache=a9a895ad49f0f55c362d050dc1ad3fa3&dir=&wwwpkgdb=fc2733d6468c073f1dd738aa598ede55) | _ | hypothetical protein |
| [OCAR_3079](https://www.genoscope.cns.fr/agc/mage/wwwpkgdb/Info/getInfoLabel.php?id=3481934&wwwpkgdb=fc2733d6468c073f1dd738aa598ede55&nocache=a9a895ad49f0f55c362d050dc1ad3fa3&dir=&wwwpkgdb=fc2733d6468c073f1dd738aa598ede55) | _ | _ |
| [OCAR_7128](https://www.genoscope.cns.fr/agc/mage/wwwpkgdb/Info/getInfoLabel.php?id=3483747&wwwpkgdb=fc2733d6468c073f1dd738aa598ede55&nocache=a9a895ad49f0f55c362d050dc1ad3fa3&dir=&wwwpkgdb=fc2733d6468c073f1dd738aa598ede55) | _ | hypothetical protein |
| [OCAR_7129](https://www.genoscope.cns.fr/agc/mage/wwwpkgdb/Info/getInfoLabel.php?id=3483748&wwwpkgdb=fc2733d6468c073f1dd738aa598ede55&nocache=a9a895ad49f0f55c362d050dc1ad3fa3&dir=&wwwpkgdb=fc2733d6468c073f1dd738aa598ede55) | _ | glycosyl transferase, group 1 |
| [OCAR_7130](https://www.genoscope.cns.fr/agc/mage/wwwpkgdb/Info/getInfoLabel.php?id=3485565&wwwpkgdb=fc2733d6468c073f1dd738aa598ede55&nocache=a9a895ad49f0f55c362d050dc1ad3fa3&dir=&wwwpkgdb=fc2733d6468c073f1dd738aa598ede55) | _ | hypothetical protein |
| [OCAR_7137](https://www.genoscope.cns.fr/agc/mage/wwwpkgdb/Info/getInfoLabel.php?id=3485567&wwwpkgdb=fc2733d6468c073f1dd738aa598ede55&nocache=a9a895ad49f0f55c362d050dc1ad3fa3&dir=&wwwpkgdb=fc2733d6468c073f1dd738aa598ede55) | _ | hypothetical protein |
| [OCAR_7140](https://www.genoscope.cns.fr/agc/mage/wwwpkgdb/Info/getInfoLabel.php?id=3483756&wwwpkgdb=fc2733d6468c073f1dd738aa598ede55&nocache=a9a895ad49f0f55c362d050dc1ad3fa3&dir=&wwwpkgdb=fc2733d6468c073f1dd738aa598ede55) | ccoQ | cytochrome c oxidase, Cbb3-type, CcoQ subunit |
| [OCAR_7142](https://www.genoscope.cns.fr/agc/mage/wwwpkgdb/Info/getInfoLabel.php?id=3483758&wwwpkgdb=fc2733d6468c073f1dd738aa598ede55&nocache=a9a895ad49f0f55c362d050dc1ad3fa3&dir=&wwwpkgdb=fc2733d6468c073f1dd738aa598ede55) | _ | hypothetical protein |
| [OCAR_7150](https://www.genoscope.cns.fr/agc/mage/wwwpkgdb/Info/getInfoLabel.php?id=3485570&wwwpkgdb=fc2733d6468c073f1dd738aa598ede55&nocache=a9a895ad49f0f55c362d050dc1ad3fa3&dir=&wwwpkgdb=fc2733d6468c073f1dd738aa598ede55) | _ | hypothetical protein |
| [OCAR_7151](https://www.genoscope.cns.fr/agc/mage/wwwpkgdb/Info/getInfoLabel.php?id=3485571&wwwpkgdb=fc2733d6468c073f1dd738aa598ede55&nocache=a9a895ad49f0f55c362d050dc1ad3fa3&dir=&wwwpkgdb=fc2733d6468c073f1dd738aa598ede55) | _ | hypothetical protein |
| [OCAR_7159](https://www.genoscope.cns.fr/agc/mage/wwwpkgdb/Info/getInfoLabel.php?id=3483766&wwwpkgdb=fc2733d6468c073f1dd738aa598ede55&nocache=a9a895ad49f0f55c362d050dc1ad3fa3&dir=&wwwpkgdb=fc2733d6468c073f1dd738aa598ede55) | _ | hypothetical protein |
| [OCAR_7160](https://www.genoscope.cns.fr/agc/mage/wwwpkgdb/Info/getInfoLabel.php?id=3483767&wwwpkgdb=fc2733d6468c073f1dd738aa598ede55&nocache=a9a895ad49f0f55c362d050dc1ad3fa3&dir=&wwwpkgdb=fc2733d6468c073f1dd738aa598ede55) | _ | transporter, dme family |
| [OCAR_7165](https://www.genoscope.cns.fr/agc/mage/wwwpkgdb/Info/getInfoLabel.php?id=3483768&wwwpkgdb=fc2733d6468c073f1dd738aa598ede55&nocache=a9a895ad49f0f55c362d050dc1ad3fa3&dir=&wwwpkgdb=fc2733d6468c073f1dd738aa598ede55) | _ | hypothetical protein |
| [OCAR_7176](https://www.genoscope.cns.fr/agc/mage/wwwpkgdb/Info/getInfoLabel.php?id=3483772&wwwpkgdb=fc2733d6468c073f1dd738aa598ede55&nocache=a9a895ad49f0f55c362d050dc1ad3fa3&dir=&wwwpkgdb=fc2733d6468c073f1dd738aa598ede55) | _ | hypothetical protein |
| [OCAR_3143](https://www.genoscope.cns.fr/agc/mage/wwwpkgdb/Info/getInfoLabel.php?id=3481933&wwwpkgdb=fc2733d6468c073f1dd738aa598ede55&nocache=a9a895ad49f0f55c362d050dc1ad3fa3&dir=&wwwpkgdb=fc2733d6468c073f1dd738aa598ede55) | _ | _ |
| [OCAR_7179](https://www.genoscope.cns.fr/agc/mage/wwwpkgdb/Info/getInfoLabel.php?id=3483774&wwwpkgdb=fc2733d6468c073f1dd738aa598ede55&nocache=a9a895ad49f0f55c362d050dc1ad3fa3&dir=&wwwpkgdb=fc2733d6468c073f1dd738aa598ede55) | _ | hypothetical protein |
| [OCAR_7182](https://www.genoscope.cns.fr/agc/mage/wwwpkgdb/Info/getInfoLabel.php?id=3483775&wwwpkgdb=fc2733d6468c073f1dd738aa598ede55&nocache=a9a895ad49f0f55c362d050dc1ad3fa3&dir=&wwwpkgdb=fc2733d6468c073f1dd738aa598ede55) | _ | hypothetical protein |
| [OCAR_3157](https://www.genoscope.cns.fr/agc/mage/wwwpkgdb/Info/getInfoLabel.php?id=3482125&wwwpkgdb=fc2733d6468c073f1dd738aa598ede55&nocache=a9a895ad49f0f55c362d050dc1ad3fa3&dir=&wwwpkgdb=fc2733d6468c073f1dd738aa598ede55) | _ | _ |
| [OCAR_7198](https://www.genoscope.cns.fr/agc/mage/wwwpkgdb/Info/getInfoLabel.php?id=3483786&wwwpkgdb=fc2733d6468c073f1dd738aa598ede55&nocache=a9a895ad49f0f55c362d050dc1ad3fa3&dir=&wwwpkgdb=fc2733d6468c073f1dd738aa598ede55) | _ | hypothetical protein |
| [OCAR_7204](https://www.genoscope.cns.fr/agc/mage/wwwpkgdb/Info/getInfoLabel.php?id=3485599&wwwpkgdb=fc2733d6468c073f1dd738aa598ede55&nocache=a9a895ad49f0f55c362d050dc1ad3fa3&dir=&wwwpkgdb=fc2733d6468c073f1dd738aa598ede55) | _ | hypothetical protein |
| [OCAR_7207](https://www.genoscope.cns.fr/agc/mage/wwwpkgdb/Info/getInfoLabel.php?id=3485601&wwwpkgdb=fc2733d6468c073f1dd738aa598ede55&nocache=a9a895ad49f0f55c362d050dc1ad3fa3&dir=&wwwpkgdb=fc2733d6468c073f1dd738aa598ede55) | _ | hypothetical protein |
| [OCAR_7225](https://www.genoscope.cns.fr/agc/mage/wwwpkgdb/Info/getInfoLabel.php?id=3485616&wwwpkgdb=fc2733d6468c073f1dd738aa598ede55&nocache=a9a895ad49f0f55c362d050dc1ad3fa3&dir=&wwwpkgdb=fc2733d6468c073f1dd738aa598ede55) | _ | hypothetical protein |
| [OCAR_7233](https://www.genoscope.cns.fr/agc/mage/wwwpkgdb/Info/getInfoLabel.php?id=3485620&wwwpkgdb=fc2733d6468c073f1dd738aa598ede55&nocache=a9a895ad49f0f55c362d050dc1ad3fa3&dir=&wwwpkgdb=fc2733d6468c073f1dd738aa598ede55) | _ | hypothetical protein |
| [OCAR_3204](https://www.genoscope.cns.fr/agc/mage/wwwpkgdb/Info/getInfoLabel.php?id=3481932&wwwpkgdb=fc2733d6468c073f1dd738aa598ede55&nocache=a9a895ad49f0f55c362d050dc1ad3fa3&dir=&wwwpkgdb=fc2733d6468c073f1dd738aa598ede55) | _ | _ |
| [OCAR_7247](https://www.genoscope.cns.fr/agc/mage/wwwpkgdb/Info/getInfoLabel.php?id=3485626&wwwpkgdb=fc2733d6468c073f1dd738aa598ede55&nocache=a9a895ad49f0f55c362d050dc1ad3fa3&dir=&wwwpkgdb=fc2733d6468c073f1dd738aa598ede55) | _ | hypothetical protein |
| [OCAR_7279](https://www.genoscope.cns.fr/agc/mage/wwwpkgdb/Info/getInfoLabel.php?id=3483822&wwwpkgdb=fc2733d6468c073f1dd738aa598ede55&nocache=a9a895ad49f0f55c362d050dc1ad3fa3&dir=&wwwpkgdb=fc2733d6468c073f1dd738aa598ede55) | _ | hypothetical protein |
| [OCAR_3251](https://www.genoscope.cns.fr/agc/mage/wwwpkgdb/Info/getInfoLabel.php?id=3481931&wwwpkgdb=fc2733d6468c073f1dd738aa598ede55&nocache=a9a895ad49f0f55c362d050dc1ad3fa3&dir=&wwwpkgdb=fc2733d6468c073f1dd738aa598ede55) | _ | _ |
| [OCAR_7294](https://www.genoscope.cns.fr/agc/mage/wwwpkgdb/Info/getInfoLabel.php?id=3483823&wwwpkgdb=fc2733d6468c073f1dd738aa598ede55&nocache=a9a895ad49f0f55c362d050dc1ad3fa3&dir=&wwwpkgdb=fc2733d6468c073f1dd738aa598ede55) | _ | hypothetical protein |
| [OCAR_7300](https://www.genoscope.cns.fr/agc/mage/wwwpkgdb/Info/getInfoLabel.php?id=3485660&wwwpkgdb=fc2733d6468c073f1dd738aa598ede55&nocache=a9a895ad49f0f55c362d050dc1ad3fa3&dir=&wwwpkgdb=fc2733d6468c073f1dd738aa598ede55) | _ | hypothetical protein |
| [OCAR_7304](https://www.genoscope.cns.fr/agc/mage/wwwpkgdb/Info/getInfoLabel.php?id=3485664&wwwpkgdb=fc2733d6468c073f1dd738aa598ede55&nocache=a9a895ad49f0f55c362d050dc1ad3fa3&dir=&wwwpkgdb=fc2733d6468c073f1dd738aa598ede55) | _ | hypothetical protein |
| [OCAR_3271](https://www.genoscope.cns.fr/agc/mage/wwwpkgdb/Info/getInfoLabel.php?id=3481930&wwwpkgdb=fc2733d6468c073f1dd738aa598ede55&nocache=a9a895ad49f0f55c362d050dc1ad3fa3&dir=&wwwpkgdb=fc2733d6468c073f1dd738aa598ede55) | _ | _ |
| [OCAR_3294](https://www.genoscope.cns.fr/agc/mage/wwwpkgdb/Info/getInfoLabel.php?id=3481929&wwwpkgdb=fc2733d6468c073f1dd738aa598ede55&nocache=a9a895ad49f0f55c362d050dc1ad3fa3&dir=&wwwpkgdb=fc2733d6468c073f1dd738aa598ede55) | _ | _ |
| [OCAR_7335](https://www.genoscope.cns.fr/agc/mage/wwwpkgdb/Info/getInfoLabel.php?id=3485679&wwwpkgdb=fc2733d6468c073f1dd738aa598ede55&nocache=a9a895ad49f0f55c362d050dc1ad3fa3&dir=&wwwpkgdb=fc2733d6468c073f1dd738aa598ede55) | _ | hypothetical protein |
| [OCAR_3306](https://www.genoscope.cns.fr/agc/mage/wwwpkgdb/Info/getInfoLabel.php?id=3481928&wwwpkgdb=fc2733d6468c073f1dd738aa598ede55&nocache=a9a895ad49f0f55c362d050dc1ad3fa3&dir=&wwwpkgdb=fc2733d6468c073f1dd738aa598ede55) | _ | _ |
| [OCAR_7346](https://www.genoscope.cns.fr/agc/mage/wwwpkgdb/Info/getInfoLabel.php?id=3483844&wwwpkgdb=fc2733d6468c073f1dd738aa598ede55&nocache=a9a895ad49f0f55c362d050dc1ad3fa3&dir=&wwwpkgdb=fc2733d6468c073f1dd738aa598ede55) | ccoQ | cytochrome c oxidase, Cbb3-type, CcoQ subunit |
| [OCAR_7356](https://www.genoscope.cns.fr/agc/mage/wwwpkgdb/Info/getInfoLabel.php?id=3485689&wwwpkgdb=fc2733d6468c073f1dd738aa598ede55&nocache=a9a895ad49f0f55c362d050dc1ad3fa3&dir=&wwwpkgdb=fc2733d6468c073f1dd738aa598ede55) | _ | peptidase family M20/M25/M40 |
| [OCAR_7357](https://www.genoscope.cns.fr/agc/mage/wwwpkgdb/Info/getInfoLabel.php?id=3485690&wwwpkgdb=fc2733d6468c073f1dd738aa598ede55&nocache=a9a895ad49f0f55c362d050dc1ad3fa3&dir=&wwwpkgdb=fc2733d6468c073f1dd738aa598ede55) | _ | diaminopropionate ammonia-lyase |
| [OCAR_7362](https://www.genoscope.cns.fr/agc/mage/wwwpkgdb/Info/getInfoLabel.php?id=3485695&wwwpkgdb=fc2733d6468c073f1dd738aa598ede55&nocache=a9a895ad49f0f55c362d050dc1ad3fa3&dir=&wwwpkgdb=fc2733d6468c073f1dd738aa598ede55) | _ | putative membrane lipoprotein lipid attachment site |
| [OCAR_7364](https://www.genoscope.cns.fr/agc/mage/wwwpkgdb/Info/getInfoLabel.php?id=3485696&wwwpkgdb=fc2733d6468c073f1dd738aa598ede55&nocache=a9a895ad49f0f55c362d050dc1ad3fa3&dir=&wwwpkgdb=fc2733d6468c073f1dd738aa598ede55) | _ | hypothetical protein |
| [OCAR_7365](https://www.genoscope.cns.fr/agc/mage/wwwpkgdb/Info/getInfoLabel.php?id=3483852&wwwpkgdb=fc2733d6468c073f1dd738aa598ede55&nocache=a9a895ad49f0f55c362d050dc1ad3fa3&dir=&wwwpkgdb=fc2733d6468c073f1dd738aa598ede55) | _ | membrane lipoprotein lipid attachment site |
| [OCAR_7367](https://www.genoscope.cns.fr/agc/mage/wwwpkgdb/Info/getInfoLabel.php?id=3483854&wwwpkgdb=fc2733d6468c073f1dd738aa598ede55&nocache=a9a895ad49f0f55c362d050dc1ad3fa3&dir=&wwwpkgdb=fc2733d6468c073f1dd738aa598ede55) | xdhB | xanthine dehydrogenase, molybdopterin binding subunit |
| [OCAR_7368](https://www.genoscope.cns.fr/agc/mage/wwwpkgdb/Info/getInfoLabel.php?id=3483855&wwwpkgdb=fc2733d6468c073f1dd738aa598ede55&nocache=a9a895ad49f0f55c362d050dc1ad3fa3&dir=&wwwpkgdb=fc2733d6468c073f1dd738aa598ede55) | xdhC | xanthine dehydrogenase accessory protein XdhC |
| [OCAR_3333](https://www.genoscope.cns.fr/agc/mage/wwwpkgdb/Info/getInfoLabel.php?id=3482124&wwwpkgdb=fc2733d6468c073f1dd738aa598ede55&nocache=a9a895ad49f0f55c362d050dc1ad3fa3&dir=&wwwpkgdb=fc2733d6468c073f1dd738aa598ede55) | _ | _ |
| [OCAR_7376](https://www.genoscope.cns.fr/agc/mage/wwwpkgdb/Info/getInfoLabel.php?id=3485702&wwwpkgdb=fc2733d6468c073f1dd738aa598ede55&nocache=a9a895ad49f0f55c362d050dc1ad3fa3&dir=&wwwpkgdb=fc2733d6468c073f1dd738aa598ede55) | _ | hypothetical protein |
| [OCAR_3349](https://www.genoscope.cns.fr/agc/mage/wwwpkgdb/Info/getInfoLabel.php?id=3481927&wwwpkgdb=fc2733d6468c073f1dd738aa598ede55&nocache=a9a895ad49f0f55c362d050dc1ad3fa3&dir=&wwwpkgdb=fc2733d6468c073f1dd738aa598ede55) | _ | _ |
| [OCAR_3352](https://www.genoscope.cns.fr/agc/mage/wwwpkgdb/Info/getInfoLabel.php?id=3482123&wwwpkgdb=fc2733d6468c073f1dd738aa598ede55&nocache=a9a895ad49f0f55c362d050dc1ad3fa3&dir=&wwwpkgdb=fc2733d6468c073f1dd738aa598ede55) | _ | _ |
| [OCAR_7392](https://www.genoscope.cns.fr/agc/mage/wwwpkgdb/Info/getInfoLabel.php?id=3485711&wwwpkgdb=fc2733d6468c073f1dd738aa598ede55&nocache=a9a895ad49f0f55c362d050dc1ad3fa3&dir=&wwwpkgdb=fc2733d6468c073f1dd738aa598ede55) | _ | hypothetical protein |
| [OCAR_7394](https://www.genoscope.cns.fr/agc/mage/wwwpkgdb/Info/getInfoLabel.php?id=3485712&wwwpkgdb=fc2733d6468c073f1dd738aa598ede55&nocache=a9a895ad49f0f55c362d050dc1ad3fa3&dir=&wwwpkgdb=fc2733d6468c073f1dd738aa598ede55) | _ | hypothetical protein |
| [OCAR_7398](https://www.genoscope.cns.fr/agc/mage/wwwpkgdb/Info/getInfoLabel.php?id=3485716&wwwpkgdb=fc2733d6468c073f1dd738aa598ede55&nocache=a9a895ad49f0f55c362d050dc1ad3fa3&dir=&wwwpkgdb=fc2733d6468c073f1dd738aa598ede55) | _ | hypothetical protein |
| [OCAR_7400](https://www.genoscope.cns.fr/agc/mage/wwwpkgdb/Info/getInfoLabel.php?id=3483867&wwwpkgdb=fc2733d6468c073f1dd738aa598ede55&nocache=a9a895ad49f0f55c362d050dc1ad3fa3&dir=&wwwpkgdb=fc2733d6468c073f1dd738aa598ede55) | _ | hypothetical protein |
| [OCAR_7404](https://www.genoscope.cns.fr/agc/mage/wwwpkgdb/Info/getInfoLabel.php?id=3483870&wwwpkgdb=fc2733d6468c073f1dd738aa598ede55&nocache=a9a895ad49f0f55c362d050dc1ad3fa3&dir=&wwwpkgdb=fc2733d6468c073f1dd738aa598ede55) | _ | hypothetical protein |
| [OCAR_7406](https://www.genoscope.cns.fr/agc/mage/wwwpkgdb/Info/getInfoLabel.php?id=3483871&wwwpkgdb=fc2733d6468c073f1dd738aa598ede55&nocache=a9a895ad49f0f55c362d050dc1ad3fa3&dir=&wwwpkgdb=fc2733d6468c073f1dd738aa598ede55) | _ | hypothetical protein |
| [OCAR_7409](https://www.genoscope.cns.fr/agc/mage/wwwpkgdb/Info/getInfoLabel.php?id=3485720&wwwpkgdb=fc2733d6468c073f1dd738aa598ede55&nocache=a9a895ad49f0f55c362d050dc1ad3fa3&dir=&wwwpkgdb=fc2733d6468c073f1dd738aa598ede55) | _ | hypothetical protein |
| [OCAR_7410](https://www.genoscope.cns.fr/agc/mage/wwwpkgdb/Info/getInfoLabel.php?id=3485721&wwwpkgdb=fc2733d6468c073f1dd738aa598ede55&nocache=a9a895ad49f0f55c362d050dc1ad3fa3&dir=&wwwpkgdb=fc2733d6468c073f1dd738aa598ede55) | _ | hypothetical protein |
| [OCAR_3372](https://www.genoscope.cns.fr/agc/mage/wwwpkgdb/Info/getInfoLabel.php?id=3482122&wwwpkgdb=fc2733d6468c073f1dd738aa598ede55&nocache=a9a895ad49f0f55c362d050dc1ad3fa3&dir=&wwwpkgdb=fc2733d6468c073f1dd738aa598ede55) | _ | _ |
| [OCAR_3379](https://www.genoscope.cns.fr/agc/mage/wwwpkgdb/Info/getInfoLabel.php?id=3482121&wwwpkgdb=fc2733d6468c073f1dd738aa598ede55&nocache=a9a895ad49f0f55c362d050dc1ad3fa3&dir=&wwwpkgdb=fc2733d6468c073f1dd738aa598ede55) | _ | _ |
| [OCAR_7433](https://www.genoscope.cns.fr/agc/mage/wwwpkgdb/Info/getInfoLabel.php?id=3483886&wwwpkgdb=fc2733d6468c073f1dd738aa598ede55&nocache=a9a895ad49f0f55c362d050dc1ad3fa3&dir=&wwwpkgdb=fc2733d6468c073f1dd738aa598ede55) | _ | hypothetical protein |
| [OCAR_7445](https://www.genoscope.cns.fr/agc/mage/wwwpkgdb/Info/getInfoLabel.php?id=3483894&wwwpkgdb=fc2733d6468c073f1dd738aa598ede55&nocache=a9a895ad49f0f55c362d050dc1ad3fa3&dir=&wwwpkgdb=fc2733d6468c073f1dd738aa598ede55) | _ | hypothetical protein |
| [OCAR_7446](https://www.genoscope.cns.fr/agc/mage/wwwpkgdb/Info/getInfoLabel.php?id=3483895&wwwpkgdb=fc2733d6468c073f1dd738aa598ede55&nocache=a9a895ad49f0f55c362d050dc1ad3fa3&dir=&wwwpkgdb=fc2733d6468c073f1dd738aa598ede55) | _ | phage transcriptional regulator, AlpA |
| [OCAR_7447](https://www.genoscope.cns.fr/agc/mage/wwwpkgdb/Info/getInfoLabel.php?id=3483896&wwwpkgdb=fc2733d6468c073f1dd738aa598ede55&nocache=a9a895ad49f0f55c362d050dc1ad3fa3&dir=&wwwpkgdb=fc2733d6468c073f1dd738aa598ede55) | _ | phage integrase |
| [OCAR_7448](https://www.genoscope.cns.fr/agc/mage/wwwpkgdb/Info/getInfoLabel.php?id=3483897&wwwpkgdb=fc2733d6468c073f1dd738aa598ede55&nocache=a9a895ad49f0f55c362d050dc1ad3fa3&dir=&wwwpkgdb=fc2733d6468c073f1dd738aa598ede55) | _ | phage integrase |
| [OCAR_3413](https://www.genoscope.cns.fr/agc/mage/wwwpkgdb/Info/getInfoLabel.php?id=3481925&wwwpkgdb=fc2733d6468c073f1dd738aa598ede55&nocache=a9a895ad49f0f55c362d050dc1ad3fa3&dir=&wwwpkgdb=fc2733d6468c073f1dd738aa598ede55) | _ | _ |
| [OCAR_3414](https://www.genoscope.cns.fr/agc/mage/wwwpkgdb/Info/getInfoLabel.php?id=3481924&wwwpkgdb=fc2733d6468c073f1dd738aa598ede55&nocache=a9a895ad49f0f55c362d050dc1ad3fa3&dir=&wwwpkgdb=fc2733d6468c073f1dd738aa598ede55) | _ | _ |
| [OCAR_7451](https://www.genoscope.cns.fr/agc/mage/wwwpkgdb/Info/getInfoLabel.php?id=3483898&wwwpkgdb=fc2733d6468c073f1dd738aa598ede55&nocache=a9a895ad49f0f55c362d050dc1ad3fa3&dir=&wwwpkgdb=fc2733d6468c073f1dd738aa598ede55) | _ | integral membrane protein |
| [OCAR_3416](https://www.genoscope.cns.fr/agc/mage/wwwpkgdb/Info/getInfoLabel.php?id=3481923&wwwpkgdb=fc2733d6468c073f1dd738aa598ede55&nocache=a9a895ad49f0f55c362d050dc1ad3fa3&dir=&wwwpkgdb=fc2733d6468c073f1dd738aa598ede55) | _ | _ |
| [OCAR_7455](https://www.genoscope.cns.fr/agc/mage/wwwpkgdb/Info/getInfoLabel.php?id=3483899&wwwpkgdb=fc2733d6468c073f1dd738aa598ede55&nocache=a9a895ad49f0f55c362d050dc1ad3fa3&dir=&wwwpkgdb=fc2733d6468c073f1dd738aa598ede55) | _ | hypothetical protein |
| [OCAR_3420](https://www.genoscope.cns.fr/agc/mage/wwwpkgdb/Info/getInfoLabel.php?id=3481922&wwwpkgdb=fc2733d6468c073f1dd738aa598ede55&nocache=a9a895ad49f0f55c362d050dc1ad3fa3&dir=&wwwpkgdb=fc2733d6468c073f1dd738aa598ede55) | _ | _ |
| [OCAR_7462](https://www.genoscope.cns.fr/agc/mage/wwwpkgdb/Info/getInfoLabel.php?id=3485745&wwwpkgdb=fc2733d6468c073f1dd738aa598ede55&nocache=a9a895ad49f0f55c362d050dc1ad3fa3&dir=&wwwpkgdb=fc2733d6468c073f1dd738aa598ede55) | _ | 5-methyltetrahydropteroyltriglutamate-- homocystei ne methyltransferase |
| [OCAR_7465](https://www.genoscope.cns.fr/agc/mage/wwwpkgdb/Info/getInfoLabel.php?id=3485748&wwwpkgdb=fc2733d6468c073f1dd738aa598ede55&nocache=a9a895ad49f0f55c362d050dc1ad3fa3&dir=&wwwpkgdb=fc2733d6468c073f1dd738aa598ede55) | _ | HtxB |
| [OCAR_7466](https://www.genoscope.cns.fr/agc/mage/wwwpkgdb/Info/getInfoLabel.php?id=3485749&wwwpkgdb=fc2733d6468c073f1dd738aa598ede55&nocache=a9a895ad49f0f55c362d050dc1ad3fa3&dir=&wwwpkgdb=fc2733d6468c073f1dd738aa598ede55) | _ | hypothetical protein |
| [OCAR_7467](https://www.genoscope.cns.fr/agc/mage/wwwpkgdb/Info/getInfoLabel.php?id=3485750&wwwpkgdb=fc2733d6468c073f1dd738aa598ede55&nocache=a9a895ad49f0f55c362d050dc1ad3fa3&dir=&wwwpkgdb=fc2733d6468c073f1dd738aa598ede55) | _ | hypothetical protein |
| [OCAR_7468](https://www.genoscope.cns.fr/agc/mage/wwwpkgdb/Info/getInfoLabel.php?id=3485751&wwwpkgdb=fc2733d6468c073f1dd738aa598ede55&nocache=a9a895ad49f0f55c362d050dc1ad3fa3&dir=&wwwpkgdb=fc2733d6468c073f1dd738aa598ede55) | _ | SirA domain protein |
| [OCAR_7474](https://www.genoscope.cns.fr/agc/mage/wwwpkgdb/Info/getInfoLabel.php?id=3485757&wwwpkgdb=fc2733d6468c073f1dd738aa598ede55&nocache=a9a895ad49f0f55c362d050dc1ad3fa3&dir=&wwwpkgdb=fc2733d6468c073f1dd738aa598ede55) | _ | twin-arginine translocation pathway signal |
| [OCAR_7476](https://www.genoscope.cns.fr/agc/mage/wwwpkgdb/Info/getInfoLabel.php?id=3485758&wwwpkgdb=fc2733d6468c073f1dd738aa598ede55&nocache=a9a895ad49f0f55c362d050dc1ad3fa3&dir=&wwwpkgdb=fc2733d6468c073f1dd738aa598ede55) | _ | hypothetical protein |
| [OCAR_7477](https://www.genoscope.cns.fr/agc/mage/wwwpkgdb/Info/getInfoLabel.php?id=3485759&wwwpkgdb=fc2733d6468c073f1dd738aa598ede55&nocache=a9a895ad49f0f55c362d050dc1ad3fa3&dir=&wwwpkgdb=fc2733d6468c073f1dd738aa598ede55) | _ | hypothetical protein |
| [OCAR_7478](https://www.genoscope.cns.fr/agc/mage/wwwpkgdb/Info/getInfoLabel.php?id=3485760&wwwpkgdb=fc2733d6468c073f1dd738aa598ede55&nocache=a9a895ad49f0f55c362d050dc1ad3fa3&dir=&wwwpkgdb=fc2733d6468c073f1dd738aa598ede55) | _ | hypothetical protein |
| [OCAR_7481](https://www.genoscope.cns.fr/agc/mage/wwwpkgdb/Info/getInfoLabel.php?id=3485763&wwwpkgdb=fc2733d6468c073f1dd738aa598ede55&nocache=a9a895ad49f0f55c362d050dc1ad3fa3&dir=&wwwpkgdb=fc2733d6468c073f1dd738aa598ede55) | _ | hypothetical protein |
| [OCAR_7487](https://www.genoscope.cns.fr/agc/mage/wwwpkgdb/Info/getInfoLabel.php?id=3483904&wwwpkgdb=fc2733d6468c073f1dd738aa598ede55&nocache=a9a895ad49f0f55c362d050dc1ad3fa3&dir=&wwwpkgdb=fc2733d6468c073f1dd738aa598ede55) | _ | hypothetical protein |
| [OCAR_7488](https://www.genoscope.cns.fr/agc/mage/wwwpkgdb/Info/getInfoLabel.php?id=3485767&wwwpkgdb=fc2733d6468c073f1dd738aa598ede55&nocache=a9a895ad49f0f55c362d050dc1ad3fa3&dir=&wwwpkgdb=fc2733d6468c073f1dd738aa598ede55) | _ | asparagine synthase family |
| [OCAR_7489](https://www.genoscope.cns.fr/agc/mage/wwwpkgdb/Info/getInfoLabel.php?id=3483905&wwwpkgdb=fc2733d6468c073f1dd738aa598ede55&nocache=a9a895ad49f0f55c362d050dc1ad3fa3&dir=&wwwpkgdb=fc2733d6468c073f1dd738aa598ede55) | _ | hypothetical protein |
| [OCAR_3453](https://www.genoscope.cns.fr/agc/mage/wwwpkgdb/Info/getInfoLabel.php?id=3481921&wwwpkgdb=fc2733d6468c073f1dd738aa598ede55&nocache=a9a895ad49f0f55c362d050dc1ad3fa3&dir=&wwwpkgdb=fc2733d6468c073f1dd738aa598ede55) | _ | _ |
| [OCAR_7491](https://www.genoscope.cns.fr/agc/mage/wwwpkgdb/Info/getInfoLabel.php?id=3485769&wwwpkgdb=fc2733d6468c073f1dd738aa598ede55&nocache=a9a895ad49f0f55c362d050dc1ad3fa3&dir=&wwwpkgdb=fc2733d6468c073f1dd738aa598ede55) | _ | hypothetical protein |
| [OCAR_7493](https://www.genoscope.cns.fr/agc/mage/wwwpkgdb/Info/getInfoLabel.php?id=3483906&wwwpkgdb=fc2733d6468c073f1dd738aa598ede55&nocache=a9a895ad49f0f55c362d050dc1ad3fa3&dir=&wwwpkgdb=fc2733d6468c073f1dd738aa598ede55) | _ | putative sensor histidine kinase |
| [OCAR_7499](https://www.genoscope.cns.fr/agc/mage/wwwpkgdb/Info/getInfoLabel.php?id=3485775&wwwpkgdb=fc2733d6468c073f1dd738aa598ede55&nocache=a9a895ad49f0f55c362d050dc1ad3fa3&dir=&wwwpkgdb=fc2733d6468c073f1dd738aa598ede55) | _ | abc-type transporter, periplasmic component: haat family |
| [OCAR_7503](https://www.genoscope.cns.fr/agc/mage/wwwpkgdb/Info/getInfoLabel.php?id=3483910&wwwpkgdb=fc2733d6468c073f1dd738aa598ede55&nocache=a9a895ad49f0f55c362d050dc1ad3fa3&dir=&wwwpkgdb=fc2733d6468c073f1dd738aa598ede55) | _ | hypothetical protein |
| [OCAR_7506](https://www.genoscope.cns.fr/agc/mage/wwwpkgdb/Info/getInfoLabel.php?id=3485778&wwwpkgdb=fc2733d6468c073f1dd738aa598ede55&nocache=a9a895ad49f0f55c362d050dc1ad3fa3&dir=&wwwpkgdb=fc2733d6468c073f1dd738aa598ede55) | _ | hypothetical protein |
| [OCAR_7509](https://www.genoscope.cns.fr/agc/mage/wwwpkgdb/Info/getInfoLabel.php?id=3485781&wwwpkgdb=fc2733d6468c073f1dd738aa598ede55&nocache=a9a895ad49f0f55c362d050dc1ad3fa3&dir=&wwwpkgdb=fc2733d6468c073f1dd738aa598ede55) | _ | hypothetical protein |
| [OCAR_7516](https://www.genoscope.cns.fr/agc/mage/wwwpkgdb/Info/getInfoLabel.php?id=3483912&wwwpkgdb=fc2733d6468c073f1dd738aa598ede55&nocache=a9a895ad49f0f55c362d050dc1ad3fa3&dir=&wwwpkgdb=fc2733d6468c073f1dd738aa598ede55) | _ | hypothetical protein |
| [OCAR_7519](https://www.genoscope.cns.fr/agc/mage/wwwpkgdb/Info/getInfoLabel.php?id=3485788&wwwpkgdb=fc2733d6468c073f1dd738aa598ede55&nocache=a9a895ad49f0f55c362d050dc1ad3fa3&dir=&wwwpkgdb=fc2733d6468c073f1dd738aa598ede55) | _ | heavy metal transport/detoxification protein |
| [OCAR_3487](https://www.genoscope.cns.fr/agc/mage/wwwpkgdb/Info/getInfoLabel.php?id=3481920&wwwpkgdb=fc2733d6468c073f1dd738aa598ede55&nocache=a9a895ad49f0f55c362d050dc1ad3fa3&dir=&wwwpkgdb=fc2733d6468c073f1dd738aa598ede55) | _ | _ |
| [OCAR_7554](https://www.genoscope.cns.fr/agc/mage/wwwpkgdb/Info/getInfoLabel.php?id=3483937&wwwpkgdb=fc2733d6468c073f1dd738aa598ede55&nocache=a9a895ad49f0f55c362d050dc1ad3fa3&dir=&wwwpkgdb=fc2733d6468c073f1dd738aa598ede55) | _ | hypothetical protein |
| [OCAR_7556](https://www.genoscope.cns.fr/agc/mage/wwwpkgdb/Info/getInfoLabel.php?id=3483939&wwwpkgdb=fc2733d6468c073f1dd738aa598ede55&nocache=a9a895ad49f0f55c362d050dc1ad3fa3&dir=&wwwpkgdb=fc2733d6468c073f1dd738aa598ede55) | _ | hypothetical protein |
| [OCAR_7558](https://www.genoscope.cns.fr/agc/mage/wwwpkgdb/Info/getInfoLabel.php?id=3485801&wwwpkgdb=fc2733d6468c073f1dd738aa598ede55&nocache=a9a895ad49f0f55c362d050dc1ad3fa3&dir=&wwwpkgdb=fc2733d6468c073f1dd738aa598ede55) | _ | altronate hydrolase |
| [OCAR_7561](https://www.genoscope.cns.fr/agc/mage/wwwpkgdb/Info/getInfoLabel.php?id=3483941&wwwpkgdb=fc2733d6468c073f1dd738aa598ede55&nocache=a9a895ad49f0f55c362d050dc1ad3fa3&dir=&wwwpkgdb=fc2733d6468c073f1dd738aa598ede55) | _ | hypothetical protein |
| [OCAR_7568](https://www.genoscope.cns.fr/agc/mage/wwwpkgdb/Info/getInfoLabel.php?id=3483945&wwwpkgdb=fc2733d6468c073f1dd738aa598ede55&nocache=a9a895ad49f0f55c362d050dc1ad3fa3&dir=&wwwpkgdb=fc2733d6468c073f1dd738aa598ede55) | _ | hypothetical protein |
| [OCAR_7582](https://www.genoscope.cns.fr/agc/mage/wwwpkgdb/Info/getInfoLabel.php?id=3483956&wwwpkgdb=fc2733d6468c073f1dd738aa598ede55&nocache=a9a895ad49f0f55c362d050dc1ad3fa3&dir=&wwwpkgdb=fc2733d6468c073f1dd738aa598ede55) | _ | hypothetical protein |
| [OCAR_3548](https://www.genoscope.cns.fr/agc/mage/wwwpkgdb/Info/getInfoLabel.php?id=3481919&wwwpkgdb=fc2733d6468c073f1dd738aa598ede55&nocache=a9a895ad49f0f55c362d050dc1ad3fa3&dir=&wwwpkgdb=fc2733d6468c073f1dd738aa598ede55) | _ | _ |
| [OCAR_7597](https://www.genoscope.cns.fr/agc/mage/wwwpkgdb/Info/getInfoLabel.php?id=3483969&wwwpkgdb=fc2733d6468c073f1dd738aa598ede55&nocache=a9a895ad49f0f55c362d050dc1ad3fa3&dir=&wwwpkgdb=fc2733d6468c073f1dd738aa598ede55) | _ | hypothetical protein |
| [OCAR_7612](https://www.genoscope.cns.fr/agc/mage/wwwpkgdb/Info/getInfoLabel.php?id=3483970&wwwpkgdb=fc2733d6468c073f1dd738aa598ede55&nocache=a9a895ad49f0f55c362d050dc1ad3fa3&dir=&wwwpkgdb=fc2733d6468c073f1dd738aa598ede55) | _ | hypothetical protein |
| [OCAR_3574](https://www.genoscope.cns.fr/agc/mage/wwwpkgdb/Info/getInfoLabel.php?id=3481918&wwwpkgdb=fc2733d6468c073f1dd738aa598ede55&nocache=a9a895ad49f0f55c362d050dc1ad3fa3&dir=&wwwpkgdb=fc2733d6468c073f1dd738aa598ede55) | _ | _ |
| [OCAR_7615](https://www.genoscope.cns.fr/agc/mage/wwwpkgdb/Info/getInfoLabel.php?id=3483973&wwwpkgdb=fc2733d6468c073f1dd738aa598ede55&nocache=a9a895ad49f0f55c362d050dc1ad3fa3&dir=&wwwpkgdb=fc2733d6468c073f1dd738aa598ede55) | _ | hypothetical protein |
| [OCAR_7616](https://www.genoscope.cns.fr/agc/mage/wwwpkgdb/Info/getInfoLabel.php?id=3483974&wwwpkgdb=fc2733d6468c073f1dd738aa598ede55&nocache=a9a895ad49f0f55c362d050dc1ad3fa3&dir=&wwwpkgdb=fc2733d6468c073f1dd738aa598ede55) | _ | hypothetical protein |
| [OCAR_7618](https://www.genoscope.cns.fr/agc/mage/wwwpkgdb/Info/getInfoLabel.php?id=3485922&wwwpkgdb=fc2733d6468c073f1dd738aa598ede55&nocache=a9a895ad49f0f55c362d050dc1ad3fa3&dir=&wwwpkgdb=fc2733d6468c073f1dd738aa598ede55) | _ | heavy metal efflux pump; nonfunctional due to frameshift |
| [OCAR_7620](https://www.genoscope.cns.fr/agc/mage/wwwpkgdb/Info/getInfoLabel.php?id=3483976&wwwpkgdb=fc2733d6468c073f1dd738aa598ede55&nocache=a9a895ad49f0f55c362d050dc1ad3fa3&dir=&wwwpkgdb=fc2733d6468c073f1dd738aa598ede55) | _ | hypothetical protein |
| [OCAR_7619](https://www.genoscope.cns.fr/agc/mage/wwwpkgdb/Info/getInfoLabel.php?id=3485824&wwwpkgdb=fc2733d6468c073f1dd738aa598ede55&nocache=a9a895ad49f0f55c362d050dc1ad3fa3&dir=&wwwpkgdb=fc2733d6468c073f1dd738aa598ede55) | _ | hypothetical protein |
| [OCAR_7621](https://www.genoscope.cns.fr/agc/mage/wwwpkgdb/Info/getInfoLabel.php?id=3485925&wwwpkgdb=fc2733d6468c073f1dd738aa598ede55&nocache=a9a895ad49f0f55c362d050dc1ad3fa3&dir=&wwwpkgdb=fc2733d6468c073f1dd738aa598ede55) | _ | transposase of insertion sequence ISRm1 OrfA protein; nonfunctional due to frameshift |
| [OCAR_7622](https://www.genoscope.cns.fr/agc/mage/wwwpkgdb/Info/getInfoLabel.php?id=3483977&wwwpkgdb=fc2733d6468c073f1dd738aa598ede55&nocache=a9a895ad49f0f55c362d050dc1ad3fa3&dir=&wwwpkgdb=fc2733d6468c073f1dd738aa598ede55) | _ | putative membrane protein of unknown function with Acyltransferase 3 domain |
| [OCAR_7623](https://www.genoscope.cns.fr/agc/mage/wwwpkgdb/Info/getInfoLabel.php?id=3485825&wwwpkgdb=fc2733d6468c073f1dd738aa598ede55&nocache=a9a895ad49f0f55c362d050dc1ad3fa3&dir=&wwwpkgdb=fc2733d6468c073f1dd738aa598ede55) | _ | protein of unknown function, DUF |
| [OCAR_3587](https://www.genoscope.cns.fr/agc/mage/wwwpkgdb/Info/getInfoLabel.php?id=3481916&wwwpkgdb=fc2733d6468c073f1dd738aa598ede55&nocache=a9a895ad49f0f55c362d050dc1ad3fa3&dir=&wwwpkgdb=fc2733d6468c073f1dd738aa598ede55) | _ | _ |
| [OCAR_7625](https://www.genoscope.cns.fr/agc/mage/wwwpkgdb/Info/getInfoLabel.php?id=3485827&wwwpkgdb=fc2733d6468c073f1dd738aa598ede55&nocache=a9a895ad49f0f55c362d050dc1ad3fa3&dir=&wwwpkgdb=fc2733d6468c073f1dd738aa598ede55) | _ | putative multicopper oxidase domain |
| [OCAR_3590](https://www.genoscope.cns.fr/agc/mage/wwwpkgdb/Info/getInfoLabel.php?id=3482120&wwwpkgdb=fc2733d6468c073f1dd738aa598ede55&nocache=a9a895ad49f0f55c362d050dc1ad3fa3&dir=&wwwpkgdb=fc2733d6468c073f1dd738aa598ede55) | _ | _ |
| [OCAR_7627](https://www.genoscope.cns.fr/agc/mage/wwwpkgdb/Info/getInfoLabel.php?id=3485829&wwwpkgdb=fc2733d6468c073f1dd738aa598ede55&nocache=a9a895ad49f0f55c362d050dc1ad3fa3&dir=&wwwpkgdb=fc2733d6468c073f1dd738aa598ede55) | _ | GCN5-related N-acetyltransferase |
| [OCAR_3595](https://www.genoscope.cns.fr/agc/mage/wwwpkgdb/Info/getInfoLabel.php?id=3482119&wwwpkgdb=fc2733d6468c073f1dd738aa598ede55&nocache=a9a895ad49f0f55c362d050dc1ad3fa3&dir=&wwwpkgdb=fc2733d6468c073f1dd738aa598ede55) | _ | _ |
| [OCAR_7638](https://www.genoscope.cns.fr/agc/mage/wwwpkgdb/Info/getInfoLabel.php?id=3483979&wwwpkgdb=fc2733d6468c073f1dd738aa598ede55&nocache=a9a895ad49f0f55c362d050dc1ad3fa3&dir=&wwwpkgdb=fc2733d6468c073f1dd738aa598ede55) | _ | hypothetical protein |
| [OCAR_7640](https://www.genoscope.cns.fr/agc/mage/wwwpkgdb/Info/getInfoLabel.php?id=3485839&wwwpkgdb=fc2733d6468c073f1dd738aa598ede55&nocache=a9a895ad49f0f55c362d050dc1ad3fa3&dir=&wwwpkgdb=fc2733d6468c073f1dd738aa598ede55) | _ | hypothetical protein |
| [OCAR_7641](https://www.genoscope.cns.fr/agc/mage/wwwpkgdb/Info/getInfoLabel.php?id=3485840&wwwpkgdb=fc2733d6468c073f1dd738aa598ede55&nocache=a9a895ad49f0f55c362d050dc1ad3fa3&dir=&wwwpkgdb=fc2733d6468c073f1dd738aa598ede55) | _ | hypothetical protein |
| [OCAR_3608](https://www.genoscope.cns.fr/agc/mage/wwwpkgdb/Info/getInfoLabel.php?id=3482118&wwwpkgdb=fc2733d6468c073f1dd738aa598ede55&nocache=a9a895ad49f0f55c362d050dc1ad3fa3&dir=&wwwpkgdb=fc2733d6468c073f1dd738aa598ede55) | _ | _ |
| [OCAR_7643](https://www.genoscope.cns.fr/agc/mage/wwwpkgdb/Info/getInfoLabel.php?id=3485841&wwwpkgdb=fc2733d6468c073f1dd738aa598ede55&nocache=a9a895ad49f0f55c362d050dc1ad3fa3&dir=&wwwpkgdb=fc2733d6468c073f1dd738aa598ede55) | _ | replication protein C |
| [OCAR_7644](https://www.genoscope.cns.fr/agc/mage/wwwpkgdb/Info/getInfoLabel.php?id=3483982&wwwpkgdb=fc2733d6468c073f1dd738aa598ede55&nocache=a9a895ad49f0f55c362d050dc1ad3fa3&dir=&wwwpkgdb=fc2733d6468c073f1dd738aa598ede55) | _ | putative transcriptional regulator |
| [OCAR_7645](https://www.genoscope.cns.fr/agc/mage/wwwpkgdb/Info/getInfoLabel.php?id=3485842&wwwpkgdb=fc2733d6468c073f1dd738aa598ede55&nocache=a9a895ad49f0f55c362d050dc1ad3fa3&dir=&wwwpkgdb=fc2733d6468c073f1dd738aa598ede55) | _ | single-strand binding protein family |
| [OCAR_3613](https://www.genoscope.cns.fr/agc/mage/wwwpkgdb/Info/getInfoLabel.php?id=3482117&wwwpkgdb=fc2733d6468c073f1dd738aa598ede55&nocache=a9a895ad49f0f55c362d050dc1ad3fa3&dir=&wwwpkgdb=fc2733d6468c073f1dd738aa598ede55) | _ | _ |
| [OCAR_7646](https://www.genoscope.cns.fr/agc/mage/wwwpkgdb/Info/getInfoLabel.php?id=3485843&wwwpkgdb=fc2733d6468c073f1dd738aa598ede55&nocache=a9a895ad49f0f55c362d050dc1ad3fa3&dir=&wwwpkgdb=fc2733d6468c073f1dd738aa598ede55) | _ | replication protein C |
| [OCAR_7647](https://www.genoscope.cns.fr/agc/mage/wwwpkgdb/Info/getInfoLabel.php?id=3485844&wwwpkgdb=fc2733d6468c073f1dd738aa598ede55&nocache=a9a895ad49f0f55c362d050dc1ad3fa3&dir=&wwwpkgdb=fc2733d6468c073f1dd738aa598ede55) | _ | RepB |
| [OCAR_7648](https://www.genoscope.cns.fr/agc/mage/wwwpkgdb/Info/getInfoLabel.php?id=3485845&wwwpkgdb=fc2733d6468c073f1dd738aa598ede55&nocache=a9a895ad49f0f55c362d050dc1ad3fa3&dir=&wwwpkgdb=fc2733d6468c073f1dd738aa598ede55) | repA | plasmid partitioning protein RepA |
| [OCAR_3617](https://www.genoscope.cns.fr/agc/mage/wwwpkgdb/Info/getInfoLabel.php?id=3481914&wwwpkgdb=fc2733d6468c073f1dd738aa598ede55&nocache=a9a895ad49f0f55c362d050dc1ad3fa3&dir=&wwwpkgdb=fc2733d6468c073f1dd738aa598ede55) | _ | _ |
| [OCAR_7649](https://www.genoscope.cns.fr/agc/mage/wwwpkgdb/Info/getInfoLabel.php?id=3485846&wwwpkgdb=fc2733d6468c073f1dd738aa598ede55&nocache=a9a895ad49f0f55c362d050dc1ad3fa3&dir=&wwwpkgdb=fc2733d6468c073f1dd738aa598ede55) | _ | phage integrase |
| [OCAR_7650](https://www.genoscope.cns.fr/agc/mage/wwwpkgdb/Info/getInfoLabel.php?id=3485847&wwwpkgdb=fc2733d6468c073f1dd738aa598ede55&nocache=a9a895ad49f0f55c362d050dc1ad3fa3&dir=&wwwpkgdb=fc2733d6468c073f1dd738aa598ede55) | _ | hypothetical protein |
| [OCAR_3622](https://www.genoscope.cns.fr/agc/mage/wwwpkgdb/Info/getInfoLabel.php?id=3481913&wwwpkgdb=fc2733d6468c073f1dd738aa598ede55&nocache=a9a895ad49f0f55c362d050dc1ad3fa3&dir=&wwwpkgdb=fc2733d6468c073f1dd738aa598ede55) | _ | _ |
| [OCAR_7653](https://www.genoscope.cns.fr/agc/mage/wwwpkgdb/Info/getInfoLabel.php?id=3483983&wwwpkgdb=fc2733d6468c073f1dd738aa598ede55&nocache=a9a895ad49f0f55c362d050dc1ad3fa3&dir=&wwwpkgdb=fc2733d6468c073f1dd738aa598ede55) | _ | hypothetical protein |
| [OCAR_7654](https://www.genoscope.cns.fr/agc/mage/wwwpkgdb/Info/getInfoLabel.php?id=3483984&wwwpkgdb=fc2733d6468c073f1dd738aa598ede55&nocache=a9a895ad49f0f55c362d050dc1ad3fa3&dir=&wwwpkgdb=fc2733d6468c073f1dd738aa598ede55) | _ | ATPase |
| [OCAR_7655](https://www.genoscope.cns.fr/agc/mage/wwwpkgdb/Info/getInfoLabel.php?id=3483985&wwwpkgdb=fc2733d6468c073f1dd738aa598ede55&nocache=a9a895ad49f0f55c362d050dc1ad3fa3&dir=&wwwpkgdb=fc2733d6468c073f1dd738aa598ede55) | _ | Y4bN protein |
| [OCAR_7656](https://www.genoscope.cns.fr/agc/mage/wwwpkgdb/Info/getInfoLabel.php?id=3483986&wwwpkgdb=fc2733d6468c073f1dd738aa598ede55&nocache=a9a895ad49f0f55c362d050dc1ad3fa3&dir=&wwwpkgdb=fc2733d6468c073f1dd738aa598ede55) | _ | hypothetical protein |
| [OCAR_7657](https://www.genoscope.cns.fr/agc/mage/wwwpkgdb/Info/getInfoLabel.php?id=3485850&wwwpkgdb=fc2733d6468c073f1dd738aa598ede55&nocache=a9a895ad49f0f55c362d050dc1ad3fa3&dir=&wwwpkgdb=fc2733d6468c073f1dd738aa598ede55) | _ | hypothetical protein |
| [OCAR_7658](https://www.genoscope.cns.fr/agc/mage/wwwpkgdb/Info/getInfoLabel.php?id=3485851&wwwpkgdb=fc2733d6468c073f1dd738aa598ede55&nocache=a9a895ad49f0f55c362d050dc1ad3fa3&dir=&wwwpkgdb=fc2733d6468c073f1dd738aa598ede55) | _ | hypothetical protein |
| [OCAR_7659](https://www.genoscope.cns.fr/agc/mage/wwwpkgdb/Info/getInfoLabel.php?id=3485852&wwwpkgdb=fc2733d6468c073f1dd738aa598ede55&nocache=a9a895ad49f0f55c362d050dc1ad3fa3&dir=&wwwpkgdb=fc2733d6468c073f1dd738aa598ede55) | _ | helicase domain protein |
| [OCAR_7660](https://www.genoscope.cns.fr/agc/mage/wwwpkgdb/Info/getInfoLabel.php?id=3485853&wwwpkgdb=fc2733d6468c073f1dd738aa598ede55&nocache=a9a895ad49f0f55c362d050dc1ad3fa3&dir=&wwwpkgdb=fc2733d6468c073f1dd738aa598ede55) | _ | hypothetical protein |
| [OCAR_7661](https://www.genoscope.cns.fr/agc/mage/wwwpkgdb/Info/getInfoLabel.php?id=3485854&wwwpkgdb=fc2733d6468c073f1dd738aa598ede55&nocache=a9a895ad49f0f55c362d050dc1ad3fa3&dir=&wwwpkgdb=fc2733d6468c073f1dd738aa598ede55) | _ | hypothetical protein |
| [OCAR_7662](https://www.genoscope.cns.fr/agc/mage/wwwpkgdb/Info/getInfoLabel.php?id=3485855&wwwpkgdb=fc2733d6468c073f1dd738aa598ede55&nocache=a9a895ad49f0f55c362d050dc1ad3fa3&dir=&wwwpkgdb=fc2733d6468c073f1dd738aa598ede55) | _ | modification methylase XhoI |
| [OCAR_7665](https://www.genoscope.cns.fr/agc/mage/wwwpkgdb/Info/getInfoLabel.php?id=3483989&wwwpkgdb=fc2733d6468c073f1dd738aa598ede55&nocache=a9a895ad49f0f55c362d050dc1ad3fa3&dir=&wwwpkgdb=fc2733d6468c073f1dd738aa598ede55) | _ | hypothetical protein |
| [OCAR_7668](https://www.genoscope.cns.fr/agc/mage/wwwpkgdb/Info/getInfoLabel.php?id=3485858&wwwpkgdb=fc2733d6468c073f1dd738aa598ede55&nocache=a9a895ad49f0f55c362d050dc1ad3fa3&dir=&wwwpkgdb=fc2733d6468c073f1dd738aa598ede55) | _ | hypothetical protein |
| [OCAR_3639](https://www.genoscope.cns.fr/agc/mage/wwwpkgdb/Info/getInfoLabel.php?id=3481912&wwwpkgdb=fc2733d6468c073f1dd738aa598ede55&nocache=a9a895ad49f0f55c362d050dc1ad3fa3&dir=&wwwpkgdb=fc2733d6468c073f1dd738aa598ede55) | _ | _ |
| [OCAR_7669](https://www.genoscope.cns.fr/agc/mage/wwwpkgdb/Info/getInfoLabel.php?id=3485859&wwwpkgdb=fc2733d6468c073f1dd738aa598ede55&nocache=a9a895ad49f0f55c362d050dc1ad3fa3&dir=&wwwpkgdb=fc2733d6468c073f1dd738aa598ede55) | _ | hypothetical protein |
| [OCAR_7670](https://www.genoscope.cns.fr/agc/mage/wwwpkgdb/Info/getInfoLabel.php?id=3485860&wwwpkgdb=fc2733d6468c073f1dd738aa598ede55&nocache=a9a895ad49f0f55c362d050dc1ad3fa3&dir=&wwwpkgdb=fc2733d6468c073f1dd738aa598ede55) | _ | hypothetical protein |
| [OCAR_7671](https://www.genoscope.cns.fr/agc/mage/wwwpkgdb/Info/getInfoLabel.php?id=3485861&wwwpkgdb=fc2733d6468c073f1dd738aa598ede55&nocache=a9a895ad49f0f55c362d050dc1ad3fa3&dir=&wwwpkgdb=fc2733d6468c073f1dd738aa598ede55) | _ | hypothetical protein |
| [OCAR_7672](https://www.genoscope.cns.fr/agc/mage/wwwpkgdb/Info/getInfoLabel.php?id=3485862&wwwpkgdb=fc2733d6468c073f1dd738aa598ede55&nocache=a9a895ad49f0f55c362d050dc1ad3fa3&dir=&wwwpkgdb=fc2733d6468c073f1dd738aa598ede55) | _ | hypothetical protein |
| [OCAR_7673](https://www.genoscope.cns.fr/agc/mage/wwwpkgdb/Info/getInfoLabel.php?id=3485863&wwwpkgdb=fc2733d6468c073f1dd738aa598ede55&nocache=a9a895ad49f0f55c362d050dc1ad3fa3&dir=&wwwpkgdb=fc2733d6468c073f1dd738aa598ede55) | _ | chromosome partitioning protein |
| [OCAR_7674](https://www.genoscope.cns.fr/agc/mage/wwwpkgdb/Info/getInfoLabel.php?id=3485864&wwwpkgdb=fc2733d6468c073f1dd738aa598ede55&nocache=a9a895ad49f0f55c362d050dc1ad3fa3&dir=&wwwpkgdb=fc2733d6468c073f1dd738aa598ede55) | _ | N-6 DNA methylase |
| [OCAR_7675](https://www.genoscope.cns.fr/agc/mage/wwwpkgdb/Info/getInfoLabel.php?id=3485865&wwwpkgdb=fc2733d6468c073f1dd738aa598ede55&nocache=a9a895ad49f0f55c362d050dc1ad3fa3&dir=&wwwpkgdb=fc2733d6468c073f1dd738aa598ede55) | _ | hypothetical protein |
| [OCAR_7676](https://www.genoscope.cns.fr/agc/mage/wwwpkgdb/Info/getInfoLabel.php?id=3485866&wwwpkgdb=fc2733d6468c073f1dd738aa598ede55&nocache=a9a895ad49f0f55c362d050dc1ad3fa3&dir=&wwwpkgdb=fc2733d6468c073f1dd738aa598ede55) | _ | hypothetical protein |
| [OCAR_7677](https://www.genoscope.cns.fr/agc/mage/wwwpkgdb/Info/getInfoLabel.php?id=3485867&wwwpkgdb=fc2733d6468c073f1dd738aa598ede55&nocache=a9a895ad49f0f55c362d050dc1ad3fa3&dir=&wwwpkgdb=fc2733d6468c073f1dd738aa598ede55) | _ | hypothetical protein |
| [OCAR_7678](https://www.genoscope.cns.fr/agc/mage/wwwpkgdb/Info/getInfoLabel.php?id=3485868&wwwpkgdb=fc2733d6468c073f1dd738aa598ede55&nocache=a9a895ad49f0f55c362d050dc1ad3fa3&dir=&wwwpkgdb=fc2733d6468c073f1dd738aa598ede55) | _ | hypothetical protein |
| [OCAR_7679](https://www.genoscope.cns.fr/agc/mage/wwwpkgdb/Info/getInfoLabel.php?id=3485869&wwwpkgdb=fc2733d6468c073f1dd738aa598ede55&nocache=a9a895ad49f0f55c362d050dc1ad3fa3&dir=&wwwpkgdb=fc2733d6468c073f1dd738aa598ede55) | _ | hypothetical protein |
| [OCAR_3650](https://www.genoscope.cns.fr/agc/mage/wwwpkgdb/Info/getInfoLabel.php?id=3482116&wwwpkgdb=fc2733d6468c073f1dd738aa598ede55&nocache=a9a895ad49f0f55c362d050dc1ad3fa3&dir=&wwwpkgdb=fc2733d6468c073f1dd738aa598ede55) | _ | _ |
| [OCAR_3651](https://www.genoscope.cns.fr/agc/mage/wwwpkgdb/Info/getInfoLabel.php?id=3481911&wwwpkgdb=fc2733d6468c073f1dd738aa598ede55&nocache=a9a895ad49f0f55c362d050dc1ad3fa3&dir=&wwwpkgdb=fc2733d6468c073f1dd738aa598ede55) | _ | _ |
| [OCAR_3652](https://www.genoscope.cns.fr/agc/mage/wwwpkgdb/Info/getInfoLabel.php?id=3482115&wwwpkgdb=fc2733d6468c073f1dd738aa598ede55&nocache=a9a895ad49f0f55c362d050dc1ad3fa3&dir=&wwwpkgdb=fc2733d6468c073f1dd738aa598ede55) | _ | _ |
| [OCAR_7680](https://www.genoscope.cns.fr/agc/mage/wwwpkgdb/Info/getInfoLabel.php?id=3483990&wwwpkgdb=fc2733d6468c073f1dd738aa598ede55&nocache=a9a895ad49f0f55c362d050dc1ad3fa3&dir=&wwwpkgdb=fc2733d6468c073f1dd738aa598ede55) | _ | hypothetical protein |
| [OCAR_7681](https://www.genoscope.cns.fr/agc/mage/wwwpkgdb/Info/getInfoLabel.php?id=3483991&wwwpkgdb=fc2733d6468c073f1dd738aa598ede55&nocache=a9a895ad49f0f55c362d050dc1ad3fa3&dir=&wwwpkgdb=fc2733d6468c073f1dd738aa598ede55) | _ | RES domain superfamily |
| [OCAR_7683](https://www.genoscope.cns.fr/agc/mage/wwwpkgdb/Info/getInfoLabel.php?id=3483993&wwwpkgdb=fc2733d6468c073f1dd738aa598ede55&nocache=a9a895ad49f0f55c362d050dc1ad3fa3&dir=&wwwpkgdb=fc2733d6468c073f1dd738aa598ede55) | _ | hypothetical protein |
| [OCAR_7684](https://www.genoscope.cns.fr/agc/mage/wwwpkgdb/Info/getInfoLabel.php?id=3485870&wwwpkgdb=fc2733d6468c073f1dd738aa598ede55&nocache=a9a895ad49f0f55c362d050dc1ad3fa3&dir=&wwwpkgdb=fc2733d6468c073f1dd738aa598ede55) | _ | hypothetical protein |
| [OCAR_7685](https://www.genoscope.cns.fr/agc/mage/wwwpkgdb/Info/getInfoLabel.php?id=3485871&wwwpkgdb=fc2733d6468c073f1dd738aa598ede55&nocache=a9a895ad49f0f55c362d050dc1ad3fa3&dir=&wwwpkgdb=fc2733d6468c073f1dd738aa598ede55) | _ | hypothetical protein |
| [OCAR_7686](https://www.genoscope.cns.fr/agc/mage/wwwpkgdb/Info/getInfoLabel.php?id=3485872&wwwpkgdb=fc2733d6468c073f1dd738aa598ede55&nocache=a9a895ad49f0f55c362d050dc1ad3fa3&dir=&wwwpkgdb=fc2733d6468c073f1dd738aa598ede55) | _ | hypothetical protein |
| [OCAR_7687](https://www.genoscope.cns.fr/agc/mage/wwwpkgdb/Info/getInfoLabel.php?id=3485873&wwwpkgdb=fc2733d6468c073f1dd738aa598ede55&nocache=a9a895ad49f0f55c362d050dc1ad3fa3&dir=&wwwpkgdb=fc2733d6468c073f1dd738aa598ede55) | _ | putative lipoprotein |
| [OCAR_7688](https://www.genoscope.cns.fr/agc/mage/wwwpkgdb/Info/getInfoLabel.php?id=3485874&wwwpkgdb=fc2733d6468c073f1dd738aa598ede55&nocache=a9a895ad49f0f55c362d050dc1ad3fa3&dir=&wwwpkgdb=fc2733d6468c073f1dd738aa598ede55) | _ | nuclease |
| [OCAR_7689](https://www.genoscope.cns.fr/agc/mage/wwwpkgdb/Info/getInfoLabel.php?id=3485875&wwwpkgdb=fc2733d6468c073f1dd738aa598ede55&nocache=a9a895ad49f0f55c362d050dc1ad3fa3&dir=&wwwpkgdb=fc2733d6468c073f1dd738aa598ede55) | _ | single-strand binding protein family |
| [OCAR_7690](https://www.genoscope.cns.fr/agc/mage/wwwpkgdb/Info/getInfoLabel.php?id=3485876&wwwpkgdb=fc2733d6468c073f1dd738aa598ede55&nocache=a9a895ad49f0f55c362d050dc1ad3fa3&dir=&wwwpkgdb=fc2733d6468c073f1dd738aa598ede55) | _ | hypothetical protein |
| [OCAR_7691](https://www.genoscope.cns.fr/agc/mage/wwwpkgdb/Info/getInfoLabel.php?id=3485877&wwwpkgdb=fc2733d6468c073f1dd738aa598ede55&nocache=a9a895ad49f0f55c362d050dc1ad3fa3&dir=&wwwpkgdb=fc2733d6468c073f1dd738aa598ede55) | _ | hypothetical protein |
| [OCAR_7692](https://www.genoscope.cns.fr/agc/mage/wwwpkgdb/Info/getInfoLabel.php?id=3483994&wwwpkgdb=fc2733d6468c073f1dd738aa598ede55&nocache=a9a895ad49f0f55c362d050dc1ad3fa3&dir=&wwwpkgdb=fc2733d6468c073f1dd738aa598ede55) | _ | lytic transglycosylase, catalytic |
| [OCAR_7693](https://www.genoscope.cns.fr/agc/mage/wwwpkgdb/Info/getInfoLabel.php?id=3483995&wwwpkgdb=fc2733d6468c073f1dd738aa598ede55&nocache=a9a895ad49f0f55c362d050dc1ad3fa3&dir=&wwwpkgdb=fc2733d6468c073f1dd738aa598ede55) | _ | conjugal transfer protein, TrbC |
| [OCAR_7694](https://www.genoscope.cns.fr/agc/mage/wwwpkgdb/Info/getInfoLabel.php?id=3483996&wwwpkgdb=fc2733d6468c073f1dd738aa598ede55&nocache=a9a895ad49f0f55c362d050dc1ad3fa3&dir=&wwwpkgdb=fc2733d6468c073f1dd738aa598ede55) | _ | conjugal transfer protein |
| [OCAR_7695](https://www.genoscope.cns.fr/agc/mage/wwwpkgdb/Info/getInfoLabel.php?id=3483997&wwwpkgdb=fc2733d6468c073f1dd738aa598ede55&nocache=a9a895ad49f0f55c362d050dc1ad3fa3&dir=&wwwpkgdb=fc2733d6468c073f1dd738aa598ede55) | _ | type IV secretion/conjugal transfer ATPase, VirB4 family |
| [OCAR_7696](https://www.genoscope.cns.fr/agc/mage/wwwpkgdb/Info/getInfoLabel.php?id=3483998&wwwpkgdb=fc2733d6468c073f1dd738aa598ede55&nocache=a9a895ad49f0f55c362d050dc1ad3fa3&dir=&wwwpkgdb=fc2733d6468c073f1dd738aa598ede55) | _ | hypothetical protein |
| [OCAR_7697](https://www.genoscope.cns.fr/agc/mage/wwwpkgdb/Info/getInfoLabel.php?id=3483999&wwwpkgdb=fc2733d6468c073f1dd738aa598ede55&nocache=a9a895ad49f0f55c362d050dc1ad3fa3&dir=&wwwpkgdb=fc2733d6468c073f1dd738aa598ede55) | _ | conjugal transfer protein |
| [OCAR_7698](https://www.genoscope.cns.fr/agc/mage/wwwpkgdb/Info/getInfoLabel.php?id=3484000&wwwpkgdb=fc2733d6468c073f1dd738aa598ede55&nocache=a9a895ad49f0f55c362d050dc1ad3fa3&dir=&wwwpkgdb=fc2733d6468c073f1dd738aa598ede55) | _ | hypothetical protein |
| [OCAR_7699](https://www.genoscope.cns.fr/agc/mage/wwwpkgdb/Info/getInfoLabel.php?id=3484001&wwwpkgdb=fc2733d6468c073f1dd738aa598ede55&nocache=a9a895ad49f0f55c362d050dc1ad3fa3&dir=&wwwpkgdb=fc2733d6468c073f1dd738aa598ede55) | _ | putative lipoprotein |
| [OCAR_7700](https://www.genoscope.cns.fr/agc/mage/wwwpkgdb/Info/getInfoLabel.php?id=3484002&wwwpkgdb=fc2733d6468c073f1dd738aa598ede55&nocache=a9a895ad49f0f55c362d050dc1ad3fa3&dir=&wwwpkgdb=fc2733d6468c073f1dd738aa598ede55) | _ | TrbL/VirB6 plasmid conjugal transfer protein |
| [OCAR_7701](https://www.genoscope.cns.fr/agc/mage/wwwpkgdb/Info/getInfoLabel.php?id=3484003&wwwpkgdb=fc2733d6468c073f1dd738aa598ede55&nocache=a9a895ad49f0f55c362d050dc1ad3fa3&dir=&wwwpkgdb=fc2733d6468c073f1dd738aa598ede55) | _ | VirB8 |
| [OCAR_7702](https://www.genoscope.cns.fr/agc/mage/wwwpkgdb/Info/getInfoLabel.php?id=3484004&wwwpkgdb=fc2733d6468c073f1dd738aa598ede55&nocache=a9a895ad49f0f55c362d050dc1ad3fa3&dir=&wwwpkgdb=fc2733d6468c073f1dd738aa598ede55) | virB | P-type conjugative transfer protein VirB9 |
| [OCAR_7705](https://www.genoscope.cns.fr/agc/mage/wwwpkgdb/Info/getInfoLabel.php?id=3484007&wwwpkgdb=fc2733d6468c073f1dd738aa598ede55&nocache=a9a895ad49f0f55c362d050dc1ad3fa3&dir=&wwwpkgdb=fc2733d6468c073f1dd738aa598ede55) | _ | trag protein |
| [OCAR_7706](https://www.genoscope.cns.fr/agc/mage/wwwpkgdb/Info/getInfoLabel.php?id=3485878&wwwpkgdb=fc2733d6468c073f1dd738aa598ede55&nocache=a9a895ad49f0f55c362d050dc1ad3fa3&dir=&wwwpkgdb=fc2733d6468c073f1dd738aa598ede55) | _ | putative transporter |
| [OCAR_7709](https://www.genoscope.cns.fr/agc/mage/wwwpkgdb/Info/getInfoLabel.php?id=3484008&wwwpkgdb=fc2733d6468c073f1dd738aa598ede55&nocache=a9a895ad49f0f55c362d050dc1ad3fa3&dir=&wwwpkgdb=fc2733d6468c073f1dd738aa598ede55) | _ | hypothetical protein |
| [OCAR_7710](https://www.genoscope.cns.fr/agc/mage/wwwpkgdb/Info/getInfoLabel.php?id=3485881&wwwpkgdb=fc2733d6468c073f1dd738aa598ede55&nocache=a9a895ad49f0f55c362d050dc1ad3fa3&dir=&wwwpkgdb=fc2733d6468c073f1dd738aa598ede55) | _ | tetratricopeptide repeat domain protein |
| [OCAR_7711](https://www.genoscope.cns.fr/agc/mage/wwwpkgdb/Info/getInfoLabel.php?id=3484009&wwwpkgdb=fc2733d6468c073f1dd738aa598ede55&nocache=a9a895ad49f0f55c362d050dc1ad3fa3&dir=&wwwpkgdb=fc2733d6468c073f1dd738aa598ede55) | _ | hypothetical protein |
| [OCAR_3685](https://www.genoscope.cns.fr/agc/mage/wwwpkgdb/Info/getInfoLabel.php?id=3481910&wwwpkgdb=fc2733d6468c073f1dd738aa598ede55&nocache=a9a895ad49f0f55c362d050dc1ad3fa3&dir=&wwwpkgdb=fc2733d6468c073f1dd738aa598ede55) | _ | _ |
| [OCAR_7716](https://www.genoscope.cns.fr/agc/mage/wwwpkgdb/Info/getInfoLabel.php?id=3485884&wwwpkgdb=fc2733d6468c073f1dd738aa598ede55&nocache=a9a895ad49f0f55c362d050dc1ad3fa3&dir=&wwwpkgdb=fc2733d6468c073f1dd738aa598ede55) | _ | hypothetical protein |
| [OCAR_7718](https://www.genoscope.cns.fr/agc/mage/wwwpkgdb/Info/getInfoLabel.php?id=3485929&wwwpkgdb=fc2733d6468c073f1dd738aa598ede55&nocache=a9a895ad49f0f55c362d050dc1ad3fa3&dir=&wwwpkgdb=fc2733d6468c073f1dd738aa598ede55) | _ | transposase OrfA from IS3 family; nonfunctional due to frameshift |
| [OCAR_7720](https://www.genoscope.cns.fr/agc/mage/wwwpkgdb/Info/getInfoLabel.php?id=3484012&wwwpkgdb=fc2733d6468c073f1dd738aa598ede55&nocache=a9a895ad49f0f55c362d050dc1ad3fa3&dir=&wwwpkgdb=fc2733d6468c073f1dd738aa598ede55) | _ | iron permease FTR1 |
| [OCAR_7721](https://www.genoscope.cns.fr/agc/mage/wwwpkgdb/Info/getInfoLabel.php?id=3484013&wwwpkgdb=fc2733d6468c073f1dd738aa598ede55&nocache=a9a895ad49f0f55c362d050dc1ad3fa3&dir=&wwwpkgdb=fc2733d6468c073f1dd738aa598ede55) | _ | bile acid:sodium symporter |
| [OCAR_7722](https://www.genoscope.cns.fr/agc/mage/wwwpkgdb/Info/getInfoLabel.php?id=3484014&wwwpkgdb=fc2733d6468c073f1dd738aa598ede55&nocache=a9a895ad49f0f55c362d050dc1ad3fa3&dir=&wwwpkgdb=fc2733d6468c073f1dd738aa598ede55) | _ | bile acid:sodium symporter |
| [OCAR_7725](https://www.genoscope.cns.fr/agc/mage/wwwpkgdb/Info/getInfoLabel.php?id=3485889&wwwpkgdb=fc2733d6468c073f1dd738aa598ede55&nocache=a9a895ad49f0f55c362d050dc1ad3fa3&dir=&wwwpkgdb=fc2733d6468c073f1dd738aa598ede55) | _ | hypothetical protein |
| [OCAR_7729](https://www.genoscope.cns.fr/agc/mage/wwwpkgdb/Info/getInfoLabel.php?id=3485893&wwwpkgdb=fc2733d6468c073f1dd738aa598ede55&nocache=a9a895ad49f0f55c362d050dc1ad3fa3&dir=&wwwpkgdb=fc2733d6468c073f1dd738aa598ede55) | _ | hypothetical protein |
| [OCAR_7730](https://www.genoscope.cns.fr/agc/mage/wwwpkgdb/Info/getInfoLabel.php?id=3484015&wwwpkgdb=fc2733d6468c073f1dd738aa598ede55&nocache=a9a895ad49f0f55c362d050dc1ad3fa3&dir=&wwwpkgdb=fc2733d6468c073f1dd738aa598ede55) | _ | hypothetical protein |
| [OCAR_3708](https://www.genoscope.cns.fr/agc/mage/wwwpkgdb/Info/getInfoLabel.php?id=3481906&wwwpkgdb=fc2733d6468c073f1dd738aa598ede55&nocache=a9a895ad49f0f55c362d050dc1ad3fa3&dir=&wwwpkgdb=fc2733d6468c073f1dd738aa598ede55) | _ | _ |
| [OCAR_3709](https://www.genoscope.cns.fr/agc/mage/wwwpkgdb/Info/getInfoLabel.php?id=3482114&wwwpkgdb=fc2733d6468c073f1dd738aa598ede55&nocache=a9a895ad49f0f55c362d050dc1ad3fa3&dir=&wwwpkgdb=fc2733d6468c073f1dd738aa598ede55) | _ | _ |
| [OCAR_3710](https://www.genoscope.cns.fr/agc/mage/wwwpkgdb/Info/getInfoLabel.php?id=3481905&wwwpkgdb=fc2733d6468c073f1dd738aa598ede55&nocache=a9a895ad49f0f55c362d050dc1ad3fa3&dir=&wwwpkgdb=fc2733d6468c073f1dd738aa598ede55) | _ | _ |
| [OCAR_7731](https://www.genoscope.cns.fr/agc/mage/wwwpkgdb/Info/getInfoLabel.php?id=3485894&wwwpkgdb=fc2733d6468c073f1dd738aa598ede55&nocache=a9a895ad49f0f55c362d050dc1ad3fa3&dir=&wwwpkgdb=fc2733d6468c073f1dd738aa598ede55) | _ | putative membrane protein of unknown function |
| [OCAR_7732](https://www.genoscope.cns.fr/agc/mage/wwwpkgdb/Info/getInfoLabel.php?id=3485895&wwwpkgdb=fc2733d6468c073f1dd738aa598ede55&nocache=a9a895ad49f0f55c362d050dc1ad3fa3&dir=&wwwpkgdb=fc2733d6468c073f1dd738aa598ede55) | _ | hypothetical protein |
| [OCAR_7734](https://www.genoscope.cns.fr/agc/mage/wwwpkgdb/Info/getInfoLabel.php?id=3485897&wwwpkgdb=fc2733d6468c073f1dd738aa598ede55&nocache=a9a895ad49f0f55c362d050dc1ad3fa3&dir=&wwwpkgdb=fc2733d6468c073f1dd738aa598ede55) | _ | hypothetical protein |
| [OCAR_7736](https://www.genoscope.cns.fr/agc/mage/wwwpkgdb/Info/getInfoLabel.php?id=3485898&wwwpkgdb=fc2733d6468c073f1dd738aa598ede55&nocache=a9a895ad49f0f55c362d050dc1ad3fa3&dir=&wwwpkgdb=fc2733d6468c073f1dd738aa598ede55) | _ | putative exported protein of unknown function |
| [OCAR_7737](https://www.genoscope.cns.fr/agc/mage/wwwpkgdb/Info/getInfoLabel.php?id=3485899&wwwpkgdb=fc2733d6468c073f1dd738aa598ede55&nocache=a9a895ad49f0f55c362d050dc1ad3fa3&dir=&wwwpkgdb=fc2733d6468c073f1dd738aa598ede55) | _ | hypothetical protein |
| [OCAR_7742](https://www.genoscope.cns.fr/agc/mage/wwwpkgdb/Info/getInfoLabel.php?id=3485904&wwwpkgdb=fc2733d6468c073f1dd738aa598ede55&nocache=a9a895ad49f0f55c362d050dc1ad3fa3&dir=&wwwpkgdb=fc2733d6468c073f1dd738aa598ede55) | _ | hypothetical protein |
| [OCAR_7745](https://www.genoscope.cns.fr/agc/mage/wwwpkgdb/Info/getInfoLabel.php?id=3484016&wwwpkgdb=fc2733d6468c073f1dd738aa598ede55&nocache=a9a895ad49f0f55c362d050dc1ad3fa3&dir=&wwwpkgdb=fc2733d6468c073f1dd738aa598ede55) | _ | hypothetical protein |
| [OCAR_7750](https://www.genoscope.cns.fr/agc/mage/wwwpkgdb/Info/getInfoLabel.php?id=3484021&wwwpkgdb=fc2733d6468c073f1dd738aa598ede55&nocache=a9a895ad49f0f55c362d050dc1ad3fa3&dir=&wwwpkgdb=fc2733d6468c073f1dd738aa598ede55) | arsH1 | arsenical resistance protein ArsH |
| [OCAR_7752](https://www.genoscope.cns.fr/agc/mage/wwwpkgdb/Info/getInfoLabel.php?id=3485908&wwwpkgdb=fc2733d6468c073f1dd738aa598ede55&nocache=a9a895ad49f0f55c362d050dc1ad3fa3&dir=&wwwpkgdb=fc2733d6468c073f1dd738aa598ede55) | _ | mercuric transport protein periplasmic component |
| [OCAR_7753](https://www.genoscope.cns.fr/agc/mage/wwwpkgdb/Info/getInfoLabel.php?id=3485909&wwwpkgdb=fc2733d6468c073f1dd738aa598ede55&nocache=a9a895ad49f0f55c362d050dc1ad3fa3&dir=&wwwpkgdb=fc2733d6468c073f1dd738aa598ede55) | _ | mercuric transport protein |
| [OCAR_7757](https://www.genoscope.cns.fr/agc/mage/wwwpkgdb/Info/getInfoLabel.php?id=3485911&wwwpkgdb=fc2733d6468c073f1dd738aa598ede55&nocache=a9a895ad49f0f55c362d050dc1ad3fa3&dir=&wwwpkgdb=fc2733d6468c073f1dd738aa598ede55) | _ | hypothetical protein |
| [OCAR_7761](https://www.genoscope.cns.fr/agc/mage/wwwpkgdb/Info/getInfoLabel.php?id=3484025&wwwpkgdb=fc2733d6468c073f1dd738aa598ede55&nocache=a9a895ad49f0f55c362d050dc1ad3fa3&dir=&wwwpkgdb=fc2733d6468c073f1dd738aa598ede55) | _ | hypothetical protein |
| [OCAR_7763](https://www.genoscope.cns.fr/agc/mage/wwwpkgdb/Info/getInfoLabel.php?id=3484027&wwwpkgdb=fc2733d6468c073f1dd738aa598ede55&nocache=a9a895ad49f0f55c362d050dc1ad3fa3&dir=&wwwpkgdb=fc2733d6468c073f1dd738aa598ede55) | _ | major facilitator superfamily MFS_1 |
| [OCAR_7765](https://www.genoscope.cns.fr/agc/mage/wwwpkgdb/Info/getInfoLabel.php?id=3484029&wwwpkgdb=fc2733d6468c073f1dd738aa598ede55&nocache=a9a895ad49f0f55c362d050dc1ad3fa3&dir=&wwwpkgdb=fc2733d6468c073f1dd738aa598ede55) | _ | thioredoxin/glutaredoxin |
| [OCAR_7767](https://www.genoscope.cns.fr/agc/mage/wwwpkgdb/Info/getInfoLabel.php?id=3484031&wwwpkgdb=fc2733d6468c073f1dd738aa598ede55&nocache=a9a895ad49f0f55c362d050dc1ad3fa3&dir=&wwwpkgdb=fc2733d6468c073f1dd738aa598ede55) | merP | mercuric transport protein periplasmic component |
| [OCAR_3748](https://www.genoscope.cns.fr/agc/mage/wwwpkgdb/Info/getInfoLabel.php?id=3481904&wwwpkgdb=fc2733d6468c073f1dd738aa598ede55&nocache=a9a895ad49f0f55c362d050dc1ad3fa3&dir=&wwwpkgdb=fc2733d6468c073f1dd738aa598ede55) | _ | _ |
| [OCAR_7769](https://www.genoscope.cns.fr/agc/mage/wwwpkgdb/Info/getInfoLabel.php?id=3484033&wwwpkgdb=fc2733d6468c073f1dd738aa598ede55&nocache=a9a895ad49f0f55c362d050dc1ad3fa3&dir=&wwwpkgdb=fc2733d6468c073f1dd738aa598ede55) | _ | hypothetical protein |
| [OCAR_3751](https://www.genoscope.cns.fr/agc/mage/wwwpkgdb/Info/getInfoLabel.php?id=3481903&wwwpkgdb=fc2733d6468c073f1dd738aa598ede55&nocache=a9a895ad49f0f55c362d050dc1ad3fa3&dir=&wwwpkgdb=fc2733d6468c073f1dd738aa598ede55) | _ | _ |
| [OCAR_7772](https://www.genoscope.cns.fr/agc/mage/wwwpkgdb/Info/getInfoLabel.php?id=3484036&wwwpkgdb=fc2733d6468c073f1dd738aa598ede55&nocache=a9a895ad49f0f55c362d050dc1ad3fa3&dir=&wwwpkgdb=fc2733d6468c073f1dd738aa598ede55) | _ | hypothetical protein |
| [OCAR_3754](https://www.genoscope.cns.fr/agc/mage/wwwpkgdb/Info/getInfoLabel.php?id=3481902&wwwpkgdb=fc2733d6468c073f1dd738aa598ede55&nocache=a9a895ad49f0f55c362d050dc1ad3fa3&dir=&wwwpkgdb=fc2733d6468c073f1dd738aa598ede55) | _ | _ |
| [OCAR_7775](https://www.genoscope.cns.fr/agc/mage/wwwpkgdb/Info/getInfoLabel.php?id=3484039&wwwpkgdb=fc2733d6468c073f1dd738aa598ede55&nocache=a9a895ad49f0f55c362d050dc1ad3fa3&dir=&wwwpkgdb=fc2733d6468c073f1dd738aa598ede55) | _ | hypothetical protein |
| [OCAR_7779](https://www.genoscope.cns.fr/agc/mage/wwwpkgdb/Info/getInfoLabel.php?id=3484043&wwwpkgdb=fc2733d6468c073f1dd738aa598ede55&nocache=a9a895ad49f0f55c362d050dc1ad3fa3&dir=&wwwpkgdb=fc2733d6468c073f1dd738aa598ede55) | _ | hypothetical protein |
