# Supplementary material for: Complete genome and comparative analysis of the chemolithoautotrophic bacterium Oligotropha carboxidovorans OM5
Source: BMC Genomics. 2010 Sep 23;11:511. doi: 10.1186/1471-2164-11-511 (PMC3091675; doi:10.1186/1471-2164-11-511)
Supplement: Additional file 3 — O. carboxidovorans enzyme complexes involved in some metabolic pathways. [file 1471-2164-11-511-S3.DOC]

**Additional file 3.** *O. carboxidovorans* enzyme complexes involved in some metabolic pathways

| **Enzyme complexes** | **Gene symbol** | ***O. carboxidovorans* Locus tag** |
| --- | --- | --- |
|  |  |  |
| **Oxidative phosphorylation (Kegg pathway oca00190)** | | |
| F type ATPase | Beta/ atpD, alpha/atpA, gamma/atpG, delta/atpH, epsilon/atpC, c, atpB/a, b | OCAR_4595, OCAR_4592, OCAR_4593, OCAR_4591, OCAR_4596,OCAR_4699, OCAR_4700, OCAR_4697 |
| cytochrome c reductase | ISP/petA, cyt b, cyt 1 | OCAR_6996, OCAR_6995, OCAR_6995 |
| succinate dehydrogenase/ fumarate reductase | sdhC, sdhD, sdhA, sdhB | OCAR_4287, OCAR_4288, OCAR_4289, OCAR_4290 |
| cytochrome c oxidase | cyoE, coxC, coax/ctaD, coxB, cox11/ctaG, cox15 | OCAR_4687, OCAR_4690, OCAR_4686, OCAR_4684, OCAR_4689, OCAR_5890 |
| cytochrome c oxidase (cbb3 type) | I/ccoN, II/ccoO, IV/ccoQ, III/ccoP | OCAR_7138, OCAR_7139, OCAR_7346, OCAR_7141 |
| cytochrome bd complex | cydA, B | OCAR_4639,OCAR_4638 |
| NADH dehydrogenase | nuoA-N | OCAR_5922-OCAR_5936 |
|  |  |  |
| **Krebs cycle (Kegg pathway: oca00020)** | | |
| phosphoenolpyruvate carboxykinase | pckA | OCAR_7573 |
| pyruvate dehydrogenase complex dihydrolipoamide acetyltransferase |  | OCAR_6284 |
| pyruvate dehydrogenase E1 component subunit beta |  | OCAR_6285 |
| dihydrolipoyl dehydrogenase | lpdA | OCAR_4585 |
| malate dehydrogenase |  | OCAR_4581 |
| citrate (Si)-synthase | gltA | OCAR_5971 |
| fumarate hydratase, class II | fumC | OCAR_5274 |
| aconitate hydratase 1 | acnA | OCAR_4571 |
| Succinate dehydrogenase cytochrome b556 subunit | sdhC | OCAR_4287 |
| succinyl-CoA synthetase beta chain |  | OCAR_6554 |
| dihydrolipoyllysine-residue succinyltransferase | sucB | OCAR_4583 |
| dihydrolipoyl dehydrogenase | lpdA | OCAR_4585 |
| oxoglutarate dehydrogenase | sucA | OCAR_4582 |
| isocitrate dehydrogenase, NADP-dependent |  | OCAR_5440 |
|  |  |  |
| **Glyoxylate and dicarboxylate metabolism (Kegg pathway: oca00630)** | | |
| citrate synthase | gltA | OCAR_5971 |
| aconitate hydratase 1 | acnA | OCAR_4571 |
| tartrate dehydrogenase |  | OCAR_5921 |
| malate dehydrogenase |  | OCAR_4581 |
| malate synthase G | glcB | OCAR_7393 |
| gamma subunit of formate dehydrogenase |  | OCAR_5161 |
| bifunctional protein FolD |  | OCAR_4309 |
| formyltetrahydrofolate deformylase | purU | OCAR_5315 |
| 2-hydroxy-3-oxopropionate reductase |  | OCAR_6560 |
| glyoxylate reductase |  | OCAR_4494 |
| putative hydroxypyruvate reductase |  | OCAR_7443 |
| hydroxypyruvate isomerase | hyi | OCAR_6561 |
| glyoxylate carboligase | gcl | OCAR_6562 |
| phosphoglycolate phosphatase | gph | OCAR_6530 |
| glycolate oxidase iron-sulfur subunit |  | OCAR_7281 |
| isocitrate lyase |  | OCAR_7022 |
|  |  |  |
| **Fatty acid oxidation (Kegg pathway: oca00071)** | | |
| long-chain-fatty-acid--CoA ligase |  | OCAR_7378 |
| acyl-coenzyme a dehydrogenase | acdh | OCAR_5221 |
| fatty acid oxidation complex subunit alpha |  | OCAR_5223 |
| glutaryl-CoA dehydrogenase | gcd | OCAR_6920 |
| acetyl-CoA acetyltransferase |  | OCAR_4107 |
| S-(hydroxymethyl)glutathione dehydrogenase/class III alcohol dehydrogenase |  | OCAR_5733 |
